# Epigenetic loss of the RNA decapping enzyme NUDT16 mediates C-MYC activation in T-cell acute lymphoblastic leukemia

**DOI:** 10.1038/leu.2017.99

**Published:** 2017-04-11

**Authors:** C Anadón, G van Tetering, H J Ferreira, C Moutinho, A Martínez-Cardús, A Villanueva, M Soler, H Heyn, S Moran, M Castro de Moura, F Setien, A Vidal, E Genescà, J M Ribera, J F Nomdedeu, S Guil, M Esteller

**Affiliations:** 1 Cancer Epigenetics and Biology Program, Bellvitge Biomedical Research Institute (IDIBELL), L’Hospitalet, Barcelona, Spain; 2 Translational Research Laboratory, Catalan Institute of Oncology, Bellvitge Biomedical Research Institute (IDIBELL), L’Hospitalet, Barcelona, Spain; 3 Department of Pathological Anatomy, Bellvitge University Hospital, Bellvitge Biomedical Research Institute (IDIBELL), L’Hospitalet, Barcelona, Spain; 4 Hematology Department, Catalan Institute of Oncology, Hospital Germans Trias i Pujol, Josep Carreras Leukaemia Research Institute (IJC), Universitat Autònoma de Barcelona, Barcelona, Spain; 5 Department of Haematology, Hospital de la Santa Creu i Sant Pau, Barcelona, Spain; 6 Physiological Sciences Department, School of Medicine and Health Sciences, University of Barcelona (UB), Barcelona, Spain; 7 Institucio Catalana de Recerca i Estudis Avançats (ICREA), Barcelona, Spain

It is possible that the occurrence of intrinsic defects in RNA processing pathways, such as RNA decapping,^1, 2, 3^ contribute to the distorted RNA landscapes of cancer cells. After transcription by RNA polymerase II, RNA molecules are equipped with a 5'-end N7-methyl guanosine (m7G)-cap. This m7G-cap is essential for translation, stabilizing the RNA molecule and protecting it from exonucleolytic breakdown.^1, 2, 3^ For RNA decay to occur the m7G-cap first needs to be removed. This process is known as decapping.^1, 2, 3^ The decapping mRNA 2 (DCP2) enzyme,^4^ also known as the nucleoside diphosphate-linked moiety X motif 20 (NUDT20), was originally thought to be the only mammalian RNA decapping enzyme with multiple cofactors controlling its activity.^1, 2, 3^ DCP2 is a member of the Nudix superfamily of hydrolase proteins, which predominantly catalyze the hydrolysis of a wide range of small nucleotide substrates composed of a nucleoside diphosphate linked to another moiety X.^5^ Another Nudix member, nudix hydrolase 16 (NUDT16), has recently been shown also to have mRNA decapping activity.^6, 7, 8^

Although genetic defects in DCP2 and NUDT16 have not been reported, transcriptional silencing by promoter CpG island hypermethylation is another mechanism for inactivating genes in human cancer.^9^ Thus, we studied whether DNA methylation-associated silencing of these RNA decapping enzymes occurred in tumorigenesis. We first screened a collection of 1001 human cancer cell lines in which we have recently determined the DNA methylation status of 450 000 CpG sites^10^ for the presence of DCP2 and NUDT16 promoter CpG island methylation (Supplementary Methods). The DCP2 promoter-associated CpG island was found unmethylated in the vast majority of cell lines, with a few examples of hypermethylated CpGs without enrichment in any specific cell type (Supplementary Table S1). In contrast, the NUDT16 promoter CpG island was methylated in 73% (19 of 26) of the T-cell-derived leukemia and lymphoma cell lines included in the studied set (Supplementary Table S1; Figure 1a). Data mining from microarray expression results^10^ showed that NUTD16 hypermethylation was associated with transcript downregulation (Figure 1a). Genomic data available at the COSMIC database (http://cancer.sanger.ac.uk/cosmic) did not show the presence of NUDT16 mutations and deletions in the mentioned cell lines. Beyond T-cell-derived malignancies, NUDT16 promoter CpG island was found mostly to be unmethylated in all the other tumor types available in our data set, with the exception of osteosarcoma (6 of 40, 15%), B-cell-derived leukemia and lymphoma (8 of 60, 13.3%), and myeloid-derived malignancies (3 of 41, 7.3% Supplementary Table S1; Figure 1a). The NUDT16 and DCP2 promoter CpG islands were unmethylated in all the normal human tissues studied, including T-lymphocytes isolated from healthy individuals (Supplementary Figure S1).

Having found the aforementioned NUDT16 CpG island methylation profiles, we studied in greater detail their association with the possible transcriptional inactivation of the NUDT16 gene at the RNA and protein levels in leukemia cell lines. We first performed bisulfite genomic sequencing of mutiple clones in the T-cell Acute Lymphoblastic Leukemia (T-ALL) cell lines CCRF-CEM, Jurkat, MOLT-4 and MOLT-16 using primers that encompassed the transcription start site-associated CpG island and confirmed the hypermethylated status of the 5′-end region of NUDT16 in comparison to normal T lymphocytes (Figure 1b), validating the DNA methylation patterns obtained by the microarray approach (Supplementary Figure S2). In contrast, normal T lymphocytes, the T-ALL cell lines KOPN-8, REH and RS4;11 and the leukemia cell lines HL-60 and K562 derived from myeloid lineage were all found to be unmethylated (Figure 1b; Supplementary Figure S2). Most notably, the NUDT16-hypermethylated T-ALL cell lines CCRF-CEM, Jurkat, MOLT-4 and MOLT-16 minimally expressed the NUDT16 RNA transcript, as determined by quantitative real-time PCR (Figure 1c), and protein, as assessed by western blot (Figure 1c). Conversely, T-ALL cell lines unmethylated at the NUDT16 promoter (KOPN-8, REH and RS4;11) or unmethylated leukemia cell lines from myeloid lineage (HL-60 and K562) expressed NUDT16 RNA and protein (Figure 1c). Treatment of the T-ALL methylated cell lines with the DNA-demethylating agent 5-aza-2-deoxycytidine restored NUDT16 expression (Figure 1c).

We also sought to demonstrate that the NUDT16 hypermethylation was not a specific feature of *in vitro*-grown T-ALL cell lines and that it also occurred in primary T-ALL patients. Herein, we performed methylation-specific PCR analyses to determine the NUDT16 CpG island methylation status in 51 primary T-ALLs (Supplementary Figure S2). We observed NUDT16 promoter CpG island hypermethylation in 60.7% (31 of 51) of these T-ALL cases. We did not find any correlation between NUDT16 CpG island methylation status and the clinicopathological characteristics of the studied primary T-ALLs (Supplementary Table S2). NOTCH1 mutations were observed in 30% (12 of 40) of T-ALL patients where additional DNA was available for further testing (Supplementary Figure S3), and we did not observe any association with the NUDT16 methylation status (Supplementary Table S2). We did not detect any mutation in the CNOT3 gene (Supplementary Figure S4). Supplementary Table S3 shows the NUDT16 methylation and NOTCH1 mutational status, and the clinicopathological characteristics for each one of the studied T-ALL patients. RNA was available for five T-ALL samples, including one methylated case. Using quantitative real-time PCR, NUDT16 expression was found in the four unmethylated samples, whereas the NUDT16-hypermethylated sample did not show detectable transcript levels (Supplementary Figure S5). Data mining of a small set of primary T-ALL cases interrogated by another DNA methylation microarray platform and expression microarrays (Supplementary Methods) confirmed our results by showing that NUDT16 hypermethylation occurred in 58.8% (10 of 17) of cases in association with transcript downregulation (Supplementary Figure S5).

Once we had shown the presence of NUDT16 CpG island hypermethylation-associated transcriptional loss in T-ALL, we studied its contribution to the leukemogenic phenotype *in vitro* and *in vivo*. We first analyzed the effect of the restoration of NUDT16 expression in the T-ALL-hypermethylated cell lines MOLT-4 and MOLT-16. Upon efficient transduction of NUDT16, we observed a significant reduction in cellular growth measured by the 3-(4,5-dimethyl-2-thiazolyl)-2,5-diphenyl-2H-tetrazolium bromide (MTT) assay in comparison with empty vector-transduced cells (Figure 2a; Supplementary Figure S6). These *in vitro* data were then translated to an *in vivo* model. We tested the ability of NUDT16-transduced MOLT-4 and MOLT-16 cells to form subcutaneous tumors in nude mice compared with empty vector-transduced cells. T-ALL cells with restored expression of NUDT16 showed lower tumorigenicity than MOLT-4 and MOLT-16 empty vector-derived tumors, as shown by measuring the increase in tumor volume (Figure 2a; Supplementary Figure S6). Tumor samples obtained at the end point of the subcutaneous nude mouse experiment showed that tumor weights and volumes of the NUDT16-transduced cells were lower than those observed in empty vector-transduced cells (Figure 2a; Supplementary Figure S6).

Finally, we wondered about the molecular mechanisms involved in the anti-leukemogenic phenotype mediated by NUDT16. In this regard, it is possible that the epigenetic defect of NUDT16 can prevent the decapping and subsequent decay of target RNAs involved in cellular transformation. To address this, we first studied the intracellular localization of the NUDT16 protein using nuclear compared with cytoplasmic fractioning followed by western blot. NUDT16-hypermethylated leukemia cell lines (CCRF-CEM, Jurkat, MOLT-4 and MOLT-16), as expected, showed no evidence of the protein either in the cytosolic or the nuclear compartments (Figure 2b). However, in unmethylated leukemia cell lines (KOPN-8, HL-60 and K562), the NUDT16 protein was present exclusively in the cytosolic fraction, as expected for an RNA decapping enzyme (Figure 2b). Notably, MOLT-4 cells in which we restored NUDT16 expression, upon transduction, mimicked the pattern of NUDT16-unmethylated cells by expressing the protein in the cytosolic fraction (Supplementary Figure S7). We next sought to identify those RNA targets that avoided the normal decapping in our hypermethylated T-ALL model. To accomplish this, we performed the RNA-immunoprecipitation of m3G/m7G-capped transcripts^11^ in empty vector compared with NUDT16-transduced MOLT-4 cells, followed by hybridization of the immunoprecipitated RNA to an RNA expression microarray (Supplementary Methods; Supplementary Table S4). The expression microarray data have been deposited in the Gene Expression Omnibus (GEO) repository under Accession Number GSE84973. Among the set of transcripts derived from the RNA-immunoprecipitation experiment that were found to be decreased upon NUDT16 restoration in MOLT-4 cells, and thus putative direct substrates of the decapping activity of the enzyme, we found many genes with known anti-apoptotic or pro-proliferative activities, such as BCL11A, MAP3K2 and GSK3B, and the diminished stability of these transcripts upon NUDT16 transduction observed in microarrays was validated by quantitative real-time PCR (Supplementary Figure S7). However, our attention was particularly caught by the presence of the transcripts for FBXO28^12^ and USP37,^13^ two proteins that, through altered ubiquitylation, stabilize C-MYC, a key oncogene in the natural history of T-ALL.^14, 15^ We observed that the restoration of NUDT16 expression in MOLT-4 cells reduced the levels of both the RNA transcripts in Actinomycin D-treated cells (Figure 2c), and the western blot assays showed a reduction in levels of the FBXO28 and USP37 proteins upon NUDT16 transduction (Figure 2c). Most notably, the decay of the FBXO28 and USP37 transcripts upon NUDT16 restoration in MOLT-4 cells was also associated with a reduction in C-MYC protein levels (Figure 2c), the target of their ubiquitylation-related activities. Similar results were found in the MOLT-16 cell line where NUDT16 transduction reduced FBX028 levels (even in the absence of Actinomycin D; Supplementary Figure S8), decreased C-MYC ubiquitylation^12^ and diminished C-MYC protein levels (Supplementary Figure S8), supporting that the loss of FBXO28-dependent MYC ubiquitylation results in enhanced protein degradation, as it has been reported.^12^ Furthermore, Actinomycin D treatment results in almost a complete loss of the C-MYC protein (Supplementary Figure S8).

Overall, our data suggest the existence in T-ALL of a disrupted RNA decapping pathway, mediated by the DNA methylation-associated loss of NUDT16, which contributes to the natural history of the disease by stabilizing transforming factors, such as is the case of the leukemogenic protein C-MYC.

## Figures and Tables

**Figure 1:**
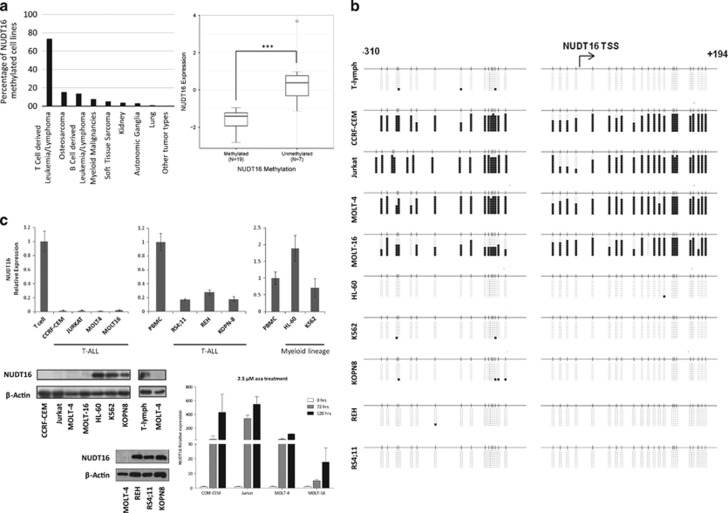
DNA methylation-associated transcriptional silencing of NUDT16 in transformed cells. (**a**) Percentage of NUDT16 methylation in the Sanger panel of cancer cell lines by tumor type. Tumor type sample sizes studied: T-cell-derived leukemia and lymphoma (*n*=26), osteosarcoma (*n*=40), B-cell-derived leukemia and lymphoma (*n*=57), myeloid-derived malignancies (*n*=35), soft tissue sarcoma (*n*=20), kidney (*n*=28), autonomic ganglia (*n*=31), lung (*n*=163), and other tumor types (*n*=602). Right, NUDT16 methylation is associated with loss of the transcript in the T-cell derived cell lines from Sanger. The box plots illustrate the distribution of expression values; the central solid line indicates the median; the limits of the box show the upper and lower percentiles. Mann–Whitney *U-*test, ***P<0.0001. (**b**) Bisulfite genomic sequencing of NUDT16 promoter CpG Island. CpG dinucleotides are represented as short vertical lines and the transcription start site (TSS) is represented as a long black arrow. Eight single clones are shown for each sample. Presence of a methylated or unmethylated cytosine is indicated by a black or a white square, respectively. (**c**) Top, expression levels of the NUDT16 transcript determined by real-time reverse transcription PCR. Data shown represent mean±s.d. of biological triplicates; below, expression levels of the NUDT16 protein determined by western blot; right, the expression of the NUDT16 RNA transcript was restored in the CCRF-CEM, Jurkat, MOLT-4 and MOLT-16 cells by treatment with the demethylating drug 5-aza-2’-deoxycytidine. Data shown represent the mean±s.d. of biological triplicates.

**Figure 2:**
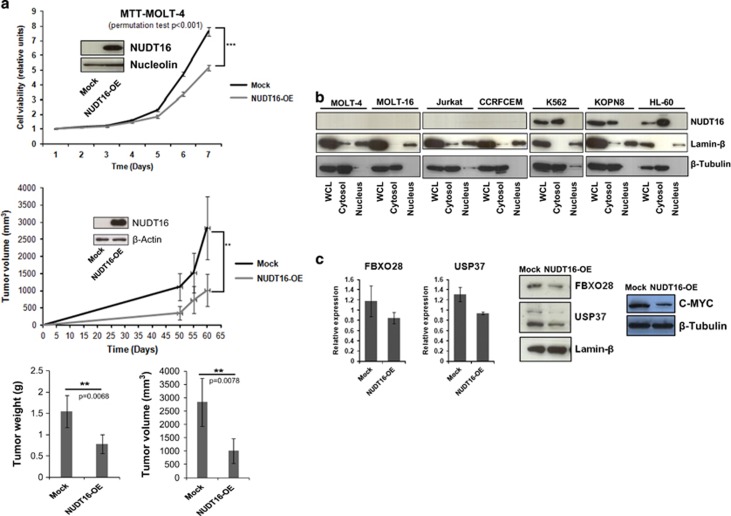
NUDT16 exerts growth-inhibitory effects and destabilizes mRNA targets that regulate MYC protein stability. (**a**) Top, restoration of NUDT16 protein expression upon stable transduction in MOLT-4 cells (NUDT16-OE) was associated with reduced viability in the 3-(4,5-dimethyl-2-thiazolyl)-2,5-diphenyl-2H-tetrazolium bromide (MTT) assay in comparison to empty vector-transduced cells. Probabilities are those from permutation test; middle, tumor volume monitoring of xenograft samples derived from NUDT16 MOLT-4 cells transduced with NUDT16 or the empty vector. ***P<0.001, **P<0.01. Restoration of NUDT16 expression was associated with smaller tumors; below, weight and volume of the tumors excised at 60 days. Both measures were lower in the xenografts derived from MOLT-4 derived cells transduced with NUDT16. Probabilities are those associated with Student’s *t*-tests. Results are presented as the mean±s.e.m., *n*=10. (**b**) Nuclear/cytoplasmic fractionation of NUDT16-methylated (CCRF-CEM, Jurkat, MOLT-4 and MOLT-16) and NUDT16-unmethylated (HL-60, K562 and KOPN-8) cell lines show expression and cytosolic accumulation of NUDT16 in the unmethylated cells. Purity of fractions was assayed with the nuclear protein β-Tubulin and the cytosolic Lamin-β. WCL, Whole Cell Lysate. (**c**) Left, validation of the mRNA expression levels by RT-qPCR following the Actinomycin D treatment of the RNA-immunoprecipitation-derived candidates FBXO28 and USP37 in NUDT16 or empty vector-transduced MOLT-4 cells. Restoration of NUDT16 expression destabilizes both mRNAs; middle, western blot assays confirm reduction of FBXO28 and USP37 protein expression upon NUDT16 transduction in MOLT-4 cells; right, western blot shows that the diminished FBXO28 and USP37 levels upon NUDT16 restoration are also associated with a decrease in their target, the C-MYC protein.

## References

[bib1] Franks TM, Lykke-Andersen J. The control of mRNA decapping and P-body formation. Mol Cell 2008; 32: 605–615. 19061636 10.1016/j.molcel.2008.11.001 PMC2630519

[bib2] Arribas-Layton M, Wu D, Lykke-Andersen J, Song H. Structural and functional control of the eukaryotic mRNA decapping machinery. Biochim Biophys Acta 2013; 1829: 580–589. 23287066 10.1016/j.bbagrm.2012.12.006 PMC3660425

[bib3] Jonas S, Izaurralde E. The role of disordered protein regions in the assembly of decapping complexes and RNP granules. Genes Dev 2013; 27: 2628–2641. 24352420 10.1101/gad.227843.113 PMC3877753

[bib4] Wang Z, Jiao X, Carr-Schmid A, Kiledjian M. The hDcp2 protein is a mammalian mRNA decapping enzyme. Proc Natl Acad Sci USA 2002; 99: 12663–12668. 12218187 10.1073/pnas.192445599 PMC130517

[bib5] Bessman MJ, Frick DN, O'Handley SF. The MutT proteins or 'Nudix' hydrolases, a family of versatile, widely distributed, 'housecleaning' enzymes. J Biol Chem 1996; 271: 25059–25062. 8810257 10.1074/jbc.271.41.25059

[bib6] Song MG, Li Y, Kiledjian M. Multiple mRNA decapping enzymes in mammalian cells. Mol Cell 2010; 40: 423–342. 21070968 10.1016/j.molcel.2010.10.010 PMC2982215

[bib7] Li Y, Song M, Kiledjian M. Differential utilization of decapping enzymes in mammalian mRNA decay pathways. RNA 2011; 17: 419–428. 21224379 10.1261/rna.2439811 PMC3039142

[bib8] Hopkins KC, Tartell MA, Herrmann C, Hackett BA, Taschuk F, Panda D et al. Virus-induced translational arrest through 4EBP1/2-dependent decay of 5'-TOP mRNAs restricts viral infection. Proc Natl Acad Sci USA 2015; 112: E2920–E2929. 26038567 10.1073/pnas.1418805112 PMC4460451

[bib9] Esteller M. Epigenetics in cancer. N Engl J Med 2008; 358: 1148–1159. 18337604 10.1056/NEJMra072067

[bib10] Iorio F, Knijnenburg TA, Vis DJ, Bignell GR, Menden MP, Schubert M et al. A landscape of pharmacogenomic interactions in cancer. Cell 2016; 166: 740–754. 27397505 10.1016/j.cell.2016.06.017 PMC4967469

[bib11] Fustin JM, Doi M, Yamaguchi Y, Hida H, Nishimura S, Yoshida M et al. RNA-methylation-dependent RNA processing controls the speed of the circadian clock. Cell 2013; 155: 793–806. 24209618 10.1016/j.cell.2013.10.026

[bib12] Cepeda D, Ng HF, Sharifi HR, Mahmoudi S, Cerrato VS, Fredlund E et al. CDK-mediated activation of the SCF(FBXO) (28) ubiquitin ligase promotes MYC-driven transcription and tumourigenesis and predicts poor survival in breast cancer. EMBO Mol Med 2013; 5: 999–1018. 10.1002/emmm.201202341 PMC3721474 23776131

[bib13] Pan J, Deng Q, Jiang C, Wang X, Niu T, Li H et al. USP37 directly deubiquitinates and stabilizes c-Myc in lung cancer. Oncogene 2015; 34: 3957–3967. 25284584 10.1038/onc.2014.327

[bib14] Weng AP, Millholland JM, Yashiro-Ohtani Y, Arcangeli ML, Lau A, Wai C et al. c-Myc is an important direct target of Notch1 in T-cell acute lymphoblastic leukemia/lymphoma. Genes Dev 2006; 20: 2096–2109. 16847353 10.1101/gad.1450406 PMC1536060

[bib15] Palomero T, Lim WK, Odom DT, Sulis ML, Real PJ, Margolin A et al. NOTCH1 directly regulates c-MYC and activates a feed-forward-loop transcriptional network promoting leukemic cell growth. Proc Natl Acad Sci USA 2006; 103: 18261–18266. 17114293 10.1073/pnas.0606108103 PMC1838740

