# Supplementary material for: Epigenetic loss of the RNA decapping enzyme NUDT16 mediates C-MYC activation in T-cell acute lymphoblastic leukemia
Source: Leukemia. 2017 Apr 11;31(7):1622–5. doi: 10.1038/leu.2017.99 (PMC5501321; doi:10.1038/leu.2017.99)
Supplement: Supplementary Information [file leu201799x1.docx]

**Supplementary Methods**

**Cell lines and leukemia samples**

All cell lines used in this study were obtained from the American Type Culture Collection (Manassas, VA) or the Leibniz Institute DSMZ-German Collection of Microorganisms and Cell Cultures (Leibniz, Germany). The DNA methylation inhibitor used was 5-aza-2'-deoxycytidine. MOLT-4 and MOLT-16 cells (mock-transduced or NUDT16-transduced) were treated with DMSO or 10 μg/ml Actinomycin D for 2 h. Total RNA was extracted with Trizol® Reagent (Thermo Fisher Scientific), reverse-transcribed and amplified by qPCR. For protein analysis, total extracts were collected 7 h after Actinomycin D treatment was finished and probed by western blot. Primary samples from T-ALL patients were obtained from the Department of Hematology at the Hospital de la Santa Creu i Sant Pau in Barcelona (Catalonia, Spain) and the Josep Carreras Leukaemia Research Institute in Badalona (Catalonia, Spain), with full informed consent from the patients and the approval of the corresponding Institutional Review Boards.

**DNA Methylation microarray**

DNA methylation microarray data of 1,001 cancer cell lines are publicly available from our recent analyses.^1^ Briefly, cell line DNA samples were assessed for integrity, quantity and purity by electrophoresis in a 1.3% agarose gel, Picogreen quantification, and Nanodrop measurements. All samples were randomly distributed into 96 well plates. Bisulfite conversion of 500 ng of genomic DNA was performed using EZ DNA methylation kit (Zymo Research. Irvine) following manufacturer’s instructions. 200 ng of bisulfite converted DNA were used for hybridization on the HumanMethylation450 BeadChip (Illumina, Inc. San Diego) following manufacturer’s indications. Fluorescent signal from microarrays were measured with a HiScan scanner (Illumina, Inc. San Diego) using iScan Control Software (V 3.3.29). Raw methylation profiles are available on Gene Expression Omnibus (GEO) (accession number: GSE68379). Additionally, CpG methylation beta values from normal human tissues in healthy individuals were retrieved from the COAD and LUAD TCGA projects that are also publicly available. Z-score expression differences based in the DNA methylation status in T-ALL cell lines^1^ and primary T-ALL samples^2^ were tested by applying Mann-Whitney U statistical tests.

**NUDT16 overexpression**

Overexpression of NUDT16 was achieved by lentivirus-mediated expression of ORF-containing plasmids. The NUDT16 IMAGE clone was inserted via PCR-directed subcloning into the pLVX-IRES-ZsGreen (Clontech) by adding EcoRI and BamHI sites to the 5’ and 3’ ends, respectively. Briefly, HEK293T cells were transfected with pLVX-shRNA2-constructs plus packaging plasmids with jetPRIME® (Polyplus transfection) according to the manufacturer’s recommendations. At 48 h post-transfection, the supernatant containing viral particles was used to infect the MOLT-4 and MOLT-16 cell lines. RNA was collected and analyzed after at least eight cell passages post-infection. Positive clones were FACS-sorted.

**Methylation-specific PCR (MSP) and bisulfite genomic sequencing**

DNA was pre-treated with bisulfite reagent using the EZ DNA Methylation Kit (Zymo). Primers for MSP or bisulfite genomic sequencing were designed by Methyl Primer Express v1.0 (Applied Biosystems, Carlsbad, CA, USA). For bisulfite sequencing amplicons were cloned into pGEM-T-easy (Promega) and grown to colonies. Plasmids were isolated and used for sequencing with T7 primers. Results were analyzed with BioEdit software and methylated cytosines were mapped using BSMap software.

**Analyses of NOTCH1 and CNOT3 mutational status**

Sanger sequencing was performed to screen for NOTCH1 and CNOT3 mutations in primary T-ALL samples using described primer sequences.^3,4^ Briefly, all the amplicons were purified and sequenced using the ABI Prism dGTP BigDye Terminator Ready Reaction Kit (Perkin Elmer, Foster City,CA) following manufacture's instruction and an ABI Prism 3100 Genetic Analyser (Perkin Elmer, Foster City, CA). The results were analyzed using 3100 data collection software and CodonCode Aligner. Sequencing was performed in both strands. In cases with suspected mutations PCR amplification was repeated and samples re-sequenced to rule out PCR artefacts.

**Western blot and antibodies**

Cells were lysed in RIPA buffer (150 mM NaCl, 1% Nonidet P-40, 0.5% deoxycholate, 0.1% SDS, 50 mM Tris pH 8.0) for 20 min at 4 ºC. Lysates were spun down for 15 min at 14,000 rpm at 4 ºC. Supernatant was added to 2x Laemmli buffer. Samples were boiled for 5 min and loaded onto SDS-PAGE.

**Quantitative real-time PCR (qRT-PCR)**

Total RNA was isolated from all cancer cell lines by Trizol extraction according to the manufacturer's instructions (Invitrogen). For RNA expression analysis, 1 μg of treated RNA was retrotranscribed using ThermoScript™ RT–PCR System (Invitrogen). All the real-time PCR reactions were carried out in triplicate on Applied Biosystems 7900HT Fast Real Time PCR System using 20 ng of cDNA in a final volume of 10 μl in a 384-well plate. Data were normalized with respect to B2M expression.

**T cell isolation**

T lymphocytes were isolated from the blood of healthy volunteers, using the Pan T Cell Isolation Kit (Miltenyi Biotech) according to the manufacturer’s instructions. The purity of the isolated samples was assessed by qRT-PCR using CD3 primers.

**Cell fractionation**

5x10^6^ cells were washed in cold PBS and lysed in RBS buffer (10 mM Tris pH 7.4, 10 mM NaCl, 6 mM MgCl_2_) with 0.05% Nonidet-P 40. Lysate was resuspended with caution. Samples were spun at 700 g for 10 s and the supernatant was spun again and the cytoplasmic fraction collected. The pellet was washed in RBS buffer with 0.055% Nonidet-P 40 and lysed in RIPA buffer and spun for 1 min at 20,000 g. The supernatant was spun again for 1 min at 20,000 g and the nuclear fraction was collected. As a control, whole-cell lysate was taken using only RIPA. All buffers were supplemented with Protease Inhibitor Cocktail (Roche).

**MTT proliferation assay**

10,000 cells per well were seeded into 96-wells plates. At various times, MTT reagent was added and incubated for 3 h, after which the cells were lysed for 16 h with MTT lysis buffer (50% N-N dimethylformide, 20% sodium dodecyl formate DS, 2.5% glacial acetic acid, 2.1% 1N HCl, at pH 4.7). Plates were measured at 560 nm using a spectrophotometer.

**Mouse subcutaneous injections**

Athymic nude male mice were subcutaneously injected in each flank with NUDT16-negative cells in the right flank (5x10^6^ of MOLT-4 or MOLT-16 wild type) and NUDT16-positive cells in the left flank (5x10^6^ of MOLT4 or MOLT-16-overexpressing NUDT16). Tumor growth was monitored every 2 days by measuring width (W) and length (L). Tumor volume, V, was then estimated from the formula V=π/(6 × L × W^2^). Mice were killed 60 (MOLT-4) or 30 (MOLT-16) days after injection and the tumor weight and volume were measured. All mice experiments were approved by the IDIBELL Animal Care and Use Committee and performed in accordance with guidelines stated in The International Guiding Principles for Biomedical Research involving Animals, developed by the Council for International Organizations of Medical Sciences (CIOMS).

**RNA immunoprecipitation and expression microarray analysis**

Total RNA from mock-transfected or NUDT16-overexpressing MOLT-4 cells was extracted using Trizol® Reagent (Thermo Fisher Scientific). 20 μg of total RNA was used in each cap-immunoprecipitation reaction as described.^5^ In brief, Dynabeads® Protein G beads (Thermo Fisher Scientific) (50 μl per reaction) were washed three times with 1 ml PBS buffer before incubation with 10 μl of the anti-m_3_G/m^7^G antibody (Synaptic-System) for 3 h at 4 ºC. Mouse IgG was used in parallel control reactions. Beads were then washed three times with ice-cold PBS and twice with IPP buffer (0.1 M PBS, 0.01% Triton X-100, 0.1 mg/ml BSA, 1 mM DTT, 0.4 U/μl RNase inhibitor), resuspended in IPP buffer and the RNA added in a total volume of 250 μl. The mixture was incubated for 3 h, rocking at 4 ºC, after which the supernatant (unbound fraction) was kept to estimate the pull-down efficiency. Beads were washed five times in 1 ml IPP buffer, and the captured RNA was recovered by phenol/chloroform extraction and ethanol precipitation. The RNA eluates from mock and NUDT16 cells were then hybridized onto expression microarrays (Agilent Gene Expression one-color chip human 8x60K) at the Center for Genomic Regulation (CRG, Barcelona). Hybridization, washing, staining and scanning were performed using Agilent system instruments and protocols. The expression data microarray analysis were performed with the Bioconductor limma library v3.28 ^6^ in the R v3.3.0 statistical environment. Extracted intensities were background corrected using the normexp method. Background corrected log2-transformed intensities were normalized using quantile normalization to make data from all arrays comparable. For determining differentially regulated mRNAs between RIP mock-transfected and NUDT16-overexpressing MOLT-4 cells a linear model followed by moderated t-test were applied. The expression microarray data obtained have been deposited in the Gene Expression Omnibus (GEO) repository under Accession Number GSE84973 (Link for the reviewers until acceptance of the manuscript:

<http://www.ncbi.nlm.nih.gov/geo/query/acc.cgi?token=ipcraqmufnwdrgv&acc=GSE84973>.

**MYC immunoprecipitation and ubiquitination analysis**

Whole cell lysates from MOLT-16 control or NUDT16-overexpressing cells were prepared in RIPA buffer using standard protocols. Endogenous MYC protein was immunoprecipitated overnight at 4°C using 1 mg of lysate, 5 μg of mouse monoclonal to C-MYC (Abcam) and 50 μl of Dynabeads protein G (Life Technologies). Proteins were eluted by addition of 40 μl SDS loading buffer and incubated for 10min at 70°C. 20 μl of each sample was run on a 12% SDS PAGE gel, transferred to a nitrocellulose membrane, blocked and probed with either a rabbit anti-MYC antibody (Cell Signaling) in 5% BSA or with a mouse anti-ubiquitin antibody (Millipore) in 5% milk.

**Statistical Evaluation**

Statistical analyses were performed using GraphPad Prism Software 5.04 (San Diego, California, USA) and SPSS version 17.0 (SPSS, Chicago, IL, USA) for Windows XP (Microsoft, Redmond, WA, USA). Experimental data are represented as the mean (±SEM) of a minimum of three biological replicates. For comparisons, Student’s t-tests were performed as appropriate. Level of statistical significance: *, p < 0.05; **, p < 0.01; ***, p < 0.001; ****, p < 0.0001. We used the Kaplan-Meier method for the survival analysis of cancer patients.

**REFERENCES**

1. Iorio F, Knijnenburg TA, Vis DJ, Bignell GR, Menden MP, Schubert M, *et al*. A landscape of pharmacogenomic interactions in cancer. *Cell* 2016; **166**: 740-54.
2. Borssén M, Palmqvist L, Karrman K, Abrahamsson J, Behrendtz M, Heldrup J, *et al*. Promoter DNA methylation pattern identifies prognostic subgroups in childhood T-cell acute lymphoblastic leukemia. *PLoS One* 2013; **8**: e65373.
3. Weng AP, Ferrando AA, Lee W, Morris JP 4th, Silverman LB, Sanchez-Irizarry C, *et al*. Activating mutations of NOTCH1 in human T cell acute lymphoblastic leukemia. *Science* 2004; **306**: 269-71.
4. De Keersmaecker K, Atak ZK, Li N, Vicente C, Patchett S, Girardi T, *et al*. Exome sequencing identifies mutation in CNOT3 and ribosomal genes RPL5 and RPL10 in T-cell acute lymphoblastic leukemia. *Nat Genet* 2013; **45**: 186-90.
5. Fustin JM, Doi M, Yamaguchi Y, Hida H, Nishimura S, Yoshida M, *et al*. RNA-methylation-dependent RNA processing controls the speed of the circadian clock. *Cell* 2013; **155**: 793-806.
6. Ritchie ME, Phipson B, Wu D, Hu Y, Law CW, Shi W, *et al*. limma powers differential expression analyses for RNA-sequencing and microarray studies. *Nucleic Acids Res* 2015; **43**: e47.

**Antibodies used in the study**

| **Name** | **Reference** | **Company** | **Application** | **Dilution** |
| --- | --- | --- | --- | --- |
| Anti-USP37 | HPA045160 | Atlas Antibodies | WB | 1:250 |
| Anti-FBXO28 | ab154068 | Abcam | WB | 1:1000 |
| Anti-c-myc | D84C12 | Cell Signaling | WB | 1:1000 |
| Anti-c-myc | ab56 | Abcam | IP | 1:200 |
| Anti-ubiquitin | MAB1510 | Millipore | WB | 1:500 |
| Anti-NUDT16 | MBS839126 | MyBiosource | WB | 1:1000 |
| Anti-β-Actin-HRP | A3854 | Sigma | WB | 1:20,000 |
| Anti-beta Tubulin-HRP | Ab21058 | Abcam | WB | 1:1000 |
| Anti-lamin B1 | Ab16048 | Abcam | WB | 1:1000 |

**Primer sequences used in the study**

| **Name** | **Sequence** | **Application** |
| --- | --- | --- |
| GT-RT CD3e F1 | CTCTTATCAGTTGGCGTTTGG | qRT-PCR |
| GT-RT CD3e R1 | CATCTTCTGGTTTGCTTCCT | qRT-PCR |
| qPCR-Nudt16_Fw | TACGCCATACTGATGCAGA | qRT-PCR |
| qPCR-Nudt16_Rv | GTCCTCTAGGCTTCTGTCCT | qRT-PCR |
| qPCR-B2M_Fw | ATGAGTATGCCTGCCGTGTGA | qRT-PCR |
| qPCR-B2M_Rv | GGCATCTTCAAACCTCCATG | qRT-PCR |
| qPCR_ GSK3B_ Fw | CTCCTCATGCTCGGATTCA | qRT-PCR |
| qPCR_ GSK3B_ Rv | TGCAGAAGCAGCATTATTGG | qRT-PCR |
| qPCR_ BCL11A_ Fw | GGGGATTAGAGCTCCATGTG | qRT-PCR |
| qPCR_BCL11A_ Rv | TCTAGAGGAATTTGCCCCAA | qRT-PCR |
| qPCR_ MAP3K2_ Fw | ACAGAGCTACCCAGATAATCA | qRT-PCR |
| qPCR_MAP3K2_ Rv | CTTCTTGGATAAGTTCCTCCTT | qRT-PCR |
| qPCR_ FBXO28_ Fw | CCAGGAAAGGTGATTGATGAG | qRT-PCR |
| qPCR_FBXO28_ Rv | AGAGCTGCATTGTTGTTAGG | qRT-PCR |
| qPCR_ USP37_ Fw | TCTGCCTCATTCGTACCG | qRT-PCR |
| qPCR_USP37_ Rv | ACGGGTAGTCTTCCCCACTTC | qRT-PCR |
| MSP_M-NUDT16_Fw | TGTTTTTCGTATCGGTCGC | Methylation specific PCR |
| MSP_M-NUDT16_Rv | ACGAATCTCACCTCGAACC | Methylation specific PCR |
| MSP_U-NUDT16_Fw | AGTTGTTTTTTGTATTGGTTGT | Methylation specific PCR |
| MSP_U-NUDT16_Rv | AAAACAAATCTCACCTCAAACC | Methylation specific PCR |
| NUDT16_Bs1_Fw | GTTTTTTGATTTGTTTTTTTGG | Bisulfite seq |
| NUDT16_Bs1_Rv | CTACAAAACCCAATCCCTACA | Bisulfite seq |
| NUDT16_Bs2_Fw | TGTAGGGATTGGGTTTTGTAGT | Bisulfite seq |
| NUDT16_Bs2_Rv | CCCCCTTCTCACCAATATAAC | Bisulfite seq |
| Notch1HDnterm_FW1 | AGCCCCCTGTACGACCAGTA | Sanger seq |
| Notch1HDnterm_Rv1 | CTTGCGCAGCTCCTCCTC | Sanger seq |
| Notch1HDnterm_FW2 | GTGCTGCACACCAACGTG | Sanger seq |
| Notch1HDnterm_Rv2 | GAGGGCCCAGGAGAGTTG | Sanger seq |
| Notch1HDcterm_FW | CATGGGCCTCAGTGTCCT | Sanger seq |
| Notch1HDcterm_Rv | GCACAAACAGCCAGCGTGTC | Sanger seq |
| Notch1PEST_FW1 | GCAGCATGGCATGGTAGG | Sanger seq |
| Notch1PEST_Rv1 | AACATGTGTTTTAAAAAGGCTCCTC | Sanger seq |
| Notch1PEST_FW2 | AAACATCCAGCAGCAGCAAA | Sanger seq |
| Notch1PEST_Rv2 | CACAGGCGAGGAGTAGCTGTG | Sanger seq |
| Notch1TAD_FW1 | AGACTGGCCCACCTCGTCTCT | Sanger seq |
| Notch1TAD_RV1 | GCTCTCCACTCAGGAAGCTC | Sanger seq |
| CNOT3_ex2-3F | tttaccagccagggaatacg | Sanger seq |
| CNOT3_ex2-3R | ctggtcaacacccagaggtc | Sanger seq |
| CNOT3_Ex4F | ggtcctcgagtccctagcat | Sanger seq |
| CNOT3_Ex4R | gcagtccactctcccagttc | Sanger seq |
| CNOT3_Ex5F | gaactgggagagtggactgc | Sanger seq |
| CNOT3_Ex5R | gtgaccttcccacctctctg | Sanger seq |
| CNOT3_Ex6-7F | gtctgctggcccttagtcag | Sanger seq |
| CNOT3_Ex6-7R | ccaactcccaggagcataaa | Sanger seq |
| CNOT3_Ex8-9F | actgaggacaggttctgtgg | Sanger seq |
| CNOT3_Ex8-9R | cagatttgtctcctcgagtcc | Sanger seq |
| CNOT3_Ex10F | cattcagagattggcggttc | Sanger seq |
| CNOT3_Ex10R | ctcaagatgcctttgggaag | Sanger seq |
| CNOT3_Ex14-15F | cctgtgtcaggctgcactt | Sanger seq |
| CNOT3_Ex14-15R | actgcctcctccgtaagact | Sanger seq |
| CNOT3_Ex16F | tgtctgagcacccttttgat | Sanger seq |
| CNOT3_Ex16R | cccaggagtgggaataggg | Sanger seq |
| CNOT3_Ex18F | tgacacatccacagccctaa | Sanger seq |
| CNOT3_Ex18R | cctccctccagtcttccag | Sanger seq |
