# Supplementary material for: Epigenetic loss of the RNA decapping enzyme NUDT16 mediates C-MYC activation in T-cell acute lymphoblastic leukemia
Source: Leukemia. 2017 Apr 11;31(7):1622–5. doi: 10.1038/leu.2017.99 (PMC5501321; doi:10.1038/leu.2017.99)
Supplement: Supplementary Figure S1 [file leu201799x2.ppt]

## Slide 1
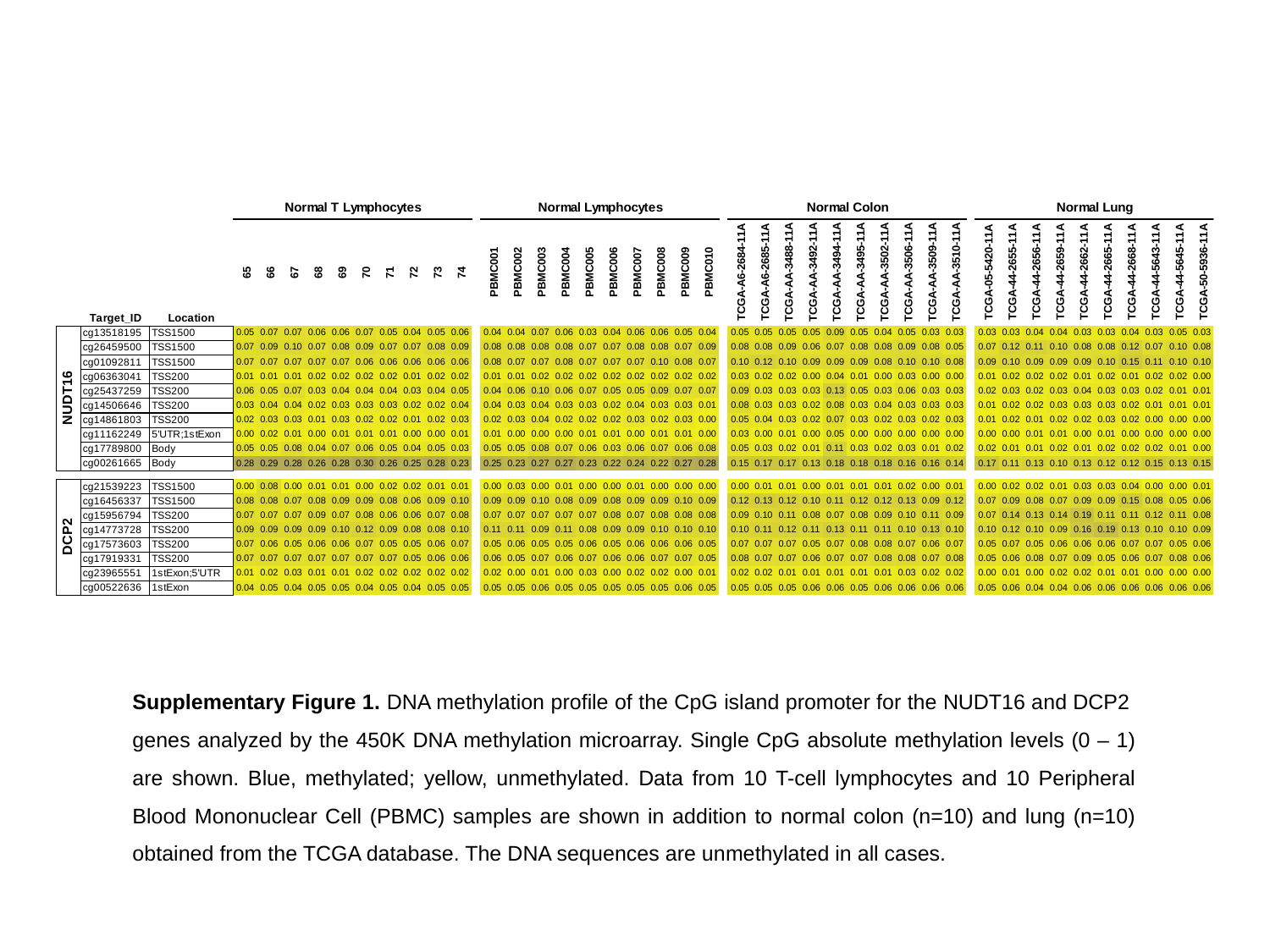

Supplementary Figure 1. DNA methylation profile of the CpG island promoter for the NUDT16 and DCP2 genes analyzed by the 450K DNA methylation microarray. Single CpG absolute methylation levels (0 – 1) are shown. Blue, methylated; yellow, unmethylated. Data from 10 T-cell lymphocytes and 10 Peripheral Blood Mononuclear Cell (PBMC) samples are shown in addition to normal colon (n=10) and lung (n=10) obtained from the TCGA database. The DNA sequences are unmethylated in all cases.
