# Supplementary material for: Epigenetic loss of the RNA decapping enzyme NUDT16 mediates C-MYC activation in T-cell acute lymphoblastic leukemia
Source: Leukemia. 2017 Apr 11;31(7):1622–5. doi: 10.1038/leu.2017.99 (PMC5501321; doi:10.1038/leu.2017.99)
Supplement: Supplementary Figure S2 [file leu201799x3.ppt]

## Slide 1
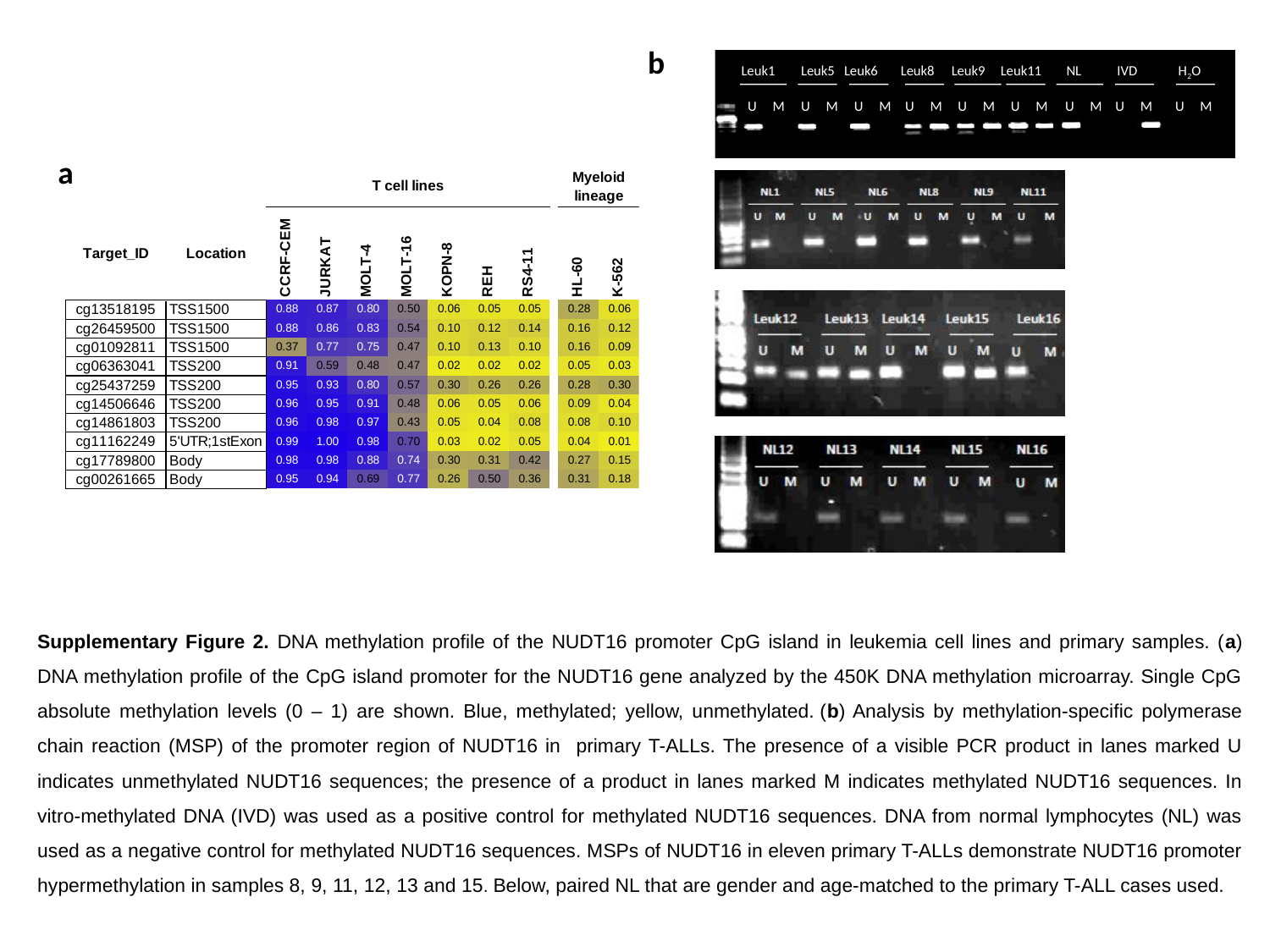

b
Leuk1
Leuk5
 Leuk6
 Leuk8
Leuk9
Leuk11
 NL
IVD
H2O
U M
 U M
 U M
U M
U M
 U M
U M
U M
U M
a
Supplementary Figure 2. DNA methylation profile of the NUDT16 promoter CpG island in leukemia cell lines and primary samples. (a) DNA methylation profile of the CpG island promoter for the NUDT16 gene analyzed by the 450K DNA methylation microarray. Single CpG absolute methylation levels (0 – 1) are shown. Blue, methylated; yellow, unmethylated. (b) Analysis by methylation-specific polymerase chain reaction (MSP) of the promoter region of NUDT16 in primary T-ALLs. The presence of a visible PCR product in lanes marked U indicates unmethylated NUDT16 sequences; the presence of a product in lanes marked M indicates methylated NUDT16 sequences. In vitro-methylated DNA (IVD) was used as a positive control for methylated NUDT16 sequences. DNA from normal lymphocytes (NL) was used as a negative control for methylated NUDT16 sequences. MSPs of NUDT16 in eleven primary T-ALLs demonstrate NUDT16 promoter hypermethylation in samples 8, 9, 11, 12, 13 and 15. Below, paired NL that are gender and age-matched to the primary T-ALL cases used.
