# Supplementary material for: Epigenetic loss of the RNA decapping enzyme NUDT16 mediates C-MYC activation in T-cell acute lymphoblastic leukemia
Source: Leukemia. 2017 Apr 11;31(7):1622–5. doi: 10.1038/leu.2017.99 (PMC5501321; doi:10.1038/leu.2017.99)
Supplement: Supplementary Figure S3 [file leu201799x4.ppt]

## Slide 1
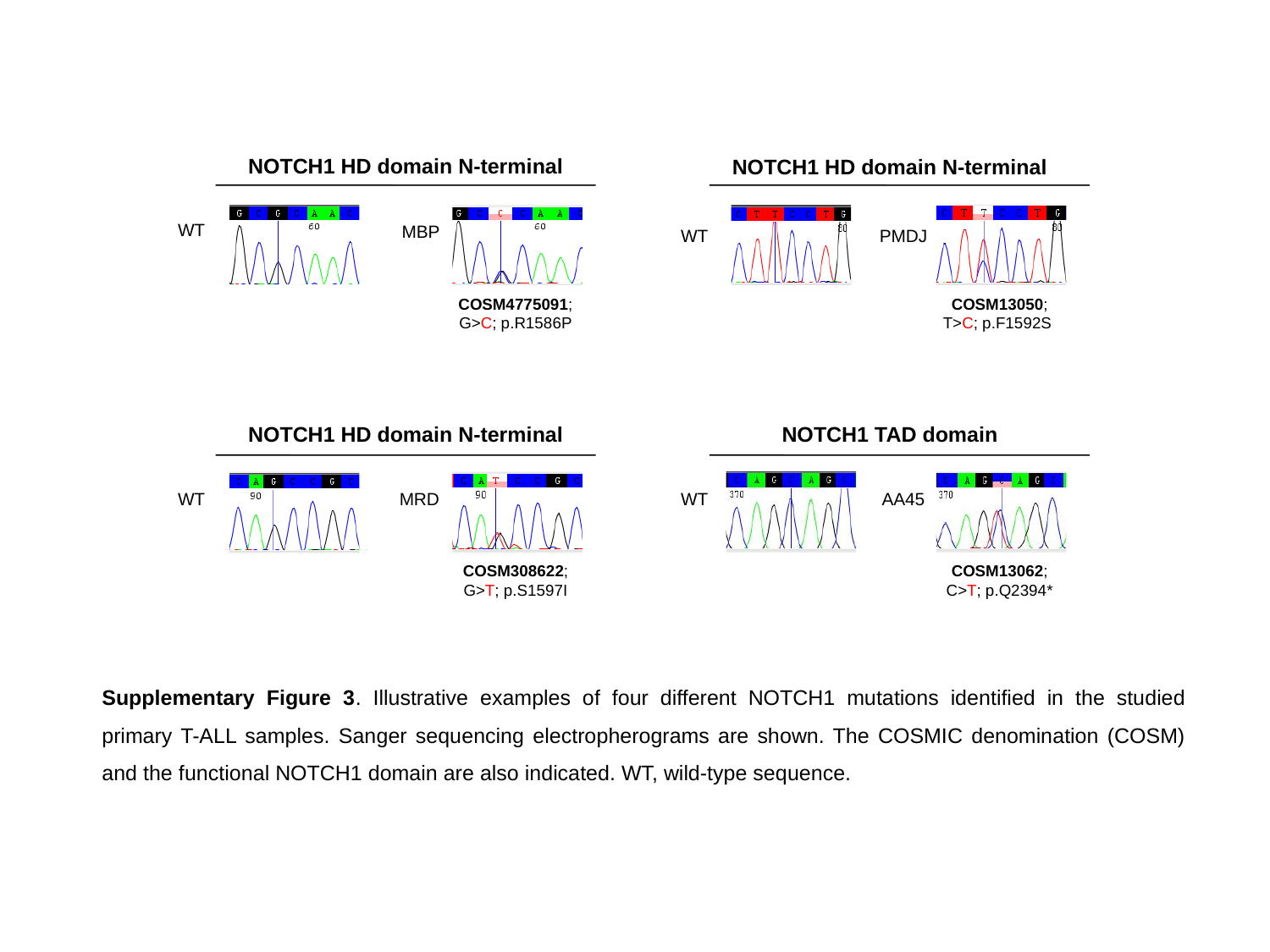

NOTCH1 HD domain N-terminal
NOTCH1 HD domain N-terminal
WT
MBP
WT
PMDJ
COSM4775091;
G>C; p.R1586P
COSM13050;
T>C; p.F1592S
NOTCH1 HD domain N-terminal
NOTCH1 TAD domain
WT
MRD
WT
AA45
COSM308622;
G>T; p.S1597I
COSM13062;
C>T; p.Q2394*
Supplementary Figure 3. Illustrative examples of four different NOTCH1 mutations identified in the studied primary T-ALL samples. Sanger sequencing electropherograms are shown. The COSMIC denomination (COSM) and the functional NOTCH1 domain are also indicated. WT, wild-type sequence.
