# Supplementary material for: Epigenetic loss of the RNA decapping enzyme NUDT16 mediates C-MYC activation in T-cell acute lymphoblastic leukemia
Source: Leukemia. 2017 Apr 11;31(7):1622–5. doi: 10.1038/leu.2017.99 (PMC5501321; doi:10.1038/leu.2017.99)
Supplement: Supplementary Figure S4 [file leu201799x5.ppt]

## Slide 1
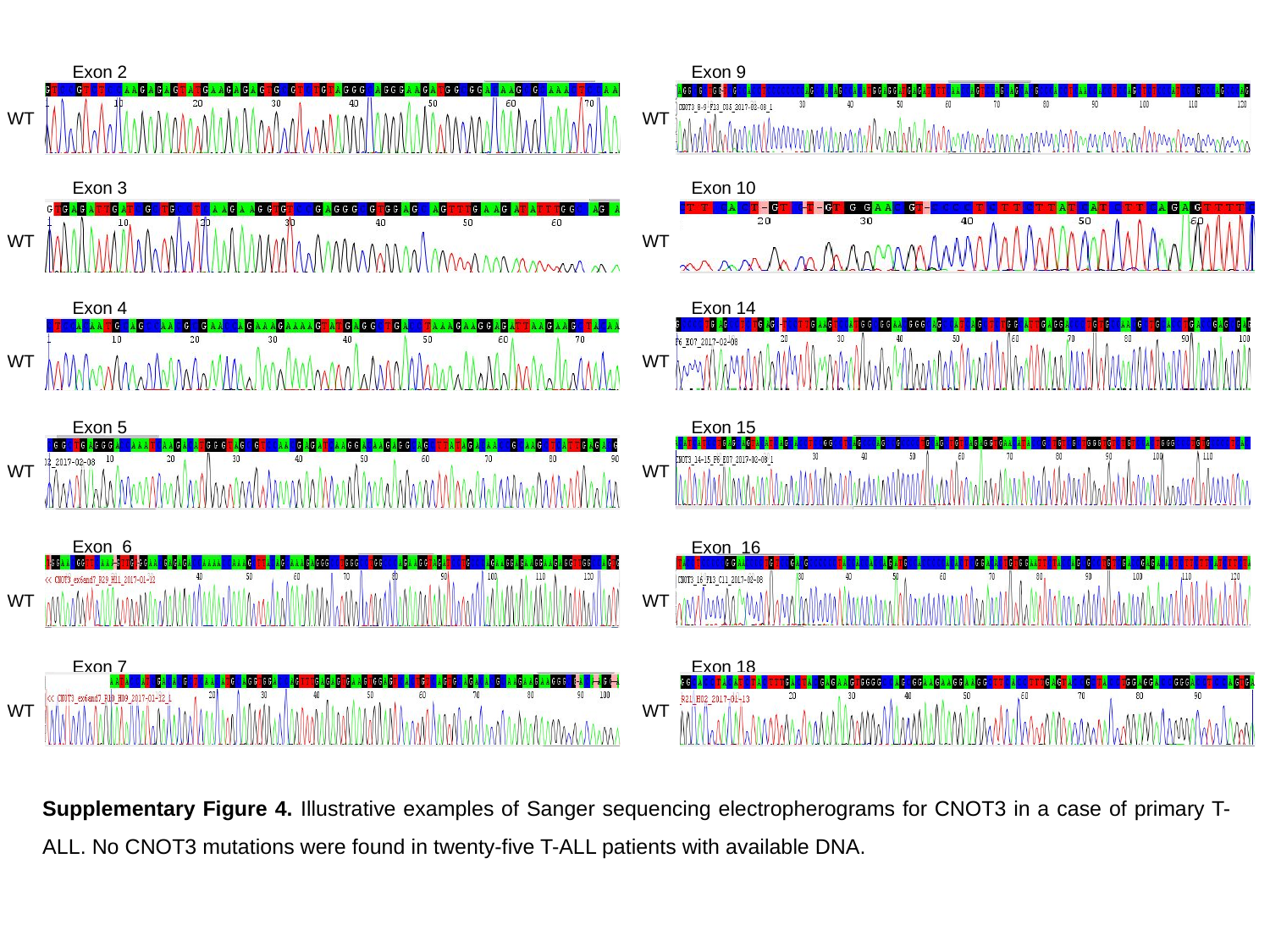

Exon 2
Exon 9
WT
WT
Exon 3
Exon 10
WT
WT
Exon 4
Exon 14
WT
WT
Exon 5
Exon 15
WT
WT
Exon 6
Exon 16
WT
WT
Exon 7
Exon 18
WT
WT
Supplementary Figure 4. Illustrative examples of Sanger sequencing electropherograms for CNOT3 in a case of primary T-ALL. No CNOT3 mutations were found in twenty-five T-ALL patients with available DNA.
