# Supplementary material for: Epigenetic loss of the RNA decapping enzyme NUDT16 mediates C-MYC activation in T-cell acute lymphoblastic leukemia
Source: Leukemia. 2017 Apr 11;31(7):1622–5. doi: 10.1038/leu.2017.99 (PMC5501321; doi:10.1038/leu.2017.99)
Supplement: Supplementary Figure S5 [file leu201799x6.ppt]

## Slide 1
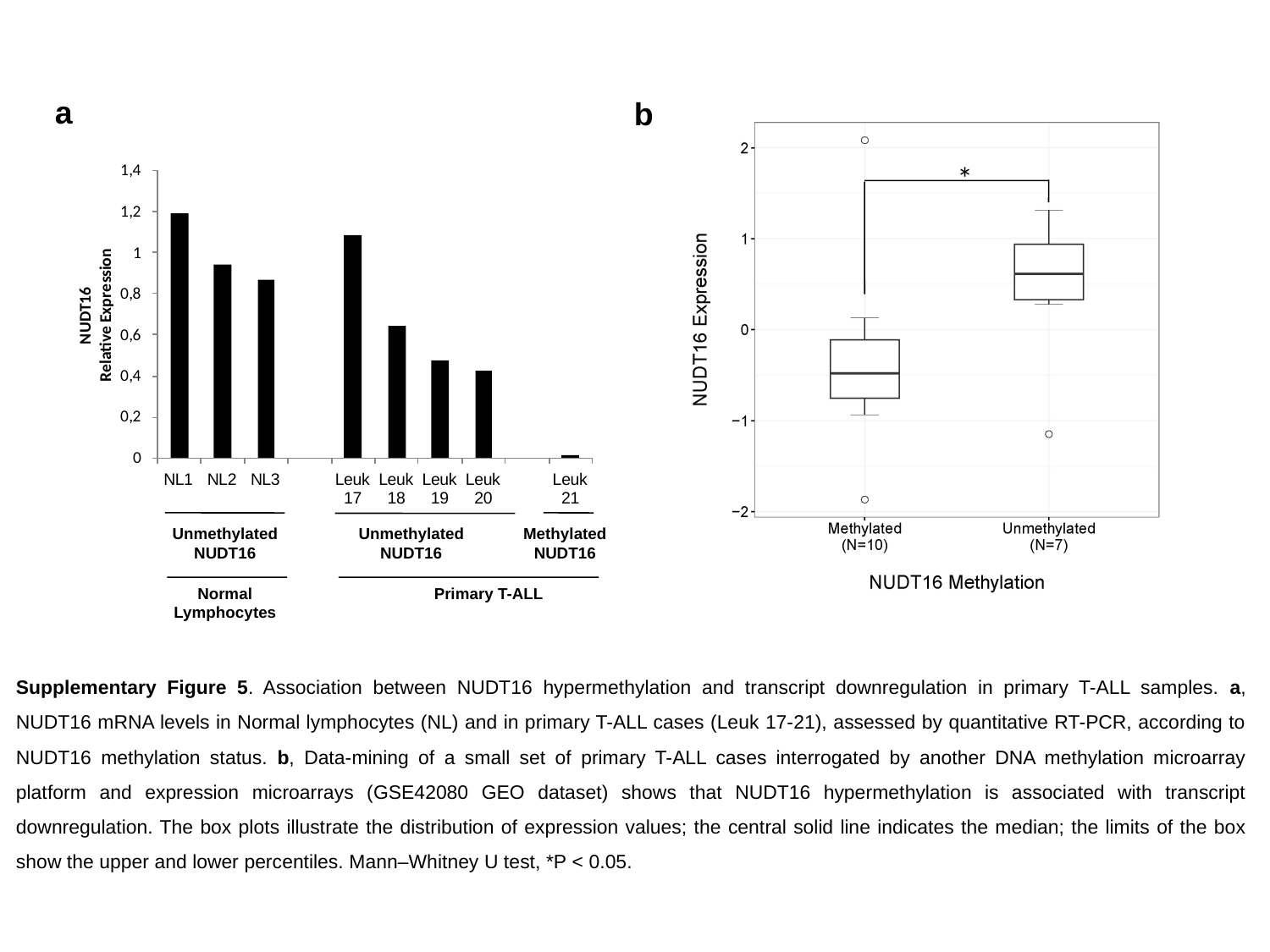

a
b
*
Unmethylated NUDT16
Unmethylated NUDT16
Methylated NUDT16
Normal Lymphocytes
Primary T-ALL
Supplementary Figure 5. Association between NUDT16 hypermethylation and transcript downregulation in primary T-ALL samples. a, NUDT16 mRNA levels in Normal lymphocytes (NL) and in primary T-ALL cases (Leuk 17-21), assessed by quantitative RT-PCR, according to NUDT16 methylation status. b, Data-mining of a small set of primary T-ALL cases interrogated by another DNA methylation microarray platform and expression microarrays (GSE42080 GEO dataset) shows that NUDT16 hypermethylation is associated with transcript downregulation. The box plots illustrate the distribution of expression values; the central solid line indicates the median; the limits of the box show the upper and lower percentiles. Mann–Whitney U test, *P < 0.05.
