# Supplementary material for: Epigenetic loss of the RNA decapping enzyme NUDT16 mediates C-MYC activation in T-cell acute lymphoblastic leukemia
Source: Leukemia. 2017 Apr 11;31(7):1622–5. doi: 10.1038/leu.2017.99 (PMC5501321; doi:10.1038/leu.2017.99)
Supplement: Supplementary Figure S6 [file leu201799x7.ppt]

## Slide 1
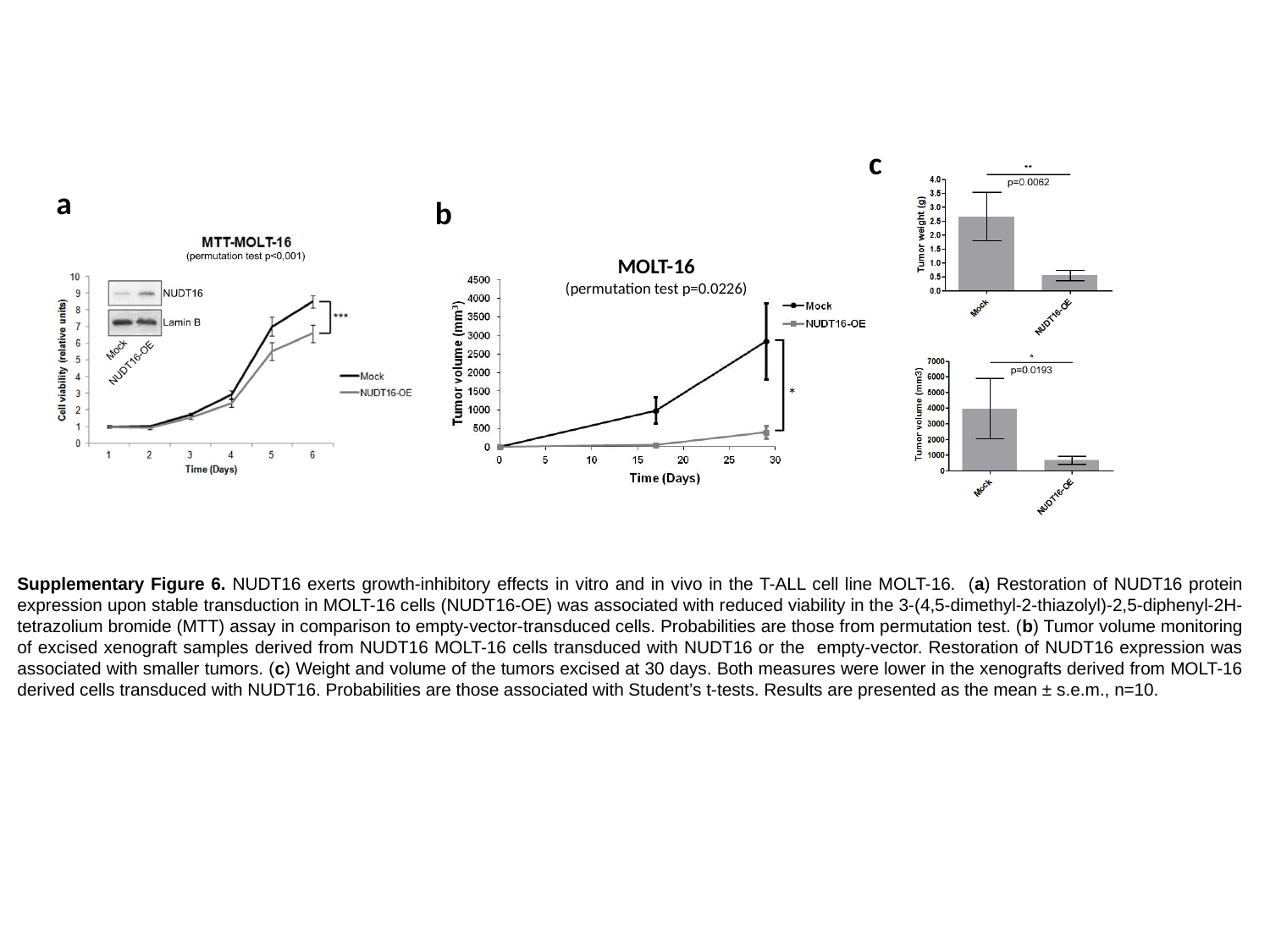

c
a
b
MOLT-16
(permutation test p=0.0226)
*
Supplementary Figure 6. NUDT16 exerts growth-inhibitory effects in vitro and in vivo in the T-ALL cell line MOLT-16. (a) Restoration of NUDT16 protein expression upon stable transduction in MOLT-16 cells (NUDT16-OE) was associated with reduced viability in the 3-(4,5-dimethyl-2-thiazolyl)-2,5-diphenyl-2H-tetrazolium bromide (MTT) assay in comparison to empty-vector-transduced cells. Probabilities are those from permutation test. (b) Tumor volume monitoring of excised xenograft samples derived from NUDT16 MOLT-16 cells transduced with NUDT16 or the empty-vector. Restoration of NUDT16 expression was associated with smaller tumors. (c) Weight and volume of the tumors excised at 30 days. Both measures were lower in the xenografts derived from MOLT-16 derived cells transduced with NUDT16. Probabilities are those associated with Student’s t-tests. Results are presented as the mean ± s.e.m., n=10.
