# Supplementary material for: Epigenetic loss of the RNA decapping enzyme NUDT16 mediates C-MYC activation in T-cell acute lymphoblastic leukemia
Source: Leukemia. 2017 Apr 11;31(7):1622–5. doi: 10.1038/leu.2017.99 (PMC5501321; doi:10.1038/leu.2017.99)
Supplement: Supplementary Figure S7 [file leu201799x8.ppt]

## Slide 1
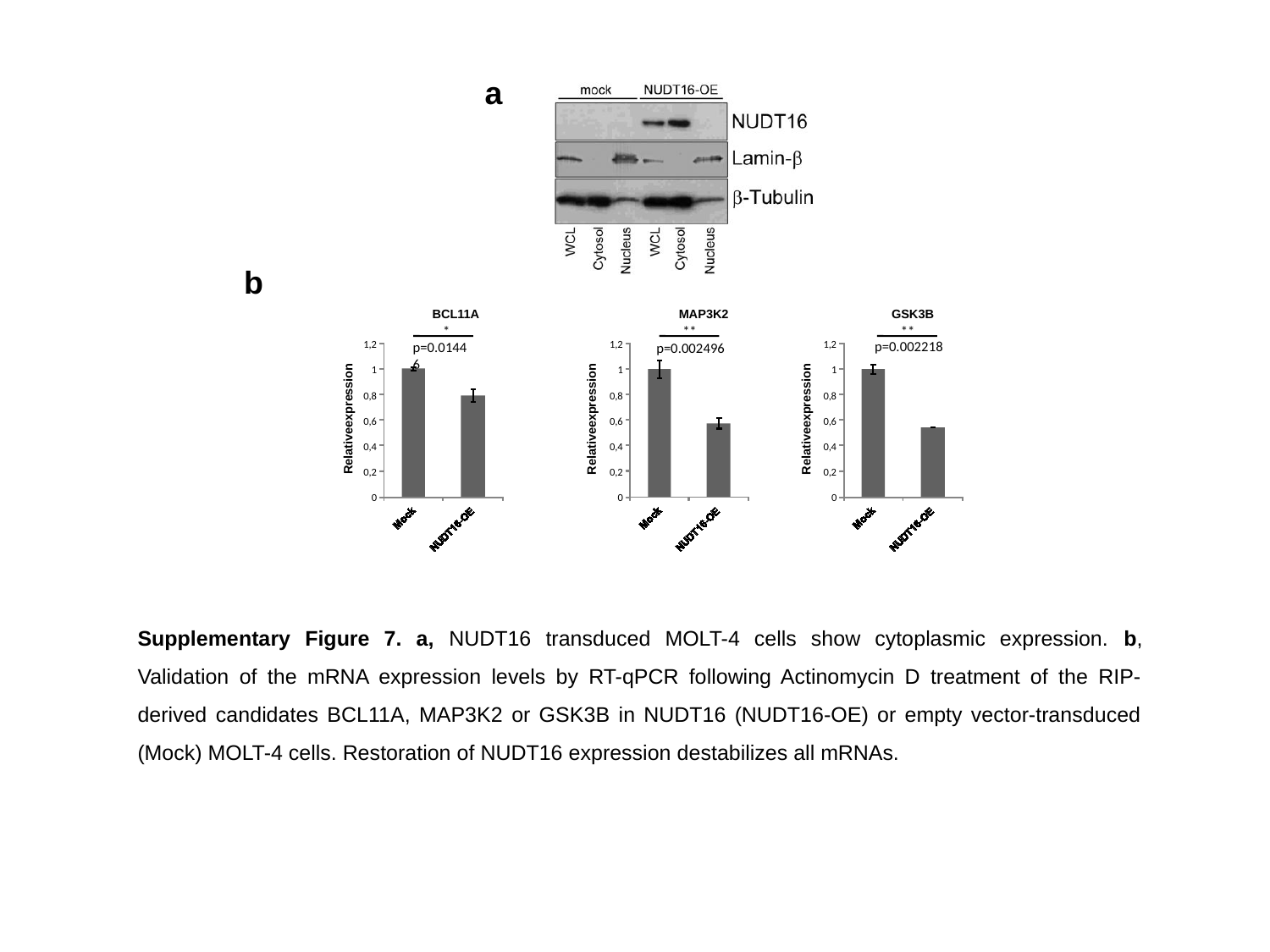

a
b
BCL11A
p=0.01446
1,2
1
0,8
0,6
0,4
0,2
0
*
Relativeexpression
MAP3K2
1,2
1
0,8
0,6
0,4
0,2
0
**
p=0.002496
Relativeexpression
GSK3B
1,2
1
0,8
0,6
0,4
0,2
0
**
p=0.002218
Relativeexpression
Supplementary Figure 7. a, NUDT16 transduced MOLT-4 cells show cytoplasmic expression. b, Validation of the mRNA expression levels by RT-qPCR following Actinomycin D treatment of the RIP-derived candidates BCL11A, MAP3K2 or GSK3B in NUDT16 (NUDT16-OE) or empty vector-transduced (Mock) MOLT-4 cells. Restoration of NUDT16 expression destabilizes all mRNAs.
