# Supplementary material for: Epigenetic loss of the RNA decapping enzyme NUDT16 mediates C-MYC activation in T-cell acute lymphoblastic leukemia
Source: Leukemia. 2017 Apr 11;31(7):1622–5. doi: 10.1038/leu.2017.99 (PMC5501321; doi:10.1038/leu.2017.99)
Supplement: Supplementary Figure S8 [file leu201799x9.ppt]

## Slide 1
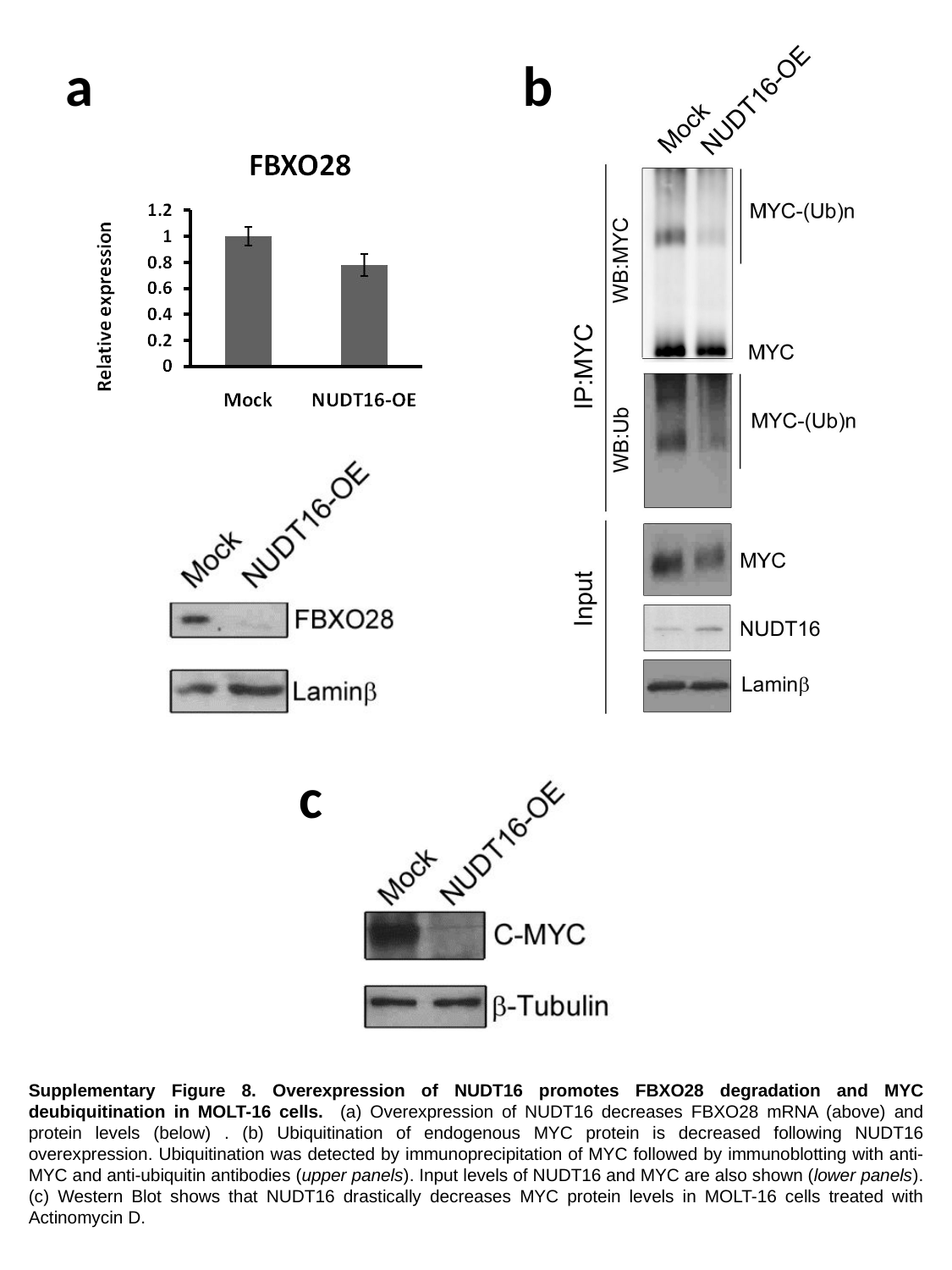

a
b
c
Supplementary Figure 8. Overexpression of NUDT16 promotes FBXO28 degradation and MYC deubiquitination in MOLT-16 cells. (a) Overexpression of NUDT16 decreases FBXO28 mRNA (above) and protein levels (below) . (b) Ubiquitination of endogenous MYC protein is decreased following NUDT16 overexpression. Ubiquitination was detected by immunoprecipitation of MYC followed by immunoblotting with anti-MYC and anti-ubiquitin antibodies (upper panels). Input levels of NUDT16 and MYC are also shown (lower panels). (c) Western Blot shows that NUDT16 drastically decreases MYC protein levels in MOLT-16 cells treated with Actinomycin D.
