# Supplementary material for: Epigenetic loss of the RNA decapping enzyme NUDT16 mediates C-MYC activation in T-cell acute lymphoblastic leukemia
Source: Leukemia. 2017 Apr 11;31(7):1622–5. doi: 10.1038/leu.2017.99 (PMC5501321; doi:10.1038/leu.2017.99)
Supplement: Supplementary Table S1 [file leu201799x10.pdf]

Supplementary Table 1. B-values for the methylation status of the CpG dinucleotides located in the DCP2 and NUDT16 CpG island associated with the transcription start site of DCP2 and NUDT16 in the Sanger panel of cell lines

		TargetID	cg21539223	cg16456337	cg15956794	cg14773728	cg17573603	cg17919331	cg23965551	cg00522636	cg13518195	cg26459500	cg01092811	cg06363041	cg25437259	cg14506646	cg14861803	cg11162249	cg17789800	cg00261665
		UCSC_REFGENE_NAME	DCP2								NUDT16									
		UCSC_REFGENE_GROUP	TSS1500	TSS1500	TSS200	TSS200	TSS200	TSS200	1stExon;5'UTR	1stExon	TSS1500	TSS1500	TSS1500	TSS200	TSS200	TSS200	TSS200	5'UTR;1stExon	Body	Body
Cell Line	Tissue	Cell Line Type																		
SW13	Adrenal Gland	Adenocortical Carcinoma	0.01	0.08	0.07	0.10	0.07	0.08	0.04	0.05	0.27	0.24	0.13	0.03	0.33	0.06	0.04	0.01	0.36	0.53
SK-N-AS	Autonomic Ganglia	Neuroblastoma	0.01	0.07	0.10	0.10	0.09	0.08	0.03	0.09	0.07	0.10	0.14	0.04	0.28	0.04	0.04	0.02	0.25	0.27
SK-N-BE(2)	Autonomic Ganglia	Neuroblastoma	0.01	0.07	0.08	0.09	0.08	0.07	0.02	0.06	0.06	0.10	0.10	0.02	0.29	0.03	0.04	0.00	0.26	0.23
SK-N-FI	Autonomic Ganglia	Neuroblastoma	0.01	0.10	0.07	0.11	0.07	0.08	0.02	0.06	0.03	0.07	0.07	0.02	0.32	0.04	0.06	0.01	0.30	0.35
IMR-32	Autonomic Ganglia	Neuroblastoma	0.02	0.09	0.08	0.10	0.08	0.08	0.02	0.07	0.21	0.12	0.15	0.07	0.38	0.22	0.12	0.21	0.31	0.28
KP-N-SI9S	Autonomic Ganglia	Neuroblastoma	0.01	0.06	0.08	0.10	0.09	0.05	0.02	0.07	0.04	0.12	0.10	0.02	0.32	0.03	0.05	0.01	0.27	0.21
GOTO	Autonomic Ganglia	Neuroblastoma	0.03	0.12	0.09	0.12	0.06	0.07	0.03	0.06	0.10	0.10	0.10	0.03	0.29	0.05	0.04	0.02	0.29	0.24
SK-N-SH	Autonomic Ganglia	Neuroblastoma	0.03	0.09	0.07	0.09	0.06	0.07	0.01	0.05	0.04	0.10	0.08	0.02	0.29	0.05	0.03	0.01	0.28	0.28
BE2-M17	Autonomic Ganglia	Neuroblastoma	0.00	0.11	0.06	0.08	0.06	0.08	0.01	0.07	0.06	0.08	0.09	0.03	0.29	0.04	0.03	0.00	0.28	0.21
MC-IXC	Autonomic Ganglia	Neuroblastoma	0.08	0.12	0.08	0.11	0.07	0.07	0.04	0.07	0.85	0.81	0.53	0.71	0.88	0.94	0.89	0.99	0.87	0.92
NB69	Autonomic Ganglia	Neuroblastoma	0.04	0.12	0.08	0.10	0.07	0.08	0.02	0.06	0.05	0.09	0.08	0.03	0.29	0.05	0.04	0.01	0.32	0.24
KELLY	Autonomic Ganglia	Neuroblastoma	0.02	0.11	0.08	0.10	0.07	0.08	0.03	0.07	0.06	0.09	0.08	0.03	0.29	0.04	0.04	0.02	0.31	0.24
SK-N-DZ	Autonomic Ganglia	Neuroblastoma	0.00	0.11	0.08	0.10	0.07	0.08	0.02	0.07	0.05	0.09	0.10	0.03	0.29	0.04	0.05	0.01	0.29	0.23
MHH-NB-11	Autonomic Ganglia	Neuroblastoma	0.07	0.10	0.07	0.11	0.07	0.07	0.05	0.06	0.05	0.10	0.08	0.03	0.31	0.05	0.03	0.01	0.31	0.24
CHP-212	Autonomic Ganglia	Neuroblastoma	0.14	0.10	0.08	0.10	0.07	0.08	0.03	0.06	0.07	0.08	0.09	0.03	0.28	0.04	0.03	0.01	0.31	0.29
ACN	Autonomic Ganglia	Neuroblastoma	0.02	0.15	0.08	0.11	0.09	0.09	0.05	0.09	0.08	0.12	0.10	0.03	0.25	0.05	0.08	0.00	0.18	0.24
GI-ME-N	Autonomic Ganglia	Neuroblastoma	0.10	0.17	0.12	0.12	0.10	0.11	0.05	0.08	0.07	0.11	0.10	0.03	0.29	0.08	0.11	0.02	0.39	0.45
NB12	Autonomic Ganglia	Neuroblastoma	0.00	0.08	0.08	0.11	0.07	0.09	0.08	0.06	0.68	0.68	0.51	0.53	0.67	0.72	0.70	0.65	0.57	0.44
NB13	Autonomic Ganglia	Neuroblastoma	0.00	0.10	0.07	0.10	0.09	0.07	0.03	0.09	0.07	0.12	0.12	0.04	0.31	0.04	0.05	0.02	0.33	0.34
NBSUSSR	Autonomic Ganglia	Neuroblastoma	0.76	0.28	0.38	0.30	0.07	0.06	0.07	0.06	0.61	0.65	0.54	0.51	0.62	0.64	0.65	0.60	0.52	0.71
NB17	Autonomic Ganglia	Neuroblastoma	0.00	0.11	0.08	0.11	0.08	0.07	0.01	0.07	0.45	0.32	0.25	0.22	0.48	0.44	0.21	0.32	0.43	0.44
NB14	Autonomic Ganglia	Neuroblastoma	0.00	0.10	0.09	0.09	0.10	0.09	0.04	0.08	0.06	0.10	0.15	0.04	0.30	0.05	0.05	0.02	0.27	0.23
NB5	Autonomic Ganglia	Neuroblastoma	0.02	0.07	0.08	0.12	0.07	0.08	0.04	0.08	0.06	0.13	0.12	0.04	0.30	0.07	0.04	0.01	0.29	0.33
KP-N-YS	Autonomic Ganglia	Neuroblastoma	0.01	0.10	0.10	0.11	0.08	0.08	0.02	0.07	0.37	0.24	0.19	0.16	0.41	0.34	0.13	0.26	0.39	0.41
NB7	Autonomic Ganglia	Neuroblastoma	0.00	0.11	0.09	0.11	0.08	0.07	0.03	0.07	0.07	0.10	0.12	0.04	0.31	0.05	0.04	0.01	0.26	0.22
CHP-134	Autonomic Ganglia	Neuroblastoma	0.03	0.10	0.08	0.09	0.07	0.08	0.03	0.07	0.08	0.10	0.09	0.04	0.32	0.04	0.03	0.02	0.31	0.47
LAN-6	Autonomic Ganglia	Neuroblastoma	0.00	0.11	0.07	0.10	0.06	0.06	0.03	0.06	0.05	0.08	0.14	0.03	0.31	0.05	0.03	0.01	0.30	0.28
NB10	Autonomic Ganglia	Neuroblastoma	0.00	0.10	0.08	0.08	0.06	0.07	0.03	0.06	0.04	0.11	0.11	0.03	0.29	0.04	0.03	0.01	0.30	0.28
IMR-5	Autonomic Ganglia	Neuroblastoma	0.10	0.11	0.07	0.11	0.07	0.07	0.02	0.07	0.39	0.27	0.25	0.21	0.43	0.37	0.31	0.36	0.35	0.34
NB6	Autonomic Ganglia	Neuroblastoma	0.01	0.10	0.07	0.11	0.06	0.07	0.02	0.07	0.05	0.10	0.10	0.02	0.28	0.04	0.03	0.01	0.28	0.23
CHP-126	Autonomic Ganglia	Neuroblastoma	0.23	0.13	0.08	0.13	0.10	0.09	0.04	0.09	0.11	0.13	0.15	0.04	0.31	0.08	0.08	0.02	0.30	0.21
KP-N-RT-BM-1	Autonomic Ganglia	Neuroblastoma	0.00	0.09	0.07	0.09	0.06	0.06	0.02	0.04	0.67	0.60	0.45	0.49	0.58	0.59	0.61	0.60	0.51	0.63
KP-N-YN	Autonomic Ganglia	Neuroblastoma	0.02	0.15	0.08	0.10	0.07	0.07	0.03	0.07	0.06	0.09	0.08	0.03	0.30	0.04	0.09	0.00	0.28	0.26
SIMA	Autonomic Ganglia	Neuroblastoma	0.01	0.09	0.08	0.10	0.06	0.06	0.03	0.04	0.04	0.07	0.08	0.03	0.29	0.05	0.04	0.01	0.26	0.21
NH-12	Autonomic Ganglia	Neuroblastoma	0.03	0.10	0.08	0.10	0.06	0.07	0.02	0.05	0.05	0.09	0.07	0.02	0.28	0.03	0.02	0.00	0.38	0.42
TGW	Autonomic Ganglia	Neuroblastoma	0.01	0.12	0.08	0.10	0.08	0.09	0.04	0.07	0.38	0.19	0.15	0.08	0.41	0.17	0.18	0.17	0.32	0.35
NB1	Autonomic Ganglia	Neuroblastoma	0.00	0.13	0.09	0.09	0.08	0.09	0.03	0.08	0.29	0.15	0.10	0.03	0.32	0.06	0.07	0.01	0.31	0.26
EGI-1	Biliary Tract	Bile Duct Adenocarcinoma	0.27	0.10	0.10	0.10	0.06	0.08	0.07	0.07	0.06	0.12	0.11	0.03	0.03	0.04	0.03	0.01	0.08	0.36
ETK-1	Biliary Tract	Bile Duct Adenocarcinoma	0.03	0.10	0.06	0.09	0.06	0.06	0.04	0.05	0.05	0.11	0.10	0.02	0.06	0.04	0.02	0.01	0.04	0.20
HUCCT1	Biliary Tract	Bile Duct Carcinoma	0.06	0.13	0.09	0.10	0.08	0.10	0.02	0.10	0.06	0.10	0.11	0.02	0.04	0.04	0.05	0.00	0.03	0.17
TGBC1TKB	Biliary Tract	Gallbladder Adenocarcinoma	0.00	0.08	0.07	0.10	0.06	0.06	0.03	0.04	0.05	0.08	0.08	0.03	0.02	0.03	0.03	0.00	0.01	0.11
TGBC24TKB	Biliary Tract	Gallbladder Adenocarcinoma	0.02	0.10	0.08	0.12	0.07	0.07	0.06	0.06	0.06	0.12	0.10	0.04	0.04	0.05	0.05	0.01	0.07	0.13
CAL-29	Bladder	Bladder Transitional Cell Carcinoma	0.02	0.13	0.09	0.11	0.09	0.08	0.04	0.08	0.06	0.13	0.11	0.03	0.05	0.04	0.04	0.01	0.03	0.19
SCI-1	Blood	B Cell Lymphoma	0.08	0.13	0.09	0.11	0.08	0.10	0.03	0.07	0.08	0.13	0.11	0.03	0.34	0.06	0.09	0.01	0.51	0.32
KARPAS-1106P	Blood	B Cell Lymphoma	0.37	0.16	0.09	0.10	0.06	0.08	0.03	0.07	0.05	0.11	0.08	0.02	0.26	0.04	0.05	0.01	0.29	0.61
RCH-ACV	Blood	B Cell Precursor Leukemia	0.52	0.17	0.14	0.11	0.09	0.10	0.03	0.08	0.24	0.19	0.12	0.04	0.36	0.15	0.18	0.13	0.52	0.76
ALL-SIL	Blood	Leukemia (T Cell)	0.25	0.15	0.09	0.10	0.08	0.10	0.05	0.08	0.91	0.85	0.81	0.89	0.94	0.96	0.98	0.99	0.97	0.92
EJM	Blood	Multiple Myeloma	0.02	0.11	0.10	0.11	0.08	0.10	0.02	0.09	0.06	0.09	0.09	0.02	0.06	0.04	0.03	0.01	0.02	0.17
MOLP-8	Blood	Multiple Myeloma	0.02	0.11	0.10	0.11	0.07	0.08	0.04	0.08	0.06	0.11	0.11	0.03	0.27	0.04	0.04	0.01	0.01	0.32
SW1353	Bone	Chondrosarcoma	0.01	0.10	0.07	0.07	0.06	0.08	0.06	0.06	0.06	0.09	0.08	0.03	0.22	0.03	0.03	0.01	0.15	0.19
H-EMC-SS	Bone	Chondrosarcoma	0.23	0.12	0.12	0.13	0.09	0.10	0.04	0.08	0.10	0.14	0.16	0.04	0.33	0.07	0.06	0.01	0.38	0.58
CHSA8926	Bone	Chondrosarcoma	0.01	0.14	0.08	0.10	0.07	0.09	0.05	0.07	0.06	0.10	0.09	0.03	0.07	0.04	0.05	0.00	0.03	0.17
CAL-78	Bone	Chondrosarcoma	0.04	0.11	0.09	0.12	0.08	0.09	0.02	0.08	0.06	0.11	0.10	0.03	0.14	0.03	0.05	0.01	0.03	0.18
U-CH2	Bone	Chordoma	0.03	0.08	0.06	0.08	0.05	0.07	0.02	0.05	0.05	0.08	0.06	0.01	0.02	0.04	0.02	0.01	0.01	0.11
TC-71	Bone	Ewing's Sarcoma	0.00	0.11	0.08	0.11	0.09	0.07	0.06	0.08	0.89	0.79	0.73	0.45	0.90	0.94	0.95	0.99	0.89	0.94
ES4	Bone	Ewing'S Sarcoma-Peripheral Primitive Neuroectodermal Tumour	0.25	0.10	0.08	0.10	0.06	0.07	0.05	0.05	0.15	0.10	0.08	0.03	0.27	0.04	0.03	0.00	0.31	0.33
EW-18	Bone	Ewing'S Sarcoma-Peripheral Primitive Neuroectodermal Tumour	0.06	0.10	0.08	0.10	0.06	0.06	0.03	0.04	0.47	0.43	0.22	0.06	0.31	0.14	0.06	0.04	0.34	0.36
EW-22	Bone	Ewing'S Sarcoma-Peripheral Primitive Neuroectodermal Tumour	0.04	0.10	0.08	0.10	0.06	0.06	0.04	0.05	0.24	0.17	0.11	0.02	0.16	0.05	0.03	0.02	0.24	0.28
ES8	Bone	Ewing'S Sarcoma-Peripheral Primitive Neuroectodermal Tumour	0.03	0.11	0.08	0.11	0.07	0.07	0.03	0.06	0.18	0.11	0.09	0.02	0.26	0.04	0.03	0.01	0.28	0.29
ES1	Bone	Ewing'S Sarcoma-Peripheral Primitive Neuroectodermal Tumour	0.01	0.09	0.07	0.10	0.06	0.08	0.02	0.05	0.88	0.85	0.76	0.54	0.93	0.96	0.90	0.97	0.92	0.91
EW-13	Bone	Ewing'S Sarcoma-Peripheral Primitive Neuroectodermal Tumour	0.04	0.10	0.07	0.09	0.07	0.07	0.03	0.05	0.58	0.28	0.15	0.02	0.21	0.05	0.04	0.02	0.20	0.22
EW-16	Bone	Ewing'S Sarcoma-Peripheral Primitive Neuroectodermal Tumour	0.50	0.12	0.07	0.12	0.06	0.07	0.04	0.05	0.17	0.14	0.11	0.12	0.37	0.19	0.10	0.18	0.33	0.37
ES7	Bone	Ewing'S Sarcoma-Peripheral Primitive Neuroectodermal Tumour	0.03	0.12	0.08	0.10	0.07	0.07	0.03	0.04	0.65	0.76	0.26	0.08	0.54	0.59	0.42	0.47	0.62	0.53
ES5	Bone	Ewing'S Sarcoma-Peripheral Primitive Neuroectodermal Tumour	0.16	0.10	0.09	0.11	0.06	0.07	0.03	0.05	0.82	0.76	0.50	0.43	0.85	0.85	0.75	0.72	0.59	0.61
EW-1	Bone	Ewing'S Sarcoma-Peripheral Primitive Neuroectodermal Tumour	0.03	0.11	0.12	0.10	0.06	0.07	0.03	0.05	0.81	0.75	0.33	0.16	0.60	0.72	0.46	0.50	0.59	0.83
EW-24	Bone	Ewing'S Sarcoma-Peripheral Primitive Neuroectodermal Tumour	0.02	0.10	0.09	0.10	0.07	0.07	0.03	0.04	0.64	0.41	0.30	0.20	0.53	0.61	0.44	0.54	0.55	0.67
EW-7	Bone	Ewing'S Sarcoma-Peripheral Primitive Neuroectodermal Tumour	0.03	0.10	0.06	0.08	0.06	0.07	0.03	0.05	0.84	0.83	0.63	0.89	0.95	0.96	0.98	0.99	0.96	0.93
SK-PN-DW	Bone	Ewing'S Sarcoma-Peripheral Primitive Neuroectodermal Tumour	0.02	0.11	0.07	0.10	0.07	0.06	0.03	0.05	0.84	0.76	0.69	0.44	0.75	0.88	0.79	0.84	0.87	0.83
EW-3	Bone	Ewing'S Sarcoma-Peripheral Primitive Neuroectodermal Tumour	0.03	0.11	0.07	0.11	0.06	0.07	0.04	0.05	0.40	0.30	0.17	0.05	0.23	0.11	0.08	0.06	0.44	0.56
ES3	Bone	Ewing'S Sarcoma-Peripheral Primitive Neuroectodermal Tumour	0.03	0.10	0.07	0.09	0.05	0.06	0.03	0.06	0.82	0.70	0.50	0.41	0.64	0.70	0.63	0.70	0.55	0.67
EW-11	Bone	Ewing'S Sarcoma-Peripheral Primitive Neuroectodermal Tumour	0.15	0.10	0.07	0.10	0.05	0.07	0.02	0.04	0.80	0.67	0.42	0.31	0.59	0.68	0.52	0.62	0.57	0.63
EW-12	Bone	Ewing'S Sarcoma-Peripheral Primitive Neuroectodermal Tumour	0.04	0.10	0.08	0.10	0.06	0.06	0.03	0.05	0.65	0.18	0.19	0.02	0.32	0.11	0.08	0.05	0.22	0.19
MHH-ES-1	Bone	Ewing'S Sarcoma-Peripheral Primitive Neuroectodermal Tumour	0.63	0.16	0.12	0.12	0.07	0.08	0.03	0.07	0.84	0.62	0.31	0.26	0.48	0.49	0.41	0.36	0.52	0.40
CADO-ES1	Bone	Ewing'S Sarcoma-Peripheral Primitive Neuroectodermal Tumour	0.07	0.15	0.09	0.10	0.08	0.09	0.05	0.08	0.40	0.30	0.19	0.19	0.45	0.36	0.23	0.31	0.47	0.65
SJSA-1	Bone	Multipotential Sarcoma	0.01	0.11	0.08	0.11	0.07	0.08	0.03	0.06	0.07	0.09	0.09	0.02	0.12	0.06	0.12	0.02	0.11	0.12
G-292-CLONE-A141B1	Bone	Osteosarcoma	0.05	0.12	0.09	0.09	0.08	0.10	0.05	0.07	0.09	0.13	0.13	0.02	0.32	0.07	0.07	0.01	0.33	0.29
SAOS-2	Bone	Osteosarcoma	0.01	0.10	0.08	0.09	0.07	0.07	0.04	0.05	0.05	0.09	0.08	0.02	0.12	0.03	0.03	0.01	0.09	0.21
MG-63	Bone	Osteosarcoma	0.02	0.12	0.08	0.10	0.06	0.08	0.03	0.05	0.05	0.09	0.08	0.02	0.27	0.04	0.05	0.01	0.27	0.27
NOS-1	Bone	Osteosarcoma	0.03	0.11	0.08	0.09	0.06	0.09	0.02	0.05	0.06	0.09	0.08	0.02	0.18	0.03	0.04	0.00	0.04	0.17
CAL-72	Bone	Osteosarcoma	0.02	0.12	0.08	0.11	0.08	0.07	0.03	0.05	0.06	0.09	0.08	0.03	0.19	0.04	0.03	0.01	0.19	0.22
HOS	Bone	Osteosarcoma	0.01	0.12	0.08	0.10	0.06	0.07	0.02	0.05	0.05	0.08	0.08	0.02	0.28	0.04	0.03	0.01	0.27	0.20
HUO-3N1	Bone	Osteosarcoma	0.01	0.09	0.07	0.10	0.06	0.07	0.02	0.05	0.06	0.10	0.09	0.02	0.21	0.03	0.03	0.00	0.12	0.21
HUO9	Bone	Osteosarcoma	0.00	0.09	0.09	0.12	0.07	0.07	0.03	0.05	0.03	0.09	0.08	0.03	0.02	0.01	0.02	0.01	0.01	0.08
NY	Bone	Osteosarcoma	0.02	0.10	0.07	0.10	0.05	0.06	0.03	0.05	0.04	0.09	0.08	0.03	0.18	0.03	0.02	0.01	0.11	0.16
U-2-OS	Bone	Osteosarcoma	0.01	0.13	0.09	0.12	0.09	0.09	0.04	0.08	0.05	0.09	0.09	0.03	0.30	0.04	0.06	0.02	0.17	0.23
SK-ES-1	Bone	Sarcoma Peripheral Primitive Neuroectodermal	0.11	0.11	0.08	0.09	0.06	0.06	0.02	0.06	0.87	0.64	0.51	0.42	0.66	0.82	0.81	0.80	0.92	0.86
ES6	Bone	Sarcoma Peripheral Primitive Neuroectodermal	0.01	0.08	0.06	0.08	0.05	0.06	0.02	0.05	0.04	0.08	0.06	0.01	0.25	0.03	0.02	0.01	0.22	0.23
SCCH-196	Bone	Small Round Cell Sarcoma	0.12	0.15	0.09	0.12	0.09	0.09	0.05	0.08	0.85	0.55	0.31	0.23	0.56	0.56	0.37	0.49	0.66	0.64
HCC1428	Breast	Breast Adenocarcinoma	0.16	0.10	0.05	0.08	0.05	0.05	0.03	0.04	0.04	0.07	0.06	0.02	0.31	0.02	0.02	0.01	0.29	0.23
MDA-MB-231	Breast	Breast Carcinoma	0.00	0.07	0.05	0.09	0.05	0.05	0.01	0.05	0.04	0.06	0.06	0.00	0.01	0.02	0.01	0.00	0.03	0.09
MCF7	Breast	Breast Carcinoma	0.03	0.08	0.07	0.09	0.06	0.06	0.02	0.06	0.04	0.08	0.07	0.02	0.29	0.02	0.03	0.00	0.29	0.22
MDA-MB-468	Breast	Breast Carcinoma	0.94	0.47	0.42	0.33	0.06	0.06	0.01	0.06	0.05	0.08	0.08	0.02	0.27	0.02	0.03	0.00	0.23	0.56
MDA-MB-436	Breast	Breast Carcinoma	0.05	0.07	0.06	0.09	0.05	0.05	0.03	0.05	0.04	0.09	0.06	0.02	0.28	0.04	0.04	0.01	0.22	0.24
CAL-85-1	Breast	Breast Carcinoma	0.00	0.08	0.07	0.10	0.05	0.06	0.01	0.05	0.03	0.07	0.08	0.02	0.26	0.03	0.03	0.01	0.18	0.11
MDA-MB-453	Breast	Breast Carcinoma	0.11	0.09	0.06	0.10	0.06	0.06	0.02	0.05	0.03	0.09	0.07	0.02	0.31	0.04	0.03	0.01	0.26	0.24
OCUB-M	Breast	Breast Carcinoma	0.02	0.09	0.05	0.10	0.04	0.07	0.01	0.05	0.05	0.08	0.11	0.02	0.29	0.03	0.04	0.01	0.27	0.21
EFM-192A	Breast	Breast Carcinoma	0.00	0.09	0.06	0.10	0.07	0.05	0.02	0.05	0.05	0.08	0.11	0.01	0.29	0.03	0.02	0.00	0.29	0.22
HDQ-P1	Breast	Breast Carcinoma	0.02	0.09	0.07	0.09	0.05	0.07	0.02	0.05	0.04	0.07	0.11	0.01	0.27	0.04	0.03	0.01	0.26	0.21
CAL-120	Breast	Breast Carcinoma	0.16	0.09	0.07	0.07	0.06	0.08	0.01	0.04	0.05	0.08	0.10	0.01	0.07	0.02	0.02	0.00	0.12	0.24
CAMA-1	Breast	Breast Carcinoma	0.01	0.08	0.08	0.09	0.07	0.05	0.03	0.06	0.06	0.09	0.11	0.03	0.27	0.03	0.02	0.01	0.27	0.29
CAL-51	Breast	Breast Carcinoma	0.01	0.09	0.06	0.11	0.08	0.07	0.03	0.06	0.05	0.09	0.13	0.02	0.28	0.04	0.03	0.00	0.31	0.22
BT-20	Breast	Breast Carcinoma	0.06	0.09	0.05	0.09	0.05	0.06	0.02	0.05	0.04	0.08	0.06	0.01	0.28	0.03	0.03	0.01	0.27	0.19
COLO-824	Breast	Breast Carcinoma	0.46	0.33	0.42	0.36	0.14	0.10	0.02	0.07	0.33	0.25	0.18	0.05	0.37	0.16	0.07	0.04	0.43	0.50
AU565	Breast	Breast Carcinoma	0.01	0.09	0.07	0.12	0.06	0.06	0.03	0.07	0.04	0.10	0.10	0.02	0.32	0.04	0.03	0.01	0.30	0.25

Cell Line	Tissue	Cell Line Type	cg21539223	cg16456337	cg15956794	cg14773728	cg17573603	cg17919331	cg23965551	cg00522636	cg13518195	cg26459500	cg01092811	cg06363041	cg25437259	cg14506646	cg14861803	cg11162249	cg17789800	cg00261665
		UCSC_REFGENE_NAME	DCP2								NUDT16									
		UCSC_REFGENE_GROUP	TSS1500	TSS1500	TSS200	TSS200	TSS200	TSS200	1stExon;5'UTR	1stExon	TSS1500	TSS1500	TSS1500	TSS200	TSS200	TSS200	TSS200	5'UTR;1stExon	Body	Body
EVSA-T	Breast	Breast Carcinoma	0.09	0.10	0.08	0.09	0.07	0.08	0.04	0.06	0.40	0.33	0.31	0.03	0.46	0.22	0.09	0.06	0.41	0.33
MDA-MB-361	Breast	Breast Carcinoma	0.04	0.09	0.06	0.10	0.06	0.07	0.04	0.04	0.04	0.08	0.07	0.03	0.29	0.03	0.03	0.01	0.30	0.22
HCC1569	Breast	Breast Carcinoma	0.63	0.29	0.48	0.46	0.09	0.08	0.04	0.07	0.05	0.09	0.12	0.03	0.28	0.05	0.37	0.01	0.34	0.28
MRK-NU-1	Breast	Breast Carcinoma	0.01	0.15	0.09	0.10	0.08	0.09	0.05	0.08	0.19	0.15	0.11	0.02	0.37	0.11	0.13	0.07	0.34	0.36
MDA-MB-415	Breast	Breast Carcinoma	0.03	0.10	0.08	0.10	0.07	0.09	0.03	0.07	0.06	0.06	0.07	0.02	0.02	0.03	0.01	0.00	0.12	0.16
HS-578-T	Breast	Breast Carcinoma	0.00	0.13	0.08	0.10	0.08	0.08	0.03	0.07	0.05	0.08	0.10	0.03	0.31	0.04	0.07	0.01	0.25	0.25
YMB-1-E	Breast	Breast Carcinoma	0.04	0.07	0.06	0.09	0.06	0.06	0.04	0.05	0.04	0.08	0.07	0.02	0.27	0.04	0.03	0.01	0.26	0.20
T47D	Breast	Breast Ductal Carcinoma	0.01	0.08	0.07	0.08	0.05	0.05	0.00	0.05	0.04	0.07	0.07	0.01	0.10	0.01	0.02	0.00	0.19	0.14
BT-549	Breast	Breast Ductal Carcinoma	0.01	0.09	0.10	0.10	0.06	0.06	0.02	0.06	0.04	0.08	0.08	0.02	0.29	0.03	0.03	0.01	0.28	0.20
MDA-MB-134-VI	Breast	Breast Ductal Carcinoma	0.03	0.07	0.06	0.09	0.05	0.05	0.01	0.05	0.04	0.08	0.08	0.02	0.24	0.02	0.02	0.01	0.24	0.18
HCC38	Breast	Breast Ductal Carcinoma	0.21	0.12	0.07	0.09	0.07	0.05	0.02	0.05	0.05	0.08	0.08	0.03	0.29	0.04	0.07	0.01	0.29	0.27
BT-474	Breast	Breast Ductal Carcinoma	0.03	0.12	0.06	0.09	0.05	0.05	0.03	0.05	0.06	0.09	0.08	0.02	0.27	0.06	0.05	0.01	0.27	0.25
UACC-812	Breast	Breast Ductal Carcinoma	0.02	0.09	0.08	0.09	0.08	0.06	0.02	0.07	0.06	0.11	0.09	0.03	0.31	0.04	0.04	0.01	0.26	0.21
CAL-148	Breast	Breast Ductal Carcinoma	0.01	0.08	0.08	0.09	0.06	0.06	0.02	0.05	0.03	0.08	0.08	0.02	0.28	0.03	0.03	0.01	0.26	0.20
BT-483	Breast	Breast Ductal Carcinoma	0.01	0.08	0.07	0.08	0.05	0.06	0.02	0.04	0.04	0.09	0.07	0.00	0.27	0.02	0.02	0.00	0.30	0.23
EFM-19	Breast	Breast Ductal Carcinoma	0.03	0.08	0.07	0.09	0.06	0.06	0.03	0.04	0.05	0.07	0.08	0.02	0.24	0.03	0.03	0.00	0.30	0.23
UACC-893	Breast	Breast Ductal Carcinoma	0.01	0.10	0.08	0.09	0.07	0.04	0.05	0.06	0.07	0.10	0.14	0.03	0.31	0.03	0.04	0.01	0.28	0.25
ZR-75-30	Breast	Breast Ductal Carcinoma	0.00	0.09	0.09	0.12	0.07	0.07	0.03	0.06	0.06	0.11	0.11	0.02	0.25	0.03	0.03	0.01	0.35	0.36
MFM-223	Breast	Breast Ductal Carcinoma	0.90	0.49	0.47	0.49	0.44	0.47	0.59	0.38	0.06	0.11	0.14	0.03	0.30	0.04	0.03	0.01	0.30	0.50
HCC2157	Breast	Breast Ductal Carcinoma	0.01	0.09	0.07	0.10	0.05	0.07	0.02	0.04	0.04	0.10	0.09	0.02	0.28	0.04	0.31	0.00	0.33	0.32
HCC70	Breast	Breast Ductal Carcinoma	0.16	0.13	0.15	0.14	0.07	0.08	0.03	0.08	0.06	0.12	0.14	0.04	0.26	0.05	0.04	0.01	0.25	0.20
HCC1954	Breast	Breast Ductal Carcinoma	0.21	0.09	0.10	0.12	0.06	0.09	0.03	0.08	0.10	0.10	0.14	0.02	0.32	0.04	0.04	0.02	0.30	0.25
HCC1187	Breast	Breast Ductal Carcinoma	0.04	0.13	0.18	0.17	0.08	0.08	0.03	0.07	0.09	0.15	0.17	0.03	0.30	0.05	0.04	0.01	0.33	0.31
HCC2218	Breast	Breast Ductal Carcinoma	0.01	0.10	0.07	0.10	0.07	0.08	0.03	0.06	0.05	0.10	0.09	0.03	0.30	0.04	0.04	0.01	0.29	0.19
MDA-MB-157	Breast	Breast Ductal Carcinoma	0.01	0.10	0.09	0.11	0.06	0.05	0.04	0.05	0.08	0.07	0.07	0.02	0.28	0.03	0.03	0.02	0.22	0.31
HCC1599	Breast	Breast Ductal Carcinoma	0.00	0.10	0.08	0.09	0.06	0.06	0.02	0.06	0.33	0.21	0.22	0.05	0.38	0.22	0.08	0.10	0.33	0.32
HCC202	Breast	Breast Ductal Carcinoma	0.01	0.10	0.07	0.08	0.06	0.05	0.02	0.04	0.05	0.07	0.07	0.02	0.31	0.03	0.03	0.01	0.30	0.24
HCC1500	Breast	Breast Ductal Carcinoma	0.03	0.12	0.08	0.09	0.06	0.09	0.02	0.05	0.04	0.08	0.06	0.02	0.28	0.04	0.03	0.01	0.29	0.23
DU-4475	Breast	Breast Ductal Carcinoma	0.00	0.11	0.07	0.08	0.06	0.07	0.03	0.04	0.03	0.07	0.06	0.01	0.02	0.03	0.02	0.01	0.01	0.10
HCC1419	Breast	Breast Ductal Carcinoma	0.01	0.07	0.08	0.10	0.08	0.08	0.03	0.07	0.06	0.11	0.10	0.02	0.32	0.03	0.03	0.00	0.29	0.26
HCC1395	Breast	Breast Ductal Carcinoma	0.02	0.09	0.07	0.08	0.06	0.07	0.01	0.03	0.47	0.49	0.23	0.04	0.42	0.33	0.19	0.33	0.40	0.61
HCC1143	Breast	Breast Ductal Carcinoma	0.01	0.14	0.09	0.12	0.08	0.11	0.05	0.08	0.06	0.10	0.08	0.03	0.30	0.05	0.08	0.01	0.28	0.23
HCC1937	Breast	Breast Ductal Carcinoma	0.27	0.15	0.09	0.11	0.09	0.09	0.04	0.08	0.06	0.11	0.10	0.02	0.26	0.05	0.08	0.01	0.29	0.31
HCC1806	Breast	Breast Squamous Cell Carcinoma	0.01	0.12	0.11	0.14	0.09	0.10	0.05	0.10	0.08	0.14	0.19	0.04	0.24	0.06	0.05	0.02	0.31	0.23
MDA-MB-330	Breast	Mixed	0.00	0.12	0.08	0.11	0.08	0.09	0.04	0.08	0.05	0.13	0.10	0.02	0.32	0.05	0.08	0.01	0.32	0.39
CS1	Cartilage	Ns	0.02	0.14	0.15	0.12	0.09	0.08	0.05	0.07	0.05	0.10	0.12	0.02	0.33	0.05	0.08	0.01	0.24	0.25
U251	Central Nervous System	Astrocytoma	0.02	0.07	0.07	0.06	0.05	0.05	0.01	0.05	0.05	0.08	0.07	0.02	0.28	0.03	0.04	0.01	0.34	0.46
SF295	Central Nervous System	Astrocytoma	0.02	0.08	0.07	0.09	0.05	0.06	0.02	0.05	0.03	0.08	0.06	0.01	0.20	0.03	0.02	0.01	0.03	0.20
T98G	Central Nervous System	Astrocytoma	0.26	0.13	0.09	0.12	0.07	0.08	0.02	0.08	0.08	0.10	0.09	0.02	0.27	0.03	0.05	0.01	0.14	0.21
LN-18	Central Nervous System	Astrocytoma	0.00	0.07	0.10	0.11	0.07	0.08	0.02	0.07	0.06	0.09	0.09	0.02	0.28	0.02	0.03	0.01	0.13	0.18
D-336MG	Central Nervous System	Astrocytoma	0.01	0.11	0.09	0.11	0.08	0.09	0.04	0.06	0.06	0.10	0.12	0.04	0.12	0.04	0.04	0.01	0.04	0.15
NO-10	Central Nervous System	Astrocytoma	0.17	0.11	0.09	0.09	0.07	0.07	0.03	0.07	0.04	0.09	0.10	0.02	0.29	0.05	0.04	0.01	0.32	0.24
NO-11	Central Nervous System	Astrocytoma	0.02	0.10	0.07	0.10	0.07	0.08	0.04	0.05	0.06	0.07	0.10	0.01	0.30	0.03	0.02	0.01	0.44	0.44
KINGS-1	Central Nervous System	Astrocytoma	0.03	0.10	0.08	0.10	0.08	0.06	0.03	0.06	0.05	0.11	0.12	0.02	0.16	0.04	0.03	0.02	0.11	0.18
D-263MG	Central Nervous System	Astrocytoma	0.02	0.10	0.09	0.09	0.08	0.07	0.03	0.06	0.06	0.10	0.09	0.02	0.31	0.06	0.03	0.01	0.21	0.23
D-247MG	Central Nervous System	Astrocytoma	0.02	0.11	0.07	0.10	0.06	0.07	0.03	0.05	0.05	0.08	0.08	0.03	0.26	0.04	0.03	0.01	0.14	0.21
D-502MG	Central Nervous System	Astrocytoma	0.02	0.10	0.09	0.12	0.08	0.08	0.05	0.06	0.06	0.12	0.13	0.03	0.24	0.06	0.03	0.01	0.22	0.26
D-392MG	Central Nervous System	Astrocytoma	0.01	0.12	0.09	0.10	0.07	0.08	0.05	0.06	0.04	0.12	0.11	0.02	0.29	0.05	0.04	0.02	0.26	0.24
D-245MG	Central Nervous System	Astrocytoma	0.05	0.14	0.11	0.15	0.11	0.12	0.05	0.10	0.08	0.11	0.16	0.04	0.32	0.07	0.09	0.01	0.28	0.31
D-566MG	Central Nervous System	Astrocytoma	0.00	0.14	0.08	0.12	0.08	0.09	0.03	0.07	0.07	0.11	0.11	0.03	0.11	0.04	0.05	0.01	0.11	0.18
MOG-G-CCM	Central Nervous System	Astrocytoma	0.01	0.09	0.08	0.10	0.06	0.07	0.03	0.05	0.05	0.09	0.08	0.02	0.06	0.03	0.02	0.01	0.09	0.16
MOG-G-UVW	Central Nervous System	Astrocytoma	0.02	0.13	0.09	0.11	0.08	0.09	0.03	0.09	0.07	0.10	0.09	0.02	0.17	0.04	0.03	0.01	0.03	0.22
D-423MG	Central Nervous System	Astrocytoma	0.05	0.08	0.08	0.10	0.08	0.07	0.02	0.06	0.05	0.10	0.08	0.01	0.12	0.02	0.02	0.01	0.05	0.15
KS-1	Central Nervous System	Glioblastoma Multiforme	0.04	0.11	0.06	0.09	0.05	0.06	0.04	0.06	0.04	0.09	0.11	0.03	0.28	0.02	0.02	0.00	0.28	0.24
AM-38	Central Nervous System	Glioblastoma Multiforme	0.48	0.13	0.08	0.12	0.09	0.08	0.06	0.07	0.06	0.14	0.15	0.04	0.33	0.05	0.04	0.01	0.12	0.20
CAS-1	Central Nervous System	Glioblastoma Multiforme	0.01	0.08	0.08	0.10	0.06	0.07	0.02	0.05	0.05	0.08	0.09	0.02	0.27	0.05	0.02	0.00	0.11	0.20
BECKER	Central Nervous System	Glioblastoma Multiforme	0.02	0.10	0.06	0.09	0.06	0.07	0.03	0.05	0.04	0.09	0.08	0.01	0.30	0.03	0.02	0.00	0.10	0.20
DK-MG	Central Nervous System	Glioblastoma Multiforme	0.12	0.11	0.07	0.09	0.07	0.07	0.03	0.06	0.05	0.09	0.08	0.03	0.28	0.05	0.03	0.01	0.12	0.21
M059K	Central Nervous System	Glioblastoma Multiforme	0.11	0.11	0.08	0.10	0.07	0.08	0.02	0.05	0.05	0.09	0.08	0.02	0.25	0.03	0.02	0.01	0.11	0.17
M059J	Central Nervous System	Glioblastoma Multiforme	0.00	0.08	0.07	0.09	0.06	0.08	0.02	0.05	0.04	0.07	0.08	0.03	0.24	0.04	0.03	0.00	0.16	0.22
LN-229	Central Nervous System	Glioblastoma Multiforme	0.02	0.11	0.07	0.10	0.06	0.08	0.01	0.08	0.06	0.11	0.08	0.02	0.26	0.04	0.03	0.01	0.10	0.17
DBTRG-05MG	Central Nervous System	Glioblastoma Multiforme	0.01	0.12	0.08	0.10	0.08	0.08	0.03	0.06	0.06	0.11	0.09	0.02	0.21	0.06	0.03	0.01	0.25	0.18
GMS-10	Central Nervous System	Glioblastoma Multiforme	0.06	0.11	0.08	0.10	0.07	0.07	0.03	0.07	0.06	0.12	0.09	0.02	0.28	0.05	0.03	0.01	0.25	0.23
LN-405	Central Nervous System	Glioblastoma Multiforme	0.03	0.11	0.08	0.09	0.08	0.06	0.03	0.07	0.05	0.11	0.08	0.03	0.26	0.03	0.03	0.01	0.24	0.22
LNZTA3WT4	Central Nervous System	Glioblastoma Multiforme	0.12	0.13	0.08	0.11	0.07	0.07	0.06	0.06	0.07	0.10	0.09	0.03	0.32	0.06	0.08	0.01	0.27	0.26
CCF-STTG1	Central Nervous System	Glioblastoma Multiforme	0.02	0.12	0.09	0.10	0.07	0.08	0.07	0.07	0.05	0.13	0.09	0.03	0.29	0.05	0.04	0.01	0.20	0.18
NMC-G1	Central Nervous System	Glioma	0.13	0.10	0.07	0.10	0.06	0.07	0.03	0.05	0.04	0.09	0.07	0.03	0.20	0.05	0.04	0.00	0.12	0.17
KALS-1	Central Nervous System	Glioma	0.01	0.11	0.09	0.09	0.07	0.07	0.03	0.06	0.04	0.10	0.09	0.02	0.21	0.04	0.03	0.01	0.19	0.23
D-542MG	Central Nervous System	Glioma	0.00	0.12	0.07	0.09	0.07	0.08	0.04	0.05	0.06	0.10	0.11	0.03	0.30	0.04	0.04	0.01	0.39	0.39
KNS-42	Central Nervous System	Glioma	0.00	0.11	0.08	0.09	0.06	0.06	0.05	0.05	0.04	0.09	0.07	0.02	0.29	0.03	0.03	0.01	0.29	0.24
SK-MG-1	Central Nervous System	Glioma	0.01	0.12	0.08	0.09	0.07	0.07	0.03	0.05	0.05	0.09	0.08	0.02	0.24	0.04	0.03	0.01	0.34	0.35
YH-13	Central Nervous System	Glioma	0.01	0.10	0.08	0.11	0.08	0.08	0.04	0.06	0.05	0.13	0.11	0.03	0.30	0.03	0.04	0.01	0.10	0.17
SNB75	Central Nervous System	Glioma	0.02	0.11	0.09	0.10	0.08	0.08	0.05	0.07	0.05	0.14	0.13	0.02	0.25	0.05	0.03	0.01	0.10	0.19
KNS-81-FD	Central Nervous System	Glioma	0.04	0.10	0.08	0.10	0.06	0.08	0.04	0.06	0.07	0.10	0.11	0.03	0.29	0.05	0.05	0.01	0.25	0.33
GB-1	Central Nervous System	Glioma	0.33	0.09	0.07	0.07	0.07	0.07	0.04	0.04	0.07	0.11	0.07	0.02	0.29	0.03	0.03	0.01	0.40	0.39
8-MG-BA	Central Nervous System	Glioma	0.00	0.07	0.08	0.07	0.06	0.05	0.03	0.04	0.05	0.08	0.08	0.02	0.29	0.03	0.02	0.01	0.31	0.23
GAMG	Central Nervous System	Glioma	0.08	0.11	0.07	0.11	0.06	0.07	0.04	0.06	0.05	0.09	0.07	0.02	0.25	0.03	0.04	0.01	0.14	0.21
YKG-1	Central Nervous System	Glioma	0.00	0.09	0.07	0.10	0.06	0.07	0.02	0.07	0.05	0.08	0.09	0.02	0.23	0.04	0.03	0.01	0.11	0.17
SW1088	Central Nervous System	Glioma	0.01	0.09	0.08	0.10	0.06	0.07	0.04	0.06	0.05	0.09	0.09	0.02	0.27	0.04	0.03	0.01	0.07	0.15
H4	Central Nervous System	Glioma	0.50	0.10	0.08	0.09	0.06	0.07	0.03	0.06	0.05	0.09	0.08	0.01	0.29	0.04	0.02	0.01	0.42	0.37
HS-683	Central Nervous System	Glioma	0.01	0.09	0.09	0.10	0.07	0.08	0.03	0.05	0.05	0.10	0.09	0.02	0.16	0.02	0.02	0.01	0.09	0.12
42-MG-BA	Central Nervous System	Glioma	0.15	0.09	0.07	0.09	0.06	0.06	0.02	0.05	0.04	0.08	0.07	0.02	0.31	0.03	0.02	0.00	0.25	0.20
SW1783	Central Nervous System	Glioma	0.02	0.11	0.09	0.11	0.06	0.08	0.03	0.07	0.05	0.11	0.10	0.03	0.11	0.04	0.03	0.01	0.03	0.10
U-118-MG	Central Nervous System	Glioma	0.06	0.11	0.07	0.09	0.08	0.07	0.05	0.06	0.06	0.13	0.10	0.02	0.30	0.03	0.03	0.01	0.21	0.18
SF126	Central Nervous System	Glioma	0.61	0.13	0.08	0.11	0.07	0.09	0.05	0.08	0.07	0.12	0.11	0.03	0.32	0.06	0.08	0.01	0.29	0.41
SF539	Central Nervous System	Glioma	0.01	0.14	0.09	0.12	0.09	0.09	0.03	0.09	0.06	0.10	0.10	0.03	0.30	0.04	0.08	0.01	0.26	0.24
A172	Central Nervous System	Glioma	0.12	0.15	0.08	0.11	0.09	0.09	0.04	0.09	0.08	0.11	0.11	0.03	0.33	0.05	0.10	0.01	0.33	0.32
U-87-MG	Central Nervous System	Glioma	0.00	0.11	0.09	0.14	0.09	0.08	0.01	0.11	0.10	0.20	0.13	0.01	0.27	0.05	0.08	0.00	0.22	0.25
SF268	Central Nervous System	Glioma	0.05	0.17	0.09	0.13	0.09	0.09	0.04	0.10	0.09	0.12	0.12	0.03	0.28	0.06	0.11	0.01	0.24	0.28
GI-1	Central Nervous System	Gliosarcoma	0.10	0.11	0.08	0.13	0.07	0.07	0.03	0.05	0.04	0.10	0.09	0.03	0.31	0.03	0.03	0.01	0.28	0.25
D-283MED	Central Nervous System	Primitive Neuroectodermal Tumour-Medulloblastoma	0.92	0.54	0.50	0.51	0.11	0.08	0.05	0.06	0.51	0.46	0.49	0.45	0.55	0.60	0.59	0.58	0.47	0.57
ONS-76	Central Nervous System	Primitive Neuroectodermal Tumour-Medulloblastoma	0.00	0.08	0.08	0.09	0.07	0.06	0.03	0.05	0.05	0.09	0.08	0.01	0.30	0.03	0.03	0.00	0.23	0.39
PFSK-1	Central Nervous System	Primitive Neuroectodermal Tumour-Medulloblastoma	0.01	0.11	0.08	0.10	0.07	0.08	0.03	0.06	0.04	0.11	0.07	0.02	0.32	0.03	0.03	0.01	0.15	0.18
DAOY	Central Nervous System	Primitive Neuroectodermal Tumour-Medulloblastoma	0.03	0.09	0.08	0.10	0.08	0.07	0.02	0.07	0.06	0.10	0.09	0.02	0.18	0.03	0.03	0.01	0.06	0.13
SISO	Cervix	Cervix Adenocarcinoma	0.18	0.14	0.09	0.11	0.08	0.11	0.05	0.09	0.13	0.19	0.13	0.03	0.31	0.06	0.09	0.02	0.49	0.59
HELA	Cervix	Cervix Adenocarcinoma	0.99	0.96	0.65	0.59	0.12	0.08	0.04	0.07	0.05	0.10	0.08	0.03	0.06	0.05	0.04	0.01	0.02	0.18
HELASF	Cervix	Cervix Carcinoma	1.00	0.96	0.68	0.61	0.15	0.09	0.03	0.06	0.05	0.11	0.08	0.03	0.05	0.03	0.03	0.01	0.03	0.15
C-33-A	Cervix	Cervix Carcinoma	0.01	0.13	0.08	0.11	0.09	0.09	0.06	0.10	0.22	0.18	0.15	0.04	0.36	0.18	0.14	0.05	0.38	0.44
BOKU	Cervix	Cervix Carcinoma	0.30	0.24	0.22	0.14	0.07	0.09	0.04	0.07	0.08	0.11	0.12	0.02	0.05	0.06	0.05	0.02	0.10	0.37
DOTC2-4510	Cervix	Cervix Carcinoma	0.01	0.14	0.09	0.11	0.09	0.08	0.04	0.09	0.07	0.12	0.11	0.03	0.03	0.05	0.03	0.02	0.02	0.18
SKG-IIIA	Cervix	Cervix Carcinoma	0.86	0.28	0.13	0.12	0.08	0.08	0.04	0.08	0.06	0.12	0.09	0.02	0.04	0.04	0.04	0.01	0.04	0.19
SW756	Cervix	Cervix Carcinoma	0.99	0.77	0.67	0.70	0.65	0.52	0.49	0.30	0.05	0.09	0.09	0.02	0.28	0.06	0.06	0.01	0.31	0.33
ME-180	Cervix	Cervix Carcinoma	0.09	0.15	0.08	0.11	0.07	0.09	0.04	0.08	0.07	0.12	0.13	0.04	0.30	0.07	0.09	0.02	0.39	0.49
MS751	Cervix	Cervix Carcinoma	0.09	0.14	0.08	0.12	0.09	0.10	0.05	0.08	0.07	0.14	0.13	0.04	0.06	0.07	0.05	0.01	0.07	0.22
HT-3	Cervix	Cervix Carcinoma	0.70	0.18	0.10	0.12	0.09	0.08	0.03	0.08	0.48	0.46	0.42	0.33	0.55	0.55	0.57	0.53	0.51	0.70
C-4-I	Cervix	Cervix Carcinoma	0.02	0.16	0.10	0.11	0.09	0.10	0.06	0.09	0.07	0.13	0.10	0.03	0.03	0.04	0.03	0.01	0.03	0.18
TC-YIK	Cervix	Cervix Small Cell Carcinoma	0.02	0.13	0.08	0.11	0.09	0.08	0.05	0.08	0.06	0.13	0.11	0.05	0.06	0.06	0.04	0.01	0.13	0.18

Cell Line	Tissue	Cell Line Type	cg21539223	cg16456337	cg15956794	cg14773728	cg17573603	cg17919331	cg23965551	cg00522636	cg13518195	cg26459500	cg01092811	cg06363041	cg25437259	cg14506646	cg14861803	cg11162249	cg17789800	cg00261665
		UCSC_REFGENE_NAME	DCP2								NUDT16									
		UCSC_REFGENE_GROUP	TSS1500	TSS1500	TSS200	TSS200	TSS200	TSS200	1stExon;5'UTR	1stExon	TSS1500	TSS1500	TSS1500	TSS200	TSS200	TSS200	TSS200	5'UTR;1stExon	Body	Body
OMC-1	Cervix	Cervix Squamous Cell Carcinoma	0.11	0.13	0.09	0.11	0.07	0.09	0.04	0.08	0.06	0.11	0.12	0.03	0.08	0.05	0.04	0.01	0.24	0.24
SIHA	Cervix	Cervix Squamous Cell Carcinoma	0.33	0.13	0.08	0.12	0.08	0.11	0.03	0.07	0.19	0.18	0.11	0.03	0.07	0.06	0.05	0.01	0.18	0.34
CA-SKI	Cervix	Cervix Squamous Cell Carcinoma	0.73	0.61	0.47	0.42	0.10	0.08	0.08	0.09	0.16	0.14	0.10	0.03	0.25	0.07	0.07	0.01	0.37	0.54
HT-29	Colon	Colon Adenocarcinoma	0.04	0.09	0.06	0.07	0.05	0.05	0.02	0.05	0.03	0.06	0.07	0.02	0.02	0.01	0.01	0.01	0.04	0.25
HCT-15	Colon	Colon Adenocarcinoma	0.55	0.08	0.07	0.07	0.04	0.06	0.02	0.04	0.03	0.06	0.08	0.02	0.24	0.01	0.03	0.00	0.21	0.16
COLO-205	Colon	Colon Adenocarcinoma	0.01	0.07	0.07	0.09	0.06	0.07	0.02	0.05	0.05	0.08	0.08	0.01	0.01	0.03	0.02	0.00	0.02	0.10
KM12	Colon	Colon Adenocarcinoma	0.16	0.05	0.06	0.07	0.04	0.05	0.02	0.05	0.04	0.07	0.06	0.01	0.28	0.01	0.02	0.00	0.26	0.17
SW48	Colon	Colon Adenocarcinoma	0.06	0.05	0.06	0.07	0.04	0.05	0.01	0.05	0.04	0.07	0.11	0.00	0.21	0.02	0.02	0.00	0.20	0.32
CCK-81	Colon	Colon Adenocarcinoma	0.02	0.09	0.07	0.10	0.04	0.07	0.06	0.04	0.04	0.11	0.09	0.02	0.01	0.04	0.03	0.01	0.08	0.22
LS-180	Colon	Colon Adenocarcinoma	0.12	0.09	0.07	0.08	0.06	0.06	0.05	0.05	0.05	0.09	0.09	0.02	0.03	0.04	0.03	0.00	0.05	0.41
HCC-56	Colon	Colon Adenocarcinoma	0.01	0.09	0.07	0.09	0.06	0.06	0.03	0.05	0.06	0.09	0.07	0.02	0.03	0.04	0.03	0.01	0.04	0.30
MDST8	Colon	Colorectal Carcinoma	0.05	0.09	0.07	0.09	0.05	0.06	0.04	0.04	0.07	0.10	0.11	0.02	0.27	0.05	0.04	0.01	0.25	0.24
HT-115	Colon	Colorectal Carcinoma	0.46	0.09	0.06	0.09	0.06	0.04	0.04	0.04	0.04	0.09	0.09	0.01	0.03	0.04	0.02	0.01	0.02	0.14
CL-40	Colon	Colorectal Carcinoma	0.69	0.12	0.09	0.11	0.07	0.09	0.05	0.06	0.06	0.11	0.09	0.02	0.03	0.04	0.03	0.01	0.02	0.18
ISHIKAWA	Endometrium	Endometrial Adenocarcinoma	0.01	0.14	0.08	0.10	0.06	0.08	0.02	0.07	0.06	0.08	0.10	0.02	0.26	0.05	0.05	0.01	0.15	0.16
AN3-CA	Endometrium	Endometrial Adenocarcinoma	0.02	0.14	0.07	0.11	0.08	0.08	0.02	0.07	0.52	0.56	0.46	0.50	0.58	0.60	0.58	0.57	0.49	0.58
MFE-280	Endometrium	Endometrial Adenocarcinoma	0.07	0.11	0.09	0.15	0.09	0.10	0.05	0.10	0.10	0.13	0.19	0.04	0.31	0.06	0.04	0.01	0.29	0.32
COLO-684	Endometrium	Endometrial Adenocarcinoma	0.01	0.09	0.07	0.10	0.06	0.06	0.04	0.06	0.51	0.60	0.47	0.50	0.58	0.62	0.61	0.60	0.49	0.57
KLE	Endometrium	Endometrial Adenocarcinoma	0.01	0.09	0.07	0.09	0.07	0.07	0.02	0.06	0.06	0.10	0.11	0.04	0.30	0.05	0.03	0.02	0.27	0.25
MFE-319	Endometrium	Endometrial Adenocarcinoma	0.01	0.10	0.07	0.11	0.06	0.08	0.04	0.08	0.06	0.13	0.12	0.03	0.33	0.06	0.05	0.01	0.30	0.20
HEC-1	Endometrium	Endometrial Adenocarcinoma	0.02	0.10	0.08	0.11	0.07	0.08	0.04	0.05	0.05	0.10	0.10	0.02	0.30	0.05	0.03	0.01	0.26	0.25
MFE-296	Endometrium	Endometrial Adenocarcinoma	0.03	0.11	0.08	0.10	0.07	0.07	0.04	0.08	0.06	0.11	0.08	0.02	0.32	0.06	0.09	0.01	0.23	0.19
SNG-M	Endometrium	Endometrial Adenocarcinoma	0.01	0.13	0.08	0.12	0.09	0.10	0.05	0.07	0.06	0.11	0.10	0.02	0.17	0.05	0.05	0.01	0.14	0.22
EN	Endometrium	Endometrial Carcinoma	0.03	0.09	0.11	0.13	0.10	0.10	0.03	0.11	0.07	0.12	0.16	0.03	0.30	0.05	0.04	0.02	0.36	0.36
TOV-112D	Endometrium	Endometrial Carcinoma	0.11	0.12	0.08	0.10	0.07	0.10	0.05	0.07	0.87	0.50	0.37	0.21	0.46	0.61	0.25	0.25	0.40	0.35
ESS-1	Endometrium	Endometrial Carcinosarcoma-Malignant Mesodermal Mixed Tumour	0.10	0.11	0.08	0.11	0.07	0.07	0.02	0.05	0.57	0.60	0.40	0.24	0.42	0.24	0.13	0.11	0.37	0.50
RL95-2	Endometrium	Endometrial Mixed Adenosquamous Carcinoma	0.24	0.11	0.06	0.09	0.06	0.05	0.06	0.05	0.04	0.08	0.06	0.01	0.25	0.05	0.03	0.01	0.24	0.23
KYSE-150	Esophagus	Oesophagus Squamous Cell Carcinoma	0.07	0.07	0.09	0.08	0.08	0.08	0.04	0.09	0.07	0.11	0.10	0.03	0.30	0.04	0.02	0.01	0.50	0.79
HCE-7	Esophagus	Oesophagus Squamous Cell Carcinoma	0.01	0.13	0.10	0.10	0.08	0.08	0.03	0.08	0.06	0.08	0.09	0.02	0.22	0.06	0.07	0.00	0.35	0.42
ECC4	Gastrointestinal Tract (Site Indeterminate)	Gastrointestinal Tract Small Cell Carcinoma	0.01	0.08	0.07	0.09	0.06	0.05	0.01	0.04	0.05	0.09	0.09	0.02	0.03	0.04	0.02	0.01	0.05	0.22
RS4-11	Haematopoietic And Lymphoid Tissue	Acute Leukemia	0.14	0.12	0.07	0.10	0.06	0.07	0.04	0.06	0.05	0.14	0.10	0.02	0.26	0.06	0.08	0.05	0.42	0.36
MV-4-11	Haematopoietic And Lymphoid Tissue	Acute Leukemia (Ambiguous Lineage)	0.03	0.10	0.08	0.10	0.08	0.08	0.02	0.06	0.06	0.09	0.14	0.02	0.12	0.04	0.03	0.01	0.09	0.19
KY821	Haematopoietic And Lymphoid Tissue	Acute Leukemia (Ambiguous Lineage)	0.01	0.10	0.07	0.10	0.07	0.07	0.02	0.05	0.05	0.08	0.07	0.02	0.11	0.04	0.03	0.01	0.08	0.19
MY-M12	Haematopoietic And Lymphoid Tissue	Acute Leukemia (Biphenotic)	0.62	0.13	0.07	0.08	0.05	0.06	0.03	0.05	0.53	0.52	0.48	0.04	0.53	0.53	0.53	0.59	0.68	0.47
RPMI-8226	Haematopoietic And Lymphoid Tissue	Acute Lymphoblastic Leukemia	0.02	0.07	0.07	0.09	0.06	0.05	0.01	0.05	0.04	0.07	0.09	0.01	0.23	0.02	0.02	0.00	0.28	0.21
U-937	Haematopoietic And Lymphoid Tissue	Acute Lymphoblastic Leukemia	0.37	0.06	0.08	0.09	0.07	0.06	0.06	0.05	0.06	0.11	0.08	0.02	0.28	0.02	0.02	0.01	0.27	0.42
LP-1	Haematopoietic And Lymphoid Tissue	Acute Lymphoblastic Leukemia	0.00	0.07	0.09	0.11	0.09	0.09	0.02	0.07	0.06	0.10	0.09	0.02	0.30	0.05	0.03	0.00	0.24	0.25
RAJI	Haematopoietic And Lymphoid Tissue	Acute Lymphoblastic Leukemia	0.56	0.06	0.09	0.11	0.07	0.08	0.03	0.08	0.26	0.20	0.27	0.03	0.30	0.05	0.62	0.02	0.53	0.35
OPM-2	Haematopoietic And Lymphoid Tissue	Acute Lymphoblastic Leukemia	0.98	0.41	0.44	0.39	0.20	0.21	0.44	0.24	0.07	0.11	0.10	0.02	0.28	0.03	0.06	0.01	0.22	0.19
MINO	Haematopoietic And Lymphoid Tissue	Acute Lymphoblastic Leukemia	0.06	0.11	0.08	0.10	0.06	0.07	0.05	0.05	0.05	0.10	0.08	0.02	0.34	0.07	0.05	0.00	0.17	0.16
MOLT-16	Haematopoietic And Lymphoid Tissue	Acute Lymphoblastic Leukemia	0.01	0.06	0.07	0.10	0.08	0.07	0.03	0.06	0.50	0.54	0.47	0.47	0.57	0.48	0.43	0.70	0.74	0.77
HDLM-2	Haematopoietic And Lymphoid Tissue	Acute Lymphoblastic Leukemia	0.02	0.10	0.07	0.09	0.07	0.08	0.07	0.06	0.04	0.08	0.08	0.03	0.30	0.05	0.06	0.02	0.26	0.54
SUP-B15	Haematopoietic And Lymphoid Tissue	Acute Lymphoblastic Leukemia	0.22	0.38	0.11	0.11	0.05	0.07	0.03	0.18	0.27	0.24	0.19	0.03	0.35	0.30	0.33	0.33	0.41	0.52
KARPAS-45	Haematopoietic And Lymphoid Tissue	Acute Lymphoblastic Leukemia	0.83	0.41	0.38	0.29	0.12	0.15	0.05	0.06	0.89	0.84	0.84	0.96	0.94	0.95	0.97	1.00	0.97	0.95
697	Haematopoietic And Lymphoid Tissue	Acute Lymphoblastic Leukemia	0.07	0.10	0.07	0.10	0.06	0.07	0.05	0.04	0.54	0.60	0.50	0.84	0.61	0.63	0.62	0.62	0.95	0.93
GR-ST	Haematopoietic And Lymphoid Tissue	Acute Lymphoblastic Leukemia	0.38	0.10	0.08	0.10	0.06	0.07	0.07	0.04	0.42	0.59	0.41	0.13	0.58	0.64	0.63	0.57	0.46	0.60
EOL-1-CELL	Haematopoietic And Lymphoid Tissue	Acute Lymphoblastic Leukemia	0.32	0.10	0.08	0.11	0.07	0.06	0.05	0.05	0.05	0.10	0.09	0.02	0.26	0.04	0.05	0.01	0.50	0.51
ALL-PO	Haematopoietic And Lymphoid Tissue	Acute Lymphoblastic Leukemia	0.41	0.27	0.15	0.13	0.06	0.08	0.07	0.05	0.85	0.82	0.59	0.40	0.85	0.96	0.97	1.00	0.91	0.93
BE-13	Haematopoietic And Lymphoid Tissue	Acute Lymphoblastic Leukemia	0.04	0.10	0.07	0.11	0.06	0.06	0.06	0.04	0.89	0.85	0.83	0.88	0.96	0.95	0.98	1.00	0.96	0.92
KE-37	Haematopoietic And Lymphoid Tissue	Acute Lymphoblastic Leukemia	0.03	0.13	0.07	0.10	0.12	0.11	0.05	0.05	0.86	0.78	0.61	0.66	0.90	0.96	0.94	1.00	0.96	0.95
HAL-01	Haematopoietic And Lymphoid Tissue	Acute Lymphoblastic Leukemia	0.31	0.24	0.09	0.10	0.07	0.08	0.05	0.07	0.22	0.21	0.14	0.03	0.35	0.31	0.29	0.46	0.31	0.42
P30-OHK	Haematopoietic And Lymphoid Tissue	Acute Lymphoblastic Leukemia	0.01	0.12	0.09	0.11	0.09	0.10	0.05	0.08	0.07	0.12	0.10	0.04	0.32	0.05	0.12	0.01	0.28	0.31
LC4-1	Haematopoietic And Lymphoid Tissue	Acute Lymphoblastic Leukemia	0.65	0.25	0.11	0.12	0.06	0.06	0.03	0.06	0.05	0.10	0.10	0.01	0.24	0.03	0.04	0.00	0.25	0.22
CCRF-CEM	Haematopoietic And Lymphoid Tissue	Acute Lymphoblastic Leukemia	0.92	0.36	0.22	0.19	0.08	0.09	0.03	0.09	0.88	0.88	0.37	0.91	0.95	0.96	0.96	0.99	0.98	0.95
SUP-B8	Haematopoietic And Lymphoid Tissue	Acute Lymphoblastic Leukemia (B Cell)	0.07	0.10	0.08	0.08	0.06	0.08	0.05	0.06	0.06	0.11	0.10	0.02	0.28	0.03	0.05	0.01	0.50	0.61
MN-60	Haematopoietic And Lymphoid Tissue	Acute Lymphoblastic Leukemia (B Cell)	0.76	0.39	0.15	0.14	0.07	0.08	0.03	0.08	0.25	0.35	0.31	0.02	0.34	0.23	0.16	0.33	0.54	0.93
REH	Haematopoietic And Lymphoid Tissue	Acute Lymphoblastic Leukemia (B Cell)	0.03	0.10	0.09	0.10	0.07	0.07	0.03	0.08	0.05	0.12	0.13	0.02	0.26	0.05	0.04	0.02	0.31	0.50
U-698-M	Haematopoietic And Lymphoid Tissue	Acute Lymphoblastic Leukemia (B Cell)	0.39	0.11	0.06	0.10	0.06	0.06	0.04	0.05	0.56	0.65	0.54	0.14	0.60	0.64	0.64	0.63	0.80	0.96
BALL-1	Haematopoietic And Lymphoid Tissue	Acute Lymphoblastic Leukemia (B Cell)	0.23	0.11	0.08	0.09	0.07	0.06	0.04	0.04	0.08	0.09	0.08	0.02	0.31	0.06	0.06	0.06	0.41	0.56
NALM-6	Haematopoietic And Lymphoid Tissue	Acute Lymphoblastic Leukemia (B Cell)	0.35	0.13	0.07	0.11	0.06	0.09	0.07	0.07	0.22	0.21	0.18	0.05	0.55	0.56	0.64	0.67	0.91	0.93
MHH-PREB-1	Haematopoietic And Lymphoid Tissue	Acute Lymphoblastic Leukemia (B Cell)	0.90	0.34	0.08	0.08	0.05	0.07	0.04	0.04	0.90	0.85	0.86	0.20	0.95	0.96	0.99	1.00	0.98	0.96
MHH-CALL-2	Haematopoietic And Lymphoid Tissue	Acute Lymphoblastic Leukemia (B Cell)	0.08	0.12	0.29	0.15	0.07	0.07	0.03	0.06	0.06	0.09	0.07	0.01	0.24	0.04	0.02	0.01	0.16	0.15
MHH-CALL-4	Haematopoietic And Lymphoid Tissue	Acute Lymphoblastic Leukemia (B Cell)	0.47	0.20	0.13	0.15	0.08	0.10	0.05	0.09	0.09	0.16	0.12	0.02	0.34	0.07	0.10	0.02	0.53	0.41
MOLT-4	Haematopoietic And Lymphoid Tissue	Acute Lymphoblastic Leukemia (T Cell)	0.01	0.09	0.05	0.08	0.05	0.05	0.02	0.06	0.80	0.83	0.75	0.48	0.80	0.91	0.97	0.98	0.88	0.69
JURKAT	Haematopoietic And Lymphoid Tissue	Acute Lymphoblastic Leukemia (T Cell)	0.01	0.08	0.08	0.09	0.08	0.08	0.02	0.09	0.87	0.86	0.77	0.59	0.93	0.95	0.98	1.00	0.98	0.94
J-RT3-T3-5	Haematopoietic And Lymphoid Tissue	Acute Lymphoblastic Leukemia (T Cell)	0.09	0.09	0.07	0.09	0.07	0.07	0.05	0.05	0.85	0.84	0.79	0.61	0.90	0.95	0.97	0.99	0.95	0.91
SUP-T1	Haematopoietic And Lymphoid Tissue	Acute Lymphoblastic Leukemia (T Cell)	0.28	0.12	0.12	0.12	0.06	0.07	0.06	0.04	0.86	0.84	0.81	0.72	0.94	0.95	0.97	0.98	0.95	0.94
P12-ICHIKAWA	Haematopoietic And Lymphoid Tissue	Acute Lymphoblastic Leukemia (T Cell)	0.05	0.10	0.07	0.09	0.06	0.07	0.05	0.05	0.88	0.85	0.81	0.93	0.93	0.96	0.98	1.00	0.96	0.93
ATN-1	Haematopoietic And Lymphoid Tissue	Acute Lymphoblastic Leukemia (T Cell)	0.47	0.13	0.09	0.09	0.06	0.07	0.06	0.05	0.06	0.10	0.07	0.02	0.31	0.04	0.04	0.01	0.32	0.48
I-9-2	Haematopoietic And Lymphoid Tissue	Acute Lymphoblastic Leukemia (T Cell)	0.00	0.09	0.08	0.11	0.06	0.06	0.04	0.05	0.86	0.85	0.78	0.57	0.94	0.96	0.99	0.99	0.96	0.92
RPMI-8402	Haematopoietic And Lymphoid Tissue	Acute Lymphoblastic Leukemia (T Cell)	0.09	0.09	0.07	0.09	0.06	0.07	0.05	0.04	0.88	0.87	0.82	0.98	0.96	0.95	0.98	1.00	0.99	0.93
PF-382	Haematopoietic And Lymphoid Tissue	Acute Lymphoblastic Leukemia (T Cell)	0.38	0.13	0.09	0.12	0.07	0.09	0.03	0.07	0.82	0.83	0.81	0.65	0.95	0.96	0.99	0.99	0.96	0.91
LOUCY	Haematopoietic And Lymphoid Tissue	Acute Lymphoblastic Leukemia (T Cell)	0.00	0.07	0.04	0.09	0.04	0.04	0.04	0.03	0.87	0.87	0.81	0.97	0.95	0.97	0.99	1.00	0.99	0.95
MOLT-13	Haematopoietic And Lymphoid Tissue	Acute Lymphoblastic Leukemia (T Cell)	0.04	0.09	0.06	0.08	0.05	0.05	0.03	0.04	0.88	0.87	0.84	0.94	0.95	0.97	0.99	1.00	0.97	0.94
MONO-MAC-1	Haematopoietic And Lymphoid Tissue	Acute Monocytic Leukemia	0.02	0.09	0.09	0.12	0.06	0.08	0.04	0.05	0.04	0.09	0.11	0.01	0.03	0.04	0.03	0.01	0.02	0.13
HL-60	Haematopoietic And Lymphoid Tissue	Acute Myeloid Leukemia	0.00	0.11	0.09	0.13	0.08	0.07	0.04	0.06	0.28	0.16	0.16	0.05	0.28	0.09	0.08	0.04	0.27	0.31
HEL	Haematopoietic And Lymphoid Tissue	Acute Myeloid Leukemia	0.15	0.07	0.08	0.09	0.07	0.08	0.02	0.07	0.06	0.09	0.07	0.01	0.23	0.04	0.02	0.01	0.36	0.55
GDM-1	Haematopoietic And Lymphoid Tissue	Acute Myeloid Leukemia	0.05	0.10	0.08	0.09	0.06	0.07	0.03	0.06	0.05	0.07	0.08	0.02	0.30	0.05	0.07	0.00	0.37	0.49
MONO-MAC-6	Haematopoietic And Lymphoid Tissue	Acute Myeloid Leukemia	0.02	0.09	0.08	0.13	0.05	0.07	0.03	0.07	0.04	0.09	0.14	0.02	0.08	0.06	0.02	0.01	0.02	0.17
P31-FUJ	Haematopoietic And Lymphoid Tissue	Acute Myeloid Leukemia	0.04	0.08	0.07	0.12	0.07	0.06	0.04	0.07	0.52	0.57	0.50	0.10	0.57	0.60	0.61	0.57	0.48	0.57
THP-1	Haematopoietic And Lymphoid Tissue	Acute Myeloid Leukemia	0.10	0.10	0.08	0.10	0.05	0.07	0.02	0.04	0.06	0.08	0.10	0.01	0.02	0.02	0.02	0.01	0.07	0.16
OCI-AML3	Haematopoietic And Lymphoid Tissue	Acute Myeloid Leukemia	0.19	0.09	0.09	0.11	0.06	0.06	0.03	0.06	0.05	0.10	0.11	0.01	0.04	0.08	0.02	0.01	0.03	0.15
NKM-1	Haematopoietic And Lymphoid Tissue	Acute Myeloid Leukemia	0.38	0.11	0.09	0.11	0.07	0.07	0.05	0.06	0.88	0.86	0.50	0.57	0.94	0.96	0.97	0.98	0.96	0.94
NOMO-1	Haematopoietic And Lymphoid Tissue	Acute Myeloid Leukemia	0.02	0.09	0.07	0.12	0.06	0.07	0.04	0.05	0.05	0.08	0.08	0.01	0.04	0.04	0.02	0.00	0.03	0.15
KASUMI-1	Haematopoietic And Lymphoid Tissue	Acute Myeloid Leukemia	0.00	0.08	0.07	0.10	0.06	0.07	0.05	0.05	0.06	0.10	0.08	0.02	0.28	0.04	0.02	0.01	0.29	0.21
QIMR-WIL	Haematopoietic And Lymphoid Tissue	Acute Myeloid Leukemia	0.02	0.10	0.07	0.09	0.07	0.07	0.03	0.04	0.04	0.10	0.07	0.02	0.29	0.05	0.04	0.01	0.26	0.24
MOLM-13	Haematopoietic And Lymphoid Tissue	Acute Myeloid Leukemia	0.03	0.08	0.09	0.10	0.07	0.06	0.03	0.05	0.05	0.08	0.09	0.02	0.03	0.03	0.03	0.01	0.06	0.14
ME-1	Haematopoietic And Lymphoid Tissue	Acute Myeloid Leukemia	0.02	0.11	0.07	0.09	0.06	0.07	0.04	0.05	0.05	0.09	0.09	0.02	0.19	0.03	0.04	0.01	0.25	0.22
PL-21	Haematopoietic And Lymphoid Tissue	Acute Myeloid Leukemia	0.26	0.14	0.10	0.13	0.07	0.08	0.04	0.05	0.06	0.09	0.10	0.02	0.32	0.05	0.10	0.01	0.28	0.48
OCI-M1	Haematopoietic And Lymphoid Tissue	Acute Myeloid Leukemia	0.01	0.06	0.08	0.09	0.08	0.08	0.04	0.07	0.05	0.11	0.11	0.02	0.02	0.04	0.02	0.00	0.01	0.45
OCI-AML5	Haematopoietic And Lymphoid Tissue	Acute Myeloid Leukemia	0.20	0.11	0.11	0.11	0.07	0.07	0.02	0.06	0.08	0.09	0.10	0.02	0.32	0.06	0.03	0.02	0.59	0.89
K052	Haematopoietic And Lymphoid Tissue	Acute Myeloid Leukemia	0.00	0.06	0.06	0.10	0.07	0.07	0.02	0.06	0.05	0.10	0.06	0.01	0.30	0.02	0.02	0.00	0.24	0.18
OCI-AML2	Haematopoietic And Lymphoid Tissue	Acute Myeloid Leukemia	0.03	0.08	0.07	0.08	0.05	0.06	0.03	0.04	0.04	0.07	0.05	0.01	0.26	0.03	0.02	0.03	0.41	0.33
ML-2	Haematopoietic And Lymphoid Tissue	Acute Myeloid Leukemia	0.14	0.12	0.08	0.09	0.07	0.07	0.05	0.05	0.04	0.06	0.08	0.01	0.31	0.03	0.03	0.01	0.22	0.24
KG-1	Haematopoietic And Lymphoid Tissue	Acute Myeloid Leukemia	0.01	0.07	0.06	0.10	0.06	0.06	0.05	0.04	0.11	0.10	0.09	0.04	0.28	0.07	0.05	0.03	0.38	0.44
KMOE-2	Haematopoietic And Lymphoid Tissue	Acute Myeloid Leukemia	0.01	0.08	0.07	0.09	0.06	0.06	0.07	0.04	0.05	0.09	0.08	0.02	0.27	0.04	0.03	0.01	0.28	0.38
CESS	Haematopoietic And Lymphoid Tissue	Acute Myeloid Leukemia	0.00	0.13	0.08	0.10	0.08	0.08	0.03	0.07	0.05	0.10	0.11	0.03	0.12	0.04	0.03	0.01	0.14	0.26
SIG-M5	Haematopoietic And Lymphoid Tissue	Acute Myeloid Leukemia	0.05	0.15	0.09	0.09	0.08	0.09	0.06	0.08	0.15	0.42	0.11	0.03	0.32	0.07	0.13	0.03	0.31	0.54
KO52	Haematopoietic And Lymphoid Tissue	Acute Myeloid Leukemia	0.01	0.08	0.06	0.09	0.07	0.06	0.03	0.05	0.05	0.09	0.08	0.01	0.27	0.03	0.03	0.00	0.21	0.19
NB4	Haematopoietic And Lymphoid Tissue	Acute Myeloid Leukemia	0.29	0.07	0.08	0.14	0.08	0.08	0.05	0.06	0.08	0.14	0.12	0.01	0.29	0.04	0.05	0.01	0.52	0.61
HH	Haematopoietic And Lymphoid Tissue	Acute T Cell Lymphoma-Leukemia	0.03	0.14	0.10	0.12	0.08	0.10	0.08	0.08	0.50	0.59	0.47	0.16	0.56	0.64	0.75	0.83	0.56	0.92
C8166	Haematopoietic And Lymphoid Tissue	Adult T Cell Lymphoma-Leukemia	0.02	0.10	0.06	0.08	0.06	0.06	0.06	0.05	0.49	0.55	0.48	0.11	0.58	0.59	0.60	0.57	0.48	0.56
SU-DHL-1	Haematopoietic And Lymphoid Tissue	Anaplastic Large Cell Lymphoma	0.05	0.13	0.09	0.11	0.07	0.07	0.06	0.07	0.07	0.11	0.12	0.02	0.34	0.06	0.09	0.02	0.42	0.62
SUP-M2	Haematopoietic And Lymphoid Tissue	Anaplastic Large Cell Lymphoma	0.02	0.14	0.09	0.14	0.09	0.09	0.04	0.09	0.81	0.61	0.50	0.17	0.74	0.84	0.83	0.80	0.59	0.72
KARPAS-299	Haematopoietic And Lymphoid Tissue	Anaplastic Large Cell Lymphoma	0.05	0.12	0.08	0.10	0.07	0.08	0.07	0.06	0.05	0.11	0.08	0.01	0.30	0.05	0.05	0.01	0.29	0.25
TUR	Haematopoietic And Lymphoid Tissue	B Cell Lymphoma	0.68	0.11	0.08	0.10	0.07	0.08	0.04	0.04	0.05	0.11	0.12	0.02	0.30	0.04	0.04	0.01	0.32	0.43
CTB-1	Haematopoietic And Lymphoid Tissue	B Cell Lymphoma	0.41	0.08	0.07	0.08	0.06	0.06	0.05	0.05	0.89	0.85	0.86	0.40	0.94	0.95	0.95	0.99	0.96	0.94

		TargetID	cg21539223	cg16456337	cg15956794	cg14773728	cg17573603	cg17919331	cg23965551	cg00522636	cg13518195	cg26459500	cg01092811	cg06363041	cg25437259	cg14506646	cg14861803	cg11162249	cg17789800	cg00261665
		UCSC_REFGENE_NAME	DCP2								NUDT16									
		UCSC_REFGENE_GROUP	TSS1500	TSS1500	TSS200	TSS200	TSS200	TSS200	1stExon;5'UTR	1stExon	TSS1500	TSS1500	TSS1500	TSS200	TSS200	TSS200	TSS200	5'UTR;1stExon	Body	Body
**Cell Line**	**Tissue**	**Cell Line Type**																		
CRO-AP2	Haematopoietic And Lymphoid Tissue	B Cell Lymphoma	0.14	0.11	0.08	0.12	0.09	0.10	0.09	0.08	0.09	0.15	0.15	0.04	0.30	0.05	0.04	0.01	0.30	0.93
JEKO-1	Haematopoietic And Lymphoid Tissue	B Cell Lymphoma	0.47	0.21	0.12	0.13	0.07	0.08	0.03	0.08	0.07	0.12	0.10	0.02	0.33	0.05	0.12	0.01	0.26	0.51
SCC-3	Haematopoietic And Lymphoid Tissue	B Cell Lymphoma	0.26	0.11	0.08	0.12	0.07	0.08	0.08	0.07	0.07	0.13	0.12	0.02	0.32	0.07	0.07	0.01	0.32	0.23
RL	Haematopoietic And Lymphoid Tissue	B Cell Lymphoma	0.56	0.14	0.12	0.16	0.08	0.07	0.07	0.06	0.54	0.83	0.52	0.13	0.87	0.92	0.97	0.98	0.59	0.31
SU-DHL-4	Haematopoietic And Lymphoid Tissue	B Cell Lymphoma	0.41	0.08	0.08	0.10	0.06	0.06	0.04	0.04	0.48	0.52	0.44	0.14	0.39	0.35	0.47	0.39	0.48	0.50
SU-DHL-16	Haematopoietic And Lymphoid Tissue	B Cell Lymphoma	0.38	0.09	0.09	0.12	0.06	0.08	0.04	0.04	0.05	0.09	0.11	0.01	0.29	0.03	0.03	0.00	0.28	0.57
SU-DHL-8	Haematopoietic And Lymphoid Tissue	B Cell Lymphoma	0.97	0.14	0.17	0.12	0.07	0.08	0.07	0.05	0.50	0.54	0.48	0.14	0.57	0.59	0.59	0.59	0.66	0.54
RC-K8	Haematopoietic And Lymphoid Tissue	B Cell Lymphoma	0.03	0.08	0.09	0.10	0.08	0.09	0.03	0.07	0.56	0.58	0.50	0.43	0.60	0.58	0.61	0.59	0.51	0.60
SU-DHL-10	Haematopoietic And Lymphoid Tissue	B Cell Lymphoma	0.03	0.07	0.09	0.11	0.07	0.08	0.03	0.07	0.87	0.83	0.48	0.03	0.47	0.58	0.91	0.99	0.50	0.25
JSC-1	Haematopoietic And Lymphoid Tissue	B Cell Lymphoma	0.04	0.08	0.08	0.11	0.06	0.06	0.04	0.06	0.05	0.07	0.11	0.01	0.32	0.03	0.04	0.00	0.30	0.45
NU-DUL-1	Haematopoietic And Lymphoid Tissue	B Cell Lymphoma	0.10	0.12	0.08	0.10	0.06	0.09	0.04	0.06	0.89	0.87	0.85	0.79	0.95	0.96	0.99	0.99	0.98	0.95
TK	Haematopoietic And Lymphoid Tissue	B Cell Lymphoma	0.01	0.10	0.07	0.09	0.06	0.08	0.03	0.05	0.06	0.09	0.07	0.02	0.31	0.03	0.03	0.01	0.31	0.24
GRANTA-519	Haematopoietic And Lymphoid Tissue	B Cell Lymphoma	0.03	0.09	0.07	0.09	0.07	0.05	0.04	0.04	0.06	0.09	0.09	0.01	0.30	0.03	0.03	0.01	0.25	0.19
VAL	Haematopoietic And Lymphoid Tissue	B Cell Lymphoma	0.55	0.13	0.09	0.09	0.07	0.07	0.02	0.06	0.86	0.76	0.53	0.30	0.64	0.73	0.70	0.97	0.98	0.68
WSU-DLCL2	Haematopoietic And Lymphoid Tissue	B Cell Lymphoma	0.01	0.09	0.05	0.07	0.05	0.05	0.03	0.06	0.87	0.19	0.13	0.02	0.26	0.07	0.38	0.82	0.33	0.20
OCI-LY-19	Haematopoietic And Lymphoid Tissue	B Cell Lymphoma	0.96	0.11	0.08	0.09	0.04	0.04	0.04	0.05	0.51	0.56	0.26	0.03	0.33	0.25	0.31	0.32	0.44	0.65
MC116	Haematopoietic And Lymphoid Tissue	B Cell Lymphoma	0.06	0.09	0.07	0.08	0.06	0.07	0.06	0.04	0.25	0.34	0.18	0.04	0.28	0.15	0.15	0.12	0.39	0.34
BC-1	Haematopoietic And Lymphoid Tissue	B Cell Lymphoma	0.07	0.14	0.08	0.11	0.08	0.07	0.04	0.07	0.07	0.12	0.10	0.03	0.35	0.05	0.07	0.01	0.54	0.85
BC-3	Haematopoietic And Lymphoid Tissue	B Cell Lymphoma	0.52	0.17	0.10	0.14	0.08	0.08	0.05	0.08	0.05	0.09	0.12	0.04	0.06	0.07	0.04	0.01	0.05	0.17
SU-DHL-6	Haematopoietic And Lymphoid Tissue	B Cell Lymphoma	0.99	0.14	0.09	0.12	0.07	0.08	0.04	0.08	0.20	0.14	0.20	0.03	0.35	0.07	0.12	0.03	0.51	0.61
SU-DHL-5	Haematopoietic And Lymphoid Tissue	B Cell Lymphoma	0.99	0.23	0.11	0.11	0.07	0.08	0.04	0.08	0.40	0.47	0.37	0.14	0.54	0.61	0.48	0.58	0.60	0.63
HT	Haematopoietic And Lymphoid Tissue	B Cell Lymphoma	0.01	0.11	0.09	0.11	0.08	0.09	0.03	0.07	0.06	0.12	0.10	0.02	0.21	0.05	0.06	0.00	0.52	0.58
JM1	Haematopoietic And Lymphoid Tissue	B Cell Lymphoma	0.01	0.08	0.09	0.09	0.05	0.06	0.03	0.05	0.05	0.11	0.07	0.01	0.26	0.03	0.03	0.00	0.21	0.31
KOPN-8	Haematopoietic And Lymphoid Tissue	B Cell Precursor Leukemia	0.12	0.13	0.09	0.11	0.07	0.07	0.04	0.04	0.06	0.10	0.10	0.02	0.30	0.06	0.05	0.03	0.30	0.26
WIL2-NS	Haematopoietic And Lymphoid Tissue	B Lymphocyte	0.01	0.08	0.07	0.10	0.08	0.09	0.06	0.08	0.07	0.11	0.11	0.03	0.34	0.05	0.03	0.01	0.51	0.73
EM-2	Haematopoietic And Lymphoid Tissue	Blast Phase Chronic Myeloid Leukemia	0.01	0.14	0.08	0.11	0.08	0.08	0.07	0.08	0.07	0.12	0.12	0.03	0.31	0.08	0.09	0.01	0.35	0.31
MEG-01	Haematopoietic And Lymphoid Tissue	Blast Phase Chronic Myeloid Leukemia	0.01	0.09	0.07	0.10	0.06	0.06	0.02	0.05	0.04	0.10	0.07	0.01	0.26	0.04	0.03	0.01	0.31	0.24
LAMA-84	Haematopoietic And Lymphoid Tissue	Blast Phase Chronic Myeloid Leukemia	0.00	0.10	0.09	0.10	0.06	0.08	0.02	0.06	0.06	0.09	0.08	0.03	0.25	0.03	0.04	0.01	0.34	0.41
CML-T1	Haematopoietic And Lymphoid Tissue	Blast Phase Chronic Myeloid Leukemia	0.01	0.12	0.09	0.10	0.08	0.08	0.05	0.08	0.74	0.74	0.57	0.60	0.67	0.75	0.71	0.73	0.95	0.92
DG-75	Haematopoietic And Lymphoid Tissue	Burkitt'S Lymphoma	0.63	0.11	0.07	0.10	0.04	0.07	0.04	0.04	0.54	0.60	0.51	0.11	0.57	0.60	0.57	0.56	0.48	0.61
RAMOS-2G6-4C10	Haematopoietic And Lymphoid Tissue	Burkitt'S Lymphoma	0.38	0.09	0.07	0.08	0.06	0.07	0.06	0.04	0.07	0.15	0.09	0.02	0.31	0.04	0.04	0.01	0.08	0.25
P32-ISH	Haematopoietic And Lymphoid Tissue	Burkitt'S Lymphoma	0.08	0.12	0.09	0.09	0.07	0.08	0.04	0.06	0.16	0.17	0.10	0.02	0.13	0.07	0.06	0.04	0.25	0.34
ST486	Haematopoietic And Lymphoid Tissue	Burkitt'S Lymphoma	0.94	0.13	0.08	0.09	0.06	0.05	0.08	0.05	0.12	0.14	0.10	0.02	0.32	0.10	0.11	0.09	0.94	0.93
EB2	Haematopoietic And Lymphoid Tissue	Burkitt'S Lymphoma	0.03	0.14	0.09	0.12	0.06	0.08	0.06	0.07	0.06	0.10	0.10	0.02	0.32	0.06	0.07	0.01	0.33	0.28
DAUDI	Haematopoietic And Lymphoid Tissue	Burkitt'S Lymphoma	0.32	0.12	0.07	0.08	0.07	0.07	0.08	0.06	0.45	0.48	0.25	0.40	0.59	0.51	0.79	0.63	0.94	0.58
NAMALWA	Haematopoietic And Lymphoid Tissue	Burkitt'S Lymphoma	0.59	0.14	0.08	0.10	0.07	0.08	0.05	0.06	0.06	0.11	0.10	0.02	0.33	0.07	0.44	0.05	0.62	0.76
GA-10	Haematopoietic And Lymphoid Tissue	Burkitt'S Lymphoma	0.29	0.15	0.11	0.11	0.08	0.07	0.05	0.07	0.07	0.15	0.11	0.03	0.31	0.07	0.06	0.01	0.35	0.36
CA46	Haematopoietic And Lymphoid Tissue	Burkitt'S Lymphoma	0.62	0.14	0.08	0.11	0.07	0.06	0.06	0.07	0.88	0.83	0.75	0.39	0.93	0.94	0.96	0.99	0.93	0.91
EB-3	Haematopoietic And Lymphoid Tissue	Burkitt'S Lymphoma	0.03	0.14	0.09	0.11	0.08	0.08	0.06	0.09	0.08	0.12	0.14	0.03	0.33	0.07	0.08	0.02	0.49	0.59
JIYOYEP-2003	Haematopoietic And Lymphoid Tissue	Burkitt'S Lymphoma	0.72	0.13	0.07	0.10	0.07	0.08	0.06	0.07	0.05	0.11	0.11	0.02	0.33	0.04	0.08	0.01	0.44	0.73
BL-41	Haematopoietic And Lymphoid Tissue	Burkitt'S Lymphoma	0.76	0.14	0.09	0.15	0.09	0.10	0.05	0.08	0.58	0.67	0.40	0.34	0.61	0.62	0.65	0.66	0.97	0.93
GA-10-CLONE-4	Haematopoietic And Lymphoid Tissue	Burkitt'S Lymphoma	0.98	0.30	0.30	0.16	0.08	0.08	0.04	0.09	0.18	0.17	0.11	0.03	0.35	0.08	0.12	0.02	0.36	0.61
MEC-2	Haematopoietic And Lymphoid Tissue	Chronic Leukemia (B Cell)	0.01	0.09	0.07	0.11	0.06	0.06	0.02	0.05	0.03	0.08	0.09	0.03	0.18	0.05	0.03	0.00	0.07	0.19
MEC-1	Haematopoietic And Lymphoid Tissue	Chronic Leukemia (B Cell)	0.02	0.09	0.07	0.11	0.06	0.06	0.04	0.05	0.04	0.10	0.08	0.02	0.08	0.04	0.02	0.01	0.07	0.61
EHEB	Haematopoietic And Lymphoid Tissue	Chronic Lymphocytic Leukemia-Small Lymphocytic Lymphoma	0.00	0.11	0.07	0.10	0.07	0.07	0.00	0.04	0.05	0.09	0.09	0.02	0.04	0.03	0.02	0.00	0.02	0.16
JVM-2	Haematopoietic And Lymphoid Tissue	Chronic Lymphocytic Leukemia-Small Lymphocytic Lymphoma	0.01	0.11	0.07	0.10	0.06	0.07	0.03	0.05	0.05	0.09	0.10	0.02	0.31	0.05	0.03	0.01	0.05	0.17
JVM-3	Haematopoietic And Lymphoid Tissue	Chronic Lymphocytic Leukemia-Small Lymphocytic Lymphoma	0.02	0.12	0.07	0.09	0.06	0.08	0.04	0.05	0.05	0.11	0.08	0.02	0.29	0.03	0.02	0.00	0.15	0.53
RPMI-8866	Haematopoietic And Lymphoid Tissue	Chronic Myeloid Leukemia	0.02	0.08	0.07	0.11	0.07	0.06	0.04	0.09	0.07	0.09	0.13	0.03	0.30	0.03	0.03	0.00	0.36	0.96
KCL-22	Haematopoietic And Lymphoid Tissue	Chronic Myeloid Leukemia	0.00	0.08	0.07	0.10	0.06	0.06	0.05	0.05	0.05	0.07	0.08	0.02	0.26	0.04	0.03	0.01	0.39	0.41
JURL-MK1	Haematopoietic And Lymphoid Tissue	Chronic Myeloid Leukemia	0.01	0.09	0.07	0.10	0.07	0.07	0.02	0.05	0.05	0.08	0.09	0.02	0.02	0.05	0.03	0.01	0.01	0.11
KU812	Haematopoietic And Lymphoid Tissue	Chronic Myeloid Leukemia	0.00	0.08	0.06	0.09	0.05	0.05	0.04	0.03	0.05	0.08	0.07	0.02	0.21	0.03	0.06	0.00	0.27	0.22
K-562	Haematopoietic And Lymphoid Tissue	Chronic Myeloid Leukemia	0.00	0.13	0.07	0.09	0.08	0.08	0.04	0.08	0.06	0.12	0.09	0.03	0.30	0.04	0.10	0.01	0.15	0.18
DOHH-2	Haematopoietic And Lymphoid Tissue	Diffuse Large B Cell Lymphoma	0.06	0.12	0.07	0.11	0.06	0.07	0.05	0.05	0.52	0.66	0.50	0.21	0.65	0.69	0.69	0.68	0.57	0.60
KARPAS-422	Haematopoietic And Lymphoid Tissue	Diffuse Large B Cell Lymphoma	0.02	0.09	0.08	0.09	0.06	0.06	0.03	0.04	0.55	0.67	0.53	0.05	0.62	0.64	0.64	0.58	0.55	0.90
DB	Haematopoietic And Lymphoid Tissue	Diffuse Large B Cell Lymphoma	0.34	0.13	0.08	0.10	0.07	0.09	0.08	0.08	0.48	0.79	0.80	0.36	0.63	0.87	0.89	0.99	0.83	0.91
WSU-NHL	Haematopoietic And Lymphoid Tissue	Follicular Lymphoma	0.03	0.09	0.10	0.09	0.05	0.07	0.04	0.05	0.06	0.10	0.07	0.03	0.29	0.03	0.03	0.01	0.19	0.21
BV-173	Haematopoietic And Lymphoid Tissue	Haematopoietic Neoplasm	0.42	0.30	0.12	0.11	0.08	0.07	0.04	0.07	0.44	0.45	0.35	0.13	0.63	0.76	0.86	0.97	0.98	0.94
MO-T	Haematopoietic And Lymphoid Tissue	Hairy Cell Leukemia	0.02	0.11	0.07	0.09	0.06	0.07	0.03	0.05	0.09	0.10	0.08	0.02	0.27	0.04	0.05	0.02	0.31	0.54
BONNA-12	Haematopoietic And Lymphoid Tissue	Hairy Cell Leukemia	0.01	0.10	0.07	0.08	0.05	0.07	0.02	0.05	0.07	0.10	0.08	0.02	0.04	0.03	0.03	0.01	0.03	0.61
HC-1	Haematopoietic And Lymphoid Tissue	Hairy Cell Leukemia	0.00	0.12	0.08	0.10	0.08	0.08	0.02	0.07	0.06	0.08	0.09	0.01	0.08	0.03	0.03	0.00	0.16	0.18
MO	Haematopoietic And Lymphoid Tissue	Hairy Cell Leukemia	0.05	0.13	0.08	0.10	0.08	0.08	0.04	0.06	0.12	0.13	0.09	0.03	0.34	0.09	0.13	0.03	0.34	0.50
MLMA	Haematopoietic And Lymphoid Tissue	Hairy Cell Leukemia	0.80	0.12	0.09	0.09	0.08	0.08	0.04	0.06	0.07	0.07	0.09	0.02	0.28	0.04	0.10	0.00	0.29	0.23
HD-MY-Z	Haematopoietic And Lymphoid Tissue	Hodgkin'S Lymphoma	0.01	0.09	0.09	0.10	0.07	0.06	0.03	0.05	0.10	0.13	0.09	0.02	0.28	0.04	0.03	0.01	0.26	0.40
L-428	Haematopoietic And Lymphoid Tissue	Hodgkin'S Lymphoma	0.01	0.11	0.08	0.11	0.07	0.07	0.05	0.05	0.05	0.10	0.06	0.01	0.31	0.04	0.04	0.01	0.44	0.59
L-1236	Haematopoietic And Lymphoid Tissue	Hodgkin'S Lymphoma	0.02	0.10	0.08	0.10	0.07	0.07	0.06	0.05	0.09	0.10	0.11	0.02	0.33	0.06	0.04	0.01	0.48	0.63
L-540	Haematopoietic And Lymphoid Tissue	Hodgkin'S Lymphoma	0.46	0.09	0.07	0.10	0.06	0.06	0.03	0.04	0.46	0.31	0.26	0.05	0.56	0.55	0.55	0.54	0.62	0.93
SUP-HD1	Haematopoietic And Lymphoid Tissue	Hodgkin'S Lymphoma	0.00	0.14	0.08	0.11	0.07	0.08	0.04	0.07	0.05	0.08	0.10	0.02	0.33	0.06	0.07	0.01	0.52	0.94
RPMI-6666	Haematopoietic And Lymphoid Tissue	Hodgkin'S Lymphoma	0.01	0.11	0.09	0.10	0.07	0.08	0.03	0.08	0.07	0.12	0.11	0.03	0.12	0.06	0.05	0.02	0.03	0.19
HS-445	Haematopoietic And Lymphoid Tissue	Hodgkin'S Lymphoma	0.01	0.13	0.08	0.11	0.07	0.09	0.03	0.07	0.05	0.10	0.08	0.02	0.04	0.03	0.03	0.01	0.06	0.20
KM-H2	Haematopoietic And Lymphoid Tissue	Hodgkin'S Lymphoma	0.04	0.14	0.10	0.12	0.09	0.10	0.08	0.09	0.06	0.13	0.10	0.02	0.33	0.10	0.09	0.01	0.43	0.64
ROS-50	Haematopoietic And Lymphoid Tissue	Leukemia (B Cell)	0.29	0.10	0.11	0.09	0.06	0.07	0.05	0.05	0.61	0.78	0.53	0.35	0.59	0.68	0.68	0.69	0.96	0.90
KARPAS-231	Haematopoietic And Lymphoid Tissue	Leukemia (B Cell)	0.32	0.17	0.11	0.09	0.06	0.06	0.03	0.04	0.06	0.10	0.09	0.02	0.30	0.04	0.03	0.01	0.23	0.39
DND-41	Haematopoietic And Lymphoid Tissue	Leukemia (T Cell)	0.04	0.14	0.10	0.12	0.08	0.08	0.06	0.08	0.88	0.88	0.81	0.70	0.96	0.96	0.97	1.00	0.97	0.95
DEL	Haematopoietic And Lymphoid Tissue	Lymphoid Neoplasm	0.02	0.09	0.08	0.11	0.08	0.08	0.04	0.05	0.04	0.09	0.10	0.02	0.24	0.05	0.03	0.01	0.33	0.44
SR	Haematopoietic And Lymphoid Tissue	Lymphoid Neoplasm	0.69	0.11	0.08	0.11	0.06	0.06	0.05	0.05	0.43	0.46	0.30	0.05	0.52	0.57	0.56	0.55	0.56	0.88
KMS-12-BM	Haematopoietic And Lymphoid Tissue	Multiple Myeloma	0.03	0.08	0.08	0.07	0.05	0.06	0.02	0.04	0.06	0.08	0.07	0.02	0.30	0.03	0.03	0.00	0.22	0.42
H9	Haematopoietic And Lymphoid Tissue	Mycosis Fungoides-Sezary Syndrome	0.01	0.10	0.07	0.08	0.06	0.06	0.06	0.04	0.06	0.08	0.08	0.03	0.32	0.03	0.05	0.01	0.41	0.54
SKM-1	Haematopoietic And Lymphoid Tissue	Myelodysplastic Syndrome	0.04	0.09	0.07	0.10	0.05	0.07	0.05	0.05	0.06	0.11	0.07	0.02	0.22	0.05	0.04	0.01	0.36	0.40
MM1S	Haematopoietic And Lymphoid Tissue	Myeloma	0.00	0.14	0.09	0.13	0.07	0.10	0.04	0.08	0.06	0.12	0.10	0.02	0.30	0.05	0.06	0.00	0.40	0.41
YT	Haematopoietic And Lymphoid Tissue	Nk-T Cell Lymphoma	0.00	0.08	0.07	0.09	0.05	0.07	0.04	0.05	0.89	0.83	0.65	0.56	0.95	0.97	0.98	1.00	0.98	0.94
NK-92MI	Haematopoietic And Lymphoid Tissue	Non-Hodghin'S Lymphoma	0.00	0.06	0.08	0.09	0.07	0.07	0.04	0.06	0.30	0.53	0.53	0.12	0.57	0.57	0.56	0.54	0.47	0.64
FARAGE	Haematopoietic And Lymphoid Tissue	Non-Hodgkin'S Lymphoma (B Cell)	0.05	0.15	0.09	0.13	0.07	0.08	0.05	0.07	0.09	0.12	0.11	0.03	0.32	0.07	0.07	0.04	0.27	0.34
JJN-3	Haematopoietic And Lymphoid Tissue	Plasma Cell Leukemia	0.01	0.08	0.07	0.08	0.07	0.07	0.02	0.05	0.04	0.07	0.08	0.02	0.03	0.02	0.03	0.00	0.01	0.14
KARPAS-620	Haematopoietic And Lymphoid Tissue	Plasma Cell Leukemia	0.00	0.08	0.05	0.10	0.06	0.06	0.03	0.04	0.04	0.10	0.07	0.02	0.30	0.03	0.02	0.00	0.24	0.29
CTV-1	Haematopoietic And Lymphoid Tissue	Plasma Cell Myeloma	0.01	0.08	0.05	0.06	0.05	0.04	0.06	0.03	0.89	0.86	0.84	0.97	0.95	0.95	0.99	0.99	0.98	0.95
SK-MM-2	Haematopoietic And Lymphoid Tissue	Plasma Cell Myeloma	0.04	0.08	0.10	0.11	0.07	0.08	0.03	0.07	0.05	0.10	0.11	0.02	0.15	0.03	0.03	0.01	0.05	0.57
AMO-1	Haematopoietic And Lymphoid Tissue	Plasma Cell Myeloma	0.04	0.12	0.05	0.08	0.07	0.07	0.04	0.05	0.05	0.08	0.11	0.01	0.32	0.03	0.08	0.00	0.25	0.24
NCI-H929	Haematopoietic And Lymphoid Tissue	Plasma Cell Myeloma	0.01	0.10	0.07	0.10	0.07	0.07	0.05	0.07	0.05	0.11	0.07	0.03	0.33	0.04	0.06	0.01	0.07	0.19
L-363	Haematopoietic And Lymphoid Tissue	Plasma Cell Myeloma	0.03	0.09	0.08	0.10	0.07	0.07	0.02	0.05	0.09	0.09	0.08	0.02	0.20	0.03	0.02	0.01	0.34	0.56
U-266	Haematopoietic And Lymphoid Tissue	Plasma Cell Myeloma	0.01	0.08	0.08	0.11	0.05	0.06	0.05	0.04	0.04	0.08	0.08	0.02	0.22	0.02	0.02	0.01	0.09	0.17
ARH-77	Haematopoietic And Lymphoid Tissue	Plasma Cell Myeloma	0.28	0.12	0.08	0.10	0.09	0.09	0.04	0.07	0.06	0.10	0.10	0.02	0.07	0.05	0.04	0.01	0.09	0.47
IM-9	Haematopoietic And Lymphoid Tissue	Plasma Cell Myeloma	0.03	0.14	0.09	0.11	0.08	0.08	0.06	0.07	0.07	0.11	0.09	0.02	0.08	0.05	0.05	0.02	0.21	0.21
MC-CAR	Haematopoietic And Lymphoid Tissue	Plasma Cell Myeloma	0.06	0.14	0.09	0.11	0.08	0.08	0.07	0.07	0.44	0.36	0.27	0.39	0.52	0.50	0.50	0.51	0.35	0.41
SLVL	Haematopoietic And Lymphoid Tissue	Splenic B-Cell Lymphoma	0.04	0.07	0.09	0.10	0.08	0.07	0.04	0.06	0.09	0.10	0.10	0.03	0.31	0.03	0.03	0.01	0.26	0.43
SACC-83	Head & Neck	Adenoid Cystic Carcinoma	0.85	0.57	0.38	0.28	0.09	0.09	0.03	0.08	0.08	0.11	0.11	0.03	0.35	0.05	0.07	0.01	0.46	0.57
PCI-38	Head & Neck	Ns	0.00	0.08	0.07	0.11	0.07	0.08	0.07	0.07	0.06	0.11	0.07	0.03	0.24	0.03	0.03	0.01	0.26	0.35
BICR31	Head & Neck	Tongue Squamous Cell Carcinoma	0.03	0.13	0.08	0.13	0.08	0.09	0.04	0.08	0.06	0.12	0.10	0.02	0.05	0.05	0.04	0.00	0.05	0.32
DIFI	Intestine	Ns	0.00	0.13	0.07	0.10	0.07	0.08	0.04	0.08	0.06	0.13	0.10	0.04	0.04	0.05	0.05	0.01	0.03	0.19
NB(TU)1-10	Kidney	Adrenal Gland Neuroblastoma	0.01	0.12	0.08	0.09	0.08	0.09	0.03	0.07	0.07	0.09	0.08	0.03	0.30	0.04	0.11	0.01	0.28	0.29
RCC-FG2	Kidney	Carcinoma	0.02	0.13	0.07	0.10	0.07	0.05	0.04	0.07	0.07	0.09	0.09	0.02	0.16	0.06	0.06	0.01	0.04	0.61
RCC-AB	Kidney	Carcinoma	0.02	0.07	0.09	0.12	0.09	0.07	0.02	0.08	0.06	0.11	0.11	0.03	0.10	0.04	0.02	0.01	0.02	0.14
RCC-MF	Kidney	Carcinoma	0.03	0.08	0.08	0.09	0.07	0.08	0.03	0.07	0.06	0.09	0.11	0.02	0.10	0.04	0.03	0.01	0.09	0.19
RCC-ER	Kidney	Carcinoma	0.03	0.08	0.07	0.10	0.08	0.07	0.01	0.06	0.05	0.10	0.08	0.02	0.19	0.02	0.01	0.00	0.14	0.17
RCC-JW	Kidney	Carcinoma	0.01	0.14	0.08	0.10	0.08	0.08	0.03	0.09	0.05	0.11	0.10	0.03	0.03	0.04	0.04	0.01	0.04	0.19
KMRC-1	Kidney	Carcinoma	0.01	0.11	0.08	0.10	0.07	0.08	0.03	0.05	0.05	0.13	0.08	0.02	0.02	0.04	0.03	0.01	0.03	0.34
BFTC-909	Kidney	Carcinoma	0.31	0.11	0.08	0.10	0.06	0.06	0.03	0.05	0.04	0.10	0.08	0.02	0.22	0.05	0.03	0.01	0.21	0.24
SW156	Kidney	Hypernephroma	0.02	0.09	0.09	0.09	0.06	0.09	0.08	0.04	0.04	0.10	0.09	0.02	0.03	0.04	0.03	0.01	0.01	0.16

		TargetID	cg21539223	cg16456337	cg15956794	cg14773728	cg17573603	cg17919331	cg23965551	cg00522636	cg13518195	cg26459500	cg01092811	cg06363041	cg25437259	cg14506646	cg14861803	cg11162249	cg17789800	cg00261665
		UCSC_REFGENE_NAME	DCP2								NUDT16									
Cell Line	Tissue	Cell Line Type / UCSC_REFGENE_GROUP	TSS1500	TSS1500	TSS200	TSS200	TSS200	TSS200	1stExon;5'UTR	1stExon	TSS1500	TSS1500	TSS1500	TSS200	TSS200	TSS200	TSS200	5'UTR;1stExon	Body	Body
NCC021	Kidney	Ns	0.31	0.14	0.08	0.10	0.08	0.09	0.02	0.08	0.05	0.09	0.08	0.02	0.09	0.04	0.04	0.01	0.20	0.21
NCC010	Kidney	Ns	0.36	0.23	0.09	0.11	0.07	0.08	0.04	0.07	0.05	0.10	0.08	0.02	0.21	0.05	0.04	0.01	0.09	0.20
KMRC-20	Kidney	Ns	0.00	0.09	0.08	0.10	0.07	0.09	0.03	0.05	0.05	0.10	0.07	0.02	0.03	0.02	0.01	0.01	0.01	0.16
A498	Kidney	Renal Cell Carcinoma	0.04	0.08	0.06	0.09	0.06	0.06	0.01	0.05	0.05	0.07	0.07	0.01	0.03	0.02	0.02	0.00	0.03	0.12
ACHN	Kidney	Renal Cell Carcinoma	0.03	0.03	0.03	0.07	0.03	0.05	0.03	0.05	0.02	0.04	0.05	0.00	0.01	0.01	0.00	0.00	0.01	0.05
RCC10RGB	Kidney	Renal Cell Carcinoma	0.02	0.09	0.07	0.10	0.06	0.08	0.03	0.06	0.05	0.11	0.09	0.03	0.03	0.03	0.03	0.01	0.02	0.15
TK10	Kidney	Renal Cell Carcinoma	0.05	0.11	0.08	0.10	0.07	0.06	0.04	0.05	0.05	0.10	0.08	0.01	0.02	0.03	0.02	0.01	0.02	0.12
OS-RC-2	Kidney	Renal Cell Carcinoma	0.06	0.10	0.08	0.10	0.06	0.06	0.04	0.04	0.06	0.07	0.08	0.02	0.05	0.01	0.01	0.00	0.07	0.13
VMRC-RCZ	Kidney	Renal Cell Carcinoma	0.00	0.15	0.08	0.10	0.08	0.09	0.04	0.08	0.06	0.11	0.10	0.02	0.03	0.05	0.04	0.01	0.03	0.55
BB65-RCC	Kidney	Renal Cell Carcinoma	0.27	0.14	0.09	0.11	0.07	0.09	0.06	0.08	0.07	0.15	0.10	0.02	0.05	0.06	0.03	0.01	0.03	0.15
HA7-RCC	Kidney	Renal Cell Carcinoma	0.00	0.10	0.09	0.09	0.07	0.07	0.04	0.05	0.05	0.08	0.08	0.02	0.21	0.04	0.02	0.01	0.16	0.19
LB1047-RCC	Kidney	Renal Cell Carcinoma	0.00	0.09	0.08	0.09	0.06	0.06	0.04	0.06	0.04	0.10	0.08	0.02	0.08	0.04	0.03	0.01	0.09	0.19
LB2241-RCC	Kidney	Renal Cell Carcinoma	0.03	0.09	0.08	0.10	0.06	0.07	0.04	0.05	0.06	0.10	0.08	0.01	0.03	0.05	0.02	0.01	0.02	0.15
A704	Kidney	Renal Cell Carcinoma	0.03	0.11	0.08	0.11	0.06	0.08	0.03	0.05	0.04	0.09	0.08	0.02	0.15	0.05	0.12	0.01	0.26	0.29
CAL-54	Kidney	Renal Cell Carcinoma	0.03	0.08	0.06	0.10	0.06	0.07	0.05	0.04	0.05	0.09	0.09	0.01	0.02	0.04	0.02	0.01	0.07	0.16
LB996-RCC	Kidney	Renal Cell Carcinoma	0.03	0.10	0.06	0.09	0.05	0.07	0.02	0.05	0.04	0.08	0.07	0.02	0.03	0.06	0.03	0.01	0.02	0.15
SN12C	Kidney	Renal Cell Carcinoma	0.09	0.13	0.14	0.13	0.09	0.10	0.03	0.09	0.06	0.10	0.11	0.01	0.07	0.04	0.04	0.01	0.03	0.16
RXF393	Kidney	Renal Cell Carcinoma	0.01	0.13	0.09	0.12	0.07	0.10	0.04	0.08	0.06	0.15	0.10	0.02	0.05	0.05	0.03	0.01	0.04	0.16
U031	Kidney	Renal Cell Carcinoma	0.01	0.14	0.08	0.11	0.08	0.09	0.03	0.08	0.06	0.10	0.10	0.03	0.06	0.05	0.04	0.01	0.02	0.17
786-0	Kidney	Renal Clear Cell Carcinoma	0.00	0.08	0.07	0.10	0.06	0.05	0.02	0.06	0.04	0.06	0.08	0.02	0.08	0.02	0.00	0.00	0.25	0.60
769-P	Kidney	Renal Clear Cell Carcinoma	0.02	0.11	0.06	0.09	0.05	0.06	0.02	0.04	0.04	0.08	0.08	0.02	0.04	0.02	0.02	0.01	0.05	0.18
CAKI-1	Kidney	Renal Clear Cell Carcinoma	0.03	0.11	0.09	0.12	0.09	0.10	0.03	0.09	0.06	0.13	0.13	0.02	0.21	0.04	0.05	0.01	0.18	0.18
G-401	Kidney	Rhabdoid Tumour	0.01	0.09	0.08	0.10	0.06	0.06	0.02	0.05	0.05	0.08	0.07	0.02	0.26	0.04	0.03	0.00	0.27	0.45
SK-NEP-1	Kidney	Wilms Tumour	0.08	0.11	0.08	0.10	0.06	0.07	0.04	0.05	0.72	0.60	0.55	0.26	0.72	0.84	0.72	0.80	0.78	0.94
SNU-C5	Large Intestine	Adenocarcinoma	0.30	0.13	0.09	0.09	0.07	0.09	0.04	0.07	0.07	0.10	0.08	0.03	0.02	0.04	0.03	0.01	0.01	0.14
LS-513	Large Intestine	Caecum Adenocarcinoma	0.05	0.09	0.06	0.09	0.06	0.06	0.04	0.05	0.04	0.09	0.08	0.02	0.03	0.03	0.04	0.01	0.02	0.14
NCI-H747	Large Intestine	Caecum Adenocarcinoma	0.05	0.10	0.08	0.10	0.06	0.06	0.02	0.05	0.05	0.08	0.08	0.02	0.05	0.03	0.03	0.01	0.04	0.27
SNU-C2B	Large Intestine	Caecum Adenocarcinoma	0.01	0.10	0.06	0.10	0.07	0.06	0.06	0.05	0.05	0.08	0.10	0.02	0.11	0.04	0.03	0.01	0.30	0.22
NCI-H508	Large Intestine	Caecum Adenocarcinoma	0.00	0.05	0.07	0.09	0.05	0.07	0.03	0.05	0.04	0.08	0.08	0.02	0.01	0.03	0.01	0.00	0.01	0.14
SNU-283	Large Intestine	Carcinoma	0.01	0.13	0.08	0.10	0.07	0.08	0.07	0.06	0.07	0.11	0.09	0.03	0.04	0.03	0.03	0.01	0.02	0.17
SNU-1040	Large Intestine	Carcinoma	0.03	0.14	0.09	0.10	0.07	0.08	0.02	0.07	0.05	0.10	0.09	0.03	0.02	0.03	0.02	0.01	0.01	0.11
SNU-175	Large Intestine	Carcinoma	0.03	0.13	0.09	0.10	0.08	0.08	0.04	0.09	0.07	0.13	0.09	0.02	0.03	0.04	0.04	0.01	0.10	0.23
SNU-81	Large Intestine	Carcinoma	0.85	0.15	0.11	0.12	0.07	0.09	0.04	0.10	0.06	0.10	0.11	0.03	0.05	0.05	0.04	0.01	0.24	0.40
SNU-61	Large Intestine	Carcinoma	0.02	0.13	0.08	0.09	0.07	0.08	0.07	0.06	0.06	0.10	0.09	0.02	0.03	0.05	0.04	0.01	0.02	0.18
SNU-407	Large Intestine	Carcinoma	0.05	0.12	0.10	0.12	0.09	0.09	0.05	0.09	0.07	0.13	0.12	0.03	0.05	0.06	0.04	0.01	0.10	0.20
SW-480	Large Intestine	Colon Adenocarcinoma	0.00	0.14	0.08	0.10	0.07	0.07	0.03	0.06	0.05	0.10	0.11	0.02	0.05	0.04	0.04	0.01	0.08	0.17
HCT-116	Large Intestine	Colon Adenocarcinoma	0.55	0.05	0.04	0.06	0.05	0.03	0.00	0.05	0.03	0.05	0.06	0.00	0.02	0.03	0.01	0.01	0.29	0.53
RKO	Large Intestine	Colon Adenocarcinoma	0.92	0.18	0.12	0.12	0.07	0.07	0.03	0.06	0.07	0.11	0.08	0.02	0.03	0.03	0.04	0.01	0.02	0.09
SK-CO-1	Large Intestine	Colon Adenocarcinoma	0.10	0.09	0.07	0.08	0.05	0.05	0.04	0.04	0.05	0.08	0.07	0.02	0.01	0.03	0.02	0.00	0.01	0.13
LOVO	Large Intestine	Colon Adenocarcinoma	0.03	0.07	0.09	0.09	0.08	0.07	0.04	0.08	0.05	0.11	0.08	0.02	0.25	0.03	0.03	0.02	0.22	0.22
COLO-320	Large Intestine	Colon Adenocarcinoma	0.00	0.08	0.09	0.09	0.07	0.08	0.02	0.06	0.12	0.09	0.09	0.02	0.28	0.06	0.04	0.01	0.19	0.28
LS-411N	Large Intestine	Colon Adenocarcinoma	0.03	0.08	0.08	0.09	0.07	0.07	0.02	0.07	0.05	0.08	0.08	0.01	0.03	0.03	0.01	0.01	0.20	0.16
SW837	Large Intestine	Colon Adenocarcinoma	0.01	0.09	0.08	0.10	0.07	0.07	0.01	0.07	0.05	0.09	0.07	0.02	0.02	0.04	0.02	0.00	0.00	0.13
SW1417	Large Intestine	Colon Adenocarcinoma	0.01	0.10	0.07	0.08	0.05	0.06	0.03	0.06	0.04	0.07	0.07	0.02	0.02	0.02	0.03	0.00	0.01	0.12
LS-1034	Large Intestine	Colon Adenocarcinoma	0.39	0.12	0.07	0.10	0.07	0.06	0.02	0.07	0.04	0.07	0.07	0.01	0.02	0.03	0.03	0.02	0.01	0.24
GP5D	Large Intestine	Colon Adenocarcinoma	0.02	0.08	0.06	0.09	0.06	0.05	0.05	0.04	0.05	0.09	0.10	0.02	0.02	0.05	0.02	0.01	0.02	0.17
C2BBE1	Large Intestine	Colon Adenocarcinoma	0.00	0.09	0.07	0.09	0.06	0.06	0.05	0.06	0.06	0.08	0.08	0.02	0.14	0.04	0.03	0.01	0.09	0.15
LS-123	Large Intestine	Colon Adenocarcinoma	0.10	0.09	0.07	0.08	0.06	0.06	0.05	0.06	0.05	0.10	0.08	0.02	0.04	0.02	0.04	0.01	0.03	0.16
COLO-320-HSR	Large Intestine	Colon Adenocarcinoma	0.01	0.08	0.07	0.09	0.06	0.07	0.03	0.05	0.08	0.09	0.09	0.02	0.32	0.07	0.04	0.01	0.15	0.31
SW948	Large Intestine	Colon Adenocarcinoma	0.34	0.08	0.07	0.09	0.06	0.07	0.04	0.05	0.07	0.11	0.12	0.03	0.05	0.03	0.03	0.01	0.06	0.18
NCI-H716	Large Intestine	Colon Adenocarcinoma	0.04	0.11	0.09	0.10	0.07	0.07	0.03	0.05	0.05	0.11	0.08	0.03	0.13	0.04	0.03	0.01	0.05	0.16
SNU-C1	Large Intestine	Colon Adenocarcinoma	0.02	0.08	0.06	0.07	0.06	0.05	0.05	0.05	0.06	0.07	0.09	0.02	0.08	0.03	0.02	0.00	0.05	0.26
SW1116	Large Intestine	Colon Adenocarcinoma	0.01	0.10	0.06	0.09	0.05	0.06	0.03	0.05	0.04	0.09	0.07	0.01	0.02	0.03	0.02	0.00	0.03	0.13
HT55	Large Intestine	Colon Adenocarcinoma	0.02	0.09	0.06	0.10	0.06	0.06	0.05	0.04	0.05	0.10	0.09	0.02	0.02	0.04	0.02	0.01	0.02	0.50
HCC2998	Large Intestine	Colon Adenocarcinoma	0.00	0.10	0.09	0.09	0.07	0.08	0.04	0.07	0.03	0.09	0.09	0.02	0.03	0.03	0.03	0.01	0.01	0.14
CW-2	Large Intestine	Colorectal Carcinoma	0.17	0.08	0.07	0.08	0.05	0.06	0.04	0.04	0.04	0.08	0.08	0.01	0.04	0.04	0.03	0.01	0.02	0.09
CL-11	Large Intestine	Colorectal Carcinoma	0.01	0.09	0.07	0.10	0.06	0.06	0.03	0.05	0.05	0.10	0.09	0.02	0.02	0.04	0.03	0.01	0.03	0.12
T84	Large Intestine	Colorectal Carcinoma	0.15	0.13	0.09	0.12	0.07	0.07	0.05	0.07	0.06	0.11	0.10	0.02	0.04	0.06	0.04	0.00	0.03	0.25
COLO-741	Large Intestine	Colorectal Carcinoma	0.02	0.11	0.09	0.12	0.07	0.08	0.05	0.08	0.06	0.12	0.12	0.03	0.30	0.06	0.09	0.01	0.26	0.23
COLO-678	Large Intestine	Colorectal Carcinoma	0.00	0.13	0.09	0.12	0.07	0.08	0.04	0.08	0.06	0.12	0.11	0.03	0.05	0.06	0.04	0.01	0.08	0.17
NCI-H630	Large Intestine	Rectum Adenocarcinoma	0.01	0.08	0.06	0.10	0.07	0.06	0.02	0.05	0.04	0.07	0.08	0.02	0.04	0.04	0.03	0.01	0.01	0.13
RCM-1	Large Intestine	Rectum Adenocarcinoma	0.02	0.10	0.06	0.10	0.06	0.07	0.07	0.04	0.04	0.08	0.08	0.02	0.02	0.03	0.02	0.01	0.01	0.13
SW1463	Large Intestine	Rectum Adenocarcinoma	0.00	0.07	0.04	0.08	0.05	0.06	0.05	0.04	0.05	0.11	0.08	0.01	0.02	0.03	0.02	0.01	0.01	0.13
CAR-1	Large Intestine	Rectum Adenocarcinoma	0.25	0.14	0.11	0.12	0.09	0.09	0.05	0.08	0.07	0.09	0.12	0.02	0.04	0.03	0.03	0.01	0.03	0.18
HEP_G2	Liver	Hepatocellular Carcinoma	0.01	0.13	0.08	0.10	0.06	0.07	0.05	0.06	0.04	0.09	0.08	0.02	0.29	0.03	0.04	0.00	0.20	0.20
SNU-387	Liver	Hepatocellular Carcinoma	0.01	0.11	0.07	0.09	0.06	0.06	0.03	0.05	0.05	0.07	0.09	0.02	0.03	0.04	0.03	0.00	0.04	0.18
SNU-423	Liver	Hepatocellular Carcinoma	0.01	0.09	0.06	0.10	0.06	0.06	0.02	0.06	0.05	0.09	0.08	0.02	0.24	0.04	0.05	0.01	0.24	0.26
SNU-398	Liver	Hepatocellular Carcinoma	0.02	0.11	0.07	0.10	0.06	0.06	0.02	0.06	0.05	0.08	0.07	0.02	0.28	0.03	0.06	0.00	0.21	0.22
HLF	Liver	Hepatocellular Carcinoma	0.01	0.10	0.07	0.08	0.06	0.06	0.02	0.06	0.05	0.05	0.07	0.01	0.29	0.04	0.04	0.01	0.09	0.15
SNU-182	Liver	Hepatocellular Carcinoma	0.02	0.07	0.09	0.10	0.08	0.07	0.02	0.07	0.06	0.09	0.09	0.03	0.28	0.05	0.05	0.02	0.23	0.21
JHH-4	Liver	Hepatocellular Carcinoma	0.02	0.11	0.08	0.10	0.06	0.07	0.04	0.06	0.06	0.10	0.11	0.03	0.08	0.04	0.03	0.01	0.04	0.17
JHH-2	Liver	Hepatocellular Carcinoma	0.06	0.12	0.08	0.10	0.05	0.08	0.06	0.05	0.05	0.12	0.09	0.03	0.03	0.04	0.03	0.01	0.03	0.13
C3A	Liver	Hepatocellular Carcinoma	0.03	0.08	0.08	0.10	0.07	0.07	0.04	0.07	0.05	0.13	0.10	0.02	0.30	0.05	0.04	0.01	0.24	0.20
JHH-1	Liver	Hepatocellular Carcinoma	0.00	0.08	0.07	0.11	0.06	0.05	0.04	0.06	0.06	0.10	0.10	0.03	0.30	0.04	0.03	0.01	0.39	0.44
HUH-7	Liver	Hepatocellular Carcinoma	0.03	0.09	0.07	0.10	0.06	0.07	0.04	0.06	0.05	0.09	0.11	0.03	0.26	0.05	0.03	0.01	0.17	0.34
HLE	Liver	Hepatocellular Carcinoma	0.01	0.08	0.08	0.10	0.07	0.07	0.02	0.06	0.05	0.11	0.09	0.02	0.31	0.04	0.03	0.01	0.24	0.22
JHH-7	Liver	Hepatocellular Carcinoma	0.02	0.07	0.07	0.11	0.05	0.08	0.04	0.05	0.06	0.13	0.09	0.02	0.30	0.06	0.05	0.01	0.34	0.40
SNU-475	Liver	Hepatocellular Carcinoma	0.17	0.17	0.10	0.12	0.07	0.09	0.04	0.08	0.06	0.11	0.11	0.03	0.15	0.03	0.06	0.01	0.19	0.30
SNU-449	Liver	Hepatocellular Carcinoma	0.01	0.12	0.09	0.10	0.08	0.09	0.04	0.08	0.08	0.11	0.12	0.03	0.34	0.06	0.06	0.01	0.13	0.22
HEP3B2-1-7	Liver	Hepatocellular Carcinoma	0.00	0.12	0.08	0.09	0.07	0.08	0.03	0.08	0.05	0.12	0.09	0.03	0.26	0.04	0.07	0.00	0.33	0.41
HUH-1	Liver	Hepatoma	0.00	0.09	0.08	0.10	0.07	0.07	0.01	0.05	0.04	0.08	0.09	0.01	0.02	0.03	0.02	0.01	0.05	0.09
SK-HEP-1	Liver	Liver Adenocarcinoma	0.03	0.08	0.08	0.11	0.07	0.07	0.04	0.10	0.04	0.11	0.10	0.04	0.04	0.04	0.03	0.01	0.03	0.16
JHH-6	Liver	Undifferentiated Hepatocellular Carcinoma	0.02	0.09	0.07	0.09	0.06	0.07	0.04	0.05	0.04	0.12	0.10	0.02	0.31	0.05	0.03	0.01	0.07	0.17
201T	Lung	Adenocarcinoma	0.00	0.10	0.04	0.08	0.03	0.04	0.04	0.02	0.03	0.07	0.06	0.01	0.05	0.02	0.03	0.01	0.18	0.19
NCI-H1573	Lung	Lung Adenocarcinoma	0.00	0.08	0.08	0.10	0.07	0.06	0.02	0.07	0.06	0.09	0.09	0.02	0.14	0.03	0.02	0.01	0.19	0.19
NCI-H441	Lung	Lung Adenocarcinoma	0.15	0.12	0.07	0.10	0.05	0.06	0.02	0.05	0.03	0.06	0.06	0.01	0.05	0.04	0.01	0.01	0.07	0.30
RERF-LC-MS	Lung	Lung Adenocarcinoma	0.01	0.06	0.08	0.09	0.07	0.07	0.03	0.06	0.05	0.09	0.07	0.02	0.15	0.03	0.03	0.01	0.20	0.23
NCI-H3122	Lung	Lung Adenocarcinoma	0.00	0.07	0.07	0.09	0.07	0.07	0.03	0.06	0.05	0.10	0.09	0.03	0.04	0.05	0.03	0.02	0.04	0.22
COR-L105	Lung	Lung Adenocarcinoma	0.00	0.06	0.07	0.09	0.06	0.06	0.05	0.06	0.06	0.11	0.09	0.02	0.24	0.04	0.04	0.00	0.05	0.20
NCI-H2030	Lung	Lung Adenocarcinoma	0.09	0.09	0.09	0.09	0.07	0.07	0.03	0.06	0.06	0.10	0.08	0.03	0.20	0.04	0.03	0.01	0.24	0.18
EKVX	Lung	Lung Adenocarcinoma	0.03	0.08	0.07	0.11	0.08	0.07	0.03	0.06	0.08	0.10	0.08	0.02	0.10	0.05	0.03	0.01	0.14	0.18
NCI-H2122	Lung	Lung Adenocarcinoma	0.02	0.11	0.07	0.10	0.07	0.08	0.03	0.05	0.05	0.12	0.08	0.02	0.02	0.03	0.02	0.00	0.01	0.12
NCI-H1651	Lung	Lung Adenocarcinoma	0.03	0.08	0.07	0.10	0.06	0.06	0.03	0.06	0.05	0.08	0.08	0.02	0.12	0.04	0.02	0.01	0.10	0.18
LXF-289	Lung	Lung Adenocarcinoma	0.03	0.10	0.10	0.11	0.07	0.08	0.02	0.06	0.06	0.10	0.08	0.02	0.05	0.04	0.04	0.01	0.02	0.14
NCI-H1781	Lung	Lung Adenocarcinoma	0.01	0.10	0.08	0.10	0.07	0.07	0.05	0.06	0.06	0.09	0.07	0.02	0.26	0.04	0.03	0.01	0.32	0.23
LC-2-AD	Lung	Lung Adenocarcinoma	0.03	0.09	0.08	0.10	0.07	0.09	0.02	0.07	0.05	0.09	0.09	0.02	0.03	0.05	0.02	0.00	0.01	0.17
ABC-1	Lung	Lung Adenocarcinoma	0.00	0.13	0.08	0.10	0.07	0.07	0.06	0.08	0.08	0.10	0.09	0.03	0.31	0.05	0.10	0.01	0.42	0.67
NCI-H1563	Lung	Lung Adenocarcinoma	0.13	0.15	0.06	0.10	0.08	0.07	0.04	0.07	0.06	0.11	0.10	0.03	0.34	0.06	0.10	0.01	0.32	0.26
RERF-LC-KJ	Lung	Lung Adenocarcinoma	0.01	0.09	0.08	0.10	0.07	0.06	0.04	0.04	0.05	0.09	0.09	0.02	0.02	0.03	0.02	0.01	0.04	0.12
SK-LU-1	Lung	Lung Adenocarcinoma	0.00	0.10	0.07	0.09	0.05	0.05	0.03	0.03	0.06	0.09	0.08	0.02	0.20	0.05	0.02	0.01	0.20	0.24
NCI-H1793	Lung	Lung Adenocarcinoma	0.01	0.10	0.08	0.08	0.07	0.08	0.03	0.05	0.05	0.07	0.08	0.02	0.02	0.02	0.02	0.01	0.04	0.16
H3255	Lung	Lung Adenocarcinoma	0.00	0.10	0.06	0.07	0.05	0.05	0.03	0.03	0.05	0.07	0.08	0.05	0.23	0.22	0.21	0.18	0.25	0.23
NCI-H1734	Lung	Lung Adenocarcinoma	0.00	0.07	0.07	0.07	0.06	0.06	0.02	0.05	0.05	0.09	0.07	0.00	0.05	0.03	0.02	0.00	0.06	0.21
NCI-H1993	Lung	Lung Adenocarcinoma	0.01	0.09	0.07	0.09	0.06	0.06	0.02	0.04	0.04	0.11	0.06	0.02	0.02	0.03	0.01	0.01	0.05	0.15
NCI-H1755	Lung	Lung Adenocarcinoma	0.01	0.10	0.08	0.12	0.05	0.08	0.02	0.05	0.04	0.08	0.07	0.02	0.03	0.03	0.01	0.01	0.01	0.13
PC-3_JPC-3	Lung	Lung Adenocarcinoma	0.02	0.07	0.07	0.08	0.06	0.04	0.03	0.04	0.04	0.06	0.06	0.01	0.01	0.02	0.02	0.01	0.02	0.13
VMRC-LCD	Lung	Lung Adenocarcinoma	0.01	0.07	0.07	0.11	0.08	0.07	0.03	0.05	0.04	0.11	0.08	0.02	0.02	0.03	0.02	0.01	0.01	0.24

Cell Line	Tissue	Cell Line Type	cg21539223	cg16456337	cg15956794	cg14773728	cg17573603	cg17919331	cg23965551	cg00522636	cg13518195	cg26459500	cg01092811	cg06363041	cg25437259	cg14506646	cg14861803	cg11162249	cg17789800	cg00261665
		UCSC_REFGENE_NAME	DCP2								NUDT16									
		UCSC_REFGENE_GROUP	TSS1500	TSS1500	TSS200	TSS200	TSS200	TSS200	1stExon;5'UTR	1stExon	TSS1500	TSS1500	TSS1500	TSS200	TSS200	TSS200	TSS200	5'UTR;1stExon	Body	Body
NCI-H1666	Lung	Lung Bronchioloalveolar Adenocarcinoma	0.01	0.08	0.09	0.11	0.08	0.06	0.02	0.06	0.05	0.11	0.07	0.02	0.09	0.05	0.03	0.01	0.16	0.18
NCI-H358	Lung	Lung Bronchioloalveolar Adenocarcinoma	0.00	0.09	0.09	0.10	0.07	0.07	0.03	0.07	0.05	0.09	0.08	0.01	0.03	0.03	0.02	0.01	0.00	0.13
NCI-H322M	Lung	Lung Bronchioloalveolar Adenocarcinoma	0.02	0.10	0.08	0.11	0.07	0.08	0.04	0.06	0.07	0.11	0.08	0.04	0.27	0.04	0.04	0.01	0.25	0.25
UMC-11	Lung	Lung Carcinoid-Endocrine Tumour	0.01	0.07	0.06	0.10	0.06	0.06	0.02	0.06	0.06	0.10	0.09	0.01	0.02	0.01	0.02	0.00	0.00	0.17
NCI-H835	Lung	Lung Carcinoid-Endocrine Tumour	0.08	0.09	0.09	0.10	0.06	0.08	0.05	0.06	0.07	0.08	0.09	0.02	0.26	0.02	0.02	0.01	0.13	0.30
LB647-SCLC	Lung	Lung Carcinoma	0.02	0.09	0.08	0.09	0.07	0.06	0.02	0.06	0.15	0.13	0.09	0.03	0.32	0.05	0.07	0.02	0.27	0.31
CHAGO-K-1	Lung	Lung Carcinoma	0.41	0.10	0.09	0.11	0.06	0.06	0.03	0.05	0.04	0.09	0.07	0.03	0.29	0.03	0.02	0.01	0.29	0.27
NCI-H727	Lung	Lung Carcinoma	0.01	0.09	0.07	0.11	0.08	0.06	0.03	0.06	0.03	0.11	0.08	0.03	0.03	0.02	0.02	0.01	0.06	0.12
BEN	Lung	Lung Carcinoma	0.02	0.10	0.08	0.08	0.07	0.07	0.02	0.06	0.05	0.09	0.07	0.02	0.02	0.04	0.01	0.01	0.01	0.12
NCI-H720	Lung	Lung Carcinoma	0.01	0.14	0.07	0.10	0.09	0.08	0.05	0.09	0.07	0.11	0.12	0.02	0.34	0.05	0.07	0.01	0.37	0.41
A427	Lung	Lung Carcinoma	0.34	0.22	0.13	0.13	0.07	0.06	0.03	0.05	0.06	0.07	0.08	0.01	0.23	0.03	0.02	0.00	0.28	0.66
COR-L321	Lung	Lung Carcinoma	0.03	0.13	0.08	0.08	0.08	0.07	0.05	0.07	0.06	0.13	0.08	0.04	0.25	0.05	0.09	0.01	0.19	0.26
LU-99A	Lung	Lung Giant Cell Carcinoma	0.35	0.12	0.08	0.12	0.08	0.07	0.02	0.08	0.06	0.07	0.08	0.02	0.04	0.05	0.02	0.01	0.02	0.15
NCI-H810	Lung	Lung Large Cell Carcinoma	0.00	0.08	0.07	0.10	0.06	0.08	0.04	0.05	0.06	0.08	0.07	0.02	0.04	0.05	0.09	0.01	0.02	0.19
HOP-92	Lung	Lung Large Cell Carcinoma	0.01	0.07	0.06	0.09	0.07	0.05	0.02	0.06	0.05	0.08	0.08	0.01	0.02	0.05	0.02	0.00	0.01	0.13
NCI-H1581	Lung	Lung Large Cell Carcinoma	0.00	0.08	0.07	0.09	0.06	0.08	0.04	0.05	0.05	0.09	0.08	0.03	0.31	0.04	0.05	0.03	0.30	0.24
LCLC-97TM1	Lung	Lung Large Cell Carcinoma	0.09	0.09	0.07	0.08	0.06	0.05	0.01	0.04	0.03	0.06	0.05	0.01	0.02	0.03	0.02	0.00	0.00	0.16
IA-LM	Lung	Lung Large Cell Carcinoma	0.06	0.08	0.07	0.10	0.07	0.07	0.03	0.05	0.18	0.18	0.13	0.04	0.30	0.19	0.03	0.07	0.39	0.66
NCI-H650	Lung	Lung Large Cell Carcinoma	0.35	0.27	0.39	0.23	0.05	0.07	0.03	0.05	0.05	0.08	0.10	0.02	0.02	0.04	0.02	0.00	0.01	0.13
NCI-H596	Lung	Lung Mixed Adenosquamous Carcinoma	0.01	0.08	0.07	0.11	0.07	0.07	0.04	0.05	0.06	0.09	0.07	0.01	0.14	0.03	0.02	0.01	0.15	0.18
NCI-H292	Lung	Lung Mucoepidermoid Carcinoma	0.04	0.08	0.08	0.10	0.06	0.06	0.03	0.06	0.06	0.09	0.09	0.03	0.04	0.04	0.03	0.01	0.03	0.11
NCI-H23	Lung	Lung Non Small Cell Carcinoma	0.01	0.08	0.07	0.10	0.06	0.07	0.02	0.05	0.04	0.06	0.09	0.01	0.12	0.02	0.02	0.01	0.24	0.17
A549	Lung	Lung Non Small Cell Carcinoma	0.00	0.10	0.07	0.10	0.06	0.05	0.01	0.06	0.04	0.09	0.07	0.02	0.24	0.02	0.02	0.00	0.22	0.21
NCI-H1437	Lung	Lung Non Small Cell Carcinoma	0.00	0.05	0.04	0.07	0.05	0.06	0.01	0.05	0.04	0.08	0.07	0.02	0.02	0.01	0.02	0.00	0.01	0.12
NCI-H1299	Lung	Lung Non Small Cell Carcinoma	0.00	0.06	0.05	0.07	0.05	0.06	0.02	0.06	0.06	0.07	0.08	0.02	0.21	0.02	0.04	0.01	0.17	0.31
NCI-H1975	Lung	Lung Non Small Cell Carcinoma	0.03	0.08	0.06	0.08	0.05	0.05	0.01	0.05	0.05	0.08	0.07	0.02	0.03	0.03	0.01	0.01	0.04	0.20
CALU-3	Lung	Lung Non Small Cell Carcinoma	0.01	0.11	0.10	0.11	0.09	0.09	0.02	0.10	0.07	0.10	0.12	0.02	0.03	0.04	0.03	0.00	0.01	0.15
CAL-12T	Lung	Lung Non Small Cell Carcinoma	0.01	0.12	0.06	0.07	0.06	0.06	0.03	0.06	0.05	0.10	0.08	0.02	0.03	0.04	0.03	0.01	0.06	0.49
PC-14	Lung	Lung Non Small Cell Carcinoma	0.02	0.05	0.09	0.10	0.06	0.05	0.04	0.06	0.06	0.09	0.07	0.02	0.01	0.03	0.03	0.01	0.02	0.16
LCLC-103H	Lung	Lung Non Small Cell Carcinoma	0.10	0.09	0.08	0.10	0.07	0.07	0.01	0.06	0.05	0.08	0.08	0.02	0.04	0.05	0.02	0.02	0.02	0.15
NCI-H1915	Lung	Lung Non Small Cell Carcinoma	0.04	0.06	0.08	0.11	0.06	0.07	0.02	0.06	0.06	0.10	0.09	0.02	0.07	0.05	0.04	0.01	0.03	0.13
NCI-H2291	Lung	Lung Non Small Cell Carcinoma	0.03	0.06	0.08	0.11	0.06	0.07	0.05	0.05	0.05	0.07	0.08	0.02	0.03	0.02	0.02	0.01	0.02	0.20
NCI-H661	Lung	Lung Non Small Cell Carcinoma	0.02	0.07	0.09	0.10	0.07	0.07	0.04	0.05	0.06	0.08	0.08	0.02	0.19	0.04	0.03	0.01	0.16	0.25
NCI-H2228	Lung	Lung Non Small Cell Carcinoma	0.19	0.08	0.11	0.11	0.07	0.07	0.02	0.06	0.07	0.08	0.08	0.03	0.05	0.04	0.03	0.01	0.04	0.15
NCI-H1792	Lung	Lung Non Small Cell Carcinoma	0.00	0.09	0.08	0.09	0.07	0.07	0.03	0.06	0.05	0.09	0.08	0.02	0.04	0.02	0.02	0.01	0.01	0.13
SW900	Lung	Lung Non Small Cell Carcinoma	0.59	0.07	0.08	0.09	0.08	0.08	0.02	0.06	0.06	0.09	0.08	0.01	0.08	0.03	0.02	0.01	0.08	0.16
NCI-H2347	Lung	Lung Non Small Cell Carcinoma	0.00	0.09	0.07	0.10	0.07	0.07	0.02	0.07	0.06	0.10	0.09	0.02	0.04	0.02	0.02	0.00	0.02	0.16
NCI-H522	Lung	Lung Non Small Cell Carcinoma	0.00	0.10	0.06	0.09	0.06	0.07	0.03	0.06	0.05	0.10	0.09	0.02	0.27	0.03	0.03	0.01	0.25	0.19
NCI-H1650	Lung	Lung Non Small Cell Carcinoma	0.01	0.09	0.08	0.12	0.07	0.07	0.05	0.07	0.07	0.09	0.10	0.02	0.20	0.04	0.02	0.01	0.17	0.22
COR-L23	Lung	Lung Non Small Cell Carcinoma	0.16	0.10	0.07	0.10	0.07	0.08	0.04	0.06	0.06	0.09	0.10	0.02	0.05	0.03	0.02	0.01	0.06	0.20
NCI-H1355	Lung	Lung Non Small Cell Carcinoma	0.01	0.08	0.07	0.09	0.07	0.07	0.05	0.06	0.05	0.09	0.08	0.02	0.17	0.03	0.03	0.01	0.22	0.17
NCI-H2170	Lung	Lung Non Small Cell Carcinoma	0.00	0.08	0.07	0.08	0.06	0.07	0.01	0.06	0.05	0.10	0.07	0.02	0.03	0.04	0.02	0.00	0.02	0.25
NCI-H2085	Lung	Lung Non Small Cell Carcinoma	0.00	0.09	0.07	0.10	0.08	0.07	0.02	0.06	0.05	0.10	0.08	0.02	0.22	0.03	0.02	0.01	0.18	0.21
NCI-H2342	Lung	Lung Non Small Cell Carcinoma	0.00	0.08	0.06	0.09	0.07	0.07	0.04	0.06	0.06	0.10	0.08	0.02	0.16	0.03	0.02	0.01	0.26	0.31
NCI-H1395	Lung	Lung Non Small Cell Carcinoma	0.01	0.09	0.09	0.09	0.07	0.07	0.03	0.06	0.05	0.10	0.07	0.02	0.03	0.03	0.03	0.01	0.01	0.12
NCI-H1623	Lung	Lung Non Small Cell Carcinoma	0.00	0.09	0.07	0.08	0.07	0.07	0.04	0.06	0.05	0.10	0.08	0.01	0.05	0.03	0.02	0.01	0.13	0.15
NCI-H1838	Lung	Lung Non Small Cell Carcinoma	0.01	0.11	0.07	0.11	0.07	0.08	0.04	0.06	0.04	0.10	0.08	0.02	0.04	0.04	0.02	0.01	0.01	0.14
NCI-H2023	Lung	Lung Non Small Cell Carcinoma	0.01	0.08	0.07	0.08	0.07	0.07	0.03	0.06	0.06	0.08	0.08	0.01	0.04	0.03	0.02	0.00	0.07	0.13
NCI-H2444	Lung	Lung Non Small Cell Carcinoma	0.04	0.10	0.08	0.11	0.07	0.09	0.05	0.07	0.06	0.10	0.09	0.02	0.04	0.03	0.02	0.01	0.38	0.50
NCI-H1648	Lung	Lung Non Small Cell Carcinoma	0.01	0.07	0.07	0.09	0.07	0.06	0.03	0.07	0.07	0.09	0.09	0.01	0.05	0.04	0.03	0.00	0.02	0.19
CALU-6	Lung	Lung Non Small Cell Carcinoma	0.00	0.07	0.08	0.10	0.07	0.06	0.02	0.06	0.07	0.10	0.09	0.01	0.16	0.03	0.02	0.00	0.10	0.16
NCI-H2405	Lung	Lung Non Small Cell Carcinoma	0.03	0.09	0.07	0.11	0.06	0.08	0.02	0.07	0.06	0.10	0.07	0.02	0.04	0.03	0.02	0.01	0.04	0.18
NCI-H2009	Lung	Lung Non Small Cell Carcinoma	0.00	0.08	0.08	0.09	0.08	0.07	0.02	0.06	0.04	0.09	0.08	0.01	0.03	0.01	0.02	0.00	0.02	0.14
NCI-H1693	Lung	Lung Non Small Cell Carcinoma	0.01	0.09	0.06	0.11	0.08	0.07	0.01	0.06	0.06	0.08	0.08	0.01	0.29	0.03	0.03	0.00	0.26	0.31
NCI-H838	Lung	Lung Non Small Cell Carcinoma	0.01	0.13	0.08	0.10	0.09	0.09	0.05	0.07	0.06	0.12	0.12	0.02	0.06	0.05	0.04	0.01	0.03	0.15
SW1573	Lung	Lung Non Small Cell Carcinoma	0.16	0.16	0.08	0.12	0.07	0.08	0.05	0.07	0.06	0.10	0.10	0.02	0.25	0.06	0.07	0.01	0.21	0.18
NCI-H1944	Lung	Lung Non Small Cell Carcinoma	0.03	0.09	0.08	0.09	0.06	0.06	0.04	0.04	0.05	0.08	0.07	0.02	0.06	0.03	0.03	0.00	0.13	0.17
NCI-H2172	Lung	Lung Non Small Cell Carcinoma	0.01	0.09	0.07	0.09	0.06	0.06	0.03	0.04	0.05	0.07	0.08	0.02	0.09	0.03	0.03	0.01	0.08	0.20
HCC-78	Lung	Lung Non Small Cell Carcinoma	0.07	0.08	0.08	0.12	0.06	0.08	0.00	0.06	0.05	0.09	0.08	0.03	0.02	0.01	0.02	0.00	0.04	0.17
LK-2	Lung	Lung Non Small Cell Carcinoma	0.02	0.07	0.06	0.09	0.04	0.06	0.03	0.04	0.04	0.09	0.08	0.02	0.20	0.03	0.02	0.01	0.19	0.20
NCI-H2126	Lung	Lung Non Small Cell Carcinoma	0.01	0.09	0.07	0.08	0.07	0.07	0.04	0.04	0.24	0.19	0.14	0.04	0.20	0.16	0.05	0.06	0.25	0.31
HCC-827	Lung	Lung Non Small Cell Carcinoma	0.02	0.09	0.08	0.08	0.05	0.07	0.05	0.05	0.04	0.08	0.08	0.05	0.15	0.13	0.14	0.11	0.15	0.19
NCI-H1568	Lung	Lung Non Small Cell Carcinoma	0.02	0.08	0.06	0.08	0.05	0.06	0.02	0.04	0.04	0.09	0.05	0.00	0.30	0.04	0.03	0.00	0.26	0.17
NCI-H1869	Lung	Lung Non Small Cell Carcinoma	0.01	0.09	0.06	0.09	0.06	0.07	0.02	0.04	0.04	0.08	0.07	0.02	0.02	0.04	0.02	0.01	0.01	0.13
LU-65	Lung	Lung Non Small Cell Carcinoma	0.05	0.10	0.08	0.11	0.06	0.08	0.05	0.04	0.60	0.86	0.61	0.16	0.66	0.92	0.96	1.00	0.75	0.41
NCI-H1770	Lung	Lung Non Small Cell Carcinoma	0.01	0.09	0.08	0.09	0.06	0.07	0.03	0.04	0.05	0.09	0.08	0.03	0.24	0.03	0.03	0.01	0.16	0.22
NCI-H647	Lung	Lung Non Small Cell Carcinoma	0.00	0.10	0.08	0.10	0.07	0.06	0.02	0.06	0.05	0.08	0.08	0.02	0.03	0.04	0.03	0.01	0.01	0.16
NCI-H2087	Lung	Lung Non Small Cell Carcinoma	0.01	0.09	0.06	0.09	0.05	0.06	0.02	0.04	0.04	0.06	0.06	0.01	0.02	0.03	0.02	0.01	0.01	0.12
HCC-44	Lung	Lung Non Small Cell Carcinoma	0.01	0.09	0.08	0.08	0.05	0.06	0.02	0.05	0.03	0.07	0.07	0.01	0.00	0.05	0.00	0.00	0.01	0.13
NCI-H2135	Lung	Lung Non Small Cell Carcinoma	0.00	0.10	0.06	0.09	0.06	0.05	0.02	0.04	0.06	0.08	0.07	0.01	0.01	0.02	0.02	0.00	0.00	0.11
NCI-H1435	Lung	Lung Non Small Cell Carcinoma	0.01	0.10	0.08	0.12	0.08	0.07	0.06	0.07	0.06	0.09	0.08	0.02	0.03	0.03	0.02	0.01	0.01	0.17
HCC-15	Lung	Lung Non Small Cell Carcinoma	0.47	0.14	0.08	0.11	0.08	0.07	0.05	0.06	0.05	0.13	0.08	0.02	0.04	0.05	0.04	0.00	0.02	0.11
NCI-H2110	Lung	Lung Non Small Cell Carcinoma	0.01	0.14	0.10	0.12	0.09	0.09	0.03	0.08	0.07	0.09	0.10	0.02	0.03	0.04	0.03	0.02	0.01	0.12
DMS-114	Lung	Lung Small Cell Carcinoma	0.01	0.08	0.07	0.08	0.04	0.05	0.02	0.06	0.05	0.07	0.08	0.02	0.18	0.02	0.04	0.00	0.15	0.16
NCI-H510A	Lung	Lung Small Cell Carcinoma	0.02	0.06	0.08	0.06	0.08	0.07	0.03	0.07	0.05	0.07	0.10	0.02	0.04	0.05	0.02	0.01	0.02	0.12
NCI-H2171	Lung	Lung Small Cell Carcinoma	0.01	0.10	0.07	0.09	0.06	0.05	0.04	0.04	0.06	0.07	0.08	0.03	0.28	0.04	0.05	0.01	0.29	0.37
CPC-N	Lung	Lung Small Cell Carcinoma	0.00	0.07	0.07	0.12	0.06	0.07	0.02	0.06	0.05	0.09	0.09	0.02	0.25	0.03	0.03	0.00	0.07	0.19
NCI-H211	Lung	Lung Small Cell Carcinoma	0.02	0.09	0.08	0.10	0.06	0.06	0.03	0.06	0.04	0.10	0.08	0.02	0.21	0.04	0.03	0.01	0.23	0.33
NCI-H526	Lung	Lung Small Cell Carcinoma	0.04	0.07	0.08	0.10	0.07	0.06	0.03	0.05	0.05	0.08	0.08	0.03	0.27	0.04	0.02	0.01	0.25	0.25
NCI-H82	Lung	Lung Small Cell Carcinoma	0.04	0.08	0.08	0.09	0.06	0.07	0.02	0.06	0.08	0.09	0.08	0.02	0.30	0.05	0.03	0.01	0.26	0.25
SBC-5	Lung	Lung Small Cell Carcinoma	0.01	0.08	0.07	0.10	0.07	0.06	0.04	0.05	0.07	0.09	0.08	0.01	0.28	0.04	0.03	0.00	0.24	0.24
NCI-H446	Lung	Lung Small Cell Carcinoma	0.03	0.07	0.07	0.09	0.07	0.06	0.00	0.06	0.26	0.13	0.09	0.02	0.05	0.04	0.04	0.01	0.02	0.16
NCI-H146	Lung	Lung Small Cell Carcinoma	0.01	0.08	0.08	0.10	0.08	0.07	0.02	0.06	0.06	0.08	0.10	0.02	0.24	0.04	0.03	0.01	0.34	0.34
IST-SL1	Lung	Lung Small Cell Carcinoma	0.02	0.08	0.08	0.11	0.06	0.07	0.02	0.06	0.05	0.08	0.09	0.02	0.04	0.02	0.02	0.01	0.09	0.38
NCI-H1963	Lung	Lung Small Cell Carcinoma	0.07	0.13	0.23	0.15	0.04	0.08	0.05	0.04	0.13	0.13	0.10	0.02	0.30	0.03	0.06	0.04	0.40	0.60
MS-1	Lung	Lung Small Cell Carcinoma	0.93	0.47	0.40	0.39	0.11	0.09	0.04	0.06	0.51	0.54	0.33	0.16	0.54	0.52	0.45	0.48	0.43	0.44
LU-139	Lung	Lung Small Cell Carcinoma	0.00	0.10	0.07	0.11	0.07	0.08	0.03	0.06	0.34	0.17	0.10	0.02	0.31	0.05	0.05	0.02	0.31	0.43
NCI-H64	Lung	Lung Small Cell Carcinoma	0.01	0.07	0.06	0.07	0.05	0.06	0.03	0.05	0.21	0.11	0.10	0.02	0.03	0.05	0.03	0.01	0.07	0.40
COLO-668	Lung	Lung Small Cell Carcinoma	0.02	0.08	0.07	0.08	0.06	0.05	0.02	0.05	0.05	0.08	0.07	0.02	0.04	0.03	0.05	0.01	0.02	0.07
NCI-H196	Lung	Lung Small Cell Carcinoma	0.02	0.08	0.08	0.10	0.07	0.07	0.04	0.06	0.06	0.08	0.09	0.02	0.12	0.03	0.03	0.01	0.12	0.21
NCI-H69	Lung	Lung Small Cell Carcinoma	0.00	0.09	0.08	0.10	0.07	0.08	0.02	0.06	0.06	0.11	0.08	0.03	0.04	0.04	0.02	0.01	0.10	0.34
LU-165	Lung	Lung Small Cell Carcinoma	0.05	0.09	0.07	0.10	0.07	0.06	0.02	0.06	0.06	0.09	0.07	0.02	0.27	0.03	0.03	0.01	0.26	0.20
IST-SL2	Lung	Lung Small Cell Carcinoma	0.01	0.09	0.06	0.09	0.07	0.06	0.04	0.05	0.06	0.09	0.07	0.02	0.28	0.04	0.03	0.00	0.30	0.28
COR-L88	Lung	Lung Small Cell Carcinoma	0.00	0.08	0.07	0.08	0.04	0.07	0.03	0.05	0.05	0.09	0.09	0.02	0.04	0.02	0.02	0.01	0.02	0.16
LU-134-A	Lung	Lung Small Cell Carcinoma	0.05	0.09	0.07	0.10	0.07	0.06	0.02	0.06	0.05	0.09	0.07	0.01	0.26	0.03	0.03	0.00	0.11	0.18
NCI-H345	Lung	Lung Small Cell Carcinoma	0.01	0.14	0.08	0.12	0.09	0.08	0.05	0.08	0.07	0.13	0.13	0.02	0.06	0.09	0.04	0.01	0.06	0.25
NCI-H2227	Lung	Lung Small Cell Carcinoma	0.01	0.13	0.08	0.11	0.08	0.09	0.04	0.07	0.05	0.11	0.13	0.03	0.33	0.05	0.09	0.01	0.32	0.25
NCI-H841	Lung	Lung Small Cell Carcinoma	0.02	0.15	0.08	0.11	0.09	0.09	0.03	0.07	0.06	0.10	0.09	0.03	0.35	0.07	0.11	0.01	0.31	0.25
SBC-3	Lung	Lung Small Cell Carcinoma	0.12	0.09	0.09	0.09	0.06	0.06	0.04	0.05	0.05	0.12	0.10	0.02	0.29	0.05	0.03	0.01	0.29	0.26
NCI-H187	Lung	Lung Small Cell Carcinoma	0.56	0.16	0.07	0.10	0.08	0.08	0.02	0.07	0.05	0.09	0.08	0.01	0.03	0.04	0.02	0.01	0.09	0.11
HCC-33	Lung	Lung Small Cell Carcinoma	0.07	0.08	0.09	0.11	0.08	0.09	0.03	0.07	0.25	0.11	0.10	0.03	0.29	0.04	0.03	0.01	0.39	0.46
NCI-H1341	Lung	Lung Small Cell Carcinoma	0.78	0.66	0.61	0.59	0.13	0.11	0.03	0.05	0.46	0.21	0.19	0.05	0.30	0.08	0.04	0.02	0.30	0.50
NCI-H2066	Lung	Lung Small Cell Carcinoma	0.02	0.13	0.09	0.13	0.08	0.08	0.05	0.07	0.06	0.11	0.09	0.02	0.05	0.04	0.03	0.01	0.05	0.17
NCI-H128	Lung	Lung Small Cell Carcinoma	0.06	0.10	0.10	0.13	0.06	0.05	0.03	0.04	0.70	0.68	0.36	0.49	0.53	0.56	0.31	0.47	0.57	0.73
NCI-H1105	Lung	Lung Small Cell Carcinoma	0.00	0.09	0.06	0.08	0.05	0.05	0.03	0.05	0.05	0.06	0.08	0.01	0.23	0.03	0.02	0.01	0.22	0.36
SBC-1	Lung	Lung Small Cell Carcinoma	0.02	0.10	0.08	0.11	0.06	0.07	0.02	0.05	0.06	0.08	0.08	0.02	0.28	0.03	0.03	0.00	0.29	0.46
DMS-53	Lung	Lung Small Cell Carcinoma	0.01	0.10	0.06	0.09	0.06	0.05	0.01	0.04	0.04	0.09	0.07	0.01	0.02	0.02	0.02	0.00	0.00	0.11

		TargetID	cg21539223	cg16456337	cg15956794	cg14773728	cg17573603	cg17919331	cg23965551	cg00522636	cg13518195	cg26459500	cg01092811	cg06363041	cg25437259	cg14506646	cg14861803	cg11162249	cg17789800	cg00261665
		UCSC_REFGENE_NAME	DCP2								NUDT16									
		UCSC_REFGENE_GROUP	TSS1500	TSS1500	TSS200	TSS200	TSS200	TSS200	1stExon;5'UTR	1stExon	TSS1500	TSS1500	TSS1500	TSS200	TSS200	TSS200	TSS200	5'UTR;1stExon	Body	Body
**Cell Line**	**Tissue**	**Cell Line Type**																		
NCI-H1092	Lung	Lung Small Cell Carcinoma	0.01	0.08	0.07	0.08	0.05	0.06	0.04	0.03	0.06	0.08	0.07	0.02	0.23	0.03	0.03	0.01	0.03	0.19
NCI-H1155	Lung	Lung Small Cell Carcinoma	0.01	0.08	0.07	0.08	0.05	0.06	0.01	0.05	0.04	0.09	0.07	0.01	0.25	0.04	0.03	0.00	0.40	0.36
NCI-H1688	Lung	Lung Small Cell Carcinoma	0.03	0.09	0.06	0.09	0.05	0.06	0.01	0.04	0.04	0.08	0.06	0.02	0.01	0.03	0.02	0.01	0.00	0.13
NCI-H2081	Lung	Lung Small Cell Carcinoma	0.01	0.09	0.06	0.10	0.06	0.05	0.04	0.04	0.07	0.08	0.07	0.02	0.20	0.05	0.01	0.00	0.17	0.28
NCI-H2196	Lung	Lung Small Cell Carcinoma	0.00	0.10	0.05	0.10	0.06	0.07	0.03	0.05	0.05	0.08	0.07	0.02	0.01	0.03	0.02	0.00	0.05	0.44
NCI-H209	Lung	Lung Small Cell Carcinoma	0.05	0.08	0.07	0.08	0.06	0.05	0.03	0.04	0.04	0.11	0.07	0.02	0.04	0.03	0.02	0.00	0.04	0.18
NCI-H2029	Lung	Lung Small Cell Carcinoma	0.02	0.09	0.07	0.10	0.06	0.07	0.04	0.04	0.05	0.10	0.07	0.02	0.02	0.03	0.03	0.01	0.03	0.22
NCI-H1694	Lung	Lung Small Cell Carcinoma	0.02	0.10	0.08	0.10	0.07	0.06	0.02	0.05	0.78	0.54	0.24	0.05	0.32	0.22	0.20	0.06	0.52	0.88
RERF-LC-FM	Lung	Lung Small Cell Carcinoma	0.00	0.09	0.06	0.08	0.07	0.05	0.03	0.04	0.04	0.08	0.08	0.01	0.02	0.04	0.02	0.01	0.02	0.24
LU-135	Lung	Lung Small Cell Carcinoma	0.13	0.11	0.08	0.10	0.06	0.06	0.02	0.05	0.23	0.16	0.12	0.02	0.30	0.05	0.05	0.02	0.35	0.34
NCI-H748	Lung	Lung Small Cell Carcinoma	0.00	0.11	0.08	0.09	0.06	0.07	0.02	0.05	0.04	0.06	0.07	0.02	0.33	0.03	0.03	0.00	0.19	0.23
NCI-H378	Lung	Lung Small Cell Carcinoma	0.21	0.18	0.09	0.10	0.05	0.07	0.01	0.05	0.03	0.08	0.08	0.01	0.06	0.03	0.03	0.00	0.02	0.17
NCI-H524	Lung	Lung Small Cell Carcinoma	0.01	0.16	0.08	0.08	0.08	0.08	0.05	0.07	0.30	0.13	0.14	0.02	0.28	0.06	0.08	0.02	0.30	0.50
NCI-H1876	Lung	Lung Small Cell Carcinoma	0.77	0.58	0.52	0.54	0.12	0.09	0.06	0.06	0.07	0.09	0.09	0.03	0.31	0.05	0.10	0.01	0.40	0.44
DMS-79	Lung	Lung Small Cell Carcinoma	0.02	0.15	0.09	0.09	0.08	0.09	0.03	0.07	0.08	0.10	0.11	0.03	0.33	0.05	0.12	0.01	0.34	0.34
NCI-H1417	Lung	Lung Small Cell Carcinoma	0.01	0.11	0.07	0.08	0.07	0.07	0.05	0.06	0.05	0.16	0.07	0.02	0.02	0.04	0.02	0.00	0.04	0.24
NCI-H1436	Lung	Lung Small Cell Carcinoma	0.03	0.12	0.09	0.12	0.08	0.08	0.03	0.07	0.06	0.09	0.08	0.02	0.28	0.03	0.07	0.01	0.28	0.47
COR-L311	Lung	Lung Small Cell Carcinoma	0.01	0.11	0.08	0.10	0.07	0.08	0.03	0.08	0.06	0.10	0.09	0.03	0.31	0.05	0.09	0.01	0.30	0.39
COR-L303	Lung	Lung Small Cell Carcinoma	0.01	0.11	0.07	0.10	0.07	0.07	0.03	0.06	0.05	0.09	0.08	0.03	0.28	0.04	0.06	0.01	0.13	0.46
NCI-H1836	Lung	Lung Small Cell Carcinoma	0.03	0.13	0.07	0.09	0.08	0.06	0.05	0.07	0.08	0.11	0.08	0.02	0.24	0.06	0.08	0.01	0.14	0.22
COR-L279	Lung	Lung Small Cell Carcinoma	0.02	0.12	0.09	0.10	0.08	0.09	0.03	0.07	0.07	0.10	0.09	0.03	0.04	0.04	0.03	0.01	0.02	0.20
NCI-H1048	Lung	Lung Small Cell Carcinoma	0.01	0.11	0.09	0.11	0.08	0.08	0.05	0.07	0.06	0.11	0.09	0.03	0.33	0.04	0.08	0.01	0.27	0.22
NCI-H847	Lung	Lung Small Cell Carcinoma	0.02	0.13	0.09	0.11	0.07	0.09	0.02	0.08	0.06	0.13	0.11	0.03	0.32	0.07	0.09	0.01	0.28	0.27
DMS-273	Lung	Lung Small Cell Carcinoma	0.13	0.16	0.13	0.11	0.09	0.10	0.05	0.08	0.21	0.11	0.09	0.03	0.32	0.05	0.07	0.01	0.29	0.32
NCI-H2141	Lung	Lung Small Cell Carcinoma	0.00	0.07	0.04	0.06	0.06	0.07	0.03	0.05	0.05	0.08	0.07	0.02	0.30	0.03	0.05	0.01	0.24	0.15
NCI-H250	Lung	Lung Small Cell Carcinoma	0.01	0.14	0.09	0.10	0.08	0.09	0.04	0.08	0.06	0.10	0.09	0.02	0.06	0.04	0.05	0.01	0.33	0.42
NCI-H510A	Lung	Lung Small Cell Carcinoma	0.01	0.07	0.06	0.07	0.04	0.05	0.03	0.04	0.03	0.06	0.05	0.01	0.01	0.03	0.03	0.01	0.01	0.11
NCI-H1304	Lung	Lung Small Cell Carcinoma	0.42	0.35	0.48	0.39	0.13	0.07	0.03	0.05	0.06	0.07	0.06	0.01	0.10	0.03	0.03	0.00	0.22	0.48
SW1271	Lung	Lung Small Cell Carcinoma	0.04	0.09	0.08	0.11	0.07	0.06	0.01	0.06	0.05	0.10	0.07	0.00	0.02	0.02	0.01	0.01	0.01	0.20
COR-L95	Lung	Lung Small Cell Carcinoma	0.02	0.11	0.08	0.09	0.07	0.07	0.03	0.06	0.04	0.09	0.07	0.02	0.30	0.03	0.03	0.01	0.20	0.34
NCI-H226	Lung	Lung Squamous Cell Carcinoma	0.01	0.09	0.07	0.09	0.06	0.05	0.01	0.05	0.04	0.07	0.07	0.02	0.02	0.03	0.02	0.00	0.05	0.14
NCI-H520	Lung	Lung Squamous Cell Carcinoma	0.05	0.07	0.08	0.12	0.08	0.08	0.03	0.07	0.05	0.08	0.10	0.03	0.15	0.04	0.03	0.01	0.16	0.20
EBC-1	Lung	Lung Squamous Cell Carcinoma	0.04	0.09	0.06	0.09	0.06	0.07	0.05	0.05	0.07	0.09	0.08	0.02	0.03	0.03	0.02	0.01	0.28	0.55
SK-MES-1	Lung	Lung Squamous Cell Carcinoma	0.00	0.08	0.08	0.11	0.06	0.07	0.03	0.06	0.05	0.09	0.09	0.02	0.04	0.03	0.03	0.00	0.01	0.13
LC-1F	Lung	Lung Squamous Cell Carcinoma	0.01	0.11	0.09	0.12	0.07	0.07	0.02	0.07	0.04	0.09	0.07	0.03	0.32	0.04	0.03	0.01	0.29	0.24
KNS-62	Lung	Lung Squamous Cell Carcinoma	0.00	0.09	0.07	0.09	0.06	0.07	0.04	0.06	0.05	0.09	0.08	0.02	0.00	0.03	0.02	0.00	0.01	0.15
LOU-NH91	Lung	Lung Squamous Cell Carcinoma	0.00	0.07	0.08	0.12	0.07	0.08	0.04	0.04	0.05	0.10	0.11	0.02	0.02	0.04	0.01	0.01	0.01	0.11
RERF-LC-SQ1	Lung	Lung Squamous Cell Carcinoma	0.00	0.10	0.07	0.09	0.07	0.06	0.03	0.05	0.05	0.09	0.07	0.02	0.15	0.02	0.03	0.01	0.32	0.39
EPLC-272H	Lung	Lung Squamous Cell Carcinoma	0.05	0.14	0.06	0.11	0.06	0.06	0.03	0.05	0.05	0.09	0.09	0.01	0.11	0.03	0.02	0.01	0.24	0.20
HARA	Lung	Lung Squamous Cell Carcinoma	0.09	0.10	0.07	0.10	0.05	0.06	0.02	0.05	0.05	0.09	0.08	0.01	0.23	0.04	0.03	0.01	0.31	0.28
LC-1-SQ	Lung	Lung Squamous Cell Carcinoma	0.02	0.15	0.10	0.12	0.08	0.08	0.04	0.09	0.04	0.12	0.10	0.03	0.32	0.06	0.09	0.01	0.28	0.39
NCI-H1703	Lung	Mixed Adenosquamous Carcinoma	0.01	0.08	0.07	0.11	0.07	0.08	0.04	0.07	0.05	0.10	0.13	0.03	0.26	0.05	0.04	0.01	0.29	0.28
EMC-BAC-2	Lung	Ns	0.03	0.11	0.07	0.11	0.07	0.08	0.04	0.08	0.06	0.13	0.11	0.02	0.06	0.04	0.04	0.01	0.09	0.19
EMC-BAC-1	Lung	Ns	0.02	0.14	0.09	0.11	0.09	0.09	0.03	0.09	0.05	0.09	0.12	0.02	0.06	0.06	0.04	0.00	0.01	0.15
NCI-H740	Lung	Ns	0.01	0.13	0.09	0.10	0.07	0.07	0.03	0.06	0.06	0.09	0.10	0.02	0.19	0.05	0.05	0.01	0.18	0.40
HCC-366	Lung	Ns	0.00	0.13	0.09	0.10	0.07	0.08	0.04	0.07	0.06	0.12	0.11	0.02	0.07	0.04	0.04	0.00	0.14	0.19
COR-L32	Lung	Small Cell Carcinoma	0.01	0.14	0.09	0.10	0.08	0.08	0.05	0.05	0.29	0.18	0.12	0.03	0.34	0.10	0.17	0.04	0.24	0.27
TASK1	Na	Neuroectodermal Tumor	0.32	0.10	0.07	0.09	0.08	0.08	0.03	0.06	0.86	0.81	0.71	0.59	0.90	0.95	0.97	0.99	0.93	0.92
SK-GT-2	Oesophagus	Ns	0.09	0.12	0.09	0.11	0.07	0.07	0.06	0.07	0.06	0.13	0.11	0.02	0.03	0.06	0.04	0.01	0.03	0.14
ESO26	Oesophagus	Oesophagus Adenocarcinoma	0.07	0.14	0.08	0.10	0.07	0.09	0.09	0.07	0.05	0.12	0.09	0.02	0.03	0.05	0.05	0.00	0.02	0.12
OACM5-1	Oesophagus	Oesophagus Adenocarcinoma	0.01	0.15	0.07	0.11	0.07	0.09	0.03	0.08	0.07	0.12	0.10	0.02	0.04	0.06	0.04	0.01	0.03	0.16
FLO-1	Oesophagus	Oesophagus Adenocarcinoma	0.00	0.14	0.07	0.10	0.07	0.08	0.05	0.07	0.07	0.13	0.10	0.02	0.35	0.09	0.12	0.01	0.31	0.29
SK-GT-4	Oesophagus	Oesophagus Adenocarcinoma	0.02	0.12	0.07	0.10	0.07	0.09	0.04	0.07	0.07	0.11	0.09	0.03	0.03	0.05	0.03	0.00	0.02	0.24
OACP4C	Oesophagus	Oesophagus Adenocarcinoma	0.02	0.06	0.08	0.11	0.07	0.07	0.03	0.06	0.07	0.11	0.08	0.02	0.04	0.04	0.03	0.01	0.02	0.12
ESO51	Oesophagus	Oesophagus Adenocarcinoma	0.00	0.06	0.07	0.08	0.05	0.06	0.04	0.05	0.05	0.10	0.08	0.02	0.01	0.03	0.01	0.00	0.01	0.13
KYAE-1	Oesophagus	Oesophagus Adenocarcinoma	0.02	0.15	0.08	0.13	0.07	0.09	0.05	0.07	0.05	0.10	0.08	0.03	0.02	0.05	0.04	0.02	0.01	0.15
OE19	Oesophagus	Oesophagus Adenocarcinoma	0.01	0.10	0.08	0.09	0.06	0.08	0.06	0.05	0.04	0.08	0.07	0.02	0.02	0.04	0.02	0.01	0.02	0.14
KYSE-410	Oesophagus	Oesophagus Carcinoma	0.22	0.09	0.06	0.07	0.06	0.04	0.02	0.05	0.38	0.36	0.32	0.01	0.46	0.45	0.49	0.39	0.49	0.55
KYSE-140	Oesophagus	Oesophagus Carcinoma	0.00	0.10	0.06	0.07	0.05	0.05	0.02	0.04	0.04	0.07	0.07	0.00	0.28	0.04	0.03	0.00	0.37	0.59
TE-1	Oesophagus	Oesophagus Carcinoma	0.00	0.09	0.05	0.08	0.04	0.06	0.02	0.04	0.02	0.07	0.06	0.00	0.01	0.02	0.01	0.00	0.00	0.13
TE-10	Oesophagus	Oesophagus Carcinoma	0.01	0.10	0.06	0.09	0.07	0.06	0.02	0.05	0.03	0.06	0.08	0.01	0.14	0.02	0.03	0.00	0.21	0.42
HCE-4	Oesophagus	Oesophagus Carcinoma	0.20	0.11	0.07	0.09	0.05	0.07	0.00	0.04	0.05	0.07	0.07	0.01	0.22	0.02	0.02	0.00	0.30	0.33
TE-15	Oesophagus	Oesophagus Carcinoma	0.00	0.11	0.08	0.09	0.06	0.06	0.01	0.04	0.05	0.07	0.07	0.01	0.02	0.03	0.02	0.00	0.00	0.14
EC-GI-10	Oesophagus	Oesophagus Carcinoma	0.02	0.10	0.07	0.09	0.05	0.05	0.02	0.04	0.04	0.09	0.06	0.01	0.27	0.03	0.04	0.00	0.30	0.31
KYSE-70	Oesophagus	Oesophagus Carcinoma	0.28	0.08	0.08	0.09	0.06	0.06	0.05	0.04	0.51	0.57	0.50	0.17	0.58	0.61	0.62	0.58	0.47	0.66
TE-8	Oesophagus	Oesophagus Carcinoma	0.00	0.09	0.07	0.09	0.05	0.06	0.03	0.04	0.04	0.11	0.08	0.01	0.27	0.04	0.04	0.00	0.28	0.23
KYSE-520	Oesophagus	Oesophagus Carcinoma	0.08	0.09	0.06	0.10	0.05	0.06	0.04	0.04	0.04	0.09	0.08	0.01	0.21	0.03	0.03	0.00	0.28	0.24
OE33	Oesophagus	Oesophagus Metaplasia	0.03	0.10	0.06	0.09	0.06	0.08	0.04	0.05	0.05	0.09	0.07	0.02	0.02	0.03	0.02	0.03	0.04	0.34
TE-4	Oesophagus	Oesophagus Squamous Cell Carcinoma	0.02	0.11	0.07	0.12	0.09	0.08	0.06	0.07	0.06	0.12	0.13	0.03	0.27	0.05	0.07	0.01	0.25	0.23
T-T	Oesophagus	Oesophagus Squamous Cell Carcinoma	0.02	0.09	0.09	0.12	0.08	0.08	0.04	0.07	0.07	0.10	0.09	0.02	0.30	0.04	0.04	0.01	0.29	0.54
TE-11	Oesophagus	Oesophagus Squamous Cell Carcinoma	0.00	0.09	0.07	0.08	0.05	0.05	0.02	0.04	0.05	0.09	0.07	0.00	0.26	0.03	0.02	0.00	0.21	0.25
KYSE-270	Oesophagus	Oesophagus Squamous Cell Carcinoma	0.19	0.21	0.12	0.11	0.06	0.06	0.02	0.04	0.05	0.08	0.08	0.01	0.19	0.04	0.03	0.00	0.16	0.17
TE-6	Oesophagus	Oesophagus Squamous Cell Carcinoma	0.00	0.10	0.06	0.07	0.06	0.06	0.03	0.05	0.04	0.09	0.07	0.02	0.29	0.03	0.03	0.01	0.33	0.32
TE-5	Oesophagus	Oesophagus Squamous Cell Carcinoma	0.01	0.10	0.06	0.09	0.04	0.06	0.02	0.04	0.05	0.09	0.07	0.01	0.29	0.03	0.03	0.01	0.32	0.52
KYSE-510	Oesophagus	Oesophagus Squamous Cell Carcinoma	0.01	0.10	0.08	0.10	0.06	0.07	0.03	0.06	0.05	0.08	0.07	0.02	0.30	0.04	0.03	0.01	0.34	0.52
KYSE-450	Oesophagus	Oesophagus Squamous Cell Carcinoma	0.02	0.11	0.07	0.09	0.06	0.07	0.04	0.05	0.05	0.08	0.07	0.01	0.28	0.03	0.03	0.00	0.39	0.44
KYSE-50	Oesophagus	Oesophagus Squamous Cell Carcinoma	0.02	0.09	0.08	0.09	0.06	0.07	0.03	0.05	0.37	0.31	0.30	0.35	0.51	0.48	0.47	0.49	0.42	0.61
OE21	Oesophagus	Oesophagus Squamous Cell Carcinoma	0.01	0.08	0.06	0.07	0.06	0.07	0.04	0.04	0.05	0.10	0.07	0.02	0.28	0.03	0.02	0.00	0.28	0.26
KYSE-180	Oesophagus	Oesophagus Squamous Cell Carcinoma	0.01	0.09	0.07	0.08	0.06	0.07	0.03	0.05	0.04	0.07	0.08	0.01	0.30	0.03	0.04	0.00	0.27	0.30
TE-12	Oesophagus	Oesophagus Squamous Cell Carcinoma	0.05	0.11	0.07	0.10	0.06	0.06	0.03	0.04	0.05	0.09	0.07	0.02	0.28	0.05	0.03	0.00	0.36	0.24
COLO-680N	Oesophagus	Oesophagus Squamous Cell Carcinoma	0.04	0.08	0.05	0.07	0.06	0.05	0.05	0.04	0.05	0.08	0.08	0.02	0.10	0.04	0.02	0.01	0.18	0.23
KYSE-220	Oesophagus	Oesophagus Squamous Cell Carcinoma	0.01	0.07	0.06	0.09	0.05	0.06	0.03	0.04	0.04	0.09	0.07	0.02	0.26	0.05	0.03	0.00	0.39	0.52
TE-9	Oesophagus	Oesophagus Squamous Cell Carcinoma	0.15	0.11	0.07	0.09	0.05	0.06	0.04	0.04	0.05	0.08	0.07	0.02	0.30	0.04	0.03	0.01	0.30	0.26
OVMIU	Ovary	Adenocarcinoma	0.02	0.08	0.07	0.09	0.07	0.06	0.02	0.05	0.04	0.07	0.08	0.01	0.26	0.03	0.02	0.01	0.21	0.27
OVK-18	Ovary	Edometroid Carcinoma	0.01	0.09	0.07	0.10	0.06	0.07	0.03	0.05	0.05	0.08	0.11	0.02	0.27	0.04	0.03	0.00	0.27	0.20
KGN	Ovary	Granulosa Cell Tumour	0.01	0.09	0.08	0.09	0.04	0.06	0.02	0.05	0.04	0.08	0.07	0.01	0.12	0.03	0.02	0.00	0.11	0.20
OV-17R	Ovary	Ns	0.02	0.17	0.08	0.12	0.07	0.09	0.04	0.08	0.07	0.08	0.11	0.02	0.04	0.07	0.04	0.01	0.02	0.30
IOSE-523(-)	Ovary	Ns	0.02	0.14	0.09	0.09	0.08	0.07	0.04	0.07	0.06	0.09	0.09	0.02	0.02	0.05	0.04	0.01	0.02	0.16
UWB1-289	Ovary	Ns	0.02	0.13	0.10	0.11	0.07	0.08	0.04	0.06	0.05	0.11	0.10	0.03	0.02	0.04	0.03	0.02	0.01	0.16
JHOS-3	Ovary	Ns	0.01	0.16	0.09	0.13	0.10	0.11	0.04	0.08	0.07	0.12	0.12	0.02	0.08	0.08	0.05	0.02	0.15	0.20
IOSE-397	Ovary	Ns	0.02	0.09	0.09	0.12	0.08	0.09	0.02	0.06	0.06	0.10	0.09	0.03	0.04	0.04	0.02	0.01	0.02	0.14
IOSE-364(-)	Ovary	Ns	0.02	0.12	0.08	0.10	0.08	0.08	0.03	0.07	0.07	0.08	0.08	0.02	0.06	0.03	0.03	0.00	0.04	0.16
IOSE-75-16SV40	Ovary	Ns	0.02	0.15	0.10	0.12	0.09	0.09	0.03	0.08	0.07	0.12	0.09	0.03	0.05	0.04	0.05	0.01	0.04	0.16
OV-56	Ovary	Ns	0.02	0.10	0.10	0.10	0.07	0.08	0.03	0.08	0.06	0.09	0.10	0.03	0.28	0.05	0.05	0.01	0.23	0.24
SK-OV-3	Ovary	Ovary Adenocarcinoma	0.01	0.09	0.07	0.09	0.05	0.05	0.01	0.05	0.04	0.07	0.10	0.01	0.18	0.02	0.01	0.00	0.28	0.34
IGROV-1	Ovary	Ovary Adenocarcinoma	0.01	0.07	0.07	0.09	0.04	0.06	0.02	0.05	0.04	0.07	0.06	0.01	0.29	0.02	0.02	0.01	0.24	0.15
OVKATE	Ovary	Ovary Adenocarcinoma	0.00	0.10	0.07	0.07	0.06	0.06	0.01	0.04	0.04	0.07	0.09	0.01	0.03	0.04	0.02	0.01	0.01	0.12
OVISE	Ovary	Ovary Adenocarcinoma	0.00	0.09	0.07	0.09	0.06	0.05	0.03	0.06	0.06	0.09	0.08	0.02	0.30	0.04	0.05	0.00	0.31	0.26
OVCAR-3	Ovary	Ovary Adenocarcinoma	0.00	0.10	0.07	0.09	0.06	0.06	0.02	0.04	0.04	0.09	0.08	0.02	0.33	0.04	0.03	0.01	0.32	0.26
A2780	Ovary	Ovary Adenocarcinoma	0.01	0.12	0.09	0.09	0.07	0.06	0.03	0.05	0.04	0.07	0.07	0.03	0.26	0.04	0.04	0.01	0.35	0.37
SW626	Ovary	Ovary Adenocarcinoma	0.01	0.11	0.06	0.09	0.05	0.07	0.03	0.06	0.05	0.08	0.09	0.03	0.05	0.03	0.02	0.01	0.15	0.19
CAOV-3	Ovary	Ovary Adenocarcinoma	0.01	0.11	0.07	0.08	0.06	0.06	0.00	0.05	0.04	0.07	0.09	0.03	0.02	0.03	0.02	0.00	0.00	0.13
TOV-21G	Ovary	Ovary Adenocarcinoma	0.18	0.09	0.07	0.10	0.06	0.06	0.04	0.05	0.05	0.08	0.07	0.01	0.32	0.05	0.03	0.01	0.24	0.19
OV-90	Ovary	Ovary Adenocarcinoma	0.02	0.08	0.08	0.10	0.06	0.05	0.02	0.04	0.04	0.10	0.10	0.02	0.05	0.04	0.02	0.00	0.11	0.17
JHOS-2	Ovary	Ovary Adenocarcinoma	0.20	0.12	0.08	0.11	0.08	0.08	0.04	0.07	0.05	0.10	0.09	0.02	0.03	0.04	0.04	0.01	0.06	0.16
JHOS-4	Ovary	Ovary Adenocarcinoma	0.01	0.14	0.09	0.11	0.07	0.08	0.05	0.08	0.05	0.11	0.10	0.03	0.13	0.06	0.06	0.01	0.11	0.23
CAOV-4	Ovary	Ovary Adenocarcinoma	0.02	0.12	0.09	0.11	0.08	0.09	0.04	0.08	0.07	0.10	0.12	0.04	0.28	0.05	0.07	0.01	0.28	0.25

TargetID			cg21539223	cg16456337	cg15956794	cg14773728	cg17573603	cg17919331	cg23965551	cg00522636	cg13518195	cg26459500	cg01092811	cg06363041	cg25437259	cg14506646	cg14861803	cg11162249	cg17789800	cg00261665
UCSC_REFGENE_NAME			DCP2								NUDT16									
UCSC_REFGENE_GROUP			TSS1500	TSS1500	TSS200	TSS200	TSS200	TSS200	1stExon;5'UTR	1stExon	TSS1500	TSS1500	TSS1500	TSS200	TSS200	TSS200	TSS200	5'UTR;1stExon	Body	Body
**Cell Line**	**Tissue**	**Cell Line Type**																		
OVTOKO	Ovary	Ovary Adenocarcinoma	0.01	0.14	0.09	0.12	0.08	0.09	0.05	0.08	0.07	0.10	0.09	0.03	0.31	0.06	0.08	0.01	0.33	0.25
OVCAR-4	Ovary	Ovary Carcinoma	0.00	0.08	0.06	0.09	0.06	0.05	0.01	0.05	0.05	0.08	0.07	0.01	0.05	0.02	0.03	0.02	0.18	0.20
OVCAR-8	Ovary	Ovary Carcinoma	0.01	0.10	0.08	0.10	0.06	0.05	0.02	0.06	0.05	0.07	0.08	0.02	0.23	0.04	0.02	0.01	0.21	0.21
KURAMOCHI	Ovary	Ovary Carcinoma	0.00	0.10	0.06	0.07	0.05	0.05	0.03	0.04	0.06	0.08	0.10	0.02	0.20	0.02	0.04	0.01	0.21	0.21
OAW-28	Ovary	Ovary Carcinoma	0.02	0.09	0.07	0.09	0.06	0.07	0.02	0.07	0.05	0.09	0.13	0.02	0.23	0.05	0.04	0.01	0.22	0.21
FU-OV-1	Ovary	Ovary Carcinoma	0.00	0.10	0.08	0.08	0.07	0.07	0.01	0.05	0.06	0.08	0.11	0.02	0.31	0.04	0.03	0.01	0.24	0.21
OVCA420	Ovary	Ovary Carcinoma	0.02	0.12	0.08	0.11	0.08	0.08	0.05	0.07	0.06	0.06	0.09	0.03	0.20	0.03	0.07	0.01	0.28	0.23
OV-7	Ovary	Ovary Carcinoma	0.01	0.14	0.10	0.12	0.09	0.09	0.02	0.08	0.08	0.10	0.11	0.02	0.03	0.03	0.03	0.01	0.02	0.17
PEO1	Ovary	Ovary Carcinoma	0.09	0.15	0.09	0.13	0.07	0.08	0.02	0.08	0.07	0.14	0.09	0.02	0.03	0.05	0.03	0.01	0.06	0.14
TYK-NU	Ovary	Ovary Carcinoma	0.41	0.28	0.22	0.16	0.08	0.09	0.04	0.07	0.46	0.47	0.40	0.38	0.53	0.54	0.54	0.53	0.92	0.94
OVCAR-5	Ovary	Ovary Carcinoma	0.01	0.16	0.10	0.12	0.10	0.10	0.04	0.08	0.07	0.12	0.09	0.01	0.11	0.04	0.04	0.01	0.03	0.09
DOV13	Ovary	Ovary Carcinoma	0.10	0.10	0.06	0.09	0.06	0.05	0.02	0.06	0.04	0.07	0.06	0.02	0.09	0.04	0.03	0.01	0.06	0.12
Hey	Ovary	Ovary Carcinoma	0.25	0.09	0.07	0.10	0.06	0.07	0.03	0.06	0.05	0.07	0.09	0.02	0.16	0.05	0.02	0.01	0.20	0.20
OVCA433	Ovary	Ovary Carcinoma	0.77	0.18	0.23	0.16	0.08	0.07	0.01	0.06	0.06	0.08	0.08	0.02	0.21	0.02	0.01	0.00	0.23	0.23
ES-2	Ovary	Ovary Carcinoma (Clear Cell)	0.05	0.10	0.07	0.08	0.06	0.07	0.02	0.06	0.04	0.07	0.10	0.02	0.28	0.03	0.03	0.00	0.20	0.23
RMG-I	Ovary	Ovary Carcinoma (Clear Cell)	0.01	0.10	0.07	0.09	0.06	0.07	0.03	0.05	0.04	0.08	0.10	0.02	0.24	0.03	0.02	0.01	0.21	0.21
EFO-27	Ovary	Ovary Carcinoma (Mucinous)	0.01	0.06	0.10	0.09	0.07	0.06	0.02	0.06	0.05	0.09	0.08	0.02	0.29	0.04	0.02	0.01	0.27	0.18
OAW-42	Ovary	Ovary Carcinoma (Mucinous)	0.01	0.10	0.08	0.09	0.07	0.07	0.04	0.07	0.06	0.08	0.10	0.04	0.31	0.04	0.05	0.02	0.31	0.24
EFO-21	Ovary	Ovary Carcinoma (Serous)	0.01	0.08	0.08	0.10	0.05	0.07	0.01	0.05	0.04	0.06	0.08	0.02	0.09	0.02	0.02	0.00	0.25	0.22
OC-314	Ovary	Ovary Carcinoma (Serous)	0.02	0.10	0.06	0.09	0.06	0.06	0.02	0.04	0.03	0.07	0.07	0.01	0.22	0.03	0.02	0.00	0.21	0.19
PA-1	Ovary	Ovary Teratoma	0.02	0.09	0.09	0.09	0.06	0.06	0.02	0.05	0.05	0.08	0.09	0.02	0.32	0.05	0.03	0.01	0.30	0.27
SU8686	Pancreas	Adenocarcinoma	0.01	0.16	0.11	0.12	0.08	0.09	0.04	0.07	0.07	0.11	0.11	0.03	0.06	0.11	0.03	0.01	0.03	0.19
QGP-1	Pancreas	Islet Cell Carcinoma	0.01	0.14	0.08	0.10	0.07	0.09	0.05	0.06	0.06	0.12	0.13	0.02	0.36	0.05	0.08	0.00	0.33	0.26
PL18	Pancreas	Ns	0.02	0.06	0.09	0.12	0.08	0.07	0.02	0.07	0.06	0.08	0.09	0.02	0.10	0.02	0.03	0.01	0.06	0.17
HS-766T	Pancreas	Pancreas Adenocarcinoma	0.11	0.07	0.10	0.11	0.08	0.08	0.03	0.06	0.05	0.11	0.09	0.02	0.05	0.02	0.02	0.01	0.03	0.19
HPAC	Pancreas	Pancreas Adenocarcinoma	0.02	0.15	0.08	0.11	0.07	0.08	0.05	0.08	0.06	0.11	0.09	0.03	0.03	0.05	0.03	0.01	0.01	0.17
PA-TU-8988T	Pancreas	Pancreas Adenocarcinoma	0.04	0.08	0.10	0.13	0.08	0.09	0.02	0.08	0.06	0.09	0.11	0.02	0.15	0.04	0.02	0.01	0.16	0.22
DAN-G	Pancreas	Pancreas Adenocarcinoma	0.03	0.09	0.09	0.11	0.08	0.08	0.02	0.07	0.07	0.11	0.09	0.02	0.06	0.03	0.02	0.01	0.04	0.22
PANC-02-03	Pancreas	Pancreas Adenocarcinoma	0.59	0.18	0.12	0.11	0.09	0.10	0.04	0.08	0.07	0.11	0.11	0.03	0.04	0.05	0.04	0.01	0.03	0.17
PA-TU-8902	Pancreas	Pancreas Adenocarcinoma	0.11	0.13	0.09	0.12	0.08	0.08	0.04	0.07	0.08	0.11	0.09	0.03	0.03	0.04	0.03	0.01	0.39	0.51
PANC-04-03	Pancreas	Pancreas Adenocarcinoma	0.12	0.14	0.09	0.13	0.08	0.09	0.05	0.08	0.06	0.14	0.10	0.02	0.04	0.04	0.03	0.01	0.05	0.16
KP-3	Pancreas	Pancreas Adenosquamous Carcinoma	0.03	0.15	0.07	0.10	0.08	0.08	0.05	0.07	0.06	0.09	0.10	0.03	0.14	0.06	0.05	0.01	0.21	0.22
HUP-T4	Pancreas	Pancreas Carcinoma	0.01	0.08	0.07	0.10	0.07	0.06	0.04	0.07	0.06	0.10	0.11	0.02	0.04	0.04	0.03	0.00	0.02	0.13
YAPC	Pancreas	Pancreas Carcinoma	0.01	0.08	0.09	0.11	0.09	0.07	0.02	0.07	0.06	0.10	0.09	0.03	0.04	0.04	0.02	0.01	0.03	0.13
PL4	Pancreas	Pancreas Carcinoma	0.01	0.10	0.07	0.09	0.06	0.08	0.02	0.05	0.04	0.08	0.07	0.01	0.02	0.03	0.01	0.01	0.01	0.11
KP-1N	Pancreas	Pancreas Carcinoma	0.01	0.07	0.10	0.11	0.08	0.09	0.02	0.08	0.07	0.10	0.09	0.03	0.18	0.03	0.02	0.01	0.15	0.21
BXPC-3	Pancreas	Pancreas Ductal Carcinoma	0.00	0.05	0.08	0.09	0.04	0.05	0.01	0.05	0.04	0.06	0.08	0.02	0.04	0.02	0.02	0.01	0.02	0.15
SW1990	Pancreas	Pancreas Ductal Carcinoma	0.00	0.11	0.08	0.09	0.06	0.07	0.04	0.06	0.05	0.11	0.10	0.02	0.04	0.07	0.04	0.01	0.02	0.07
HPAF-II	Pancreas	Pancreas Ductal Carcinoma	0.08	0.14	0.09	0.11	0.08	0.09	0.05	0.10	0.06	0.10	0.12	0.03	0.04	0.05	0.04	0.01	0.02	0.17
PANC-10-05	Pancreas	Pancreas Ductal Carcinoma	0.06	0.11	0.07	0.10	0.06	0.07	0.05	0.06	0.05	0.11	0.08	0.02	0.05	0.04	0.03	0.02	0.06	0.12
PSN1	Pancreas	Pancreas Ductal Carcinoma	0.00	0.11	0.07	0.10	0.06	0.06	0.03	0.05	0.05	0.10	0.08	0.02	0.06	0.06	0.04	0.02	0.02	0.14
PANC-08-13	Pancreas	Pancreas Ductal Carcinoma	0.02	0.14	0.09	0.12	0.07	0.08	0.04	0.07	0.07	0.11	0.09	0.03	0.06	0.05	0.04	0.02	0.02	0.18
ASPC-1	Pancreas	Pancreas Ductal Carcinoma	0.06	0.12	0.08	0.12	0.06	0.08	0.05	0.06	0.05	0.14	0.09	0.03	0.06	0.08	0.04	0.01	0.04	0.20
CAPAN-2	Pancreas	Pancreas Ductal Carcinoma	0.03	0.15	0.08	0.11	0.09	0.08	0.06	0.08	0.06	0.12	0.10	0.02	0.03	0.06	0.03	0.01	0.04	0.17
KP-4	Pancreas	Pancreas Ductal Carcinoma	0.03	0.12	0.08	0.11	0.07	0.08	0.03	0.06	0.06	0.10	0.08	0.02	0.23	0.05	0.03	0.01	0.24	0.26
MIA-PACA-2	Pancreas	Pancreas Ductal Carcinoma	0.32	0.12	0.08	0.12	0.06	0.08	0.04	0.05	0.13	0.12	0.11	0.03	0.28	0.06	0.05	0.01	0.30	0.37
PANC-03-27	Pancreas	Pancreas Ductal Carcinoma	0.01	0.05	0.08	0.10	0.06	0.07	0.03	0.07	0.05	0.09	0.08	0.02	0.03	0.04	0.03	0.01	0.05	0.14
CAPAN-1	Pancreas	Pancreas Ductal Carcinoma	0.03	0.06	0.07	0.10	0.06	0.07	0.05	0.06	0.05	0.10	0.09	0.02	0.04	0.03	0.02	0.00	0.01	0.13
CFPAC-1	Pancreas	Pancreas Ductal Carcinoma	0.05	0.09	0.08	0.08	0.06	0.08	0.03	0.07	0.06	0.09	0.08	0.01	0.03	0.04	0.03	0.00	0.02	0.13
HUP-T3	Pancreas	Pancreas Ductal Carcinoma	0.00	0.11	0.08	0.10	0.07	0.07	0.04	0.05	0.05	0.08	0.09	0.02	0.03	0.04	0.03	0.00	0.10	0.14
MZ1-PC	Pancreas	Pancreas Ductal Carcinoma	0.01	0.11	0.10	0.11	0.07	0.09	0.03	0.08	0.05	0.10	0.09	0.02	0.04	0.05	0.03	0.01	0.03	0.24
SUIT-2	Pancreas	Pancreas Tubular Adenocarcinoma	0.23	0.15	0.08	0.11	0.07	0.08	0.05	0.07	0.06	0.10	0.08	0.02	0.07	0.07	0.04	0.01	0.15	0.20
JAR	Placenta	Choriocarcinoma	0.01	0.12	0.09	0.12	0.08	0.08	0.04	0.09	0.07	0.12	0.11	0.03	0.28	0.06	0.11	0.01	0.23	0.27
JEG-3	Placenta	Choriocarcinoma	0.00	0.12	0.07	0.09	0.09	0.08	0.03	0.08	0.04	0.06	0.08	0.01	0.30	0.05	0.09	0.01	0.20	0.20
IST-MES1	Pleura	Mesothelioma	0.01	0.09	0.08	0.08	0.07	0.07	0.02	0.06	0.06	0.11	0.07	0.01	0.18	0.03	0.03	0.01	0.30	0.31
NCI-H2452	Pleura	Pleural Mesothelioma	0.01	0.08	0.07	0.09	0.07	0.07	0.03	0.05	0.05	0.09	0.09	0.02	0.03	0.03	0.03	0.01	0.01	0.13
MPP-89	Pleura	Pleural Mesothelioma	0.04	0.08	0.08	0.10	0.06	0.06	0.03	0.06	0.06	0.08	0.09	0.02	0.06	0.04	0.01	0.01	0.24	0.23
H513	Pleura	Pleural Mesothelioma	0.00	0.08	0.08	0.09	0.06	0.08	0.04	0.08	0.07	0.12	0.09	0.01	0.04	0.03	0.01	0.00	0.20	0.22
H2869	Pleura	Pleural Mesothelioma	0.00	0.08	0.08	0.08	0.06	0.07	0.02	0.06	0.04	0.09	0.08	0.01	0.12	0.04	0.01	0.01	0.09	0.14
MSTO-211H	Pleura	Pleural Mesothelioma	0.43	0.16	0.18	0.14	0.07	0.08	0.03	0.06	0.05	0.09	0.09	0.02	0.30	0.02	0.03	0.01	0.08	0.23
H2591	Pleura	Pleural Mesothelioma	0.83	0.15	0.07	0.10	0.07	0.06	0.04	0.06	0.05	0.09	0.07	0.03	0.28	0.04	0.04	0.01	0.29	0.33
H290	Pleura	Pleural Mesothelioma	0.43	0.45	0.41	0.35	0.07	0.07	0.02	0.06	0.04	0.10	0.08	0.02	0.29	0.03	0.03	0.01	0.25	0.24
H2804	Pleura	Pleural Mesothelioma	0.03	0.10	0.07	0.10	0.08	0.08	0.02	0.06	0.06	0.09	0.08	0.02	0.04	0.03	0.02	0.01	0.04	0.17
H2810	Pleura	Pleural Mesothelioma	0.00	0.13	0.09	0.12	0.08	0.08	0.04	0.08	0.09	0.12	0.10	0.02	0.10	0.06	0.07	0.01	0.18	0.23
H2595	Pleura	Pleural Mesothelioma	0.01	0.15	0.09	0.10	0.06	0.08	0.04	0.07	0.06	0.10	0.09	0.03	0.16	0.05	0.05	0.00	0.25	0.28
H2722	Pleura	Pleural Mesothelioma	0.22	0.10	0.07	0.10	0.05	0.06	0.01	0.04	0.04	0.08	0.07	0.01	0.03	0.04	0.01	0.00	0.09	0.18
H2731	Pleura	Pleural Mesothelioma	0.14	0.10	0.07	0.11	0.05	0.06	0.05	0.04	0.05	0.08	0.07	0.02	0.24	0.04	0.03	0.00	0.26	0.30
H2818	Pleura	Pleural Mesothelioma	0.45	0.10	0.06	0.09	0.05	0.07	0.03	0.05	0.04	0.06	0.07	0.01	0.02	0.03	0.02	0.01	0.03	0.14
H2373	Pleura	Pleural Mesothelioma	0.03	0.09	0.07	0.08	0.06	0.06	0.03	0.05	0.05	0.08	0.07	0.02	0.03	0.03	0.02	0.01	0.11	0.37
H2795	Pleura	Pleural Mesothelioma	0.08	0.10	0.08	0.10	0.06	0.07	0.03	0.05	0.05	0.09	0.08	0.02	0.07	0.04	0.03	0.01	0.10	0.45
H2803	Pleura	Pleural Mesothelioma	0.01	0.10	0.08	0.11	0.08	0.07	0.03	0.06	0.05	0.09	0.10	0.02	0.03	0.03	0.02	0.00	0.03	0.10
NCI-H28	Pleura	Pleural Mesothelioma	0.00	0.10	0.09	0.09	0.06	0.07	0.05	0.05	0.05	0.10	0.08	0.03	0.22	0.05	0.12	0.02	0.32	0.43
H2369	Pleura	Pleural Mesothelioma	0.00	0.09	0.08	0.08	0.05	0.05	0.02	0.04	0.03	0.07	0.06	0.01	0.02	0.03	0.00	0.00	0.01	0.15
H2461	Pleura	Pleural Mesothelioma	0.00	0.10	0.07	0.10	0.06	0.06	0.03	0.04	0.04	0.07	0.07	0.02	0.24	0.04	0.03	0.00	0.31	0.42
NCI-H2052	Pleura	Pleural Mesothelioma	0.01	0.10	0.05	0.10	0.05	0.06	0.03	0.04	0.04	0.07	0.07	0.00	0.02	0.03	0.02	0.01	0.04	0.09
RWPE2-W99	Prostate	Carcinoma	0.00	0.11	0.07	0.09	0.06	0.06	0.02	0.05	0.04	0.09	0.08	0.02	0.03	0.04	0.02	0.00	0.03	0.16
VCAP	Prostate	Hormone Refractory Prostate Cancer	0.02	0.09	0.08	0.10	0.07	0.06	0.04	0.07	0.05	0.12	0.12	0.04	0.28	0.06	0.03	0.01	0.29	0.24
PC-3	Prostate	Prostate Adenocarcinoma	0.18	0.08	0.06	0.09	0.05	0.06	0.02	0.05	0.04	0.08	0.06	0.01	0.15	0.01	0.03	0.00	0.14	0.35
LNCAP-CLONE-FGC	Prostate	Prostate Adenocarcinoma	0.01	0.11	0.07	0.08	0.06	0.07	0.02	0.07	0.04	0.08	0.08	0.02	0.06	0.04	0.02	0.02	0.02	0.13
22RV1	Prostate	Prostate Adenocarcinoma	0.00	0.13	0.08	0.10	0.07	0.07	0.03	0.08	0.06	0.10	0.09	0.02	0.30	0.04	0.08	0.00	0.22	0.21
DU-145	Prostate	Prostate Carcinoma	0.07	0.09	0.06	0.09	0.04	0.05	0.02	0.04	0.04	0.08	0.06	0.01	0.02	0.03	0.02	0.01	0.01	0.11
PWR-1E	Prostate	Prostate Carcinoma	0.01	0.16	0.09	0.12	0.09	0.09	0.03	0.09	0.06	0.11	0.10	0.03	0.35	0.05	0.06	0.02	0.28	0.22
RWPE-1	Prostate	Prostate Carcinoma	0.03	0.14	0.10	0.11	0.08	0.10	0.03	0.09	0.06	0.11	0.11	0.02	0.07	0.05	0.04	0.00	0.06	0.15
BPH-1	Prostate	Prostate Hyperplasia	0.01	0.09	0.09	0.10	0.08	0.07	0.03	0.07	0.05	0.11	0.10	0.03	0.19	0.06	0.03	0.01	0.07	0.17
NCI-H660	Prostate	Prostate Small Cell Carcinoma	0.05	0.13	0.09	0.09	0.07	0.09	0.04	0.08	0.06	0.10	0.11	0.02	0.03	0.05	0.04	0.01	0.03	0.16
A253	Salivary Gland	Mucoepidermoid Carcinoma	0.02	0.08	0.06	0.10	0.07	0.07	0.04	0.07	0.07	0.07	0.07	0.03	0.29	0.03	0.07	0.01	0.31	0.50
H3118	Salivary Gland	Salivary Gland (Parotid) Mucoepidermoid Carcinoma	0.04	0.07	0.09	0.12	0.08	0.09	0.04	0.08	0.07	0.11	0.10	0.02	0.08	0.04	0.03	0.02	0.26	0.24
A388	Skin	Carcinoma	0.19	0.14	0.08	0.08	0.07	0.08	0.05	0.08	0.07	0.11	0.13	0.03	0.31	0.06	0.07	0.01	0.36	0.44
A4-FUK	Skin	Diffuse Large B Cell Lymphoma	0.13	0.09	0.08	0.10	0.08	0.08	0.06	0.06	0.10	0.11	0.12	0.03	0.32	0.03	0.04	0.02	0.23	0.27
IGR-37	Skin	Malignant Melanoma	0.01	0.08	0.07	0.08	0.04	0.05	0.01	0.04	0.04	0.06	0.08	0.01	0.26	0.04	0.03	0.00	0.18	0.15
WM793B	Skin	Malignant Melanoma	0.00	0.13	0.06	0.10	0.05	0.06	0.02	0.05	0.04	0.08	0.09	0.02	0.36	0.04	0.04	0.00	0.31	0.18
IGR39	Skin	Malignant Melanoma	0.01	0.14	0.08	0.10	0.04	0.07	0.02	0.07	0.05	0.09	0.08	0.01	0.28	0.04	0.04	0.01	0.29	0.18
C32	Skin	Malignant Melanoma	0.00	0.16	0.09	0.09	0.07	0.07	0.04	0.06	0.07	0.09	0.12	0.04	0.30	0.07	0.04	0.01	0.30	0.19
UACC-257	Skin	Malignant Melanoma	0.01	0.09	0.07	0.08	0.05	0.06	0.02	0.05	0.04	0.07	0.09	0.01	0.10	0.01	0.02	0.01	0.06	0.12
MALME-3M	Skin	Malignant Melanoma	0.00	0.07	0.06	0.08	0.05	0.05	0.01	0.05	0.05	0.07	0.08	0.02	0.08	0.03	0.03	0.02	0.02	0.11
UACC-62	Skin	Malignant Melanoma	0.00	0.07	0.07	0.08	0.05	0.05	0.02	0.05	0.04	0.08	0.07	0.02	0.29	0.02	0.04	0.02	0.29	0.18
SK-MEL-28	Skin	Malignant Melanoma	0.00	0.06	0.05	0.10	0.06	0.05	0.01	0.05	0.04	0.08	0.07	0.01	0.18	0.02	0.02	0.00	0.21	0.17
MDA-MB-435	Skin	Malignant Melanoma	0.00	0.07	0.07	0.08	0.05	0.04	0.01	0.05	0.04	0.06	0.06	0.02	0.27	0.02	0.02	0.00	0.16	0.22
LOXIMVI	Skin	Malignant Melanoma	0.04	0.09	0.07	0.08	0.05	0.06	0.02	0.05	0.07	0.10	0.07	0.02	0.31	0.03	0.02	0.01	0.34	0.36
SK-MEL-5	Skin	Malignant Melanoma	0.02	0.08	0.08	0.11	0.06	0.06	0.02	0.05	0.16	0.11	0.09	0.02	0.25	0.04	0.03	0.01	0.09	0.15
MZ2-MEL	Skin	Malignant Melanoma	0.00	0.11	0.07	0.10	0.08	0.07	0.03	0.06	0.04	0.10	0.14	0.02	0.29	0.05	0.04	0.01	0.29	0.35
COLO-829	Skin	Malignant Melanoma	0.01	0.10	0.07	0.10	0.06	0.07	0.04	0.05	0.07	0.09	0.10	0.02	0.26	0.04	0.04	0.01	0.22	0.21
COLO-792	Skin	Malignant Melanoma	0.03	0.10	0.08	0.10	0.06	0.08	0.05	0.05	0.05	0.09	0.08	0.02	0.32	0.05	0.04	0.01	0.37	0.40
IGR-1	Skin	Malignant Melanoma	0.02	0.10	0.09	0.12	0.07	0.08	0.02	0.06	0.06	0.11	0.12	0.02	0.31	0.05	0.04	0.01	0.19	0.19
SK-MEL-1	Skin	Malignant Melanoma	0.00	0.09	0.07	0.09	0.07	0.07	0.03	0.07	0.05	0.10	0.10	0.03	0.30	0.04	0.04	0.00	0.23	0.23
SK-MEL-24	Skin	Malignant Melanoma	0.08	0.10	0.07	0.10	0.06	0.08	0.03	0.05	0.05	0.07	0.11	0.03	0.27	0.06	0.05	0.00	0.12	0.21
WM1552C	Skin	Malignant Melanoma	0.03	0.08	0.09	0.10	0.07	0.07	0.03	0.05	0.05	0.12	0.14	0.02	0.22	0.04	0.03	0.01	0.19	0.20

		TargetID	cg21539223	cg16456337	cg15956794	cg14773728	cg17573603	cg17919331	cg23965551	cg00522636	cg13518195	cg26459500	cg01092811	cg06363041	cg25437259	cg14506646	cg14861803	cg11162249	cg17789800	cg00261665
		UCSC_REFGENE_NAME	DCP2								NUDT16									
		UCSC_REFGENE_GROUP	TSS1500	TSS1500	TSS200	TSS200	TSS200	TSS200	1stExon;5'UTR	1stExon	TSS1500	TSS1500	TSS1500	TSS200	TSS200	TSS200	TSS200	5'UTR;1stExon	Body	Body
Cell Line	Tissue	Cell Line Type																		
WM35	Skin	Malignant Melanoma	0.01	0.09	0.09	0.10	0.06	0.07	0.03	0.08	0.06	0.10	0.12	0.02	0.28	0.04	0.05	0.01	0.27	0.22
G-361	Skin	Malignant Melanoma	0.01	0.07	0.06	0.11	0.08	0.07	0.04	0.08	0.06	0.11	0.17	0.03	0.26	0.05	0.04	0.01	0.14	0.21
LB373-MEL-D	Skin	Malignant Melanoma	0.01	0.10	0.07	0.10	0.07	0.06	0.04	0.07	0.05	0.11	0.14	0.03	0.27	0.07	0.03	0.01	0.22	0.19
SK-MEL-30	Skin	Malignant Melanoma	0.02	0.10	0.09	0.10	0.07	0.07	0.03	0.06	0.06	0.11	0.09	0.02	0.31	0.04	0.03	0.01	0.26	0.23
LB2518-MEL	Skin	Malignant Melanoma	0.00	0.11	0.08	0.11	0.07	0.06	0.03	0.06	0.06	0.09	0.10	0.02	0.28	0.04	0.04	0.01	0.25	0.25
HS-944-T	Skin	Malignant Melanoma	0.02	0.10	0.08	0.12	0.07	0.09	0.04	0.07	0.05	0.11	0.13	0.02	0.28	0.04	0.04	0.02	0.22	0.19
G-MEL	Skin	Malignant Melanoma	0.00	0.09	0.08	0.09	0.06	0.07	0.03	0.06	0.04	0.08	0.12	0.02	0.07	0.11	0.03	0.01	0.05	0.17
RPMI-7951	Skin	Malignant Melanoma	0.02	0.10	0.08	0.10	0.07	0.08	0.04	0.09	0.06	0.12	0.14	0.02	0.14	0.06	0.03	0.01	0.10	0.18
CP50-MEL-B	Skin	Malignant Melanoma	0.02	0.12	0.08	0.09	0.08	0.09	0.02	0.05	0.06	0.09	0.12	0.02	0.31	0.07	0.04	0.01	0.27	0.19
CP66-MEL	Skin	Malignant Melanoma	0.24	0.09	0.07	0.09	0.05	0.06	0.02	0.05	0.04	0.07	0.07	0.01	0.29	0.03	0.02	0.01	0.28	0.22
MZ7-MEL	Skin	Malignant Melanoma	0.00	0.09	0.06	0.09	0.06	0.07	0.03	0.06	0.04	0.07	0.09	0.01	0.28	0.03	0.02	0.01	0.21	0.21
WM278	Skin	Malignant Melanoma	0.02	0.08	0.06	0.08	0.06	0.08	0.02	0.06	0.04	0.07	0.12	0.02	0.24	0.04	0.03	0.02	0.23	0.21
RVH-421	Skin	Malignant Melanoma	0.00	0.08	0.06	0.08	0.05	0.07	0.01	0.04	0.04	0.08	0.07	0.01	0.31	0.01	0.02	0.00	0.27	0.17
SK-MEL-3	Skin	Malignant Melanoma	0.01	0.10	0.07	0.10	0.07	0.07	0.03	0.05	0.06	0.09	0.09	0.02	0.29	0.04	0.02	0.01	0.28	0.19
CHL-1	Skin	Malignant Melanoma	0.02	0.10	0.07	0.10	0.06	0.06	0.04	0.05	0.05	0.11	0.08	0.02	0.32	0.05	0.02	0.00	0.25	0.25
COLO-679	Skin	Malignant Melanoma	0.02	0.10	0.08	0.11	0.06	0.07	0.05	0.04	0.04	0.13	0.10	0.02	0.29	0.04	0.03	0.00	0.28	0.23
IST-MEL1	Skin	Malignant Melanoma	0.05	0.12	0.07	0.09	0.07	0.07	0.02	0.06	0.05	0.11	0.12	0.02	0.27	0.04	0.03	0.01	0.23	0.43
MEL-HO	Skin	Malignant Melanoma	0.01	0.10	0.08	0.08	0.07	0.08	0.03	0.06	0.04	0.08	0.10	0.01	0.26	0.04	0.05	0.00	0.22	0.22
COLO-800	Skin	Malignant Melanoma	0.06	0.10	0.06	0.09	0.07	0.08	0.04	0.05	0.27	0.17	0.15	0.04	0.34	0.17	0.26	0.18	0.25	0.26
CP67-MEL	Skin	Malignant Melanoma	0.03	0.11	0.09	0.10	0.07	0.06	0.06	0.05	0.04	0.11	0.07	0.03	0.31	0.04	0.04	0.01	0.12	0.25
MEL-JUSO	Skin	Malignant Melanoma	0.02	0.11	0.08	0.12	0.07	0.07	0.05	0.07	0.05	0.11	0.09	0.02	0.27	0.05	0.04	0.02	0.27	0.23
MEWO	Skin	Malignant Melanoma	0.00	0.11	0.07	0.09	0.06	0.07	0.02	0.05	0.04	0.09	0.08	0.01	0.10	0.04	0.03	0.01	0.03	0.20
A375	Skin	Malignant Melanoma	0.01	0.10	0.08	0.10	0.08	0.08	0.05	0.08	0.06	0.11	0.14	0.03	0.27	0.05	0.04	0.02	0.18	0.19
HMV-II	Skin	Malignant Melanoma	0.01	0.09	0.07	0.11	0.07	0.05	0.06	0.05	0.06	0.11	0.13	0.01	0.30	0.07	0.03	0.01	0.26	0.21
HT-144	Skin	Malignant Melanoma	0.02	0.08	0.07	0.09	0.07	0.08	0.02	0.06	0.06	0.11	0.10	0.03	0.31	0.05	0.03	0.01	0.11	0.17
A101D	Skin	Malignant Melanoma	0.00	0.10	0.08	0.09	0.07	0.06	0.02	0.04	0.06	0.07	0.07	0.01	0.28	0.03	0.03	0.00	0.25	0.26
A2058	Skin	Malignant Melanoma	0.00	0.09	0.07	0.09	0.05	0.04	0.02	0.05	0.04	0.09	0.06	0.02	0.29	0.03	0.02	0.00	0.25	0.20
GAK	Skin	Malignant Melanoma	0.00	0.10	0.08	0.09	0.07	0.06	0.03	0.05	0.04	0.10	0.07	0.02	0.30	0.03	0.03	0.01	0.14	0.20
MMAC-SF	Skin	Malignant Melanoma	0.02	0.10	0.07	0.09	0.07	0.07	0.02	0.06	0.05	0.10	0.07	0.03	0.30	0.03	0.02	0.00	0.20	0.19
SH-4	Skin	Malignant Melanoma	0.01	0.12	0.08	0.09	0.08	0.06	0.02	0.04	0.04	0.08	0.07	0.01	0.31	0.04	0.02	0.01	0.19	0.18
IPC-298	Skin	Malignant Melanoma	0.02	0.13	0.08	0.12	0.08	0.08	0.04	0.08	0.06	0.12	0.13	0.02	0.29	0.05	0.10	0.01	0.22	0.23
451LU	Skin	Malignant Melanoma	0.00	0.09	0.09	0.10	0.06	0.06	0.05	0.05	0.04	0.11	0.10	0.02	0.30	0.05	0.03	0.01	0.15	0.16
WM-115	Skin	Malignant Melanoma	0.01	0.11	0.09	0.11	0.08	0.09	0.03	0.08	0.05	0.10	0.09	0.03	0.28	0.04	0.07	0.01	0.17	0.22
SK-MEL-2	Skin	Malignant Melanoma	0.01	0.12	0.10	0.12	0.08	0.10	0.04	0.09	0.06	0.10	0.09	0.03	0.18	0.04	0.06	0.00	0.13	0.19
SK-MEL-31	Skin	Malignant Melanoma	0.01	0.14	0.10	0.12	0.09	0.10	0.04	0.07	0.06	0.12	0.09	0.03	0.20	0.05	0.06	0.01	0.09	0.18
M14	Skin	Malignant Melanoma	0.00	0.12	0.10	0.12	0.09	0.08	0.03	0.10	0.07	0.12	0.10	0.02	0.24	0.06	0.07	0.01	0.04	0.16
WM-266-4	Skin	Malignant Melanoma	0.00	0.10	0.10	0.10	0.09	0.08	0.02	0.07	0.08	0.11	0.11	0.03	0.29	0.03	0.07	0.00	0.04	0.23
HS-940-T	Skin	Malignant Melanoma	0.02	0.13	0.09	0.10	0.09	0.09	0.03	0.10	0.07	0.12	0.12	0.04	0.25	0.06	0.07	0.01	0.19	0.22
Hs-939-T	Skin	Malignant Melanoma	0.01	0.08	0.06	0.09	0.07	0.06	0.03	0.05	0.04	0.09	0.07	0.02	0.18	0.03	0.02	0.01	0.08	0.17
DJM-1	Skin	Malignant Trichilemmal Cyst	0.10	0.10	0.07	0.09	0.06	0.06	0.02	0.05	0.03	0.08	0.07	0.01	0.28	0.06	0.03	0.01	0.28	0.31
A431	Skin	Skin Squamous Cell Carcinoma	0.01	0.12	0.08	0.10	0.08	0.08	0.03	0.09	0.08	0.08	0.11	0.02	0.10	0.04	0.04	0.01	0.17	0.26
HUTU-80	Small Intestine	Duodenum Adenocarcinoma	0.01	0.13	0.10	0.11	0.07	0.08	0.04	0.07	0.16	0.12	0.11	0.05	0.35	0.07	0.08	0.02	0.38	0.48
RD	Soft Tissue	Bladder Carcinoma	0.04	0.10	0.09	0.11	0.07	0.08	0.04	0.06	0.07	0.11	0.08	0.03	0.31	0.05	0.03	0.01	0.30	0.28
A673	Soft Tissue	Ewing'S Sarcoma-Peripheral Primitive Neuroectodermal Tumour	0.08	0.11	0.09	0.10	0.06	0.07	0.05	0.06	0.71	0.43	0.28	0.17	0.38	0.28	0.16	0.19	0.47	0.42
SW684	Soft Tissue	Fibrosarcoma	0.01	0.11	0.08	0.12	0.07	0.08	0.07	0.06	0.06	0.13	0.10	0.04	0.28	0.05	0.08	0.00	0.24	0.24
HT-1080	Soft Tissue	Fibrosarcoma	0.00	0.14	0.09	0.11	0.08	0.08	0.04	0.07	0.07	0.10	0.11	0.03	0.24	0.05	0.07	0.01	0.15	0.25
HS-633T	Soft Tissue	Fibrosarcoma	0.02	0.15	0.10	0.14	0.10	0.09	0.10	0.14	0.06	0.14	0.10	0.05	0.23	0.06	0.05	0.01	0.17	0.20
SKN	Soft Tissue	Leio Myoma	0.03	0.08	0.07	0.11	0.07	0.07	0.03	0.05	0.05	0.13	0.10	0.02	0.32	0.04	0.03	0.00	0.21	0.18
G-402	Soft Tissue	Leiomyoblastoma	0.01	0.08	0.09	0.13	0.07	0.07	0.03	0.07	0.05	0.09	0.09	0.02	0.29	0.04	0.03	0.01	0.29	0.26
SK-UT-1	Soft Tissue	Leiomyosarcoma	0.04	0.11	0.06	0.11	0.07	0.06	0.01	0.06	0.11	0.08	0.08	0.02	0.19	0.06	0.04	0.00	0.19	0.22
SW872	Soft Tissue	Liposarcoma	0.01	0.14	0.10	0.13	0.08	0.08	0.03	0.08	0.06	0.11	0.09	0.02	0.22	0.05	0.05	0.01	0.13	0.09
MFH-INO	Soft Tissue	Malignant Fibrous Histiocytoma-Pleomorphic Sarcoma	0.03	0.12	0.09	0.10	0.07	0.08	0.04	0.06	0.07	0.12	0.09	0.02	0.31	0.06	0.07	0.01	0.25	0.25
GCT	Soft Tissue	Malignant Fibrous Histiocytoma-Pleomorphic Sarcoma	0.04	0.12	0.11	0.13	0.08	0.09	0.03	0.08	0.06	0.12	0.10	0.04	0.18	0.06	0.05	0.01	0.21	0.23
RKN	Soft Tissue	Myoma Ovarii	0.04	0.13	0.09	0.11	0.08	0.09	0.03	0.08	0.06	0.11	0.10	0.02	0.31	0.06	0.09	0.01	0.25	0.23
RH-41	Soft Tissue	Rhabdomyosarcoma	0.20	0.09	0.12	0.12	0.07	0.06	0.04	0.06	0.51	0.55	0.51	0.44	0.59	0.60	0.51	0.54	0.46	0.56
KYM-1	Soft Tissue	Rhabdomyosarcoma	0.02	0.08	0.08	0.09	0.06	0.06	0.04	0.06	0.06	0.09	0.12	0.03	0.32	0.06	0.04	0.01	0.28	0.24
A204	Soft Tissue	Rhabdomyosarcoma	0.11	0.09	0.09	0.11	0.08	0.09	0.05	0.08	0.07	0.11	0.13	0.04	0.28	0.07	0.04	0.01	0.29	0.26
SJRH30	Soft Tissue	Rhabdomyosarcoma	0.01	0.09	0.08	0.09	0.07	0.07	0.04	0.06	0.06	0.11	0.08	0.03	0.29	0.03	0.04	0.01	0.21	0.24
TE-441-T	Soft Tissue	Rhabdomyosarcoma	0.00	0.08	0.07	0.10	0.06	0.07	0.05	0.06	0.05	0.12	0.11	0.03	0.30	0.04	0.04	0.01	0.18	0.21
RH-1	Soft Tissue	Rhabdomyosarcoma	0.01	0.10	0.06	0.08	0.07	0.06	0.04	0.06	0.87	0.75	0.60	0.38	0.91	0.93	0.95	1.00	0.92	0.93
RH-18	Soft Tissue	Rhabdomyosarcoma Embryonal	0.14	0.11	0.12	0.11	0.06	0.07	0.04	0.06	0.58	0.54	0.41	0.39	0.56	0.54	0.54	0.52	0.48	0.60
VA-ES-BJ	Soft Tissue	Sarcoma	0.01	0.11	0.07	0.11	0.07	0.08	0.04	0.07	0.07	0.11	0.09	0.02	0.17	0.06	0.04	0.01	0.19	0.28
SK-LMS-1	Soft Tissue	Sarcoma	0.31	0.19	0.09	0.11	0.07	0.07	0.05	0.08	0.09	0.12	0.11	0.03	0.17	0.05	0.05	0.01	0.35	0.59
S-117	Soft Tissue	Sarcoma	0.01	0.14	0.09	0.11	0.09	0.09	0.05	0.09	0.08	0.11	0.09	0.02	0.22	0.04	0.06	0.01	0.28	0.40
MES-SA	Soft Tissue	Sarcoma	0.02	0.14	0.09	0.12	0.07	0.10	0.05	0.07	0.08	0.13	0.14	0.04	0.34	0.06	0.07	0.01	0.32	0.24
SW982	Soft Tissue	Synovial Sarcoma	0.04	0.13	0.09	0.11	0.08	0.09	0.04	0.09	0.06	0.09	0.11	0.03	0.31	0.06	0.05	0.01	0.31	0.24
A3-KAW	Stomach	Diffuse Large B Cell Lymphoma	0.06	0.14	0.09	0.11	0.09	0.11	0.05	0.09	0.08	0.12	0.14	0.02	0.34	0.07	0.11	0.01	0.35	0.39
OCUM-1	Stomach	Signet Ring Adenocarcinoma	0.00	0.07	0.07	0.08	0.07	0.08	0.03	0.08	0.05	0.10	0.07	0.02	0.02	0.02	0.02	0.00	0.03	0.13
NUGC-4	Stomach	Signet Ring Adenocarcinoma	0.20	0.11	0.08	0.11	0.08	0.08	0.04	0.07	0.05	0.09	0.09	0.01	0.02	0.03	0.02	0.00	0.00	0.15
SNU-1	Stomach	Stomach Adenocarcinoma	0.00	0.11	0.06	0.09	0.06	0.08	0.03	0.06	0.05	0.08	0.07	0.02	0.28	0.04	0.04	0.00	0.23	0.21
SNU-5	Stomach	Stomach Adenocarcinoma	0.01	0.09	0.07	0.11	0.08	0.08	0.03	0.07	0.06	0.11	0.10	0.02	0.06	0.04	0.03	0.00	0.03	0.14
GCIY	Stomach	Stomach Adenocarcinoma	0.00	0.07	0.07	0.09	0.06	0.08	0.02	0.05	0.05	0.11	0.08	0.02	0.03	0.03	0.02	0.01	0.09	0.19
NUGC-3	Stomach	Stomach Adenocarcinoma	0.02	0.09	0.08	0.10	0.07	0.06	0.04	0.06	0.04	0.09	0.07	0.02	0.03	0.03	0.02	0.00	0.04	0.50
TGBC11TKB	Stomach	Stomach Adenocarcinoma	0.17	0.10	0.07	0.09	0.05	0.07	0.04	0.06	0.05	0.10	0.08	0.02	0.20	0.03	0.03	0.01	0.16	0.18
RF-48	Stomach	Stomach Adenocarcinoma	0.05	0.11	0.06	0.10	0.06	0.06	0.03	0.06	0.05	0.10	0.08	0.02	0.25	0.03	0.04	0.01	0.22	0.25
GT3TKB	Stomach	Stomach Adenocarcinoma	0.01	0.07	0.06	0.10	0.05	0.06	0.02	0.05	0.06	0.09	0.08	0.01	0.03	0.04	0.02	0.02	0.03	0.18
MKN1	Stomach	Stomach Adenocarcinoma	0.02	0.07	0.08	0.09	0.07	0.07	0.03	0.06	0.06	0.08	0.07	0.02	0.03	0.03	0.02	0.01	0.01	0.17
NCI-SNU-1	Stomach	Stomach Adenocarcinoma	0.00	0.07	0.06	0.09	0.05	0.06	0.03	0.05	0.05	0.09	0.08	0.01	0.29	0.03	0.02	0.01	0.27	0.17
RERF-GC-1B	Stomach	Stomach Adenocarcinoma	0.01	0.09	0.07	0.09	0.06	0.07	0.03	0.05	0.04	0.13	0.08	0.02	0.22	0.03	0.01	0.00	0.24	0.20
MKN28	Stomach	Stomach Adenocarcinoma	0.00	0.07	0.09	0.09	0.07	0.08	0.05	0.05	0.06	0.11	0.08	0.02	0.29	0.04	0.02	0.01	0.25	0.76
MKN45	Stomach	Stomach Adenocarcinoma	0.20	0.09	0.08	0.10	0.06	0.08	0.05	0.07	0.05	0.12	0.09	0.02	0.04	0.06	0.03	0.00	0.05	0.21
IM-95	Stomach	Stomach Adenocarcinoma	0.00	0.11	0.10	0.10	0.07	0.08	0.05	0.06	0.06	0.11	0.08	0.02	0.03	0.02	0.03	0.01	0.01	0.15
NCI-N87	Stomach	Stomach Adenocarcinoma	0.47	0.15	0.10	0.10	0.07	0.07	0.05	0.06	0.04	0.09	0.08	0.03	0.04	0.06	0.03	0.01	0.04	0.46
NCI-SNU-5	Stomach	Stomach Adenocarcinoma	0.00	0.10	0.07	0.10	0.07	0.06	0.03	0.06	0.04	0.14	0.07	0.02	0.09	0.04	0.03	0.01	0.08	0.13
FU97	Stomach	Stomach Adenocarcinoma	0.02	0.09	0.08	0.09	0.06	0.08	0.04	0.06	0.06	0.12	0.09	0.02	0.03	0.03	0.03	0.01	0.02	0.14
HGC-27	Stomach	Stomach Adenocarcinoma	0.02	0.10	0.08	0.11	0.09	0.07	0.06	0.07	0.13	0.11	0.08	0.03	0.29	0.04	0.04	0.01	0.25	0.19
KATOIII	Stomach	Stomach Adenocarcinoma	0.70	0.12	0.08	0.09	0.07	0.07	0.08	0.07	0.06	0.12	0.11	0.03	0.04	0.05	0.04	0.01	0.02	0.16
23132-87	Stomach	Stomach Adenocarcinoma	0.07	0.06	0.07	0.09	0.06	0.06	0.02	0.06	0.04	0.10	0.07	0.02	0.06	0.02	0.01	0.00	0.12	0.18
AGS	Stomach	Stomach Adenocarcinoma	0.02	0.06	0.05	0.10	0.07	0.07	0.04	0.05	0.05	0.08	0.08	0.01	0.25	0.03	0.03	0.00	0.35	0.58
NCI-SNU-16	Stomach	Stomach Adenocarcinoma	0.01	0.13	0.08	0.10	0.08	0.08	0.04	0.07	0.06	0.11	0.10	0.02	0.03	0.04	0.03	0.01	0.01	0.14
ECC12	Stomach	Stomach Adenocarcinoma (Small Cell)	0.01	0.10	0.08	0.10	0.07	0.07	0.02	0.06	0.04	0.08	0.06	0.03	0.25	0.04	0.03	0.01	0.26	0.49
ECC10	Stomach	Stomach Adenocarcinoma (Small Cell)	0.00	0.08	0.06	0.08	0.06	0.04	0.06	0.04	0.04	0.09	0.08	0.02	0.29	0.03	0.03	0.01	0.27	0.20
MKN7	Stomach	Stomach Adenocarcinoma (Tubular)	0.00	0.10	0.07	0.09	0.07	0.07	0.03	0.06	0.05	0.08	0.08	0.02	0.07	0.03	0.02	0.01	0.22	0.17
HS746T	Stomach	Stomach Carcinoma	0.07	0.11	0.09	0.11	0.08	0.07	0.05	0.07	0.06	0.12	0.09	0.03	0.11	0.03	0.03	0.01	0.03	0.14
HSC-39	Stomach	Stomach Carcinoma	0.05	0.12	0.07	0.09	0.06	0.07	0.05	0.05	0.05	0.11	0.08	0.01	0.03	0.05	0.03	0.01	0.01	0.06
SCH	Stomach	Stomach Choriocarcinoma	0.01	0.10	0.08	0.11	0.07	0.06	0.02	0.06	0.04	0.08	0.08	0.03	0.19	0.03	0.03	0.01	0.15	0.18
NTERA-S-CL-D1	Testis	Testis Embryonal Carcinoma	0.28	0.35	0.35	0.31	0.08	0.08	0.03	0.06	0.08	0.09	0.08	0.03	0.29	0.05	0.03	0.01	0.27	0.41
NEC8	Testis	Testis Embryonal Carcinoma	0.02	0.15	0.08	0.11	0.07	0.07	0.03	0.07	0.05	0.13	0.11	0.02	0.05	0.05	0.05	0.01	0.03	0.18
NCCIT	Testis	Testis Teratocarcinoma	0.00	0.10	0.07	0.09	0.05	0.07	0.03	0.06	0.04	0.11	0.09	0.03	0.29	0.04	0.04	0.01	0.26	0.22
CAL-62	Thyroid	Anaplastic Thyroid Carcinoma	0.05	0.15	0.09	0.10	0.08	0.08	0.04	0.08	0.07	0.10	0.10	0.02	0.04	0.05	0.04	0.01	0.03	0.16
KMH-2	Thyroid	Anaplastic Thyroid Carcinoma	0.56	0.16	0.10	0.11	0.09	0.08	0.06	0.08	0.31	0.41	0.39	0.07	0.54	0.61	0.57	0.57	0.52	0.60
BHT-101	Thyroid	Anaplastic Thyroid Carcinoma	0.04	0.14	0.09	0.12	0.08	0.08	0.04	0.08	0.06	0.12	0.10	0.03	0.10	0.04	0.05	0.01	0.02	0.15
ASH-3	Thyroid	Anaplastic Thyroid Carcinoma	0.03	0.06	0.07	0.10	0.06	0.07	0.02	0.06	0.05	0.07	0.08	0.01	0.31	0.02	0.02	0.00	0.29	0.28
TT2609-C02	Thyroid	Follicular Thyroid Carcinoma	0.02	0.14	0.10	0.11	0.09	0.10	0.05	0.08	0.07	0.10	0.10	0.03	0.04	0.07	0.04	0.01	0.03	0.21
ML-1	Thyroid	Follicular Thyroid Carcinoma	0.02	0.14	0.12	0.13	0.08	0.10	0.03	0.09	0.07	0.12	0.12	0.03	0.30	0.07	0.10	0.01	0.24	0.20
IHH-4	Thyroid	Papillary Thyroid Carcinoma	0.03	0.16	0.09	0.11	0.09	0.10	0.04	0.09	0.07	0.12	0.09	0.04	0.33	0.07	0.11	0.01	0.20	0.23
8505C	Thyroid	Thyroid Anaplastic Carcinoma	0.03	0.13	0.09	0.11	0.08	0.08	0.02	0.08	0.07	0.12	0.10	0.02	0.03	0.04	0.04	0.01	0.04	0.12

		TargetID	cg21539223	cg16456337	cg15956794	cg14773728	cg17573603	cg17919331	cg23965551	cg00522636	cg13518195	cg26459500	cg01092811	cg06363041	cg25437259	cg14506646	cg14861803	cg11162249	cg17789800	cg00261665
		UCSC_REFGENE_NAME	DCP2								NUDT16									
		UCSC_REFGENE_GROUP	TSS1500	TSS1500	TSS200	TSS200	TSS200	TSS200	1stExon;5'UTR	1stExon	TSS1500	TSS1500	TSS1500	TSS200	TSS200	TSS200	TSS200	5'UTR;1stExon	Body	Body
**Cell Line**	**Tissue**	**Cell Line Type**																		
8305C	Thyroid	Thyroid Anaplastic Carcinoma	0.03	0.12	0.09	0.11	0.07	0.11	0.03	0.07	0.07	0.12	0.11	0.04	0.07	0.05	0.03	0.01	0.03	0.19
HTC-C3	Thyroid	Thyroid Carcinoma	0.03	0.12	0.09	0.11	0.09	0.08	0.04	0.08	0.07	0.12	0.11	0.03	0.06	0.05	0.03	0.00	0.03	0.18
FTC-133	Thyroid	Thyroid Carcinoma (Follicular)	0.00	0.13	0.08	0.11	0.09	0.08	0.05	0.07	0.07	0.10	0.10	0.03	0.04	0.04	0.04	0.01	0.01	0.19
CGTH-W-1	Thyroid	Thyroid Carcinoma (Follicular)	0.01	0.13	0.06	0.10	0.08	0.08	0.06	0.06	0.06	0.10	0.09	0.02	0.29	0.05	0.09	0.01	0.29	0.28
K5	Thyroid	Thyroid Carcinoma (Follicular)	0.18	0.14	0.08	0.11	0.09	0.10	0.06	0.08	0.07	0.11	0.11	0.03	0.23	0.05	0.05	0.01	0.22	0.25
RO82-W-1	Thyroid	Thyroid Carcinoma (Follicular)	0.62	0.15	0.10	0.11	0.08	0.09	0.06	0.06	0.06	0.12	0.10	0.03	0.17	0.06	0.05	0.01	0.15	0.22
TT	Thyroid	Thyroid Carcinoma (Medullary)	0.00	0.14	0.10	0.10	0.08	0.08	0.04	0.07	0.05	0.10	0.09	0.02	0.03	0.03	0.04	0.00	0.02	0.17
BCPAP	Thyroid	Thyroid Carcinoma (Papillary)	0.01	0.14	0.11	0.10	0.08	0.09	0.04	0.08	0.08	0.11	0.09	0.03	0.03	0.05	0.04	0.01	0.03	0.16
BICR10	Upper Aerodigestive Tract	Buccal Mucosa Squamous Carcinoma	0.01	0.09	0.07	0.12	0.08	0.08	0.03	0.05	0.04	0.10	0.09	0.02	0.30	0.04	0.03	0.01	0.21	0.20
HSC-2	Upper Aerodigestive Tract	Head & Neck Squamous Cell Carcinoma	0.01	0.12	0.10	0.17	0.12	0.09	0.05	0.11	0.09	0.15	0.19	0.03	0.34	0.06	0.08	0.01	0.36	0.67
BB49-HNC	Upper Aerodigestive Tract	Head & Neck Squamous Cell Carcinoma	0.04	0.13	0.08	0.12	0.08	0.09	0.05	0.08	0.07	0.11	0.14	0.03	0.31	0.06	0.09	0.01	0.31	0.29
CA9-22	Upper Aerodigestive Tract	Head & Neck Squamous Cell Carcinoma	0.04	0.12	0.07	0.10	0.07	0.08	0.07	0.06	0.08	0.10	0.08	0.03	0.31	0.06	0.07	0.01	0.29	0.46
KOSC-2	Upper Aerodigestive Tract	Head & Neck Squamous Cell Carcinoma	0.40	0.23	0.08	0.10	0.08	0.07	0.05	0.07	0.06	0.09	0.10	0.02	0.30	0.06	0.10	0.01	0.27	0.30
LB771-HNC	Upper Aerodigestive Tract	Head & Neck Squamous Cell Carcinoma	0.01	0.09	0.06	0.09	0.07	0.06	0.04	0.07	0.05	0.10	0.07	0.03	0.29	0.05	0.08	0.01	0.29	0.27
JHU-022	Upper Aerodigestive Tract	Head & Neck Squamous Cell Carcinoma	0.01	0.08	0.10	0.11	0.08	0.08	0.03	0.08	0.06	0.11	0.10	0.03	0.23	0.03	0.04	0.01	0.26	0.28
BB30-HNC	Upper Aerodigestive Tract	Head & Neck Squamous Cell Carcinoma	0.09	0.05	0.06	0.08	0.06	0.07	0.03	0.06	0.07	0.09	0.08	0.02	0.28	0.03	0.03	0.01	0.31	0.24
CAL-33	Upper Aerodigestive Tract	Head & Neck Squamous Cell Carcinoma	0.24	0.13	0.08	0.11	0.08	0.08	0.06	0.07	0.06	0.09	0.10	0.02	0.30	0.06	0.10	0.01	0.32	0.47
HO-1-U-1	Upper Aerodigestive Tract	Head & Neck Squamous Cell Carcinoma	0.01	0.07	0.07	0.11	0.08	0.08	0.04	0.06	0.07	0.14	0.08	0.03	0.30	0.02	0.03	0.00	0.30	0.24
BHY	Upper Aerodigestive Tract	Head & Neck Squamous Cell Carcinoma	0.01	0.07	0.09	0.10	0.07	0.08	0.02	0.06	0.05	0.11	0.09	0.02	0.23	0.03	0.03	0.01	0.33	0.21
FADU	Upper Aerodigestive Tract	Hypopharynx Squamous Cell Carcinoma	0.27	0.17	0.08	0.11	0.09	0.08	0.10	0.07	0.07	0.09	0.10	0.02	0.26	0.07	0.08	0.01	0.25	0.48
BICR22	Upper Aerodigestive Tract	Metastasis From Lymph Node	0.06	0.07	0.09	0.10	0.07	0.07	0.04	0.07	0.06	0.10	0.08	0.02	0.28	0.03	0.02	0.00	0.24	0.22
KON	Upper Aerodigestive Tract	Mouth Squamous Cell Carcinoma	0.06	0.15	0.07	0.10	0.08	0.08	0.05	0.07	0.06	0.11	0.08	0.02	0.34	0.06	0.10	0.01	0.27	0.23
HN	Upper Aerodigestive Tract	Mouth Squamous Cell Carcinoma	0.00	0.12	0.08	0.10	0.08	0.08	0.05	0.08	0.06	0.09	0.09	0.03	0.03	0.05	0.05	0.01	0.06	0.30
HO-1-N-1	Upper Aerodigestive Tract	Mucoepidermoid Carcinoma	0.01	0.08	0.09	0.11	0.07	0.07	0.02	0.06	0.05	0.12	0.08	0.03	0.03	0.03	0.01	0.01	0.01	0.13
JHU-011	Upper Aerodigestive Tract	Ns	0.05	0.15	0.07	0.07	0.07	0.07	0.04	0.06	0.05	0.10	0.09	0.02	0.22	0.04	0.06	0.02	0.28	0.25
PCI-30	Upper Aerodigestive Tract	Ns	0.03	0.06	0.06	0.08	0.08	0.06	0.04	0.06	0.07	0.09	0.08	0.01	0.25	0.03	0.04	0.00	0.26	0.31
PCI-15A	Upper Aerodigestive Tract	Ns	0.06	0.14	0.10	0.10	0.09	0.08	0.05	0.08	0.06	0.12	0.09	0.03	0.19	0.05	0.07	0.01	0.29	0.30
PCI-6A	Upper Aerodigestive Tract	Ns	0.03	0.14	0.08	0.10	0.06	0.09	0.06	0.07	0.09	0.11	0.10	0.03	0.04	0.04	0.04	0.01	0.02	0.17
PCI-4B	Upper Aerodigestive Tract	Ns	0.05	0.14	0.11	0.11	0.08	0.09	0.04	0.08	0.07	0.10	0.10	0.03	0.03	0.07	0.04	0.01	0.23	0.21
BICR78	Upper Aerodigestive Tract	Oral Alveolus Squamous Carcinoma	0.02	0.14	0.08	0.09	0.08	0.07	0.05	0.07	0.06	0.11	0.09	0.03	0.33	0.06	0.09	0.01	0.28	0.30
SAT	Upper Aerodigestive Tract	Oral Squamous Cell Carcinoma	0.01	0.09	0.06	0.09	0.06	0.06	0.06	0.04	0.04	0.09	0.08	0.01	0.29	0.05	0.03	0.00	0.19	0.39
SKN-3	Upper Aerodigestive Tract	Oral Squamous Cell Carcinoma	0.00	0.07	0.09	0.09	0.08	0.08	0.00	0.08	0.07	0.09	0.10	0.01	0.02	0.04	0.01	0.00	0.03	0.13
PE-CA-PJ15	Upper Aerodigestive Tract	Oral Squamous Cell Carcinoma	0.07	0.15	0.09	0.10	0.07	0.08	0.05	0.08	0.06	0.11	0.09	0.04	0.29	0.07	0.08	0.01	0.34	0.56
DETROIT562	Upper Aerodigestive Tract	Pharynx Carcinoma	0.06	0.07	0.10	0.12	0.08	0.08	0.04	0.07	0.08	0.11	0.09	0.04	0.25	0.03	0.03	0.01	0.27	0.25
HCE-T	Upper Aerodigestive Tract	Sinus Squamous Cell Carcinoma	0.01	0.10	0.09	0.09	0.07	0.07	0.02	0.08	0.06	0.12	0.13	0.02	0.28	0.04	0.04	0.01	0.29	0.23
RPMI-2650	Upper Aerodigestive Tract	Squamous Cell Carcinoma	0.01	0.08	0.08	0.10	0.08	0.07	0.03	0.07	0.47	0.46	0.18	0.10	0.44	0.30	0.17	0.25	0.40	0.43
DOK	Upper Aerodigestive Tract	Tonge Dysplasia	0.05	0.08	0.08	0.12	0.08	0.08	0.03	0.06	0.06	0.11	0.08	0.04	0.08	0.04	0.02	0.00	0.12	0.22
CAL-27	Upper Aerodigestive Tract	Tongue Squamous Cell Carcinoma	0.32	0.11	0.08	0.10	0.07	0.08	0.05	0.07	0.07	0.12	0.14	0.03	0.15	0.08	0.03	0.02	0.30	0.26
OSC-19	Upper Aerodigestive Tract	Tongue Squamous Cell Carcinoma	0.04	0.13	0.10	0.13	0.08	0.10	0.05	0.09	0.44	0.53	0.30	0.05	0.53	0.63	0.59	0.57	0.45	0.72
HSC-4	Upper Aerodigestive Tract	Tongue Squamous Cell Carcinoma	0.02	0.12	0.07	0.08	0.07	0.07	0.03	0.07	0.05	0.08	0.09	0.02	0.30	0.05	0.10	0.01	0.22	0.18
SCC-9	Upper Aerodigestive Tract	Tongue Squamous Cell Carcinoma	0.02	0.14	0.07	0.09	0.07	0.08	0.03	0.07	0.06	0.09	0.09	0.03	0.07	0.05	0.03	0.01	0.05	0.15
SCC-4	Upper Aerodigestive Tract	Tongue Squamous Cell Carcinoma	0.01	0.13	0.08	0.10	0.07	0.08	0.06	0.09	0.05	0.11	0.10	0.01	0.23	0.05	0.08	0.01	0.23	0.22
SAS	Upper Aerodigestive Tract	Tongue Squamous Cell Carcinoma	0.61	0.09	0.10	0.11	0.08	0.09	0.05	0.07	0.14	0.12	0.10	0.04	0.20	0.03	0.03	0.01	0.33	0.29
OSC-20	Upper Aerodigestive Tract	Tongue Squamous Cell Carcinoma	0.06	0.08	0.10	0.12	0.08	0.09	0.04	0.09	0.07	0.12	0.14	0.02	0.04	0.04	0.02	0.02	0.05	0.21
HSC-3	Upper Aerodigestive Tract	Tongue Squamous Cell Carcinoma	0.15	0.08	0.09	0.11	0.08	0.09	0.04	0.08	0.08	0.09	0.09	0.02	0.10	0.04	0.03	0.01	0.29	0.36
SCC-25	Upper Aerodigestive Tract	Tongue Squamous Cell Carcinoma	0.02	0.15	0.08	0.11	0.08	0.09	0.03	0.08	0.06	0.11	0.12	0.03	0.07	0.07	0.05	0.01	0.22	0.61
SCC-15	Upper Aerodigestive Tract	Tongue Squamous Cell Carcinoma	0.00	0.07	0.09	0.11	0.07	0.08	0.03	0.06	0.06	0.10	0.09	0.01	0.23	0.04	0.02	0.01	0.29	0.55
LB831-BLC	Urinary Tract	Bladder Carcinoma	0.01	0.13	0.10	0.12	0.08	0.09	0.05	0.08	0.06	0.11	0.09	0.02	0.07	0.04	0.05	0.01	0.09	0.17
TCCSUP	Urinary Tract	Bladder Carcinoma	0.02	0.13	0.09	0.12	0.07	0.09	0.04	0.08	0.05	0.11	0.09	0.03	0.09	0.06	0.04	0.01	0.03	0.19
UM-UC-3	Urinary Tract	Bladder Carcinoma	0.07	0.12	0.08	0.11	0.08	0.10	0.07	0.08	0.06	0.11	0.10	0.02	0.26	0.05	0.08	0.01	0.26	0.25
5637	Urinary Tract	Bladder Carcinoma	0.02	0.12	0.09	0.10	0.08	0.06	0.05	0.08	0.06	0.11	0.09	0.03	0.24	0.05	0.07	0.01	0.31	0.36
DSH1	Urinary Tract	Bladder Carcinoma	0.03	0.14	0.09	0.12	0.09	0.08	0.06	0.07	0.07	0.11	0.09	0.03	0.19	0.07	0.08	0.01	0.24	0.23
SW780	Urinary Tract	Bladder Carcinoma	0.00	0.17	0.10	0.11	0.08	0.10	0.05	0.07	0.06	0.12	0.10	0.04	0.05	0.05	0.03	0.01	0.03	0.19
J82	Urinary Tract	Bladder Carcinoma	0.03	0.14	0.11	0.12	0.08	0.09	0.04	0.08	0.07	0.12	0.10	0.03	0.08	0.05	0.05	0.02	0.10	0.20
T-24	Urinary Tract	Bladder Carcinoma	0.05	0.16	0.10	0.11	0.08	0.10	0.05	0.09	0.08	0.10	0.11	0.04	0.03	0.05	0.04	0.02	0.09	0.38
RT4	Urinary Tract	Bladder Carcinoma	0.03	0.17	0.10	0.11	0.09	0.10	0.06	0.08	0.07	0.11	0.10	0.03	0.30	0.07	0.09	0.02	0.29	0.54
RT-112	Urinary Tract	Bladder Trasnitional Cell Carcinoma	0.04	0.14	0.07	0.09	0.08	0.10	0.07	0.07	0.06	0.12	0.10	0.03	0.32	0.06	0.10	0.01	0.33	0.31
SW1710	Urinary Tract	Bladder Trasnitional Cell Carcinoma	0.02	0.13	0.08	0.11	0.07	0.08	0.06	0.08	0.07	0.11	0.10	0.03	0.19	0.05	0.07	0.01	0.15	0.20
HT-1197	Urinary Tract	Bladder Trasnitional Cell Carcinoma	0.02	0.14	0.09	0.12	0.07	0.08	0.04	0.08	0.06	0.10	0.08	0.04	0.25	0.06	0.10	0.01	0.17	0.19
647-V	Urinary Tract	Bladder Trasnitional Cell Carcinoma	0.03	0.14	0.09	0.12	0.08	0.07	0.02	0.08	0.06	0.10	0.12	0.04	0.08	0.06	0.04	0.01	0.18	0.21
BFTC-905	Urinary Tract	Bladder Trasnitional Cell Carcinoma	0.02	0.13	0.08	0.11	0.08	0.08	0.04	0.07	0.06	0.14	0.12	0.03	0.05	0.05	0.05	0.01	0.07	0.19
KU-19-19	Urinary Tract	Bladder Trasnitional Cell Carcinoma	0.01	0.13	0.09	0.14	0.07	0.09	0.04	0.07	0.06	0.10	0.09	0.03	0.31	0.07	0.08	0.01	0.30	0.40
VM-CUB-1	Urinary Tract	Bladder Trasnitional Cell Carcinoma	0.12	0.15	0.08	0.10	0.08	0.07	0.05	0.07	0.05	0.10	0.09	0.02	0.03	0.07	0.03	0.01	0.19	0.26
HT-1376	Urinary Tract	Bladder Trasnitional Cell Carcinoma	0.04	0.15	0.09	0.11	0.08	0.09	0.04	0.08	0.07	0.11	0.11	0.05	0.04	0.03	0.04	0.01	0.03	0.17
SCABER	Urinary Tract	Squamous Cell Carcinoma	0.04	0.14	0.08	0.11	0.08	0.09	0.05	0.07	0.07	0.12	0.11	0.03	0.28	0.05	0.08	0.00	0.31	0.24
639-V	Urinary Tract	Ureter Transitional Cell Carcinoma	0.37	0.12	0.09	0.10	0.08	0.08	0.06	0.07	0.06	0.10	0.10	0.02	0.22	0.05	0.06	0.01	0.16	0.23
CAL-39	Vulva	Vulva Squamous Cell Carcinoma	0.01	0.12	0.08	0.10	0.07	0.07	0.06	0.06	0.05	0.10	0.08	0.02	0.04	0.03	0.02	0.01	0.04	0.20
SW954	Vulva	Vulva Squamous Cell Carcinoma	0.01	0.10	0.08	0.10	0.08	0.09	0.04	0.08	0.06	0.14	0.15	0.03	0.10	0.05	0.04	0.03	0.19	0.32
SW962	Vulva	Vulva Squamous Cell Carcinoma	0.02	0.09	0.08	0.09	0.07	0.06	0.03	0.06	0.06	0.11	0.12	0.02	0.12	0.06	0.03	0.01	0.08	0.22
