# Supplementary material for: Epigenetic loss of the RNA decapping enzyme NUDT16 mediates C-MYC activation in T-cell acute lymphoblastic leukemia
Source: Leukemia. 2017 Apr 11;31(7):1622–5. doi: 10.1038/leu.2017.99 (PMC5501321; doi:10.1038/leu.2017.99)
Supplement: Supplementary Table S2 [file leu201799x11.docx]

| **Supplementary Table 2.** Clinical characteristics of the studied T-ALL cohort according to NUDT16 methylation status | | | | | | | | | | |
| --- | --- | --- | --- | --- | --- | --- | --- | --- | --- | --- |
| **N = 51** |  |  |  | NUDT16 | | | | |  | *P-value |
|  | N | % |  | Unmethylated (U) | |  | Methylated (M) | |  |  |
|  |  |  |  | **N** | **%** |  | **N** | **%** |  |  |
| **Age** |  |  |  |  |  |  |  |  |  |  |
| Childhood (< 16) | 8 | 16% |  | 3 | 37% |  | 5 | 63% |  | 0.31 |
| AYA (16 – 29) | 22 | 43% |  | 9 | 41% |  | 13 | 59% |  |  |
| Adult (> 29) | 20 | 39% |  | 7 | 35% |  | 13 | 65% |  |  |
| Unknown | 1 | 2% |  | 1 | 100% |  | 0 | 0% |  |  |
| **Gender** |  |  |  |  |  |  |  |  |  |  |
| Male | 40 | 78% |  | 16 | 40% |  | 24 | 60% |  | 0.55 |
| Female | 11 | 22% |  | 4 | 36% |  | 7 | 64% |  |  |
| **Diagnostic** |  |  |  |  |  |  |  |  |  |  |
| T-ALL | 49 | 96% |  | 19 | 39% |  | 30 | 61% |  | 0.63 |
| Relapsed T-ALL | 2 | 4% |  | 1 | 50% |  | 1 | 50% |  |  |
| **Cytogenetics** |  |  |  |  |  |  |  |  |  |  |
| Normal | 13 | 26% |  | 6 | 46% |  | 7 | 54% |  | 0.31 |
| Abnornal | 16 | 31% |  | 8 | 50% |  | 8 | 50% |  |  |
| Failed/Missing | 22 | 43% |  | 6 | 27% |  | 16 | 73% |  |  |
| **NOTCH1 mutations** |  |  |  |  |  |  |  |  |  |  |
| Wild-type | 26 | 51% |  | 13 | 50% |  | 13 | 50% |  | 0.27 |
| Mutated | 11 | 22% |  | 3 | 27% |  | 8 | 73% |  |  |
| Unknown | 14 | 27% |  | 4 | 29% |  | 10 | 71% |  |  |
| **Suvival status** |  |  |  |  |  |  |  |  |  |  |
| Alive | 26 | 51% |  | 8 | 31% |  | 18 | 69% |  | 0.13 |
| Dead | 23 | 45% |  | 11 | 48% |  | 12 | 52% |  |  |
| Unknown | 2 | 4% |  | 1 | 50% |  | 1 | 50% |  |  |
|  |  |  |  |  |  |  |  |  |  |  |
| AYA= adolescents and young adults. * P-value was assessed according Chi-Square, or Fisher's test whenever required; P-value<0.05 is considered statistical significant. | | | | | | | | | | |
