# Supplementary material for: Epigenetic loss of the RNA decapping enzyme NUDT16 mediates C-MYC activation in T-cell acute lymphoblastic leukemia
Source: Leukemia. 2017 Apr 11;31(7):1622–5. doi: 10.1038/leu.2017.99 (PMC5501321; doi:10.1038/leu.2017.99)
Supplement: Supplementary Table S3 [file leu201799x12.docx]

**Supplementary Table 3.** List of clinicopathological and molecular characteristics for each T-ALL patient included in the study (N=51)

| **PATIENT**  **ID** | **GENDER** | **AGE GROUP** | **DIAGNOSTIC** | **CYTOGENETICS** | **NOTCH1 MUTATIONS** | **SURVIVAL**  **STATUS** | **OS**  **(months)** | **NUTD16 METHYLATION** |
| --- | --- | --- | --- | --- | --- | --- | --- | --- |
| 1 | Female | AYA | T-ALL | - | Wild-type | Alive | 2.35 | U |
| 2 | Male | AYA | T-ALL | - | Wild-type | Alive | 17.45 | U |
| 3 | Male | AYA | T-ALL | 46,XY[20] | Mutated | Dead | 18.50 | M |
| 4 | Male | AYA | T-ALL | 46,XY,i(9)(q10),del(11)(q23),inc.[12]/46,XY[3] | Wild-type | Dead | 1.39 | M |
| 5 | Male | Childhood | T-ALL | - | Wild-type | Unknown | Unknown | U |
| 6 | Male | Adult | Relapsed T-ALL | - | Unknown | Dead | 1.00 | U |
| 7 | Male | AYA | T-ALL | - | Wild-type | Alive | 9.08 | U |
| 8 | Male | AYA | T-ALL | - | Wild-type | Alive | 10.58 | M |
| 9 | Female | AYA | T-ALL | - | Mutated | Alive | 35.27 | M |
| 10 | Male | Adult | T-ALL | 46,XY,add(6)(q25),-7,+mar[3] | Wild-type | Dead | 2.65 | U |
| 11 | Female | AYA | T-ALL | - | Mutated | Alive | 41.87 | M |
| 12 | Male | Childhood | T-ALL | - | Mutated | Alive | 3.48 | M |
| 13 | Male | Adult | T-ALL | - | Wild-type | Alive | 0.66 | M |
| 14 | Male | AYA | T-ALL | 46,XY[20] | Unknown | Dead | 22.07 | U |
| 15 | Male | Childhood | T-ALL | - | Wild-type | Unknown | Unknown | M |
| 16 | Male | AYA | T-ALL | 46,XY[30] | Wild-type | Dead | 5.54 | U |
| 17 | Male | AYA | T-ALL | 46,XY, +8 | Wild-type | Alive | 22.47 | U |
| 18 | Female | Adult | T-ALL | Abn11 | Mutated | Dead | 2.35 | U |
| 19 | Male | Adult | T-ALL | 46, XY[20] | Mutated | Alive | 8.19 | U |
| 20 | Female | Adult | T-ALL | 46, XX[20] | Mutated | Alive | 4.60 | U |
| 21 | Female | Adult | T-ALL | 47,XX,+1,t(2;10)(p21;p11.2),-8,add(9)(p12),add(9)(q13),-13,-16,+mar1,+mar2,+mar3[13] /46,XX[7] | Wild-type | Alive | 20.00 | M |
| 22 | Female | Adult | T-ALL | 46,XX[20] | Wild-type | Dead | 10.00 | M |
| 23 | Male | Childhood | T-ALL | - | Wild-type | Alive | 46.09 | M |
| 24 | Male | Unknown | T-ALL | - | Wild-type | Alive | 49.05 | U |
| 25 | Male | Childhood | T-ALL | 46, XY[20] | Wild-type | Alive | 25.65 | U |
| 26 | Female | Adult | T-ALL | - | Mutated | Dead | 5.44 | M |
| 27 | Male | AYA | Relapsed T-ALL | 46, XY,der(1), -10, t(11;?)(q1) | Wild-type | Dead | 2.41 | M |
| 28 | Male | Childhood | T-ALL | 46,XY [20] | Mutated | Alive | 73.84 | M |
| 29 | Male | AYA | T-ALL | 46,XY [20] | Wild-type | Dead | 15.82 | U |
| 30 | Male | AYA | T-ALL | - | Wild-type | Dead | 9.05 | U |
| 31 | Female | Adult | T-ALL | Del6q | Wild-type | Alive | 6.80 | M |
| 32 | Male | Adult | T-ALL | - | Unknown | Alive | 22.31 | M |
| 33 | Male | AYA | T-ALL | - | Unknown | Alive | 3.74 | M |
| 34 | Male | Childhood | T-ALL | - | Unknown | Alive | 4.90 | M |
| 35 | Male | Childhood | T-ALL | 46;XY, 7q+; t(10;11)(p11-12;q1 | Wild-type | Dead | 15.58 | U |
| 36 | Male | AYA | T-ALL | 46,XY[20] | Wild-type | Alive | 106.70 | M |
| 37 | Male | AYA | T-ALL | 46, XY; t(11;14)(q23;q11-12) | Wild-type | Dead | 9.66 | U |
| 38 | Female | Adult | T-ALL | 46,XX, der(5),t(5;?)(q(?;?), 8 | Unknown | Dead | 29.97 | U |
| 39 | Male | AYA | T-ALL | - | Unknown | Dead | 3.81 | M |
| 40 | Male | Adult | T-ALL | 46,XY, 6q- | Unknown | Dead | 3.30 | U |
| 41 | Male | AYA | T-ALL | - | Unknown | Dead | 1.00 | M |
| 42 | Male | Adult | T-ALL | 46,XY,del(6)(q21q27),add(13)(q34)[17]/46,XY[3] | Unknown | Alive | 24.01 | M |
| 43 | Male | Adult | T-ALL | - | Unknown | Dead | 20.03 | M |
| 44 | Male | Adult | T-ALL | 46,XY[20] | Unknown | Alive | 137.72 | M |
| 45 | Male | AYA | T-ALL | 46,XY[20] | Mutated | Alive | 12.07 | M |
| 46 | Male | AYA | T-ALL | - | Unknown | Dead | 1.16 | M |
| 47 | Male | AYA | T-ALL | 46,XY[30] | Unknown | Dead | 11.77 | M |
| 48 | Male | Adult | T-ALL | 46,XY,del(2)(P10),+mar,inc[3]/46,XY[5] | Mutated | Dead | 4.63 | M |
| 49 | Male | Adult | T-ALL | - | Wild-type | Alive | 85.41 | M |
| 50 | Male | Adult | T-ALL | 46,XY,t(14;20)(q22;q13.1)[20] | Wild-type | Dead | 22.07 | M |
| 51 | Female | Adult | T-ALL | 46,XX,del(13)(q12q22),del(3)(q25)[15] / 46,XX[5] | Wild-type | Alive | 6.87 | M |

AYA: adolescents and young adults (16-29 years old); M: methylated; OS: overall survival; U: unmethylated
