# Supplementary material for: Epigenetic loss of the RNA decapping enzyme NUDT16 mediates C-MYC activation in T-cell acute lymphoblastic leukemia
Source: Leukemia. 2017 Apr 11;31(7):1622–5. doi: 10.1038/leu.2017.99 (PMC5501321; doi:10.1038/leu.2017.99)
Supplement: Supplementary Table S4 [file leu201799x13.docx]

**Supplementary Table 4.** Genes obtained from the NUDT16-RIP expression array.

| **GeneName** | **SystematicName** | **Description** |
| --- | --- | --- |
| USP37 | NM_020935 | ubiquitin specific peptidase 37 (USP37), mRNA [NM_020935] |
| MAP3K2 | NM_006609 | mitogen-activated protein kinase kinase kinase 2 (MAP3K2), mRNA [NM_006609] |
| GSK3B | NM_002093 | glycogen synthase kinase 3 beta (GSK3B), transcript variant 1, mRNA [NM_002093] |
| BCL11A | NM_022893 | B-cell CLL/lymphoma 11A (zinc finger protein) (BCL11A), transcript variant 1, mRNA [NM_022893] |
| FBXO28 | NM_015176 | F-box protein 28 (FBXO28), transcript variant 1, mRNA [NM_015176] |
| GNAS | ENST00000481768 | ens|GNAS complex locus [Source:HGNC Symbol;Acc:4392] [ENST00000481768] |
| EIF5B | NM_015904 | eukaryotic translation initiation factor 5B (EIF5B), mRNA [NM_015904] |
| XLOC_l2_013189 | TCONS_l2_00025009 | linc|BROAD Institute lincRNA (XLOC_l2_013189), lincRNA [TCONS_l2_00025009] |
| XLOC_000562 | ENST00000420347 | linc|BROAD Institute lincRNA (XLOC_000562), lincRNA [TCONS_00000392] |
| PRPF38B | NM_018061 | PRP38 pre-mRNA processing factor 38 (yeast) domain containing B (PRPF38B), transcript variant 1, mRNA [NM_018061] |
| PHAX | NM_032177 | phosphorylated adaptor for RNA export (PHAX), mRNA [NM_032177] |
| SNORD75 | NR_003941 | small nucleolar RNA, C/D box 75 (SNORD75), small nucleolar RNA [NR_003941] |
| PPIG | NM_004792 | peptidylprolyl isomerase G (cyclophilin G) (PPIG), mRNA [NM_004792] |
| KIAA1826 | NM_032424 | KIAA1826 (KIAA1826), mRNA [NM_032424] |
| CYP26C1 | AK131099 | gb|Homo sapiens mRNA for FLJ00329 protein. [AK131099] |
| GCC2 | NM_181453 | GRIP and coiled-coil domain containing 2 (GCC2), transcript variant 1, mRNA [NM_181453] |
| SNORD101 | NR_002434 | small nucleolar RNA, C/D box 101 (SNORD101), small nucleolar RNA [NR_002434] |
| ANKRD12 | NM_001204056 | ankyrin repeat domain 12 (ANKRD12), transcript variant 3, mRNA [NM_001204056] |
| EIF3A | NM_003750 | eukaryotic translation initiation factor 3, subunit A (EIF3A), mRNA [NM_003750] |
| SNORD77 | NR_003943 | small nucleolar RNA, C/D box 77 (SNORD77), small nucleolar RNA [NR_003943] |
| TRIM4 | NM_033017 | tripartite motif containing 4 (TRIM4), transcript variant alpha, mRNA [NM_033017] |
| MPHOSPH8 | NM_017520 | M-phase phosphoprotein 8 (MPHOSPH8), mRNA [NM_017520] |
| RPS6 | ENST00000380381 | ens|ribosomal protein S6 [Source:HGNC Symbol;Acc:10429] [ENST00000380381] |
| LOC100506302 | XR_108708 | ref|PREDICTED: Homo sapiens hypothetical LOC100506302 (LOC100506302), miscRNA [XR_108708] |
| DW451783 | DW451783 | gb|HHAGE004513 Human liver regeneration after partial hepatectomy Homo sapiens cDNA, mRNA sequence [DW451783] |
| ZC3H15 | NM_018471 | zinc finger CCCH-type containing 15 (ZC3H15), mRNA [NM_018471] |
| ZNF23 | NM_145911 | zinc finger protein 23 (KOX 16) (ZNF23), mRNA [NM_145911] |
| HNRNPA2B1 | NM_002137 | heterogeneous nuclear ribonucleoprotein A2/B1 (HNRNPA2B1), transcript variant A2, mRNA [NM_002137] |
| DHX40 | NM_024612 | DEAH (Asp-Glu-Ala-His) box polypeptide 40 (DHX40), transcript variant 1, mRNA [NM_024612] |
| C3orf33 | NM_173657 | chromosome 3 open reading frame 33 (C3orf33), mRNA [NM_173657] |
| MIER3 | NM_152622 | mesoderm induction early response 1, family member 3 (MIER3), mRNA [NM_152622] |
| XLOC_007502 | TCONS_00016114 | linc|BROAD Institute lincRNA (XLOC_007502), lincRNA [TCONS_00016114] |
| ZNF565 | NM_001042474 | zinc finger protein 565 (ZNF565), transcript variant 1, mRNA [NM_001042474] |
| SNORD44 | NR_002750 | small nucleolar RNA, C/D box 44 (SNORD44), small nuclear RNA [NR_002750] |
| RPS6KB1 | NM_003161 | ribosomal protein S6 kinase, 70kDa, polypeptide 1 (RPS6KB1), mRNA [NM_003161] |
| BMPR1A | NM_004329 | bone morphogenetic protein receptor, type IA (BMPR1A), mRNA [NM_004329] |
| RBM34 | NM_015014 | RNA binding motif protein 34 (RBM34), transcript variant 1, mRNA [NM_015014] |
| RPS6KC1 | NM_012424 | ribosomal protein S6 kinase, 52kDa, polypeptide 1 (RPS6KC1), transcript variant 1, mRNA [NM_012424] |
| CHD6 | ENST00000373222 | ens|chromodomain helicase DNA binding protein 6 [Source:HGNC Symbol;Acc:19057] [ENST00000373222] |
| NCL | NM_005381 | nucleolin (NCL), mRNA [NM_005381] |
| HBP1 | NM_012257 | HMG-box transcription factor 1 (HBP1), transcript variant 2, mRNA [NM_012257] |
| RNF20 | NM_019592 | ring finger protein 20 (RNF20), mRNA [NM_019592] |
| ARIH1 | NM_005744 | ariadne homolog, ubiquitin-conjugating enzyme E2 binding protein, 1 (Drosophila) (ARIH1), mRNA [NM_005744] |
| RBM25 | NM_021239 | RNA binding motif protein 25 (RBM25), mRNA [NM_021239] |
| TCERG1L | NM_174937 | transcription elongation regulator 1-like (TCERG1L), mRNA [NM_174937] |
| CCDC90A | NM_001031713 | coiled-coil domain containing 90A (CCDC90A), mRNA [NM_001031713] |
| ABCD3 | NM_001122674 | ATP-binding cassette, sub-family D (ALD), member 3 (ABCD3), transcript variant 2, mRNA [NM_001122674] |
| NP1202467 | NP1202467 | tc|GB|CR616535.1|CR616535.1 full-length cDNA clone CS0DI034YC12 of Placenta Cot 25-normalized of Homo sapiens (human) [NP1202467] |
| ANKRD11 | NM_013275 | ankyrin repeat domain 11 (ANKRD11), mRNA [NM_013275] |
| ARID4A | NM_002892 | AT rich interactive domain 4A (RBP1-like) (ARID4A), transcript variant 1, mRNA [NM_002892] |
| METTL14 | NM_020961 | methyltransferase like 14 (METTL14), mRNA [NM_020961] |
| GADD45A | NM_001924 | growth arrest and DNA-damage-inducible, alpha (GADD45A), transcript variant 1, mRNA [NM_001924] |
| LTV1 | NM_032860 | LTV1 homolog (S. cerevisiae) (LTV1), mRNA [NM_032860] |
| MGA | NM_001080541 | MAX gene associated (MGA), transcript variant 2, mRNA [NM_001080541] |
| A_33_P3229527 | A_33_P3229527 | Unknown |
| CCDC43 | NM_144609 | coiled-coil domain containing 43 (CCDC43), transcript variant 1, mRNA [NM_144609] |
| C9orf156 | NM_016481 | chromosome 9 open reading frame 156 (C9orf156), mRNA [NM_016481] |
| WDR53 | NM_182627 | WD repeat domain 53 (WDR53), mRNA [NM_182627] |
| PSMC3IP | NM_013290 | PSMC3 interacting protein (PSMC3IP), transcript variant 1, mRNA [NM_013290] |
| NRBF2 | NM_030759 | nuclear receptor binding factor 2 (NRBF2), mRNA [NM_030759] |
| ZKSCAN1 | NM_003439 | zinc finger with KRAB and SCAN domains 1 (ZKSCAN1), mRNA [NM_003439] |
| ERLIN1 | NM_006459 | ER lipid raft associated 1 (ERLIN1), mRNA [NM_006459] |
| A_33_P3264424 | A_33_P3264424 | Unknown |
| TASP1 | NM_017714 | taspase, threonine aspartase, 1 (TASP1), mRNA [NM_017714] |
| RPS27 | ENST00000392558 | ens|ribosomal protein S27 [Source:HGNC Symbol;Acc:10416] [ENST00000392558] |
| ZNF225 | NM_013362 | zinc finger protein 225 (ZNF225), mRNA [NM_013362] |
| ZNF148 | NM_021964 | zinc finger protein 148 (ZNF148), mRNA [NM_021964] |
| CEP89 | NM_032816 | centrosomal protein 89kDa (CEP89), mRNA [NM_032816] |
| ZBTB43 | NM_014007 | zinc finger and BTB domain containing 43 (ZBTB43), transcript variant 1, mRNA [NM_014007] |
| CSNK2A1 | NM_177559 | casein kinase 2, alpha 1 polypeptide (CSNK2A1), transcript variant 1, mRNA [NM_177559] |
| XLOC_l2_010558 | TCONS_l2_00020391 | linc|BROAD Institute lincRNA (XLOC_l2_010558), lincRNA [TCONS_l2_00020391] |
| SNORD8 | NR_002916 | small nucleolar RNA, C/D box 8 (SNORD8), small nucleolar RNA [NR_002916] |
| SNORD24 | NR_002447 | small nucleolar RNA, C/D box 24 (SNORD24), small nucleolar RNA [NR_002447] |
| XLOC_l2_005438 | TCONS_l2_00010088 | linc|BROAD Institute lincRNA (XLOC_l2_005438), lincRNA [TCONS_l2_00010088] |
| FAIM | NM_001033030 | Fas apoptotic inhibitory molecule (FAIM), transcript variant 1, mRNA [NM_001033030] |
| CEBPZ | NM_005760 | CCAAT/enhancer binding protein (C/EBP), zeta (CEBPZ), mRNA [NM_005760] |
| LOC399900 | XR_111182 | ref|PREDICTED: Homo sapiens hypothetical LOC399900 (LOC399900), miscRNA [XR_111182] |
| ZNF507 | AB029007 | gb|Homo sapiens mRNA for KIAA1084 protein, partial cds. [AB029007] |
| MARS2 | NM_138395 | methionyl-tRNA synthetase 2, mitochondrial (MARS2), nuclear gene encoding mitochondrial protein, mRNA [NM_138395] |
| EFTUD1 | NM_024580 | elongation factor Tu GTP binding domain containing 1 (EFTUD1), transcript variant 1, mRNA [NM_024580] |
| EBLN2 | NM_018029 | endogenous Bornavirus-like nucleoprotein 2 (EBLN2), mRNA [NM_018029] |
| ZNF684 | NM_152373 | zinc finger protein 684 (ZNF684), mRNA [NM_152373] |
| ENST00000370708 | ENST00000370708 | ens|zinc finger protein 451 [Source:HGNC Symbol;Acc:21091] [ENST00000370708] |
| UTP3 | NM_020368 | UTP3, small subunit (SSU) processome component, homolog (S. cerevisiae) (UTP3), mRNA [NM_020368] |
| GNAQ | NM_002072 | guanine nucleotide binding protein (G protein), q polypeptide (GNAQ), mRNA [NM_002072] |
| RNU105A | NR_004404 | RNA, U105A small nucleolar (RNU105A), small nucleolar RNA [NR_004404] |
| LRRC8C | ENST00000479252 | linc|BROAD Institute lincRNA (XLOC_000290), lincRNA [TCONS_00001023] |
| EHBP1 | NM_015252 | EH domain binding protein 1 (EHBP1), transcript variant 1, mRNA [NM_015252] |
| NOP58 | NM_015934 | NOP58 ribonucleoprotein homolog (yeast) (NOP58), mRNA [NM_015934] |
| ATF7IP | NM_018179 | activating transcription factor 7 interacting protein (ATF7IP), mRNA [NM_018179] |
| AHCTF1 | NM_015446 | AT hook containing transcription factor 1 (AHCTF1), mRNA [NM_015446] |
| BMS1P5 | BC065722 | tc|D80009 Start codon is not identified {Homo sapiens} (exp=-1; wgp=0; cg=0), partial (12%) [THC2480131] |
| PDCL | NM_005388 | phosducin-like (PDCL), mRNA [NM_005388] |
| ALS2 | ENST00000410052 | ens|amyotrophic lateral sclerosis 2 (juvenile) [Source:HGNC Symbol;Acc:443] [ENST00000410052] |
| ETF1 | NM_004730 | eukaryotic translation termination factor 1 (ETF1), mRNA [NM_004730] |
| MRPL32 | NM_031903 | mitochondrial ribosomal protein L32 (MRPL32), nuclear gene encoding mitochondrial protein, mRNA [NM_031903] |
| CHEK1 | NM_001114121 | CHK1 checkpoint homolog (S. pombe) (CHEK1), transcript variant 2, mRNA [NM_001114121] |
| SNORD65 | NR_003054 | small nucleolar RNA, C/D box 65 (SNORD65), small nucleolar RNA [NR_003054] |
| XLOC_l2_003627 | TCONS_l2_00006722 | linc|BROAD Institute lincRNA (XLOC_l2_003627), lincRNA [TCONS_l2_00006722] |
| SNORA6 | NR_002325 | small nucleolar RNA, H/ACA box 6 (SNORA6), small nucleolar RNA [NR_002325] |
| E2F6 | NM_198256 | E2F transcription factor 6 (E2F6), mRNA [NM_198256] |
| C18orf21 | NM_031446 | chromosome 18 open reading frame 21 (C18orf21), transcript variant 1, mRNA [NM_031446] |
| SNORD72 | NR_002583 | small nucleolar RNA, C/D box 72 (SNORD72), small nucleolar RNA [NR_002583] |
| AIMP1 | NM_004757 | aminoacyl tRNA synthetase complex-interacting multifunctional protein 1 (AIMP1), transcript variant 1, mRNA [NM_004757] |
| XLOC_l2_004595 | THC2554687 | linc|BROAD Institute lincRNA (XLOC_l2_004595), lincRNA [TCONS_l2_00008487] |
| VEZT | NM_017599 | vezatin, adherens junctions transmembrane protein (VEZT), transcript variant 1, mRNA [NM_017599] |
| TTF1 | NM_007344 | transcription termination factor, RNA polymerase I (TTF1), transcript variant 1, mRNA [NM_007344] |
| TYW1B | NM_001145441 | tRNA-yW synthesizing protein 1 homolog B (S. cerevisiae) (TYW1B), transcript variant 2, mRNA [NM_001145441] |
| KIN | NM_012311 | KIN, antigenic determinant of recA protein homolog (mouse) (KIN), mRNA [NM_012311] |
| SNORD66 | NR_003055 | small nucleolar RNA, C/D box 66 (SNORD66), small nucleolar RNA [NR_003055] |
| WDR20 | NM_001242417 | WD repeat domain 20 (WDR20), transcript variant 7, mRNA [NM_001242417] |
| TTTY7 | NR_001534 | testis-specific transcript, Y-linked 7 (non-protein coding) (TTTY7), non-coding RNA [NR_001534] |
| CSNK1G1 | ENST00000447727 | ens|casein kinase 1, gamma 1 [Source:HGNC Symbol;Acc:2454] [ENST00000447727] |
| GNL1 | NM_005275 | guanine nucleotide binding protein-like 1 (GNL1), mRNA [NM_005275] |
| ZNF426 | NM_024106 | zinc finger protein 426 (ZNF426), mRNA [NM_024106] |
| C6orf105 | ENST00000379413 | ens|chromosome 6 open reading frame 105 [Source:HGNC Symbol;Acc:21214] [ENST00000379413] |
| FAM176A | NM_032181 | family with sequence similarity 176, member A (FAM176A), transcript variant 2, mRNA [NM_032181] |
| LOC730101 | NR_024403 | uncharacterized LOC730101 (LOC730101), transcript variant 1, non-coding RNA [NR_024403] |
| ZNF614 | NM_025040 | zinc finger protein 614 (ZNF614), mRNA [NM_025040] |
| ZNF813 | NM_001004301 | zinc finger protein 813 (ZNF813), mRNA [NM_001004301] |
| HIST1H2AC | NM_003512 | histone cluster 1, H2ac (HIST1H2AC), mRNA [NM_003512] |
| SMN2 | NM_022877 | survival of motor neuron 2, centromeric (SMN2), transcript variant c, mRNA [NM_022877] |
| A_33_P3280502 | A_33_P3280502 | Unknown |
| ZNF287 | NM_020653 | zinc finger protein 287 (ZNF287), mRNA [NM_020653] |
| FAM36A | NM_198076 | family with sequence similarity 36, member A (FAM36A), mRNA [NM_198076] |
| PGM5P2 | NR_002836 | phosphoglucomutase 5 pseudogene 2 (PGM5P2), non-coding RNA [NR_002836] |
| YIPF5 | NM_001024947 | Yip1 domain family, member 5 (YIPF5), transcript variant 1, mRNA [NM_001024947] |
| C1orf55 | NM_152608 | chromosome 1 open reading frame 55 (C1orf55), mRNA [NM_152608] |
| UTRN | NM_007124 | utrophin (UTRN), mRNA [NM_007124] |
| PTGR1 | ENST00000374313 | ens|prostaglandin reductase 1 [Source:HGNC Symbol;Acc:18429] [ENST00000374313] |
| AFTPH | NM_203437 | aftiphilin (AFTPH), transcript variant 1, mRNA [NM_203437] |
| ENST00000448363 | ENST00000448363 | ref|PREDICTED: Homo sapiens hypothetical protein LOC100507172 (LOC100507172), mRNA [XM_003118552] |
| NOLC1 | NM_004741 | nucleolar and coiled-body phosphoprotein 1 (NOLC1), mRNA [NM_004741] |
| MIS18A | NM_018944 | MIS18 kinetochore protein homolog A (S. pombe) (MIS18A), mRNA [NM_018944] |
| GUCY1A3 | NM_001130686 | guanylate cyclase 1, soluble, alpha 3 (GUCY1A3), transcript variant 6, mRNA [NM_001130686] |
| USP27X | NM_001145073 | ubiquitin specific peptidase 27, X-linked (USP27X), mRNA [NM_001145073] |
| NEK4 | NM_003157 | NIMA (never in mitosis gene a)-related kinase 4 (NEK4), transcript variant 1, mRNA [NM_003157] |
| TRIM44 | NM_017583 | tripartite motif containing 44 (TRIM44), mRNA [NM_017583] |
| OBFC2A | NM_001031716 | oligonucleotide/oligosaccharide-binding fold containing 2A (OBFC2A), transcript variant 1, mRNA [NM_001031716] |
| LOC100506294 | XR_109196 | ref|PREDICTED: Homo sapiens hypothetical LOC100506294, transcript variant 2 (LOC100506294), miscRNA [XR_109196] |
| HDAC2 | NM_001527 | histone deacetylase 2 (HDAC2), transcript variant 1, mRNA [NM_001527] |
| UBTFL1 | NM_001143975 | upstream binding transcription factor, RNA polymerase I-like 1 (UBTFL1), mRNA [NM_001143975] |
| MRPL46 | NM_022163 | mitochondrial ribosomal protein L46 (MRPL46), nuclear gene encoding mitochondrial protein, mRNA [NM_022163] |
| EXOSC9 | NM_005033 | exosome component 9 (EXOSC9), transcript variant 2, mRNA [NM_005033] |
| NAP1L5 | NM_153757 | nucleosome assembly protein 1-like 5 (NAP1L5), mRNA [NM_153757] |
| TAPT1 | NM_153365 | transmembrane anterior posterior transformation 1 (TAPT1), mRNA [NM_153365] |
| ZNF12 | NM_016265 | zinc finger protein 12 (ZNF12), transcript variant 1, mRNA [NM_016265] |
| RAPGEF2 | NM_014247 | Rap guanine nucleotide exchange factor (GEF) 2 (RAPGEF2), mRNA [NM_014247] |
| CSGALNACT2 | NM_018590 | chondroitin sulfate N-acetylgalactosaminyltransferase 2 (CSGALNACT2), mRNA [NM_018590] |
| TAF3 | NM_031923 | TAF3 RNA polymerase II, TATA box binding protein (TBP)-associated factor, 140kDa (TAF3), mRNA [NM_031923] |
| IVNS1ABP | NM_006469 | influenza virus NS1A binding protein (IVNS1ABP), mRNA [NM_006469] |
| ENST00000453166 | ENST00000453166 | ens|immunoglobulin kappa variable 2D-28 [Source:HGNC Symbol;Acc:5799] [ENST00000453166] |
| ZNF181 | NM_001029997 | zinc finger protein 181 (ZNF181), transcript variant 1, mRNA [NM_001029997] |
| BPTF | ENST00000342579 | ens|bromodomain PHD finger transcription factor [Source:HGNC Symbol;Acc:3581] [ENST00000342579] |
| AFG3L2 | NM_006796 | AFG3 ATPase family gene 3-like 2 (S. cerevisiae) (AFG3L2), nuclear gene encoding mitochondrial protein, mRNA [NM_006796] |
| ZNF217 | NM_006526 | zinc finger protein 217 (ZNF217), mRNA [NM_006526] |
| CCDC76 | ENST00000370139 | ens|coiled-coil domain containing 76 [Source:HGNC Symbol;Acc:25502] [ENST00000370139] |
| CRNKL1 | NM_016652 | crooked neck pre-mRNA splicing factor-like 1 (Drosophila) (CRNKL1), mRNA [NM_016652] |
| NDEL1 | NM_030808 | nudE nuclear distribution gene E homolog (A. nidulans)-like 1 (NDEL1), transcript variant 2, mRNA [NM_030808] |
| XLOC_009233 | THC2674548 | tc|ALU1_HUMAN (P39188) Alu subfamily J sequence contamination warning entry, partial (15%) [THC2674548] |
| PPP1R8 | NM_138558 | protein phosphatase 1, regulatory subunit 8 (PPP1R8), transcript variant 2, mRNA [NM_138558] |
| UBXN7 | NM_015562 | UBX domain protein 7 (UBXN7), mRNA [NM_015562] |
| A_33_P3268408 | A_33_P3268408 | Unknown |
| LOC100499466 | NR_027418 | uncharacterized LOC100499466 (LOC100499466), non-coding RNA [NR_027418] |
| ZNF480 | NM_144684 | zinc finger protein 480 (ZNF480), mRNA [NM_144684] |
| SNORD12 | NR_003030 | small nucleolar RNA, C/D box 12 (SNORD12), small nucleolar RNA [NR_003030] |
| SNORD32A | NR_000021 | small nucleolar RNA, C/D box 32A (SNORD32A), small nucleolar RNA [NR_000021] |
| THC2524986 | THC2524986 | tc|CDC2L1S13 PITSLRE protein kinase alpha SV9 isoform {Homo sapiens} (exp=-1; wgp=0; cg=0), partial (38%) [THC2524986] |
| XLOC_l2_014050 | TCONS_l2_00026968 | linc|BROAD Institute lincRNA (XLOC_l2_014050), lincRNA [TCONS_l2_00026968] |
| XLOC_000152 | THC2785820 | linc|BROAD Institute lincRNA (XLOC_000152), lincRNA [TCONS_00001989] |
| LOC439911 | AK127450 | gb|Homo sapiens cDNA FLJ45542 fis, clone BRTHA2033320. [AK127450] |
| RRAGD | NM_021244 | Ras-related GTP binding D (RRAGD), mRNA [NM_021244] |
| SNORD58A | NR_002571 | small nucleolar RNA, C/D box 58A (SNORD58A), small nuclear RNA [NR_002571] |
| RPGR | NM_000328 | retinitis pigmentosa GTPase regulator (RPGR), transcript variant A, mRNA [NM_000328] |
| E2F3 | NM_001949 | E2F transcription factor 3 (E2F3), transcript variant 1, mRNA [NM_001949] |
| PDIA5 | NM_006810 | protein disulfide isomerase family A, member 5 (PDIA5), transcript variant 1, mRNA [NM_006810] |
| RBBP7 | NM_002893 | retinoblastoma binding protein 7 (RBBP7), transcript variant 2, mRNA [NM_002893] |
| CYP20A1 | NM_177538 | cytochrome P450, family 20, subfamily A, polypeptide 1 (CYP20A1), mRNA [NM_177538] |
| USP32 | NM_032582 | ubiquitin specific peptidase 32 (USP32), mRNA [NM_032582] |
| LEPROT | NM_017526 | leptin receptor overlapping transcript (LEPROT), transcript variant 1, mRNA [NM_017526] |
| ZNF134 | NM_003435 | zinc finger protein 134 (ZNF134), mRNA [NM_003435] |
| FLJ43681 | NR_029406 | ribosomal protein L23a pseudogene (FLJ43681), non-coding RNA [NR_029406] |
| SOD2 | NM_001024465 | superoxide dismutase 2, mitochondrial (SOD2), nuclear gene encoding mitochondrial protein, transcript variant 2, mRNA [NM_001024465] |
| UGCG | NM_003358 | UDP-glucose ceramide glucosyltransferase (UGCG), mRNA [NM_003358] |
| RABGGTB | NM_004582 | Rab geranylgeranyltransferase, beta subunit (RABGGTB), mRNA [NM_004582] |
| C20orf106 | NM_001012971 | chromosome 20 open reading frame 106 (C20orf106), mRNA [NM_001012971] |
| EPC1 | NM_025209 | enhancer of polycomb homolog 1 (Drosophila) (EPC1), mRNA [NM_025209] |
| KCTD3 | NM_016121 | potassium channel tetramerisation domain containing 3 (KCTD3), mRNA [NM_016121] |
| ZAK | NM_016653 | sterile alpha motif and leucine zipper containing kinase AZK (ZAK), transcript variant 1, mRNA [NM_016653] |
| PPCS | ENST00000372560 | ens|phosphopantothenoylcysteine synthetase [Source:HGNC Symbol;Acc:25686] [ENST00000372560] |
| NT5C3 | NM_001002010 | 5'-nucleotidase, cytosolic III (NT5C3), transcript variant 1, mRNA [NM_001002010] |
| BTBD7 | NM_018167 | BTB (POZ) domain containing 7 (BTBD7), transcript variant 2, mRNA [NM_018167] |
| METTL18 | NM_033418 | methyltransferase like 18 (METTL18), mRNA [NM_033418] |
| ARRDC4 | NM_183376 | arrestin domain containing 4 (ARRDC4), mRNA [NM_183376] |
| NKRF | NM_017544 | NFKB repressing factor (NKRF), transcript variant 2, mRNA [NM_017544] |
| XLOC_005990 | ENST00000445093 | linc|BROAD Institute lincRNA (XLOC_005990), lincRNA [TCONS_00012957] |
| THC2665222 | THC2665222 | tc|Q6QI92_RAT (Q6QI92) LRRG00116, partial (5%) [THC2665222] |
| HYLS1 | NM_145014 | hydrolethalus syndrome 1 (HYLS1), transcript variant 1, mRNA [NM_145014] |
| CUL1 | NM_003592 | cullin 1 (CUL1), mRNA [NM_003592] |
| LOC100129961 | NR_036549 | uncharacterized LOC100129961 (LOC100129961), non-coding RNA [NR_036549] |
| RAB12 | NM_001025300 | RAB12, member RAS oncogene family (RAB12), mRNA [NM_001025300] |
| PDHX | NM_003477 | pyruvate dehydrogenase complex, component X (PDHX), nuclear gene encoding mitochondrial protein, transcript variant 1, mRNA [NM_003477] |
| PIK3R1 | NM_181523 | phosphoinositide-3-kinase, regulatory subunit 1 (alpha) (PIK3R1), transcript variant 1, mRNA [NM_181523] |
| LOC100130442 | AK131364 | gb|Homo sapiens cDNA FLJ16405 fis, clone UTERU2011220. [AK131364] |
| XLOC_007777 | TCONS_00016406 | linc|BROAD Institute lincRNA (XLOC_007777), lincRNA [TCONS_00016406] |
| SNORD28 | NR_002562 | small nucleolar RNA, C/D box 28 (SNORD28), small nucleolar RNA [NR_002562] |
| ZNF146 | NM_007145 | zinc finger protein 146 (ZNF146), transcript variant 1, mRNA [NM_007145] |
| XLOC_010525 | THC2670501 | tc|Q7Z5D8_HUMAN (Q7Z5D8) Homeobox C14 protein, partial (13%) [THC2670501] |
| TTC25 | NM_031421 | tetratricopeptide repeat domain 25 (TTC25), mRNA [NM_031421] |
| NCBP1 | NM_002486 | nuclear cap binding protein subunit 1, 80kDa (NCBP1), mRNA [NM_002486] |
| CDADC1 | NM_001193478 | cytidine and dCMP deaminase domain containing 1 (CDADC1), transcript variant 2, mRNA [NM_001193478] |
| LOC100130744 | AF289590 | gb|Homo sapiens clone pp7583 unknown mRNA. [AF289590] |
| XLOC_005834 | TCONS_00012278 | linc|BROAD Institute lincRNA (XLOC_005834), lincRNA [TCONS_00012278] |
| CCNA2 | NM_001237 | cyclin A2 (CCNA2), mRNA [NM_001237] |
| TAOK3 | NM_016281 | TAO kinase 3 (TAOK3), mRNA [NM_016281] |
| TBPL1 | NM_004865 | TBP-like 1 (TBPL1), mRNA [NM_004865] |
| XLOC_007433 | BI046002 | gb|MR3-FN0206-020201-006-h05 FN0206 Homo sapiens cDNA, mRNA sequence [BI046002] |
| PARP4 | NM_006437 | poly (ADP-ribose) polymerase family, member 4 (PARP4), mRNA [NM_006437] |
| ZNF669 | NM_024804 | zinc finger protein 669 (ZNF669), transcript variant 1, mRNA [NM_024804] |
| FLJ38717 | XR_108650 | ref|PREDICTED: Homo sapiens FLJ38717 protein (FLJ38717), miscRNA [XR_108650] |
| LOC153546 | AK055939 | gb|Homo sapiens cDNA FLJ31377 fis, clone NESOP1000087. [AK055939] |
| CYB5D1 | NM_144607 | cytochrome b5 domain containing 1 (CYB5D1), mRNA [NM_144607] |
| HIST1H2AA | NM_170745 | histone cluster 1, H2aa (HIST1H2AA), mRNA [NM_170745] |
| XLOC_006258 | TCONS_00014217 | linc|BROAD Institute lincRNA (XLOC_006258), lincRNA [TCONS_00014217] |
| USP47 | ENST00000525257 | ens|ubiquitin specific peptidase 47 [Source:HGNC Symbol;Acc:20076] [ENST00000525257] |
| XLOC_012440 | BC062794 | tc|Q2VF20_PAROL (Q2VF20) Myogenic factor MyoD, partial (6%) [THC2558618] |
| ZFAND4 | NM_174890 | AN1, ubiquitin-like, homolog (Xenopus laevis) (ANUBL1), transcript variant 1, mRNA [NM_174890] |
| NMT2 | NM_004808 | N-myristoyltransferase 2 (NMT2), mRNA [NM_004808] |
| ZNF699 | NM_198535 | zinc finger protein 699 (ZNF699), mRNA [NM_198535] |
| LOC100294145 | NR_037178 | uncharacterized LOC100294145 (LOC100294145), transcript variant 2, non-coding RNA [NR_037178] |
| USP45 | NM_001080481 | ubiquitin specific peptidase 45 (USP45), mRNA [NM_001080481] |
| LOC100287765 | NR_038988 | uncharacterized LOC100287765 (LOC100287765), non-coding RNA [NR_038988] |
| CD55 | NM_000574 | CD55 molecule, decay accelerating factor for complement (Cromer blood group) (CD55), transcript variant 1, mRNA [NM_000574] |
| XLOC_l2_015491 | THC2487640 | linc|BROAD Institute lincRNA (XLOC_l2_015491), lincRNA [TCONS_l2_00030182] |
| MARCH7 | NM_022826 | membrane-associated ring finger (C3HC4) 7 (MARCH7), mRNA [NM_022826] |
| ZNF544 | NM_014480 | zinc finger protein 544 (ZNF544), mRNA [NM_014480] |
| KAT6B | NM_012330 | K(lysine) acetyltransferase 6B (KAT6B), mRNA [NM_012330] |
| ING3 | NM_019071 | inhibitor of growth family, member 3 (ING3), transcript variant 1, mRNA [NM_019071] |
| ADAM17 | NM_003183 | ADAM metallopeptidase domain 17 (ADAM17), mRNA [NM_003183] |
| C17orf76-AS1 | NR_045024 | C17orf76 antisense RNA 1 (non-protein coding) (C17orf76-AS1), transcript variant 27, non-coding RNA [NR_045024] |
| KLHL11 | NM_018143 | kelch-like 11 (Drosophila) (KLHL11), mRNA [NM_018143] |
| A_33_P3282649 | A_33_P3282649 | Unknown |
| FTSJD1 | NM_018348 | FtsJ methyltransferase domain containing 1 (FTSJD1), transcript variant 1, mRNA [NM_018348] |
| WDR70 | NM_018034 | WD repeat domain 70 (WDR70), mRNA [NM_018034] |
| SBDS | NM_016038 | Shwachman-Bodian-Diamond syndrome (SBDS), mRNA [NM_016038] |
| ZNF812 | NM_001199814 | zinc finger protein 812 (ZNF812), mRNA [NM_001199814] |
| CALU | NM_001219 | calumenin (CALU), transcript variant 1, mRNA [NM_001219] |
| LOC100507904 | XM_003119663 | ref|PREDICTED: Homo sapiens hypothetical protein LOC100507904 (LOC100507904), mRNA [XM_003119663] |
| C17orf48 | NM_020233 | chromosome 17 open reading frame 48 (C17orf48), mRNA [NM_020233] |
| TERF1 | NM_017489 | telomeric repeat binding factor (NIMA-interacting) 1 (TERF1), transcript variant 1, mRNA [NM_017489] |
| RSRC2 | NM_023012 | arginine/serine-rich coiled-coil 2 (RSRC2), transcript variant 1, mRNA [NM_023012] |
| UBR2 | NM_015255 | ubiquitin protein ligase E3 component n-recognin 2 (UBR2), transcript variant 1, mRNA [NM_015255] |
| XLOC_003399 | ENST00000441644 | gb|AGENCOURT_10626084 NIH_MGC_141 Homo sapiens cDNA clone IMAGE:6739185 5', mRNA sequence [BU960446] |
| TRA2B | NM_001243879 | transformer 2 beta homolog (Drosophila) (TRA2B), transcript variant 2, mRNA [NM_001243879] |
| ZNF326 | NM_182975 | zinc finger protein 326 (ZNF326), transcript variant 3, mRNA [NM_182975] |
| RIOK2 | NM_018343 | RIO kinase 2 (yeast) (RIOK2), transcript variant 1, mRNA [NM_018343] |
| LLPH | NM_032338 | LLP homolog, long-term synaptic facilitation (Aplysia) (LLPH), mRNA [NM_032338] |
| FIGNL1 | NM_001042762 | fidgetin-like 1 (FIGNL1), transcript variant 1, mRNA [NM_001042762] |
| XLOC_l2_014048 | ENST00000481762 | linc|BROAD Institute lincRNA (XLOC_l2_014048), lincRNA [TCONS_l2_00027517] |
| ZNF235 | NM_004234 | zinc finger protein 235 (ZNF235), mRNA [NM_004234] |
| ZNF350 | NM_021632 | zinc finger protein 350 (ZNF350), mRNA [NM_021632] |
| AK021933 | AK021933 | gb|Homo sapiens cDNA FLJ11871 fis, clone HEMBA1007052. [AK021933] |
| HIST3H2A | NM_033445 | histone cluster 3, H2a (HIST3H2A), mRNA [NM_033445] |
| KIAA1429 | NM_183009 | KIAA1429 (KIAA1429), transcript variant 2, mRNA [NM_183009] |
| SP140L | NM_138402 | SP140 nuclear body protein-like (SP140L), mRNA [NM_138402] |
| FTSJ3 | NM_017647 | FtsJ homolog 3 (E. coli) (FTSJ3), mRNA [NM_017647] |
| DDX19B | NM_007242 | DEAD (Asp-Glu-Ala-As) box polypeptide 19B (DDX19B), transcript variant 1, mRNA [NM_007242] |
| COQ10B | NM_025147 | coenzyme Q10 homolog B (S. cerevisiae) (COQ10B), nuclear gene encoding mitochondrial protein, mRNA [NM_025147] |
| LOC100506694 | XR_132651 | ref|PREDICTED: Homo sapiens hypothetical LOC100506694, transcript variant 4 (LOC100506694), miscRNA [XR_132651] |
| XLOC_l2_003886 | TCONS_l2_00007050 | linc|BROAD Institute lincRNA (XLOC_l2_003886), lincRNA [TCONS_l2_00007050] |
| SNORD94 | NR_004378 | small nucleolar RNA, C/D box 94 (SNORD94), small nucleolar RNA [NR_004378] |
| STAT1 | NM_139266 | signal transducer and activator of transcription 1, 91kDa (STAT1), transcript variant beta, mRNA [NM_139266] |
| NR3C1 | NM_001018077 | nuclear receptor subfamily 3, group C, member 1 (glucocorticoid receptor) (NR3C1), transcript variant 5, mRNA [NM_001018077] |
| PHF20 | NM_016436 | PHD finger protein 20 (PHF20), mRNA [NM_016436] |
| EIF4ENIF1 | NM_001164502 | eukaryotic translation initiation factor 4E nuclear import factor 1 (EIF4ENIF1), transcript variant 3, mRNA [NM_001164502] |
| OR9H1P | AY358215 | gb|Homo sapiens clone DNA175742 VNFT9373 (UNQ9373) mRNA, complete cds. [AY358215] |
| SNRNP40 | NM_004814 | small nuclear ribonucleoprotein 40kDa (U5) (SNRNP40), mRNA [NM_004814] |
| ZCCHC6 | NM_024617 | zinc finger, CCHC domain containing 6 (ZCCHC6), transcript variant 1, mRNA [NM_024617] |
| PPFIA1 | NM_177423 | protein tyrosine phosphatase, receptor type, f polypeptide (PTPRF), interacting protein (liprin), alpha 1 (PPFIA1), transcript variant 1, mRNA [NM_177423] |
| SETMAR | NM_006515 | SET domain and mariner transposase fusion gene (SETMAR), transcript variant 1, mRNA [NM_006515] |
| XLOC_l2_012081 | ENST00000447012 | linc|BROAD Institute lincRNA (XLOC_l2_012081), lincRNA [TCONS_l2_00023773] |
| AMACR | NM_001167595 | alpha-methylacyl-CoA racemase (AMACR), nuclear gene encoding mitochondrial protein, transcript variant 3, mRNA [NM_001167595] |
| MOB1A | AK123865 | linc|BROAD Institute lincRNA (XLOC_001532), lincRNA [TCONS_00002935] |
| NELL2 | NM_006159 | NEL-like 2 (chicken) (NELL2), transcript variant 2, mRNA [NM_006159] |
| METTL16 | NM_024086 | methyltransferase like 16 (METTL16), mRNA [NM_024086] |
| A_33_P3240652 | A_33_P3240652 | Unknown |
| ALKBH8 | NM_138775 | alkB, alkylation repair homolog 8 (E. coli) (ALKBH8), mRNA [NM_138775] |
| ADAMTSL4 | NM_019032 | ADAMTS-like 4 (ADAMTSL4), transcript variant 1, mRNA [NM_019032] |
| TTLL5 | NM_015072 | tubulin tyrosine ligase-like family, member 5 (TTLL5), mRNA [NM_015072] |
| A_32_P101844 | A_32_P101844 | Unknown |
| LOC100507599 | XR_109753 | ref|PREDICTED: Homo sapiens hypothetical LOC100507599 (LOC100507599), miscRNA [XR_109753] |
| SAP30BP | NM_013260 | SAP30 binding protein (SAP30BP), mRNA [NM_013260] |
| C10orf12 | NM_015652 | chromosome 10 open reading frame 12 (C10orf12), mRNA [NM_015652] |
| TMSB4Y | NM_004202 | thymosin beta 4, Y-linked (TMSB4Y), mRNA [NM_004202] |
| SNORD19B | NR_003687 | small nucleolar RNA, C/D box 19B (SNORD19B), small nucleolar RNA [NR_003687] |
| XLOC_013439 | ENST00000418739 | gb|AGENCOURT_7575824 NIH_MGC_72 Homo sapiens cDNA clone IMAGE:6047489 5', mRNA sequence [BQ218150] |
| C9orf123 | NM_033428 | chromosome 9 open reading frame 123 (C9orf123), mRNA [NM_033428] |
| SNORA75 | AW382724 | gb|PM2-HT0338-051199-001-c04 HT0338 Homo sapiens cDNA, mRNA sequence [AW382724] |
| XLOC_005541 | TCONS_00012017 | linc|BROAD Institute lincRNA (XLOC_005541), lincRNA [TCONS_00012017] |
| MDM2 | NM_002392 | Mdm2 p53 binding protein homolog (mouse) (MDM2), transcript variant MDM2, mRNA [NM_002392] |
| TRIM38 | NM_006355 | tripartite motif containing 38 (TRIM38), mRNA [NM_006355] |
| UBE3A | ENST00000428984 | ens|ubiquitin protein ligase E3A [Source:HGNC Symbol;Acc:12496] [ENST00000428984] |
| CCDC59 | NM_014167 | coiled-coil domain containing 59 (CCDC59), transcript variant 1, mRNA [NM_014167] |
| AKAP12 | NM_144497 | A kinase (PRKA) anchor protein 12 (AKAP12), transcript variant 2, mRNA [NM_144497] |
| ZNF10 | NM_015394 | zinc finger protein 10 (ZNF10), mRNA [NM_015394] |
| CDC14A | NM_033313 | CDC14 cell division cycle 14 homolog A (S. cerevisiae) (CDC14A), transcript variant 3, mRNA [NM_033313] |
| CNTRL | NM_007018 | centriolin (CNTRL), mRNA [NM_007018] |
| USP10 | NM_005153 | ubiquitin specific peptidase 10 (USP10), mRNA [NM_005153] |
| HCFC2 | NM_013320 | host cell factor C2 (HCFC2), mRNA [NM_013320] |
| GCNT4 | NM_016591 | glucosaminyl (N-acetyl) transferase 4, core 2 (GCNT4), mRNA [NM_016591] |
| SNORA41 | NR_002590 | small nucleolar RNA, H/ACA box 41 (SNORA41), small nucleolar RNA [NR_002590] |
| ZNF131 | NM_003432 | zinc finger protein 131 (ZNF131), mRNA [NM_003432] |
| PNN | NM_002687 | pinin, desmosome associated protein (PNN), mRNA [NM_002687] |
| XLOC_009387 | TCONS_00020046 | linc|BROAD Institute lincRNA (XLOC_009387), lincRNA [TCONS_00020046] |
| SNORD88C | NR_003069 | small nucleolar RNA, C/D box 88C (SNORD88C), small nucleolar RNA [NR_003069] |
| CAP1 | NM_006367 | CAP, adenylate cyclase-associated protein 1 (yeast) (CAP1), transcript variant 1, mRNA [NM_006367] |
| ARID4B | NM_016374 | AT rich interactive domain 4B (RBP1-like) (ARID4B), transcript variant 1, mRNA [NM_016374] |
| ARID5A | AY358167 | gb|Homo sapiens clone DNA128322 RFVG5814 (UNQ5814) mRNA, complete cds. [AY358167] |
| SPEN | NM_015001 | spen homolog, transcriptional regulator (Drosophila) (SPEN), mRNA [NM_015001] |
| TAF9 | NM_001015891 | TAF9 RNA polymerase II, TATA box binding protein (TBP)-associated factor, 32kDa (TAF9), transcript variant 3, mRNA [NM_001015891] |
| XLOC_002471 | TCONS_00004561 | linc|BROAD Institute lincRNA (XLOC_002471), lincRNA [TCONS_00004561] |
| RNF14 | NM_004290 | ring finger protein 14 (RNF14), transcript variant 1, mRNA [NM_004290] |
| SMAD4 | NM_005359 | SMAD family member 4 (SMAD4), mRNA [NM_005359] |
| DNAJC27 | NM_016544 | DnaJ (Hsp40) homolog, subfamily C, member 27 (DNAJC27), transcript variant 1, mRNA [NM_016544] |
| ENST00000399576 | ENST00000399576 | ens|Uncharacterized protein [Source:UniProtKB/TrEMBL;Acc:E7EQU4] [ENST00000399576] |
| GPAM | ENST00000369425 | ens|glycerol-3-phosphate acyltransferase, mitochondrial [Source:HGNC Symbol;Acc:24865] [ENST00000369425] |
| RBBP6 | NM_032626 | retinoblastoma binding protein 6 (RBBP6), transcript variant 3, mRNA [NM_032626] |
| FUT10 | NM_032664 | fucosyltransferase 10 (alpha (1,3) fucosyltransferase) (FUT10), mRNA [NM_032664] |
| LOC441455 | NR_026792 | makorin ring finger protein 1 pseudogene (LOC441455), non-coding RNA [NR_026792] |
| WAC | NM_016628 | WW domain containing adaptor with coiled-coil (WAC), transcript variant 1, mRNA [NM_016628] |
| LOC100652970 | XM_003403442 | ref|PREDICTED: Homo sapiens putative IQ and AAA domain-containing protein 1-like (LOC100652970), mRNA [XM_003403442] |
| SNORA61 | NR_002987 | small nucleolar RNA, H/ACA box 61 (SNORA61), small nucleolar RNA [NR_002987] |
| XLOC_012079 | TCONS_00025273 | linc|BROAD Institute lincRNA (XLOC_012079), lincRNA [TCONS_00025273] |
| SNORD18B | NR_002442 | small nucleolar RNA, C/D box 18B (SNORD18B), small nucleolar RNA [NR_002442] |
| ANKRD17 | NM_032217 | ankyrin repeat domain 17 (ANKRD17), transcript variant 1, mRNA [NM_032217] |
| ZNF416 | NM_017879 | zinc finger protein 416 (ZNF416), mRNA [NM_017879] |
| NMI | NM_004688 | N-myc (and STAT) interactor (NMI), mRNA [NM_004688] |
| XLOC_008503 | TCONS_00018225 | linc|BROAD Institute lincRNA (XLOC_008503), lincRNA [TCONS_00018225] |
| DDX50 | NM_024045 | DEAD (Asp-Glu-Ala-Asp) box polypeptide 50 (DDX50), mRNA [NM_024045] |
| MFAP1 | NM_005926 | microfibrillar-associated protein 1 (MFAP1), mRNA [NM_005926] |
| WDSUB1 | NM_152528 | WD repeat, sterile alpha motif and U-box domain containing 1 (WDSUB1), transcript variant 3, mRNA [NM_152528] |
| LRRC37BP1 | NR_015341 | leucine rich repeat containing 37B pseudogene 1 (LRRC37BP1), non-coding RNA [NR_015341] |
| ZNF41 | NM_153380 | zinc finger protein 41 (ZNF41), transcript variant 2, mRNA [NM_153380] |
| TET1 | NM_030625 | tet methylcytosine dioxygenase 1 (TET1), mRNA [NM_030625] |
| RASGEF1B | NM_152545 | RasGEF domain family, member 1B (RASGEF1B), mRNA [NM_152545] |
| ENST00000376775 | ENST00000376775 | ens|Uncharacterized proteincDNA FLJ26048 fis, clone PRS02384 [Source:UniProtKB/TrEMBL;Acc:Q6ZPC8] [ENST00000376775] |
| C4orf41 | NM_199053 | chromosome 4 open reading frame 41 (C4orf41), transcript variant 2, mRNA [NM_199053] |
| MTERFD2 | NM_182501 | MTERF domain containing 2 (MTERFD2), transcript variant 1, mRNA [NM_182501] |
| POLR3B | NM_018082 | polymerase (RNA) III (DNA directed) polypeptide B (POLR3B), transcript variant 1, mRNA [NM_018082] |
| ZNF564 | NM_144976 | zinc finger protein 564 (ZNF564), mRNA [NM_144976] |
| PCMTD2 | ENST00000299468 | tc|HSU37230 ribosomal protein L23a {Homo sapiens} (exp=-1; wgp=0; cg=0), partial (46%) [THC2591738] |
| TRDMT1 | NM_004412 | tRNA aspartic acid methyltransferase 1 (TRDMT1), mRNA [NM_004412] |
| ZNF567 | NM_152603 | zinc finger protein 567 (ZNF567), mRNA [NM_152603] |
| FAM18B2 | NM_145301 | family with sequence similarity 18, member B2 (FAM18B2), transcript variant 1, mRNA [NM_145301] |
| TTC3 | NM_003316 | tetratricopeptide repeat domain 3 (TTC3), transcript variant 1, mRNA [NM_003316] |
| ZBTB37 | NM_032522 | zinc finger and BTB domain containing 37 (ZBTB37), transcript variant 2, mRNA [NM_032522] |
| TMEM87A | NM_015497 | transmembrane protein 87A (TMEM87A), transcript variant 1, mRNA [NM_015497] |
| ZNF45 | NM_003425 | zinc finger protein 45 (ZNF45), mRNA [NM_003425] |
| NAP1L2 | NM_021963 | nucleosome assembly protein 1-like 2 (NAP1L2), mRNA [NM_021963] |
| RABGAP1L | NM_001243765 | RAB GTPase activating protein 1-like (RABGAP1L), transcript variant 4, mRNA [NM_001243765] |
| METTL2B | NM_018396 | methyltransferase like 2B (METTL2B), mRNA [NM_018396] |
| THC2560329 | THC2560329 | tc|Q71V99_HUMAN (Q71V99) Cyclophilin , partial (12%) [THC2560329] |
| TLE4 | ENST00000461758 | ens|transducin-like enhancer of split 4 (E(sp1) homolog, Drosophila) [Source:HGNC Symbol;Acc:11840] [ENST00000461758] |
| LOC100507280 | XR_110418 | ref|PREDICTED: Homo sapiens hypothetical LOC100507280 (LOC100507280), miscRNA [XR_110418] |
| MCM9 | NM_017696 | minichromosome maintenance complex component 9 (MCM9), transcript variant 1, mRNA [NM_017696] |
| SNW1 | NM_012245 | SNW domain containing 1 (SNW1), mRNA [NM_012245] |
| PARD3 | NM_001184792 | par-3 partitioning defective 3 homolog (C. elegans) (PARD3), transcript variant 9, mRNA [NM_001184792] |
| PPM1B | NM_001033556 | protein phosphatase, Mg2+/Mn2+ dependent, 1B (PPM1B), transcript variant 4, mRNA [NM_001033556] |
| SPATA13 | NM_153023 | spermatogenesis associated 13 (SPATA13), transcript variant 2, mRNA [NM_153023] |
| SLC16A6 | NM_004694 | solute carrier family 16, member 6 (monocarboxylic acid transporter 7) (SLC16A6), transcript variant 2, mRNA [NM_004694] |
| C1orf131 | NM_152379 | chromosome 1 open reading frame 131 (C1orf131), mRNA [NM_152379] |
| XLOC_l2_012432 | DB051956 | gb|DB051956 TESTI2 Homo sapiens cDNA clone TESTI2042017 5', mRNA sequence [DB051956] |
| RNU11 | NR_004407 | RNA, U11 small nuclear (RNU11), small nuclear RNA [NR_004407] |
| LRRC8B | NM_015350 | leucine rich repeat containing 8 family, member B (LRRC8B), transcript variant 1, mRNA [NM_015350] |
| ZNF586 | NM_017652 | zinc finger protein 586 (ZNF586), transcript variant 1, mRNA [NM_017652] |
| RNF19B | NM_153341 | ring finger protein 19B (RNF19B), transcript variant 1, mRNA [NM_153341] |
| IFI44 | NM_006417 | interferon-induced protein 44 (IFI44), mRNA [NM_006417] |
| human | CR601050 | gb|full-length cDNA clone CS0DF024YK11 of Fetal brain of Homo sapiens (human) [CR601050] |
| SYNCRIP | NM_006372 | synaptotagmin binding, cytoplasmic RNA interacting protein (SYNCRIP), transcript variant 1, mRNA [NM_006372] |
| HMBOX1 | NM_024567 | homeobox containing 1 (HMBOX1), transcript variant 1, mRNA [NM_024567] |
| SMC4 | NM_005496 | structural maintenance of chromosomes 4 (SMC4), transcript variant 1, mRNA [NM_005496] |
| THC2591311 | THC2591311 | tc|NP1L1_HUMAN (P55209) Nucleosome assembly protein 1-like 1 (NAP-1-related protein) (hNRP), partial (38%) [THC2591311] |
| THC2686131 | THC2686131 | Unknown |
| SNORD12B | NR_003695 | small nucleolar RNA, C/D box 12B (SNORD12B), small nucleolar RNA [NR_003695] |
| LOC100652786 | XR_132582 | ref|PREDICTED: Homo sapiens hypothetical LOC100652786 (LOC100652786), miscRNA [XR_132582] |
| ZNF606 | NM_025027 | zinc finger protein 606 (ZNF606), mRNA [NM_025027] |
| XLOC_l2_000010 | ENST00000457084 | tc|S72008 CDC10 homolog {Homo sapiens} (exp=-1; wgp=0; cg=0), partial (9%) [THC2551621] |
| ERG | NM_004449 | v-ets erythroblastosis virus E26 oncogene homolog (avian) (ERG), transcript variant 2, mRNA [NM_004449] |
| PDXDC1 | NM_015027 | pyridoxal-dependent decarboxylase domain containing 1 (PDXDC1), mRNA [NM_015027] |
| H3F3B | NM_005324 | H3 histone, family 3B (H3.3B) (H3F3B), mRNA [NM_005324] |
| XLOC_002779 | BC035247 | gb|AGENCOURT_6855839 NIH_MGC_47 Homo sapiens cDNA clone IMAGE:5923453 5', mRNA sequence [BQ070964] |
| PSME4 | NM_014614 | proteasome (prosome, macropain) activator subunit 4 (PSME4), mRNA [NM_014614] |
| MAP4 | NM_001134364 | microtubule-associated protein 4 (MAP4), transcript variant 4, mRNA [NM_001134364] |
| AASDH | NM_181806 | aminoadipate-semialdehyde dehydrogenase (AASDH), mRNA [NM_181806] |
| TRIM37 | NM_001005207 | tripartite motif containing 37 (TRIM37), transcript variant 2, mRNA [NM_001005207] |
| SNORD12C | NR_002433 | small nucleolar RNA, C/D box 12C (SNORD12C), small nucleolar RNA [NR_002433] |
| XLOC_008467 | ENST00000442526 | tc|ALU1_HUMAN (P39188) Alu subfamily J sequence contamination warning entry, partial (15%) [THC2667127] |
| IPP | NM_005897 | intracisternal A particle-promoted polypeptide (IPP), transcript variant 1, mRNA [NM_005897] |
| GALK2 | NM_001001556 | galactokinase 2 (GALK2), transcript variant 2, mRNA [NM_001001556] |
| PET117 | NM_001164811 | cytochrome c oxidase assembly factor-like (PET117), mRNA [NM_001164811] |
| A_33_P3338539 | A_33_P3338539 | Unknown |
| BRWD1 | NM_033656 | bromodomain and WD repeat domain containing 1 (BRWD1), transcript variant 2, mRNA [NM_033656] |
| HCG18 | NR_024052 | HLA complex group 18 (non-protein coding) (HCG18), non-coding RNA [NR_024052] |
| RNASEH1 | NM_002936 | ribonuclease H1 (RNASEH1), mRNA [NM_002936] |
| ITGBL1 | NM_004791 | integrin, beta-like 1 (with EGF-like repeat domains) (ITGBL1), mRNA [NM_004791] |
| ZNF761 | NM_001008401 | zinc finger protein 761 (ZNF761), mRNA [NM_001008401] |
| ALDH9A1 | NM_000696 | aldehyde dehydrogenase 9 family, member A1 (ALDH9A1), mRNA [NM_000696] |
| LINC00235 | NR_024121 | long intergenic non-protein coding RNA 235 (LINC00235), non-coding RNA [NR_024121] |
| ACSL1 | NM_001995 | acyl-CoA synthetase long-chain family member 1 (ACSL1), mRNA [NM_001995] |
| FTSJ2 | NM_013393 | FtsJ homolog 2 (E. coli) (FTSJ2), mRNA [NM_013393] |
| C20orf94 | NM_001009608 | chromosome 20 open reading frame 94 (C20orf94), mRNA [NM_001009608] |
| AQR | ENST00000156471 | ens|aquarius homolog (mouse) [Source:HGNC Symbol;Acc:29513] [ENST00000156471] |
| PRAMEF3 | NM_001013692 | PRAME family member 3 (PRAMEF3), mRNA [NM_001013692] |
| NARG2 | NM_024611 | NMDA receptor regulated 2 (NARG2), transcript variant 1, mRNA [NM_024611] |
| ADPGK | NM_031284 | ADP-dependent glucokinase (ADPGK), transcript variant 1, mRNA [NM_031284] |
| DKFZP586I1420 | NR_002186 | uncharacterized protein DKFZp586I1420 (DKFZP586I1420), non-coding RNA [NR_002186] |
| ZBTB8A | NM_001040441 | zinc finger and BTB domain containing 8A (ZBTB8A), mRNA [NM_001040441] |
| REL | NM_002908 | v-rel reticuloendotheliosis viral oncogene homolog (avian) (REL), mRNA [NM_002908] |
| IL6ST | NM_001190981 | interleukin 6 signal transducer (gp130, oncostatin M receptor) (IL6ST), transcript variant 3, mRNA [NM_001190981] |
| TTC32 | NM_001008237 | tetratricopeptide repeat domain 32 (TTC32), mRNA [NM_001008237] |
| PPTC7 | NM_139283 | PTC7 protein phosphatase homolog (S. cerevisiae) (PPTC7), mRNA [NM_139283] |
| TCF4 | NM_003199 | transcription factor 4 (TCF4), transcript variant 2, mRNA [NM_003199] |
| C2orf15 | NM_144706 | chromosome 2 open reading frame 15 (C2orf15), mRNA [NM_144706] |
| STRN | NM_003162 | striatin, calmodulin binding protein (STRN), mRNA [NM_003162] |
| A_33_P3298980 | A_33_P3298980 | Unknown |
| XLOC_006664 | TCONS_00014020 | linc|BROAD Institute lincRNA (XLOC_006664), lincRNA [TCONS_00014020] |
| HIST2H2BE | NM_003528 | histone cluster 2, H2be (HIST2H2BE), mRNA [NM_003528] |
| PLAA | NM_001031689 | phospholipase A2-activating protein (PLAA), mRNA [NM_001031689] |
| PSMC2 | NM_002803 | proteasome (prosome, macropain) 26S subunit, ATPase, 2 (PSMC2), transcript variant 1, mRNA [NM_002803] |
| SMG1 | NM_015092 | smg-1 homolog, phosphatidylinositol 3-kinase-related kinase (C. elegans) (SMG1), mRNA [NM_015092] |
| NFX1 | NM_147133 | nuclear transcription factor, X-box binding 1 (NFX1), transcript variant 2, mRNA [NM_147133] |
| C6orf132 | NM_001164446 | chromosome 6 open reading frame 132 (C6orf132), mRNA [NM_001164446] |
| UXS1 | NM_025076 | UDP-glucuronate decarboxylase 1 (UXS1), mRNA [NM_025076] |
| ZBTB2 | NM_020861 | zinc finger and BTB domain containing 2 (ZBTB2), mRNA [NM_020861] |
| LOC100289455 | XR_109018 | ref|PREDICTED: Homo sapiens hypothetical LOC100289455 (LOC100289455), miscRNA [XR_109018] |
| FBXL19-AS1 | NR_024348 | FBXL19 antisense RNA 1 (non-protein coding) (FBXL19-AS1), non-coding RNA [NR_024348] |
| ANGEL2 | NM_144567 | angel homolog 2 (Drosophila) (ANGEL2), mRNA [NM_144567] |
| GTF2I | NM_032999 | general transcription factor IIi (GTF2I), transcript variant 1, mRNA [NM_032999] |
| FGF17 | NM_003867 | fibroblast growth factor 17 (FGF17), mRNA [NM_003867] |
| SCARNA23 | NR_003007 | small Cajal body-specific RNA 23 (SCARNA23), guide RNA [NR_003007] |
| OXNAD1 | NM_138381 | oxidoreductase NAD-binding domain containing 1 (OXNAD1), mRNA [NM_138381] |
| A_33_P3283206 | A_33_P3283206 | Unknown |
| ASTE1 | NM_014065 | asteroid homolog 1 (Drosophila) (ASTE1), mRNA [NM_014065] |
| LIN28A | NM_024674 | lin-28 homolog A (C. elegans) (LIN28A), mRNA [NM_024674] |
| IP6K2 | NM_016291 | inositol hexakisphosphate kinase 2 (IP6K2), transcript variant 1, mRNA [NM_016291] |
| GTF2A2 | NM_004492 | general transcription factor IIA, 2, 12kDa (GTF2A2), mRNA [NM_004492] |
| BTN2A1 | NM_001197234 | butyrophilin, subfamily 2, member A1 (BTN2A1), transcript variant 4, mRNA [NM_001197234] |
| MBTD1 | NM_017643 | mbt domain containing 1 (MBTD1), mRNA [NM_017643] |
| PLCB1 | NM_015192 | phospholipase C, beta 1 (phosphoinositide-specific) (PLCB1), transcript variant 1, mRNA [NM_015192] |
| PRKRIR | NM_004705 | protein-kinase, interferon-inducible double stranded RNA dependent inhibitor, repressor of (P58 repressor) (PRKRIR), mRNA [NM_004705] |
| FCER2 | NM_002002 | Fc fragment of IgE, low affinity II, receptor for (CD23) (FCER2), transcript variant 1, mRNA [NM_002002] |
| PAK1IP1 | NM_017906 | PAK1 interacting protein 1 (PAK1IP1), mRNA [NM_017906] |
| BTAF1 | NM_003972 | BTAF1 RNA polymerase II, B-TFIID transcription factor-associated, 170kDa (Mot1 homolog, S. cerevisiae) (BTAF1), mRNA [NM_003972] |
| ZNF394 | NM_032164 | zinc finger protein 394 (ZNF394), mRNA [NM_032164] |
| XLOC_l2_007456 | TCONS_l2_00013854 | linc|BROAD Institute lincRNA (XLOC_l2_007456), lincRNA [TCONS_l2_00013854] |
| LOC100128811 | NR_027333 | uncharacterized LOC100128811 (LOC100128811), non-coding RNA [NR_027333] |
| ZNF620 | NM_175888 | zinc finger protein 620 (ZNF620), transcript variant 1, mRNA [NM_175888] |
| DDX55 | NM_020936 | DEAD (Asp-Glu-Ala-Asp) box polypeptide 55 (DDX55), mRNA [NM_020936] |
| XLOC_008382 | TCONS_00018121 | linc|BROAD Institute lincRNA (XLOC_008382), lincRNA [TCONS_00018121] |
| ZNF124 | NM_003431 | zinc finger protein 124 (ZNF124), transcript variant 1, mRNA [NM_003431] |
| ZWINT | NM_032997 | ZW10 interactor (ZWINT), transcript variant 2, mRNA [NM_032997] |
| ZNF468 | NM_199132 | zinc finger protein 468 (ZNF468), transcript variant 1, mRNA [NM_199132] |
| FAM45A | NM_207009 | family with sequence similarity 45, member A (FAM45A), mRNA [NM_207009] |
| TRIM43 | NM_138800 | tripartite motif containing 43 (TRIM43), mRNA [NM_138800] |
| BCL2 | NM_000657 | B-cell CLL/lymphoma 2 (BCL2), nuclear gene encoding mitochondrial protein, transcript variant beta, mRNA [NM_000657] |
| HNRNPUL2 | NM_001079559 | heterogeneous nuclear ribonucleoprotein U-like 2 (HNRNPUL2), mRNA [NM_001079559] |
| ENST00000390602 | ENST00000390602 | ens|immunoglobulin heavy variable 3-13 [Source:HGNC Symbol;Acc:5581] [ENST00000390602] |
| VAMP3 | NM_004781 | vesicle-associated membrane protein 3 (cellubrevin) (VAMP3), mRNA [NM_004781] |
| SNORD60 | NR_002736 | small nucleolar RNA, C/D box 60 (SNORD60), small nuclear RNA [NR_002736] |
| CDX4 | NM_005193 | caudal type homeobox 4 (CDX4), mRNA [NM_005193] |
| ZNF195 | NM_007152 | zinc finger protein 195 (ZNF195), transcript variant 3, mRNA [NM_007152] |
| ASAP1-IT1 | NR_002765 | ASAP1 intronic transcript 1 (non-protein coding) (ASAP1-IT1), non-coding RNA [NR_002765] |
| ZNF643 | NM_023070 | zinc finger protein 643 (ZNF643), mRNA [NM_023070] |
| A_33_P3300179 | A_33_P3300179 | Unknown |
| SHCBP1 | NM_024745 | SHC SH2-domain binding protein 1 (SHCBP1), mRNA [NM_024745] |
| ZNF816 | NM_001031665 | zinc finger protein 816 (ZNF816), transcript variant 1, mRNA [NM_001031665] |
| CDR2 | NM_001802 | cerebellar degeneration-related protein 2, 62kDa (CDR2), mRNA [NM_001802] |
| PIAS1 | NM_016166 | protein inhibitor of activated STAT, 1 (PIAS1), mRNA [NM_016166] |
| CCT2 | NM_006431 | chaperonin containing TCP1, subunit 2 (beta) (CCT2), transcript variant 1, mRNA [NM_006431] |
| LOC645954 | XR_109026 | ref|PREDICTED: Homo sapiens supervillin pseudogene (LOC645954), miscRNA [XR_109026] |
| ARAP1-AS2 | ENST00000500163 | ref|PREDICTED: Homo sapiens ARAP1 antisense RNA 2 (non-protein coding) (ARAP1-AS2), miscRNA [XR_110891] |
| PRTFDC1 | NM_020200 | phosphoribosyl transferase domain containing 1 (PRTFDC1), mRNA [NM_020200] |
| FAM133B | NM_001040057 | family with sequence similarity 133, member B (FAM133B), transcript variant 2, mRNA [NM_001040057] |
| PDE4DIP | NM_022359 | phosphodiesterase 4D interacting protein (PDE4DIP), transcript variant 3, mRNA [NM_022359] |
| DCTN6 | NM_006571 | dynactin 6 (DCTN6), mRNA [NM_006571] |
| AADACL3 | NM_001103170 | arylacetamide deacetylase-like 3 (AADACL3), transcript variant 1, mRNA [NM_001103170] |
| GREB1L | NM_001142966 | growth regulation by estrogen in breast cancer-like (GREB1L), mRNA [NM_001142966] |
| LOC100506935 | XR_108684 | ref|PREDICTED: Homo sapiens hypothetical LOC100506935 (LOC100506935), miscRNA [XR_108684] |
| CDK7 | NM_001799 | cyclin-dependent kinase 7 (CDK7), mRNA [NM_001799] |
| CR997556 | CR997556 | gb|CR997556 RZPD no.9016 Homo sapiens cDNA clone RZPDp9016I2412 5', mRNA sequence [CR997556] |
| XLOC_l2_009050 | TCONS_l2_00017027 | linc|BROAD Institute lincRNA (XLOC_l2_009050), lincRNA [TCONS_l2_00017027] |
| NCOR1 | NM_006311 | nuclear receptor corepressor 1 (NCOR1), transcript variant 1, mRNA [NM_006311] |
| BRD7 | ENST00000401491 | ens|bromodomain containing 7 [Source:HGNC Symbol;Acc:14310] [ENST00000401491] |
| ORMDL1 | AK126336 | gb|Homo sapiens cDNA FLJ44364 fis, clone TRACH3008201. [AK126336] |
| ZNF658 | NM_033160 | zinc finger protein 658 (ZNF658), mRNA [NM_033160] |
| TMEM48 | NM_018087 | transmembrane protein 48 (TMEM48), transcript variant 1, mRNA [NM_018087] |
| NR2E3 | NM_014249 | nuclear receptor subfamily 2, group E, member 3 (NR2E3), transcript variant 2, mRNA [NM_014249] |
| A_33_P3673310 | A_33_P3673310 | Unknown |
| ACSBG1 | NM_015162 | acyl-CoA synthetase bubblegum family member 1 (ACSBG1), transcript variant 1, mRNA [NM_015162] |
| NT5C2 | NM_012229 | 5'-nucleotidase, cytosolic II (NT5C2), transcript variant 1, mRNA [NM_012229] |
| SNORA62 | NR_002324 | small nucleolar RNA, H/ACA box 62 (SNORA62), small nucleolar RNA [NR_002324] |
| LOC646329 | NR_034120 | uncharacterized LOC646329 (LOC646329), non-coding RNA [NR_034120] |
| POU2F1 | NM_002697 | POU class 2 homeobox 1 (POU2F1), transcript variant 1, mRNA [NM_002697] |
| DGCR11 | NR_024157 | DiGeorge syndrome critical region gene 11 (DGCR11), non-coding RNA [NR_024157] |
| DCAF6 | NM_018442 | DDB1 and CUL4 associated factor 6 (DCAF6), transcript variant 1, mRNA [NM_018442] |
| LOC100506874 | NR_038273 | uncharacterized LOC100506874 (LOC100506874), transcript variant 1, non-coding RNA [NR_038273] |
| ZFAND5 | NM_006007 | zinc finger, AN1-type domain 5 (ZFAND5), transcript variant c, mRNA [NM_006007] |
| NDNL2 | NM_138704 | necdin-like 2 (NDNL2), mRNA [NM_138704] |
| XLOC_006150 | TCONS_00014163 | linc|BROAD Institute lincRNA (XLOC_006150), lincRNA [TCONS_00014163] |
| C19orf68 | ENST00000328759 | ref|PREDICTED: Homo sapiens chromosome 19 open reading frame 68 (C19orf68), mRNA [XM_001713770] |
| TADA1 | NM_053053 | transcriptional adaptor 1 (TADA1), mRNA [NM_053053] |
| AGXT | NM_000030 | alanine-glyoxylate aminotransferase (AGXT), mRNA [NM_000030] |
| NP106769 | NP106769 | tc|GB|AC004460.1|AAC06339.1 similar to golgi antigen; similar to U50078 (PID:g1477565) [NP106769] |
| FAM59A | NM_022751 | family with sequence similarity 59, member A (FAM59A), transcript variant 2, mRNA [NM_022751] |
| BARD1 | NM_000465 | BRCA1 associated RING domain 1 (BARD1), mRNA [NM_000465] |
| ZNF667 | NM_022103 | zinc finger protein 667 (ZNF667), transcript variant 1, mRNA [NM_022103] |
| PINX1 | NM_017884 | PIN2/TERF1 interacting, telomerase inhibitor 1 (PINX1), mRNA [NM_017884] |
| C6orf204 | NM_206921 | chromosome 6 open reading frame 204 (C6orf204), transcript variant 2, mRNA [NM_206921] |
| RPS4X | ENST00000373626 | ens|ribosomal protein S4, X-linked [Source:HGNC Symbol;Acc:10424] [ENST00000373626] |
| XLOC_l2_000297 | THC2520127 | tc|Q5D1D6_CERAE (Q5D1D6) Guanylate binding protein 1, partial (31%) [THC2520127] |
| ZNF549 | NM_153263 | zinc finger protein 549 (ZNF549), transcript variant 2, mRNA [NM_153263] |
| IPO7 | NM_006391 | importin 7 (IPO7), mRNA [NM_006391] |
| C2orf74 | NM_001143959 | chromosome 2 open reading frame 74 (C2orf74), transcript variant 1, mRNA [NM_001143959] |
| PGM3 | NM_001199919 | phosphoglucomutase 3 (PGM3), transcript variant 4, mRNA [NM_001199919] |
| RBM39 | NM_184234 | RNA binding motif protein 39 (RBM39), transcript variant 1, mRNA [NM_184234] |
| SNORA36A | NR_002969 | small nucleolar RNA, H/ACA box 36A (SNORA36A), small nucleolar RNA [NR_002969] |
| PIK3CB | NM_006219 | phosphoinositide-3-kinase, catalytic, beta polypeptide (PIK3CB), mRNA [NM_006219] |
| TOPORS | NM_005802 | topoisomerase I binding, arginine/serine-rich, E3 ubiquitin protein ligase (TOPORS), transcript variant 1, mRNA [NM_005802] |
| WRN | NM_000553 | Werner syndrome, RecQ helicase-like (WRN), mRNA [NM_000553] |
| MRPS23 | NM_016070 | mitochondrial ribosomal protein S23 (MRPS23), nuclear gene encoding mitochondrial protein, mRNA [NM_016070] |
| SNORA46 | NR_002978 | small nucleolar RNA, H/ACA box 46 (SNORA46), small nucleolar RNA [NR_002978] |
| BBX | NM_020235 | bobby sox homolog (Drosophila) (BBX), transcript variant 2, mRNA [NM_020235] |
| RPA4 | NM_013347 | replication protein A4, 30kDa (RPA4), mRNA [NM_013347] |
| CWC15 | NM_016403 | CWC15 spliceosome-associated protein homolog (S. cerevisiae) (CWC15), mRNA [NM_016403] |
| ENST00000358739 | ENST00000358739 | ens|histone cluster 1, H2ai [Source:HGNC Symbol;Acc:4725] [ENST00000358739] |
| INTS4 | NM_033547 | integrator complex subunit 4 (INTS4), mRNA [NM_033547] |
| JAKMIP2 | NM_014790 | janus kinase and microtubule interacting protein 2 (JAKMIP2), mRNA [NM_014790] |
| LOC100653004 | XR_132505 | ref|PREDICTED: Homo sapiens hypothetical LOC100653004 (LOC100653004), miscRNA [XR_132505] |
| BRD3 | NM_007371 | bromodomain containing 3 (BRD3), mRNA [NM_007371] |
| AK123993 | AK123993 | gb|Homo sapiens cDNA FLJ41999 fis, clone SPLEN2029683. [AK123993] |
| XLOC_000044 | ENST00000442636 | gb|AGENCOURT_10396508 NIH_MGC_141 Homo sapiens cDNA clone IMAGE:6605580 5', mRNA sequence [BU566406] |
| XLOC_l2_015585 | TCONS_l2_00030301 | linc|BROAD Institute lincRNA (XLOC_l2_015585), lincRNA [TCONS_l2_00030301] |
| ENST00000450721 | ENST00000450721 | ens|Uncharacterized proteincDNA FLJ40589 fis, clone THYMU2009596 [Source:UniProtKB/TrEMBL;Acc:Q8N1K4] [ENST00000450721] |
| DCAF5 | BC022967 | gb|Homo sapiens WD repeat domain 22, mRNA (cDNA clone IMAGE:5111152), complete cds. [BC022967] |
| ZC3H13 | NM_015070 | zinc finger CCCH-type containing 13 (ZC3H13), mRNA [NM_015070] |
| PBX3 | NM_006195 | pre-B-cell leukemia homeobox 3 (PBX3), transcript variant 1, mRNA [NM_006195] |
| BHMT | NM_001713 | betaine--homocysteine S-methyltransferase (BHMT), mRNA [NM_001713] |
| FAM108B1 | NM_016014 | family with sequence similarity 108, member B1 (FAM108B1), transcript variant 1, mRNA [NM_016014] |
| CMAHP | NR_002174 | cytidine monophospho-N-acetylneuraminic acid hydroxylase, pseudogene (CMAHP), transcript variant 1, non-coding RNA [NR_002174] |
| AK130343 | AK130343 | gb|Homo sapiens cDNA FLJ26833 fis, clone PRS07140. [AK130343] |
| BC029571 | BC029571 | gb|Homo sapiens cDNA clone IMAGE:5262686. [BC029571] |
| MAP7D3 | NM_024597 | MAP7 domain containing 3 (MAP7D3), transcript variant 1, mRNA [NM_024597] |
| UMPS | NM_000373 | uridine monophosphate synthetase (UMPS), transcript variant 1, mRNA [NM_000373] |
| MINPP1 | NM_004897 | multiple inositol-polyphosphate phosphatase 1 (MINPP1), transcript variant 1, mRNA [NM_004897] |
| YPLR6490 | XR_110047 | ref|PREDICTED: Homo sapiens hypothetical LOC389102 (YPLR6490), miscRNA [XR_110047] |
| LOC100128281 | AK128353 | gb|Homo sapiens cDNA FLJ46495 fis, clone THYMU3028461. [AK128353] |
| POLE2 | NM_002692 | polymerase (DNA directed), epsilon 2 (p59 subunit) (POLE2), transcript variant 1, mRNA [NM_002692] |
| CCNT1 | NM_001240 | cyclin T1 (CCNT1), mRNA [NM_001240] |
| TDRD6 | NM_001010870 | tudor domain containing 6 (TDRD6), transcript variant 1, mRNA [NM_001010870] |
| XLOC_005900 | ENST00000430078 | tc|Q6CC74_YARLI (Q6CC74) Similarity, partial (3%) [THC2487722] |
| MAK16 | NM_032509 | MAK16 homolog (S. cerevisiae) (MAK16), mRNA [NM_032509] |
| ECD | NM_007265 | ecdysoneless homolog (Drosophila) (ECD), transcript variant 1, mRNA [NM_007265] |
| ZFX | NM_003410 | zinc finger protein, X-linked (ZFX), transcript variant 1, mRNA [NM_003410] |
| DNAJC14 | NM_032364 | DnaJ (Hsp40) homolog, subfamily C, member 14 (DNAJC14), mRNA [NM_032364] |
| ZNF383 | NM_152604 | zinc finger protein 383 (ZNF383), mRNA [NM_152604] |
| RECQL5 | ENST00000420326 | ens|RecQ protein-like 5 [Source:HGNC Symbol;Acc:9950] [ENST00000420326] |
| A_33_P3240966 | A_33_P3240966 | Unknown |
| CACYBP | NM_014412 | calcyclin binding protein (CACYBP), transcript variant 1, mRNA [NM_014412] |
| PCNA-AS1 | NR_028370 | PCNA antisense RNA 1 (non-protein coding) (PCNA-AS1), non-coding RNA [NR_028370] |
| XLOC_014336 | THC2660954 | linc|BROAD Institute lincRNA (XLOC_014336), lincRNA [TCONS_00029887] |
| KLHL28 | NM_017658 | kelch-like 28 (Drosophila) (KLHL28), mRNA [NM_017658] |
| SRSF8 | NM_032102 | serine/arginine-rich splicing factor 8 (SRSF8), mRNA [NM_032102] |
| MLLT4 | NM_001207008 | myeloid/lymphoid or mixed-lineage leukemia (trithorax homolog, Drosophila); translocated to, 4 (MLLT4), transcript variant 1, mRNA [NM_001207008] |
| MMADHC | NM_015702 | methylmalonic aciduria (cobalamin deficiency) cblD type, with homocystinuria (MMADHC), nuclear gene encoding mitochondrial protein, mRNA [NM_015702] |
| XLOC_012467 | TCONS_00026069 | linc|BROAD Institute lincRNA (XLOC_012467), lincRNA [TCONS_00026069] |
| SNORD83A | NR_000027 | small nucleolar RNA, C/D box 83A (SNORD83A), small nucleolar RNA [NR_000027] |
| ZNF180 | NM_013256 | zinc finger protein 180 (ZNF180), mRNA [NM_013256] |
| ZNF20 | NM_021143 | zinc finger protein 20 (ZNF20), transcript variant 1, mRNA [NM_021143] |
| A_33_P3221318 | A_33_P3221318 | Unknown |
| SNORD97 | NR_004403 | small nucleolar RNA, C/D box 97 (SNORD97), small nucleolar RNA [NR_004403] |
| PDSS1 | NM_014317 | prenyl (decaprenyl) diphosphate synthase, subunit 1 (PDSS1), mRNA [NM_014317] |
| ZBTB10 | NM_001105539 | zinc finger and BTB domain containing 10 (ZBTB10), transcript variant 1, mRNA [NM_001105539] |
| CHMP4B | NM_176812 | charged multivesicular body protein 4B (CHMP4B), mRNA [NM_176812] |
| XLOC_008001 | CN284311 | gb|17000531326764 GRN_ES Homo sapiens cDNA 5', mRNA sequence [CN284311] |
| NCOA2 | NM_006540 | nuclear receptor coactivator 2 (NCOA2), mRNA [NM_006540] |
| APOL6 | NM_030641 | apolipoprotein L, 6 (APOL6), mRNA [NM_030641] |
| XLOC_004901 | BX109598 | gb|BX109598 NCI_CGAP_Lym12 Homo sapiens cDNA clone IMAGp998G195987, mRNA sequence [BX109598] |
| C9orf102 | NM_001010895 | chromosome 9 open reading frame 102 (C9orf102), mRNA [NM_001010895] |
| MAP3K4 | NM_006724 | mitogen-activated protein kinase kinase kinase 4 (MAP3K4), transcript variant 2, mRNA [NM_006724] |
| XLOC_006304 | ENST00000418523 | tc|COX1_DINSE (O79548) Cytochrome c oxidase subunit 1 (Cytochrome c oxidase polypeptide I) , partial (4%) [THC2694836] |
| C12orf45 | NM_152318 | chromosome 12 open reading frame 45 (C12orf45), mRNA [NM_152318] |
| BCL2L13 | NM_015367 | BCL2-like 13 (apoptosis facilitator) (BCL2L13), nuclear gene encoding mitochondrial protein, mRNA [NM_015367] |
| MRPL15 | NM_014175 | mitochondrial ribosomal protein L15 (MRPL15), nuclear gene encoding mitochondrial protein, mRNA [NM_014175] |
| EXOC6B | ENST00000272427 | ens|exocyst complex component 6B [Source:HGNC Symbol;Acc:17085] [ENST00000272427] |
| RANBP9 | NM_005493 | RAN binding protein 9 (RANBP9), mRNA [NM_005493] |
| TRIM13 | NM_213590 | tripartite motif containing 13 (TRIM13), transcript variant 3, mRNA [NM_213590] |
| PTRH2 | NM_016077 | peptidyl-tRNA hydrolase 2 (PTRH2), nuclear gene encoding mitochondrial protein, mRNA [NM_016077] |
| SNORD1B | NR_004396 | small nucleolar RNA, C/D box 1B (SNORD1B), small nucleolar RNA [NR_004396] |
| PUS3 | NM_031307 | pseudouridylate synthase 3 (PUS3), mRNA [NM_031307] |
| ZNF281 | NM_012482 | zinc finger protein 281 (ZNF281), mRNA [NM_012482] |
| GNAI3 | NM_006496 | guanine nucleotide binding protein (G protein), alpha inhibiting activity polypeptide 3 (GNAI3), mRNA [NM_006496] |
| CCDC25 | NM_018246 | coiled-coil domain containing 25 (CCDC25), mRNA [NM_018246] |
| FAM168A | NM_015159 | family with sequence similarity 168, member A (FAM168A), mRNA [NM_015159] |
| ECT2 | NM_018098 | epithelial cell transforming sequence 2 oncogene (ECT2), mRNA [NM_018098] |
| XLOC_l2_004640 | TCONS_l2_00008608 | linc|BROAD Institute lincRNA (XLOC_l2_004640), lincRNA [TCONS_l2_00008608] |
| CD99P1 | NR_033380 | CD99 molecule pseudogene 1 (CD99P1), transcript variant 1, non-coding RNA [NR_033380] |
| XLOC_003661 | TCONS_00007603 | linc|BROAD Institute lincRNA (XLOC_003661), lincRNA [TCONS_00007603] |
| BAZ1B | NM_032408 | bromodomain adjacent to zinc finger domain, 1B (BAZ1B), mRNA [NM_032408] |
| ZNF34 | NM_030580 | zinc finger protein 34 (ZNF34), mRNA [NM_030580] |
| XLOC_004679 | ENST00000507681 | tc|Q8HN55_BRUMA (Q8HN55) NADH dehydrogenase subunit 2, partial (5%) [THC2667709] |
| LOC646778 | XR_132648 | ref|PREDICTED: Homo sapiens hypothetical LOC646778 (LOC646778), miscRNA [XR_132648] |
| ALAD | NM_000031 | aminolevulinate dehydratase (ALAD), mRNA [NM_000031] |
| CENPN | NM_001100625 | centromere protein N (CENPN), transcript variant 1, mRNA [NM_001100625] |
| NGRN | NM_001033088 | neugrin, neurite outgrowth associated (NGRN), transcript variant 2, mRNA [NM_001033088] |
| RBBP5 | NM_001193273 | retinoblastoma binding protein 5 (RBBP5), transcript variant 3, mRNA [NM_001193273] |
| RRM2 | NM_001034 | ribonucleotide reductase M2 (RRM2), transcript variant 2, mRNA [NM_001034] |
| C22orf24 | NM_015372 | chromosome 22 open reading frame 24 (C22orf24), mRNA [NM_015372] |
| SNORA14B | NR_002956 | small nucleolar RNA, H/ACA box 14B (SNORA14B), small nucleolar RNA [NR_002956] |
| LOC100506021 | XR_109692 | ref|PREDICTED: Homo sapiens hypothetical LOC100506021 (LOC100506021), miscRNA [XR_109692] |
| TMEM85 | NM_016454 | transmembrane protein 85 (TMEM85), mRNA [NM_016454] |
| TRIP12 | BC037956 | gb|Homo sapiens thyroid hormone receptor interactor 12, mRNA (cDNA clone IMAGE:5742743), with apparent retained intron. [BC037956] |
| AK092835 | AK092835 | gb|Homo sapiens cDNA FLJ35516 fis, clone SPLEN2000695. [AK092835] |
| FCHSD2 | NM_014824 | FCH and double SH3 domains 2 (FCHSD2), mRNA [NM_014824] |
| LOC729770 | XR_112442 | ref|PREDICTED: Homo sapiens hypothetical LOC729770 (LOC729770), miscRNA [XR_112442] |
| SNX3 | ENST00000368982 | ens|sorting nexin 3 [Source:HGNC Symbol;Acc:11174] [ENST00000368982] |
| XLOC_014192 | TCONS_00029542 | linc|BROAD Institute lincRNA (XLOC_014192), lincRNA [TCONS_00029542] |
| ZNF673 | NM_001129899 | zinc finger family member 673 (ZNF673), transcript variant 3, mRNA [NM_001129899] |
| TSPAN3 | NM_005724 | tetraspanin 3 (TSPAN3), transcript variant 1, mRNA [NM_005724] |
| SH3GL1P2 | NR_033420 | SH3-domain GRB2-like 1 pseudogene 2 (SH3GL1P2), non-coding RNA [NR_033420] |
| ZNF788 | NR_027049 | zinc finger family member 788 (ZNF788), non-coding RNA [NR_027049] |
| XLOC_008563 | THC2683965 | tc|Q8TAW2_HUMAN (Q8TAW2) CD99L2 protein, partial (39%) [THC2683965] |
| ZMAT2 | NM_144723 | zinc finger, matrin-type 2 (ZMAT2), mRNA [NM_144723] |
| LUZP1 | ENST00000314174 | ens|leucine zipper protein 1 [Source:HGNC Symbol;Acc:14985] [ENST00000314174] |
| C10orf111 | NM_153244 | chromosome 10 open reading frame 111 (C10orf111), mRNA [NM_153244] |
| WHAMMP2 | BC066982 | tc|Q571B6_MOUSE (Q571B6) MKIAA1971 protein (Fragment), partial (50%) [THC2554418] |
| TOB1 | NM_005749 | transducer of ERBB2, 1 (TOB1), transcript variant 1, mRNA [NM_005749] |
| SGMS2 | NM_152621 | sphingomyelin synthase 2 (SGMS2), transcript variant 1, mRNA [NM_152621] |
| NRXN3 | NM_004796 | neurexin 3 (NRXN3), transcript variant 1, mRNA [NM_004796] |
| RTN4IP1 | NM_032730 | reticulon 4 interacting protein 1 (RTN4IP1), nuclear gene encoding mitochondrial protein, mRNA [NM_032730] |
| XPA | NR_027302 | xeroderma pigmentosum, complementation group A (XPA), transcript variant 2, non-coding RNA [NR_027302] |
| EIF5 | NM_001969 | eukaryotic translation initiation factor 5 (EIF5), transcript variant 1, mRNA [NM_001969] |
| IFIT2 | NM_001547 | interferon-induced protein with tetratricopeptide repeats 2 (IFIT2), mRNA [NM_001547] |
| XLOC_l2_000720 | ENST00000424587 | ref|PREDICTED: Homo sapiens hypothetical LOC100508047 (LOC100508047), miscRNA [XR_111620] |
| FAM13B | NM_016603 | family with sequence similarity 13, member B (FAM13B), transcript variant 1, mRNA [NM_016603] |
| C4orf19 | NM_018302 | chromosome 4 open reading frame 19 (C4orf19), transcript variant 2, mRNA [NM_018302] |
| FAM111B | NM_198947 | family with sequence similarity 111, member B (FAM111B), transcript variant 1, mRNA [NM_198947] |
| CD177 | NM_020406 | CD177 molecule (CD177), mRNA [NM_020406] |
| GRIPAP1 | NM_207672 | GRIP1 associated protein 1 (GRIPAP1), transcript variant 2, mRNA [NM_207672] |
| SCARNA4 | NR_003005 | small Cajal body-specific RNA 4 (SCARNA4), guide RNA [NR_003005] |
| LINC00265 | NR_026999 | long intergenic non-protein coding RNA 265 (LINC00265), non-coding RNA [NR_026999] |
| TNPO3 | NM_012470 | transportin 3 (TNPO3), transcript variant 1, mRNA [NM_012470] |
| ZNF878 | NM_001080404 | zinc finger protein 878 (ZNF878), mRNA [NM_001080404] |
| LAMTOR3 | NM_021970 | late endosomal/lysosomal adaptor, MAPK and MTOR activator 3 (LAMTOR3), transcript variant 1, mRNA [NM_021970] |
| XLOC_003221 | BC030754 | linc|BROAD Institute lincRNA (XLOC_003221), lincRNA [TCONS_00007239] |
| XLOC_000741 | TCONS_00001425 | linc|BROAD Institute lincRNA (XLOC_000741), lincRNA [TCONS_00001425] |
| THC2511093 | THC2511093 | tc|COEA1_HUMAN (Q05707) Collagen alpha-1(XIV) chain precursor (Undulin), partial (27%) [THC2511093] |
| ZNF546 | NM_178544 | zinc finger protein 546 (ZNF546), mRNA [NM_178544] |
| MTPAP | NM_018109 | mitochondrial poly(A) polymerase (MTPAP), nuclear gene encoding mitochondrial protein, mRNA [NM_018109] |
| G3BP1 | ENST00000394123 | ens|GTPase activating protein (SH3 domain) binding protein 1 [Source:HGNC Symbol;Acc:30292] [ENST00000394123] |
| C7orf70 | NM_001037163 | chromosome 7 open reading frame 70 (C7orf70), mRNA [NM_001037163] |
| ZNF671 | NM_024833 | zinc finger protein 671 (ZNF671), mRNA [NM_024833] |
| XLOC_008451 | TCONS_00018161 | linc|BROAD Institute lincRNA (XLOC_008451), lincRNA [TCONS_00018161] |
| MRPL22 | NM_001014990 | mitochondrial ribosomal protein L22 (MRPL22), nuclear gene encoding mitochondrial protein, transcript variant 2, mRNA [NM_001014990] |
| DDX59 | NM_001031725 | DEAD (Asp-Glu-Ala-Asp) box polypeptide 59 (DDX59), mRNA [NM_001031725] |
| SNORD53 | NR_002741 | small nucleolar RNA, C/D box 53 (SNORD53), small nucleolar RNA [NR_002741] |
| LMNB1 | NM_005573 | lamin B1 (LMNB1), transcript variant 1, mRNA [NM_005573] |
| GLIPR1L2 | ENST00000378689 | ens|GLI pathogenesis-related 1 like 2 [Source:HGNC Symbol;Acc:28592] [ENST00000378689] |
| CCT8 | NM_006585 | chaperonin containing TCP1, subunit 8 (theta) (CCT8), mRNA [NM_006585] |
| THC2503633 | THC2503633 | tc|Q6SPE9_RABIT (Q6SPE9) Atherin, partial (6%) [THC2503633] |
| BC018676 | BC018676 | gb|Homo sapiens, clone IMAGE:4337652, mRNA. [BC018676] |
| CUL3 | NM_003590 | cullin 3 (CUL3), mRNA [NM_003590] |
| TAF5 | NM_006951 | TAF5 RNA polymerase II, TATA box binding protein (TBP)-associated factor, 100kDa (TAF5), mRNA [NM_006951] |
| TGS1 | NM_024831 | trimethylguanosine synthase 1 (TGS1), mRNA [NM_024831] |
| LOC400657 | NR_024484 | uncharacterized LOC400657 (LOC400657), non-coding RNA [NR_024484] |
| B3GNT8 | NM_198540 | UDP-GlcNAc:betaGal beta-1,3-N-acetylglucosaminyltransferase 8 (B3GNT8), mRNA [NM_198540] |
| SHOC2 | NM_007373 | soc-2 suppressor of clear homolog (C. elegans) (SHOC2), mRNA [NM_007373] |
| XLOC_006132 | DB461521 | gb|DB461521 RIKEN full-length enriched human cDNA library, testis Homo sapiens cDNA clone H013094H15 5', mRNA sequence [DB461521] |
| LOC100129917 | NR_036511 | uncharacterized LOC100129917 (LOC100129917), transcript variant 1, non-coding RNA [NR_036511] |
| SCARNA8 | NR_003009 | small Cajal body-specific RNA 8 (SCARNA8), guide RNA [NR_003009] |
| LOC100505857 | DA569430 | gb|DA569430 HEMBA1 Homo sapiens cDNA clone HEMBA1002792 5', mRNA sequence [DA569430] |
| CREB3L3 | NM_032607 | cAMP responsive element binding protein 3-like 3 (CREB3L3), mRNA [NM_032607] |
| XLOC_001339 | ENST00000478468 | tc|ALU1_HUMAN (P39188) Alu subfamily J sequence contamination warning entry, partial (9%) [THC2627935] |
| SNORD35A | NR_000018 | small nucleolar RNA, C/D box 35A (SNORD35A), small nucleolar RNA [NR_000018] |
| LOC100128264 | NR_038945 | uncharacterized LOC100128264 (LOC100128264), non-coding RNA [NR_038945] |
| PCNXL2 | ENST00000324142 | ens|pecanex-like 2 (Drosophila) [Source:HGNC Symbol;Acc:8736] [ENST00000324142] |
| FAHD1 | NM_031208 | fumarylacetoacetate hydrolase domain containing 1 (FAHD1), nuclear gene encoding mitochondrial protein, transcript variant 2, mRNA [NM_031208] |
| XLOC_l2_009736 | TCONS_l2_00018583 | linc|BROAD Institute lincRNA (XLOC_l2_009736), lincRNA [TCONS_l2_00018583] |
| METTL19 | NM_152544 | methyltransferase like 19 (METTL19), transcript variant 2, mRNA [NM_152544] |
| CEP152 | NM_014985 | centrosomal protein 152kDa (CEP152), transcript variant 2, mRNA [NM_014985] |
| VAC14 | U25801 | gb|Human Tax1 binding protein mRNA, partial cds. [U25801] |
| BC015443 | BC015443 | gb|Homo sapiens, clone IMAGE:4424208, mRNA. [BC015443] |
| XLOC_009507 | AK054718 | linc|BROAD Institute lincRNA (XLOC_009507), lincRNA [TCONS_00019712] |
| GTF2IRD2B | ENST00000529695 | ens|GTF2I repeat domain containing 2B [Source:HGNC Symbol;Acc:33125] [ENST00000529695] |
| TIMM17A | NM_006335 | translocase of inner mitochondrial membrane 17 homolog A (yeast) (TIMM17A), nuclear gene encoding mitochondrial protein, mRNA [NM_006335] |
| ZNF354A | NM_005649 | zinc finger protein 354A (ZNF354A), mRNA [NM_005649] |
| THC2512536 | THC2512536 | Unknown |
| SYT2 | NM_001136504 | synaptotagmin II (SYT2), transcript variant 2, mRNA [NM_001136504] |
| DDX10 | NM_004398 | DEAD (Asp-Glu-Ala-Asp) box polypeptide 10 (DDX10), mRNA [NM_004398] |
| ENST00000420509 | ENST00000420509 | ref|PREDICTED: Homo sapiens hypothetical LOC729570, transcript variant 2 (LOC729570), miscRNA [XR_112431] |
| RAB3GAP2 | NM_012414 | RAB3 GTPase activating protein subunit 2 (non-catalytic) (RAB3GAP2), mRNA [NM_012414] |
| ELAC1 | NM_018696 | elaC homolog 1 (E. coli) (ELAC1), mRNA [NM_018696] |
| OLFM1 | ENST00000371801 | ens|olfactomedin 1 [Source:HGNC Symbol;Acc:17187] [ENST00000371801] |
| DCUN1D3 | NM_173475 | DCN1, defective in cullin neddylation 1, domain containing 3 (S. cerevisiae) (DCUN1D3), mRNA [NM_173475] |
| AK123308 | AK123308 | gb|Homo sapiens cDNA FLJ41314 fis, clone BRAMY2042918. [AK123308] |
| CNKSR2 | NM_014927 | connector enhancer of kinase suppressor of Ras 2 (CNKSR2), transcript variant 1, mRNA [NM_014927] |
| EXO1 | NM_003686 | exonuclease 1 (EXO1), transcript variant 3, mRNA [NM_003686] |
| XLOC_009695 | AK092571 | tc|Q07826_HUMAN (Q07826) X-linked retinopathy protein protein (Fragment), partial (31%) [THC2628135] |
| SLC4A1AP | NM_018158 | solute carrier family 4 (anion exchanger), member 1, adaptor protein (SLC4A1AP), mRNA [NM_018158] |
| TFB2M | NM_022366 | transcription factor B2, mitochondrial (TFB2M), nuclear gene encoding mitochondrial protein, mRNA [NM_022366] |
| PSMD6 | NM_014814 | proteasome (prosome, macropain) 26S subunit, non-ATPase, 6 (PSMD6), mRNA [NM_014814] |
| ZNF773 | NM_198542 | zinc finger protein 773 (ZNF773), mRNA [NM_198542] |
| FAM27A | AK093358 | gb|Homo sapiens cDNA FLJ36039 fis, clone TESTI2017311. [AK093358] |
| TAF1D | NM_024116 | TATA box binding protein (TBP)-associated factor, RNA polymerase I, D, 41kDa (TAF1D), mRNA [NM_024116] |
| ARHGEF7 | NM_145735 | Rho guanine nucleotide exchange factor (GEF) 7 (ARHGEF7), transcript variant 2, mRNA [NM_145735] |
| VHLL | NM_001004319 | von Hippel-Lindau tumor suppressor-like (VHLL), mRNA [NM_001004319] |
| ZNF419 | NM_024691 | zinc finger protein 419 (ZNF419), transcript variant 2, mRNA [NM_024691] |
| PDCD7 | NM_005707 | programmed cell death 7 (PDCD7), mRNA [NM_005707] |
| ARID1B | NM_017519 | AT rich interactive domain 1B (SWI1-like) (ARID1B), transcript variant 1, mRNA [NM_017519] |
| SNORD42B | NR_000013 | small nucleolar RNA, C/D box 42B (SNORD42B), small nucleolar RNA [NR_000013] |
| MYNN | NM_018657 | myoneurin (MYNN), transcript variant 1, mRNA [NM_018657] |
| LOC100509638 | ENST00000426006 | ref|PREDICTED: Homo sapiens protein FAM27D1-like (LOC100509638), mRNA [XM_003120093] |
| PUM1 | NM_001020658 | pumilio homolog 1 (Drosophila) (PUM1), transcript variant 1, mRNA [NM_001020658] |
| PDZRN3 | NM_015009 | PDZ domain containing ring finger 3 (PDZRN3), mRNA [NM_015009] |
| XLOC_006179 | TCONS_00014176 | linc|BROAD Institute lincRNA (XLOC_006179), lincRNA [TCONS_00014176] |
| CEP350 | NM_014810 | centrosomal protein 350kDa (CEP350), mRNA [NM_014810] |
| PPIH | ENST00000372550 | ens|peptidylprolyl isomerase H (cyclophilin H) [Source:HGNC Symbol;Acc:14651] [ENST00000372550] |
| RFC4 | NM_002916 | replication factor C (activator 1) 4, 37kDa (RFC4), transcript variant 1, mRNA [NM_002916] |
| SRSF4 | NM_005626 | serine/arginine-rich splicing factor 4 (SRSF4), mRNA [NM_005626] |
| C2orf43 | ENST00000419825 | ens|chromosome 2 open reading frame 43 [Source:HGNC Symbol;Acc:26145] [ENST00000419825] |
| CNBP | NM_003418 | CCHC-type zinc finger, nucleic acid binding protein (CNBP), transcript variant 3, mRNA [NM_003418] |
| XLOC_l2_008203 | TCONS_l2_00014793 | linc|BROAD Institute lincRNA (XLOC_l2_008203), lincRNA [TCONS_l2_00014793] |
| THC2650095 | THC2650095 | tc|MYO5B_HUMAN (Q9ULV0) Myosin-5B (Myosin Vb), partial (5%) [THC2650095] |
| DDX27 | NM_017895 | DEAD (Asp-Glu-Ala-Asp) box polypeptide 27 (DDX27), mRNA [NM_017895] |
| THC2547170 | THC2547170 | tc|Q3KR39_HUMAN (Q3KR39) LOC125893 protein, partial (29%) [THC2547170] |
| ZNF441 | NM_152355 | zinc finger protein 441 (ZNF441), mRNA [NM_152355] |
| ZNF595 | NM_182524 | zinc finger protein 595 (ZNF595), mRNA [NM_182524] |
| XLOC_000667 | TCONS_00001367 | linc|BROAD Institute lincRNA (XLOC_000667), lincRNA [TCONS_00001367] |
| HIAT1 | NM_033055 | hippocampus abundant transcript 1 (HIAT1), mRNA [NM_033055] |
| SNORA40 | NR_002973 | small nucleolar RNA, H/ACA box 40 (SNORA40), small nucleolar RNA [NR_002973] |
| FOXN3 | NM_005197 | forkhead box N3 (FOXN3), transcript variant 2, mRNA [NM_005197] |
| GTF3C3 | NM_012086 | general transcription factor IIIC, polypeptide 3, 102kDa (GTF3C3), transcript variant 1, mRNA [NM_012086] |
| SNIP1 | NM_024700 | Smad nuclear interacting protein 1 (SNIP1), mRNA [NM_024700] |
| THSD1P1 | NR_002816 | thrombospondin, type I, domain containing 1 pseudogene 1 (THSD1P1), non-coding RNA [NR_002816] |
| ENAH | NM_001008493 | enabled homolog (Drosophila) (ENAH), transcript variant 1, mRNA [NM_001008493] |
| AY927497 | AY927497 | gb|Homo sapiens mRNA sequence. [AY927497] |
| XLOC_005748 | TCONS_00012191 | linc|BROAD Institute lincRNA (XLOC_005748), lincRNA [TCONS_00012191] |
| HIST1H1B | NM_005322 | histone cluster 1, H1b (HIST1H1B), mRNA [NM_005322] |
| XLOC_l2_003610 | THC2717143 | tc|HS1188J21 FSH primary response (LRPR1 homolog, rat) 1 {Homo sapiens} (exp=0; wgp=1; cg=0), partial (35%) [THC2717143] |
| METTL21D | NM_024558 | methyltransferase like 21D (METTL21D), transcript variant 1, mRNA [NM_024558] |
| ENTPD5 | NM_001249 | ectonucleoside triphosphate diphosphohydrolase 5 (ENTPD5), mRNA [NM_001249] |
| MAGI1 | NM_004742 | membrane associated guanylate kinase, WW and PDZ domain containing 1 (MAGI1), transcript variant 2, mRNA [NM_004742] |
| CEACAM22P | NR_027754 | carcinoembryonic antigen-related cell adhesion molecule 2, pseudogene (CEACAM22P), non-coding RNA [NR_027754] |
| LOC100507213 | ENST00000443016 | ref|PREDICTED: Homo sapiens hypothetical LOC100507213 (LOC100507213), miscRNA [XR_109008] |
| ZNF501 | NM_145044 | zinc finger protein 501 (ZNF501), mRNA [NM_145044] |
| METTL21A | NM_145280 | methyltransferase like 21A (METTL21A), transcript variant 1, mRNA [NM_145280] |
| LOC100129399 | AK124011 | gb|Homo sapiens cDNA FLJ42017 fis, clone SPLEN2033153. [AK124011] |
| A_24_P786713 | A_24_P786713 | Unknown |
| RBFOX2 | NM_001031695 | RNA binding protein, fox-1 homolog (C. elegans) 2 (RBFOX2), transcript variant 1, mRNA [NM_001031695] |
| SNX5 | NM_014426 | sorting nexin 5 (SNX5), transcript variant 2, mRNA [NM_014426] |
| LOC100505648 | NR_040058 | uncharacterized LOC100505648 (LOC100505648), non-coding RNA [NR_040058] |
| RASSF8 | NM_007211 | Ras association (RalGDS/AF-6) domain family (N-terminal) member 8 (RASSF8), transcript variant 4, mRNA [NM_007211] |
| LRPPRC | NM_133259 | leucine-rich PPR-motif containing (LRPPRC), mRNA [NM_133259] |
| CHD2 | NM_001271 | chromodomain helicase DNA binding protein 2 (CHD2), transcript variant 1, mRNA [NM_001271] |
| HIST1H2AL | NM_003511 | histone cluster 1, H2al (HIST1H2AL), mRNA [NM_003511] |
| HIST1H3C | NM_003531 | histone cluster 1, H3c (HIST1H3C), mRNA [NM_003531] |
| RGS2 | NM_002923 | regulator of G-protein signaling 2, 24kDa (RGS2), mRNA [NM_002923] |
| HIST1H3J | NM_003535 | histone cluster 1, H3j (HIST1H3J), mRNA [NM_003535] |
| ZFP36L1 | ENST00000408913 | ens|zinc finger protein 36, C3H type-like 1 [Source:HGNC Symbol;Acc:1107] [ENST00000408913] |
| C1orf135 | NM_024037 | chromosome 1 open reading frame 135 (C1orf135), mRNA [NM_024037] |
| ENC1 | NM_003633 | ectodermal-neural cortex 1 (with BTB-like domain) (ENC1), mRNA [NM_003633] |
| CNTD1 | NM_173478 | cyclin N-terminal domain containing 1 (CNTD1), mRNA [NM_173478] |
| DOCK9 | NM_015296 | dedicator of cytokinesis 9 (DOCK9), transcript variant 1, mRNA [NM_015296] |
| TOP2A | NM_001067 | topoisomerase (DNA) II alpha 170kDa (TOP2A), mRNA [NM_001067] |
| FLJ40852 | NR_015392 | uncharacterized LOC285962 (FLJ40852), non-coding RNA [NR_015392] |
| ZNF211 | NM_198855 | zinc finger protein 211 (ZNF211), transcript variant 2, mRNA [NM_198855] |
| THC2634132 | THC2634132 | tc|Q25IK4_MACFA (Q25IK4) Brain cDNA, clone: QflA-18945, partial (98%) [THC2634132] |
| BC029255 | BC029255 | gb|Homo sapiens, clone IMAGE:5170250, mRNA. [BC029255] |
| XLOC_002037 | ENST00000446073 | linc|BROAD Institute lincRNA (XLOC_002037), lincRNA [TCONS_00003245] |
| XLOC_001266 | TCONS_00001887 | linc|BROAD Institute lincRNA (XLOC_001266), lincRNA [TCONS_00001887] |
| ZFP62 | NM_152283 | zinc finger protein 62 homolog (mouse) (ZFP62), transcript variant 1, mRNA [NM_152283] |
| LOC388282 | XR_111582 | ref|PREDICTED: Homo sapiens hypothetical LOC388282 (LOC388282), miscRNA [XR_111582] |
| ENST00000425104 | ENST00000425104 | Unknown |
| C19orf40 | NM_152266 | chromosome 19 open reading frame 40 (C19orf40), mRNA [NM_152266] |
| PRR3 | ENST00000461523 | ens|proline rich 3 [Source:HGNC Symbol;Acc:21149] [ENST00000461523] |
| XLOC_l2_000101 | THC2710823 | tc|Q9AMR3_AZOVI (Q9AMR3) Transposase, partial (10%) [THC2710823] |
| RPRD2 | ENST00000369067 | ens|regulation of nuclear pre-mRNA domain containing 2 [Source:HGNC Symbol;Acc:29039] [ENST00000369067] |
| BU659163 | BU659163 | gb|cl44e01.z1 Hembase; Erythroid Precursor Cells (LCB:cl library) Homo sapiens cDNA clone cl44e01 5', mRNA sequence [BU659163] |
| CNOT10 | NM_015442 | CCR4-NOT transcription complex, subunit 10 (CNOT10), mRNA [NM_015442] |
| ENST00000439955 | ENST00000439955 | Unknown |
| FOXI3 | NM_001135649 | forkhead box I3 (FOXI3), mRNA [NM_001135649] |
| XLOC_014211 | ENST00000430449 | linc|BROAD Institute lincRNA (XLOC_014211), lincRNA [TCONS_00029412] |
| NDUFS1 | NM_005006 | NADH dehydrogenase (ubiquinone) Fe-S protein 1, 75kDa (NADH-coenzyme Q reductase) (NDUFS1), nuclear gene encoding mitochondrial protein, transcript variant 1, mRNA [NM_005006] |
| ZNF729 | NM_001242680 | zinc finger protein 729 (ZNF729), mRNA [NM_001242680] |
| DPP8 | NM_130434 | dipeptidyl-peptidase 8 (DPP8), transcript variant 1, mRNA [NM_130434] |
| A_24_P246963 | A_24_P246963 | Unknown |
| LRRC4C | NM_020929 | leucine rich repeat containing 4C (LRRC4C), mRNA [NM_020929] |
| GZF1 | NM_022482 | GDNF-inducible zinc finger protein 1 (GZF1), mRNA [NM_022482] |
| LOC100129104 | XR_108889 | ref|PREDICTED: Homo sapiens hypothetical LOC100129104 (LOC100129104), miscRNA [XR_108889] |
| ENST00000453496 | ENST00000453496 | ref|PREDICTED: Homo sapiens hypothetical protein FLJ37396 (FLJ37396), mRNA [XM_003403516] |
| MEMO1 | ENST00000407893 | ens|mediator of cell motility 1 [Source:HGNC Symbol;Acc:14014] [ENST00000407893] |
| HNRNPAB | NM_004499 | heterogeneous nuclear ribonucleoprotein A/B (HNRNPAB), transcript variant 2, mRNA [NM_004499] |
| USP51 | NM_201286 | ubiquitin specific peptidase 51 (USP51), mRNA [NM_201286] |
| ZW10 | NM_004724 | ZW10, kinetochore associated, homolog (Drosophila) (ZW10), mRNA [NM_004724] |
| AAK1 | NM_014911 | AP2 associated kinase 1 (AAK1), mRNA [NM_014911] |
| SNORD45B | NR_002748 | small nucleolar RNA, C/D box 45B (SNORD45B), small nuclear RNA [NR_002748] |
| ZNF485 | NM_145312 | zinc finger protein 485 (ZNF485), mRNA [NM_145312] |
| KCTD20 | NM_173562 | potassium channel tetramerisation domain containing 20 (KCTD20), mRNA [NM_173562] |
| OCLM | NM_022375 | oculomedin (OCLM), mRNA [NM_022375] |
| LOC728802 | XM_001713923 | ref|PREDICTED: Homo sapiens myomegalin-like (LOC728802), mRNA [XM_001713923] |
| PRO1768 | NR_024620 | PRO1768 (PRO1768), non-coding RNA [NR_024620] |
| XLOC_005788 | TCONS_00012231 | linc|BROAD Institute lincRNA (XLOC_005788), lincRNA [TCONS_00012231] |
| DLD | NM_000108 | dihydrolipoamide dehydrogenase (DLD), mRNA [NM_000108] |
| PIGB | NM_004855 | phosphatidylinositol glycan anchor biosynthesis, class B (PIGB), mRNA [NM_004855] |
| XLOC_l2_009883 | TCONS_l2_00018854 | linc|BROAD Institute lincRNA (XLOC_l2_009883), lincRNA [TCONS_l2_00018854] |
| XLOC_008922 | TCONS_00018588 | linc|BROAD Institute lincRNA (XLOC_008922), lincRNA [TCONS_00018588] |
| TPP2 | ENST00000490010 | ens|tripeptidyl peptidase II [Source:HGNC Symbol;Acc:12016] [ENST00000490010] |
| DMXL2 | NM_015263 | Dmx-like 2 (DMXL2), transcript variant 2, mRNA [NM_015263] |
| COG5 | NM_006348 | component of oligomeric golgi complex 5 (COG5), transcript variant 1, mRNA [NM_006348] |
| KLF6 | NM_001300 | Kruppel-like factor 6 (KLF6), transcript variant A, mRNA [NM_001300] |
| PAPD5 | NM_001040284 | PAP associated domain containing 5 (PAPD5), transcript variant 1, mRNA [NM_001040284] |
| KLHL12 | NM_021633 | kelch-like 12 (Drosophila) (KLHL12), mRNA [NM_021633] |
| ZSCAN21 | NM_145914 | zinc finger and SCAN domain containing 21 (ZSCAN21), mRNA [NM_145914] |
| CDCA2 | NM_152562 | cell division cycle associated 2 (CDCA2), mRNA [NM_152562] |
| IMMT | NM_006839 | inner membrane protein, mitochondrial (IMMT), nuclear gene encoding mitochondrial protein, transcript variant 1, mRNA [NM_006839] |
| CRTAP | NM_006371 | cartilage associated protein (CRTAP), mRNA [NM_006371] |
| XLOC_l2_003992 | TCONS_l2_00007207 | linc|BROAD Institute lincRNA (XLOC_l2_003992), lincRNA [TCONS_l2_00007207] |
| KIF23 | NM_138555 | kinesin family member 23 (KIF23), transcript variant 1, mRNA [NM_138555] |
| OR4A5 | NM_001005272 | olfactory receptor, family 4, subfamily A, member 5 (OR4A5), mRNA [NM_001005272] |
| XLOC_011694 | TCONS_00024370 | linc|BROAD Institute lincRNA (XLOC_011694), lincRNA [TCONS_00024370] |
| XLOC_014288 | TCONS_00029633 | linc|BROAD Institute lincRNA (XLOC_014288), lincRNA [TCONS_00029633] |
| XLOC_l2_000416 | THC2561048 | tc|Q16003_HUMAN (Q16003) Lymphocyte-specific protein 1 (Fragment), complete [THC2561048] |
| XLOC_007904 | TCONS_00016520 | linc|BROAD Institute lincRNA (XLOC_007904), lincRNA [TCONS_00016520] |
| PHF7 | NM_173341 | PHD finger protein 7 (PHF7), transcript variant 2, mRNA [NM_173341] |
| MIAT | NR_033319 | myocardial infarction associated transcript (non-protein coding) (MIAT), transcript variant 2, non-coding RNA [NR_033319] |
| ENST00000390284 | ENST00000390284 | ens|immunoglobulin lambda variable 4-60 [Source:HGNC Symbol;Acc:5920] [ENST00000390284] |
| ERC1 | NM_178040 | ELKS/RAB6-interacting/CAST family member 1 (ERC1), transcript variant epsilon, mRNA [NM_178040] |
| TAF1B | NM_005680 | TATA box binding protein (TBP)-associated factor, RNA polymerase I, B, 63kDa (TAF1B), mRNA [NM_005680] |
| WDR75 | NM_032168 | WD repeat domain 75 (WDR75), mRNA [NM_032168] |
| TUBGCP5 | NM_052903 | tubulin, gamma complex associated protein 5 (TUBGCP5), transcript variant 1, mRNA [NM_052903] |
| GOLGA2 | ENST00000462089 | ens|golgin A2 [Source:HGNC Symbol;Acc:4425] [ENST00000462089] |
| XLOC_014139 | ENST00000442403 | tc|AK220401 mKIAA1543 protein {Mus musculus} (exp=-1; wgp=0; cg=0), partial (3%) [THC2764081] |
| SPTY2D1 | NM_194285 | SPT2, Suppressor of Ty, domain containing 1 (S. cerevisiae) (SPTY2D1), mRNA [NM_194285] |
| XLOC_008897 | ENST00000449882 | linc|BROAD Institute lincRNA (XLOC_008897), lincRNA [TCONS_00018020] |
| SUV39H2 | NM_024670 | suppressor of variegation 3-9 homolog 2 (Drosophila) (SUV39H2), transcript variant 3, mRNA [NM_024670] |
| PTER | NM_001001484 | phosphotriesterase related (PTER), transcript variant 1, mRNA [NM_001001484] |
| SOX11 | NM_003108 | SRY (sex determining region Y)-box 11 (SOX11), mRNA [NM_003108] |
| ZYG11B | NM_024646 | zyg-11 homolog B (C. elegans) (ZYG11B), mRNA [NM_024646] |
| ZMYND8 | ENST00000468376 | ens|zinc finger, MYND-type containing 8 [Source:HGNC Symbol;Acc:9397] [ENST00000468376] |
| WWP1 | NM_007013 | WW domain containing E3 ubiquitin protein ligase 1 (WWP1), mRNA [NM_007013] |
| LCN1 | NM_002297 | lipocalin 1 (tear prealbumin) (LCN1), mRNA [NM_002297] |
| LOC653061 | NR_038843 | golgin A8 family, member B pseudogene (LOC653061), non-coding RNA [NR_038843] |
| IRAK4 | NM_016123 | interleukin-1 receptor-associated kinase 4 (IRAK4), transcript variant 2, mRNA [NM_016123] |
| AK001057 | AK001057 | gb|Homo sapiens cDNA FLJ10195 fis, clone HEMBA1004771. [AK001057] |
| ENST00000443467 | ENST00000443467 | Unknown |
| MAGEE1 | NM_020932 | melanoma antigen family E, 1 (MAGEE1), mRNA [NM_020932] |
| LOC100130950 | NR_034082 | uncharacterized LOC100130950 (LOC100130950), non-coding RNA [NR_034082] |
| E2F7 | NM_203394 | E2F transcription factor 7 (E2F7), mRNA [NM_203394] |
| MAX | NM_145114 | MYC associated factor X (MAX), transcript variant 4, mRNA [NM_145114] |
| CENPQ | NM_018132 | centromere protein Q (CENPQ), mRNA [NM_018132] |
| MRPL19 | ENST00000409374 | ens|mitochondrial ribosomal protein L19 [Source:HGNC Symbol;Acc:14052] [ENST00000409374] |
| NR1D2 | NM_005126 | nuclear receptor subfamily 1, group D, member 2 (NR1D2), transcript variant 1, mRNA [NM_005126] |
| SCAF4 | NM_020706 | SR-related CTD-associated factor 4 (SCAF4), transcript variant 1, mRNA [NM_020706] |
| SNORD21 | NR_000006 | small nucleolar RNA, C/D box 21 (SNORD21), small nucleolar RNA [NR_000006] |
| GIGYF2 | NM_015575 | GRB10 interacting GYF protein 2 (GIGYF2), transcript variant 2, mRNA [NM_015575] |
| XLOC_004678 | ENST00000504573 | linc|BROAD Institute lincRNA (XLOC_004678), lincRNA [TCONS_00009562] |
| ZBTB8B | NM_001145720 | zinc finger and BTB domain containing 8B (ZBTB8B), mRNA [NM_001145720] |
| CHRNB1 | NM_000747 | cholinergic receptor, nicotinic, beta 1 (muscle) (CHRNB1), mRNA [NM_000747] |
| SCARNA6 | NR_003006 | small Cajal body-specific RNA 6 (SCARNA6), guide RNA [NR_003006] |
| XLOC_l2_000217 | ENST00000436033 | Unknown |
| EXOSC6 | NM_058219 | exosome component 6 (EXOSC6), mRNA [NM_058219] |
| LOC388210 | XM_001716361 | ref|PREDICTED: Homo sapiens apolipophorins-like (LOC388210), mRNA [XM_001716361] |
| PPP2R2A | NM_002717 | protein phosphatase 2, regulatory subunit B, alpha (PPP2R2A), transcript variant 1, mRNA [NM_002717] |
| XLOC_013842 | TCONS_00028469 | linc|BROAD Institute lincRNA (XLOC_013842), lincRNA [TCONS_00028469] |
| B4GALT4 | NM_212543 | UDP-Gal:betaGlcNAc beta 1,4- galactosyltransferase, polypeptide 4 (B4GALT4), transcript variant 1, mRNA [NM_212543] |
| MED23 | ENST00000368053 | ens|mediator complex subunit 23 [Source:HGNC Symbol;Acc:2372] [ENST00000368053] |
| A_33_P3354067 | A_33_P3354067 | Unknown |
| RPL15 | NM_002948 | ribosomal protein L15 (RPL15), mRNA [NM_002948] |
| MTAP | NM_002451 | methylthioadenosine phosphorylase (MTAP), mRNA [NM_002451] |
| MDN1 | NM_014611 | MDN1, midasin homolog (yeast) (MDN1), mRNA [NM_014611] |
| ATP2B4 | NM_001684 | ATPase, Ca++ transporting, plasma membrane 4 (ATP2B4), transcript variant 2, mRNA [NM_001684] |
| TMEM50A | NM_014313 | transmembrane protein 50A (TMEM50A), mRNA [NM_014313] |
| XLOC_002978 | ENST00000440556 | linc|BROAD Institute lincRNA (XLOC_002978), lincRNA [TCONS_00005699] |
| BC066878 | BC066878 | gb|Homo sapiens cDNA clone IMAGE:6971743, partial cds. [BC066878] |
| CTSL3 | NR_027917 | cathepsin L family member 3 (CTSL3), non-coding RNA [NR_027917] |
| CDKN1B | NM_004064 | cyclin-dependent kinase inhibitor 1B (p27, Kip1) (CDKN1B), mRNA [NM_004064] |
| ARHGAP10 | NM_024605 | Rho GTPase activating protein 10 (ARHGAP10), mRNA [NM_024605] |
| MCMBP | NM_024834 | minichromosome maintenance complex binding protein (MCMBP), mRNA [NM_024834] |
| XLOC_006151 | TCONS_00013502 | linc|BROAD Institute lincRNA (XLOC_006151), lincRNA [TCONS_00013502] |
| TCERG1 | NM_006706 | transcription elongation regulator 1 (TCERG1), transcript variant 1, mRNA [NM_006706] |
| P39195 | THC2648133 | tc|ALU8_HUMAN (P39195) Alu subfamily SX sequence contamination warning entry, partial (20%) [THC2648133] |
| NKIRAS1 | NM_020345 | NFKB inhibitor interacting Ras-like 1 (NKIRAS1), mRNA [NM_020345] |
| LOC100653259 | XR_133099 | ref|PREDICTED: Homo sapiens e3 ubiquitin-protein ligase HERC2-like (LOC100653259), miscRNA [XR_133099] |
| ENST00000375955 | ENST00000375955 | ens|excision repair cross-complementing rodent repair deficiency, complementation group 5 [Source:HGNC Symbol;Acc:3437] [ENST00000375955] |
| GIN1 | NM_017676 | gypsy retrotransposon integrase 1 (GIN1), mRNA [NM_017676] |
| CHAF1A | NM_005483 | chromatin assembly factor 1, subunit A (p150) (CHAF1A), mRNA [NM_005483] |
| GRHL1 | NM_198182 | grainyhead-like 1 (Drosophila) (GRHL1), mRNA [NM_198182] |
| FAM104B | NM_138362 | family with sequence similarity 104, member B (FAM104B), transcript variant 1, mRNA [NM_138362] |
| RAB14 | NM_016322 | RAB14, member RAS oncogene family (RAB14), mRNA [NM_016322] |
| EIF2C2 | NM_012154 | eukaryotic translation initiation factor 2C, 2 (EIF2C2), transcript variant 1, mRNA [NM_012154] |
| RAB9A | NM_004251 | RAB9A, member RAS oncogene family (RAB9A), transcript variant 1, mRNA [NM_004251] |
| ZFAT | ENST00000522974 | ens|zinc finger and AT hook domain containing [Source:HGNC Symbol;Acc:19899] [ENST00000522974] |
| SHPRH | NM_173082 | SNF2 histone linker PHD RING helicase (SHPRH), transcript variant 2, mRNA [NM_173082] |
| LOC145678 | AL109682 | gb|Homo sapiens mRNA full length insert cDNA clone EUROIMAGE 35394. [AL109682] |
| ANKRD27 | NM_032139 | ankyrin repeat domain 27 (VPS9 domain) (ANKRD27), mRNA [NM_032139] |
| OTX2OS1 | NR_029385 | Otx2 opposite strand transcript 1 (OTX2OS1), non-coding RNA [NR_029385] |
| XLOC_005520 | TCONS_00012005 | linc|BROAD Institute lincRNA (XLOC_005520), lincRNA [TCONS_00012005] |
| PSMD7 | NM_002811 | proteasome (prosome, macropain) 26S subunit, non-ATPase, 7 (PSMD7), mRNA [NM_002811] |
| SEC24A | NM_001252231 | SEC24 family, member A (S. cerevisiae) (SEC24A), transcript variant 2, mRNA [NM_001252231] |
| LOC100132352 | AK023536 | gb|NM19con Human primary neuroblastoma stage 4S Homo sapiens cDNA clone 10F 3', mRNA sequence [EY892389] |
| UTY | NM_182660 | ubiquitously transcribed tetratricopeptide repeat gene, Y-linked (UTY), transcript variant 1, mRNA [NM_182660] |
| CCT6A | NM_001762 | chaperonin containing TCP1, subunit 6A (zeta 1) (CCT6A), transcript variant 1, mRNA [NM_001762] |
| TSC1 | ENST00000403810 | ens|tuberous sclerosis 1 [Source:HGNC Symbol;Acc:12362] [ENST00000403810] |
| C1orf124 | NM_001010984 | chromosome 1 open reading frame 124 (C1orf124), transcript variant 2, mRNA [NM_001010984] |
| XLOC_010754 | TCONS_00022389 | linc|BROAD Institute lincRNA (XLOC_010754), lincRNA [TCONS_00022389] |
| TMEM188 | NM_153261 | transmembrane protein 188 (TMEM188), mRNA [NM_153261] |
| XLOC_l2_015561 | ENST00000453317 | linc|BROAD Institute lincRNA (XLOC_l2_015561), lincRNA [TCONS_l2_00030254] |
| GPR89B | NM_016334 | G protein-coupled receptor 89B (GPR89B), mRNA [NM_016334] |
| KATNA1 | NM_007044 | katanin p60 (ATPase containing) subunit A 1 (KATNA1), transcript variant 1, mRNA [NM_007044] |
| XLOC_005121 | TCONS_00011632 | linc|BROAD Institute lincRNA (XLOC_005121), lincRNA [TCONS_00011632] |
| DNAJB9 | NM_012328 | DnaJ (Hsp40) homolog, subfamily B, member 9 (DNAJB9), mRNA [NM_012328] |
| AFF3 | NM_002285 | AF4/FMR2 family, member 3 (AFF3), transcript variant 1, mRNA [NM_002285] |
| ENST00000507038 | ENST00000507038 | Unknown |
| ITSN1 | NM_001001132 | intersectin 1 (SH3 domain protein) (ITSN1), transcript variant 2, mRNA [NM_001001132] |
| CDK13 | NM_003718 | cyclin-dependent kinase 13 (CDK13), transcript variant 1, mRNA [NM_003718] |
| LOC727710 | NR_038858 | uncharacterized locus LOC727710 (LOC727710), non-coding RNA [NR_038858] |
| SNORD1C | NR_004397 | small nucleolar RNA, C/D box 1C (SNORD1C), small nucleolar RNA [NR_004397] |
| SUN1 | ENST00000340926 | ens|Sad1 and UNC84 domain containing 1 [Source:HGNC Symbol;Acc:18587] [ENST00000340926] |
| XLOC_006419 | TCONS_00013784 | linc|BROAD Institute lincRNA (XLOC_006419), lincRNA [TCONS_00013784] |
| FOXP1 | NM_032682 | forkhead box P1 (FOXP1), transcript variant 1, mRNA [NM_032682] |
| FLJ22447 | NR_039985 | uncharacterized LOC400221 (FLJ22447), non-coding RNA [NR_039985] |
| RUFY4 | NM_198483 | RUN and FYVE domain containing 4 (RUFY4), transcript variant 1, mRNA [NM_198483] |
| RPS6KA5 | NM_004755 | ribosomal protein S6 kinase, 90kDa, polypeptide 5 (RPS6KA5), transcript variant 1, mRNA [NM_004755] |
| GGCT | NM_024051 | gamma-glutamylcyclotransferase (GGCT), transcript variant 1, mRNA [NM_024051] |
| CHMP3 | NM_016079 | charged multivesicular body protein 3 (CHMP3), transcript variant 1, mRNA [NM_016079] |
| BCAS2 | NM_005872 | breast carcinoma amplified sequence 2 (BCAS2), mRNA [NM_005872] |
| XLOC_l2_014830 | THC2583825 | tc|FRG1_HUMAN (Q14331) FRG1 protein (FSHD region gene 1 protein), partial (23%) [THC2583825] |
| TMEM69 | NM_016486 | transmembrane protein 69 (TMEM69), mRNA [NM_016486] |
| XLOC_009937 | BX101435 | gb|BX101435 Soares_testis_NHT Homo sapiens cDNA clone IMAGp998N063521, mRNA sequence [BX101435] |
| MGC12488 | BC005372 | gb|Homo sapiens, clone IMAGE:3932794, mRNA. [BC005372] |
| LOC100506310 | ENST00000433342 | ens|chromosome 1 open reading frame 167 [Source:HGNC Symbol;Acc:25262] [ENST00000433342] |
| PRMT3 | NM_005788 | protein arginine methyltransferase 3 (PRMT3), transcript variant 1, mRNA [NM_005788] |
| FEZ2 | NM_001042548 | fasciculation and elongation protein zeta 2 (zygin II) (FEZ2), transcript variant 2, mRNA [NM_001042548] |
| LINC00229 | NR_044991 | long intergenic non-protein coding RNA 229 (LINC00229), non-coding RNA [NR_044991] |
| THPO | NM_000460 | thrombopoietin (THPO), transcript variant 1, mRNA [NM_000460] |
| RPL32 | NM_001007074 | ribosomal protein L32 (RPL32), transcript variant 3, mRNA [NM_001007074] |
| SRSF10 | NM_054016 | serine/arginine-rich splicing factor 10 (SRSF10), transcript variant 2, mRNA [NM_054016] |
| ZNF625 | NM_145233 | zinc finger protein 625 (ZNF625), transcript variant 1, mRNA [NM_145233] |
| XLOC_004924 | THC2719986 | tc|Q26195_PLAVI (Q26195) Pva1 protein, partial (11%) [THC2719986] |
| CDC23 | ENST00000394884 | ens|cell division cycle 23 homolog (S. cerevisiae) [Source:HGNC Symbol;Acc:1724] [ENST00000394884] |
| SNX25 | NM_031953 | sorting nexin 25 (SNX25), mRNA [NM_031953] |
| LOC100132069 | AK123509 | gb|Homo sapiens cDNA FLJ41515 fis, clone BRTHA2011641. [AK123509] |
| CDC14B | NM_003671 | CDC14 cell division cycle 14 homolog B (S. cerevisiae) (CDC14B), transcript variant 1, mRNA [NM_003671] |
| GSPT2 | NM_018094 | G1 to S phase transition 2 (GSPT2), mRNA [NM_018094] |
| LOC100507375 | XR_108500 | ref|PREDICTED: Homo sapiens hypothetical LOC100507375 (LOC100507375), miscRNA [XR_108500] |
| GATA2 | NM_001145661 | GATA binding protein 2 (GATA2), transcript variant 1, mRNA [NM_001145661] |
| XLOC_007500 | BC039487 | linc|BROAD Institute lincRNA (XLOC_007500), lincRNA [TCONS_00015582] |
| SFR1 | NM_145247 | SWI5-dependent recombination repair 1 (SFR1), transcript variant 2, mRNA [NM_145247] |
| SNORA2A | NR_002950 | small nucleolar RNA, H/ACA box 2A (SNORA2A), small nucleolar RNA [NR_002950] |
| SACS | NM_014363 | spastic ataxia of Charlevoix-Saguenay (sacsin) (SACS), mRNA [NM_014363] |
| ZNF101 | NM_033204 | zinc finger protein 101 (ZNF101), mRNA [NM_033204] |
| WHAMM | NM_001080435 | WAS protein homolog associated with actin, golgi membranes and microtubules (WHAMM), mRNA [NM_001080435] |
| SNAPC3 | ENST00000380799 | ens|small nuclear RNA activating complex, polypeptide 3, 50kDa [Source:HGNC Symbol;Acc:11136] [ENST00000380799] |
| LOC401164 | AK095968 | gb|Homo sapiens cDNA FLJ38649 fis, clone HHDPC2007302. [AK095968] |
| PCDHB2 | NM_018936 | protocadherin beta 2 (PCDHB2), mRNA [NM_018936] |
| CNOT4 | NM_001008225 | CCR4-NOT transcription complex, subunit 4 (CNOT4), transcript variant 2, mRNA [NM_001008225] |
| ZNF561 | NM_152289 | zinc finger protein 561 (ZNF561), mRNA [NM_152289] |
| XLOC_l2_007880 | ENST00000416534 | linc|BROAD Institute lincRNA (XLOC_l2_007880), lincRNA [TCONS_l2_00015694] |
| C3orf35 | NM_178339 | chromosome 3 open reading frame 35 (C3orf35), transcript variant B, mRNA [NM_178339] |
| PACSIN2 | NM_007229 | protein kinase C and casein kinase substrate in neurons 2 (PACSIN2), transcript variant 2, mRNA [NM_007229] |
| DNM1P46 | BC101081 | linc|BROAD Institute lincRNA (XLOC_l2_005074), lincRNA [TCONS_l2_00009545] |
| ZFYVE9 | NM_004799 | zinc finger, FYVE domain containing 9 (ZFYVE9), transcript variant 3, mRNA [NM_004799] |
| STRC | NM_153700 | stereocilin (STRC), mRNA [NM_153700] |
| XLOC_009863 | TCONS_00020550 | linc|BROAD Institute lincRNA (XLOC_009863), lincRNA [TCONS_00020550] |
| XLOC_003678 | TCONS_00008216 | linc|BROAD Institute lincRNA (XLOC_003678), lincRNA [TCONS_00008216] |
| IL18 | NM_001562 | interleukin 18 (interferon-gamma-inducing factor) (IL18), transcript variant 1, mRNA [NM_001562] |
| PAPLN | NM_173462 | papilin, proteoglycan-like sulfated glycoprotein (PAPLN), mRNA [NM_173462] |
| PRO1596 | AF119915 | gb|Homo sapiens PRO3090 mRNA, complete cds. [AF119915] |
| XLOC_012222 | BC031827 | tc|ALU2_HUMAN (P39189) Alu subfamily SB sequence contamination warning entry, partial (7%) [THC2486332] |
| XLOC_010602 | TCONS_00022278 | linc|BROAD Institute lincRNA (XLOC_010602), lincRNA [TCONS_00022278] |
| C10orf28 | NM_014472 | chromosome 10 open reading frame 28 (C10orf28), mRNA [NM_014472] |
| MXD1 | NM_002357 | MAX dimerization protein 1 (MXD1), transcript variant 1, mRNA [NM_002357] |
| A_23_P90470 | A_23_P90470 | Unknown |
| HVCN1 | NM_001040107 | hydrogen voltage-gated channel 1 (HVCN1), transcript variant 1, mRNA [NM_001040107] |
| MRPS22 | NM_020191 | mitochondrial ribosomal protein S22 (MRPS22), nuclear gene encoding mitochondrial protein, mRNA [NM_020191] |
| C9orf5 | NM_032012 | chromosome 9 open reading frame 5 (C9orf5), mRNA [NM_032012] |
| ABT1 | NM_013375 | activator of basal transcription 1 (ABT1), mRNA [NM_013375] |
| ZNF429 | NM_001001415 | zinc finger protein 429 (ZNF429), mRNA [NM_001001415] |
| LOC100130581 | AK127427 | gb|Homo sapiens cDNA FLJ45519 fis, clone BRTHA2024177. [AK127427] |
| XLOC_002997 | THC2591825 | linc|BROAD Institute lincRNA (XLOC_002997), lincRNA [TCONS_00006381] |
| IQCG | NM_032263 | IQ motif containing G (IQCG), transcript variant 1, mRNA [NM_032263] |
| LOC100131372 | XR_110620 | ref|PREDICTED: Homo sapiens hypothetical LOC100131372 (LOC100131372), miscRNA [XR_110620] |
| CCRN4L | NM_012118 | CCR4 carbon catabolite repression 4-like (S. cerevisiae) (CCRN4L), mRNA [NM_012118] |
| TMPRSS15 | NM_002772 | transmembrane protease, serine 15 (TMPRSS15), mRNA [NM_002772] |
| LOC100507165 | XR_110530 | ref|PREDICTED: Homo sapiens hypothetical LOC100507165 (LOC100507165), miscRNA [XR_110530] |
| XLOC_011059 | THC2686750 | linc|BROAD Institute lincRNA (XLOC_011059), lincRNA [TCONS_00022775] |
| XLOC_l2_008760 | ENST00000444792 | linc|BROAD Institute lincRNA (XLOC_l2_008760), lincRNA [TCONS_l2_00016849] |
| XLOC_003202 | TCONS_00007222 | linc|BROAD Institute lincRNA (XLOC_003202), lincRNA [TCONS_00007222] |
| NDNF | NM_024574 | neuron-derived neurotrophic factor (NDNF), mRNA [NM_024574] |
| XLOC_000153 | TCONS_00001990 | linc|BROAD Institute lincRNA (XLOC_000153), lincRNA [TCONS_00001990] |
| COX11 | NM_004375 | COX11 cytochrome c oxidase assembly homolog (yeast) (COX11), nuclear gene encoding mitochondrial protein, transcript variant 1, mRNA [NM_004375] |
| XLOC_010625 | THC2660639 | linc|BROAD Institute lincRNA (XLOC_010625), lincRNA [TCONS_00022029] |
| WDR82 | NM_025222 | WD repeat domain 82 (WDR82), mRNA [NM_025222] |
| ZNF642 | NM_198494 | zinc finger protein 642 (ZNF642), mRNA [NM_198494] |
| LTN1 | BC031633 | gb|Homo sapiens cDNA clone IMAGE:5172245, containing frame-shift errors. [BC031633] |
| CTR9 | NM_014633 | Ctr9, Paf1/RNA polymerase II complex component, homolog (S. cerevisiae) (CTR9), mRNA [NM_014633] |
| DIS3L | NM_133375 | DIS3 mitotic control homolog (S. cerevisiae)-like (DIS3L), transcript variant 2, mRNA [NM_133375] |
| XLOC_012771 | TCONS_00026425 | linc|BROAD Institute lincRNA (XLOC_012771), lincRNA [TCONS_00026425] |
| LOC100130741 | AK127222 | gb|Homo sapiens cDNA FLJ45289 fis, clone BRHIP3002363. [AK127222] |
| PTEN | NM_000314 | phosphatase and tensin homolog (PTEN), mRNA [NM_000314] |
| LMTK2 | NM_014916 | lemur tyrosine kinase 2 (LMTK2), mRNA [NM_014916] |
| PCSK5 | NM_006200 | proprotein convertase subtilisin/kexin type 5 (PCSK5), transcript variant 2, mRNA [NM_006200] |
| LOC100128640 | NR_028389 | uncharacterized LOC100128640 (LOC100128640), non-coding RNA [NR_028389] |
| DYNLT1 | ENST00000367088 | ens|dynein, light chain, Tctex-type 1 [Source:HGNC Symbol;Acc:11697] [ENST00000367088] |
| ZNF253 | NM_021047 | zinc finger protein 253 (ZNF253), mRNA [NM_021047] |
| POLL | NM_001174084 | polymerase (DNA directed), lambda (POLL), transcript variant 1, mRNA [NM_001174084] |
| USP12 | NM_182488 | ubiquitin specific peptidase 12 (USP12), mRNA [NM_182488] |
| CU677925 | CU677925 | gb|Synthetic construct Homo sapiens gateway clone IMAGE:100018904 3' read HNRPA1 mRNA. [CU677925] |
| TMEM185B | NM_024121 | transmembrane protein 185B (TMEM185B), mRNA [NM_024121] |
| XLOC_l2_013436 | TCONS_l2_00025925 | linc|BROAD Institute lincRNA (XLOC_l2_013436), lincRNA [TCONS_l2_00025925] |
| ZCCHC7 | ENST00000322831 | ens|zinc finger, CCHC domain containing 7 [Source:HGNC Symbol;Acc:26209] [ENST00000322831] |
| TP53RK | NM_033550 | TP53 regulating kinase (TP53RK), mRNA [NM_033550] |
| ENTPD7 | NM_020354 | ectonucleoside triphosphate diphosphohydrolase 7 (ENTPD7), mRNA [NM_020354] |
| C9orf93 | NM_173550 | chromosome 9 open reading frame 93 (C9orf93), mRNA [NM_173550] |
| XLOC_002153 | TCONS_00004303 | linc|BROAD Institute lincRNA (XLOC_002153), lincRNA [TCONS_00004303] |
| RSPH4A | NM_001010892 | radial spoke head 4 homolog A (Chlamydomonas) (RSPH4A), transcript variant 1, mRNA [NM_001010892] |
| NAT1 | NM_000662 | N-acetyltransferase 1 (arylamine N-acetyltransferase) (NAT1), transcript variant 5, mRNA [NM_000662] |
| A_33_P3334121 | A_33_P3334121 | Unknown |
| USP8 | NM_005154 | ubiquitin specific peptidase 8 (USP8), transcript variant 1, mRNA [NM_005154] |
| BC028053 | BC028053 | gb|Homo sapiens cDNA clone IMAGE:4994693. [BC028053] |
| TOM1L1 | NM_005486 | target of myb1 (chicken)-like 1 (TOM1L1), mRNA [NM_005486] |
| MSTN | NM_005259 | myostatin (MSTN), mRNA [NM_005259] |
| ADAT1 | NM_012091 | adenosine deaminase, tRNA-specific 1 (ADAT1), transcript variant 1, mRNA [NM_012091] |
| FAM89A | NM_198552 | family with sequence similarity 89, member A (FAM89A), mRNA [NM_198552] |
| CDC37L1 | NM_017913 | cell division cycle 37 homolog (S. cerevisiae)-like 1 (CDC37L1), mRNA [NM_017913] |
| ZIC5 | NM_033132 | Zic family member 5 (ZIC5), mRNA [NM_033132] |
| MLL | AK021845 | gb|Homo sapiens cDNA FLJ11783 fis, clone HEMBA1006005. [AK021845] |
| XLOC_010664 | ENST00000446391 | gb|zt72a06.r1 Soares_testis_NHT Homo sapiens cDNA clone IMAGE:727858 5' similar to contains L1.t3 L1 repetitive element ;, mRNA sequence [AA393556] |
| GORAB | NM_001146039 | golgin, RAB6-interacting (GORAB), transcript variant 3, mRNA [NM_001146039] |
| DSE | NM_013352 | dermatan sulfate epimerase (DSE), transcript variant 1, mRNA [NM_013352] |
| HIST1H3I | NM_003533 | histone cluster 1, H3i (HIST1H3I), mRNA [NM_003533] |
| MGC16025 | NR_026664 | uncharacterized LOC85009 (MGC16025), non-coding RNA [NR_026664] |
| NFIA | NM_001134673 | nuclear factor I/A (NFIA), transcript variant 1, mRNA [NM_001134673] |
| JMY | NM_152405 | junction mediating and regulatory protein, p53 cofactor (JMY), mRNA [NM_152405] |
| SFSWAP | NM_004592 | splicing factor, suppressor of white-apricot homolog (Drosophila) (SFSWAP), mRNA [NM_004592] |
| LOC285547 | NR_034054 | uncharacterized LOC285547 (LOC285547), non-coding RNA [NR_034054] |
| MSH6 | NM_000179 | mutS homolog 6 (E. coli) (MSH6), mRNA [NM_000179] |
| FREM1 | ENST00000497634 | ens|FRAS1 related extracellular matrix 1 [Source:HGNC Symbol;Acc:23399] [ENST00000497634] |
| CDC42SE2 | ENST00000515533 | linc|BROAD Institute lincRNA (XLOC_004548), lincRNA [TCONS_00010090] |
| FAM86B1 | NM_001083537 | family with sequence similarity 86, member B1 (FAM86B1), transcript variant 1, mRNA [NM_001083537] |
| XLOC_008111 | AY660578 | linc|BROAD Institute lincRNA (XLOC_008111), lincRNA [TCONS_00016946] |
| OTX1 | NM_001199770 | orthodenticle homeobox 1 (OTX1), transcript variant 2, mRNA [NM_001199770] |
| SNORA5C | NR_002991 | small nucleolar RNA, H/ACA box 5C (SNORA5C), small nucleolar RNA [NR_002991] |
| PPP1CC | NM_002710 | protein phosphatase 1, catalytic subunit, gamma isozyme (PPP1CC), transcript variant 1, mRNA [NM_002710] |
| EFHD1 | NM_025202 | EF-hand domain family, member D1 (EFHD1), transcript variant 1, mRNA [NM_025202] |
| ZSWIM6 | NM_020928 | zinc finger, SWIM-type containing 6 (ZSWIM6), mRNA [NM_020928] |
| ZNF596 | NM_001042416 | zinc finger protein 596 (ZNF596), transcript variant 1, mRNA [NM_001042416] |
| XLOC_006079 | BC042682 | tc|KCC2B_HUMAN (Q13554) Calcium/calmodulin-dependent protein kinase type II beta chain (CaM-kinase II beta chain) (CaM kinase II beta subunit) (CaMK-II beta subunit) , partial (3%) [THC2611356] |
| ENST00000371162 | ENST00000371162 | ref|PREDICTED: Homo sapiens hypothetical LOC100128130 (LOC100128130), miscRNA [XR_109904] |
| SNORD104 | NR_004380 | small nucleolar RNA, C/D box 104 (SNORD104), small nucleolar RNA [NR_004380] |
| SNORA56 | NR_002984 | small nucleolar RNA, H/ACA box 56 (SNORA56), small nucleolar RNA [NR_002984] |
| GULP1 | NM_016315 | GULP, engulfment adaptor PTB domain containing 1 (GULP1), mRNA [NM_016315] |
| AF289601 | AF289601 | gb|Homo sapiens clone pp8142 unknown mRNA. [AF289601] |
| SOCS2 | NM_003877 | suppressor of cytokine signaling 2 (SOCS2), mRNA [NM_003877] |
| BDP1 | NM_018429 | B double prime 1, subunit of RNA polymerase III transcription initiation factor IIIB (BDP1), mRNA [NM_018429] |
| SEPP1 | NM_005410 | selenoprotein P, plasma, 1 (SEPP1), transcript variant 1, mRNA [NM_005410] |
| DCLK3 | NM_033403 | doublecortin-like kinase 3 (DCLK3), mRNA [NM_033403] |
| GPX8 | NM_001008397 | glutathione peroxidase 8 (putative) (GPX8), mRNA [NM_001008397] |
| VPRBP | NM_014703 | Vpr (HIV-1) binding protein (VPRBP), transcript variant 1, mRNA [NM_014703] |
| DST | NM_015548 | dystonin (DST), transcript variant 1eA, mRNA [NM_015548] |
| IGSF6 | NM_005849 | immunoglobulin superfamily, member 6 (IGSF6), mRNA [NM_005849] |
| TTLL13 | NM_001029964 | tubulin tyrosine ligase-like family, member 13 (TTLL13), mRNA [NM_001029964] |
| XLOC_004727 | ENST00000504765 | linc|BROAD Institute lincRNA (XLOC_004727), lincRNA [TCONS_00009599] |
| BET1L | NM_016526 | blocked early in transport 1 homolog (S. cerevisiae)-like (BET1L), transcript variant 2, mRNA [NM_016526] |
| CFI | NM_000204 | complement factor I (CFI), mRNA [NM_000204] |
| XLOC_010548 | TCONS_00021951 | linc|BROAD Institute lincRNA (XLOC_010548), lincRNA [TCONS_00021951] |
| ANAPC7 | NM_016238 | anaphase promoting complex subunit 7 (ANAPC7), transcript variant 1, mRNA [NM_016238] |
| HIST1H2AD | NM_021065 | histone cluster 1, H2ad (HIST1H2AD), mRNA [NM_021065] |
| CALCA | NM_001033952 | calcitonin-related polypeptide alpha (CALCA), transcript variant 2, mRNA [NM_001033952] |
| ZNF283 | NM_181845 | zinc finger protein 283 (ZNF283), mRNA [NM_181845] |
| XLOC_004864 | BC039391 | linc|BROAD Institute lincRNA (XLOC_004864), lincRNA [TCONS_00009286] |
| ISLR2 | NM_020851 | immunoglobulin superfamily containing leucine-rich repeat 2 (ISLR2), transcript variant 2, mRNA [NM_020851] |
| MRP63 | NM_024026 | mitochondrial ribosomal protein 63 (MRP63), nuclear gene encoding mitochondrial protein, mRNA [NM_024026] |
| FER | NM_005246 | fer (fps/fes related) tyrosine kinase (FER), mRNA [NM_005246] |
| LRAT | NM_004744 | lecithin retinol acyltransferase (phosphatidylcholine--retinol O-acyltransferase) (LRAT), mRNA [NM_004744] |
| SOX2-OT | NR_004053 | SOX2 overlapping transcript (non-protein coding) (SOX2-OT), non-coding RNA [NR_004053] |
| CCL4 | NM_002984 | chemokine (C-C motif) ligand 4 (CCL4), transcript variant 1, mRNA [NM_002984] |
| LOC100505516 | XR_108351 | ref|PREDICTED: Homo sapiens hypothetical LOC100505516 (LOC100505516), miscRNA [XR_108351] |
| LOC100131015 | AK124509 | gb|Homo sapiens cDNA FLJ42518 fis, clone BRACE3000697. [AK124509] |
| ATPBD4 | NM_080650 | ATP binding domain 4 (ATPBD4), transcript variant 1, mRNA [NM_080650] |
| STMN4 | NM_030795 | stathmin-like 4 (STMN4), mRNA [NM_030795] |
| XLOC_002969 | TCONS_00006350 | linc|BROAD Institute lincRNA (XLOC_002969), lincRNA [TCONS_00006350] |
| TSC22D1 | NM_183422 | TSC22 domain family, member 1 (TSC22D1), transcript variant 1, mRNA [NM_183422] |
| HNRNPK | NM_002140 | heterogeneous nuclear ribonucleoprotein K (HNRNPK), transcript variant 1, mRNA [NM_002140] |
| FBXO38 | NM_205836 | F-box protein 38 (FBXO38), transcript variant 2, mRNA [NM_205836] |
| IQCJ-SCHIP1 | NM_001197113 | IQCJ-SCHIP1 readthrough (IQCJ-SCHIP1), transcript variant 1, mRNA [NM_001197113] |
| SCRN3 | NM_024583 | secernin 3 (SCRN3), transcript variant 1, mRNA [NM_024583] |
| SNORD59B | NR_003046 | small nucleolar RNA, C/D box 59B (SNORD59B), small nucleolar RNA [NR_003046] |
| NFXL1 | NM_152995 | nuclear transcription factor, X-box binding-like 1 (NFXL1), mRNA [NM_152995] |
| PRMT6 | NM_018137 | protein arginine methyltransferase 6 (PRMT6), mRNA [NM_018137] |
| A_33_P3295705 | A_33_P3295705 | Unknown |
| LRP11 | ENST00000367368 | ens|low density lipoprotein receptor-related protein 11 [Source:HGNC Symbol;Acc:16936] [ENST00000367368] |
| MGC16142 | NR_026902 | uncharacterized protein MGC16142 (MGC16142), non-coding RNA [NR_026902] |
| THC2532340 | THC2532340 | tc|BC012612 cathepsin L, preproprotein {Homo sapiens} (exp=-1; wgp=0; cg=0), partial (54%) [THC2532340] |
| VDR | NM_001017535 | vitamin D (1,25- dihydroxyvitamin D3) receptor (VDR), transcript variant 2, mRNA [NM_001017535] |
| RAD51B | NM_133509 | RAD51 homolog B (S. cerevisiae) (RAD51B), transcript variant 3, mRNA [NM_133509] |
| B3GNT3 | NM_014256 | UDP-GlcNAc:betaGal beta-1,3-N-acetylglucosaminyltransferase 3 (B3GNT3), mRNA [NM_014256] |
| POM121 | ENST00000395270 | ens|POM121 membrane glycoprotein [Source:HGNC Symbol;Acc:19702] [ENST00000395270] |
| ZNF818P | AK128250 | gb|Homo sapiens cDNA FLJ46385 fis, clone THYMU2038199. [AK128250] |
| XLOC_009578 | ENST00000526934 | linc|BROAD Institute lincRNA (XLOC_009578), lincRNA [TCONS_00020140] |
| PHF23 | NM_024297 | PHD finger protein 23 (PHF23), mRNA [NM_024297] |
| XLOC_002069 | ENST00000438736 | gb|DB066812 TESTI4 Homo sapiens cDNA clone TESTI4008254 5', mRNA sequence [DB066812] |
| ZNF571 | NM_016536 | zinc finger protein 571 (ZNF571), mRNA [NM_016536] |
| XLOC_l2_011531 | ENST00000503577 | gb|DKFZp686F24249_r1 686 (synonym: hlcc3) Homo sapiens cDNA clone DKFZp686F24249 5', mRNA sequence [BX485935] |
| SLCO1B7 | NM_001009562 | solute carrier organic anion transporter family, member 1B7 (non-functional) (SLCO1B7), mRNA [NM_001009562] |
| A_33_P3213526 | A_33_P3213526 | Unknown |
| ECHDC1 | ENST00000368287 | ens|enoyl CoA hydratase domain containing 1 [Source:HGNC Symbol;Acc:21489] [ENST00000368287] |
| XLOC_003658 | ENST00000426240 | linc|BROAD Institute lincRNA (XLOC_003658), lincRNA [TCONS_00007597] |
| A_33_P3315659 | A_33_P3315659 | Unknown |
| C14orf99 | AL043142 | gb|DKFZp434E1423_r1 434 (synonym: htes3) Homo sapiens cDNA clone DKFZp434E1423 5', mRNA sequence [AL043142] |
| HIST1H2AG | NM_021064 | histone cluster 1, H2ag (HIST1H2AG), mRNA [NM_021064] |
| GCLC | NM_001498 | glutamate-cysteine ligase, catalytic subunit (GCLC), transcript variant 1, mRNA [NM_001498] |
| POLQ | NM_199420 | polymerase (DNA directed), theta (POLQ), mRNA [NM_199420] |
| NUDT11 | NM_018159 | nudix (nucleoside diphosphate linked moiety X)-type motif 11 (NUDT11), mRNA [NM_018159] |
| CCDC137 | NM_199287 | coiled-coil domain containing 137 (CCDC137), mRNA [NM_199287] |
| LOC100271831 | NR_027081 | uncharacterized LOC100271831 (LOC100271831), non-coding RNA [NR_027081] |
| ENST00000390319 | ENST00000390319 | ens|immunoglobulin lambda variable 3-1 [Source:HGNC Symbol;Acc:5896] [ENST00000390319] |
| XLOC_009426 | TCONS_00019625 | linc|BROAD Institute lincRNA (XLOC_009426), lincRNA [TCONS_00019625] |
| ZNF547 | NM_173631 | zinc finger protein 547 (ZNF547), mRNA [NM_173631] |
| XLOC_l2_014757 | TCONS_l2_00028700 | linc|BROAD Institute lincRNA (XLOC_l2_014757), lincRNA [TCONS_l2_00028700] |
| DBF4B | NM_145663 | DBF4 homolog B (S. cerevisiae) (DBF4B), transcript variant 1, mRNA [NM_145663] |
| C17orf39 | NM_024052 | chromosome 17 open reading frame 39 (C17orf39), mRNA [NM_024052] |
| LOC100506190 | NR_038955 | uncharacterized LOC100506190 (LOC100506190), non-coding RNA [NR_038955] |
| PRPF4 | NM_004697 | PRP4 pre-mRNA processing factor 4 homolog (yeast) (PRPF4), transcript variant 1, mRNA [NM_004697] |
| ACP1 | NM_001040649 | acid phosphatase 1, soluble (ACP1), transcript variant 4, mRNA [NM_001040649] |
| XLOC_007913 | TCONS_00017120 | linc|BROAD Institute lincRNA (XLOC_007913), lincRNA [TCONS_00017120] |
| HIST1H2AI | NM_003509 | histone cluster 1, H2ai (HIST1H2AI), mRNA [NM_003509] |
| A_33_P3305775 | A_33_P3305775 | Unknown |
| STX18 | NM_016930 | syntaxin 18 (STX18), mRNA [NM_016930] |
| XLOC_002066 | TCONS_00004223 | linc|BROAD Institute lincRNA (XLOC_002066), lincRNA [TCONS_00004223] |
| LOC100507637 | AK026502 | tc|Q41XT8_DESHA (Q41XT8) Dihydropteroate synthase , partial (6%) [THC2523212] |
| XLOC_l2_015011 | ENST00000468244 | tc|Q351Q8_9GAMM (Q351Q8) Peptidase S41A, C-terminal protease , partial (3%) [THC2684243] |
| LOC572558 | CF127520 | gb|UI-HF-ET0-awh-f-16-0-UI.r1 NIH_MGC_214 Homo sapiens cDNA clone IMAGE:30554535 5', mRNA sequence [CF127520] |
| LOC653581 | BC009864 | gb|Homo sapiens TANK-binding kinase 1, mRNA (cDNA clone IMAGE:3938647), partial cds. [BC009864] |
| XLOC_l2_015964 | TCONS_l2_00030952 | linc|BROAD Institute lincRNA (XLOC_l2_015964), lincRNA [TCONS_l2_00030952] |
| AK002210 | AK002210 | gb|Homo sapiens cDNA FLJ11348 fis, clone PLACE4000638. [AK002210] |
| AK125099 | AK125099 | gb|Homo sapiens cDNA FLJ43109 fis, clone CTONG2025516, moderately similar to Homo sapiens general transcription factor II, i (GTF2I). [AK125099] |
| XLOC_002362 | ENST00000442706 | gb|qe05g10.x1 Soares_testis_NHT Homo sapiens cDNA clone IMAGE:1738146 3', mRNA sequence [AI140623] |
| XLOC_001496 | TCONS_00003709 | linc|BROAD Institute lincRNA (XLOC_001496), lincRNA [TCONS_00003709] |
| ALG5 | NM_013338 | asparagine-linked glycosylation 5, dolichyl-phosphate beta-glucosyltransferase homolog (S. cerevisiae) (ALG5), transcript variant 1, mRNA [NM_013338] |
| TMEM209 | NM_032842 | transmembrane protein 209 (TMEM209), mRNA [NM_032842] |
| RASEF | ENST00000340717 | ens|RAS and EF-hand domain containing [Source:HGNC Symbol;Acc:26464] [ENST00000340717] |
| PSMD5 | ENST00000373903 | ens|proteasome (prosome, macropain) 26S subunit, non-ATPase, 5 [Source:HGNC Symbol;Acc:9563] [ENST00000373903] |
| LOC100507007 | ENST00000439938 | ref|PREDICTED: Homo sapiens hypothetical LOC100507007 (LOC100507007), miscRNA [XR_110301] |
| INO80 | NM_017553 | INO80 homolog (S. cerevisiae) (INO80), mRNA [NM_017553] |
| PITPNB | NM_012399 | phosphatidylinositol transfer protein, beta (PITPNB), mRNA [NM_012399] |
| SPTLC1 | NM_178324 | serine palmitoyltransferase, long chain base subunit 1 (SPTLC1), transcript variant 2, mRNA [NM_178324] |
| XLOC_009684 | TCONS_00020354 | linc|BROAD Institute lincRNA (XLOC_009684), lincRNA [TCONS_00020354] |
| SNX29 | NM_032167 | sorting nexin 29 (SNX29), mRNA [NM_032167] |
| PERP | NM_022121 | PERP, TP53 apoptosis effector (PERP), mRNA [NM_022121] |
| BX115986 | BX115986 | gb|BX115986 Soares_testis_NHT Homo sapiens cDNA clone IMAGp998H122622, mRNA sequence [BX115986] |
| LOC391764 | XM_001713926 | ref|PREDICTED: Homo sapiens putative TAF11-like protein ENSP00000332601-like (LOC391764), mRNA [XM_001713926] |
| XLOC_013773 | TCONS_00028422 | linc|BROAD Institute lincRNA (XLOC_013773), lincRNA [TCONS_00028422] |
| Q07610 | THC2621400 | tc|Q07610_RAT (Q07610) Proline-rich proteoglycan, partial (7%) [THC2621400] |
| XLOC_004323 | BC101438 | Unknown |
| SNORD38A | NR_001456 | small nucleolar RNA, C/D box 38A (SNORD38A), small nucleolar RNA [NR_001456] |
| C14orf102 | NM_017970 | chromosome 14 open reading frame 102 (C14orf102), mRNA [NM_017970] |
| ZNF674 | NM_001039891 | zinc finger protein 674 (ZNF674), transcript variant 1, mRNA [NM_001039891] |
| ENST00000397584 | ENST00000397584 | gb|Homo sapiens pp12708 mRNA, complete cds. [AF318327] |
| ORC6 | NM_014321 | origin recognition complex, subunit 6 (ORC6), transcript variant 1, mRNA [NM_014321] |
| KCTD6 | NM_153331 | potassium channel tetramerisation domain containing 6 (KCTD6), transcript variant 1, mRNA [NM_153331] |
| XLOC_l2_001462 | ENST00000420049 | linc|BROAD Institute lincRNA (XLOC_l2_001462), lincRNA [TCONS_l2_00002879] |
| XLOC_006139 | ENST00000444745 | gb|BX116805 Soares_testis_NHT Homo sapiens cDNA clone IMAGp998B204413, mRNA sequence [BX116805] |
| LOC100129072 | AK123881 | gb|Homo sapiens cDNA FLJ41887 fis, clone OCBBF2023643. [AK123881] |
| XLOC_l2_015397 | BC014023 | linc|BROAD Institute lincRNA (XLOC_l2_015397), lincRNA [TCONS_l2_00030085] |
| TUBD1 | NM_016261 | tubulin, delta 1 (TUBD1), transcript variant 1, mRNA [NM_016261] |
| XLOC_009248 | ENST00000525832 | linc|BROAD Institute lincRNA (XLOC_009248), lincRNA [TCONS_00019437] |
| LOC401109 | NR_034088 | uncharacterized LOC401109 (LOC401109), non-coding RNA [NR_034088] |
| XLOC_007842 | ENST00000450292 | gb|BX109549 Soares_testis_NHT Homo sapiens cDNA clone IMAGp998N151795, mRNA sequence [BX109549] |
| XLOC_l2_003419 | TCONS_l2_00006314 | linc|BROAD Institute lincRNA (XLOC_l2_003419), lincRNA [TCONS_l2_00006314] |
| C6orf47 | NM_021184 | chromosome 6 open reading frame 47 (C6orf47), mRNA [NM_021184] |
| XLOC_l2_003860 | ENST00000422994 | linc|BROAD Institute lincRNA (XLOC_l2_003860), lincRNA [TCONS_l2_00007012] |
| XLOC_011112 | AK123359 | gb|Homo sapiens mRNA; cDNA DKFZp667M1023 (from clone DKFZp667M1023) [AL832802] |
| XLOC_013955 | TCONS_00029036 | linc|BROAD Institute lincRNA (XLOC_013955), lincRNA [TCONS_00029036] |
| N95477 | N95477 | gb|zb81h09.s1 Soares_senescent_fibroblasts_NbHSF Homo sapiens cDNA clone IMAGE:310049 3' similar to contains element MER22 repetitive element ;, mRNA sequence [N95477] |
| LOC100129112 | AK123839 | gb|Homo sapiens cDNA FLJ41845 fis, clone NT2RI3003095. [AK123839] |
| BEND4 | NM_207406 | BEN domain containing 4 (BEND4), transcript variant 1, mRNA [NM_207406] |
| TP53TG5 | NM_014477 | TP53 target 5 (TP53TG5), mRNA [NM_014477] |
| A_33_P3278501 | A_33_P3278501 | Unknown |
| XLOC_010111 | TCONS_00020823 | linc|BROAD Institute lincRNA (XLOC_010111), lincRNA [TCONS_00020823] |
| HBS1L | NM_006620 | HBS1-like (S. cerevisiae) (HBS1L), transcript variant 1, mRNA [NM_006620] |
| RPL32P3 | AK096589 | tc|Q9HAT9_HUMAN (Q9HAT9) SPG protein, partial (3%) [THC2509642] |
| MYEOV2 | NM_138336 | myeloma overexpressed 2 (MYEOV2), transcript variant 1, mRNA [NM_138336] |
| AFAP1-AS1 | NR_026892 | AFAP1 antisense RNA 1 (non-protein coding) (AFAP1-AS1), antisense RNA [NR_026892] |
| MIA3 | NM_198551 | melanoma inhibitory activity family, member 3 (MIA3), mRNA [NM_198551] |
| UGT2A3 | NM_024743 | UDP glucuronosyltransferase 2 family, polypeptide A3 (UGT2A3), mRNA [NM_024743] |
| LOC729324 | AK055581 | gb|Homo sapiens cDNA FLJ31019 fis, clone HLUNG2000362. [AK055581] |
| PLEKHG6 | NM_018173 | pleckstrin homology domain containing, family G (with RhoGef domain) member 6 (PLEKHG6), transcript variant 1, mRNA [NM_018173] |
| XLOC_012732 | TCONS_00026381 | linc|BROAD Institute lincRNA (XLOC_012732), lincRNA [TCONS_00026381] |
| LOC100505851 | XR_109588 | ref|PREDICTED: Homo sapiens hypothetical LOC100505851 (LOC100505851), miscRNA [XR_109588] |
| FAM54A | NM_138419 | family with sequence similarity 54, member A (FAM54A), transcript variant 2, mRNA [NM_138419] |
| POLR3C | NM_006468 | polymerase (RNA) III (DNA directed) polypeptide C (62kD) (POLR3C), mRNA [NM_006468] |
| RORB | NM_006914 | RAR-related orphan receptor B (RORB), mRNA [NM_006914] |
| SPATA5L1 | AK022348 | gb|Homo sapiens cDNA FLJ12286 fis, clone MAMMA1001768, weakly similar to CELL DIVISION CYCLE PROTEIN 48 HOMOLOG MJ1156. [AK022348] |
| LRRC69 | NM_001129890 | leucine rich repeat containing 69 (LRRC69), mRNA [NM_001129890] |
| PRSS27 | NM_031948 | protease, serine 27 (PRSS27), mRNA [NM_031948] |
| SPTBN1 | NM_178313 | spectrin, beta, non-erythrocytic 1 (SPTBN1), transcript variant 2, mRNA [NM_178313] |
| A_33_P3227300 | A_33_P3227300 | Unknown |
| XLOC_010610 | TCONS_00022282 | linc|BROAD Institute lincRNA (XLOC_010610), lincRNA [TCONS_00022282] |
| LYVE1 | NM_006691 | lymphatic vessel endothelial hyaluronan receptor 1 (LYVE1), mRNA [NM_006691] |
| XLOC_001558 | BC036617 | linc|BROAD Institute lincRNA (XLOC_001558), lincRNA [TCONS_00004837] |
| ZNF273 | NM_021148 | zinc finger protein 273 (ZNF273), transcript variant 1, mRNA [NM_021148] |
| LOC100652761 | XR_132756 | ref|PREDICTED: Homo sapiens hypothetical LOC100652761 (LOC100652761), miscRNA [XR_132756] |
| BANP | NM_017869 | BTG3 associated nuclear protein (BANP), transcript variant 1, mRNA [NM_017869] |
| DYNC2H1 | NM_001080463 | dynein, cytoplasmic 2, heavy chain 1 (DYNC2H1), transcript variant 2, mRNA [NM_001080463] |
| MAGEE2 | NM_138703 | melanoma antigen family E, 2 (MAGEE2), mRNA [NM_138703] |
| SLC7A8 | NM_182728 | solute carrier family 7 (amino acid transporter light chain, L system), member 8 (SLC7A8), transcript variant 2, mRNA [NM_182728] |
| LOC644714 | NR_033947 | uncharacterized LOC644714 (LOC644714), non-coding RNA [NR_033947] |
| ZFP161 | NM_003409 | zinc finger protein 161 homolog (mouse) (ZFP161), transcript variant 2, mRNA [NM_003409] |
| C3orf43 | NM_001077657 | chromosome 3 open reading frame 43 (C3orf43), mRNA [NM_001077657] |
| SFRP1 | NM_003012 | secreted frizzled-related protein 1 (SFRP1), mRNA [NM_003012] |
| DDB1 | ENST00000545894 | ens|damage-specific DNA binding protein 1, 127kDa [Source:HGNC Symbol;Acc:2717] [ENST00000545894] |
| MCART1 | NR_024872 | mitochondrial carrier triple repeat 1 (MCART1), transcript variant 2, non-coding RNA [NR_024872] |
| XLOC_l2_012397 | BC030116 | tc|Q5TBT5_HUMAN (Q5TBT5) OTTHUMP00000018489 (Fragment), partial (28%) [THC2610149] |
| C6orf25 | NM_025260 | chromosome 6 open reading frame 25 (C6orf25), transcript variant 1, mRNA [NM_025260] |
| FNIP2 | NM_020840 | folliculin interacting protein 2 (FNIP2), mRNA [NM_020840] |
| MTMR10 | NM_017762 | myotubularin related protein 10 (MTMR10), mRNA [NM_017762] |
| STRN3 | NM_014574 | striatin, calmodulin binding protein 3 (STRN3), transcript variant 2, mRNA [NM_014574] |
| RAB5A | NM_004162 | RAB5A, member RAS oncogene family (RAB5A), mRNA [NM_004162] |
| A_33_P3375496 | A_33_P3375496 | Unknown |
| AATF | NM_012138 | apoptosis antagonizing transcription factor (AATF), mRNA [NM_012138] |
| XLOC_l2_007707 | ENST00000467633 | linc|BROAD Institute lincRNA (XLOC_l2_007707), lincRNA [TCONS_l2_00015573] |
| XLOC_007107 | TCONS_00015028 | linc|BROAD Institute lincRNA (XLOC_007107), lincRNA [TCONS_00015028] |
| MEAF6 | NM_022756 | MYST/Esa1-associated factor 6 (MEAF6), mRNA [NM_022756] |
| ZHX3 | NM_015035 | zinc fingers and homeoboxes 3 (ZHX3), mRNA [NM_015035] |
| XLOC_003136 | ENST00000472238 | gb|DA455243 CTONG2 Homo sapiens cDNA clone CTONG2028621 5', mRNA sequence [DA455243] |
| MYL10 | NM_138403 | myosin, light chain 10, regulatory (MYL10), mRNA [NM_138403] |
| LOC100506523 | XR_109504 | ref|PREDICTED: Homo sapiens hypothetical LOC100506523 (LOC100506523), miscRNA [XR_109504] |
| GDA | NM_004293 | guanine deaminase (GDA), transcript variant 2, mRNA [NM_004293] |
| TMEM60 | NM_032936 | transmembrane protein 60 (TMEM60), mRNA [NM_032936] |
| IFIT5 | NM_012420 | interferon-induced protein with tetratricopeptide repeats 5 (IFIT5), mRNA [NM_012420] |
| FLJ32224 | AK056786 | gb|Homo sapiens cDNA FLJ32224 fis, clone PLACE6004336. [AK056786] |
| HNRNPKP3 | NR_033868 | heterogeneous nuclear ribonucleoprotein K pseudogene 3 (HNRNPKP3), non-coding RNA [NR_033868] |
| THC2570839 | THC2570839 | tc|Q53Y51_HUMAN (Q53Y51) D-dopachrome tautomerase, partial (28%) [THC2570839] |
| SORBS2 | NM_021069 | sorbin and SH3 domain containing 2 (SORBS2), transcript variant 2, mRNA [NM_021069] |
| FAS-AS1 | NR_028371 | FAS antisense RNA 1 (non-protein coding) (FAS-AS1), non-coding RNA [NR_028371] |
| FEM1B | NM_015322 | fem-1 homolog b (C. elegans) (FEM1B), mRNA [NM_015322] |
| LOC100130539 | XM_001724322 | ref|PREDICTED: Homo sapiens hypothetical protein LOC100130539 (LOC100130539), mRNA [XM_001724322] |
| XLOC_002254 | ENST00000417284 | linc|BROAD Institute lincRNA (XLOC_002254), lincRNA [TCONS_00003363] |
| LOC729799 | NR_026952 | SEC14-like 1 pseudogene (LOC729799), non-coding RNA [NR_026952] |
| LOC100506312 | XR_108794 | ref|PREDICTED: Homo sapiens hypothetical LOC100506312 (LOC100506312), miscRNA [XR_108794] |
| RAB10 | NM_016131 | RAB10, member RAS oncogene family (RAB10), mRNA [NM_016131] |
| A_33_P3279181 | A_33_P3279181 | Unknown |
| ENST00000390373 | ENST00000390373 | ens|T cell receptor beta variable 6-7 (non-functional) [Source:HGNC Symbol;Acc:12232] [ENST00000390373] |
| EBI3 | NM_005755 | Epstein-Barr virus induced 3 (EBI3), mRNA [NM_005755] |
| SNORD11B | NR_003694 | small nucleolar RNA, C/D box 11B (SNORD11B), small nucleolar RNA [NR_003694] |
| LPCAT2 | NM_017839 | lysophosphatidylcholine acyltransferase 2 (LPCAT2), mRNA [NM_017839] |
| LOC100652779 | XR_132723 | ref|PREDICTED: Homo sapiens hypothetical LOC100652779 (LOC100652779), miscRNA [XR_132723] |
| AK074144 | AK074144 | gb|Homo sapiens mRNA for FLJ00217 protein. [AK074144] |
| RALGAPA2 | NM_020343 | Ral GTPase activating protein, alpha subunit 2 (catalytic) (RALGAPA2), mRNA [NM_020343] |
| COL4A1 | NM_001845 | collagen, type IV, alpha 1 (COL4A1), mRNA [NM_001845] |
| XLOC_l2_013125 | ENST00000412602 | linc|BROAD Institute lincRNA (XLOC_l2_013125), lincRNA [TCONS_l2_00025481] |
| SNORA68 | NR_000012 | small nucleolar RNA, H/ACA box 68 (SNORA68), small nucleolar RNA [NR_000012] |
| TRMT61B | NM_017910 | tRNA methyltransferase 61 homolog B (S. cerevisiae) (TRMT61B), mRNA [NM_017910] |
| TMEM72-AS1 | NR_033842 | TMEM72 antisense RNA 1 (non-protein coding) (TMEM72-AS1), non-coding RNA [NR_033842] |
| ZNF14 | NM_021030 | zinc finger protein 14 (ZNF14), mRNA [NM_021030] |
| SVIL | NM_021738 | supervillin (SVIL), transcript variant 2, mRNA [NM_021738] |
| XLOC_l2_001971 | ENST00000532854 | linc|BROAD Institute lincRNA (XLOC_l2_001971), lincRNA [TCONS_l2_00003557] |
| HDHD1 | NM_001178135 | haloacid dehalogenase-like hydrolase domain containing 1 (HDHD1), transcript variant 3, mRNA [NM_001178135] |
| ADD2 | NM_001617 | adducin 2 (beta) (ADD2), transcript variant 1, mRNA [NM_001617] |
| RAB40B | NM_006822 | RAB40B, member RAS oncogene family (RAB40B), mRNA [NM_006822] |
| ANXA13 | NM_001003954 | annexin A13 (ANXA13), transcript variant 2, mRNA [NM_001003954] |
| RNGTT | ENST00000369475 | ens|RNA guanylyltransferase and 5'-phosphatase [Source:HGNC Symbol;Acc:10073] [ENST00000369475] |
| ERICH1 | NM_207332 | glutamate-rich 1 (ERICH1), mRNA [NM_207332] |
| FIGNL2 | NM_001013690 | fidgetin-like 2 (FIGNL2), mRNA [NM_001013690] |
| CAPZB | ENST00000264203 | ens|capping protein (actin filament) muscle Z-line, beta [Source:HGNC Symbol;Acc:1491] [ENST00000264203] |
| PEX13 | NM_002618 | peroxisomal biogenesis factor 13 (PEX13), mRNA [NM_002618] |
| NBPF1 | NM_017940 | neuroblastoma breakpoint family, member 1 (NBPF1), mRNA [NM_017940] |
| XLOC_002622 | ENST00000412811 | gb|DA970873 SYNOV2 Homo sapiens cDNA clone SYNOV2000150 5', mRNA sequence [DA970873] |
| C8orf77 | NR_026974 | chromosome 8 open reading frame 77 (C8orf77), non-coding RNA [NR_026974] |
| KLHL8 | NM_020803 | kelch-like 8 (Drosophila) (KLHL8), mRNA [NM_020803] |
| XLOC_014514 | BC012753 | tc|ALU1_HUMAN (P39188) Alu subfamily J sequence contamination warning entry, partial (8%) [THC2558572] |
| ENST00000443922 | ENST00000443922 | Unknown |
| SEC63 | NM_007214 | SEC63 homolog (S. cerevisiae) (SEC63), mRNA [NM_007214] |
| SLC26A4 | NM_000441 | solute carrier family 26, member 4 (SLC26A4), mRNA [NM_000441] |
| G2E3 | NM_017769 | G2/M-phase specific E3 ubiquitin protein ligase (G2E3), mRNA [NM_017769] |
| HACL1 | NM_012260 | 2-hydroxyacyl-CoA lyase 1 (HACL1), mRNA [NM_012260] |
| NDST3 | ENST00000394488 | ens|N-deacetylase/N-sulfotransferase (heparan glucosaminyl) 3 [Source:HGNC Symbol;Acc:7682] [ENST00000394488] |
| MCTP2 | NM_018349 | multiple C2 domains, transmembrane 2 (MCTP2), transcript variant 1, mRNA [NM_018349] |
| XLOC_005244 | THC2553854 | linc|BROAD Institute lincRNA (XLOC_005244), lincRNA [TCONS_00011775] |
| MED10 | NM_032286 | mediator complex subunit 10 (MED10), mRNA [NM_032286] |
| GPR87 | NM_023915 | G protein-coupled receptor 87 (GPR87), mRNA [NM_023915] |
| BMS1P1 | NR_026566 | BMS1 pseudogene 1 (BMS1P1), non-coding RNA [NR_026566] |
| XLOC_008645 | THC2702642 | linc|BROAD Institute lincRNA (XLOC_008645), lincRNA [TCONS_00018842] |
| XLOC_l2_011173 | ENST00000514050 | linc|BROAD Institute lincRNA (XLOC_l2_011173), lincRNA [TCONS_l2_00021909] |
| FAM86C2P | NR_024249 | family with sequence similarity 86, member C2, pseudogene (FAM86C2P), non-coding RNA [NR_024249] |
| BAMBI | NM_012342 | BMP and activin membrane-bound inhibitor homolog (Xenopus laevis) (BAMBI), mRNA [NM_012342] |
| XLOC_l2_012082 | AK130833 | linc|BROAD Institute lincRNA (XLOC_l2_012082), lincRNA [TCONS_l2_00023776] |
| PCGF6 | NM_001011663 | polycomb group ring finger 6 (PCGF6), transcript variant 1, mRNA [NM_001011663] |
| LOC541467 | BC045815 | gb|Homo sapiens hypothetical LOC541467, mRNA (cDNA clone IMAGE:4830703), partial cds. [BC045815] |
| XLOC_l2_006648 | ENST00000360204 | tc|GB|BC067843.1|BC067843.1 Homo sapiens cDNA clone IMAGE:5270688, partial cds [NP1133934] |
| XLOC_013946 | ENST00000419826 | gb|full-length cDNA clone CS0DI026YO17 of Placenta Cot 25-normalized of Homo sapiens (human) [CR624487] |
| FLJ41327 | AK123321 | gb|Homo sapiens cDNA FLJ41327 fis, clone BRAMY2047169. [AK123321] |
| ST8SIA1 | NM_003034 | ST8 alpha-N-acetyl-neuraminide alpha-2,8-sialyltransferase 1 (ST8SIA1), mRNA [NM_003034] |
| HIST1H3G | NM_003534 | histone cluster 1, H3g (HIST1H3G), mRNA [NM_003534] |
| XLOC_l2_009929 | ENST00000481334 | linc|BROAD Institute lincRNA (XLOC_l2_009929), lincRNA [TCONS_l2_00019925] |
| ENOX2 | NM_182314 | ecto-NOX disulfide-thiol exchanger 2 (ENOX2), transcript variant 2, mRNA [NM_182314] |
| XLOC_004804 | TCONS_00010323 | linc|BROAD Institute lincRNA (XLOC_004804), lincRNA [TCONS_00010323] |
| ENST00000357412 | ENST00000357412 | ens|chromosome X open reading frame 24 [Source:HGNC Symbol;Acc:27333] [ENST00000357412] |
| CCDC165 | NM_015210 | coiled-coil domain containing 165 (CCDC165), mRNA [NM_015210] |
| C19orf34 | NR_033400 | chromosome 19 open reading frame 34 (C19orf34), non-coding RNA [NR_033400] |
| RNF151 | NM_174903 | ring finger protein 151 (RNF151), mRNA [NM_174903] |
| ENST00000390304 | ENST00000390304 | ens|immunoglobulin lambda variable 3-27 [Source:HGNC Symbol;Acc:5910] [ENST00000390304] |
| XLOC_008402 | TCONS_00018127 | linc|BROAD Institute lincRNA (XLOC_008402), lincRNA [TCONS_00018127] |
| ATG5 | NM_004849 | ATG5 autophagy related 5 homolog (S. cerevisiae) (ATG5), mRNA [NM_004849] |
| FLJ42393 | NR_024413 | uncharacterized LOC401105 (FLJ42393), non-coding RNA [NR_024413] |
| XLOC_002174 | BC030125 | tc|Q86U25_HUMAN (Q86U25) Full-length cDNA clone CS0DA007YG23 of Neuroblastoma of Homo sapiens (human), partial (18%) [THC2606856] |
| LILRB2 | NM_005874 | leukocyte immunoglobulin-like receptor, subfamily B (with TM and ITIM domains), member 2 (LILRB2), transcript variant 1, mRNA [NM_005874] |
| FAM21C | ENST00000374359 | ens|family with sequence similarity 21, member C [Source:HGNC Symbol;Acc:23414] [ENST00000374359] |
| MIS12 | NM_024039 | MIS12, MIND kinetochore complex component, homolog (S. pombe) (MIS12), mRNA [NM_024039] |
| STAT4 | NM_003151 | signal transducer and activator of transcription 4 (STAT4), transcript variant 1, mRNA [NM_003151] |
| RHEB | BC009638 | gb|Homo sapiens Ras homolog enriched in brain, mRNA (cDNA clone IMAGE:3892637), partial cds. [BC009638] |
| XLOC_004122 | ENST00000508847 | linc|BROAD Institute lincRNA (XLOC_004122), lincRNA [TCONS_00009146] |
| XLOC_011456 | TCONS_00023655 | linc|BROAD Institute lincRNA (XLOC_011456), lincRNA [TCONS_00023655] |
| XLOC_l2_004213 | TCONS_l2_00007764 | linc|BROAD Institute lincRNA (XLOC_l2_004213), lincRNA [TCONS_l2_00007764] |
| C1orf74 | NM_152485 | chromosome 1 open reading frame 74 (C1orf74), mRNA [NM_152485] |
| TSSK3 | NM_052841 | testis-specific serine kinase 3 (TSSK3), mRNA [NM_052841] |
| SLC35G1 | NM_153226 | solute carrier family 35, member G1 (SLC35G1), transcript variant 2, mRNA [NM_153226] |
| LOC100132764 | AK131376 | gb|Homo sapiens cDNA FLJ16434 fis, clone BRACE3015829. [AK131376] |
| LOC100653515 | NM_001243541 | differential display clone 8 (LOC100653515), transcript variant 1, mRNA [NM_001243541] |
| ENST00000398984 | ENST00000398984 | ens|membrane-spanning 4-domains, subfamily A, member 4E [Source:HGNC Symbol;Acc:14284] [ENST00000398984] |
| XLOC_010061 | TCONS_00021307 | linc|BROAD Institute lincRNA (XLOC_010061), lincRNA [TCONS_00021307] |
| XLOC_006379 | ENST00000414127 | linc|BROAD Institute lincRNA (XLOC_006379), lincRNA [TCONS_00013143] |
| ATP1A1OS | NR_027646 | ATP1A1 opposite strand (ATP1A1OS), transcript variant 1, non-coding RNA [NR_027646] |
| DB307521 | DB307521 | gb|DB307521 BRCOC2 Homo sapiens cDNA clone BRCOC2019652 3', mRNA sequence [DB307521] |
| XLOC_014003 | TCONS_00029079 | linc|BROAD Institute lincRNA (XLOC_014003), lincRNA [TCONS_00029079] |
| CHIC1 | NM_001039840 | cysteine-rich hydrophobic domain 1 (CHIC1), mRNA [NM_001039840] |
| A_33_P3276604 | A_33_P3276604 | Unknown |
| THC2577776 | THC2577776 | tc|HSENOAL2 alpha-enolase {Homo sapiens} (exp=-1; wgp=0; cg=0) , partial (78%) [THC2577776] |
| HSF5 | NM_001080439 | heat shock transcription factor family member 5 (HSF5), mRNA [NM_001080439] |
| ENST00000444375 | ENST00000444375 | tc|Q3RU97_RALME (Q3RU97) Sulfate transporter/antisigma-factor antagonist STAS:Sulphate transporter, partial (3%) [THC2519244] |
| DRAM1 | NM_018370 | DNA-damage regulated autophagy modulator 1 (DRAM1), mRNA [NM_018370] |
| MPO | NM_000250 | myeloperoxidase (MPO), nuclear gene encoding mitochondrial protein, mRNA [NM_000250] |
| ZNF391 | NM_001076781 | zinc finger protein 391 (ZNF391), mRNA [NM_001076781] |
| XLOC_007957 | TCONS_00017155 | linc|BROAD Institute lincRNA (XLOC_007957), lincRNA [TCONS_00017155] |
| XLOC_010389 | ENST00000435843 | linc|BROAD Institute lincRNA (XLOC_010389), lincRNA [TCONS_00021550] |
| TNFSF14 | NM_003807 | tumor necrosis factor (ligand) superfamily, member 14 (TNFSF14), transcript variant 1, mRNA [NM_003807] |
| LOC100131929 | AK126439 | gb|Homo sapiens cDNA FLJ44475 fis, clone UTERU2031521. [AK126439] |
| ZNF790 | NM_001242802 | zinc finger protein 790 (ZNF790), transcript variant 4, mRNA [NM_001242802] |
| HMGN1 | NM_004965 | high mobility group nucleosome binding domain 1 (HMGN1), mRNA [NM_004965] |
| CELF4 | NM_020180 | CUGBP, Elav-like family member 4 (CELF4), transcript variant 1, mRNA [NM_020180] |
| CACNA2D4 | NM_172364 | calcium channel, voltage-dependent, alpha 2/delta subunit 4 (CACNA2D4), mRNA [NM_172364] |
| ATXN7L1 | NM_020725 | ataxin 7-like 1 (ATXN7L1), transcript variant 1, mRNA [NM_020725] |
| SNORD5 | NR_003033 | small nucleolar RNA, C/D box 5 (SNORD5), small nucleolar RNA [NR_003033] |
| PDIK1L | NM_001243533 | PDLIM1 interacting kinase 1 like (PDIK1L), transcript variant 3, mRNA [NM_001243533] |
| PRKXP1 | AI016765 | gb|ov27d07.x1 Soares_testis_NHT Homo sapiens cDNA clone IMAGE:1638541 3' similar to SW:PKX1_HUMAN P51817 PROTEIN KINASE PKX1 ;, mRNA sequence [AI016765] |
| C2orf67 | NM_152519 | chromosome 2 open reading frame 67 (C2orf67), mRNA [NM_152519] |
| ENST00000452785 | ENST00000452785 | Unknown |
| TDRD7 | NM_014290 | tudor domain containing 7 (TDRD7), mRNA [NM_014290] |
| NEK1 | NM_012224 | NIMA (never in mitosis gene a)-related kinase 1 (NEK1), transcript variant 2, mRNA [NM_012224] |
| ENST00000453111 | ENST00000453111 | Unknown |
| MID1 | NM_001193278 | midline 1 (Opitz/BBB syndrome) (MID1), transcript variant 6, mRNA [NM_001193278] |
| SLCO1A2 | NM_134431 | solute carrier organic anion transporter family, member 1A2 (SLCO1A2), transcript variant 1, mRNA [NM_134431] |
| VTRNA1-3 | NR_026705 | vault RNA 1-3 (VTRNA1-3), vault RNA [NR_026705] |
| XLOC_013932 | TCONS_00029009 | linc|BROAD Institute lincRNA (XLOC_013932), lincRNA [TCONS_00029009] |
| ZNF622 | NM_033414 | zinc finger protein 622 (ZNF622), mRNA [NM_033414] |
| A_33_P3220994 | A_33_P3220994 | Unknown |
| LOC100271722 | NR_027036 | uncharacterized LOC100271722 (LOC100271722), non-coding RNA [NR_027036] |
| PMEPA1 | NM_020182 | prostate transmembrane protein, androgen induced 1 (PMEPA1), transcript variant 1, mRNA [NM_020182] |
| FAM120A | ENST00000375389 | ens|family with sequence similarity 120A [Source:HGNC Symbol;Acc:13247] [ENST00000375389] |
| MAGOHB | NM_018048 | mago-nashi homolog B (Drosophila) (MAGOHB), mRNA [NM_018048] |
| XLOC_006738 | TCONS_00015252 | linc|BROAD Institute lincRNA (XLOC_006738), lincRNA [TCONS_00015252] |
| XLOC_011577 | TCONS_00023782 | linc|BROAD Institute lincRNA (XLOC_011577), lincRNA [TCONS_00023782] |
| LOC100652866 | XR_132760 | ref|PREDICTED: Homo sapiens hypothetical LOC100652866 (LOC100652866), miscRNA [XR_132760] |
| ZNF597 | NM_152457 | zinc finger protein 597 (ZNF597), mRNA [NM_152457] |
| VPS33A | NM_022916 | vacuolar protein sorting 33 homolog A (S. cerevisiae) (VPS33A), mRNA [NM_022916] |
| S100A9 | NM_002965 | S100 calcium binding protein A9 (S100A9), mRNA [NM_002965] |
| THBS4 | ENST00000513310 | linc|BROAD Institute lincRNA (XLOC_l2_011649), lincRNA [TCONS_l2_00022386] |
| GAPVD1 | BC110990 | gb|Homo sapiens cDNA clone IMAGE:5203707, containing frame-shift errors. [BC110990] |
| XLOC_006008 | TCONS_00013372 | linc|BROAD Institute lincRNA (XLOC_006008), lincRNA [TCONS_00013372] |
| FAM22F | NM_017561 | family with sequence similarity 22, member F (FAM22F), mRNA [NM_017561] |
| LOC100505574 | XR_109297 | ref|PREDICTED: Homo sapiens hypothetical LOC100505574 (LOC100505574), miscRNA [XR_109297] |
| NUDT9P1 | NR_002779 | nudix (nucleoside diphosphate linked moiety X)-type motif 9 pseudogene 1 (NUDT9P1), non-coding RNA [NR_002779] |
| BFAR | NM_016561 | bifunctional apoptosis regulator (BFAR), mRNA [NM_016561] |
| POP1 | NM_015029 | processing of precursor 1, ribonuclease P/MRP subunit (S. cerevisiae) (POP1), transcript variant 3, mRNA [NM_015029] |
| LOC100130880 | NM_001243523 | uncharacterized LOC100130880 (LOC100130880), mRNA [NM_001243523] |
| LOC728093 | XR_133401 | ref|PREDICTED: Homo sapiens putative POM121-like protein 1-like (LOC728093), miscRNA [XR_133401] |
| RPP38 | NM_183005 | ribonuclease P/MRP 38kDa subunit (RPP38), transcript variant 1, mRNA [NM_183005] |
| HIST2H2AB | NM_175065 | histone cluster 2, H2ab (HIST2H2AB), mRNA [NM_175065] |
| LINC00240 | NR_026775 | long intergenic non-protein coding RNA 240 (LINC00240), non-coding RNA [NR_026775] |
| PRDM1 | NM_001198 | PR domain containing 1, with ZNF domain (PRDM1), transcript variant 1, mRNA [NM_001198] |
| XLOC_006273 | ENST00000413567 | gb|K-EST0217412 L18POOL1n1 Homo sapiens cDNA clone L18POOL1n1-8-D04 5', mRNA sequence [CB158131] |
| BC137370 | BC137370 | gb|Homo sapiens chromosome 15 open reading frame 45, mRNA (cDNA clone MGC:168991 IMAGE:9021368), complete cds. [BC137370] |
| XLOC_001134 | ENST00000451690 | linc|BROAD Institute lincRNA (XLOC_001134), lincRNA [TCONS_00001763] |
| LOC100126784 | NR_015384 | uncharacterized LOC100126784 (LOC100126784), non-coding RNA [NR_015384] |
| APC2 | NM_005883 | adenomatosis polyposis coli 2 (APC2), mRNA [NM_005883] |
| ZNF568 | NM_198539 | zinc finger protein 568 (ZNF568), transcript variant 1, mRNA [NM_198539] |
| CLEC6A | NM_001007033 | C-type lectin domain family 6, member A (CLEC6A), mRNA [NM_001007033] |
| OR4C16 | NM_001004701 | olfactory receptor, family 4, subfamily C, member 16 (OR4C16), mRNA [NM_001004701] |
| GIP | NM_004123 | gastric inhibitory polypeptide (GIP), mRNA [NM_004123] |
| FAM159B | NM_001164442 | family with sequence similarity 159, member B (FAM159B), mRNA [NM_001164442] |
| NIF3L1 | NM_001142356 | NIF3 NGG1 interacting factor 3-like 1 (S. pombe) (NIF3L1), transcript variant 4, mRNA [NM_001142356] |
| EIF3D | ENST00000402116 | ens|eukaryotic translation initiation factor 3, subunit D [Source:HGNC Symbol;Acc:3278] [ENST00000402116] |
| XLOC_l2_011924 | ENST00000511721 | linc|BROAD Institute lincRNA (XLOC_l2_011924), lincRNA [TCONS_l2_00022763] |
| NLRP8 | NM_176811 | NLR family, pyrin domain containing 8 (NLRP8), mRNA [NM_176811] |
| LINC00085 | AK024362 | tc|Q8IYS5_HUMAN (Q8IYS5) OSCAR protein (Osteoclast associated receptor OSCAR-S1), partial (17%) [THC2506632] |
| C10orf112 | XM_003403619 | ref|PREDICTED: Homo sapiens chromosome 10 open reading frame 112 (C10orf112), mRNA [XM_003403619] |
| RBM44 | NM_001080504 | RNA binding motif protein 44 (RBM44), mRNA [NM_001080504] |
| OSR2 | NM_053001 | odd-skipped related 2 (Drosophila) (OSR2), transcript variant 2, mRNA [NM_053001] |
| ZFP91 | NM_053023 | zinc finger protein 91 homolog (mouse) (ZFP91), transcript variant 1, mRNA [NM_053023] |
| ZNF143 | NM_003442 | zinc finger protein 143 (ZNF143), mRNA [NM_003442] |
| VPS52 | ENST00000463641 | ens|vacuolar protein sorting 52 homolog (S. cerevisiae) [Source:HGNC Symbol;Acc:10518] [ENST00000463641] |
| XLOC_001306 | TCONS_00004655 | linc|BROAD Institute lincRNA (XLOC_001306), lincRNA [TCONS_00004655] |
| C3orf19 | NM_016474 | chromosome 3 open reading frame 19 (C3orf19), mRNA [NM_016474] |
| LOC100133612 | NR_024455 | uncharacterized LOC100133612 (LOC100133612), non-coding RNA [NR_024455] |
| RNF6 | NM_005977 | ring finger protein (C3H2C3 type) 6 (RNF6), transcript variant 1, mRNA [NM_005977] |
| GZMK | NM_002104 | granzyme K (granzyme 3; tryptase II) (GZMK), mRNA [NM_002104] |
| XLOC_005419 | TCONS_00012566 | linc|BROAD Institute lincRNA (XLOC_005419), lincRNA [TCONS_00012566] |
| CNNM2 | NM_199077 | cyclin M2 (CNNM2), transcript variant 3, mRNA [NM_199077] |
| C12orf39 | ENST00000256969 | ens|chromosome 12 open reading frame 39 [Source:HGNC Symbol;Acc:28139] [ENST00000256969] |
| C17orf78 | NM_173625 | chromosome 17 open reading frame 78 (C17orf78), mRNA [NM_173625] |
| ZNF646 | NM_014699 | zinc finger protein 646 (ZNF646), mRNA [NM_014699] |
| NPPA-AS1 | NR_037806 | NPPA antisense RNA 1 (non-protein coding) (NPPA-AS1), antisense RNA [NR_037806] |
| XLOC_010591 | TCONS_00021991 | linc|BROAD Institute lincRNA (XLOC_010591), lincRNA [TCONS_00021991] |
| XLOC_010739 | TCONS_00022320 | linc|BROAD Institute lincRNA (XLOC_010739), lincRNA [TCONS_00022320] |
| ZNF599 | NM_001007248 | zinc finger protein 599 (ZNF599), mRNA [NM_001007248] |
| XLOC_006187 | AL390145 | linc|BROAD Institute lincRNA (XLOC_006187), lincRNA [TCONS_00013551] |
| ZNF320 | NM_207333 | zinc finger protein 320 (ZNF320), mRNA [NM_207333] |
| FLJ31713 | AK056275 | gb|Homo sapiens cDNA FLJ31713 fis, clone NT2RI2006487. [AK056275] |
| IRX5 | NM_005853 | iroquois homeobox 5 (IRX5), transcript variant 1, mRNA [NM_005853] |
| EXOG | NM_005107 | endo/exonuclease (5'-3'), endonuclease G-like (EXOG), nuclear gene encoding mitochondrial protein, transcript variant 1, mRNA [NM_005107] |
| BRCA2 | NM_000059 | breast cancer 2, early onset (BRCA2), mRNA [NM_000059] |
| MCM4 | NM_005914 | minichromosome maintenance complex component 4 (MCM4), transcript variant 1, mRNA [NM_005914] |
| ARFGEF1 | NM_006421 | ADP-ribosylation factor guanine nucleotide-exchange factor 1 (brefeldin A-inhibited) (ARFGEF1), mRNA [NM_006421] |
| TM2D2 | NM_031940 | TM2 domain containing 2 (TM2D2), transcript variant 2, mRNA [NM_031940] |
| TNKS1BP1 | NM_033396 | tankyrase 1 binding protein 1, 182kDa (TNKS1BP1), mRNA [NM_033396] |
| A_33_P3372104 | A_33_P3372104 | Unknown |
| LOC283888 | NR_037158 | uncharacterized LOC283888 (LOC283888), non-coding RNA [NR_037158] |
| PHF3 | NM_015153 | PHD finger protein 3 (PHF3), mRNA [NM_015153] |
| SNORD82 | NR_004398 | small nucleolar RNA, C/D box 82 (SNORD82), small nuclear RNA [NR_004398] |
| PCDHGB1 | NM_032095 | protocadherin gamma subfamily B, 1 (PCDHGB1), transcript variant 2, mRNA [NM_032095] |
| LOC100291105 | XR_133265 | ref|PREDICTED: Homo sapiens hypothetical LOC100291105 (LOC100291105), miscRNA [XR_133265] |
| ENST00000330493 | ENST00000330493 | ens|Alpha-1,3-mannosyl-glycoprotein 4-beta-N-acetylglucosaminyltransferase-like protein LOC641515 [Source:UniProtKB/Swiss-Prot;Acc:Q49AQ9] [ENST00000330493] |
| A_33_P3233437 | A_33_P3233437 | Unknown |
| C4orf37 | NM_174952 | chromosome 4 open reading frame 37 (C4orf37), mRNA [NM_174952] |
| CLDN3 | NM_001306 | claudin 3 (CLDN3), mRNA [NM_001306] |
| MFSD1 | NM_022736 | major facilitator superfamily domain containing 1 (MFSD1), transcript variant 1, mRNA [NM_022736] |
| XLOC_006426 | ENST00000425077 | linc|BROAD Institute lincRNA (XLOC_006426), lincRNA [TCONS_00013162] |
| SERPINB12 | NM_080474 | serpin peptidase inhibitor, clade B (ovalbumin), member 12 (SERPINB12), mRNA [NM_080474] |
| GDF5 | NM_000557 | growth differentiation factor 5 (GDF5), mRNA [NM_000557] |
| SLC39A10 | NM_020342 | solute carrier family 39 (zinc transporter), member 10 (SLC39A10), transcript variant 2, mRNA [NM_020342] |
| INADL | ENST00000459752 | ens|InaD-like (Drosophila) [Source:HGNC Symbol;Acc:28881] [ENST00000459752] |
| TGFBRAP1 | ENST00000393359 | ens|transforming growth factor, beta receptor associated protein 1 [Source:HGNC Symbol;Acc:16836] [ENST00000393359] |
| A_33_P3268318 | A_33_P3268318 | Unknown |
| HNF1A | NM_000545 | HNF1 homeobox A (HNF1A), mRNA [NM_000545] |
| ZFP2 | NM_030613 | zinc finger protein 2 homolog (mouse) (ZFP2), mRNA [NM_030613] |
| LIMS3L | NR_038099 | LIM and senescent cell antigen-like domains 3-like (LIMS3L), transcript variant 2, non-coding RNA [NR_038099] |
| MAPK8 | NM_139047 | mitogen-activated protein kinase 8 (MAPK8), transcript variant JNK1-b2, mRNA [NM_139047] |
| ZNF222 | NM_013360 | zinc finger protein 222 (ZNF222), transcript variant 2, mRNA [NM_013360] |
| AK130290 | AK130290 | gb|Homo sapiens cDNA FLJ26780 fis, clone PRS03837. [AK130290] |
| TAS2R14 | NM_023922 | taste receptor, type 2, member 14 (TAS2R14), mRNA [NM_023922] |
| LOC100129387 | NR_024490 | uncharacterized LOC100129387 (LOC100129387), non-coding RNA [NR_024490] |
| LHFPL4 | NM_198560 | lipoma HMGIC fusion partner-like 4 (LHFPL4), mRNA [NM_198560] |
| ERO1LB | NM_019891 | ERO1-like beta (S. cerevisiae) (ERO1LB), mRNA [NM_019891] |
| FAM8A1 | NM_016255 | family with sequence similarity 8, member A1 (FAM8A1), mRNA [NM_016255] |
| BUB1B | NM_001211 | budding uninhibited by benzimidazoles 1 homolog beta (yeast) (BUB1B), mRNA [NM_001211] |
| XLOC_013638 | BC039673 | tc|GB|BC039673.1|BC039673.1 Homo sapiens, clone IMAGE:5170855, mRNA [NP1137624] |
| XLOC_l2_001548 | TCONS_l2_00002970 | linc|BROAD Institute lincRNA (XLOC_l2_001548), lincRNA [TCONS_l2_00002970] |
| SOS2 | NM_006939 | son of sevenless homolog 2 (Drosophila) (SOS2), mRNA [NM_006939] |
| TTTY21 | NR_001535 | testis-specific transcript, Y-linked 21 (non-protein coding) (TTTY21), non-coding RNA [NR_001535] |
| SIAH3 | NM_198849 | seven in absentia homolog 3 (Drosophila) (SIAH3), mRNA [NM_198849] |
| NCOA1 | NM_147233 | nuclear receptor coactivator 1 (NCOA1), transcript variant 3, mRNA [NM_147233] |
| CANT1 | NM_138793 | calcium activated nucleotidase 1 (CANT1), transcript variant 1, mRNA [NM_138793] |
| A_33_P3329908 | A_33_P3329908 | Unknown |
| H2AFY | ENST00000512507 | ref|PREDICTED: Homo sapiens hypothetical LOC100506200 (LOC100506200), miscRNA [XR_112759] |
| SNORD9 | NR_003029 | small nucleolar RNA, C/D box 9 (SNORD9), small nucleolar RNA [NR_003029] |
| TEX10 | NM_017746 | testis expressed 10 (TEX10), transcript variant 1, mRNA [NM_017746] |
| LRCH1 | NM_001164213 | leucine-rich repeats and calponin homology (CH) domain containing 1 (LRCH1), transcript variant 3, mRNA [NM_001164213] |
| XLOC_004277 | TCONS_00009863 | linc|BROAD Institute lincRNA (XLOC_004277), lincRNA [TCONS_00009863] |
| POLR2F | ENST00000443002 | ens|polymerase (RNA) II (DNA directed) polypeptide F [Source:HGNC Symbol;Acc:9193] [ENST00000443002] |
| XLOC_006319 | TCONS_00014242 | linc|BROAD Institute lincRNA (XLOC_006319), lincRNA [TCONS_00014242] |
| XLOC_010878 | TCONS_00022557 | linc|BROAD Institute lincRNA (XLOC_010878), lincRNA [TCONS_00022557] |
| DTL | NM_016448 | denticleless homolog (Drosophila) (DTL), mRNA [NM_016448] |
| PHYHD1 | NM_174933 | phytanoyl-CoA dioxygenase domain containing 1 (PHYHD1), transcript variant 2, mRNA [NM_174933] |
| SCFD2 | NM_152540 | sec1 family domain containing 2 (SCFD2), mRNA [NM_152540] |
| THRB | NM_001128177 | thyroid hormone receptor, beta (erythroblastic leukemia viral (v-erb-a) oncogene homolog 2, avian) (THRB), transcript variant 3, mRNA [NM_001128177] |
| XLOC_010496 | ENST00000411690 | linc|BROAD Institute lincRNA (XLOC_010496), lincRNA [TCONS_00021598] |
| ENST00000553990 | ENST00000553990 | gb|EST11357 human nasopharynx Homo sapiens cDNA, mRNA sequence [CD694834] |
| KIF11 | NM_004523 | kinesin family member 11 (KIF11), mRNA [NM_004523] |
| SART3 | NM_014706 | squamous cell carcinoma antigen recognized by T cells 3 (SART3), mRNA [NM_014706] |
| XLOC_009254 | TCONS_00019977 | linc|BROAD Institute lincRNA (XLOC_009254), lincRNA [TCONS_00019977] |
| XLOC_003551 | ENST00000509782 | gb|BX115989 Soares_testis_NHT Homo sapiens cDNA clone IMAGp998E203521, mRNA sequence [BX115989] |
| GSTT2B | NM_001080843 | glutathione S-transferase theta 2B (gene/pseudogene) (GSTT2B), mRNA [NM_001080843] |
| ZNF613 | NM_024840 | zinc finger protein 613 (ZNF613), transcript variant 2, mRNA [NM_024840] |
| ANKRD49 | NM_017704 | ankyrin repeat domain 49 (ANKRD49), mRNA [NM_017704] |
| OXSM | NM_017897 | 3-oxoacyl-ACP synthase, mitochondrial (OXSM), nuclear gene encoding mitochondrial protein, transcript variant 1, mRNA [NM_017897] |
| LOC100505492 | XR_108511 | ref|PREDICTED: Homo sapiens hypothetical LOC100505492 (LOC100505492), miscRNA [XR_108511] |
| XLOC_004290 | BX096515 | gb|BX096515 Soares_testis_NHT Homo sapiens cDNA clone IMAGp998A123517, mRNA sequence [BX096515] |
| TXNDC15 | NM_024715 | thioredoxin domain containing 15 (TXNDC15), mRNA [NM_024715] |
| LPAL2 | NR_028093 | lipoprotein, Lp(a)-like 2, pseudogene (LPAL2), transcript variant 2, non-coding RNA [NR_028093] |
| ZNF19 | NM_006961 | zinc finger protein 19 (ZNF19), mRNA [NM_006961] |
| MAGEA11 | NM_001011544 | melanoma antigen family A, 11 (MAGEA11), transcript variant 2, mRNA [NM_001011544] |
| A_33_P3389558 | A_33_P3389558 | Unknown |
| DSPP | NM_014208 | dentin sialophosphoprotein (DSPP), mRNA [NM_014208] |
| UGT2A1 | NM_001252274 | UDP glucuronosyltransferase 2 family, polypeptide A1, complex locus (UGT2A1), transcript variant 2, mRNA [NM_001252274] |
| EVI5 | NM_005665 | ecotropic viral integration site 5 (EVI5), mRNA [NM_005665] |
| LOC100506622 | XR_110105 | ref|PREDICTED: Homo sapiens hypothetical LOC100506622 (LOC100506622), miscRNA [XR_110105] |
| KSR1 | NM_014238 | kinase suppressor of ras 1 (KSR1), mRNA [NM_014238] |
| PANX1 | NM_015368 | pannexin 1 (PANX1), mRNA [NM_015368] |
| XLOC_l2_011145 | ENST00000503987 | linc|BROAD Institute lincRNA (XLOC_l2_011145), lincRNA [TCONS_l2_00021896] |
| XLOC_l2_013282 | TCONS_l2_00025671 | linc|BROAD Institute lincRNA (XLOC_l2_013282), lincRNA [TCONS_l2_00025671] |
| RCVRN | NM_002903 | recoverin (RCVRN), mRNA [NM_002903] |
| XLOC_007800 | TCONS_00016843 | linc|BROAD Institute lincRNA (XLOC_007800), lincRNA [TCONS_00016843] |
| THC2713260 | THC2713260 | Unknown |
| A_33_P3224285 | A_33_P3224285 | Unknown |
| HIST1H2AM | NM_003514 | histone cluster 1, H2am (HIST1H2AM), mRNA [NM_003514] |
| XLOC_l2_009811 | TCONS_l2_00018729 | linc|BROAD Institute lincRNA (XLOC_l2_009811), lincRNA [TCONS_l2_00018729] |
| XLOC_011281 | TCONS_00023960 | linc|BROAD Institute lincRNA (XLOC_011281), lincRNA [TCONS_00023960] |
| FOXO3 | NM_001455 | forkhead box O3 (FOXO3), transcript variant 1, mRNA [NM_001455] |
| LBP | NM_004139 | lipopolysaccharide binding protein (LBP), mRNA [NM_004139] |
| MKX | NM_173576 | mohawk homeobox (MKX), transcript variant 1, mRNA [NM_173576] |
| SLC22A2 | ENST00000366952 | ens|solute carrier family 22 (organic cation transporter), member 2 [Source:HGNC Symbol;Acc:10966] [ENST00000366952] |
| CXCL12 | NM_199168 | chemokine (C-X-C motif) ligand 12 (CXCL12), transcript variant 1, mRNA [NM_199168] |
| XLOC_008791 | ENST00000419406 | linc|BROAD Institute lincRNA (XLOC_008791), lincRNA [TCONS_00017960] |
| BEST4 | NM_153274 | bestrophin 4 (BEST4), mRNA [NM_153274] |
| LOC100506343 | NR_038951 | uncharacterized LOC100506343 (LOC100506343), transcript variant 1, non-coding RNA [NR_038951] |
| ASTL | NM_001002036 | astacin-like metallo-endopeptidase (M12 family) (ASTL), mRNA [NM_001002036] |
| DNAAF2 | NM_018139 | dynein, axonemal, assembly factor 2 (DNAAF2), transcript variant 1, mRNA [NM_018139] |
| RPL23AP7 | NR_024530 | ribosomal protein L23a pseudogene 7 (RPL23AP7), transcript variant 4, non-coding RNA [NR_024530] |
| H2BFXP | NR_003238 | H2B histone family, member X, pseudogene (H2BFXP), non-coding RNA [NR_003238] |
| XLOC_l2_007832 | ENST00000433036 | linc|BROAD Institute lincRNA (XLOC_l2_007832), lincRNA [TCONS_l2_00014331] |
| ZNF778 | NM_001201407 | zinc finger protein 778 (ZNF778), transcript variant 1, mRNA [NM_001201407] |
| AF461897 | AF461897 | gb|Homo sapiens FP15331 mRNA, complete cds. [AF461897] |
| ANKIB1 | NM_019004 | ankyrin repeat and IBR domain containing 1 (ANKIB1), mRNA [NM_019004] |
| NCOA3 | NM_181659 | nuclear receptor coactivator 3 (NCOA3), transcript variant 1, mRNA [NM_181659] |
| LOC100131667 | AK124758 | gb|Homo sapiens cDNA FLJ42768 fis, clone BRAWH3003522. [AK124758] |
| GPRC5B | NM_016235 | G protein-coupled receptor, family C, group 5, member B (GPRC5B), mRNA [NM_016235] |
| LSMD1 | NM_032356 | LSM domain containing 1 (LSMD1), mRNA [NM_032356] |
| XLOC_006252 | TCONS_00013597 | linc|BROAD Institute lincRNA (XLOC_006252), lincRNA [TCONS_00013597] |
| GDF2 | NM_016204 | growth differentiation factor 2 (GDF2), mRNA [NM_016204] |
| XLOC_008256 | TCONS_00017386 | linc|BROAD Institute lincRNA (XLOC_008256), lincRNA [TCONS_00017386] |
| NGDN | NM_001042635 | neuroguidin, EIF4E binding protein (NGDN), transcript variant 1, mRNA [NM_001042635] |
| TIGD1 | NM_145702 | tigger transposable element derived 1 (TIGD1), mRNA [NM_145702] |
| TMEM41A | ENST00000467061 | ens|transmembrane protein 41A [Source:HGNC Symbol;Acc:30544] [ENST00000467061] |
| SNORA14A | NR_002955 | small nucleolar RNA, H/ACA box 14A (SNORA14A), small nucleolar RNA [NR_002955] |
| AB385295 | AB385295 | gb|Synthetic construct DNA, clone: pF1KA0197, Homo sapiens NUP160 gene for nucleoporin 160kDa, complete cds, without stop codon, in Flexi system. [AB385295] |
| HEPACAM2 | NM_001039372 | HEPACAM family member 2 (HEPACAM2), transcript variant 1, mRNA [NM_001039372] |
| XLOC_004302 | ENST00000513041 | gb|AGENCOURT_40977101 NIH_MGC_278 Homo sapiens cDNA clone IMAGE:7773405 3', mRNA sequence [CX759005] |
| ENST00000477589 | ENST00000477589 | ref|PREDICTED: Homo sapiens cytokine receptor CRL2 (LOC100287290), mRNA [XM_002342405] |
| C1orf105 | NM_139240 | chromosome 1 open reading frame 105 (C1orf105), mRNA [NM_139240] |
| EDN3 | NM_207032 | endothelin 3 (EDN3), transcript variant 2, mRNA [NM_207032] |
| LRRC18 | NM_001006939 | leucine rich repeat containing 18 (LRRC18), mRNA [NM_001006939] |
| A_33_P3270354 | A_33_P3270354 | Unknown |
| HIST2H2AC | NM_003517 | histone cluster 2, H2ac (HIST2H2AC), mRNA [NM_003517] |
| NOP16 | NM_016391 | NOP16 nucleolar protein homolog (yeast) (NOP16), mRNA [NM_016391] |
| KCNMA1 | NM_002247 | potassium large conductance calcium-activated channel, subfamily M, alpha member 1 (KCNMA1), transcript variant 2, mRNA [NM_002247] |
| XLOC_003346 | TCONS_00006718 | linc|BROAD Institute lincRNA (XLOC_003346), lincRNA [TCONS_00006718] |
| MCM6 | NM_005915 | minichromosome maintenance complex component 6 (MCM6), mRNA [NM_005915] |
| XLOC_000822 | ENST00000420876 | tc|Q3BCG3_9ARAC (Q3BCG3) Tubuliform spidroin (Fragment), partial (5%) [THC2721785] |
| CTAGE4 | NM_198495 | CTAGE family, member 4 (CTAGE4), mRNA [NM_198495] |
| FAM82A1 | ENST00000402091 | ens|family with sequence similarity 82, member A1 [Source:HGNC Symbol;Acc:26567] [ENST00000402091] |
| IPO13 | A_21_P0014041 | Unknown |
| SNORA8 | NR_002920 | small nucleolar RNA, H/ACA box 8 (SNORA8), small nucleolar RNA [NR_002920] |
| ABL2 | NM_007314 | v-abl Abelson murine leukemia viral oncogene homolog 2 (ABL2), transcript variant b, mRNA [NM_007314] |
| UIMC1 | NM_016290 | ubiquitin interaction motif containing 1 (UIMC1), transcript variant 2, mRNA [NM_016290] |
| PP12719 | XM_003403527 | ref|PREDICTED: Homo sapiens hypothetical protein LOC100653022 (LOC100653022), mRNA [XM_003403527] |
| GDAP2 | NM_017686 | ganglioside induced differentiation associated protein 2 (GDAP2), transcript variant 1, mRNA [NM_017686] |
| TMEM200C | NM_001080209 | transmembrane protein 200C (TMEM200C), mRNA [NM_001080209] |
| XLOC_009249 | TCONS_00019972 | linc|BROAD Institute lincRNA (XLOC_009249), lincRNA [TCONS_00019972] |
| XLOC_007167 | TCONS_00015110 | linc|BROAD Institute lincRNA (XLOC_007167), lincRNA [TCONS_00015110] |
| ZDHHC21 | NM_178566 | zinc finger, DHHC-type containing 21 (ZDHHC21), mRNA [NM_178566] |
| XLOC_012676 | TCONS_00026329 | linc|BROAD Institute lincRNA (XLOC_012676), lincRNA [TCONS_00026329] |
| SMURF2 | NM_022739 | SMAD specific E3 ubiquitin protein ligase 2 (SMURF2), mRNA [NM_022739] |
| STXBP5L | NM_014980 | syntaxin binding protein 5-like (STXBP5L), mRNA [NM_014980] |
| HIST1H3H | NM_003536 | histone cluster 1, H3h (HIST1H3H), mRNA [NM_003536] |
| MTR | NM_000254 | 5-methyltetrahydrofolate-homocysteine methyltransferase (MTR), mRNA [NM_000254] |
| THC2598321 | THC2598321 | Unknown |
| XLOC_004577 | THC2506711 | linc|BROAD Institute lincRNA (XLOC_004577), lincRNA [TCONS_00009260] |
| XLOC_004257 | TCONS_00009845 | linc|BROAD Institute lincRNA (XLOC_004257), lincRNA [TCONS_00009845] |
| RMI1 | NM_024945 | RMI1, RecQ mediated genome instability 1, homolog (S. cerevisiae) (RMI1), mRNA [NM_024945] |
| XLOC_l2_014832 | ENST00000417703 | linc|BROAD Institute lincRNA (XLOC_l2_014832), lincRNA [TCONS_l2_00028814] |
| A_24_P358131 | A_24_P358131 | Unknown |
| XLOC_009582 | TCONS_00019784 | linc|BROAD Institute lincRNA (XLOC_009582), lincRNA [TCONS_00019784] |
| SMAD9 | NM_005905 | SMAD family member 9 (SMAD9), transcript variant b, mRNA [NM_005905] |
| XLOC_013218 | BX105847 | gb|BX105847 Soares_NFL_T_GBC_S1 Homo sapiens cDNA clone IMAGp998K123696, mRNA sequence [BX105847] |
| GRRP1 | NM_024869 | glycine/arginine rich protein 1 (GRRP1), mRNA [NM_024869] |
| SERINC1 | NM_020755 | serine incorporator 1 (SERINC1), mRNA [NM_020755] |
| MCF2L | ENST00000442625 | ref|PREDICTED: Homo sapiens hypothetical LOC100506063 (LOC100506063), miscRNA [XR_111408] |
| XLOC_008117 | ENST00000391359 | linc|BROAD Institute lincRNA (XLOC_008117), lincRNA [TCONS_00017042] |
| XLOC_008826 | TCONS_00018937 | linc|BROAD Institute lincRNA (XLOC_008826), lincRNA [TCONS_00018937] |
| ZNF132 | NM_003433 | zinc finger protein 132 (ZNF132), mRNA [NM_003433] |
| SERPINA11 | NM_001080451 | serpin peptidase inhibitor, clade A (alpha-1 antiproteinase, antitrypsin), member 11 (SERPINA11), mRNA [NM_001080451] |
| XLOC_l2_001583 | ENST00000427229 | linc|BROAD Institute lincRNA (XLOC_l2_001583), lincRNA [TCONS_l2_00003966] |
| XLOC_007508 | TCONS_00016123 | linc|BROAD Institute lincRNA (XLOC_007508), lincRNA [TCONS_00016123] |
| XLOC_001418 | TCONS_00003626 | linc|BROAD Institute lincRNA (XLOC_001418), lincRNA [TCONS_00003626] |
| DNAJC9 | NM_015190 | DnaJ (Hsp40) homolog, subfamily C, member 9 (DNAJC9), mRNA [NM_015190] |
| RGN | NM_152869 | regucalcin (senescence marker protein-30) (RGN), transcript variant 2, mRNA [NM_152869] |
| TBC1D30 | NM_015279 | TBC1 domain family, member 30 (TBC1D30), mRNA [NM_015279] |
| AKIRIN2 | NM_018064 | akirin 2 (AKIRIN2), mRNA [NM_018064] |
| A_33_P3324687 | A_33_P3324687 | Unknown |
| FAM86HP | AK125025 | gb|tt33d11.x1 NCI_CGAP_GC6 Homo sapiens cDNA clone IMAGE:2242581 3', mRNA sequence [AI638717] |
| GPATCH3 | NM_022078 | G patch domain containing 3 (GPATCH3), mRNA [NM_022078] |
| IL34 | NM_152456 | interleukin 34 (IL34), transcript variant 1, mRNA [NM_152456] |
| KIF2B | NM_032559 | kinesin family member 2B (KIF2B), mRNA [NM_032559] |
| AB051446 | AB051446 | gb|Homo sapiens mRNA for KIAA1659 protein, partial cds. [AB051446] |
| LOC100271832 | NR_027097 | uncharacterized LOC100271832 (LOC100271832), non-coding RNA [NR_027097] |
| FLJ27352 | NM_001198784 | uncharacterized LOC145788 (FLJ27352), mRNA [NM_001198784] |
| HIST1H1E | NM_005321 | histone cluster 1, H1e (HIST1H1E), mRNA [NM_005321] |
| LOC283143 | NR_034148 | uncharacterized LOC283143 (LOC283143), non-coding RNA [NR_034148] |
| RHBDL2 | NM_017821 | rhomboid, veinlet-like 2 (Drosophila) (RHBDL2), mRNA [NM_017821] |
| A_33_P3323989 | A_33_P3323989 | Unknown |
| XLOC_006993 | TCONS_00015403 | linc|BROAD Institute lincRNA (XLOC_006993), lincRNA [TCONS_00015403] |
| CD274 | NM_014143 | CD274 molecule (CD274), mRNA [NM_014143] |
| FAM178A | ENST00000370271 | ens|family with sequence similarity 178, member A [Source:HGNC Symbol;Acc:17814] [ENST00000370271] |
| LOC100128591 | AK128705 | linc|BROAD Institute lincRNA (XLOC_013766), lincRNA [TCONS_00028412] |
| HIST1H2AB | NM_003513 | histone cluster 1, H2ab (HIST1H2AB), mRNA [NM_003513] |
| XLOC_013301 | THC2622230 | tc|Q72GU7_THET2 (Q72GU7) Transporter, partial (6%) [THC2707376] |
| LOC100652850 | XR_132554 | ref|PREDICTED: Homo sapiens hypothetical LOC100652850 (LOC100652850), miscRNA [XR_132554] |
| ADRA2C | NM_000683 | adrenergic, alpha-2C-, receptor (ADRA2C), mRNA [NM_000683] |
| HNRNPH1 | ENST00000522256 | tc|HUM49KDA hnRNP H {Homo sapiens} (exp=-1; wgp=0; cg=0), partial (42%) [THC2530892] |
| C12orf36 | NR_036555 | chromosome 12 open reading frame 36 (C12orf36), non-coding RNA [NR_036555] |
| LOC100130776 | NR_027032 | uncharacterized LOC100130776 (LOC100130776), non-coding RNA [NR_027032] |
| APOA1 | NM_000039 | apolipoprotein A-I (APOA1), mRNA [NM_000039] |
| DNAJA2 | NM_005880 | DnaJ (Hsp40) homolog, subfamily A, member 2 (DNAJA2), mRNA [NM_005880] |
| LOC340113 | NR_033832 | uncharacterized LOC340113 (LOC340113), non-coding RNA [NR_033832] |
| CDC5L | NM_001253 | CDC5 cell division cycle 5-like (S. pombe) (CDC5L), mRNA [NM_001253] |
| RABEP1 | NM_004703 | rabaptin, RAB GTPase binding effector protein 1 (RABEP1), transcript variant 1, mRNA [NM_004703] |
| TXNRD3NB | NM_001039783 | thioredoxin reductase 3 neighbor (TXNRD3NB), mRNA [NM_001039783] |
| KLK8 | NM_144505 | kallikrein-related peptidase 8 (KLK8), transcript variant 2, mRNA [NM_144505] |
| ZNF582 | NM_144690 | zinc finger protein 582 (ZNF582), mRNA [NM_144690] |
| SLC7A14 | NM_020949 | solute carrier family 7 (orphan transporter), member 14 (SLC7A14), mRNA [NM_020949] |
| XLOC_002987 | TCONS_00006371 | linc|BROAD Institute lincRNA (XLOC_002987), lincRNA [TCONS_00006371] |
| DACT2 | NM_214462 | dapper, antagonist of beta-catenin, homolog 2 (Xenopus laevis) (DACT2), mRNA [NM_214462] |
| GAGE7 | NM_021123 | G antigen 7 (GAGE7), mRNA [NM_021123] |
| XLOC_007801 | TCONS_00016844 | linc|BROAD Institute lincRNA (XLOC_007801), lincRNA [TCONS_00016844] |
| XLOC_011821 | TCONS_00024502 | linc|BROAD Institute lincRNA (XLOC_011821), lincRNA [TCONS_00024502] |
| NEIL3 | NM_018248 | nei endonuclease VIII-like 3 (E. coli) (NEIL3), mRNA [NM_018248] |
| UNC79 | NM_020818 | unc-79 homolog (C. elegans) (UNC79), mRNA [NM_020818] |
| NFU1 | BX538347 | gb|Homo sapiens mRNA; cDNA DKFZp686H1983 (from clone DKFZp686H1983). [BX538347] |
| WHAMMP3 | NR_003521 | WAS protein homolog associated with actin, golgi membranes and microtubules pseudogene 3 (WHAMMP3), non-coding RNA [NR_003521] |
| CDYL2 | NM_152342 | chromodomain protein, Y-like 2 (CDYL2), mRNA [NM_152342] |
| AY129018 | AY129018 | gb|Homo sapiens clone FP18376 unknown mRNA. [AY129018] |
| PCDH7 | NM_002589 | protocadherin 7 (PCDH7), transcript variant a, mRNA [NM_002589] |
| VKORC1L1 | NM_173517 | vitamin K epoxide reductase complex, subunit 1-like 1 (VKORC1L1), mRNA [NM_173517] |
| MKRN1 | NM_013446 | makorin ring finger protein 1 (MKRN1), transcript variant 1, mRNA [NM_013446] |
| XLOC_008498 | TCONS_00018219 | linc|BROAD Institute lincRNA (XLOC_008498), lincRNA [TCONS_00018219] |
| GRPEL2 | NM_152407 | GrpE-like 2, mitochondrial (E. coli) (GRPEL2), nuclear gene encoding mitochondrial protein, mRNA [NM_152407] |
| XLOC_005166 | TCONS_00011691 | linc|BROAD Institute lincRNA (XLOC_005166), lincRNA [TCONS_00011691] |
| FLJ42351 | AK124342 | gb|Homo sapiens cDNA FLJ42351 fis, clone UTERU2005664. [AK124342] |
| BF106382 | BF106382 | gb|601823216F1 NIH_MGC_77 Homo sapiens cDNA clone IMAGE:4043354 5', mRNA sequence [BF106382] |
| LOC642236 | CR933606 | linc|BROAD Institute lincRNA (XLOC_l2_015213), lincRNA [TCONS_l2_00029355] |
| ATP5E | ENST00000395663 | ens|ATP synthase, H+ transporting, mitochondrial F1 complex, epsilon subunit [Source:HGNC Symbol;Acc:838] [ENST00000395663] |
| C15orf37 | NR_028330 | chromosome 15 open reading frame 37 (C15orf37), non-coding RNA [NR_028330] |
| LOC100132832 | NR_028058 | PMS2 postmeiotic segregation increased 2 (S. cerevisiae) pseudogene (LOC100132832), non-coding RNA [NR_028058] |
| ZNF425 | NM_001001661 | zinc finger protein 425 (ZNF425), mRNA [NM_001001661] |
| PRRG2 | NM_000951 | proline rich Gla (G-carboxyglutamic acid) 2 (PRRG2), mRNA [NM_000951] |
| KCTD1 | NM_198991 | potassium channel tetramerisation domain containing 1 (KCTD1), transcript variant 2, mRNA [NM_198991] |
| MAZ | NM_001042539 | MYC-associated zinc finger protein (purine-binding transcription factor) (MAZ), transcript variant 2, mRNA [NM_001042539] |
| ELL3 | NM_025165 | elongation factor RNA polymerase II-like 3 (ELL3), mRNA [NM_025165] |
| FOXI1 | NM_012188 | forkhead box I1 (FOXI1), transcript variant 1, mRNA [NM_012188] |
| KLK6 | NM_001012964 | kallikrein-related peptidase 6 (KLK6), transcript variant B, mRNA [NM_001012964] |
| LOC100270679 | NR_038394 | uncharacterized LOC100270679 (LOC100270679), non-coding RNA [NR_038394] |
| RHAG | NM_000324 | Rh-associated glycoprotein (RHAG), mRNA [NM_000324] |
| ARHGAP25 | NM_001007231 | Rho GTPase activating protein 25 (ARHGAP25), transcript variant 1, mRNA [NM_001007231] |
| XLOC_006322 | TCONS_00013661 | linc|BROAD Institute lincRNA (XLOC_006322), lincRNA [TCONS_00013661] |
| SLC5A6 | NM_021095 | solute carrier family 5 (sodium-dependent vitamin transporter), member 6 (SLC5A6), transcript variant 1, mRNA [NM_021095] |
| CHCHD3 | NM_017812 | coiled-coil-helix-coiled-coil-helix domain containing 3 (CHCHD3), mRNA [NM_017812] |
| SSH2 | ENST00000324677 | ens|slingshot homolog 2 (Drosophila) [Source:HGNC Symbol;Acc:30580] [ENST00000324677] |
| LOC100506023 | NR_037845 | uncharacterized LOC100506023 (LOC100506023), non-coding RNA [NR_037845] |
| XLOC_l2_001255 | TCONS_l2_00001700 | linc|BROAD Institute lincRNA (XLOC_l2_001255), lincRNA [TCONS_l2_00001700] |
| AUTS2 | NM_001127232 | autism susceptibility candidate 2 (AUTS2), transcript variant 3, mRNA [NM_001127232] |
| VCP | NM_007126 | valosin containing protein (VCP), mRNA [NM_007126] |
| BPIFB6 | NM_174897 | BPI fold containing family B, member 6 (BPIFB6), mRNA [NM_174897] |
| AZI2 | NM_001134433 | 5-azacytidine induced 2 (AZI2), transcript variant 3, mRNA [NM_001134433] |
| TPR | NM_003292 | translocated promoter region (to activated MET oncogene) (TPR), mRNA [NM_003292] |
| SERPINB5 | NM_002639 | serpin peptidase inhibitor, clade B (ovalbumin), member 5 (SERPINB5), mRNA [NM_002639] |
| XLOC_003572 | ENST00000504368 | gb|BX095211 Soares fetal liver spleen 1NFLS Homo sapiens cDNA clone IMAGp998P22654, mRNA sequence [BX095211] |
| SNHG4 | NR_003141 | small nucleolar RNA host gene 4 (non-protein coding) (SNHG4), transcript variant 1, non-coding RNA [NR_003141] |
| LOC145474 | NR_027046 | uncharacterized LOC145474 (LOC145474), non-coding RNA [NR_027046] |
| LOC100507217 | NR_037601 | uncharacterized LOC100507217 (LOC100507217), transcript variant 1, non-coding RNA [NR_037601] |
| A_33_P3214404 | A_33_P3214404 | Unknown |
| CARD10 | NM_014550 | caspase recruitment domain family, member 10 (CARD10), mRNA [NM_014550] |
| XLOC_002403 | ENST00000440016 | linc|BROAD Institute lincRNA (XLOC_002403), lincRNA [TCONS_00003443] |
| A_33_P3294730 | A_33_P3294730 | Unknown |
| PPP1R12B | NM_002481 | protein phosphatase 1, regulatory subunit 12B (PPP1R12B), transcript variant 1, mRNA [NM_002481] |
| AF116649 | AF116649 | gb|Homo sapiens PRO0566 mRNA, complete cds. [AF116649] |
| XLOC_005780 | TCONS_00012221 | linc|BROAD Institute lincRNA (XLOC_005780), lincRNA [TCONS_00012221] |
| ADAMTSL3 | NM_207517 | ADAMTS-like 3 (ADAMTSL3), mRNA [NM_207517] |
| ZBTB11 | NM_014415 | zinc finger and BTB domain containing 11 (ZBTB11), mRNA [NM_014415] |
| VSTM4 | NM_001031746 | V-set and transmembrane domain containing 4 (VSTM4), transcript variant 1, mRNA [NM_001031746] |
| XLOC_l2_015590 | BC028039 | gb|603183339F1 NIH_MGC_121 Homo sapiens cDNA clone IMAGE:5247537 5', mRNA sequence [BI913911] |
| TAF7 | NM_005642 | TAF7 RNA polymerase II, TATA box binding protein (TBP)-associated factor, 55kDa (TAF7), mRNA [NM_005642] |
| XLOC_005575 | TCONS_00012640 | linc|BROAD Institute lincRNA (XLOC_005575), lincRNA [TCONS_00012640] |
| XLOC_011050 | TCONS_00022768 | linc|BROAD Institute lincRNA (XLOC_011050), lincRNA [TCONS_00022768] |
| TSIX | NR_003255 | TSIX transcript, XIST antisense RNA (non-protein coding) (TSIX), antisense RNA [NR_003255] |
| LOC93622 | NR_015433 | Morf4 family associated protein 1-like 1 pseudogene (LOC93622), non-coding RNA [NR_015433] |
| CCR1 | NM_001295 | chemokine (C-C motif) receptor 1 (CCR1), mRNA [NM_001295] |
| XLOC_012879 | TCONS_00026735 | linc|BROAD Institute lincRNA (XLOC_012879), lincRNA [TCONS_00026735] |
| KIAA2026 | NM_001017969 | KIAA2026 (KIAA2026), mRNA [NM_001017969] |
| XLOC_010838 | TCONS_00022514 | linc|BROAD Institute lincRNA (XLOC_010838), lincRNA [TCONS_00022514] |
| B4GALT5 | NM_004776 | UDP-Gal:betaGlcNAc beta 1,4- galactosyltransferase, polypeptide 5 (B4GALT5), mRNA [NM_004776] |
| APPL2 | NM_018171 | adaptor protein, phosphotyrosine interaction, PH domain and leucine zipper containing 2 (APPL2), transcript variant 1, mRNA [NM_018171] |
| XLOC_006439 | TCONS_00013799 | linc|BROAD Institute lincRNA (XLOC_006439), lincRNA [TCONS_00013799] |
| XLOC_l2_007884 | ENST00000454224 | Unknown |
| SPDYE2 | NM_001031618 | speedy homolog E2 (Xenopus laevis) (SPDYE2), mRNA [NM_001031618] |
| XLOC_004726 | BC030121 | tc|Q21AG7_RHOPA (Q21AG7) Na+-transporting methylmalonyl-CoA/oxaloacetate decarboxylase, beta subunit, partial (5%) [THC2604654] |
| FGD4 | NM_139241 | FYVE, RhoGEF and PH domain containing 4 (FGD4), mRNA [NM_139241] |
| THC2584414 | THC2584414 | tc|ALU2_HUMAN (P39189) Alu subfamily SB sequence contamination warning entry, partial (25%) [THC2584414] |
| DKFZP586B0319 | AL050097 | gb|Homo sapiens mRNA; cDNA DKFZp586B0319 (from clone DKFZp586B0319) [AL050097] |
| COL20A1 | NM_020882 | collagen, type XX, alpha 1 (COL20A1), mRNA [NM_020882] |
| ATF3 | NM_001040619 | activating transcription factor 3 (ATF3), transcript variant 4, mRNA [NM_001040619] |
| CXCR4 | NM_001008540 | chemokine (C-X-C motif) receptor 4 (CXCR4), transcript variant 1, mRNA [NM_001008540] |
| ZNF470 | NM_001001668 | zinc finger protein 470 (ZNF470), mRNA [NM_001001668] |
| ZNF587 | NM_032828 | zinc finger protein 587 (ZNF587), transcript variant 1, mRNA [NM_032828] |
| XLOC_007499 | TCONS_00016111 | linc|BROAD Institute lincRNA (XLOC_007499), lincRNA [TCONS_00016111] |
| TUSC1 | NM_001004125 | tumor suppressor candidate 1 (TUSC1), mRNA [NM_001004125] |
| TXNDC9 | NM_005783 | thioredoxin domain containing 9 (TXNDC9), mRNA [NM_005783] |
| C4orf22 | NM_152770 | chromosome 4 open reading frame 22 (C4orf22), transcript variant 2, mRNA [NM_152770] |
| XLOC_l2_007644 | ENST00000416361 | tc|Q9BXE6_HUMAN (Q9BXE6) FKSG49, partial (93%) [THC2501073] |
| KLHL1 | NM_020866 | kelch-like 1 (Drosophila) (KLHL1), mRNA [NM_020866] |
| XLOC_000190 | THC2698970 | linc|BROAD Institute lincRNA (XLOC_000190), lincRNA [TCONS_00000938] |
| CD209 | NM_021155 | CD209 molecule (CD209), transcript variant 1, mRNA [NM_021155] |
| ARNT2 | NM_014862 | aryl-hydrocarbon receptor nuclear translocator 2 (ARNT2), mRNA [NM_014862] |
| MTHFD2L | NM_001144978 | methylenetetrahydrofolate dehydrogenase (NADP+ dependent) 2-like (MTHFD2L), mRNA [NM_001144978] |
| XLOC_l2_015213 | TCONS_l2_00029348 | linc|BROAD Institute lincRNA (XLOC_l2_015213), lincRNA [TCONS_l2_00029348] |
| XLOC_000095 | TCONS_00000867 | linc|BROAD Institute lincRNA (XLOC_000095), lincRNA [TCONS_00000867] |
| ZZZ3 | NM_015534 | zinc finger, ZZ-type containing 3 (ZZZ3), mRNA [NM_015534] |
| ENST00000390379 | ENST00000390379 | ens|T cell receptor beta variable 6-9 [Source:HGNC Symbol;Acc:12234] [ENST00000390379] |
| A_33_P3413514 | A_33_P3413514 | Unknown |
| XLOC_005459 | ENST00000444229 | linc|BROAD Institute lincRNA (XLOC_005459), lincRNA [TCONS_00011368] |
| YJEFN3 | NM_198537 | YjeF N-terminal domain containing 3 (YJEFN3), nuclear gene encoding mitochondrial protein, transcript variant 1, mRNA [NM_198537] |
| SOST | NM_025237 | sclerostin (SOST), mRNA [NM_025237] |
| GRID2IP | NM_001145118 | glutamate receptor, ionotropic, delta 2 (Grid2) interacting protein (GRID2IP), mRNA [NM_001145118] |
| XLOC_012429 | TCONS_00025601 | linc|BROAD Institute lincRNA (XLOC_012429), lincRNA [TCONS_00025601] |
| VN1R5 | NM_173858 | vomeronasal 1 receptor 5 (gene/pseudogene) (VN1R5), mRNA [NM_173858] |
| REEP6 | NM_138393 | receptor accessory protein 6 (REEP6), mRNA [NM_138393] |
| PRTG | NM_173814 | protogenin (PRTG), mRNA [NM_173814] |
| XLOC_l2_005793 | THC2699069 | tc|Q7SG75_NEUCR (Q7SG75) Predicted protein, partial (3%) [THC2699069] |
| ZNF254 | NM_203282 | zinc finger protein 254 (ZNF254), mRNA [NM_203282] |
| LOC100506516 | XR_108763 | ref|PREDICTED: Homo sapiens hypothetical LOC100506516 (LOC100506516), miscRNA [XR_108763] |
| A_33_P3253717 | A_33_P3253717 | Unknown |
| IL9 | NM_000590 | interleukin 9 (IL9), mRNA [NM_000590] |
| XLOC_000745 | ENST00000429666 | gb|AV715291 DCB Homo sapiens cDNA clone DCBCDE09 5', mRNA sequence [AV715291] |
| DAZL | NM_001351 | deleted in azoospermia-like (DAZL), transcript variant 2, mRNA [NM_001351] |
| SH3BP2 | NM_001145855 | SH3-domain binding protein 2 (SH3BP2), transcript variant 4, mRNA [NM_001145855] |
| BICC1 | NM_001080512 | bicaudal C homolog 1 (Drosophila) (BICC1), mRNA [NM_001080512] |
| GLYATL1 | NM_001220494 | glycine-N-acyltransferase-like 1 (GLYATL1), transcript variant 2, mRNA [NM_001220494] |
| LINC00474 | NR_024032 | long intergenic non-protein coding RNA 474 (LINC00474), non-coding RNA [NR_024032] |
| XLOC_008528 | AL353951 | gb|Homo sapiens mRNA; cDNA DKFZp761A0423 (from clone DKFZp761A0423) [AL353951] |
| MRPL18 | NM_014161 | mitochondrial ribosomal protein L18 (MRPL18), nuclear gene encoding mitochondrial protein, mRNA [NM_014161] |
| LOC100652824 | ENST00000409515 | ens|Uncharacterized protein [Source:UniProtKB/TrEMBL;Acc:E7EP55] [ENST00000409515] |
| NUP35 | NM_138285 | nucleoporin 35kDa (NUP35), mRNA [NM_138285] |
| PHF17 | NM_199320 | PHD finger protein 17 (PHF17), transcript variant L, mRNA [NM_199320] |
| CLEC18C | NM_173619 | C-type lectin domain family 18, member C (CLEC18C), mRNA [NM_173619] |
| XLOC_000428 | TCONS_00001175 | linc|BROAD Institute lincRNA (XLOC_000428), lincRNA [TCONS_00001175] |
| ENST00000376617 | ENST00000376617 | gb|Homo sapiens cDNA FLJ41123 fis, clone BRACE2014657. [AK123118] |
| FLCN | ENST00000389168 | ens|folliculin [Source:HGNC Symbol;Acc:27310] [ENST00000389168] |
| MR1 | NM_001531 | major histocompatibility complex, class I-related (MR1), transcript variant 1, mRNA [NM_001531] |
| XLOC_001446 | AK024234 | tc|ALU1_HUMAN (P39188) Alu subfamily J sequence contamination warning entry, partial (15%) [THC2634653] |
| MBD5 | NM_018328 | methyl-CpG binding domain protein 5 (MBD5), mRNA [NM_018328] |
| MBOAT4 | NM_001100916 | membrane bound O-acyltransferase domain containing 4 (MBOAT4), mRNA [NM_001100916] |
| XLOC_009926 | TCONS_00021241 | linc|BROAD Institute lincRNA (XLOC_009926), lincRNA [TCONS_00021241] |
| PRDM14 | NM_024504 | PR domain containing 14 (PRDM14), mRNA [NM_024504] |
| GGT8P | NR_003503 | gamma-glutamyltransferase 8 pseudogene (GGT8P), non-coding RNA [NR_003503] |
| SNORD30 | NR_002561 | small nucleolar RNA, C/D box 30 (SNORD30), small nucleolar RNA [NR_002561] |
| A_33_P3326802 | A_33_P3326802 | Unknown |
| XLOC_010184 | TCONS_00020905 | linc|BROAD Institute lincRNA (XLOC_010184), lincRNA [TCONS_00020905] |
| MIPEPP3 | BX648740 | gb|Homo sapiens mRNA; cDNA DKFZp686C1238 (from clone DKFZp686C1238). [BX648740] |
| SLC4A1 | NM_000342 | solute carrier family 4, anion exchanger, member 1 (erythrocyte membrane protein band 3, Diego blood group) (SLC4A1), mRNA [NM_000342] |
| XLOC_013448 | THC2758825 | linc|BROAD Institute lincRNA (XLOC_013448), lincRNA [TCONS_00028097] |
| TPM3 | NM_152263 | tropomyosin 3 (TPM3), transcript variant 1, mRNA [NM_152263] |
| PEX2 | NM_001172086 | peroxisomal biogenesis factor 2 (PEX2), transcript variant 3, mRNA [NM_001172086] |
| XLOC_l2_013873 | ENST00000412091 | linc|BROAD Institute lincRNA (XLOC_l2_013873), lincRNA [TCONS_l2_00026656] |
| XLOC_003839 | THC2771827 | linc|BROAD Institute lincRNA (XLOC_003839), lincRNA [TCONS_00008387] |
| ZCCHC8 | NM_017612 | zinc finger, CCHC domain containing 8 (ZCCHC8), mRNA [NM_017612] |
| C3orf58 | NM_173552 | chromosome 3 open reading frame 58 (C3orf58), transcript variant 1, mRNA [NM_173552] |
| HLX | NM_021958 | H2.0-like homeobox (HLX), mRNA [NM_021958] |
| ENST00000431031 | ENST00000431031 | ens|spermatogenesis associated 1 [Source:HGNC Symbol;Acc:14682] [ENST00000431031] |
| EWSR1 | NM_013986 | Ewing sarcoma breakpoint region 1 (EWSR1), transcript variant 1, mRNA [NM_013986] |
| THC2647746 | THC2647746 | tc|O52K2_HUMAN (Q8NGK3) Olfactory receptor 52K2, partial (29%) [THC2647746] |
| MUC2 | NM_002457 | mucin 2, oligomeric mucus/gel-forming (MUC2), mRNA [NM_002457] |
| SNORA22 | AL706006 | gb|DKFZp686E1139_r1 686 (synonym: hlcc3) Homo sapiens cDNA clone DKFZp686E1139 5', mRNA sequence [AL706006] |
| HIST1H3F | NM_021018 | histone cluster 1, H3f (HIST1H3F), mRNA [NM_021018] |
| CSF2RA | NM_172249 | colony stimulating factor 2 receptor, alpha, low-affinity (granulocyte-macrophage) (CSF2RA), transcript variant 6, mRNA [NM_172249] |
| BLMH | NM_000386 | bleomycin hydrolase (BLMH), mRNA [NM_000386] |
| XLOC_013037 | THC2615013 | tc|AY558543 YKL031W {Saccharomyces cerevisiae} (exp=-1; wgp=0; cg=0), partial (17%) [THC2615013] |
| XLOC_l2_013741 | TCONS_l2_00026420 | linc|BROAD Institute lincRNA (XLOC_l2_013741), lincRNA [TCONS_l2_00026420] |
| SLC38A9 | NM_173514 | solute carrier family 38, member 9 (SLC38A9), mRNA [NM_173514] |
| XLOC_004501 | TCONS_00009477 | linc|BROAD Institute lincRNA (XLOC_004501), lincRNA [TCONS_00009477] |
| XLOC_010643 | ENST00000451570 | tc|Q86UH8_HUMAN (Q86UH8) Pol protein, partial (3%) [THC2749540] |
| ATP5L2 | BG113928 | gb|602284393F1 NIH_MGC_86 Homo sapiens cDNA clone IMAGE:4371934 5', mRNA sequence [BG113928] |
| A_33_P3397298 | A_33_P3397298 | Unknown |
| RDH12 | NM_152443 | retinol dehydrogenase 12 (all-trans/9-cis/11-cis) (RDH12), mRNA [NM_152443] |
| PRKRIP1 | NM_024653 | PRKR interacting protein 1 (IL11 inducible) (PRKRIP1), mRNA [NM_024653] |
| FASLG | NM_000639 | Fas ligand (TNF superfamily, member 6) (FASLG), mRNA [NM_000639] |
| ZNF624 | NM_020787 | zinc finger protein 624 (ZNF624), mRNA [NM_020787] |
| ADAMTS19 | NM_133638 | ADAM metallopeptidase with thrombospondin type 1 motif, 19 (ADAMTS19), mRNA [NM_133638] |
| ZNF618 | NM_133374 | zinc finger protein 618 (ZNF618), mRNA [NM_133374] |
| XLOC_012751 | TCONS_00026401 | linc|BROAD Institute lincRNA (XLOC_012751), lincRNA [TCONS_00026401] |
| VCX2 | NM_016378 | variable charge, X-linked 2 (VCX2), mRNA [NM_016378] |
| EIF2S2 | NM_003908 | eukaryotic translation initiation factor 2, subunit 2 beta, 38kDa (EIF2S2), mRNA [NM_003908] |
| SVOP | NM_018711 | SV2 related protein homolog (rat) (SVOP), mRNA [NM_018711] |
| SNORA2B | NR_002951 | small nucleolar RNA, H/ACA box 2B (SNORA2B), small nucleolar RNA [NR_002951] |
| LOC285419 | AL833449 | gb|Homo sapiens mRNA; cDNA DKFZp686P12109 (from clone DKFZp686P12109). [AL833449] |
| PCDHB1 | NM_013340 | protocadherin beta 1 (PCDHB1), mRNA [NM_013340] |
| SEC13 | ENST00000397099 | ens|SEC13 homolog (S. cerevisiae) [Source:HGNC Symbol;Acc:10697] [ENST00000397099] |
| WT1 | NM_024426 | Wilms tumor 1 (WT1), transcript variant D, mRNA [NM_024426] |
| XLOC_006721 | ENST00000510244 | linc|BROAD Institute lincRNA (XLOC_006721), lincRNA [TCONS_00015231] |
| XLOC_007487 | ENST00000450399 | linc|BROAD Institute lincRNA (XLOC_007487), lincRNA [TCONS_00015723] |
| IFNG | NM_000619 | interferon, gamma (IFNG), mRNA [NM_000619] |
| RG9MTD1 | NM_017819 | RNA (guanine-9-) methyltransferase domain containing 1 (RG9MTD1), nuclear gene encoding mitochondrial protein, mRNA [NM_017819] |
| SFTPB | NM_000542 | surfactant protein B (SFTPB), transcript variant 1, mRNA [NM_000542] |
| LOC440944 | NR_027007 | uncharacterized LOC440944 (LOC440944), non-coding RNA [NR_027007] |
| FBXO36 | NM_174899 | F-box protein 36 (FBXO36), mRNA [NM_174899] |
| LOC100216546 | NR_039981 | uncharacterized LOC100216546 (LOC100216546), non-coding RNA [NR_039981] |
| LOC100507372 | XR_109516 | ref|PREDICTED: Homo sapiens hypothetical LOC100507372, transcript variant 1 (LOC100507372), miscRNA [XR_109516] |
| CYLC2 | NM_001340 | cylicin, basic protein of sperm head cytoskeleton 2 (CYLC2), mRNA [NM_001340] |
| XLOC_007850 | TCONS_00016868 | linc|BROAD Institute lincRNA (XLOC_007850), lincRNA [TCONS_00016868] |
| LOC100505902 | XR_108533 | ref|PREDICTED: Homo sapiens hypothetical LOC100505902 (LOC100505902), miscRNA [XR_108533] |
| LCN8 | ENST00000479767 | ens|lipocalin 8 [Source:HGNC Symbol;Acc:27038] [ENST00000479767] |
| PCDHB5 | NM_015669 | protocadherin beta 5 (PCDHB5), mRNA [NM_015669] |
| XLOC_l2_013931 | ENST00000464543 | tc|CG028_HUMAN (O95766) Protein C7orf28, partial (20%) [THC2543586] |
| SCN3B | NM_018400 | sodium channel, voltage-gated, type III, beta (SCN3B), transcript variant 1, mRNA [NM_018400] |
| XLOC_005160 | TCONS_00011687 | linc|BROAD Institute lincRNA (XLOC_005160), lincRNA [TCONS_00011687] |
| TRIT1 | NM_017646 | tRNA isopentenyltransferase 1 (TRIT1), mRNA [NM_017646] |
| LOC441666 | NR_024380 | zinc finger protein 91 pseudogene (LOC441666), non-coding RNA [NR_024380] |
| ZNF165 | NM_003447 | zinc finger protein 165 (ZNF165), mRNA [NM_003447] |
| HNRNPH3 | NM_012207 | heterogeneous nuclear ribonucleoprotein H3 (2H9) (HNRNPH3), transcript variant 2H9, mRNA [NM_012207] |
| LOC100505515 | XR_109648 | ref|PREDICTED: Homo sapiens hypothetical LOC100505515 (LOC100505515), miscRNA [XR_109648] |
| ABHD10 | NM_018394 | abhydrolase domain containing 10 (ABHD10), mRNA [NM_018394] |
| C1orf210 | NM_182517 | chromosome 1 open reading frame 210 (C1orf210), transcript variant 1, mRNA [NM_182517] |
| SNORD83B | NR_000028 | small nucleolar RNA, C/D box 83B (SNORD83B), small nucleolar RNA [NR_000028] |
| C5orf30 | NM_033211 | chromosome 5 open reading frame 30 (C5orf30), mRNA [NM_033211] |
| XLOC_006043 | ENST00000458087 | ref|PREDICTED: Homo sapiens hypothetical LOC100506725, transcript variant 2 (LOC100506725), miscRNA [XR_108768] |
| XLOC_011980 | TCONS_00024702 | linc|BROAD Institute lincRNA (XLOC_011980), lincRNA [TCONS_00024702] |
| USP16 | NM_001032410 | ubiquitin specific peptidase 16 (USP16), transcript variant 3, mRNA [NM_001032410] |
| XLOC_006259 | TCONS_00014218 | linc|BROAD Institute lincRNA (XLOC_006259), lincRNA [TCONS_00014218] |
| FLJ30403 | NR_034159 | uncharacterized LOC729975 (FLJ30403), non-coding RNA [NR_034159] |
| SYNRG | NM_007247 | synergin, gamma (SYNRG), transcript variant 1, mRNA [NM_007247] |
| CLOCK | NM_004898 | clock homolog (mouse) (CLOCK), mRNA [NM_004898] |
| FAM98B | ENST00000491535 | ens|family with sequence similarity 98, member B [Source:HGNC Symbol;Acc:26773] [ENST00000491535] |
| TXNDC8 | ENST00000374511 | ens|thioredoxin domain containing 8 (spermatozoa) [Source:HGNC Symbol;Acc:31454] [ENST00000374511] |
| CYLD | NM_015247 | cylindromatosis (turban tumor syndrome) (CYLD), transcript variant 1, mRNA [NM_015247] |
| PROK2 | NM_021935 | prokineticin 2 (PROK2), transcript variant 2, mRNA [NM_021935] |
| RPS6KA1 | NM_002953 | ribosomal protein S6 kinase, 90kDa, polypeptide 1 (RPS6KA1), transcript variant 1, mRNA [NM_002953] |
| FECH | NM_001012515 | ferrochelatase (FECH), nuclear gene encoding mitochondrial protein, transcript variant 1, mRNA [NM_001012515] |
| PEX3 | NM_003630 | peroxisomal biogenesis factor 3 (PEX3), mRNA [NM_003630] |
| FAM206A | NM_017832 | family with sequence similarity 206, member A (FAM206A), mRNA [NM_017832] |
| RTP2 | NM_001004312 | receptor (chemosensory) transporter protein 2 (RTP2), mRNA [NM_001004312] |
| THC2619931 | THC2619931 | tc|HUMGLIA1F glial factor-1 {Homo sapiens} (exp=-1; wgp=0; cg=0), partial (5%) [THC2619931] |
| LOC100130156 | XM_003118800 | ref|PREDICTED: Homo sapiens hypothetical protein LOC100130156 (LOC100130156), mRNA [XM_003118800] |
| ENST00000531371 | ENST00000531371 | gb|Homo sapiens hypothetical LOC643733, mRNA (cDNA clone IMAGE:40030527), containing frame-shift errors. [BC122531] |
| A_33_P3350196 | A_33_P3350196 | Unknown |
| HIST1H2BA | NM_170610 | histone cluster 1, H2ba (HIST1H2BA), mRNA [NM_170610] |
| SCNN1D | NM_001130413 | sodium channel, nonvoltage-gated 1, delta (SCNN1D), transcript variant 1, mRNA [NM_001130413] |
| ALLC | NM_018436 | allantoicase (ALLC), transcript variant 1, mRNA [NM_018436] |
| THC2631504 | THC2631504 | tc|Q4KHJ1_PSEF5 (Q4KHJ1) Glycerol-3-phosphate acyltransferase, partial (3%) [THC2631504] |
| XLOC_009748 | BC034605 | tc|Q9IKB9_9ALPH (Q9IKB9) Glycoprotein E, partial (5%) [THC2606854] |
| NCKAP5L | ENST00000491441 | ens|NCK-associated protein 5-like [Source:HGNC Symbol;Acc:29321] [ENST00000491441] |
| TAS2R7 | NM_023919 | taste receptor, type 2, member 7 (TAS2R7), mRNA [NM_023919] |
| LGSN | NM_016571 | lengsin, lens protein with glutamine synthetase domain (LGSN), transcript variant 1, mRNA [NM_016571] |
| XLOC_001738 | ENST00000425688 | gb|603047960F1 NIH_MGC_116 Homo sapiens cDNA clone IMAGE:5188262 5', mRNA sequence [BI762903] |
| LINC00486 | NR_027098 | long intergenic non-protein coding RNA 486 (LINC00486), transcript variant 1, non-coding RNA [NR_027098] |
| OR4C13 | NM_001001955 | olfactory receptor, family 4, subfamily C, member 13 (OR4C13), mRNA [NM_001001955] |
| DKFZP564C196 | NR_040063 | uncharacterized LOC284649 (DKFZP564C196), non-coding RNA [NR_040063] |
| DA399191 | DA399191 | gb|DA399191 BRTHA3 Homo sapiens cDNA clone BRTHA3001578 5', mRNA sequence [DA399191] |
| HSD17B12 | ENST00000533358 | linc|BROAD Institute lincRNA (XLOC_009116), lincRNA [TCONS_00019283] |
| A_33_P3232637 | A_33_P3232637 | Unknown |
| BG208718 | BG208718 | gb|RST28352 Athersys RAGE Library Homo sapiens cDNA, mRNA sequence [BG208718] |
| TMEM133 | NM_032021 | transmembrane protein 133 (TMEM133), mRNA [NM_032021] |
| XLOC_004254 | TCONS_00009838 | linc|BROAD Institute lincRNA (XLOC_004254), lincRNA [TCONS_00009838] |
| XLOC_012628 | TCONS_00026283 | linc|BROAD Institute lincRNA (XLOC_012628), lincRNA [TCONS_00026283] |
| TBX2 | NM_005994 | T-box 2 (TBX2), mRNA [NM_005994] |
| SNORD112 | NR_003080 | small nucleolar RNA, C/D box 112 (SNORD112), small nucleolar RNA [NR_003080] |
| SSX8 | NR_027250 | synovial sarcoma, X breakpoint 8 (SSX8), non-coding RNA [NR_027250] |
| XLOC_004135 | BI519951 | gb|603071734F1 NIH_MGC_119 Homo sapiens cDNA clone IMAGE:5163609 5', mRNA sequence [BI519951] |
| XLOC_003511 | TCONS_00008047 | linc|BROAD Institute lincRNA (XLOC_003511), lincRNA [TCONS_00008047] |
| XLOC_008054 | TCONS_00017234 | linc|BROAD Institute lincRNA (XLOC_008054), lincRNA [TCONS_00017234] |
| C12orf63 | ENST00000298953 | Unknown |
| TGM7 | NM_052955 | transglutaminase 7 (TGM7), mRNA [NM_052955] |
| TTK | NM_003318 | TTK protein kinase (TTK), transcript variant 1, mRNA [NM_003318] |
| FAM200A | NM_145111 | family with sequence similarity 200, member A (FAM200A), mRNA [NM_145111] |
| XLOC_001006 | TCONS_00001668 | linc|BROAD Institute lincRNA (XLOC_001006), lincRNA [TCONS_00001668] |
| RBM43 | NM_198557 | RNA binding motif protein 43 (RBM43), mRNA [NM_198557] |
| IGSF1 | NM_205833 | immunoglobulin superfamily, member 1 (IGSF1), transcript variant 2, mRNA [NM_205833] |
| EIF4E2 | AK303802 | gb|Homo sapiens cDNA FLJ59146 complete cds, moderately similar to Eukaryotic translation initiation factor 4E type 3. [AK303802] |
| XLOC_007309 | CR613587 | gb|full-length cDNA clone CS0DC011YE23 of Neuroblastoma Cot 25-normalized of Homo sapiens (human) [CR613587] |
| ENST00000478672 | ENST00000478672 | ens|CCZ1 vacuolar protein trafficking and biogenesis associated homolog (S. cerevisiae) [Source:HGNC Symbol;Acc:21691] [ENST00000478672] |
| TCEANC | ENST00000490617 | ens|transcription elongation factor A (SII) N-terminal and central domain containing [Source:HGNC Symbol;Acc:28277] [ENST00000490617] |
| ST6GALNAC3 | NM_001160011 | ST6 (alpha-N-acetyl-neuraminyl-2,3-beta-galactosyl-1,3)-N-acetylgalactosaminide alpha-2,6-sialyltransferase 3 (ST6GALNAC3), transcript variant 2, mRNA [NM_001160011] |
| BSN | NM_003458 | bassoon (presynaptic cytomatrix protein) (BSN), mRNA [NM_003458] |
| OR5T3 | NM_001004747 | olfactory receptor, family 5, subfamily T, member 3 (OR5T3), mRNA [NM_001004747] |
| CACNA1C | NM_199460 | calcium channel, voltage-dependent, L type, alpha 1C subunit (CACNA1C), transcript variant 1, mRNA [NM_199460] |
| C14orf86 | NR_027004 | chromosome 14 open reading frame 86 (C14orf86), non-coding RNA [NR_027004] |
| ZNF300 | NM_052860 | zinc finger protein 300 (ZNF300), transcript variant 2, mRNA [NM_052860] |
| IMPA1 | NM_005536 | inositol(myo)-1(or 4)-monophosphatase 1 (IMPA1), transcript variant 1, mRNA [NM_005536] |
| LOC100131792 | AK093561 | gb|Homo sapiens cDNA FLJ36242 fis, clone THYMU2001727. [AK093561] |
| XLOC_002612 | ENST00000415991 | linc|BROAD Institute lincRNA (XLOC_002612), lincRNA [TCONS_00005511] |
| XLOC_003762 | ENST00000512652 | linc|BROAD Institute lincRNA (XLOC_003762), lincRNA [TCONS_00007671] |
| C16orf72 | BC029878 | gb|Homo sapiens PRO0149 protein, mRNA (cDNA clone IMAGE:5172419), containing frame-shift errors. [BC029878] |
| C1QTNF3 | NM_181435 | C1q and tumor necrosis factor related protein 3 (C1QTNF3), transcript variant 2, mRNA [NM_181435] |
| ENST00000342892 | ENST00000342892 | gb|Homo sapiens cDNA FLJ44380 fis, clone TRACH3035482. [AK126351] |
| FLJ37448 | AK094767 | linc|BROAD Institute lincRNA (XLOC_012549), lincRNA [TCONS_00025728] |
| XLOC_002419 | ENST00000449835 | linc|BROAD Institute lincRNA (XLOC_002419), lincRNA [TCONS_00003462] |
| DA751309 | DA751309 | gb|DA751309 NT2RP7 Homo sapiens cDNA clone NT2RP7017155 5', mRNA sequence [DA751309] |
| XLOC_002673 | TCONS_00006032 | linc|BROAD Institute lincRNA (XLOC_002673), lincRNA [TCONS_00006032] |
| LILRB4 | NM_006847 | leukocyte immunoglobulin-like receptor, subfamily B (with TM and ITIM domains), member 4 (LILRB4), transcript variant 1, mRNA [NM_006847] |
| BBS5 | NM_152384 | Bardet-Biedl syndrome 5 (BBS5), mRNA [NM_152384] |
| PRPF40A | NM_017892 | PRP40 pre-mRNA processing factor 40 homolog A (S. cerevisiae) (PRPF40A), mRNA [NM_017892] |
| WDR66 | NM_144668 | WD repeat domain 66 (WDR66), transcript variant 1, mRNA [NM_144668] |
| NCAPH | NM_015341 | non-SMC condensin I complex, subunit H (NCAPH), mRNA [NM_015341] |
| ZBTB6 | NM_006626 | zinc finger and BTB domain containing 6 (ZBTB6), mRNA [NM_006626] |
| PPAP2A | NM_176895 | phosphatidic acid phosphatase type 2A (PPAP2A), transcript variant 2, mRNA [NM_176895] |
| FAAH2 | NM_174912 | fatty acid amide hydrolase 2 (FAAH2), mRNA [NM_174912] |
| WDFY3 | ENST00000426414 | ens|WD repeat and FYVE domain containing 3 [Source:HGNC Symbol;Acc:20751] [ENST00000426414] |
| SAP30 | NM_003864 | Sin3A-associated protein, 30kDa (SAP30), mRNA [NM_003864] |
| TSTD2 | NM_139246 | thiosulfate sulfurtransferase (rhodanese)-like domain containing 2 (TSTD2), mRNA [NM_139246] |
| HIST1H1D | NM_005320 | histone cluster 1, H1d (HIST1H1D), mRNA [NM_005320] |
| C2orf83 | NM_020161 | chromosome 2 open reading frame 83 (C2orf83), transcript variant 1, mRNA [NM_020161] |
| C9orf79 | NM_178828 | chromosome 9 open reading frame 79 (C9orf79), mRNA [NM_178828] |
| EID2B | NM_152361 | EP300 interacting inhibitor of differentiation 2B (EID2B), mRNA [NM_152361] |
| C17orf57 | NM_001195192 | chromosome 17 open reading frame 57 (C17orf57), transcript variant B, mRNA [NM_001195192] |
| EPHB4 | NM_004444 | EPH receptor B4 (EPHB4), mRNA [NM_004444] |
| RPL31 | ENST00000409000 | ens|ribosomal protein L31 [Source:HGNC Symbol;Acc:10334] [ENST00000409000] |
| AK025118 | AK025118 | gb|Homo sapiens cDNA: FLJ21465 fis, clone COL04784. [AK025118] |
| FAM174A | NM_198507 | family with sequence similarity 174, member A (FAM174A), mRNA [NM_198507] |
| LOC100130372 | AK127532 | gb|Homo sapiens cDNA FLJ45625 fis, clone BRTHA3028505. [AK127532] |
| IRF8 | NM_002163 | interferon regulatory factor 8 (IRF8), mRNA [NM_002163] |
| SCARNA7 | NR_003001 | small Cajal body-specific RNA 7 (SCARNA7), guide RNA [NR_003001] |
| XLOC_009599 | TCONS_00019800 | linc|BROAD Institute lincRNA (XLOC_009599), lincRNA [TCONS_00019800] |
| ORAI3 | NM_152288 | ORAI calcium release-activated calcium modulator 3 (ORAI3), mRNA [NM_152288] |
| CXorf68 | NM_001162936 | chromosome X open reading frame 68 (CXorf68), mRNA [NM_001162936] |
| SPAG9 | NM_003971 | sperm associated antigen 9 (SPAG9), transcript variant 3, mRNA [NM_003971] |
| XLOC_014412 | AK128164 | linc|BROAD Institute lincRNA (XLOC_014412), lincRNA [TCONS_00029494] |
| GJD2 | NM_020660 | gap junction protein, delta 2, 36kDa (GJD2), mRNA [NM_020660] |
| XLOC_004653 | TCONS_00010842 | linc|BROAD Institute lincRNA (XLOC_004653), lincRNA [TCONS_00010842] |
| TMEM35 | NM_021637 | transmembrane protein 35 (TMEM35), mRNA [NM_021637] |
| MAGEF1 | NM_022149 | melanoma antigen family F, 1 (MAGEF1), mRNA [NM_022149] |
| TMEM87B | NM_032824 | transmembrane protein 87B (TMEM87B), mRNA [NM_032824] |
| CNTNAP4 | NM_138994 | contactin associated protein-like 4 (CNTNAP4), transcript variant 2, mRNA [NM_138994] |
| FAM160A1 | NM_001109977 | family with sequence similarity 160, member A1 (FAM160A1), mRNA [NM_001109977] |
| IPCEF1 | NM_001130699 | interaction protein for cytohesin exchange factors 1 (IPCEF1), transcript variant 1, mRNA [NM_001130699] |
| XLOC_004860 | AK128486 | tc|ALU1_HUMAN (P39188) Alu subfamily J sequence contamination warning entry, partial (12%) [THC2634258] |
| XLOC_006228 | ENST00000497598 | linc|BROAD Institute lincRNA (XLOC_006228), lincRNA [TCONS_00013071] |
| SFTPC | NM_003018 | surfactant protein C (SFTPC), transcript variant 1, mRNA [NM_003018] |
| RYR3 | NM_001036 | ryanodine receptor 3 (RYR3), transcript variant 1, mRNA [NM_001036] |
| TSHZ1 | NM_005786 | teashirt zinc finger homeobox 1 (TSHZ1), mRNA [NM_005786] |
| LOC100507351 | NR_040050 | uncharacterized LOC100507351 (LOC100507351), non-coding RNA [NR_040050] |
| XLOC_l2_015673 | THC2666070 | linc|BROAD Institute lincRNA (XLOC_l2_015673), lincRNA [TCONS_l2_00030438] |
| CNTFR | NM_147164 | ciliary neurotrophic factor receptor (CNTFR), transcript variant 1, mRNA [NM_147164] |
| NEK11 | NM_024800 | NIMA (never in mitosis gene a)- related kinase 11 (NEK11), transcript variant 1, mRNA [NM_024800] |
| ENST00000534728 | ENST00000534728 | gb|Homo sapiens cDNA FLJ46155 fis, clone TESTI4001517. [AK128036] |
| C15orf33 | NM_152647 | chromosome 15 open reading frame 33 (C15orf33), mRNA [NM_152647] |
| XLOC_l2_010751 | ENST00000506864 | linc|BROAD Institute lincRNA (XLOC_l2_010751), lincRNA [TCONS_l2_00020644] |
| ZSCAN5D | XM_001725568 | ref|PREDICTED: Homo sapiens zinc finger and SCAN domain containing 5D (ZSCAN5D), mRNA [XM_001725568] |
| SYNPO2L | ENST00000394810 | ens|synaptopodin 2-like [Source:HGNC Symbol;Acc:23532] [ENST00000394810] |
| XLOC_001650 | TCONS_00003845 | linc|BROAD Institute lincRNA (XLOC_001650), lincRNA [TCONS_00003845] |
| ENST00000447310 | ENST00000447310 | ens|zinc finger protein 550 [Source:HGNC Symbol;Acc:28643] [ENST00000447310] |
| SCD5 | NM_024906 | stearoyl-CoA desaturase 5 (SCD5), transcript variant 2, mRNA [NM_024906] |
| P2RY12 | NM_022788 | purinergic receptor P2Y, G-protein coupled, 12 (P2RY12), transcript variant 1, mRNA [NM_022788] |
| WNT11 | NM_004626 | wingless-type MMTV integration site family, member 11 (WNT11), mRNA [NM_004626] |
| CSNK1G3 | NM_001044723 | casein kinase 1, gamma 3 (CSNK1G3), transcript variant 4, mRNA [NM_001044723] |
| LOC550112 | NR_015439 | uncharacterized LOC550112 (LOC550112), non-coding RNA [NR_015439] |
| XLOC_003452 | ENST00000505494 | gb|602544662F1 NIH_MGC_60 Homo sapiens cDNA clone IMAGE:4667270 5', mRNA sequence [BG499001] |
| AMPD1 | NM_000036 | adenosine monophosphate deaminase 1 (AMPD1), transcript variant 1, mRNA [NM_000036] |
| XLOC_001001 | ENST00000452883 | Unknown |
| THEM4 | NM_053055 | thioesterase superfamily member 4 (THEM4), mRNA [NM_053055] |
| LOC100505533 | XR_109157 | ref|PREDICTED: Homo sapiens hypothetical LOC100505533 (LOC100505533), miscRNA [XR_109157] |
| XLOC_003539 | ENST00000511634 | linc|BROAD Institute lincRNA (XLOC_003539), lincRNA [TCONS_00007530] |
| A_33_P3250974 | A_33_P3250974 | Unknown |
| DDX60L | NM_001012967 | DEAD (Asp-Glu-Ala-Asp) box polypeptide 60-like (DDX60L), mRNA [NM_001012967] |
| EPB41L5 | NM_001184938 | erythrocyte membrane protein band 4.1 like 5 (EPB41L5), transcript variant 3, mRNA [NM_001184938] |
| LOC100130097 | XM_001717074 | ref|PREDICTED: Homo sapiens kinesin-like protein family member 6-like (LOC100130097), mRNA [XM_001717074] |
| LOC100507295 | ENST00000554859 | ref|PREDICTED: Homo sapiens hypothetical LOC100507295 (LOC100507295), miscRNA [XR_110253] |
| PPIL6 | NM_173672 | peptidylprolyl isomerase (cyclophilin)-like 6 (PPIL6), transcript variant 1, mRNA [NM_173672] |
| C9orf25 | NM_001184941 | chromosome 9 open reading frame 25 (C9orf25), transcript variant 2, mRNA [NM_001184941] |
| MZT2A | ENST00000491265 | ens|mitotic spindle organizing protein 2A [Source:HGNC Symbol;Acc:33187] [ENST00000491265] |
| MYO1D | NM_015194 | myosin ID (MYO1D), mRNA [NM_015194] |
| XLOC_002071 | TCONS_00004231 | linc|BROAD Institute lincRNA (XLOC_002071), lincRNA [TCONS_00004231] |
| XLOC_006829 | TCONS_00014745 | linc|BROAD Institute lincRNA (XLOC_006829), lincRNA [TCONS_00014745] |
| XLOC_012016 | TCONS_00024735 | linc|BROAD Institute lincRNA (XLOC_012016), lincRNA [TCONS_00024735] |
| CCDC81 | NM_021827 | coiled-coil domain containing 81 (CCDC81), transcript variant 2, mRNA [NM_021827] |
| A_33_P3358109 | A_33_P3358109 | Unknown |
| LCA5L | NM_152505 | Leber congenital amaurosis 5-like (LCA5L), mRNA [NM_152505] |
| XPO6 | NM_015171 | exportin 6 (XPO6), mRNA [NM_015171] |
| MIRLET7BHG | NR_027033 | MIRLET7B host gene (non-protein coding) (MIRLET7BHG), non-coding RNA [NR_027033] |
| SSR1 | NM_003144 | signal sequence receptor, alpha (SSR1), mRNA [NM_003144] |
| AGGF1 | NM_018046 | angiogenic factor with G patch and FHA domains 1 (AGGF1), mRNA [NM_018046] |
| LOC220729 | NR_003266 | succinate dehydrogenase complex, subunit A, flavoprotein (Fp) pseudogene (LOC220729), non-coding RNA [NR_003266] |
| SSC5D | NM_001144950 | scavenger receptor cysteine rich domain containing (5 domains) (SSC5D), transcript variant 1, mRNA [NM_001144950] |
| XLOC_001748 | THC2711871 | linc|BROAD Institute lincRNA (XLOC_001748), lincRNA [TCONS_00003932] |
| XLOC_004495 | TCONS_00010050 | linc|BROAD Institute lincRNA (XLOC_004495), lincRNA [TCONS_00010050] |
| SNORD4B | NR_000009 | small nucleolar RNA, C/D box 4B (SNORD4B), small nucleolar RNA [NR_000009] |
| FEM1A | NM_018708 | fem-1 homolog a (C. elegans) (FEM1A), mRNA [NM_018708] |
| C11orf58 | NM_014267 | chromosome 11 open reading frame 58 (C11orf58), mRNA [NM_014267] |
| XLOC_007052 | TCONS_00014970 | linc|BROAD Institute lincRNA (XLOC_007052), lincRNA [TCONS_00014970] |
| A_33_P3234088 | A_33_P3234088 | Unknown |
| OR13F1 | NM_001004485 | olfactory receptor, family 13, subfamily F, member 1 (OR13F1), mRNA [NM_001004485] |
| A_33_P3282175 | A_33_P3282175 | Unknown |
| LOC100507639 | XR_109837 | ref|PREDICTED: Homo sapiens hypothetical LOC100507639 (LOC100507639), miscRNA [XR_109837] |
| KDM2B | ENST00000261824 | ens|lysine (K)-specific demethylase 2B [Source:HGNC Symbol;Acc:13610] [ENST00000261824] |
| A_33_P3212316 | A_33_P3212316 | Unknown |
| XLOC_l2_009539 | TCONS_l2_00018032 | linc|BROAD Institute lincRNA (XLOC_l2_009539), lincRNA [TCONS_l2_00018032] |
| LOC100507064 | NR_038288 | uncharacterized LOC100507064 (LOC100507064), transcript variant 2, non-coding RNA [NR_038288] |
| KIF15 | NM_020242 | kinesin family member 15 (KIF15), mRNA [NM_020242] |
| XLOC_010972 | TCONS_00022677 | linc|BROAD Institute lincRNA (XLOC_010972), lincRNA [TCONS_00022677] |
| CYP11B1 | NM_000497 | cytochrome P450, family 11, subfamily B, polypeptide 1 (CYP11B1), nuclear gene encoding mitochondrial protein, transcript variant 1, mRNA [NM_000497] |
| THC2658062 | THC2658062 | Unknown |
| XLOC_l2_007184 | ENST00000441014 | linc|BROAD Institute lincRNA (XLOC_l2_007184), lincRNA [TCONS_l2_00015229] |
| XLOC_004635 | THC2723318 | tc|Q330J9_HUMAN (Q330J9) Cyclin M (Fragment), partial (29%) [THC2723318] |
| LOC100130015 | NR_027336 | 5-hydroxyisourate hydrolase pseudogene (LOC100130015), transcript variant 1, non-coding RNA [NR_027336] |
| XLOC_l2_003666 | TCONS_l2_00006771 | linc|BROAD Institute lincRNA (XLOC_l2_003666), lincRNA [TCONS_l2_00006771] |
| DENND5B | NM_144973 | DENN/MADD domain containing 5B (DENND5B), mRNA [NM_144973] |
| TNFRSF19 | NM_018647 | tumor necrosis factor receptor superfamily, member 19 (TNFRSF19), transcript variant 1, mRNA [NM_018647] |
| ZNF169 | NM_194320 | zinc finger protein 169 (ZNF169), mRNA [NM_194320] |
| XLOC_l2_004385 | TCONS_l2_00008041 | linc|BROAD Institute lincRNA (XLOC_l2_004385), lincRNA [TCONS_l2_00008041] |
| A_33_P3265624 | A_33_P3265624 | Unknown |
| XLOC_l2_004192 | ENST00000454942 | tc|Q86TZ0_HUMAN (Q86TZ0) Full-length cDNA clone CS0DC023YN15 of Neuroblastoma of Homo sapiens (human) (Fragment), partial (34%) [THC2784767] |
| PDE4D | NM_001165899 | phosphodiesterase 4D, cAMP-specific (PDE4D), transcript variant 3, mRNA [NM_001165899] |
| MIR143HG | NR_027180 | MIR143 host gene (non-protein coding) (MIR143HG), non-coding RNA [NR_027180] |
| F7 | NM_000131 | coagulation factor VII (serum prothrombin conversion accelerator) (F7), transcript variant 1, mRNA [NM_000131] |
| FAM32A | NM_014077 | family with sequence similarity 32, member A (FAM32A), mRNA [NM_014077] |
| LOC100505876 | NR_037879 | uncharacterized LOC100505876 (LOC100505876), transcript variant 1, non-coding RNA [NR_037879] |
| ENST00000421204 | ENST00000421204 | Unknown |
| C7orf58 | NM_024913 | chromosome 7 open reading frame 58 (C7orf58), transcript variant 1, mRNA [NM_024913] |
| DNAI2 | NM_023036 | dynein, axonemal, intermediate chain 2 (DNAI2), transcript variant 1, mRNA [NM_023036] |
| NLRP14 | NM_176822 | NLR family, pyrin domain containing 14 (NLRP14), mRNA [NM_176822] |
| XLOC_l2_006425 | ENST00000507941 | linc|BROAD Institute lincRNA (XLOC_l2_006425), lincRNA [TCONS_l2_00011998] |
| FAM135B | NM_015912 | family with sequence similarity 135, member B (FAM135B), mRNA [NM_015912] |
| OTUD1 | NM_001145373 | OTU domain containing 1 (OTUD1), mRNA [NM_001145373] |
| ENST00000435913 | ENST00000435913 | Unknown |
| PABPC1L2B | NM_001042506 | poly(A) binding protein, cytoplasmic 1-like 2B (PABPC1L2B), mRNA [NM_001042506] |
| XLOC_l2_008416 | TCONS_l2_00015083 | linc|BROAD Institute lincRNA (XLOC_l2_008416), lincRNA [TCONS_l2_00015083] |
| ENST00000381261 | ENST00000381261 | ens|A kinase (PRKA) anchor protein 17A [Source:HGNC Symbol;Acc:18783] [ENST00000381261] |
| SPP1 | NM_001040058 | secreted phosphoprotein 1 (SPP1), transcript variant 1, mRNA [NM_001040058] |
| CEP135 | NM_025009 | centrosomal protein 135kDa (CEP135), mRNA [NM_025009] |
| XLOC_l2_005020 | TCONS_l2_00009251 | linc|BROAD Institute lincRNA (XLOC_l2_005020), lincRNA [TCONS_l2_00009251] |
| LOC100506858 | XR_108581 | ref|PREDICTED: Homo sapiens hypothetical LOC100506858 (LOC100506858), miscRNA [XR_108581] |
| CR745709 | CR745709 | gb|CR745709 Homo sapiens library (Ebert L) Homo sapiens cDNA clone IMAGp971N1473 ; IMAGE:1715597 5', mRNA sequence [CR745709] |
| ZNF461 | NM_153257 | zinc finger protein 461 (ZNF461), mRNA [NM_153257] |
| ZNF804B | NM_181646 | zinc finger protein 804B (ZNF804B), mRNA [NM_181646] |
| DIS3L2 | NM_152383 | DIS3 mitotic control homolog (S. cerevisiae)-like 2 (DIS3L2), mRNA [NM_152383] |
| A_33_P3362601 | A_33_P3362601 | Unknown |
| ERV18-1 | AK126787 | gb|Homo sapiens cDNA FLJ44837 fis, clone BRACE3048677. [AK126787] |
| ENST00000390392 | ENST00000390392 | ens|T cell receptor beta variable 4-2 [Source:HGNC Symbol;Acc:12216] [ENST00000390392] |
| HIC2 | NM_015094 | hypermethylated in cancer 2 (HIC2), mRNA [NM_015094] |
| CLCA3P | NR_024604 | chloride channel accessory 3, pseudogene (CLCA3P), non-coding RNA [NR_024604] |
| XLOC_006529 | TCONS_00013897 | linc|BROAD Institute lincRNA (XLOC_006529), lincRNA [TCONS_00013897] |
| DDIT3 | NM_004083 | DNA-damage-inducible transcript 3 (DDIT3), transcript variant 5, mRNA [NM_004083] |
| DA380926 | DA380926 | gb|DA380926 BRTHA2 Homo sapiens cDNA clone BRTHA2014320 5', mRNA sequence [DA380926] |
| LSM11 | NM_173491 | LSM11, U7 small nuclear RNA associated (LSM11), mRNA [NM_173491] |
| DYNLL2 | NM_080677 | dynein, light chain, LC8-type 2 (DYNLL2), mRNA [NM_080677] |
| LOC100652957 | XR_132533 | ref|PREDICTED: Homo sapiens hypothetical LOC100652957 (LOC100652957), miscRNA [XR_132533] |
| XLOC_l2_015219 | TCONS_l2_00029375 | linc|BROAD Institute lincRNA (XLOC_l2_015219), lincRNA [TCONS_l2_00029375] |
| TFPI | ENST00000481132 | ens|tissue factor pathway inhibitor (lipoprotein-associated coagulation inhibitor) [Source:HGNC Symbol;Acc:11760] [ENST00000481132] |
| IL12RB2 | NM_001559 | interleukin 12 receptor, beta 2 (IL12RB2), mRNA [NM_001559] |
| SLC45A2 | NM_016180 | solute carrier family 45, member 2 (SLC45A2), transcript variant 1, mRNA [NM_016180] |
| XLOC_l2_002049 | TCONS_l2_00003697 | linc|BROAD Institute lincRNA (XLOC_l2_002049), lincRNA [TCONS_l2_00003697] |
| ZNF137P | BG482973 | gb|602502986F1 NIH_MGC_77 Homo sapiens cDNA clone IMAGE:4616589 5', mRNA sequence [BG482973] |
| LIM2 | NM_030657 | lens intrinsic membrane protein 2, 19kDa (LIM2), transcript variant 1, mRNA [NM_030657] |
| SOX7 | NM_031439 | SRY (sex determining region Y)-box 7 (SOX7), mRNA [NM_031439] |
| XLOC_010073 | TCONS_00021310 | linc|BROAD Institute lincRNA (XLOC_010073), lincRNA [TCONS_00021310] |
| SLTM | ENST00000480144 | ens|SAFB-like, transcription modulator [Source:HGNC Symbol;Acc:20709] [ENST00000480144] |
| UXT | ENST00000376964 | ens|ubiquitously-expressed transcript [Source:HGNC Symbol;Acc:12641] [ENST00000376964] |
| ZNF451 | NM_001031623 | zinc finger protein 451 (ZNF451), transcript variant 1, mRNA [NM_001031623] |
| EHF | NM_012153 | ets homologous factor (EHF), transcript variant 2, mRNA [NM_012153] |
| CTAGE7P | NR_044994 | CTAGE family, member 7, pseudogene (CTAGE7P), non-coding RNA [NR_044994] |
| XLOC_004918 | ENST00000508719 | tc|Q3X7A0_METFL (Q3X7A0) Transport system permease protein precursor, partial (6%) [THC2690553] |
| ACTA1 | NM_001100 | actin, alpha 1, skeletal muscle (ACTA1), mRNA [NM_001100] |
| ANKRD44 | ENST00000328737 | ens|ankyrin repeat domain 44 [Source:HGNC Symbol;Acc:25259] [ENST00000328737] |
| LOC151475 | NR_040038 | uncharacterized LOC151475 (LOC151475), non-coding RNA [NR_040038] |
| DONSON | AK001274 | gb|Homo sapiens cDNA FLJ10412 fis, clone NT2RP1000040. [AK001274] |
| ENST00000482201 | ENST00000482201 | ens|olfactory receptor, family 9, subfamily A, member 1 pseudogene [Source:HGNC Symbol;Acc:8486] [ENST00000482201] |
| XLOC_l2_010494 | THC2659061 | linc|BROAD Institute lincRNA (XLOC_l2_010494), lincRNA [TCONS_l2_00020305] |
| NRIP3 | NM_020645 | nuclear receptor interacting protein 3 (NRIP3), mRNA [NM_020645] |
| A_33_P3333058 | A_33_P3333058 | Unknown |
| MAGEA2B | NM_153488 | melanoma antigen family A, 2B (MAGEA2B), mRNA [NM_153488] |
| FBXO33 | NM_203301 | F-box protein 33 (FBXO33), mRNA [NM_203301] |
| RAB11FIP1 | NM_001002814 | RAB11 family interacting protein 1 (class I) (RAB11FIP1), transcript variant 3, mRNA [NM_001002814] |
| THC2750697 | THC2750697 | Unknown |
| XLOC_003427 | ENST00000418666 | Unknown |
| XLOC_004925 | ENST00000513779 | linc|BROAD Institute lincRNA (XLOC_004925), lincRNA [TCONS_00009726] |
| XLOC_005372 | ENST00000444796 | gb|DA357073 BRSTN2 Homo sapiens cDNA clone BRSTN2002344 5', mRNA sequence [DA357073] |
| SFT2D3 | NM_032740 | SFT2 domain containing 3 (SFT2D3), mRNA [NM_032740] |
| TMEM196 | NM_152774 | transmembrane protein 196 (TMEM196), mRNA [NM_152774] |
| TRAM1 | NM_014294 | translocation associated membrane protein 1 (TRAM1), mRNA [NM_014294] |
| XLOC_005582 | ENST00000455327 | linc|BROAD Institute lincRNA (XLOC_005582), lincRNA [TCONS_00011431] |
| EIF4G2 | NM_001418 | eukaryotic translation initiation factor 4 gamma, 2 (EIF4G2), transcript variant 1, mRNA [NM_001418] |
| LOC100652787 | XR_132806 | ref|PREDICTED: Homo sapiens hypothetical LOC100652787 (LOC100652787), miscRNA [XR_132806] |
| HTR3C | NM_130770 | 5-hydroxytryptamine (serotonin) receptor 3, family member C (HTR3C), mRNA [NM_130770] |
| LOC100652987 | XR_132752 | ref|PREDICTED: Homo sapiens hypothetical LOC100652987 (LOC100652987), miscRNA [XR_132752] |
| SNORA16B | CU457294 | gb|CU457294 Homo sapiens ORESTES from keratinocytes Homo sapiens cDNA, mRNA sequence [CU457294] |
| C8orf37 | NM_177965 | chromosome 8 open reading frame 37 (C8orf37), mRNA [NM_177965] |
| XLOC_l2_008996 | TCONS_l2_00016983 | linc|BROAD Institute lincRNA (XLOC_l2_008996), lincRNA [TCONS_l2_00016983] |
| XLOC_003829 | TCONS_00008374 | linc|BROAD Institute lincRNA (XLOC_003829), lincRNA [TCONS_00008374] |
| DEFB113 | NM_001037729 | defensin, beta 113 (DEFB113), mRNA [NM_001037729] |
| ATOH8 | NM_032827 | atonal homolog 8 (Drosophila) (ATOH8), mRNA [NM_032827] |
| LOC100130276 | AK128269 | gb|Homo sapiens cDNA FLJ46406 fis, clone THYMU3009755. [AK128269] |
| PRORSD1P | NR_027258 | prolyl-tRNA synthetase associated domain containing 1, pseudogene (PRORSD1P), non-coding RNA [NR_027258] |
| A_33_P3297823 | A_33_P3297823 | Unknown |
| XLOC_008835 | ENST00000433019 | linc|BROAD Institute lincRNA (XLOC_008835), lincRNA [TCONS_00018940] |
| XLOC_008985 | ENST00000456514 | linc|BROAD Institute lincRNA (XLOC_008985), lincRNA [TCONS_00018066] |
| CCT7 | NM_006429 | chaperonin containing TCP1, subunit 7 (eta) (CCT7), transcript variant 1, mRNA [NM_006429] |
| BG036557 | BG036557 | gb|602326332F1 NIH_MGC_91 Homo sapiens cDNA clone IMAGE:4428126 5', mRNA sequence [BG036557] |
| AK091251 | AK091251 | gb|Homo sapiens cDNA FLJ33932 fis, clone CTONG2017798. [AK091251] |
| TFRC | NM_003234 | transferrin receptor (p90, CD71) (TFRC), transcript variant 1, mRNA [NM_003234] |
| SSPO | NM_198455 | SCO-spondin homolog (Bos taurus) (SSPO), mRNA [NM_198455] |
| TMEM231 | NM_001077419 | transmembrane protein 231 (TMEM231), transcript variant 3, mRNA [NM_001077419] |
| NRP1 | ENST00000374818 | ens|neuropilin 1 [Source:HGNC Symbol;Acc:8004] [ENST00000374818] |
| ANKRD62 | ENST00000314074 | ens|ankyrin repeat domain 62 [Source:HGNC Symbol;Acc:35241] [ENST00000314074] |
| SNAP25 | NM_003081 | synaptosomal-associated protein, 25kDa (SNAP25), transcript variant 1, mRNA [NM_003081] |
| SMG7 | ENST00000367538 | ens|smg-7 homolog, nonsense mediated mRNA decay factor (C. elegans) [Source:HGNC Symbol;Acc:16792] [ENST00000367538] |
| PDPR | NM_017990 | pyruvate dehydrogenase phosphatase regulatory subunit (PDPR), mRNA [NM_017990] |
| HSD17B7 | ENST00000367913 | ens|hydroxysteroid (17-beta) dehydrogenase 7 [Source:HGNC Symbol;Acc:5215] [ENST00000367913] |
| LOC91948 | AK025311 | gb|Homo sapiens cDNA: FLJ21658 fis, clone COL08688. [AK025311] |
| LOC100131490 | AK124265 | gb|Homo sapiens cDNA FLJ42271 fis, clone TKIDN2015788. [AK124265] |
| XLOC_000980 | ENST00000440801 | Unknown |
| PKD2L2 | ENST00000350250 | ens|polycystic kidney disease 2-like 2 [Source:HGNC Symbol;Acc:9012] [ENST00000350250] |
| XLOC_006684 | TCONS_00014573 | linc|BROAD Institute lincRNA (XLOC_006684), lincRNA [TCONS_00014573] |
| LOC390660 | XR_109177 | ref|PREDICTED: Homo sapiens FLJ00317 protein (LOC390660), miscRNA [XR_109177] |
| NACA2 | NM_199290 | nascent polypeptide-associated complex alpha subunit 2 (NACA2), mRNA [NM_199290] |
| XLOC_011648 | THC2662756 | linc|BROAD Institute lincRNA (XLOC_011648), lincRNA [TCONS_00024332] |
| ACSM2A | AK091978 | gb|Homo sapiens cDNA FLJ34659 fis, clone KIDNE2018863. [AK091978] |
| GABARAPL1 | NM_031412 | GABA(A) receptor-associated protein like 1 (GABARAPL1), mRNA [NM_031412] |
| A_33_P3225128 | A_33_P3225128 | Unknown |
| FAM179B | NM_015091 | family with sequence similarity 179, member B (FAM179B), mRNA [NM_015091] |
| XLOC_005164 | TCONS_00012423 | linc|BROAD Institute lincRNA (XLOC_005164), lincRNA [TCONS_00012423] |
| SLC6A13 | NM_001243392 | solute carrier family 6 (neurotransmitter transporter, GABA), member 13 (SLC6A13), transcript variant 3, mRNA [NM_001243392] |
| SLMAP | ENST00000467901 | ens|sarcolemma associated protein [Source:HGNC Symbol;Acc:16643] [ENST00000467901] |
| SYDE2 | ENST00000234668 | ens|synapse defective 1, Rho GTPase, homolog 2 (C. elegans) [Source:HGNC Symbol;Acc:25841] [ENST00000234668] |
| LOC100507059 | XR_109002 | ref|PREDICTED: Homo sapiens hypothetical LOC100507059 (LOC100507059), miscRNA [XR_109002] |
| YTHDC1 | NM_001031732 | YTH domain containing 1 (YTHDC1), transcript variant 1, mRNA [NM_001031732] |
| FNDC8 | NM_017559 | fibronectin type III domain containing 8 (FNDC8), mRNA [NM_017559] |
| TTLL11 | NM_194252 | tubulin tyrosine ligase-like family, member 11 (TTLL11), transcript variant 2, mRNA [NM_194252] |
| MRPL3 | NM_007208 | mitochondrial ribosomal protein L3 (MRPL3), nuclear gene encoding mitochondrial protein, mRNA [NM_007208] |
| LOC100505769 | XR_109186 | ref|PREDICTED: Homo sapiens hypothetical LOC100505769 (LOC100505769), miscRNA [XR_109186] |
| ENST00000418620 | ENST00000418620 | gb|CR736977 Soares_testis_NHT Homo sapiens cDNA clone IMAGp971B2176 ; IMAGE:1755160 5', mRNA sequence [CR736977] |
| XLOC_007195 | TCONS_00015151 | linc|BROAD Institute lincRNA (XLOC_007195), lincRNA [TCONS_00015151] |
| SIRPB1 | NM_001135844 | signal-regulatory protein beta 1 (SIRPB1), transcript variant 3, mRNA [NM_001135844] |
| XLOC_010847 | TCONS_00022524 | linc|BROAD Institute lincRNA (XLOC_010847), lincRNA [TCONS_00022524] |
| LOC100129520 | NM_001195272 | testis expressed sequence 13-like (LOC100129520), mRNA [NM_001195272] |
| XLOC_l2_003753 | ENST00000431656 | linc|BROAD Institute lincRNA (XLOC_l2_003753), lincRNA [TCONS_l2_00007418] |
| XLOC_006709 | TCONS_00015214 | linc|BROAD Institute lincRNA (XLOC_006709), lincRNA [TCONS_00015214] |
| CROCCP2 | AK123337 | gb|Homo sapiens cDNA FLJ41343 fis, clone BRAWH2001973. [AK123337] |
| XLOC_013657 | TCONS_00028320 | linc|BROAD Institute lincRNA (XLOC_013657), lincRNA [TCONS_00028320] |
| MYO9A | NM_006901 | myosin IXA (MYO9A), mRNA [NM_006901] |
| PTPRJ | NM_002843 | protein tyrosine phosphatase, receptor type, J (PTPRJ), transcript variant 1, mRNA [NM_002843] |
| XLOC_006933 | THC2649910 | linc|BROAD Institute lincRNA (XLOC_006933), lincRNA [TCONS_00014864] |
| C20orf112 | NM_080616 | chromosome 20 open reading frame 112 (C20orf112), mRNA [NM_080616] |
| KRTCAP3 | NM_173853 | keratinocyte associated protein 3 (KRTCAP3), transcript variant 2, mRNA [NM_173853] |
| XLOC_008942 | TCONS_00018599 | linc|BROAD Institute lincRNA (XLOC_008942), lincRNA [TCONS_00018599] |
| XLOC_001417 | THC2568759 | tc|ALU1_HUMAN (P39188) Alu subfamily J sequence contamination warning entry, partial (6%) [THC2568759] |
| XLOC_013664 | TCONS_00028323 | linc|BROAD Institute lincRNA (XLOC_013664), lincRNA [TCONS_00028323] |
| EBF3 | ENST00000440978 | ens|early B-cell factor 3 [Source:HGNC Symbol;Acc:19087] [ENST00000440978] |
| TDRD3 | ENST00000484389 | ens|tudor domain containing 3 [Source:HGNC Symbol;Acc:20612] [ENST00000484389] |
| KCTD16 | NM_020768 | potassium channel tetramerisation domain containing 16 (KCTD16), mRNA [NM_020768] |
| A_33_P3336148 | A_33_P3336148 | Unknown |
| XLOC_008381 | TCONS_00018678 | linc|BROAD Institute lincRNA (XLOC_008381), lincRNA [TCONS_00018678] |
| ENST00000417510 | ENST00000417510 | gb|full-length cDNA clone CS0DI013YN06 of Placenta Cot 25-normalized of Homo sapiens (human) [CR597597] |
| XLOC_002275 | TCONS_00004391 | linc|BROAD Institute lincRNA (XLOC_002275), lincRNA [TCONS_00004391] |
| SNORD52 | NR_002742 | small nucleolar RNA, C/D box 52 (SNORD52), small nuclear RNA [NR_002742] |
| XLOC_l2_001592 | TCONS_l2_00003049 | linc|BROAD Institute lincRNA (XLOC_l2_001592), lincRNA [TCONS_l2_00003049] |
| SKP2 | NM_032637 | S-phase kinase-associated protein 2 (p45) (SKP2), transcript variant 2, mRNA [NM_032637] |
| XLOC_l2_012743 | TCONS_l2_00024448 | linc|BROAD Institute lincRNA (XLOC_l2_012743), lincRNA [TCONS_l2_00024448] |
| PIAS2 | NM_004671 | protein inhibitor of activated STAT, 2 (PIAS2), transcript variant beta, mRNA [NM_004671] |
| ZNF639 | NM_016331 | zinc finger protein 639 (ZNF639), mRNA [NM_016331] |
| CELA1 | NM_001971 | chymotrypsin-like elastase family, member 1 (CELA1), mRNA [NM_001971] |
| RDH14 | NM_020905 | retinol dehydrogenase 14 (all-trans/9-cis/11-cis) (RDH14), mRNA [NM_020905] |
| LPPR1 | NM_207299 | lipid phosphate phosphatase-related protein type 1 (LPPR1), transcript variant 1, mRNA [NM_207299] |
| XLOC_006702 | ENST00000520332 | Unknown |
| RP1 | NM_006269 | retinitis pigmentosa 1 (autosomal dominant) (RP1), mRNA [NM_006269] |
| STMN1 | NM_001145454 | stathmin 1 (STMN1), transcript variant 4, mRNA [NM_001145454] |
| XLOC_012053 | THC2654949 | linc|BROAD Institute lincRNA (XLOC_012053), lincRNA [TCONS_00025091] |
| HIST1H3B | NM_003537 | histone cluster 1, H3b (HIST1H3B), mRNA [NM_003537] |
| HUNK | NM_014586 | hormonally up-regulated Neu-associated kinase (HUNK), mRNA [NM_014586] |
| LOC100507373 | NR_045214 | uncharacterized LOC100507373 (LOC100507373), non-coding RNA [NR_045214] |
| ENST00000374801 | ENST00000374801 | ens|chromosome 9 open reading frame 107 [Source:HGNC Symbol;Acc:31372] [ENST00000374801] |
| XLOC_004178 | ENST00000506984 | gb|RST4985 Athersys RAGE Library Homo sapiens cDNA, mRNA sequence [BG186029] |
| ZFR2 | NM_015174 | zinc finger RNA binding protein 2 (ZFR2), transcript variant 1, mRNA [NM_015174] |
| PDZRN4 | NM_013377 | PDZ domain containing ring finger 4 (PDZRN4), transcript variant 2, mRNA [NM_013377] |
| XLOC_010560 | ENST00000415283 | linc|BROAD Institute lincRNA (XLOC_010560), lincRNA [TCONS_00021631] |
| DUOX1 | NM_017434 | dual oxidase 1 (DUOX1), transcript variant 1, mRNA [NM_017434] |
| LRRC6 | NM_012472 | leucine rich repeat containing 6 (LRRC6), mRNA [NM_012472] |
| CHST1 | NM_003654 | carbohydrate (keratan sulfate Gal-6) sulfotransferase 1 (CHST1), mRNA [NM_003654] |
| HABP2 | NM_004132 | hyaluronan binding protein 2 (HABP2), transcript variant 1, mRNA [NM_004132] |
| XLOC_l2_003292 | TCONS_l2_00006110 | linc|BROAD Institute lincRNA (XLOC_l2_003292), lincRNA [TCONS_l2_00006110] |
| ENST00000455269 | ENST00000455269 | Unknown |
| XLOC_012338 | ENST00000442532 | linc|BROAD Institute lincRNA (XLOC_012338), lincRNA [TCONS_00025202] |
| UNC93A | NM_018974 | unc-93 homolog A (C. elegans) (UNC93A), transcript variant 1, mRNA [NM_018974] |
| A_33_P3305820 | A_33_P3305820 | Unknown |
| LOC100616530 | NR_038209 | tospeak (LOC100616530), transcript variant 9, non-coding RNA [NR_038209] |
| XLOC_012687 | TCONS_00026625 | linc|BROAD Institute lincRNA (XLOC_012687), lincRNA [TCONS_00026625] |
| ZSCAN20 | NM_145238 | zinc finger and SCAN domain containing 20 (ZSCAN20), mRNA [NM_145238] |
| SYT10 | NM_198992 | synaptotagmin X (SYT10), mRNA [NM_198992] |
| DKFZp564H213 | AL049275 | tc|GB|AL049275.1|AL049275.1 Homo sapiens mRNA; cDNA DKFZp564H213 (from clone DKFZp564H213) [NP1168846] |
| LDLRAD1 | NM_001010978 | low density lipoprotein receptor class A domain containing 1 (LDLRAD1), mRNA [NM_001010978] |
| XLOC_006107 | TCONS_00014135 | linc|BROAD Institute lincRNA (XLOC_006107), lincRNA [TCONS_00014135] |
| KIAA1244 | NM_020340 | KIAA1244 (KIAA1244), mRNA [NM_020340] |
| GDE1 | NM_016641 | glycerophosphodiester phosphodiesterase 1 (GDE1), mRNA [NM_016641] |
| DLGAP1 | NM_004746 | discs, large (Drosophila) homolog-associated protein 1 (DLGAP1), transcript variant 1, mRNA [NM_004746] |
| XLOC_003235 | ENST00000514281 | linc|BROAD Institute lincRNA (XLOC_003235), lincRNA [TCONS_00007251] |
| ARID5B | NM_032199 | AT rich interactive domain 5B (MRF1-like) (ARID5B), transcript variant 1, mRNA [NM_032199] |
| XLOC_l2_010908 | BG194895 | gb|RST13952 Athersys RAGE Library Homo sapiens cDNA, mRNA sequence [BG194895] |
| CFL2 | NM_021914 | cofilin 2 (muscle) (CFL2), transcript variant 1, mRNA [NM_021914] |
| ZNRF4 | NM_181710 | zinc and ring finger 4 (ZNRF4), mRNA [NM_181710] |
| LOC51145 | XR_108997 | ref|PREDICTED: Homo sapiens erythrocyte transmembrane protein (LOC51145), miscRNA [XR_108997] |
| OR4K13 | NM_001004714 | olfactory receptor, family 4, subfamily K, member 13 (OR4K13), mRNA [NM_001004714] |
| FAM104A | NM_032837 | family with sequence similarity 104, member A (FAM104A), transcript variant 2, mRNA [NM_032837] |
| ZNF548 | NM_001172773 | zinc finger protein 548 (ZNF548), transcript variant 1, mRNA [NM_001172773] |
| ENST00000445310 | ENST00000445310 | ens|KCNQ5 intronic transcript 1 (non-protein coding) [Source:HGNC Symbol;Acc:41354] [ENST00000445310] |
| LOC285740 | NR_027114 | uncharacterized LOC285740 (LOC285740), non-coding RNA [NR_027114] |
| XLOC_004270 | TCONS_00010610 | linc|BROAD Institute lincRNA (XLOC_004270), lincRNA [TCONS_00010610] |
| C2CD4A | NM_207322 | C2 calcium-dependent domain containing 4A (C2CD4A), mRNA [NM_207322] |
| XLOC_008177 | ENST00000454131 | linc|BROAD Institute lincRNA (XLOC_008177), lincRNA [TCONS_00017063] |
| MAGEA1 | NM_004988 | melanoma antigen family A, 1 (directs expression of antigen MZ2-E) (MAGEA1), mRNA [NM_004988] |
| XLOC_009762 | TCONS_00020432 | linc|BROAD Institute lincRNA (XLOC_009762), lincRNA [TCONS_00020432] |
| XLOC_l2_012614 | TCONS_l2_00024298 | linc|BROAD Institute lincRNA (XLOC_l2_012614), lincRNA [TCONS_l2_00024298] |
| LOC100653003 | XR_132788 | ref|PREDICTED: Homo sapiens hypothetical LOC100653003, transcript variant 2 (LOC100653003), miscRNA [XR_132788] |
| XLOC_001259 | ENST00000457698 | tc|ALU6_HUMAN (P39193) Alu subfamily SP sequence contamination warning entry, partial (19%) [THC2618738] |
| C9orf103 | NM_001001551 | chromosome 9 open reading frame 103 (C9orf103), transcript variant 1, mRNA [NM_001001551] |
| FLJ31104 | XR_108600 | ref|PREDICTED: Homo sapiens hypothetical LOC441072 (FLJ31104), miscRNA [XR_108600] |
| SNORD115-3 | NR_003295 | small nucleolar RNA, C/D box 115-3 (SNORD115-3), small nucleolar RNA [NR_003295] |
| LOC284408 | NR_040027 | uncharacterized LOC284408 (LOC284408), transcript variant 1, non-coding RNA [NR_040027] |
| DDX52 | NM_007010 | DEAD (Asp-Glu-Ala-Asp) box polypeptide 52 (DDX52), mRNA [NM_007010] |
| LOC100506540 | NR_038360 | uncharacterized LOC100506540 (LOC100506540), non-coding RNA [NR_038360] |
| PTK2 | NM_153831 | PTK2 protein tyrosine kinase 2 (PTK2), transcript variant 1, mRNA [NM_153831] |
| XLOC_012162 | TCONS_00025888 | linc|BROAD Institute lincRNA (XLOC_012162), lincRNA [TCONS_00025888] |
| ANKRD33B | NM_001164440 | ankyrin repeat domain 33B (ANKRD33B), mRNA [NM_001164440] |
| ANAPC1 | NM_022662 | anaphase promoting complex subunit 1 (ANAPC1), mRNA [NM_022662] |
| A_33_P3381044 | A_33_P3381044 | Unknown |
| C21orf81 | BC036580 | gb|DB570154 RIKEN full-length enriched human cDNA library, hypothalamus Homo sapiens cDNA clone H033071O06 3', mRNA sequence [DB570154] |
| CHRNA4 | NM_000744 | cholinergic receptor, nicotinic, alpha 4 (CHRNA4), mRNA [NM_000744] |
| MAEL | NM_032858 | maelstrom homolog (Drosophila) (MAEL), mRNA [NM_032858] |
| CASP2 | NM_032982 | caspase 2, apoptosis-related cysteine peptidase (CASP2), transcript variant 1, mRNA [NM_032982] |
| XLOC_012428 | TCONS_00025600 | linc|BROAD Institute lincRNA (XLOC_012428), lincRNA [TCONS_00025600] |
| CHRM5 | NM_012125 | cholinergic receptor, muscarinic 5 (CHRM5), mRNA [NM_012125] |
| ZNF75A | NM_153028 | zinc finger protein 75a (ZNF75A), mRNA [NM_153028] |
| XLOC_l2_000727 | TCONS_l2_00000968 | linc|BROAD Institute lincRNA (XLOC_l2_000727), lincRNA [TCONS_l2_00000968] |
| OR2AE1 | NM_001005276 | olfactory receptor, family 2, subfamily AE, member 1 (OR2AE1), mRNA [NM_001005276] |
| HUS1B | NM_148959 | HUS1 checkpoint homolog b (S. pombe) (HUS1B), mRNA [NM_148959] |
| XLOC_010808 | TCONS_00022479 | linc|BROAD Institute lincRNA (XLOC_010808), lincRNA [TCONS_00022479] |
| MTUS1 | NM_001001924 | microtubule associated tumor suppressor 1 (MTUS1), transcript variant 1, mRNA [NM_001001924] |
| XLOC_011259 | TCONS_00023414 | linc|BROAD Institute lincRNA (XLOC_011259), lincRNA [TCONS_00023414] |
| CGRRF1 | NM_006568 | cell growth regulator with ring finger domain 1 (CGRRF1), mRNA [NM_006568] |
| ADAMTS1 | NM_006988 | ADAM metallopeptidase with thrombospondin type 1 motif, 1 (ADAMTS1), mRNA [NM_006988] |
| LOC100506950 | ENST00000415255 | ref|PREDICTED: Homo sapiens hypothetical LOC100506950 (LOC100506950), miscRNA [XR_110500] |
| C18orf63 | NM_001174123 | chromosome 18 open reading frame 63 (C18orf63), mRNA [NM_001174123] |
| AK094155 | AK094155 | gb|Homo sapiens cDNA FLJ36836 fis, clone ASTRO2011149. [AK094155] |
| XLOC_006976 | TCONS_00014909 | linc|BROAD Institute lincRNA (XLOC_006976), lincRNA [TCONS_00014909] |
| A_33_P3305526 | A_33_P3305526 | Unknown |
| POLR1D | NM_152705 | polymerase (RNA) I polypeptide D, 16kDa (POLR1D), transcript variant 2, mRNA [NM_152705] |
| LOC100134937 | BC035129 | gb|Homo sapiens cDNA clone IMAGE:5263734. [BC035129] |
| XLOC_l2_009572 | DT932569 | gb|MGC8.7.1.1.1.G03.F.1 NIH_MGC_331 Homo sapiens cDNA clone MGC8.7.1.1.1.G03, mRNA sequence [DT932569] |
| XLOC_012586 | TCONS_00026175 | linc|BROAD Institute lincRNA (XLOC_012586), lincRNA [TCONS_00026175] |
| ENST00000514853 | ENST00000514853 | ref|PREDICTED: Homo sapiens hCG1981531 (LOC728586), miscRNA [XR_110564] |
| TAB3 | ENST00000378928 | ens|TGF-beta activated kinase 1/MAP3K7 binding protein 3 [Source:HGNC Symbol;Acc:30681] [ENST00000378928] |
| SCN7A | NM_002976 | sodium channel, voltage-gated, type VII, alpha (SCN7A), mRNA [NM_002976] |
| CAGE1 | NM_001170692 | cancer antigen 1 (CAGE1), transcript variant 1, mRNA [NM_001170692] |
| XLOC_001809 | TCONS_00003986 | linc|BROAD Institute lincRNA (XLOC_001809), lincRNA [TCONS_00003986] |
| KRT37 | NM_003770 | keratin 37 (KRT37), mRNA [NM_003770] |
| XLOC_l2_009009 | THC2783854 | linc|BROAD Institute lincRNA (XLOC_l2_009009), lincRNA [TCONS_l2_00016988] |
| XLOC_012167 | TCONS_00025347 | linc|BROAD Institute lincRNA (XLOC_012167), lincRNA [TCONS_00025347] |
| ENST00000440540 | ENST00000440540 | tc|BC034563 ACBD3 protein {Homo sapiens} (exp=-1; wgp=0; cg=0), partial (17%) [THC2682346] |
| A_33_P3252444 | A_33_P3252444 | Unknown |
| XLOC_005596 | TCONS_00011443 | linc|BROAD Institute lincRNA (XLOC_005596), lincRNA [TCONS_00011443] |
| MYO6 | NM_004999 | myosin VI (MYO6), mRNA [NM_004999] |
| HERC3 | BC038960 | gb|Homo sapiens hect domain and RLD 3, mRNA (cDNA clone IMAGE:6050308), complete cds. [BC038960] |
| XLOC_003665 | TCONS_00008209 | linc|BROAD Institute lincRNA (XLOC_003665), lincRNA [TCONS_00008209] |
| XLOC_004546 | TCONS_00010087 | linc|BROAD Institute lincRNA (XLOC_004546), lincRNA [TCONS_00010087] |
| XLOC_011213 | TCONS_00023356 | linc|BROAD Institute lincRNA (XLOC_011213), lincRNA [TCONS_00023356] |
| LOC375295 | NR_040001 | uncharacterized LOC375295 (LOC375295), non-coding RNA [NR_040001] |
| SLC35F5 | ENST00000498768 | ens|solute carrier family 35, member F5 [Source:HGNC Symbol;Acc:23617] [ENST00000498768] |
| DBC1 | ENST00000373964 | ens|deleted in bladder cancer 1 [Source:HGNC Symbol;Acc:2687] [ENST00000373964] |
| LOC100507043 | NR_038861 | uncharacterized LOC100507043 (LOC100507043), non-coding RNA [NR_038861] |
| TSPAN14 | NM_030927 | tetraspanin 14 (TSPAN14), transcript variant 1, mRNA [NM_030927] |
| ATPAF1 | ENST00000371937 | ens|ATP synthase mitochondrial F1 complex assembly factor 1 [Source:HGNC Symbol;Acc:18803] [ENST00000371937] |
| SNORA53 | NR_003015 | small nucleolar RNA, H/ACA box 53 (SNORA53), small nucleolar RNA [NR_003015] |
| CLMP | NM_024769 | CXADR-like membrane protein (CLMP), mRNA [NM_024769] |
| FAM189A2 | NM_004816 | family with sequence similarity 189, member A2 (FAM189A2), transcript variant 1, mRNA [NM_004816] |
| SLC10A7 | NM_001029998 | solute carrier family 10 (sodium/bile acid cotransporter family), member 7 (SLC10A7), transcript variant 2, mRNA [NM_001029998] |
| LOC100128651 | AK127243 | gb|Homo sapiens cDNA FLJ45310 fis, clone BRHIP3004774. [AK127243] |
| XLOC_l2_001273 | TCONS_l2_00001718 | linc|BROAD Institute lincRNA (XLOC_l2_001273), lincRNA [TCONS_l2_00001718] |
| XLOC_002250 | ENST00000455614 | linc|BROAD Institute lincRNA (XLOC_002250), lincRNA [TCONS_00003361] |
| RSPH1 | NM_080860 | radial spoke head 1 homolog (Chlamydomonas) (RSPH1), mRNA [NM_080860] |
| LOC100505844 | XR_108529 | ref|PREDICTED: Homo sapiens hypothetical LOC100505844 (LOC100505844), miscRNA [XR_108529] |
| ENST00000367690 | ENST00000367690 | ens|RAB GTPase activating protein 1-like [Source:HGNC Symbol;Acc:24663] [ENST00000367690] |
| GNL3L | NM_019067 | guanine nucleotide binding protein-like 3 (nucleolar)-like (GNL3L), transcript variant 2, mRNA [NM_019067] |
| FAM83A | NM_207006 | family with sequence similarity 83, member A (FAM83A), transcript variant 2, mRNA [NM_207006] |
| C3orf27 | NM_007354 | chromosome 3 open reading frame 27 (C3orf27), mRNA [NM_007354] |
| LOC100507173 | NR_038293 | uncharacterized LOC100507173 (LOC100507173), transcript variant 2, non-coding RNA [NR_038293] |
| A_33_P3262217 | A_33_P3262217 | Unknown |
| XLOC_l2_008190 | ENST00000445083 | gb|CR748429 Soares_testis_NHT Homo sapiens cDNA clone IMAGp971I0459 ; IMAGE:1394786 5', mRNA sequence [CR748429] |
| BU963192 | BU963192 | gb|AGENCOURT_10615922 NIH_MGC_141 Homo sapiens cDNA clone IMAGE:6744194 5', mRNA sequence [BU963192] |
| PNPT1 | NM_033109 | polyribonucleotide nucleotidyltransferase 1 (PNPT1), mRNA [NM_033109] |
| RASGRP3 | NM_001139488 | RAS guanyl releasing protein 3 (calcium and DAG-regulated) (RASGRP3), transcript variant 1, mRNA [NM_001139488] |
| XLOC_008433 | TCONS_00018702 | linc|BROAD Institute lincRNA (XLOC_008433), lincRNA [TCONS_00018702] |
| ACSL3 | NM_004457 | acyl-CoA synthetase long-chain family member 3 (ACSL3), transcript variant 1, mRNA [NM_004457] |
| LOC400654 | NR_033983 | uncharacterized LOC400654 (LOC400654), non-coding RNA [NR_033983] |
| CEP76 | NM_024899 | centrosomal protein 76kDa (CEP76), mRNA [NM_024899] |
| C14orf165 | NR_024081 | chromosome 14 open reading frame 165 (C14orf165), non-coding RNA [NR_024081] |
| PLEK | NM_002664 | pleckstrin (PLEK), mRNA [NM_002664] |
| OBSL1 | NM_001173408 | obscurin-like 1 (OBSL1), transcript variant 3, mRNA [NM_001173408] |
| SPINT4 | NM_178455 | serine peptidase inhibitor, Kunitz type 4 (SPINT4), mRNA [NM_178455] |
| CAPRIN1 | NM_005898 | cell cycle associated protein 1 (CAPRIN1), transcript variant 1, mRNA [NM_005898] |
| PCLO | NM_033026 | piccolo (presynaptic cytomatrix protein) (PCLO), transcript variant 1, mRNA [NM_033026] |
| XLOC_011856 | TCONS_00024546 | linc|BROAD Institute lincRNA (XLOC_011856), lincRNA [TCONS_00024546] |
| XLOC_005616 | ENST00000456440 | linc|BROAD Institute lincRNA (XLOC_005616), lincRNA [TCONS_00011457] |
| XLOC_000555 | TCONS_00001267 | linc|BROAD Institute lincRNA (XLOC_000555), lincRNA [TCONS_00001267] |
| PTPRN2 | NM_002847 | protein tyrosine phosphatase, receptor type, N polypeptide 2 (PTPRN2), transcript variant 1, mRNA [NM_002847] |
| XLOC_004593 | ENST00000512571 | gb|BX109342 Soares_NFL_T_GBC_S1 Homo sapiens cDNA clone IMAGp998D224004, mRNA sequence [BX109342] |
| LOC284276 | NR_015417 | uncharacterized LOC284276 (LOC284276), non-coding RNA [NR_015417] |
| XLOC_001824 | ENST00000435643 | gb|DB028315 TESTI2 Homo sapiens cDNA clone TESTI2011177 5', mRNA sequence [DB028315] |
| C6orf147 | NR_027005 | chromosome 6 open reading frame 147 (C6orf147), non-coding RNA [NR_027005] |
| SLC35E4 | ENST00000300385 | ens|solute carrier family 35, member E4 [Source:HGNC Symbol;Acc:17058] [ENST00000300385] |
| ZNF890P | NR_034163 | zinc finger protein 890, pseudogene (ZNF890P), non-coding RNA [NR_034163] |
| SETBP1 | NM_001130110 | SET binding protein 1 (SETBP1), transcript variant 2, mRNA [NM_001130110] |
| SLC15A4 | NM_145648 | solute carrier family 15, member 4 (SLC15A4), mRNA [NM_145648] |
| XLOC_003991 | TCONS_00009076 | linc|BROAD Institute lincRNA (XLOC_003991), lincRNA [TCONS_00009076] |
| XLOC_010679 | THC2672811 | linc|BROAD Institute lincRNA (XLOC_010679), lincRNA [TCONS_00022076] |
| CAMK2N1 | NM_018584 | calcium/calmodulin-dependent protein kinase II inhibitor 1 (CAMK2N1), mRNA [NM_018584] |
| TMIGD1 | NM_206832 | transmembrane and immunoglobulin domain containing 1 (TMIGD1), mRNA [NM_206832] |
| LAMB4 | NM_007356 | laminin, beta 4 (LAMB4), mRNA [NM_007356] |
| SLC33A1 | NM_001190992 | solute carrier family 33 (acetyl-CoA transporter), member 1 (SLC33A1), transcript variant 2, mRNA [NM_001190992] |
| XLOC_011565 | TCONS_00024199 | linc|BROAD Institute lincRNA (XLOC_011565), lincRNA [TCONS_00024199] |
| XLOC_014104 | TCONS_00029160 | linc|BROAD Institute lincRNA (XLOC_014104), lincRNA [TCONS_00029160] |
| NEDD9 | NM_006403 | neural precursor cell expressed, developmentally down-regulated 9 (NEDD9), transcript variant 1, mRNA [NM_006403] |
| TP53AIP1 | NM_001195195 | tumor protein p53 regulated apoptosis inducing protein 1 (TP53AIP1), nuclear gene encoding mitochondrial protein, transcript variant 2, mRNA [NM_001195195] |
| ENST00000451368 | ENST00000451368 | Unknown |
| A_33_P3363959 | A_33_P3363959 | Unknown |
| LINC00222 | NR_033376 | long intergenic non-protein coding RNA 222 (LINC00222), non-coding RNA [NR_033376] |
| GNRHR2 | NR_002328 | gonadotropin-releasing hormone (type 2) receptor 2 (GNRHR2), non-coding RNA [NR_002328] |
| MB21D2 | NM_178496 | Mab-21 domain containing 2 (MB21D2), mRNA [NM_178496] |
| XLOC_l2_009332 | TCONS_l2_00017669 | linc|BROAD Institute lincRNA (XLOC_l2_009332), lincRNA [TCONS_l2_00017669] |
| APOBEC4 | NM_203454 | apolipoprotein B mRNA editing enzyme, catalytic polypeptide-like 4 (putative) (APOBEC4), mRNA [NM_203454] |
| LOC646903 | NR_036538 | uncharacterized LOC646903 (LOC646903), non-coding RNA [NR_036538] |
| LOC100130357 | NM_001242698 | uncharacterized LOC100130357 (LOC100130357), mRNA [NM_001242698] |
| ASH2L | NM_004674 | ash2 (absent, small, or homeotic)-like (Drosophila) (ASH2L), transcript variant 1, mRNA [NM_004674] |
| XPNPEP3 | NM_001204827 | X-prolyl aminopeptidase (aminopeptidase P) 3, putative (XPNPEP3), transcript variant 2, mRNA [NM_001204827] |
| SLC22A4 | NM_003059 | solute carrier family 22 (organic cation/ergothioneine transporter), member 4 (SLC22A4), mRNA [NM_003059] |
| XLOC_001869 | TCONS_00004031 | linc|BROAD Institute lincRNA (XLOC_001869), lincRNA [TCONS_00004031] |
| TIGD2 | NM_145715 | tigger transposable element derived 2 (TIGD2), mRNA [NM_145715] |
| LOC90834 | NR_026993 | uncharacterized protein BC001742 (LOC90834), non-coding RNA [NR_026993] |
| NOP56 | NM_006392 | NOP56 ribonucleoprotein homolog (yeast) (NOP56), transcript variant 1, mRNA [NM_006392] |
| XLOC_007101 | ENST00000521061 | linc|BROAD Institute lincRNA (XLOC_007101), lincRNA [TCONS_00015024] |
| XLOC_004272 | ENST00000506093 | linc|BROAD Institute lincRNA (XLOC_004272), lincRNA [TCONS_00009339] |
| DB221055 | DB221055 | gb|DB221055 TRACH3 Homo sapiens cDNA clone TRACH3011617 5', mRNA sequence [DB221055] |
| XLOC_001002 | THC2591706 | tc|HUMFMO5A flavin-containing monooxygenase 5 {Homo sapiens} (exp=-1; wgp=0; cg=0), partial (20%) [THC2508527] |
| GRIK1 | NM_000830 | glutamate receptor, ionotropic, kainate 1 (GRIK1), transcript variant 1, mRNA [NM_000830] |
| C5orf41 | NM_153607 | chromosome 5 open reading frame 41 (C5orf41), transcript variant 1, mRNA [NM_153607] |
| USP1 | NM_003368 | ubiquitin specific peptidase 1 (USP1), transcript variant 1, mRNA [NM_003368] |
| RNASE12 | NM_001024822 | ribonuclease, RNase A family, 12 (non-active) (RNASE12), mRNA [NM_001024822] |
| C7orf33 | NM_145304 | chromosome 7 open reading frame 33 (C7orf33), mRNA [NM_145304] |
| XLOC_010507 | BX649107 | linc|BROAD Institute lincRNA (XLOC_010507), lincRNA [TCONS_00021603] |
| XLOC_000627 | ENST00000423963 | gb|RST8688 Athersys RAGE Library Homo sapiens cDNA, mRNA sequence [BG189643] |
| LIMCH1 | NM_014988 | LIM and calponin homology domains 1 (LIMCH1), transcript variant 1, mRNA [NM_014988] |
| MCM7 | NM_005916 | minichromosome maintenance complex component 7 (MCM7), transcript variant 1, mRNA [NM_005916] |
| PLEKHM3 | NM_001080475 | pleckstrin homology domain containing, family M, member 3 (PLEKHM3), mRNA [NM_001080475] |
| C11orf1 | ENST00000260276 | ens|chromosome 11 open reading frame 1 [Source:HGNC Symbol;Acc:1163] [ENST00000260276] |
| XLOC_000830 | TCONS_00001490 | linc|BROAD Institute lincRNA (XLOC_000830), lincRNA [TCONS_00001490] |
| CPA1 | NM_001868 | carboxypeptidase A1 (pancreatic) (CPA1), mRNA [NM_001868] |
| DHX29 | NM_019030 | DEAH (Asp-Glu-Ala-His) box polypeptide 29 (DHX29), mRNA [NM_019030] |
| XLOC_010544 | TCONS_00022239 | linc|BROAD Institute lincRNA (XLOC_010544), lincRNA [TCONS_00022239] |
| XLOC_008977 | ENST00000415509 | linc|BROAD Institute lincRNA (XLOC_008977), lincRNA [TCONS_00018062] |
| XLOC_000090 | TCONS_00000863 | linc|BROAD Institute lincRNA (XLOC_000090), lincRNA [TCONS_00000863] |
| EIF2AK4 | NM_001013703 | eukaryotic translation initiation factor 2 alpha kinase 4 (EIF2AK4), mRNA [NM_001013703] |
| OR8H1 | NM_001005199 | olfactory receptor, family 8, subfamily H, member 1 (OR8H1), mRNA [NM_001005199] |
| PFN1P2 | NR_003242 | profilin 1 pseudogene 2 (PFN1P2), non-coding RNA [NR_003242] |
| XLOC_013309 | TCONS_00027311 | linc|BROAD Institute lincRNA (XLOC_013309), lincRNA [TCONS_00027311] |
| KCNE3 | NM_005472 | potassium voltage-gated channel, Isk-related family, member 3 (KCNE3), mRNA [NM_005472] |
| UBE2D3 | ENST00000503418 | ens|ubiquitin-conjugating enzyme E2D 3 [Source:HGNC Symbol;Acc:12476] [ENST00000503418] |
| THC2569912 | THC2569912 | tc|CG024_HUMAN (O75223) Protein C7orf24, partial (73%) [THC2569912] |
| XLOC_012616 | TCONS_00026260 | linc|BROAD Institute lincRNA (XLOC_012616), lincRNA [TCONS_00026260] |
| XLOC_009404 | TCONS_00019602 | linc|BROAD Institute lincRNA (XLOC_009404), lincRNA [TCONS_00019602] |
| LOC100505841 | NM_001195535 | zinc finger protein 474-like (LOC100505841), mRNA [NM_001195535] |
| CEACAM1 | NM_001712 | carcinoembryonic antigen-related cell adhesion molecule 1 (biliary glycoprotein) (CEACAM1), transcript variant 1, mRNA [NM_001712] |
| SLC20A2 | ENST00000523340 | ens|solute carrier family 20 (phosphate transporter), member 2 [Source:HGNC Symbol;Acc:10947] [ENST00000523340] |
| ZNF710 | NM_198526 | zinc finger protein 710 (ZNF710), mRNA [NM_198526] |
| ARHGEF40 | NM_018071 | Rho guanine nucleotide exchange factor (GEF) 40 (ARHGEF40), mRNA [NM_018071] |
| XLOC_010979 | TCONS_00022688 | linc|BROAD Institute lincRNA (XLOC_010979), lincRNA [TCONS_00022688] |
| XLOC_000757 | TCONS_00001441 | linc|BROAD Institute lincRNA (XLOC_000757), lincRNA [TCONS_00001441] |
| LOC100507332 | XR_108981 | ref|PREDICTED: Homo sapiens hypothetical LOC100507332 (LOC100507332), miscRNA [XR_108981] |
| XLOC_l2_010831 | AK093416 | tc|Q73P46_TREDE (Q73P46) Branched-chain amino acid ABC transporter, permease protein, partial (5%) [THC2614189] |
| A_33_P3255359 | A_33_P3255359 | Unknown |
| SMCR7 | ENST00000395703 | ens|Smith-Magenis syndrome chromosome region, candidate 7 [Source:HGNC Symbol;Acc:17920] [ENST00000395703] |
| XLOC_010123 | BC042465 | linc|BROAD Institute lincRNA (XLOC_010123), lincRNA [TCONS_00021357] |
| ZNF845 | NM_138374 | zinc finger protein 845 (ZNF845), mRNA [NM_138374] |
| LOC100128191 | NR_027157 | uncharacterized LOC100128191 (LOC100128191), non-coding RNA [NR_027157] |
| A_33_P3275065 | A_33_P3275065 | Unknown |
| XLOC_012020 | TCONS_00024740 | linc|BROAD Institute lincRNA (XLOC_012020), lincRNA [TCONS_00024740] |
| ERMN | NM_020711 | ermin, ERM-like protein (ERMN), transcript variant 2, mRNA [NM_020711] |
| XLOC_002618 | TCONS_00006817 | linc|BROAD Institute lincRNA (XLOC_002618), lincRNA [TCONS_00006817] |
| ZCCHC18 | NM_001143978 | zinc finger, CCHC domain containing 18 (ZCCHC18), transcript variant 1, mRNA [NM_001143978] |
| XLOC_013045 | AK125858 | linc|BROAD Institute lincRNA (XLOC_013045), lincRNA [TCONS_00026786] |
| AFMID | NR_027083 | arylformamidase (AFMID), transcript variant 3, non-coding RNA [NR_027083] |
| CRB2 | ENST00000359999 | ens|crumbs homolog 2 (Drosophila) [Source:HGNC Symbol;Acc:18688] [ENST00000359999] |
| GCKR | NM_001486 | glucokinase (hexokinase 4) regulator (GCKR), mRNA [NM_001486] |
| PRLR | NM_000949 | prolactin receptor (PRLR), transcript variant 1, mRNA [NM_000949] |
| GAL3ST1 | NM_004861 | galactose-3-O-sulfotransferase 1 (GAL3ST1), mRNA [NM_004861] |
| QRSL1 | NM_018292 | glutaminyl-tRNA synthase (glutamine-hydrolyzing)-like 1 (QRSL1), mRNA [NM_018292] |
| CCDC151 | NM_145045 | coiled-coil domain containing 151 (CCDC151), mRNA [NM_145045] |
| LOC100128675 | NR_024561 | uncharacterized LOC100128675 (LOC100128675), transcript variant 1, non-coding RNA [NR_024561] |
| XLOC_004325 | AK098570 | linc|BROAD Institute lincRNA (XLOC_004325), lincRNA [TCONS_00010650] |
| SORBS1 | NM_001034954 | sorbin and SH3 domain containing 1 (SORBS1), transcript variant 3, mRNA [NM_001034954] |
| OSBPL6 | NM_032523 | oxysterol binding protein-like 6 (OSBPL6), transcript variant 1, mRNA [NM_032523] |
| ATPAF1-AS1 | NM_001145474 | ATPAF1 antisense RNA 1 (non-protein coding) (ATPAF1-AS1), mRNA [NM_001145474] |
| CALCR | NM_001742 | calcitonin receptor (CALCR), transcript variant 2, mRNA [NM_001742] |
| LOC100129345 | NR_033943 | uncharacterized LOC100129345 (LOC100129345), non-coding RNA [NR_033943] |
| XLOC_008168 | ENST00000448761 | linc|BROAD Institute lincRNA (XLOC_008168), lincRNA [TCONS_00017060] |
| LOR | NM_000427 | loricrin (LOR), mRNA [NM_000427] |
| XLOC_009456 | TCONS_00019651 | linc|BROAD Institute lincRNA (XLOC_009456), lincRNA [TCONS_00019651] |
| XLOC_011008 | TCONS_00022730 | linc|BROAD Institute lincRNA (XLOC_011008), lincRNA [TCONS_00022730] |
| S1PR5 | NM_030760 | sphingosine-1-phosphate receptor 5 (S1PR5), transcript variant 1, mRNA [NM_030760] |
| MCTP1 | NM_024717 | multiple C2 domains, transmembrane 1 (MCTP1), transcript variant L, mRNA [NM_024717] |
| FAM166A | NM_001001710 | family with sequence similarity 166, member A (FAM166A), mRNA [NM_001001710] |
| FLJ12334 | AK022396 | tc|ALU7_HUMAN (P39194) Alu subfamily SQ sequence contamination warning entry, partial (9%) [THC2497478] |
| B3GALT1 | NM_020981 | UDP-Gal:betaGlcNAc beta 1,3-galactosyltransferase, polypeptide 1 (B3GALT1), mRNA [NM_020981] |
| MAST4 | NM_001164664 | microtubule associated serine/threonine kinase family member 4 (MAST4), transcript variant 3, mRNA [NM_001164664] |
| CKAP2L | NM_152515 | cytoskeleton associated protein 2-like (CKAP2L), mRNA [NM_152515] |
| LOC338651 | NR_021489 | uncharacterized LOC338651 (LOC338651), non-coding RNA [NR_021489] |
| OR51I1 | NM_001005288 | olfactory receptor, family 51, subfamily I, member 1 (OR51I1), mRNA [NM_001005288] |
| KIAA1024L | XM_001721301 | gb|PREDICTED: Homo sapiens KIAA1024-like (KIAA1024L), mRNA [XM_001721301] |
| CCRL2 | NM_003965 | chemokine (C-C motif) receptor-like 2 (CCRL2), transcript variant 1, mRNA [NM_003965] |
| LOC100289251 | XR_110280 | ref|PREDICTED: Homo sapiens hypothetical LOC100289251 (LOC100289251), miscRNA [XR_110280] |
| LOC100506733 | NR_038973 | uncharacterized LOC100506733 (LOC100506733), non-coding RNA [NR_038973] |
| LOC100509247 | XM_003120038 | ref|PREDICTED: Homo sapiens hypothetical protein LOC100509247 (LOC100509247), mRNA [XM_003120038] |
| COG3 | NM_031431 | component of oligomeric golgi complex 3 (COG3), mRNA [NM_031431] |
| XLOC_l2_008396 | ENST00000432711 | gb|DB550107 RIKEN full-length enriched human cDNA library, hippocampus Homo sapiens cDNA clone H023089H16 3', mRNA sequence [DB550107] |
| PRO2214 | AF119867 | gb|Homo sapiens PRO2214 mRNA, complete cds. [AF119867] |
| A_33_P3223059 | A_33_P3223059 | Unknown |
| LOC100507233 | XR_109824 | ref|PREDICTED: Homo sapiens hypothetical LOC100507233 (LOC100507233), miscRNA [XR_109824] |
| LOC283624 | NR_038970 | uncharacterized LOC283624 (LOC283624), transcript variant 1, non-coding RNA [NR_038970] |
| XLOC_002878 | TCONS_00007008 | linc|BROAD Institute lincRNA (XLOC_002878), lincRNA [TCONS_00007008] |
| XLOC_005921 | TCONS_00012361 | linc|BROAD Institute lincRNA (XLOC_005921), lincRNA [TCONS_00012361] |
| NR6A1 | ENST00000487099 | ens|nuclear receptor subfamily 6, group A, member 1 [Source:HGNC Symbol;Acc:7985] [ENST00000487099] |
| XLOC_011470 | TCONS_00023671 | linc|BROAD Institute lincRNA (XLOC_011470), lincRNA [TCONS_00023671] |
| XLOC_001607 | TCONS_00003808 | linc|BROAD Institute lincRNA (XLOC_001607), lincRNA [TCONS_00003808] |
| HSPB11 | ENST00000371377 | ens|heat shock protein family B (small), member 11 [Source:HGNC Symbol;Acc:25019] [ENST00000371377] |
| LOC100507209 | XR_132691 | ref|PREDICTED: Homo sapiens hypothetical LOC100507209 (LOC100507209), miscRNA [XR_132691] |
| XLOC_001964 | ENST00000450467 | ref|PREDICTED: Homo sapiens hypothetical LOC100506216 (LOC100506216), miscRNA [XR_108384] |
| SPRY2 | NM_005842 | sprouty homolog 2 (Drosophila) (SPRY2), mRNA [NM_005842] |
| PLEKHA3 | NM_019091 | pleckstrin homology domain containing, family A (phosphoinositide binding specific) member 3 (PLEKHA3), mRNA [NM_019091] |
| OR6C68 | NM_001005519 | olfactory receptor, family 6, subfamily C, member 68 (OR6C68), mRNA [NM_001005519] |
| GHITM | NM_014394 | growth hormone inducible transmembrane protein (GHITM), mRNA [NM_014394] |
| XLOC_008729 | ENST00000435106 | linc|BROAD Institute lincRNA (XLOC_008729), lincRNA [TCONS_00017926] |
| A_33_P3229958 | A_33_P3229958 | Unknown |
| XLOC_007962 | ENST00000440955 | tc|Q5FWS0_XENTR (Q5FWS0) MGC107780 protein, partial (7%) [THC2631358] |
| XLOC_004262 | TCONS_00009850 | linc|BROAD Institute lincRNA (XLOC_004262), lincRNA [TCONS_00009850] |
| ARPP21 | NM_001025069 | cAMP-regulated phosphoprotein, 21kDa (ARPP21), transcript variant 4, mRNA [NM_001025069] |
| XLOC_003194 | TCONS_00006567 | linc|BROAD Institute lincRNA (XLOC_003194), lincRNA [TCONS_00006567] |
| NUDT16 | NM_001171906 | nudix (nucleoside diphosphate linked moiety X)-type motif 16 (NUDT16), transcript variant 1, mRNA [NM_001171906] |
| XLOC_008245 | TCONS_00017380 | linc|BROAD Institute lincRNA (XLOC_008245), lincRNA [TCONS_00017380] |
| ORC5 | NM_002553 | origin recognition complex, subunit 5 (ORC5), transcript variant 1, mRNA [NM_002553] |
| XLOC_l2_015946 | ENST00000505047 | tc|AF332226 heat shock transcription factor 2-like protein {Homo sapiens} (exp=-1; wgp=0; cg=0), partial (44%) [THC2643044] |
| PTHLH | NM_198965 | parathyroid hormone-like hormone (PTHLH), transcript variant 1, mRNA [NM_198965] |
| XLOC_008424 | AK058127 | tc|GB|AK058127.1|AK058127.1 Homo sapiens cDNA FLJ25398 fis, clone TST02653 [NP1154375] |
| A_33_P3318069 | A_33_P3318069 | Unknown |
| UBE4B | ENST00000377153 | ens|ubiquitination factor E4B [Source:HGNC Symbol;Acc:12500] [ENST00000377153] |
| LOC550643 | BC048131 | Unknown |
| MTFR1 | NM_014637 | mitochondrial fission regulator 1 (MTFR1), nuclear gene encoding mitochondrial protein, transcript variant 1, mRNA [NM_014637] |
| XLOC_l2_005179 | TCONS_l2_00009674 | linc|BROAD Institute lincRNA (XLOC_l2_005179), lincRNA [TCONS_l2_00009674] |
| XLOC_013243 | TCONS_00027203 | linc|BROAD Institute lincRNA (XLOC_013243), lincRNA [TCONS_00027203] |
| PDSS2 | NM_020381 | prenyl (decaprenyl) diphosphate synthase, subunit 2 (PDSS2), mRNA [NM_020381] |
| APOB | NM_000384 | apolipoprotein B (including Ag(x) antigen) (APOB), mRNA [NM_000384] |
| XLOC_000269 | ENST00000419658 | linc|BROAD Institute lincRNA (XLOC_000269), lincRNA [TCONS_00000243] |
| LOC286272 | AK093004 | gb|Homo sapiens cDNA FLJ35685 fis, clone SPLEN2019257. [AK093004] |
| RFTN2 | NM_144629 | raftlin family member 2 (RFTN2), mRNA [NM_144629] |
| XLOC_004641 | TCONS_00010179 | linc|BROAD Institute lincRNA (XLOC_004641), lincRNA [TCONS_00010179] |
| CEACAM8 | NM_001816 | carcinoembryonic antigen-related cell adhesion molecule 8 (CEACAM8), mRNA [NM_001816] |
| TLR3 | NM_003265 | toll-like receptor 3 (TLR3), mRNA [NM_003265] |
| CHD1 | NM_001270 | chromodomain helicase DNA binding protein 1 (CHD1), mRNA [NM_001270] |
| XLOC_012623 | TCONS_00026276 | linc|BROAD Institute lincRNA (XLOC_012623), lincRNA [TCONS_00026276] |
| METTL15 | NM_152636 | methyltransferase like 15 (METTL15), transcript variant 2, mRNA [NM_152636] |
| XLOC_001945 | ENST00000457813 | linc|BROAD Institute lincRNA (XLOC_001945), lincRNA [TCONS_00003152] |
| LOC554206 | NR_038379 | leucine carboxyl methyltransferase 1 pseudogene (LOC554206), non-coding RNA [NR_038379] |
| ENST00000469965 | ENST00000469965 | gb|DA760426 NT2RP8 Homo sapiens cDNA clone NT2RP8009290 5', mRNA sequence [DA760426] |
| JKAMP | NM_016475 | JNK1/MAPK8-associated membrane protein (JKAMP), transcript variant 1, mRNA [NM_016475] |
| LATS2 | NM_014572 | LATS, large tumor suppressor, homolog 2 (Drosophila) (LATS2), mRNA [NM_014572] |
| XLOC_013015 | TCONS_00026959 | linc|BROAD Institute lincRNA (XLOC_013015), lincRNA [TCONS_00026959] |
| RBMY1B | NM_001006121 | RNA binding motif protein, Y-linked, family 1, member B (RBMY1B), mRNA [NM_001006121] |
| MICAL2 | NM_014632 | microtubule associated monoxygenase, calponin and LIM domain containing 2 (MICAL2), mRNA [NM_014632] |
| VN1R1 | NM_020633 | vomeronasal 1 receptor 1 (VN1R1), mRNA [NM_020633] |
| MICAL3 | NM_001136004 | microtubule associated monoxygenase, calponin and LIM domain containing 3 (MICAL3), transcript variant 2, mRNA [NM_001136004] |
| C1orf138 | AK127688 | gb|Homo sapiens cDNA FLJ45786 fis, clone NETRP2008488. [AK127688] |
| NCRNA00083 | ENST00000445098 | ref|PREDICTED: Homo sapiens non-protein coding RNA 83 (NCRNA00083), miscRNA [XR_108314] |
| ZNF816-ZNF321P | NM_001202473 | ZNF816-ZNF321P readthrough (ZNF816-ZNF321P), mRNA [NM_001202473] |
| FAM19A3 | NM_001004440 | family with sequence similarity 19 (chemokine (C-C motif)-like), member A3 (FAM19A3), transcript variant 2, mRNA [NM_001004440] |
| LRRC66 | NM_001024611 | leucine rich repeat containing 66 (LRRC66), mRNA [NM_001024611] |
| XLOC_000032 | TCONS_00000823 | linc|BROAD Institute lincRNA (XLOC_000032), lincRNA [TCONS_00000823] |
| DUSP12 | NM_007240 | dual specificity phosphatase 12 (DUSP12), mRNA [NM_007240] |
| XLOC_000340 | TCONS_00001094 | linc|BROAD Institute lincRNA (XLOC_000340), lincRNA [TCONS_00001094] |
| THC2641208 | THC2641208 | tc|Q6QMY6_RAT (Q6QMY6) Hepatic protein EIIH, partial (11%) [THC2641208] |
| XLOC_l2_007566 | BX098119 | gb|BX098119 Soares placenta Nb2HP Homo sapiens cDNA clone IMAGp998I02228, mRNA sequence [BX098119] |
| A_33_P3296696 | A_33_P3296696 | Unknown |
| AK124190 | AK124190 | gb|Homo sapiens cDNA FLJ42196 fis, clone THYMU2033816. [AK124190] |
| MLK7-AS1 | NR_033882 | MLK7 antisense RNA 1 (non-protein coding) (MLK7-AS1), non-coding RNA [NR_033882] |
| XLOC_010112 | ENST00000543387 | gb|603071124F1 NIH_MGC_119 Homo sapiens cDNA clone IMAGE:5163484 5', mRNA sequence [BI520265] |
| EGFR | NM_005228 | epidermal growth factor receptor (EGFR), transcript variant 1, mRNA [NM_005228] |
| TMEM44 | NM_001011655 | transmembrane protein 44 (TMEM44), transcript variant 2, mRNA [NM_001011655] |
| AURKC | NM_001015878 | aurora kinase C (AURKC), transcript variant 1, mRNA [NM_001015878] |
| SNORD114-26 | NR_003219 | small nucleolar RNA, C/D box 114-26 (SNORD114-26), small nucleolar RNA [NR_003219] |
| DQ786249 | DQ786249 | gb|Homo sapiens clone HLS_IMAGE_234376 mRNA sequence. [DQ786249] |
| LOC285191 | AK096937 | linc|BROAD Institute lincRNA (XLOC_001920), lincRNA [TCONS_00004072] |
| KLF9 | NM_001206 | Kruppel-like factor 9 (KLF9), mRNA [NM_001206] |
| XLOC_000371 | AI678218 | gb|wc24f11.x1 NCI_CGAP_Pr28 Homo sapiens cDNA clone IMAGE:2316141 3', mRNA sequence [AI678218] |
| LOC641365 | NR_037866 | uncharacterized LOC641365 (LOC641365), non-coding RNA [NR_037866] |
| PLCXD3 | NM_001005473 | phosphatidylinositol-specific phospholipase C, X domain containing 3 (PLCXD3), mRNA [NM_001005473] |
| XLOC_l2_004318 | BI821606 | gb|603036639F1 NIH_MGC_115 Homo sapiens cDNA clone IMAGE:5177672 5', mRNA sequence [BI821606] |
| LOC100508177 | XR_111763 | ref|PREDICTED: Homo sapiens hypothetical LOC100508177 (LOC100508177), miscRNA [XR_111763] |
| TRIM61 | NM_001012414 | tripartite motif containing 61 (TRIM61), mRNA [NM_001012414] |
| FAM75D4 | NM_001145197 | family with sequence similarity 75, member D4 (FAM75D4), mRNA [NM_001145197] |
| F3 | NM_001993 | coagulation factor III (thromboplastin, tissue factor) (F3), transcript variant 1, mRNA [NM_001993] |
| LOC100128682 | NR_040046 | uncharacterized LOC100128682 (LOC100128682), non-coding RNA [NR_040046] |
| XLOC_005012 | THC2785600 | linc|BROAD Institute lincRNA (XLOC_005012), lincRNA [TCONS_00010493] |
| XLOC_006198 | TCONS_00014189 | linc|BROAD Institute lincRNA (XLOC_006198), lincRNA [TCONS_00014189] |
| KIAA0196 | NM_014846 | KIAA0196 (KIAA0196), mRNA [NM_014846] |
| LOC280665 | AF547222 | gb|Homo sapiens putative anti-CNG alpha 1 cation channel translation product mRNA, complete cds. [AF547222] |
| POLR2B | NM_000938 | polymerase (RNA) II (DNA directed) polypeptide B, 140kDa (POLR2B), mRNA [NM_000938] |
| EIF5A2 | NM_020390 | eukaryotic translation initiation factor 5A2 (EIF5A2), mRNA [NM_020390] |
| MAPKAPK5 | NM_139078 | mitogen-activated protein kinase-activated protein kinase 5 (MAPKAPK5), transcript variant 2, mRNA [NM_139078] |
| XLOC_001823 | TCONS_00003999 | linc|BROAD Institute lincRNA (XLOC_001823), lincRNA [TCONS_00003999] |
| LMO4 | NM_006769 | LIM domain only 4 (LMO4), mRNA [NM_006769] |
| A_33_P3404360 | A_33_P3404360 | Unknown |
| LOC100507094 | XR_109558 | ref|PREDICTED: Homo sapiens hypothetical LOC100507094 (LOC100507094), miscRNA [XR_109558] |
| XLOC_000494 | TCONS_00001221 | linc|BROAD Institute lincRNA (XLOC_000494), lincRNA [TCONS_00001221] |
| OSTBETA | NM_178859 | organic solute transporter beta (OSTBETA), mRNA [NM_178859] |
| ITGA2 | NM_002203 | integrin, alpha 2 (CD49B, alpha 2 subunit of VLA-2 receptor) (ITGA2), mRNA [NM_002203] |
| APTX | NM_001195249 | aprataxin (APTX), transcript variant 7, mRNA [NM_001195249] |
| FAM123C | NM_152698 | family with sequence similarity 123C (FAM123C), transcript variant 1, mRNA [NM_152698] |
| ANKRD35 | NM_144698 | ankyrin repeat domain 35 (ANKRD35), mRNA [NM_144698] |
| RIN2 | NM_018993 | Ras and Rab interactor 2 (RIN2), transcript variant 2, mRNA [NM_018993] |
| AVL9 | NM_015060 | AVL9 homolog (S. cerevisiase) (AVL9), mRNA [NM_015060] |
| XLOC_013290 | TCONS_00027288 | linc|BROAD Institute lincRNA (XLOC_013290), lincRNA [TCONS_00027288] |
| XLOC_002223 | ENST00000414965 | linc|BROAD Institute lincRNA (XLOC_002223), lincRNA [TCONS_00003340] |
| SUZ12 | NM_015355 | suppressor of zeste 12 homolog (Drosophila) (SUZ12), mRNA [NM_015355] |
| MMRN1 | NM_007351 | multimerin 1 (MMRN1), mRNA [NM_007351] |
| DOK2 | NM_003974 | docking protein 2, 56kDa (DOK2), mRNA [NM_003974] |
| XLOC_l2_010061 | ENST00000415451 | gb|BX111391 Soares_testis_NHT Homo sapiens cDNA clone IMAGp998D244108, mRNA sequence [BX111391] |
| DCLRE1B | NM_022836 | DNA cross-link repair 1B (DCLRE1B), mRNA [NM_022836] |
| XLOC_007746 | BC042427 | tc|Q8DGI1_SYNEL (Q8DGI1) Tll2336 protein, partial (3%) [THC2606440] |
| XLOC_011074 | TCONS_00023132 | linc|BROAD Institute lincRNA (XLOC_011074), lincRNA [TCONS_00023132] |
| XLOC_002255 | ENST00000424355 | linc|BROAD Institute lincRNA (XLOC_002255), lincRNA [TCONS_00003364] |
| RND3 | NM_005168 | Rho family GTPase 3 (RND3), mRNA [NM_005168] |
| MGC39372 | NR_033851 | serpin peptidase inhibitor, clade B (ovalbumin), member 9 pseudogene (MGC39372), non-coding RNA [NR_033851] |
| DCAF10 | NM_024345 | DDB1 and CUL4 associated factor 10 (DCAF10), mRNA [NM_024345] |
| HORMAD1 | NM_032132 | HORMA domain containing 1 (HORMAD1), transcript variant 1, mRNA [NM_032132] |
| XLOC_002334 | TCONS_00004459 | linc|BROAD Institute lincRNA (XLOC_002334), lincRNA [TCONS_00004459] |
| LOC100131702 | AK126983 | gb|Homo sapiens cDNA FLJ45037 fis, clone BRAWH3019820. [AK126983] |
| FOXN4 | NM_213596 | forkhead box N4 (FOXN4), mRNA [NM_213596] |
| NFKBIB | NR_040515 | nuclear factor of kappa light polypeptide gene enhancer in B-cells inhibitor, beta (NFKBIB), transcript variant 3, non-coding RNA [NR_040515] |
| PTAFR | NM_000952 | platelet-activating factor receptor (PTAFR), transcript variant 3, mRNA [NM_000952] |
| XLOC_010148 | DA233116 | gb|DA233116 BRAWH3 Homo sapiens cDNA clone BRAWH3028924 5', mRNA sequence [DA233116] |
| MMP21 | NM_147191 | matrix metallopeptidase 21 (MMP21), mRNA [NM_147191] |
| AARSD1 | NM_025267 | alanyl-tRNA synthetase domain containing 1 (AARSD1), transcript variant 2, mRNA [NM_025267] |
| PHC1 | NM_004426 | polyhomeotic homolog 1 (Drosophila) (PHC1), mRNA [NM_004426] |
| XLOC_014097 | ENST00000431150 | gb|17000600332949 GRN_PRENEU Homo sapiens cDNA 5', mRNA sequence [CN278680] |
| GUCA1B | NM_002098 | guanylate cyclase activator 1B (retina) (GUCA1B), mRNA [NM_002098] |
| AF370399 | AF370399 | gb|Homo sapiens PP10881 mRNA, complete cds. [AF370399] |
| OR51B2 | NM_033180 | olfactory receptor, family 51, subfamily B, member 2 (OR51B2), mRNA [NM_033180] |
| ZNF3 | NM_017715 | zinc finger protein 3 (ZNF3), transcript variant 1, mRNA [NM_017715] |
| BEST1 | NM_004183 | bestrophin 1 (BEST1), transcript variant 1, mRNA [NM_004183] |
| XLOC_l2_013868 | TCONS_l2_00026623 | linc|BROAD Institute lincRNA (XLOC_l2_013868), lincRNA [TCONS_l2_00026623] |
| XLOC_002587 | TCONS_00005953 | linc|BROAD Institute lincRNA (XLOC_002587), lincRNA [TCONS_00005953] |
| LOC116437 | NR_026670 | uncharacterized LOC116437 (LOC116437), non-coding RNA [NR_026670] |
| LOC100129269 | NR_034126 | uncharacterized LOC100129269 (LOC100129269), non-coding RNA [NR_034126] |
| CXCL13 | NM_006419 | chemokine (C-X-C motif) ligand 13 (CXCL13), mRNA [NM_006419] |
| OR4A16 | NM_001005274 | olfactory receptor, family 4, subfamily A, member 16 (OR4A16), mRNA [NM_001005274] |
| PWRN2 | NR_026647 | Prader-Willi region non-protein coding RNA 2 (PWRN2), non-coding RNA [NR_026647] |
| SRP68 | NM_014230 | signal recognition particle 68kDa (SRP68), mRNA [NM_014230] |
| ENST00000451884 | ENST00000451884 | Unknown |
| CASP3 | NM_004346 | caspase 3, apoptosis-related cysteine peptidase (CASP3), transcript variant alpha, mRNA [NM_004346] |
| XLOC_l2_011969 | ENST00000511029 | linc|BROAD Institute lincRNA (XLOC_l2_011969), lincRNA [TCONS_l2_00023691] |
| XLOC_007647 | TCONS_00016270 | linc|BROAD Institute lincRNA (XLOC_007647), lincRNA [TCONS_00016270] |
| NPR3 | NM_001204375 | natriuretic peptide receptor C/guanylate cyclase C (atrionatriuretic peptide receptor C) (NPR3), transcript variant 1, mRNA [NM_001204375] |
| GAL3ST3 | NM_033036 | galactose-3-O-sulfotransferase 3 (GAL3ST3), mRNA [NM_033036] |
| SNORD99 | NR_003077 | small nucleolar RNA, C/D box 99 (SNORD99), small nucleolar RNA [NR_003077] |
| XLOC_013812 | TCONS_00028710 | linc|BROAD Institute lincRNA (XLOC_013812), lincRNA [TCONS_00028710] |
| LOC100505729 | XR_108524 | ref|PREDICTED: Homo sapiens hypothetical LOC100505729 (LOC100505729), miscRNA [XR_108524] |
| ADRA1A | ENST00000380573 | ens|adrenergic, alpha-1A-, receptor [Source:HGNC Symbol;Acc:277] [ENST00000380573] |
| ENST00000514376 | ENST00000514376 | Unknown |
| C17orf98 | NM_001080465 | chromosome 17 open reading frame 98 (C17orf98), mRNA [NM_001080465] |
| RLIM | NM_183353 | ring finger protein, LIM domain interacting (RLIM), transcript variant 2, mRNA [NM_183353] |
| PATZ1 | NM_032051 | POZ (BTB) and AT hook containing zinc finger 1 (PATZ1), transcript variant 4, mRNA [NM_032051] |
| A_19_P00811234 | A_19_P00811234 | Unknown |
| CDCP1 | NM_022842 | CUB domain containing protein 1 (CDCP1), transcript variant 1, mRNA [NM_022842] |
| XLOC_011082 | TCONS_00022805 | linc|BROAD Institute lincRNA (XLOC_011082), lincRNA [TCONS_00022805] |
| XLOC_l2_009790 | BC063131 | tc|MUSKE3A ribosomal protein {Mus musculus} (exp=-1; wgp=0; cg=0), partial (26%) [THC2611235] |
| BC009492 | BC009492 | gb|Homo sapiens hypothetical LOC114130, mRNA (cDNA clone IMAGE:3937896). [BC009492] |
| LPHN3 | NM_015236 | latrophilin 3 (LPHN3), mRNA [NM_015236] |
| SOX14 | NM_004189 | SRY (sex determining region Y)-box 14 (SOX14), mRNA [NM_004189] |
| XLOC_010287 | ENST00000422082 | linc|BROAD Institute lincRNA (XLOC_010287), lincRNA [TCONS_00021524] |
| CLIC5 | NM_016929 | chloride intracellular channel 5 (CLIC5), nuclear gene encoding mitochondrial protein, transcript variant 2, mRNA [NM_016929] |
| CBFB | NM_001755 | core-binding factor, beta subunit (CBFB), transcript variant 2, mRNA [NM_001755] |
| THC2539168 | THC2539168 | tc|ALU1_HUMAN (P39188) Alu subfamily J sequence contamination warning entry, partial (11%) [THC2539168] |
| XLOC_l2_002790 | AK126420 | linc|BROAD Institute lincRNA (XLOC_l2_002790), lincRNA [TCONS_l2_00005211] |
| C7orf74 | NM_175884 | chromosome 7 open reading frame 74 (C7orf74), mRNA [NM_175884] |
| ENST00000469997 | ENST00000469997 | ens|ribosomal protein S2 pseudogene 45 [Source:HGNC Symbol;Acc:25709] [ENST00000469997] |
| ENST00000557121 | ENST00000557121 | gb|AF150244 Human mRNA from cd34+ stem cells Homo sapiens cDNA clone CBFBBE12, mRNA sequence [AF150244] |
| MUC12 | NM_001164462 | mucin 12, cell surface associated (MUC12), mRNA [NM_001164462] |
| A_33_P3221059 | A_33_P3221059 | Unknown |
| Q6UWM8 | AK022898 | tc|Q6UWM8_HUMAN (Q6UWM8) GNNC2999, partial (16%) [THC2606542] |
| ZNF223 | NM_013361 | zinc finger protein 223 (ZNF223), mRNA [NM_013361] |
| XLOC_l2_006578 | TCONS_l2_00012272 | linc|BROAD Institute lincRNA (XLOC_l2_006578), lincRNA [TCONS_l2_00012272] |
| MAML1 | NM_014757 | mastermind-like 1 (Drosophila) (MAML1), mRNA [NM_014757] |
| LOC100134167 | XR_133543 | ref|PREDICTED: Homo sapiens uncharacterized protein C2orf27-like (LOC100134167), miscRNA [XR_133543] |
| NECAP1 | NM_015509 | NECAP endocytosis associated 1 (NECAP1), transcript variant 1, mRNA [NM_015509] |
| XLOC_l2_003178 | TCONS_l2_00005930 | linc|BROAD Institute lincRNA (XLOC_l2_003178), lincRNA [TCONS_l2_00005930] |
| LOC100509323 | XM_003119950 | ref|PREDICTED: Homo sapiens baculoviral IAP repeat-containing protein 1-like (LOC100509323), mRNA [XM_003119950] |
| ORC4 | NM_002552 | origin recognition complex, subunit 4 (ORC4), transcript variant 2, mRNA [NM_002552] |
| XLOC_005592 | AK126470 | tc|BC008282 SH3-domain binding protein 1 {Homo sapiens} (exp=-1; wgp=0; cg=0), partial (4%) [THC2618827] |
| IL7 | NM_000880 | interleukin 7 (IL7), transcript variant 1, mRNA [NM_000880] |
| XLOC_l2_010723 | THC2524841 | linc|BROAD Institute lincRNA (XLOC_l2_010723), lincRNA [TCONS_l2_00021618] |
| THC2581562 | THC2581562 | tc|Q6PII7_HUMAN (Q6PII7) GATS protein (Fragment), partial (10%) [THC2581562] |
| XLOC_004459 | TCONS_00010736 | linc|BROAD Institute lincRNA (XLOC_004459), lincRNA [TCONS_00010736] |
| CRISP2 | ENST00000211238 | ens|cysteine-rich secretory protein 2 [Source:HGNC Symbol;Acc:12024] [ENST00000211238] |
| C1orf194 | NM_001122961 | chromosome 1 open reading frame 194 (C1orf194), mRNA [NM_001122961] |
| XLOC_006337 | ENST00000437145 | linc|BROAD Institute lincRNA (XLOC_006337), lincRNA [TCONS_00013118] |
| DIRC3 | NR_026597 | disrupted in renal carcinoma 3 (DIRC3), non-coding RNA [NR_026597] |
| C2orf49 | ENST00000258457 | ens|chromosome 2 open reading frame 49 [Source:HGNC Symbol;Acc:28772] [ENST00000258457] |
| KRT83 | NM_002282 | keratin 83 (KRT83), mRNA [NM_002282] |
| LOC100132147 | BC036435 | gb|Homo sapiens cDNA clone IMAGE:4816083, partial cds. [BC036435] |
| ENST00000372387 | ENST00000372387 | ens|Uncharacterized proteincDNA FLJ43696 fis, clone TBAES2007964 [Source:UniProtKB/TrEMBL;Acc:Q6ZUH9] [ENST00000372387] |
| GRIA4 | NM_001077243 | glutamate receptor, ionotrophic, AMPA 4 (GRIA4), transcript variant 2, mRNA [NM_001077243] |
| MS4A5 | NM_023945 | membrane-spanning 4-domains, subfamily A, member 5 (MS4A5), mRNA [NM_023945] |
| FAM65B | NM_015864 | family with sequence similarity 65, member B (FAM65B), transcript variant 2, mRNA [NM_015864] |
| LENG1 | NM_024316 | leukocyte receptor cluster (LRC) member 1 (LENG1), mRNA [NM_024316] |
| XLOC_004294 | TCONS_00009874 | linc|BROAD Institute lincRNA (XLOC_004294), lincRNA [TCONS_00009874] |
| XLOC_013675 | ENST00000455890 | linc|BROAD Institute lincRNA (XLOC_013675), lincRNA [TCONS_00028014] |
| XLOC_009769 | ENST00000548617 | linc|BROAD Institute lincRNA (XLOC_009769), lincRNA [TCONS_00020439] |
| EPHA3 | NM_182644 | EPH receptor A3 (EPHA3), transcript variant 2, mRNA [NM_182644] |
| SLC27A6 | NM_001017372 | solute carrier family 27 (fatty acid transporter), member 6 (SLC27A6), transcript variant 2, mRNA [NM_001017372] |
| SLC6A4 | NM_001045 | solute carrier family 6 (neurotransmitter transporter, serotonin), member 4 (SLC6A4), mRNA [NM_001045] |
| HERC2 | NM_004667 | hect domain and RLD 2 (HERC2), mRNA [NM_004667] |
| A_33_P3342146 | A_33_P3342146 | Unknown |
| FAM26D | NM_153036 | family with sequence similarity 26, member D (FAM26D), mRNA [NM_153036] |
| LOC100507065 | XR_110371 | ref|PREDICTED: Homo sapiens hypothetical LOC100507065, transcript variant 3 (LOC100507065), miscRNA [XR_110371] |
| XLOC_002560 | TCONS_00005376 | linc|BROAD Institute lincRNA (XLOC_002560), lincRNA [TCONS_00005376] |
| KIF18B | NM_001080443 | kinesin family member 18B (KIF18B), mRNA [NM_001080443] |
| KDM4C | NM_015061 | lysine (K)-specific demethylase 4C (KDM4C), transcript variant 1, mRNA [NM_015061] |
| XLOC_002070 | TCONS_00004230 | linc|BROAD Institute lincRNA (XLOC_002070), lincRNA [TCONS_00004230] |
| MAGI2 | NM_012301 | membrane associated guanylate kinase, WW and PDZ domain containing 2 (MAGI2), mRNA [NM_012301] |
| ANO4 | NM_178826 | anoctamin 4 (ANO4), mRNA [NM_178826] |
| TAS2R41 | NM_176883 | taste receptor, type 2, member 41 (TAS2R41), mRNA [NM_176883] |
| ZNF174 | NM_003450 | zinc finger protein 174 (ZNF174), transcript variant 1, mRNA [NM_003450] |
| XLOC_013938 | ENST00000416842 | gb|BX089509 Soares_testis_NHT Homo sapiens cDNA clone IMAGp998K124452 ; IMAGE:1751939, mRNA sequence [BX089509] |
| XLOC_004877 | TCONS_00010374 | linc|BROAD Institute lincRNA (XLOC_004877), lincRNA [TCONS_00010374] |
| OR51G1 | NM_001005237 | olfactory receptor, family 51, subfamily G, member 1 (OR51G1), mRNA [NM_001005237] |
| ZNF677 | NM_182609 | zinc finger protein 677 (ZNF677), mRNA [NM_182609] |
| TMF1 | NM_007114 | TATA element modulatory factor 1 (TMF1), mRNA [NM_007114] |
| LOC100289673 | NR_033175 | phosphoglycerate mutase family member 5 pseudogene (LOC100289673), non-coding RNA [NR_033175] |
| CSMD2 | ENST00000373377 | ens|CUB and Sushi multiple domains 2 [Source:HGNC Symbol;Acc:19290] [ENST00000373377] |
| BRD8 | NM_139199 | bromodomain containing 8 (BRD8), transcript variant 2, mRNA [NM_139199] |
| INPP5F | NM_014937 | inositol polyphosphate-5-phosphatase F (INPP5F), transcript variant 1, mRNA [NM_014937] |
| XLOC_011131 | ENST00000556035 | Unknown |
| OR1J2 | NM_054107 | olfactory receptor, family 1, subfamily J, member 2 (OR1J2), mRNA [NM_054107] |
| MRPL34 | NM_023937 | mitochondrial ribosomal protein L34 (MRPL34), nuclear gene encoding mitochondrial protein, mRNA [NM_023937] |
| LOC100653063 | XM_003403397 | ref|PREDICTED: Homo sapiens hypothetical protein LOC100653063 (LOC100653063), mRNA [XM_003403397] |
| XLOC_005771 | ENST00000430931 | linc|BROAD Institute lincRNA (XLOC_005771), lincRNA [TCONS_00011522] |
| SLC26A3 | NM_000111 | solute carrier family 26, member 3 (SLC26A3), mRNA [NM_000111] |
| AFF2 | NM_002025 | AF4/FMR2 family, member 2 (AFF2), transcript variant 1, mRNA [NM_002025] |
| C8orf86 | NM_207412 | chromosome 8 open reading frame 86 (C8orf86), mRNA [NM_207412] |
| PIWIL1 | NM_004764 | piwi-like 1 (Drosophila) (PIWIL1), transcript variant 1, mRNA [NM_004764] |
| SORCS2 | NM_020777 | sortilin-related VPS10 domain containing receptor 2 (SORCS2), mRNA [NM_020777] |
| MYOF | NM_133337 | myoferlin (MYOF), transcript variant 2, mRNA [NM_133337] |
| P02616 | ENST00000414404 | tc|PRVB_AMPME (P02616) Parvalbumin beta, partial (12%) [THC2671238] |
| LOC441461 | NR_038853 | uncharacterized LOC441461 (LOC441461), non-coding RNA [NR_038853] |
| THC2715547 | THC2715547 | tc|Q96DD6_HUMAN (Q96DD6) LOC119710 protein (HEPIS), partial (10%) [THC2715547] |
| LOC100505536 | NR_040043 | uncharacterized LOC100505536 (LOC100505536), non-coding RNA [NR_040043] |
| ZNF563 | NM_145276 | zinc finger protein 563 (ZNF563), mRNA [NM_145276] |
| FLJ33544 | AK090863 | gb|Homo sapiens cDNA FLJ33544 fis, clone BRAMY2008333. [AK090863] |
| LOC440910 | NR_030728 | uncharacterized LOC440910 (LOC440910), non-coding RNA [NR_030728] |
| AFG3L1P | NR_003226 | AFG3 ATPase family gene 3-like 1 (S. cerevisiae), pseudogene (AFG3L1P), transcript variant 1, non-coding RNA [NR_003226] |
| XLOC_009307 | TCONS_00019509 | linc|BROAD Institute lincRNA (XLOC_009307), lincRNA [TCONS_00019509] |
| UCHL1 | NM_004181 | ubiquitin carboxyl-terminal esterase L1 (ubiquitin thiolesterase) (UCHL1), mRNA [NM_004181] |
| GYPA | NM_002099 | glycophorin A (MNS blood group) (GYPA), mRNA [NM_002099] |
| XLOC_003780 | TCONS_00008324 | linc|BROAD Institute lincRNA (XLOC_003780), lincRNA [TCONS_00008324] |
| XLOC_012959 | TCONS_00027473 | linc|BROAD Institute lincRNA (XLOC_012959), lincRNA [TCONS_00027473] |
| MEIS1 | NM_002398 | Meis homeobox 1 (MEIS1), mRNA [NM_002398] |
| A_33_P3418571 | A_33_P3418571 | Unknown |
| COL4A3BP | NM_001130105 | collagen, type IV, alpha 3 (Goodpasture antigen) binding protein (COL4A3BP), transcript variant 3, mRNA [NM_001130105] |
| XLOC_006390 | THC2773766 | tc|Q3KII3_PSEPF (Q3KII3) Inner-membrane translocator, partial (6%) [THC2773766] |
| XLOC_008282 | TCONS_00017630 | linc|BROAD Institute lincRNA (XLOC_008282), lincRNA [TCONS_00017630] |
| XLOC_013781 | ENST00000417781 | tc|ALU6_HUMAN (P39193) Alu subfamily SP sequence contamination warning entry, partial (10%) [THC2682870] |
| UNC45B | NM_173167 | unc-45 homolog B (C. elegans) (UNC45B), transcript variant 1, mRNA [NM_173167] |
| NCKAP1 | NM_205842 | NCK-associated protein 1 (NCKAP1), transcript variant 2, mRNA [NM_205842] |
| XLOC_001937 | ENST00000445279 | ref|PREDICTED: Homo sapiens hypothetical LOC100508751 (LOC100508751), miscRNA [XR_112271] |
| XLOC_l2_001543 | THC2700693 | linc|BROAD Institute lincRNA (XLOC_l2_001543), lincRNA [TCONS_l2_00002966] |
| NRK | ENST00000536164 | ens|Nik related kinase [Source:HGNC Symbol;Acc:25391] [ENST00000536164] |
| CLDN5 | NM_001130861 | claudin 5 (CLDN5), transcript variant 1, mRNA [NM_001130861] |
| ZNF534 | NM_001143939 | zinc finger protein 534 (ZNF534), transcript variant 2, mRNA [NM_001143939] |
| MPP5 | NM_022474 | membrane protein, palmitoylated 5 (MAGUK p55 subfamily member 5) (MPP5), mRNA [NM_022474] |
| XLOC_001637 | TCONS_00003827 | linc|BROAD Institute lincRNA (XLOC_001637), lincRNA [TCONS_00003827] |
| WFDC6 | NM_080827 | WAP four-disulfide core domain 6 (WFDC6), mRNA [NM_080827] |
| A_33_P3286699 | A_33_P3286699 | Unknown |
| LOC100506578 | XR_132581 | ref|PREDICTED: Homo sapiens uncharacterized protein C12orf71-like (LOC100506578), miscRNA [XR_132581] |
| XLOC_l2_013124 | ENST00000417483 | Unknown |
| XLOC_004874 | TCONS_00010957 | linc|BROAD Institute lincRNA (XLOC_004874), lincRNA [TCONS_00010957] |
| XIRP2 | NM_152381 | xin actin-binding repeat containing 2 (XIRP2), transcript variant 1, mRNA [NM_152381] |
| XLOC_008379 | BG698024 | gb|602659169F1 NCI_CGAP_Skn3 Homo sapiens cDNA clone IMAGE:4802393 5', mRNA sequence [BG698024] |
| LRIG1 | NM_015541 | leucine-rich repeats and immunoglobulin-like domains 1 (LRIG1), mRNA [NM_015541] |
| PDE10A | NM_006661 | phosphodiesterase 10A (PDE10A), transcript variant 2, mRNA [NM_006661] |
| XLOC_013181 | TCONS_00027642 | linc|BROAD Institute lincRNA (XLOC_013181), lincRNA [TCONS_00027642] |
| FGF7 | NM_002009 | fibroblast growth factor 7 (FGF7), mRNA [NM_002009] |
| PRSS8 | NM_002773 | protease, serine, 8 (PRSS8), mRNA [NM_002773] |
| LOC100128242 | XR_110532 | ref|PREDICTED: Homo sapiens hypothetical LOC100128242 (LOC100128242), miscRNA [XR_110532] |
| DEFA4 | NM_001925 | defensin, alpha 4, corticostatin (DEFA4), mRNA [NM_001925] |
| LOC100506165 | XR_110206 | ref|PREDICTED: Homo sapiens hypothetical LOC100506165 (LOC100506165), miscRNA [XR_110206] |
| XLOC_l2_001851 | TCONS_l2_00003384 | linc|BROAD Institute lincRNA (XLOC_l2_001851), lincRNA [TCONS_l2_00003384] |
| A_33_P3418380 | A_33_P3418380 | Unknown |
| XLOC_007889 | ENST00000420883 | gb|602490627F1 NIH_MGC_18 Homo sapiens cDNA clone IMAGE:4622599 5', mRNA sequence [BG437415] |
| XLOC_005319 | TCONS_00011831 | linc|BROAD Institute lincRNA (XLOC_005319), lincRNA [TCONS_00011831] |
| XLOC_000730 | ENST00000418743 | linc|BROAD Institute lincRNA (XLOC_000730), lincRNA [TCONS_00000478] |
| TNS1 | NM_022648 | tensin 1 (TNS1), mRNA [NM_022648] |
| XLOC_012078 | H92146 | gb|ys84c11.r1 Soares retina N2b4HR Homo sapiens cDNA clone IMAGE:221492 5', mRNA sequence [H92146] |
| PLA2G4A | NM_024420 | phospholipase A2, group IVA (cytosolic, calcium-dependent) (PLA2G4A), mRNA [NM_024420] |
| LOC644192 | XR_109225 | ref|PREDICTED: Homo sapiens hypothetical LOC644192 (LOC644192), miscRNA [XR_109225] |
| MTRR | ENST00000511639 | ref|PREDICTED: Homo sapiens hypothetical LOC100288963, transcript variant 1 (LOC100288963), miscRNA [XR_108558] |
| NFIB | ENST00000380924 | ens|nuclear factor I/B [Source:HGNC Symbol;Acc:7785] [ENST00000380924] |
| LOC440386 | BC029817 | gb|Homo sapiens hypothetical gene supported by BC029817, mRNA (cDNA clone IMAGE:5167804), with apparent retained intron. [BC029817] |
| TARS2 | ENST00000369053 | ens|threonyl-tRNA synthetase 2, mitochondrial (putative) [Source:HGNC Symbol;Acc:30740] [ENST00000369053] |
| ZNF323 | NM_030899 | zinc finger protein 323 (ZNF323), transcript variant 1, mRNA [NM_030899] |
| XLOC_010414 | TCONS_00021813 | linc|BROAD Institute lincRNA (XLOC_010414), lincRNA [TCONS_00021813] |
| FZD5 | NM_003468 | frizzled family receptor 5 (FZD5), mRNA [NM_003468] |
| PKP3 | NM_007183 | plakophilin 3 (PKP3), mRNA [NM_007183] |
| HMGB3 | NM_005342 | high mobility group box 3 (HMGB3), mRNA [NM_005342] |
| VNN2 | NM_004665 | vanin 2 (VNN2), transcript variant 1, mRNA [NM_004665] |
| FLJ37035 | NR_033847 | uncharacterized LOC399821 (FLJ37035), non-coding RNA [NR_033847] |
| XLOC_003197 | TCONS_00006570 | linc|BROAD Institute lincRNA (XLOC_003197), lincRNA [TCONS_00006570] |
| LOC650623 | NR_027512 | BEN domain containing 3 pseudogene (LOC650623), non-coding RNA [NR_027512] |
| XLOC_000256 | ENST00000440762 | tc|ALU3_HUMAN (P39190) Alu subfamily SB1 sequence contamination warning entry, partial (18%) [THC2617426] |
| BCOR | ENST00000501455 | ens|BCL6 corepressor [Source:HGNC Symbol;Acc:20893] [ENST00000501455] |
| XLOC_003242 | TCONS_00006651 | linc|BROAD Institute lincRNA (XLOC_003242), lincRNA [TCONS_00006651] |
| ZNF780A | NM_001142579 | zinc finger protein 780A (ZNF780A), transcript variant 4, mRNA [NM_001142579] |
| FST | NM_013409 | follistatin (FST), transcript variant FST344, mRNA [NM_013409] |
| XLOC_l2_003401 | TCONS_l2_00006293 | linc|BROAD Institute lincRNA (XLOC_l2_003401), lincRNA [TCONS_l2_00006293] |
| TMOD1 | NM_003275 | tropomodulin 1 (TMOD1), transcript variant 1, mRNA [NM_003275] |
| SNORD92 | NR_003074 | small nucleolar RNA, C/D box 92 (SNORD92), small nucleolar RNA [NR_003074] |
| RAI1 | ENST00000395776 | ens|retinoic acid induced 1 [Source:HGNC Symbol;Acc:9834] [ENST00000395776] |
| XLOC_l2_015762 | TCONS_l2_00030539 | linc|BROAD Institute lincRNA (XLOC_l2_015762), lincRNA [TCONS_l2_00030539] |
| C22orf28 | NM_014306 | chromosome 22 open reading frame 28 (C22orf28), mRNA [NM_014306] |
| GFOD1 | NM_018988 | glucose-fructose oxidoreductase domain containing 1 (GFOD1), transcript variant 1, mRNA [NM_018988] |
| DTNBP1 | NM_183040 | dystrobrevin binding protein 1 (DTNBP1), transcript variant 2, mRNA [NM_183040] |
| XLOC_007605 | TCONS_00016213 | linc|BROAD Institute lincRNA (XLOC_007605), lincRNA [TCONS_00016213] |
| ARHGEF4 | NM_015320 | Rho guanine nucleotide exchange factor (GEF) 4 (ARHGEF4), transcript variant 1, mRNA [NM_015320] |
| LOC100292082 | XR_112768 | ref|PREDICTED: Homo sapiens hypothetical LOC100292082 (LOC100292082), miscRNA [XR_112768] |
| ZMYM5 | NM_001039650 | zinc finger, MYM-type 5 (ZMYM5), transcript variant 1, mRNA [NM_001039650] |
| LOC389906 | NR_034031 | zinc finger protein 839 pseudogene (LOC389906), non-coding RNA [NR_034031] |
| OR2A5 | NM_012365 | olfactory receptor, family 2, subfamily A, member 5 (OR2A5), mRNA [NM_012365] |
| LOC284570 | BC040156 | gb|Homo sapiens, clone IMAGE:4941949, mRNA. [BC040156] |
| XLOC_012515 | ENST00000508851 | linc|BROAD Institute lincRNA (XLOC_012515), lincRNA [TCONS_00025694] |
| PLCH1 | NM_014996 | phospholipase C, eta 1 (PLCH1), transcript variant 2, mRNA [NM_014996] |
| ZNF407 | NM_017757 | zinc finger protein 407 (ZNF407), transcript variant 1, mRNA [NM_017757] |
| APC | NM_000038 | adenomatous polyposis coli (APC), transcript variant 3, mRNA [NM_000038] |
| TFAP4 | NM_003223 | transcription factor AP-4 (activating enhancer binding protein 4) (TFAP4), mRNA [NM_003223] |
| ACER3 | NM_018367 | alkaline ceramidase 3 (ACER3), mRNA [NM_018367] |
| MIA2 | NM_054024 | melanoma inhibitory activity 2 (MIA2), mRNA [NM_054024] |
| XLOC_004565 | TCONS_00010100 | linc|BROAD Institute lincRNA (XLOC_004565), lincRNA [TCONS_00010100] |
| PVR | NM_006505 | poliovirus receptor (PVR), transcript variant 1, mRNA [NM_006505] |
| LOC150005 | AK057475 | gb|Homo sapiens cDNA FLJ32913 fis, clone TESTI2006255. [AK057475] |
| ANGPTL1 | NM_004673 | angiopoietin-like 1 (ANGPTL1), mRNA [NM_004673] |
| EFCAB6 | NM_022785 | EF-hand calcium binding domain 6 (EFCAB6), transcript variant 1, mRNA [NM_022785] |
| ZFAND3 | NM_021943 | zinc finger, AN1-type domain 3 (ZFAND3), mRNA [NM_021943] |
| XLOC_012645 | TCONS_00026300 | linc|BROAD Institute lincRNA (XLOC_012645), lincRNA [TCONS_00026300] |
| XLOC_010668 | ENST00000450187 | linc|BROAD Institute lincRNA (XLOC_010668), lincRNA [TCONS_00021675] |
| A_33_P3231247 | A_33_P3231247 | Unknown |
| XLOC_001638 | TCONS_00003829 | linc|BROAD Institute lincRNA (XLOC_001638), lincRNA [TCONS_00003829] |
| GPLD1 | NM_177483 | glycosylphosphatidylinositol specific phospholipase D1 (GPLD1), transcript variant 2, mRNA [NM_177483] |
| C1orf195 | AV653872 | gb|AV653872 GLC Homo sapiens cDNA clone GLCDPE06 3', mRNA sequence [AV653872] |
| XLOC_001619 | ENST00000439893 | gb|full-length cDNA clone CS0DD009YK02 of Neuroblastoma Cot 50-normalized of Homo sapiens (human) [CR626349] |
| XLOC_l2_007468 | TCONS_l2_00015425 | linc|BROAD Institute lincRNA (XLOC_l2_007468), lincRNA [TCONS_l2_00015425] |
| XLOC_l2_001222 | ENST00000420760 | linc|BROAD Institute lincRNA (XLOC_l2_001222), lincRNA [TCONS_l2_00002729] |
| XLOC_l2_005168 | TCONS_l2_00009650 | linc|BROAD Institute lincRNA (XLOC_l2_005168), lincRNA [TCONS_l2_00009650] |
| LOC100132495 | AK127414 | gb|Homo sapiens cDNA FLJ45506 fis, clone BRTHA2020695. [AK127414] |
| XLOC_l2_015710 | AK056524 | linc|BROAD Institute lincRNA (XLOC_l2_015710), lincRNA [TCONS_l2_00030806] |
| HCG27 | NR_026791 | HLA complex group 27 (non-protein coding) (HCG27), non-coding RNA [NR_026791] |
| XLOC_008375 | TCONS_00018115 | linc|BROAD Institute lincRNA (XLOC_008375), lincRNA [TCONS_00018115] |
| A_33_P3282624 | A_33_P3282624 | Unknown |
| RBPJL | NM_014276 | recombination signal binding protein for immunoglobulin kappa J region-like (RBPJL), mRNA [NM_014276] |
| LOC100131599 | AK126221 | gb|Homo sapiens cDNA FLJ44233 fis, clone THYMU3006963. [AK126221] |
| NUS1 | NM_138459 | nuclear undecaprenyl pyrophosphate synthase 1 homolog (S. cerevisiae) (NUS1), mRNA [NM_138459] |
| CSHL1 | NM_022579 | chorionic somatomammotropin hormone-like 1 (CSHL1), transcript variant 1, mRNA [NM_022579] |
| TACC1 | NM_006283 | transforming, acidic coiled-coil containing protein 1 (TACC1), transcript variant 1, mRNA [NM_006283] |
| FLJ44715 | AK126671 | gb|Homo sapiens cDNA FLJ44715 fis, clone BRACE3021430. [AK126671] |
| LOC339894 | NR_034007 | uncharacterized LOC339894 (LOC339894), non-coding RNA [NR_034007] |
| IDAS | NM_001190787 | Idas protein (IDAS), mRNA [NM_001190787] |
| UBE2O | NM_022066 | ubiquitin-conjugating enzyme E2O (UBE2O), mRNA [NM_022066] |
| C12orf33 | AK123808 | gb|Homo sapiens cDNA FLJ41814 fis, clone NT2RI2011683. [AK123808] |
| LOC100506847 | ENST00000451775 | ref|PREDICTED: Homo sapiens hypothetical LOC100506847 (LOC100506847), miscRNA [XR_111729] |
| XLOC_004929 | ENST00000515153 | ref|PREDICTED: Homo sapiens hypothetical LOC100505822 (LOC100505822), miscRNA [XR_110563] |
| GNG11 | NM_004126 | guanine nucleotide binding protein (G protein), gamma 11 (GNG11), mRNA [NM_004126] |
| ALG2 | NM_033087 | asparagine-linked glycosylation 2, alpha-1,3-mannosyltransferase homolog (S. cerevisiae) (ALG2), transcript variant 1, mRNA [NM_033087] |
| AK123255 | AK123255 | gb|Homo sapiens cDNA FLJ41261 fis, clone BRAMY2034920. [AK123255] |
| A_33_P3351388 | A_33_P3351388 | Unknown |
| CTNND1 | NM_001331 | catenin (cadherin-associated protein), delta 1 (CTNND1), transcript variant 3, mRNA [NM_001331] |
| TXNDC11 | NM_015914 | thioredoxin domain containing 11 (TXNDC11), mRNA [NM_015914] |
| FLJ16124 | BC033059 | tc|ALU5_HUMAN (P39192) Alu subfamily SC sequence contamination warning entry, partial (9%) [THC2488342] |
| LOC100506022 | XR_109872 | ref|PREDICTED: Homo sapiens hypothetical LOC100506022 (LOC100506022), miscRNA [XR_109872] |
| UBQLN2 | NM_013444 | ubiquilin 2 (UBQLN2), mRNA [NM_013444] |
| SLCO3A1 | NM_013272 | solute carrier organic anion transporter family, member 3A1 (SLCO3A1), transcript variant 1, mRNA [NM_013272] |
| ZNF365 | NM_199450 | zinc finger protein 365 (ZNF365), transcript variant B, mRNA [NM_199450] |
| DUOXA1 | EU927394 | gb|Homo sapiens dual oxidase maturation factor 1 alpha (DUOXA1) mRNA, complete cds, alternatively spliced. [EU927394] |
| SELP | NM_003005 | selectin P (granule membrane protein 140kDa, antigen CD62) (SELP), mRNA [NM_003005] |
| SPDYE8P | NR_003664 | speedy homolog E8 (Xenopus laevis), pseudogene (SPDYE8P), non-coding RNA [NR_003664] |
| XLOC_006409 | TCONS_00014282 | linc|BROAD Institute lincRNA (XLOC_006409), lincRNA [TCONS_00014282] |
| C18orf18 | NR_026849 | chromosome 18 open reading frame 18 (C18orf18), non-coding RNA [NR_026849] |
| PAPPA2 | NM_021936 | pappalysin 2 (PAPPA2), transcript variant 2, mRNA [NM_021936] |
| DQ786257 | DQ786257 | gb|Homo sapiens clone HLS_IMAGE_281777 mRNA sequence. [DQ786257] |
| NFIL3 | NM_005384 | nuclear factor, interleukin 3 regulated (NFIL3), mRNA [NM_005384] |
| MGST2 | NM_002413 | microsomal glutathione S-transferase 2 (MGST2), transcript variant 1, mRNA [NM_002413] |
| POLR3G | NM_006467 | polymerase (RNA) III (DNA directed) polypeptide G (32kD) (POLR3G), mRNA [NM_006467] |
| A_33_P3236117 | A_33_P3236117 | Unknown |
| ENST00000441009 | ENST00000441009 | gb|Homo sapiens hypothetical protein LOC339622, mRNA (cDNA clone IMAGE:4836841). [BC040319] |
| NFASC | NM_001005389 | neurofascin (NFASC), transcript variant 5, mRNA [NM_001005389] |
| ARL4A | NM_005738 | ADP-ribosylation factor-like 4A (ARL4A), transcript variant 1, mRNA [NM_005738] |
| VTCN1 | NM_024626 | V-set domain containing T cell activation inhibitor 1 (VTCN1), mRNA [NM_024626] |
| DIEXF | NM_014388 | digestive organ expansion factor homolog (zebrafish) (DIEXF), mRNA [NM_014388] |
| ARAF | ENST00000377039 | ens|v-raf murine sarcoma 3611 viral oncogene homolog [Source:HGNC Symbol;Acc:646] [ENST00000377039] |
| UTS2 | NM_021995 | urotensin 2 (UTS2), transcript variant 1, mRNA [NM_021995] |
| ZNF782 | NM_001001662 | zinc finger protein 782 (ZNF782), mRNA [NM_001001662] |
| LOC100270804 | NR_026885 | uncharacterized LOC100270804 (LOC100270804), non-coding RNA [NR_026885] |
| C11orf83 | NM_001085372 | chromosome 11 open reading frame 83 (C11orf83), mRNA [NM_001085372] |
| XLOC_007393 | TCONS_00016002 | linc|BROAD Institute lincRNA (XLOC_007393), lincRNA [TCONS_00016002] |
| NAT8 | NM_003960 | N-acetyltransferase 8 (GCN5-related, putative) (NAT8), mRNA [NM_003960] |
| RAB1A | NM_004161 | RAB1A, member RAS oncogene family (RAB1A), transcript variant 1, mRNA [NM_004161] |
| XLOC_001048 | ENST00000428641 | gb|DA336930 BRHIP3 Homo sapiens cDNA clone BRHIP3037662 5', mRNA sequence [DA336930] |
| CABP7 | NM_182527 | calcium binding protein 7 (CABP7), mRNA [NM_182527] |
| FLJ42022 | AK124016 | gb|Homo sapiens cDNA FLJ42022 fis, clone SPLEN2034678. [AK124016] |
| ZIC3 | NM_003413 | Zic family member 3 (ZIC3), mRNA [NM_003413] |
| XLOC_006722 | TCONS_00014625 | linc|BROAD Institute lincRNA (XLOC_006722), lincRNA [TCONS_00014625] |
| XLOC_004478 | TCONS_00010749 | linc|BROAD Institute lincRNA (XLOC_004478), lincRNA [TCONS_00010749] |
| MUC21 | NM_001010909 | mucin 21, cell surface associated (MUC21), mRNA [NM_001010909] |
| PLCZ1 | NM_033123 | phospholipase C, zeta 1 (PLCZ1), mRNA [NM_033123] |
| C6orf164 | NR_026784 | chromosome 6 open reading frame 164 (C6orf164), non-coding RNA [NR_026784] |
| TAS1R2 | NM_152232 | taste receptor, type 1, member 2 (TAS1R2), mRNA [NM_152232] |
| XLOC_000008 | TCONS_00001909 | linc|BROAD Institute lincRNA (XLOC_000008), lincRNA [TCONS_00001909] |
| ENST00000479981 | ENST00000479981 | ens|immunoglobulin kappa variable 1-16 [Source:HGNC Symbol;Acc:5732] [ENST00000479981] |
| XLOC_003786 | TCONS_00008336 | linc|BROAD Institute lincRNA (XLOC_003786), lincRNA [TCONS_00008336] |
| SNORD119 | NR_003684 | small nucleolar RNA, C/D box 119 (SNORD119), small nucleolar RNA [NR_003684] |
| XLOC_004435 | ENST00000512650 | linc|BROAD Institute lincRNA (XLOC_004435), lincRNA [TCONS_00009428] |
| LOC100129125 | AK125712 | gb|Homo sapiens cDNA FLJ43724 fis, clone TESOP2007688. [AK125712] |
| REV3L | NM_002912 | REV3-like, catalytic subunit of DNA polymerase zeta (yeast) (REV3L), mRNA [NM_002912] |
| GPR65 | NM_003608 | G protein-coupled receptor 65 (GPR65), mRNA [NM_003608] |
| MMP1 | NM_002421 | matrix metallopeptidase 1 (interstitial collagenase) (MMP1), transcript variant 1, mRNA [NM_002421] |
| XLOC_l2_011098 | ENST00000515128 | linc|BROAD Institute lincRNA (XLOC_l2_011098), lincRNA [TCONS_l2_00021860] |
| XLOC_000255 | TCONS_00000984 | linc|BROAD Institute lincRNA (XLOC_000255), lincRNA [TCONS_00000984] |
| WNT10A | NM_025216 | wingless-type MMTV integration site family, member 10A (WNT10A), mRNA [NM_025216] |
| ENST00000531730 | ENST00000531730 | gb|BX404796 Homo sapiens FETAL LIVER Homo sapiens cDNA clone CS0DM011YO11 5-PRIME, mRNA sequence [BX404796] |
| LOC100500938 | NM_001195637 | uncharacterized LOC100500938 (LOC100500938), mRNA [NM_001195637] |
| LOC100505633 | NR_038849 | uncharacterized LOC100505633 (LOC100505633), non-coding RNA [NR_038849] |
| ALK | NM_004304 | anaplastic lymphoma receptor tyrosine kinase (ALK), mRNA [NM_004304] |
| OR13A1 | NM_001004297 | olfactory receptor, family 13, subfamily A, member 1 (OR13A1), mRNA [NM_001004297] |
| LOC283070 | NR_027322 | uncharacterized LOC283070 (LOC283070), non-coding RNA [NR_027322] |
| XLOC_008149 | TCONS_00017313 | linc|BROAD Institute lincRNA (XLOC_008149), lincRNA [TCONS_00017313] |
| CR749422 | CR749422 | gb|Homo sapiens mRNA; cDNA DKFZp686E13230 (from clone DKFZp686E13230). [CR749422] |
| OLR1 | NM_002543 | oxidized low density lipoprotein (lectin-like) receptor 1 (OLR1), transcript variant 1, mRNA [NM_002543] |
| ZFYVE28 | NM_020972 | zinc finger, FYVE domain containing 28 (ZFYVE28), transcript variant 2, mRNA [NM_020972] |
| SSFA2 | NM_006751 | sperm specific antigen 2 (SSFA2), transcript variant 2, mRNA [NM_006751] |
| TDRD5 | NM_001199085 | tudor domain containing 5 (TDRD5), transcript variant 1, mRNA [NM_001199085] |
| ENST00000547786 | ENST00000547786 | gb|603078292F1 NIH_MGC_119 Homo sapiens cDNA clone IMAGE:5169862 5', mRNA sequence [BI828513] |
| LINC00221 | NR_027457 | long intergenic non-protein coding RNA 221 (LINC00221), non-coding RNA [NR_027457] |
| LOC100505815 | NR_045370 | uncharacterized LOC100505815 (LOC100505815), non-coding RNA [NR_045370] |
| XLOC_007469 | BC071732 | gb|DKFZp686M0829_r1 686 (synonym: hlcc3) Homo sapiens cDNA clone DKFZp686M0829 5', mRNA sequence [AL704255] |
| ISPD | NM_001101426 | isoprenoid synthase domain containing (ISPD), transcript variant 1, mRNA [NM_001101426] |
| XLOC_l2_005826 | ENST00000506394 | linc|BROAD Institute lincRNA (XLOC_l2_005826), lincRNA [TCONS_l2_00011572] |
| HELB | NM_033647 | helicase (DNA) B (HELB), mRNA [NM_033647] |
| XLOC_008165 | TCONS_00017322 | linc|BROAD Institute lincRNA (XLOC_008165), lincRNA [TCONS_00017322] |
| LOC100506115 | ENST00000422459 | ref|PREDICTED: Homo sapiens hypothetical LOC100506115, transcript variant 1 (LOC100506115), miscRNA [XR_109621] |
| KNDC1 | NM_152643 | kinase non-catalytic C-lobe domain (KIND) containing 1 (KNDC1), transcript variant 1, mRNA [NM_152643] |
| IQCF3 | NM_001085479 | IQ motif containing F3 (IQCF3), transcript variant 1, mRNA [NM_001085479] |
| LOC400940 | AK123041 | linc|BROAD Institute lincRNA (XLOC_001327), lincRNA [TCONS_00002804] |
| XLOC_006735 | ENST00000519996 | tc|Q3AVL6_SYNS9 (Q3AVL6) Phospho-N-acetylmuramoyl-pentapeptide transferase , partial (5%) [THC2617891] |
| A_33_P3211098 | A_33_P3211098 | Unknown |
| FKSG29 | NR_024013 | FKSG29 (FKSG29), non-coding RNA [NR_024013] |
| XLOC_007556 | AK126245 | tc|Q86TS6_HUMAN (Q86TS6) Full-length cDNA 5-PRIME end of clone CS0CAP004YO05 of Thymus of Homo sapiens (human) (Fragment), partial (13%) [THC2506250] |
| XAGE3 | NM_130776 | X antigen family, member 3 (XAGE3), transcript variant 2, mRNA [NM_130776] |
| A_33_P3330972 | A_33_P3330972 | Unknown |
| POLR1E | BC014180 | Unknown |
| CCR2 | NM_001123396 | chemokine (C-C motif) receptor 2 (CCR2), transcript variant B, mRNA [NM_001123396] |
| SLITRK2 | NM_032539 | SLIT and NTRK-like family, member 2 (SLITRK2), transcript variant 1, mRNA [NM_032539] |
| XLOC_005488 | TCONS_00011978 | linc|BROAD Institute lincRNA (XLOC_005488), lincRNA [TCONS_00011978] |
| GCFC1-AS1 | NR_038879 | GCFC1 antisense RNA 1 (non-protein coding) (GCFC1-AS1), transcript variant 1, non-coding RNA [NR_038879] |
| OR8G5 | NM_001005198 | olfactory receptor, family 8, subfamily G, member 5 (OR8G5), mRNA [NM_001005198] |
| XLOC_l2_011095 | TCONS_l2_00021100 | linc|BROAD Institute lincRNA (XLOC_l2_011095), lincRNA [TCONS_l2_00021100] |
| FAM22A | NM_001099338 | family with sequence similarity 22, member A (FAM22A), mRNA [NM_001099338] |
| XLOC_006817 | ENST00000520862 | Unknown |
| ATRX | ENST00000373341 | ens|alpha thalassemia/mental retardation syndrome X-linked [Source:HGNC Symbol;Acc:886] [ENST00000373341] |
| AP4E1 | NM_007347 | adaptor-related protein complex 4, epsilon 1 subunit (AP4E1), transcript variant 1, mRNA [NM_007347] |
| LRRC17 | NM_005824 | leucine rich repeat containing 17 (LRRC17), transcript variant 2, mRNA [NM_005824] |
| SAT1 | ENST00000379253 | ens|spermidine/spermine N1-acetyltransferase 1 [Source:HGNC Symbol;Acc:10540] [ENST00000379253] |
| C15orf41 | NM_032499 | chromosome 15 open reading frame 41 (C15orf41), transcript variant 2, mRNA [NM_032499] |
| XLOC_l2_015037 | AK057050 | linc|BROAD Institute lincRNA (XLOC_l2_015037), lincRNA [TCONS_l2_00029078] |
| XLOC_008395 | ENST00000448272 | linc|BROAD Institute lincRNA (XLOC_008395), lincRNA [TCONS_00017790] |
| SERPINA7 | NM_000354 | serpin peptidase inhibitor, clade A (alpha-1 antiproteinase, antitrypsin), member 7 (SERPINA7), mRNA [NM_000354] |
| SNORA69 | NR_002584 | small nucleolar RNA, H/ACA box 69 (SNORA69), small nucleolar RNA [NR_002584] |
| THC2773489 | THC2773489 | Unknown |
| TMEM229A | NM_001136002 | transmembrane protein 229A (TMEM229A), mRNA [NM_001136002] |
| XLOC_011143 | TCONS_00022889 | linc|BROAD Institute lincRNA (XLOC_011143), lincRNA [TCONS_00022889] |
| GPN1 | NM_007266 | GPN-loop GTPase 1 (GPN1), transcript variant 1, mRNA [NM_007266] |
| LOC100652951 | XR_132888 | ref|PREDICTED: Homo sapiens hypothetical LOC100652951 (LOC100652951), miscRNA [XR_132888] |
| OR2AG1 | NM_001004489 | olfactory receptor, family 2, subfamily AG, member 1 (OR2AG1), mRNA [NM_001004489] |
| TDG | NM_003211 | thymine-DNA glycosylase (TDG), mRNA [NM_003211] |
| VIL1 | NM_007127 | villin 1 (VIL1), mRNA [NM_007127] |
| WFDC10A | NM_080753 | WAP four-disulfide core domain 10A (WFDC10A), mRNA [NM_080753] |
| LOC400620 | BC014643 | gb|Homo sapiens, clone IMAGE:3342755, mRNA, partial cds. [BC014643] |
| A_33_P3232644 | A_33_P3232644 | Unknown |
| AOX2P | NR_001557 | aldehyde oxidase 2 pseudogene (AOX2P), non-coding RNA [NR_001557] |
| C1QTNF9 | NM_178540 | C1q and tumor necrosis factor related protein 9 (C1QTNF9), mRNA [NM_178540] |
| SPATA9 | NM_031952 | spermatogenesis associated 9 (SPATA9), mRNA [NM_031952] |
| XLOC_013558 | ENST00000444478 | linc|BROAD Institute lincRNA (XLOC_013558), lincRNA [TCONS_00027945] |
| CCIN | NM_005893 | calicin (CCIN), mRNA [NM_005893] |
| FOPNL | NM_144600 | FGFR1OP N-terminal like (FOPNL), mRNA [NM_144600] |
| LINC00472 | NR_026807 | long intergenic non-protein coding RNA 472 (LINC00472), non-coding RNA [NR_026807] |
| HIVEP3 | NM_024503 | human immunodeficiency virus type I enhancer binding protein 3 (HIVEP3), transcript variant 1, mRNA [NM_024503] |
| LECT1 | NM_007015 | leukocyte cell derived chemotaxin 1 (LECT1), transcript variant 1, mRNA [NM_007015] |
| LOC100506995 | XR_108482 | ref|PREDICTED: Homo sapiens hypothetical LOC100506995 (LOC100506995), miscRNA [XR_108482] |
| LOC100506003 | ENST00000417305 | ref|PREDICTED: Homo sapiens hypothetical LOC100506003 (LOC100506003), miscRNA [XR_109785] |
| SEZ6 | NM_178860 | seizure related 6 homolog (mouse) (SEZ6), transcript variant 1, mRNA [NM_178860] |
| XLOC_006344 | TCONS_00013682 | linc|BROAD Institute lincRNA (XLOC_006344), lincRNA [TCONS_00013682] |
| BEND3 | NM_001080450 | BEN domain containing 3 (BEND3), mRNA [NM_001080450] |
| XLOC_012617 | TCONS_00026269 | linc|BROAD Institute lincRNA (XLOC_012617), lincRNA [TCONS_00026269] |
| CDH1 | NM_004360 | cadherin 1, type 1, E-cadherin (epithelial) (CDH1), mRNA [NM_004360] |
| XLOC_005915 | AK090788 | linc|BROAD Institute lincRNA (XLOC_005915), lincRNA [TCONS_00011240] |
| FAM183A | ENST00000409396 | ens|family with sequence similarity 183, member A [Source:HGNC Symbol;Acc:34347] [ENST00000409396] |
| RBMY3AP | NR_001573 | RNA binding motif protein, Y-linked, family 3, member A pseudogene (RBMY3AP), non-coding RNA [NR_001573] |
| IMPG1 | NM_001563 | interphotoreceptor matrix proteoglycan 1 (IMPG1), mRNA [NM_001563] |
| LOC100129397 | AK095841 | gb|Homo sapiens cDNA FLJ38522 fis, clone HCHON2000818. [AK095841] |
| YPEL2 | NM_001005404 | yippee-like 2 (Drosophila) (YPEL2), mRNA [NM_001005404] |
| FNDC3B | NM_022763 | fibronectin type III domain containing 3B (FNDC3B), transcript variant 1, mRNA [NM_022763] |
| ENST00000424630 | ENST00000424630 | Unknown |
| XLOC_000628 | TCONS_00001333 | linc|BROAD Institute lincRNA (XLOC_000628), lincRNA [TCONS_00001333] |
| A_33_P3351499 | A_33_P3351499 | Unknown |
| C9orf57 | NM_001128618 | chromosome 9 open reading frame 57 (C9orf57), mRNA [NM_001128618] |
| BFSP1 | NM_001195 | beaded filament structural protein 1, filensin (BFSP1), transcript variant 1, mRNA [NM_001195] |
| L26245 | L26245 | gb|Human effector cell protease receptor-1 (EPR-1) mRNA, partial cds. [L26245] |
| PARVA | NM_018222 | parvin, alpha (PARVA), mRNA [NM_018222] |
| XLOC_014046 | TCONS_00029108 | linc|BROAD Institute lincRNA (XLOC_014046), lincRNA [TCONS_00029108] |
| LINC00113 | NR_024357 | long intergenic non-protein coding RNA 113 (LINC00113), non-coding RNA [NR_024357] |
| TRIM9 | NM_015163 | tripartite motif containing 9 (TRIM9), transcript variant 1, mRNA [NM_015163] |
| DNAJC16 | ENST00000375838 | ens|DnaJ (Hsp40) homolog, subfamily C, member 16 [Source:HGNC Symbol;Acc:29157] [ENST00000375838] |
| ENST00000507857 | ENST00000507857 | Unknown |
| ZNF557 | NM_024341 | zinc finger protein 557 (ZNF557), transcript variant 1, mRNA [NM_024341] |
| C5orf55 | NM_138464 | chromosome 5 open reading frame 55 (C5orf55), mRNA [NM_138464] |
| ENST00000376271 | ENST00000376271 | Unknown |
| SCAF11 | ENST00000395453 | ens|SR-related CTD-associated factor 11 [Source:HGNC Symbol;Acc:10784] [ENST00000395453] |
| XLOC_013323 | TCONS_00027327 | linc|BROAD Institute lincRNA (XLOC_013323), lincRNA [TCONS_00027327] |
| ENST00000370702 | ENST00000370702 | ens|zinc finger protein 451 [Source:HGNC Symbol;Acc:21091] [ENST00000370702] |
| LOC100653120 | XM_003403783 | ref|PREDICTED: Homo sapiens hypothetical protein LOC100653120 (LOC100653120), partial mRNA [XM_003403783] |
| XLOC_l2_004844 | TCONS_l2_00008931 | linc|BROAD Institute lincRNA (XLOC_l2_004844), lincRNA [TCONS_l2_00008931] |
| A_33_P3395843 | A_33_P3395843 | Unknown |
| XLOC_010586 | ENST00000421190 | linc|BROAD Institute lincRNA (XLOC_010586), lincRNA [TCONS_00021642] |
| THC2563836 | THC2563836 | tc|Q3SWV3_HUMAN (Q3SWV3) LOC389833 protein (Fragment), partial (8%) [THC2563836] |
| WLS | AB097018 | gb|Homo sapiens mRNA for putative NFkB activating protein, complete cds, clone: 373. [AB097018] |
| FLJ45950 | AK127847 | gb|Homo sapiens cDNA FLJ45950 fis, clone PLACE7008136. [AK127847] |
| LOC100009676 | NR_024407 | uncharacterized LOC100009676 (LOC100009676), non-coding RNA [NR_024407] |
| A_33_P3409294 | A_33_P3409294 | Unknown |
| XLOC_003284 | TCONS_00007281 | linc|BROAD Institute lincRNA (XLOC_003284), lincRNA [TCONS_00007281] |
| BLZF1 | NM_003666 | basic leucine zipper nuclear factor 1 (BLZF1), mRNA [NM_003666] |
| DNAL4 | ENST00000406199 | ens|dynein, axonemal, light chain 4 [Source:HGNC Symbol;Acc:2955] [ENST00000406199] |
| THC2601170 | THC2601170 | tc|Q1W9F5_9CHON (Q1W9F5) NADH dehydrogenase subunit 2, partial (5%) [THC2601170] |
| XLOC_l2_002100 | ENST00000447757 | gb|CR741727 Soares_testis_NHT Homo sapiens cDNA clone IMAGp971J0269 ; IMAGE:1641700 5', mRNA sequence [CR741727] |
| PPP4R1L | NR_003505 | protein phosphatase 4, regulatory subunit 1-like (PPP4R1L), non-coding RNA [NR_003505] |
| FAM26E | NM_153711 | family with sequence similarity 26, member E (FAM26E), mRNA [NM_153711] |
| C1orf101 | NM_173807 | chromosome 1 open reading frame 101 (C1orf101), transcript variant 2, mRNA [NM_173807] |
| ABCF1 | NM_001025091 | ATP-binding cassette, sub-family F (GCN20), member 1 (ABCF1), transcript variant 1, mRNA [NM_001025091] |
| SAMM50 | AK124895 | gb|Homo sapiens cDNA FLJ42905 fis, clone BRHIP3015751. [AK124895] |
| MALT1 | NM_006785 | mucosa associated lymphoid tissue lymphoma translocation gene 1 (MALT1), transcript variant 1, mRNA [NM_006785] |
| LOC100129502 | XR_132594 | ref|PREDICTED: Homo sapiens hypothetical LOC100129502 (LOC100129502), miscRNA [XR_132594] |
| TGM2 | NM_198951 | transglutaminase 2 (C polypeptide, protein-glutamine-gamma-glutamyltransferase) (TGM2), transcript variant 2, mRNA [NM_198951] |
| PASD1 | NM_173493 | PAS domain containing 1 (PASD1), mRNA [NM_173493] |
| PADI1 | NM_013358 | peptidyl arginine deiminase, type I (PADI1), mRNA [NM_013358] |
| XLOC_009371 | TCONS_00019572 | linc|BROAD Institute lincRNA (XLOC_009371), lincRNA [TCONS_00019572] |
| LOC730081 | AK097502 | gb|Homo sapiens cDNA FLJ40183 fis, clone TESTI2018421. [AK097502] |
| CLCA2 | NM_006536 | chloride channel accessory 2 (CLCA2), mRNA [NM_006536] |
| XLOC_011167 | TCONS_00023298 | linc|BROAD Institute lincRNA (XLOC_011167), lincRNA [TCONS_00023298] |
| P39194 | ENST00000431554 | tc|ALU7_HUMAN (P39194) Alu subfamily SQ sequence contamination warning entry, partial (13%) [THC2491736] |
| XLOC_005916 | TCONS_00012357 | linc|BROAD Institute lincRNA (XLOC_005916), lincRNA [TCONS_00012357] |
| XLOC_012840 | TCONS_00026519 | linc|BROAD Institute lincRNA (XLOC_012840), lincRNA [TCONS_00026519] |
| SNORA37 | AW835571 | gb|QV4-LT0016-090200-100-b12 LT0016 Homo sapiens cDNA, mRNA sequence [AW835571] |
| ECE2 | NM_014693 | endothelin converting enzyme 2 (ECE2), transcript variant 1, mRNA [NM_014693] |
| XLOC_005140 | TCONS_00011658 | linc|BROAD Institute lincRNA (XLOC_005140), lincRNA [TCONS_00011658] |
| ZCCHC14 | NM_015144 | zinc finger, CCHC domain containing 14 (ZCCHC14), mRNA [NM_015144] |
| GSTTP1 | NR_003081 | glutathione S-transferase theta pseudogene 1 (GSTTP1), non-coding RNA [NR_003081] |
| SENP1 | NM_014554 | SUMO1/sentrin specific peptidase 1 (SENP1), mRNA [NM_014554] |
| LOC400685 | NR_033982 | uncharacterized LOC400685 (LOC400685), non-coding RNA [NR_033982] |
| LOC100132474 | AK125160 | gb|Homo sapiens cDNA FLJ43170 fis, clone FCBBF3004847. [AK125160] |
| A_33_P3228868 | A_33_P3228868 | Unknown |
| KIF9 | ENST00000487440 | ens|kinesin family member 9 [Source:HGNC Symbol;Acc:16666] [ENST00000487440] |
| FOXA1 | NM_004496 | forkhead box A1 (FOXA1), mRNA [NM_004496] |
| A_33_P3359832 | A_33_P3359832 | Unknown |
| GRM4 | NM_000841 | glutamate receptor, metabotropic 4 (GRM4), mRNA [NM_000841] |
| THC2562053 | THC2562053 | tc|HSA312319 OVARIAN/Breast septin gamma {Homo sapiens} (exp=-1; wgp=0; cg=0), partial (23%) [THC2562053] |
| ANKH | NM_054027 | ankylosis, progressive homolog (mouse) (ANKH), mRNA [NM_054027] |
| AGMAT | NM_024758 | agmatine ureohydrolase (agmatinase) (AGMAT), mRNA [NM_024758] |
| XLOC_l2_005187 | TCONS_l2_00009686 | linc|BROAD Institute lincRNA (XLOC_l2_005187), lincRNA [TCONS_l2_00009686] |
| CD40 | NM_001250 | CD40 molecule, TNF receptor superfamily member 5 (CD40), transcript variant 1, mRNA [NM_001250] |
| LOC100506605 | XR_108966 | ref|PREDICTED: Homo sapiens hypothetical LOC100506605 (LOC100506605), miscRNA [XR_108966] |
| TATDN3 | NM_001146171 | TatD DNase domain containing 3 (TATDN3), transcript variant 5, mRNA [NM_001146171] |
| FNBP1L | NM_001024948 | formin binding protein 1-like (FNBP1L), transcript variant 1, mRNA [NM_001024948] |
| LOC349160 | AF279773 | gb|Homo sapiens clone N1 NTera2D1 teratocarcinoma mRNA. [AF279773] |
| LOC100128993 | NR_038919 | uncharacterized LOC100128993 (LOC100128993), non-coding RNA [NR_038919] |
| TBR1 | NM_006593 | T-box, brain, 1 (TBR1), mRNA [NM_006593] |
| XLOC_014022 | ENST00000452500 | gb|RST41718 Athersys RAGE Library Homo sapiens cDNA, mRNA sequence [BG221901] |
| XLOC_011790 | AI791749 | gb|oq51g11.y5 NCI_CGAP_Kid5 Homo sapiens cDNA clone IMAGE:1589924 5', mRNA sequence [AI791749] |
| XLOC_l2_012035 | BU568763 | gb|AGENCOURT_10402336 NIH_MGC_82 Homo sapiens cDNA clone IMAGE:6616007 5', mRNA sequence [BU568763] |
| KCNN2 | NM_021614 | potassium intermediate/small conductance calcium-activated channel, subfamily N, member 2 (KCNN2), transcript variant 1, mRNA [NM_021614] |
| XLOC_l2_001554 | TCONS_l2_00002984 | linc|BROAD Institute lincRNA (XLOC_l2_001554), lincRNA [TCONS_l2_00002984] |
| XLOC_l2_011921 | ENST00000506305 | gb|602698987F1 NIH_MGC_97 Homo sapiens cDNA clone IMAGE:4830932 5', mRNA sequence [BG717992] |
| OSTalpha | NM_152672 | organic solute transporter alpha (OSTalpha), mRNA [NM_152672] |
| MTSS1 | NM_014751 | metastasis suppressor 1 (MTSS1), mRNA [NM_014751] |
| MGC27382 | NR_027310 | uncharacterized MGC27382 (MGC27382), non-coding RNA [NR_027310] |
| LOC730020 | AK057873 | gb|Homo sapiens cDNA FLJ25144 fis, clone CBR07272. [AK057873] |
| DNAAF1 | NM_178452 | dynein, axonemal, assembly factor 1 (DNAAF1), mRNA [NM_178452] |
| MGC27345 | XR_108807 | ref|PREDICTED: Homo sapiens hypothetical protein MGC27345 (MGC27345), miscRNA [XR_108807] |
| XLOC_003578 | THC2668863 | linc|BROAD Institute lincRNA (XLOC_003578), lincRNA [TCONS_00008111] |
| ZNF827 | NM_178835 | zinc finger protein 827 (ZNF827), mRNA [NM_178835] |
| XLOC_007811 | ENST00000421507 | linc|BROAD Institute lincRNA (XLOC_007811), lincRNA [TCONS_00015858] |
| XLOC_010856 | ENST00000554187 | gb|DB051725 TESTI2 Homo sapiens cDNA clone TESTI2041710 5', mRNA sequence [DB051725] |
| BG719595 | BG719595 | gb|602689955F1 NIH_MGC_97 Homo sapiens cDNA clone IMAGE:4822453 5', mRNA sequence [BG719595] |
| XLOC_005807 | THC2745380 | linc|BROAD Institute lincRNA (XLOC_005807), lincRNA [TCONS_00012251] |
| SNORD90 | NR_003071 | small nucleolar RNA, C/D box 90 (SNORD90), small nucleolar RNA [NR_003071] |
| XLOC_002455 | TCONS_00004551 | linc|BROAD Institute lincRNA (XLOC_002455), lincRNA [TCONS_00004551] |
| AKAP4 | NM_003886 | A kinase (PRKA) anchor protein 4 (AKAP4), transcript variant 1, mRNA [NM_003886] |
| XLOC_l2_004120 | ENST00000453395 | tc|Q44G43_CHRSL (Q44G43) Twin-arginine translocation protein TatB, partial (9%) [THC2569855] |
| WDR55 | ENST00000358337 | tc|ALU4_HUMAN (P39191) Alu subfamily SB2 sequence contamination warning entry, partial (8%) [THC2546064] |
| LOC100506392 | XR_110490 | ref|PREDICTED: Homo sapiens hypothetical LOC100506392 (LOC100506392), miscRNA [XR_110490] |
| H1F0 | NM_005318 | H1 histone family, member 0 (H1F0), mRNA [NM_005318] |
| TLE1 | ENST00000376463 | ens|transducin-like enhancer of split 1 (E(sp1) homolog, Drosophila) [Source:HGNC Symbol;Acc:11837] [ENST00000376463] |
| MAP7 | NM_003980 | microtubule-associated protein 7 (MAP7), transcript variant 4, mRNA [NM_003980] |
| XLOC_006951 | ENST00000522374 | gb|BX108468 Soares_testis_NHT Homo sapiens cDNA clone IMAGp998J144405, mRNA sequence [BX108468] |
| LRRK2 | NM_198578 | leucine-rich repeat kinase 2 (LRRK2), mRNA [NM_198578] |
| XLOC_006051 | TCONS_00013414 | linc|BROAD Institute lincRNA (XLOC_006051), lincRNA [TCONS_00013414] |
| ZNF804A | NM_194250 | zinc finger protein 804A (ZNF804A), mRNA [NM_194250] |
| LOC100127961 | XM_001716322 | ref|PREDICTED: Homo sapiens hypothetical protein LOC100127961 (LOC100127961), mRNA [XM_001716322] |
| XLOC_008708 | TCONS_00018419 | linc|BROAD Institute lincRNA (XLOC_008708), lincRNA [TCONS_00018419] |
| XLOC_012572 | TCONS_00025762 | linc|BROAD Institute lincRNA (XLOC_012572), lincRNA [TCONS_00025762] |
| FCRL6 | NM_001004310 | Fc receptor-like 6 (FCRL6), mRNA [NM_001004310] |
| XLOC_l2_011399 | TCONS_l2_00021455 | linc|BROAD Institute lincRNA (XLOC_l2_011399), lincRNA [TCONS_l2_00021455] |
| DOCK8 | ENST00000382331 | ens|dedicator of cytokinesis 8 [Source:HGNC Symbol;Acc:19191] [ENST00000382331] |
| XLOC_l2_013277 | ENST00000447999 | linc|BROAD Institute lincRNA (XLOC_l2_013277), lincRNA [TCONS_l2_00027046] |
| XLOC_000933 | TCONS_00002412 | linc|BROAD Institute lincRNA (XLOC_000933), lincRNA [TCONS_00002412] |
| ENST00000434311 | ENST00000434311 | tc|XM_322591 predicted protein {Neurospora crassa} (exp=-1; wgp=0; cg=0), partial (9%) [THC2496490] |
| XLOC_001726 | TCONS_00003921 | linc|BROAD Institute lincRNA (XLOC_001726), lincRNA [TCONS_00003921] |
| GHRL | NM_016362 | ghrelin/obestatin prepropeptide (GHRL), transcript variant 1, mRNA [NM_016362] |
| RTP4 | NM_022147 | receptor (chemosensory) transporter protein 4 (RTP4), mRNA [NM_022147] |
| MCOLN3 | NM_018298 | mucolipin 3 (MCOLN3), mRNA [NM_018298] |
| KCNJ13 | NM_002242 | potassium inwardly-rectifying channel, subfamily J, member 13 (KCNJ13), transcript variant 1, mRNA [NM_002242] |
| XLOC_004984 | TCONS_00010463 | linc|BROAD Institute lincRNA (XLOC_004984), lincRNA [TCONS_00010463] |
| NLGN1 | NM_014932 | neuroligin 1 (NLGN1), mRNA [NM_014932] |
| AK093006 | AK093006 | gb|Homo sapiens cDNA FLJ35687 fis, clone SPLEN2019349. [AK093006] |
| DCAF4L1 | NM_001029955 | DDB1 and CUL4 associated factor 4-like 1 (DCAF4L1), mRNA [NM_001029955] |
| PCSK9 | NM_174936 | proprotein convertase subtilisin/kexin type 9 (PCSK9), mRNA [NM_174936] |
| TCF24 | NM_001193502 | transcription factor 24 (TCF24), mRNA [NM_001193502] |
| LOC643923 | NR_028328 | uncharacterized LOC643923 (LOC643923), non-coding RNA [NR_028328] |
| NCR2 | NM_004828 | natural cytotoxicity triggering receptor 2 (NCR2), transcript variant 1, mRNA [NM_004828] |
| C9orf170 | NM_001001709 | chromosome 9 open reading frame 170 (C9orf170), mRNA [NM_001001709] |
| SEPT7P2 | NR_024271 | septin 7 pseudogene 2 (SEPT7P2), non-coding RNA [NR_024271] |
| LOC100506385 | NR_038885 | uncharacterized LOC100506385 (LOC100506385), transcript variant 1, non-coding RNA [NR_038885] |
| SEC14L2 | NM_012429 | SEC14-like 2 (S. cerevisiae) (SEC14L2), transcript variant 1, mRNA [NM_012429] |
| LINC00207 | NR_028409 | long intergenic non-protein coding RNA 207 (LINC00207), transcript variant 1, non-coding RNA [NR_028409] |
| RDH8 | NM_015725 | retinol dehydrogenase 8 (all-trans) (RDH8), mRNA [NM_015725] |
| XLOC_012831 | BC051727 | tc|Q97C69_THEVO (Q97C69) TVG0245034 protein, partial (11%) [THC2605784] |
| A_33_P3209703 | A_33_P3209703 | Unknown |
| CD200 | NM_001004196 | CD200 molecule (CD200), transcript variant 2, mRNA [NM_001004196] |
| UNC5C | NM_003728 | unc-5 homolog C (C. elegans) (UNC5C), mRNA [NM_003728] |
| XLOC_l2_005175 | TCONS_l2_00009664 | linc|BROAD Institute lincRNA (XLOC_l2_005175), lincRNA [TCONS_l2_00009664] |
| LOC100131034 | AK126091 | gb|Homo sapiens cDNA FLJ44103 fis, clone TESTI4044084. [AK126091] |
| XLOC_014118 | ENST00000426578 | gb|UI-E-CK1-afk-m-13-0-UI.r1 UI-E-CK1 Homo sapiens cDNA clone UI-E-CK1-afk-m-13-0-UI 5', mRNA sequence [BM703952] |
| SIK1 | NM_173354 | salt-inducible kinase 1 (SIK1), mRNA [NM_173354] |
| XLOC_006009 | TCONS_00013374 | linc|BROAD Institute lincRNA (XLOC_006009), lincRNA [TCONS_00013374] |
| OSBP2 | NM_030758 | oxysterol binding protein 2 (OSBP2), transcript variant 1, mRNA [NM_030758] |
| SEL1L2 | NM_025229 | sel-1 suppressor of lin-12-like 2 (C. elegans) (SEL1L2), mRNA [NM_025229] |
| XLOC_000294 | TCONS_00001027 | linc|BROAD Institute lincRNA (XLOC_000294), lincRNA [TCONS_00001027] |
| COG8 | NM_032382 | component of oligomeric golgi complex 8 (COG8), mRNA [NM_032382] |
| XLOC_l2_000604 | TCONS_l2_00000794 | linc|BROAD Institute lincRNA (XLOC_l2_000604), lincRNA [TCONS_l2_00000794] |
| DNPEP | NM_012100 | aspartyl aminopeptidase (DNPEP), mRNA [NM_012100] |
| A_33_P3406923 | A_33_P3406923 | Unknown |
| LOC201617 | NR_038221 | uncharacterized LOC201617 (LOC201617), non-coding RNA [NR_038221] |
| KDM6A | NM_021140 | lysine (K)-specific demethylase 6A (KDM6A), mRNA [NM_021140] |
| C3orf38 | NM_173824 | chromosome 3 open reading frame 38 (C3orf38), mRNA [NM_173824] |
| XLOC_l2_008405 | ENST00000417006 | Unknown |
| FAM181B | NM_175885 | family with sequence similarity 181, member B (FAM181B), mRNA [NM_175885] |
| XLOC_010236 | TCONS_00020260 | linc|BROAD Institute lincRNA (XLOC_010236), lincRNA [TCONS_00020260] |
| COL13A1 | NM_080801 | collagen, type XIII, alpha 1 (COL13A1), transcript variant 5, mRNA [NM_080801] |
| MACC1 | NM_182762 | metastasis associated in colon cancer 1 (MACC1), mRNA [NM_182762] |
| TRIM32 | NM_012210 | tripartite motif containing 32 (TRIM32), transcript variant 1, mRNA [NM_012210] |
| PDE6A | NM_000440 | phosphodiesterase 6A, cGMP-specific, rod, alpha (PDE6A), mRNA [NM_000440] |
| XLOC_007953 | ENST00000423919 | linc|BROAD Institute lincRNA (XLOC_007953), lincRNA [TCONS_00016976] |
| LOC100507584 | NR_038989 | uncharacterized LOC100507584 (LOC100507584), non-coding RNA [NR_038989] |
| PRR5L | NM_024841 | proline rich 5 like (PRR5L), transcript variant 2, mRNA [NM_024841] |
| XLOC_007165 | ENST00000505564 | tc|ALU2_HUMAN (P39189) Alu subfamily SB sequence contamination warning entry, partial (9%) [THC2504761] |
| BG428231 | BG428231 | gb|602498889F1 NIH_MGC_75 Homo sapiens cDNA clone IMAGE:4612510 5', mRNA sequence [BG428231] |
| ANKRD32 | NM_032290 | ankyrin repeat domain 32 (ANKRD32), mRNA [NM_032290] |
| ABCA6 | NM_080284 | ATP-binding cassette, sub-family A (ABC1), member 6 (ABCA6), mRNA [NM_080284] |
| NAP1L6 | NR_027291 | nucleosome assembly protein 1-like 6 (NAP1L6), non-coding RNA [NR_027291] |
| CA5B | ENST00000380319 | ens|carbonic anhydrase VB, mitochondrial [Source:HGNC Symbol;Acc:1378] [ENST00000380319] |
| XLOC_013945 | THC2753734 | tc|Q5SH65_THET8 (Q5SH65) Serine protease, partial (4%) [THC2753734] |
| XLOC_011256 | TCONS_00023942 | linc|BROAD Institute lincRNA (XLOC_011256), lincRNA [TCONS_00023942] |
| SLC26A5 | NM_206883 | solute carrier family 26, member 5 (prestin) (SLC26A5), transcript variant b, mRNA [NM_206883] |
| XLOC_009529 | TCONS_00019737 | linc|BROAD Institute lincRNA (XLOC_009529), lincRNA [TCONS_00019737] |
| PLEKHO1 | NM_016274 | pleckstrin homology domain containing, family O member 1 (PLEKHO1), mRNA [NM_016274] |
| TTC12 | NM_017868 | tetratricopeptide repeat domain 12 (TTC12), mRNA [NM_017868] |
| XLOC_l2_000941 | TCONS_l2_00001279 | linc|BROAD Institute lincRNA (XLOC_l2_000941), lincRNA [TCONS_l2_00001279] |
| XLOC_004134 | TCONS_00009150 | linc|BROAD Institute lincRNA (XLOC_004134), lincRNA [TCONS_00009150] |
| LOC100652849 | XR_132692 | ref|PREDICTED: Homo sapiens hypothetical LOC100652849 (LOC100652849), miscRNA [XR_132692] |
| BRPF3 | NM_015695 | bromodomain and PHD finger containing, 3 (BRPF3), mRNA [NM_015695] |
| THC2505774 | THC2505774 | Unknown |
| A_33_P3300117 | A_33_P3300117 | Unknown |
| SNAR-G2 | NR_024244 | small ILF3/NF90-associated RNA G2 (SNAR-G2), small nuclear RNA [NR_024244] |
| AKR7L | NR_040288 | aldo-keto reductase family 7-like (AKR7L), transcript variant 1, non-coding RNA [NR_040288] |
| XLOC_l2_013636 | TCONS_l2_00026251 | linc|BROAD Institute lincRNA (XLOC_l2_013636), lincRNA [TCONS_l2_00026251] |
| LOC643401 | NR_038848 | uncharacterized LOC643401 (LOC643401), non-coding RNA [NR_038848] |
| XLOC_000539 | ENST00000417084 | linc|BROAD Institute lincRNA (XLOC_000539), lincRNA [TCONS_00000374] |
| C1orf174 | NM_207356 | chromosome 1 open reading frame 174 (C1orf174), mRNA [NM_207356] |
| XLOC_002961 | ENST00000434957 | gb|DB302275 BRAMY2 Homo sapiens cDNA clone BRAMY2024252 3', mRNA sequence [DB302275] |
| XLOC_011748 | ENST00000415422 | gb|DB275065 UTERU2 Homo sapiens cDNA clone UTERU2038356 5', mRNA sequence [DB275065] |
| LOC442421 | NR_024496 | uncharacterized LOC442421 (LOC442421), non-coding RNA [NR_024496] |
| XLOC_010897 | TCONS_00022584 | linc|BROAD Institute lincRNA (XLOC_010897), lincRNA [TCONS_00022584] |
| C17orf13 | CA309819 | gb|UI-H-FT1-bic-j-12-0-UI.s1 NCI_CGAP_FT1 Homo sapiens cDNA clone UI-H-FT1-bic-j-12-0-UI 3', mRNA sequence [CA309819] |
| STAU2 | NM_001164380 | staufen, RNA binding protein, homolog 2 (Drosophila) (STAU2), transcript variant 1, mRNA [NM_001164380] |
| TTC28 | NM_001145418 | tetratricopeptide repeat domain 28 (TTC28), mRNA [NM_001145418] |
| RNF7 | NM_014245 | ring finger protein 7 (RNF7), transcript variant 1, mRNA [NM_014245] |
| ENST00000529417 | ENST00000529417 | Unknown |
| RPA2 | NM_002946 | replication protein A2, 32kDa (RPA2), mRNA [NM_002946] |
| XLOC_000031 | CB105455 | gb|K-EST0126482 S13KMS5s1 Homo sapiens cDNA clone S13KMS5s1-30-G12 5', mRNA sequence [CB105455] |
| PPBP | NM_002704 | pro-platelet basic protein (chemokine (C-X-C motif) ligand 7) (PPBP), mRNA [NM_002704] |
| XLOC_002736 | ENST00000473756 | ref|PREDICTED: Homo sapiens hypothetical LOC100506377 (LOC100506377), miscRNA [XR_108458] |
| DIO2 | NM_001242502 | deiodinase, iodothyronine, type II (DIO2), transcript variant 4, mRNA [NM_001242502] |
| XLOC_007036 | TCONS_00014957 | linc|BROAD Institute lincRNA (XLOC_007036), lincRNA [TCONS_00014957] |
| CCNB3 | NM_033031 | cyclin B3 (CCNB3), transcript variant 3, mRNA [NM_033031] |
| SLC30A4 | NM_013309 | solute carrier family 30 (zinc transporter), member 4 (SLC30A4), mRNA [NM_013309] |
| OR4K14 | NM_001004712 | olfactory receptor, family 4, subfamily K, member 14 (OR4K14), mRNA [NM_001004712] |
| KIAA1875 | NR_024207 | KIAA1875 (KIAA1875), non-coding RNA [NR_024207] |
| A_33_P3240295 | A_33_P3240295 | Unknown |
| XLOC_l2_005731 | THC2569535 | tc|Q5IRE6_MOUSE (Q5IRE6) E3 ubiquitin ligase SMURF2, partial (8%) [THC2569535] |
| SCN1A | NM_001202435 | sodium channel, voltage-gated, type I, alpha subunit (SCN1A), transcript variant 4, mRNA [NM_001202435] |
| HS3ST2 | NM_006043 | heparan sulfate (glucosamine) 3-O-sulfotransferase 2 (HS3ST2), mRNA [NM_006043] |
| SEMA3G | NM_020163 | sema domain, immunoglobulin domain (Ig), short basic domain, secreted, (semaphorin) 3G (SEMA3G), mRNA [NM_020163] |
| XLOC_005633 | ENST00000424083 | linc|BROAD Institute lincRNA (XLOC_005633), lincRNA [TCONS_00011461] |
| FOXF2 | NM_001452 | forkhead box F2 (FOXF2), mRNA [NM_001452] |
| XLOC_l2_006905 | TCONS_l2_00012844 | linc|BROAD Institute lincRNA (XLOC_l2_006905), lincRNA [TCONS_l2_00012844] |
| XLOC_l2_009163 | TCONS_l2_00017174 | linc|BROAD Institute lincRNA (XLOC_l2_009163), lincRNA [TCONS_l2_00017174] |
| RP9 | NM_203288 | retinitis pigmentosa 9 (autosomal dominant) (RP9), mRNA [NM_203288] |
| XLOC_l2_015448 | ENST00000432442 | tc|GB|AK057918.1|AK057918.1 Homo sapiens cDNA FLJ25189 fis, clone CBR09529 [NP1154520] |
| XLOC_005937 | ENST00000457833 | linc|BROAD Institute lincRNA (XLOC_005937), lincRNA [TCONS_00011624] |
| TEX14 | NM_198393 | testis expressed 14 (TEX14), transcript variant 1, mRNA [NM_198393] |
| ENST00000355513 | ENST00000355513 | ens|chromosome 9 open reading frame 38 [Source:HGNC Symbol;Acc:23398] [ENST00000355513] |
| C16orf91 | NM_001010878 | chromosome 16 open reading frame 91 (C16orf91), mRNA [NM_001010878] |
| A_33_P3311308 | A_33_P3311308 | Unknown |
| FAM86DP | NR_024241 | family with sequence similarity 86, member D, pseudogene (FAM86DP), non-coding RNA [NR_024241] |
| XLOC_009383 | TCONS_00019585 | linc|BROAD Institute lincRNA (XLOC_009383), lincRNA [TCONS_00019585] |
| KCNK17 | NM_031460 | potassium channel, subfamily K, member 17 (KCNK17), transcript variant 1, mRNA [NM_031460] |
| SEC31A | NM_001077207 | SEC31 homolog A (S. cerevisiae) (SEC31A), transcript variant 5, mRNA [NM_001077207] |
| XLOC_004841 | TCONS_00010352 | linc|BROAD Institute lincRNA (XLOC_004841), lincRNA [TCONS_00010352] |
| XLOC_002204 | TCONS_00004331 | linc|BROAD Institute lincRNA (XLOC_002204), lincRNA [TCONS_00004331] |
| POMZP3 | NM_152992 | POM121 and ZP3 fusion (POMZP3), transcript variant 2, mRNA [NM_152992] |
| XLOC_005555 | ENST00000430250 | linc|BROAD Institute lincRNA (XLOC_005555), lincRNA [TCONS_00011423] |
| XLOC_007709 | ENST00000417850 | Unknown |
| PIK3R5 | NM_014308 | phosphoinositide-3-kinase, regulatory subunit 5 (PIK3R5), transcript variant 2, mRNA [NM_014308] |
| PLXNC1 | NM_005761 | plexin C1 (PLXNC1), transcript variant 1, mRNA [NM_005761] |
| XLOC_003702 | ENST00000507365 | tc|Q4NLF0_9MICC (Q4NLF0) Binding-protein-dependent transport systems inner membrane component, partial (7%) [THC2633405] |
| ENST00000456897 | ENST00000456897 | gb|Homo sapiens cDNA FLJ30558 fis, clone BRAWH2004044. [AK055120] |
| AIM2 | NM_004833 | absent in melanoma 2 (AIM2), mRNA [NM_004833] |
| LOC100506898 | XR_132625 | ref|PREDICTED: Homo sapiens protein mago nashi homolog 2-like (LOC100506898), miscRNA [XR_132625] |
| LOC100506895 | NR_038276 | uncharacterized LOC100506895 (LOC100506895), non-coding RNA [NR_038276] |
| GPKOW | NM_015698 | G patch domain and KOW motifs (GPKOW), mRNA [NM_015698] |
| SMEK3P | NR_002784 | SMEK homolog 3, suppressor of mek1 (Dictyostelium) pseudogene (SMEK3P), non-coding RNA [NR_002784] |
| A_33_P3391542 | A_33_P3391542 | Unknown |
| XLOC_001595 | TCONS_00003799 | linc|BROAD Institute lincRNA (XLOC_001595), lincRNA [TCONS_00003799] |
| A_33_P3249731 | A_33_P3249731 | Unknown |
| XLOC_006827 | THC2750669 | linc|BROAD Institute lincRNA (XLOC_006827), lincRNA [TCONS_00015291] |
| OR1G1 | NM_003555 | olfactory receptor, family 1, subfamily G, member 1 (OR1G1), mRNA [NM_003555] |
| XLOC_l2_011996 | TCONS_l2_00022844 | linc|BROAD Institute lincRNA (XLOC_l2_011996), lincRNA [TCONS_l2_00022844] |
| XLOC_005026 | TCONS_00010506 | linc|BROAD Institute lincRNA (XLOC_005026), lincRNA [TCONS_00010506] |
| CXCR6 | NM_006564 | chemokine (C-X-C motif) receptor 6 (CXCR6), mRNA [NM_006564] |
| PAX7 | NM_001135254 | paired box 7 (PAX7), transcript variant 3, mRNA [NM_001135254] |
| XLOC_007567 | ENST00000454034 | linc|BROAD Institute lincRNA (XLOC_007567), lincRNA [TCONS_00015753] |
| BRD7P3 | NR_002730 | bromodomain containing 7 pseudogene 3 (BRD7P3), non-coding RNA [NR_002730] |
| XLOC_l2_011620 | TCONS_l2_00023408 | linc|BROAD Institute lincRNA (XLOC_l2_011620), lincRNA [TCONS_l2_00023408] |
| GABRB2 | NM_021911 | gamma-aminobutyric acid (GABA) A receptor, beta 2 (GABRB2), transcript variant 1, mRNA [NM_021911] |
| RALGAPB | NM_020336 | Ral GTPase activating protein, beta subunit (non-catalytic) (RALGAPB), mRNA [NM_020336] |
| BHLHE22 | NM_152414 | basic helix-loop-helix family, member e22 (BHLHE22), mRNA [NM_152414] |
| SLC22A24 | NM_001136506 | solute carrier family 22, member 24 (SLC22A24), mRNA [NM_001136506] |
| XLOC_008604 | TCONS_00018323 | linc|BROAD Institute lincRNA (XLOC_008604), lincRNA [TCONS_00018323] |
| SNAI1 | NM_005985 | snail homolog 1 (Drosophila) (SNAI1), mRNA [NM_005985] |
| COIL | NM_004645 | coilin (COIL), mRNA [NM_004645] |
| XLOC_009340 | CD172981 | gb|AGENCOURT_13891730 NIH_MGC_172 Homo sapiens cDNA 5', mRNA sequence [CD172981] |
| XLOC_008233 | THC2697511 | linc|BROAD Institute lincRNA (XLOC_008233), lincRNA [TCONS_00017369] |
| TM4SF1 | NM_014220 | transmembrane 4 L six family member 1 (TM4SF1), mRNA [NM_014220] |
| XLOC_011603 | TCONS_00024213 | linc|BROAD Institute lincRNA (XLOC_011603), lincRNA [TCONS_00024213] |
| XLOC_003498 | TCONS_00008034 | linc|BROAD Institute lincRNA (XLOC_003498), lincRNA [TCONS_00008034] |
| XLOC_004300 | THC2691515 | tc|Q91YW8_MOUSE (Q91YW8) Pprc1 protein, partial (3%) [THC2691515] |
| XLOC_006898 | TCONS_00014543 | linc|BROAD Institute lincRNA (XLOC_006898), lincRNA [TCONS_00014543] |
| XLOC_012684 | THC2711140 | linc|BROAD Institute lincRNA (XLOC_012684), lincRNA [TCONS_00026337] |
| LOC642366 | CR936786 | tc|AY557971 YMR073C {Saccharomyces cerevisiae} (exp=-1; wgp=0; cg=0), partial (8%) [THC2618380] |
| ARL17A | NM_016632 | ADP-ribosylation factor-like 17A (ARL17A), transcript variant 2, mRNA [NM_016632] |
| DNAH11 | NM_003777 | dynein, axonemal, heavy chain 11 (DNAH11), mRNA [NM_003777] |
| DGKG | NM_001346 | diacylglycerol kinase, gamma 90kDa (DGKG), transcript variant 1, mRNA [NM_001346] |
| SDR9C7 | NM_148897 | short chain dehydrogenase/reductase family 9C, member 7 (SDR9C7), mRNA [NM_148897] |
| ENST00000371629 | ENST00000371629 | ens|chromosome 9 open reading frame 141 [Source:HGNC Symbol;Acc:18147] [ENST00000371629] |
| SCPEP1 | NM_021626 | serine carboxypeptidase 1 (SCPEP1), mRNA [NM_021626] |
| LOC100506860 | XR_108813 | ref|PREDICTED: Homo sapiens hypothetical LOC100506860, transcript variant 1 (LOC100506860), miscRNA [XR_108813] |
| HPX | NM_000613 | hemopexin (HPX), mRNA [NM_000613] |
| XLOC_002643 | ENST00000416209 | ref|PREDICTED: Homo sapiens hypothetical LOC100506637 (LOC100506637), miscRNA [XR_110001] |
| MRPL50 | NM_019051 | mitochondrial ribosomal protein L50 (MRPL50), nuclear gene encoding mitochondrial protein, mRNA [NM_019051] |
| XLOC_006385 | TCONS_00013741 | linc|BROAD Institute lincRNA (XLOC_006385), lincRNA [TCONS_00013741] |
| KIAA1731 | NM_033395 | KIAA1731 (KIAA1731), mRNA [NM_033395] |
| CNTN5 | NM_014361 | contactin 5 (CNTN5), transcript variant 1, mRNA [NM_014361] |
| B4GALNT3 | NM_173593 | beta-1,4-N-acetyl-galactosaminyl transferase 3 (B4GALNT3), mRNA [NM_173593] |
| CYP26A1 | NM_057157 | cytochrome P450, family 26, subfamily A, polypeptide 1 (CYP26A1), transcript variant 2, mRNA [NM_057157] |
| IGF1 | NM_000618 | insulin-like growth factor 1 (somatomedin C) (IGF1), transcript variant 4, mRNA [NM_000618] |
| FOLR2 | NM_000803 | folate receptor 2 (fetal) (FOLR2), transcript variant 1, mRNA [NM_000803] |
| A_33_P3365705 | A_33_P3365705 | Unknown |
| A_33_P3348927 | A_33_P3348927 | Unknown |
| IL3 | NM_000588 | interleukin 3 (colony-stimulating factor, multiple) (IL3), mRNA [NM_000588] |
| A_33_P3401394 | A_33_P3401394 | Unknown |
| CAPN8 | NM_001143962 | calpain 8 (CAPN8), mRNA [NM_001143962] |
| XLOC_006438 | ENST00000457592 | linc|BROAD Institute lincRNA (XLOC_006438), lincRNA [TCONS_00013168] |
| MCPH1 | NM_024596 | microcephalin 1 (MCPH1), transcript variant 1, mRNA [NM_024596] |
| ZNF185 | NM_001178106 | zinc finger protein 185 (LIM domain) (ZNF185), transcript variant 1, mRNA [NM_001178106] |
| SETD1B | NM_015048 | SET domain containing 1B (SETD1B), mRNA [NM_015048] |
| LOC728218 | BC065757 | gb|Homo sapiens cDNA clone IMAGE:4556546, partial cds. [BC065757] |
| ST8SIA5 | NM_013305 | ST8 alpha-N-acetyl-neuraminide alpha-2,8-sialyltransferase 5 (ST8SIA5), mRNA [NM_013305] |
| RYR2 | NM_001035 | ryanodine receptor 2 (cardiac) (RYR2), mRNA [NM_001035] |
| GDF6 | NM_001001557 | growth differentiation factor 6 (GDF6), mRNA [NM_001001557] |
| XLOC_006154 | ENST00000454234 | linc|BROAD Institute lincRNA (XLOC_006154), lincRNA [TCONS_00012883] |
| CCDC87 | NM_018219 | coiled-coil domain containing 87 (CCDC87), mRNA [NM_018219] |
| XLOC_005238 | TCONS_00012453 | linc|BROAD Institute lincRNA (XLOC_005238), lincRNA [TCONS_00012453] |
| THBS3 | CR933610 | gb|Homo sapiens mRNA; cDNA DKFZp686J1738 (from clone DKFZp686J1738). [CR933610] |
| XLOC_009437 | TCONS_00019635 | linc|BROAD Institute lincRNA (XLOC_009437), lincRNA [TCONS_00019635] |
| A_33_P3279388 | A_33_P3279388 | Unknown |
| XLOC_014161 | ENST00000444039 | linc|BROAD Institute lincRNA (XLOC_014161), lincRNA [TCONS_00029333] |
| LARP1B | NM_032239 | La ribonucleoprotein domain family, member 1B (LARP1B), transcript variant 3, mRNA [NM_032239] |
| XLOC_l2_011693 | ENST00000510435 | linc|BROAD Institute lincRNA (XLOC_l2_011693), lincRNA [TCONS_l2_00023491] |
| XLOC_l2_013485 | TCONS_l2_00026048 | linc|BROAD Institute lincRNA (XLOC_l2_013485), lincRNA [TCONS_l2_00026048] |
| LINC00158 | NR_024027 | long intergenic non-protein coding RNA 158 (LINC00158), non-coding RNA [NR_024027] |
| HAUS3 | NM_024511 | HAUS augmin-like complex, subunit 3 (HAUS3), mRNA [NM_024511] |
| XLOC_l2_012791 | TCONS_l2_00024511 | linc|BROAD Institute lincRNA (XLOC_l2_012791), lincRNA [TCONS_l2_00024511] |
| A_33_P3338071 | A_33_P3338071 | Unknown |
| QTRTD1 | NM_024638 | queuine tRNA-ribosyltransferase domain containing 1 (QTRTD1), mRNA [NM_024638] |
| ZMAT3 | NM_022470 | zinc finger, matrin-type 3 (ZMAT3), transcript variant 1, mRNA [NM_022470] |
| UNC5D | NM_080872 | unc-5 homolog D (C. elegans) (UNC5D), mRNA [NM_080872] |
| LOC100506073 | XM_003119000 | ref|PREDICTED: Homo sapiens hypothetical protein LOC100506073 (LOC100506073), mRNA [XM_003119000] |
| SCAF8 | NM_014892 | SR-related CTD-associated factor 8 (SCAF8), mRNA [NM_014892] |
| BCL6 | NM_001130845 | B-cell CLL/lymphoma 6 (BCL6), transcript variant 2, mRNA [NM_001130845] |
| DPYD | NM_000110 | dihydropyrimidine dehydrogenase (DPYD), transcript variant 1, mRNA [NM_000110] |
| NS3BP | XR_109071 | ref|PREDICTED: Homo sapiens NS3BP (NS3BP), miscRNA [XR_109071] |
| LOC100134015 | NR_038395 | uncharacterized LOC100134015 (LOC100134015), non-coding RNA [NR_038395] |
| SLC23A1 | NM_152685 | solute carrier family 23 (nucleobase transporters), member 1 (SLC23A1), transcript variant 2, mRNA [NM_152685] |
| XLOC_007930 | TCONS_00017140 | linc|BROAD Institute lincRNA (XLOC_007930), lincRNA [TCONS_00017140] |
| ENST00000390297 | ENST00000390297 | ens|immunoglobulin lambda variable 1-44 [Source:HGNC Symbol;Acc:5879] [ENST00000390297] |
| XLOC_009323 | TCONS_00020019 | linc|BROAD Institute lincRNA (XLOC_009323), lincRNA [TCONS_00020019] |
| X04925 | X04925 | gb|Human mRNA for T-cell receptor beta-chain (clone HBVP41) (Family Member: V beta 8.3). [X04925] |
| LOC283352 | AK097496 | gb|Homo sapiens cDNA FLJ40177 fis, clone TESTI2017816. [AK097496] |
| XLOC_l2_005130 | GD264120 | gb|de0_010084 SARS-Cov infected lung tissue Homo sapiens cDNA clone de0_010084 5', mRNA sequence [GD264120] |
| ART4 | NM_021071 | ADP-ribosyltransferase 4 (Dombrock blood group) (ART4), mRNA [NM_021071] |
| LOC100506187 | XM_003118527 | ref|PREDICTED: Homo sapiens hypothetical protein LOC100506187 (LOC100506187), mRNA [XM_003118527] |
| KRT19 | NM_002276 | keratin 19 (KRT19), mRNA [NM_002276] |
| AATK | NM_001080395 | apoptosis-associated tyrosine kinase (AATK), transcript variant 1, mRNA [NM_001080395] |
| APOH | NM_000042 | apolipoprotein H (beta-2-glycoprotein I) (APOH), mRNA [NM_000042] |
| LOC100506291 | XR_110464 | ref|PREDICTED: Homo sapiens hypothetical LOC100506291, transcript variant 1 (LOC100506291), miscRNA [XR_110464] |
| XLOC_003449 | TCONS_00007979 | linc|BROAD Institute lincRNA (XLOC_003449), lincRNA [TCONS_00007979] |
| USH1C | NM_005709 | Usher syndrome 1C (autosomal recessive, severe) (USH1C), transcript variant 1, mRNA [NM_005709] |
| ALOX5 | ENST00000475300 | ens|arachidonate 5-lipoxygenase [Source:HGNC Symbol;Acc:435] [ENST00000475300] |
| SUFU | NM_016169 | suppressor of fused homolog (Drosophila) (SUFU), transcript variant 1, mRNA [NM_016169] |
| XLOC_008721 | TCONS_00018426 | linc|BROAD Institute lincRNA (XLOC_008721), lincRNA [TCONS_00018426] |
| A_33_P3340454 | A_33_P3340454 | Unknown |
| SI | NM_001041 | sucrase-isomaltase (alpha-glucosidase) (SI), mRNA [NM_001041] |
| XLOC_009301 | TCONS_00019500 | linc|BROAD Institute lincRNA (XLOC_009301), lincRNA [TCONS_00019500] |
| SP9 | ENST00000394967 | ens|Sp9 transcription factor homolog (mouse) [Source:HGNC Symbol;Acc:30690] [ENST00000394967] |
| XLOC_002952 | TCONS_00006334 | linc|BROAD Institute lincRNA (XLOC_002952), lincRNA [TCONS_00006334] |
| LOC100505588 | XR_108995 | ref|PREDICTED: Homo sapiens hypothetical LOC100505588 (LOC100505588), miscRNA [XR_108995] |
| LOC100506189 | XR_108925 | ref|PREDICTED: Homo sapiens hypothetical LOC100506189 (LOC100506189), miscRNA [XR_108925] |
| XLOC_012348 | TCONS_00026002 | linc|BROAD Institute lincRNA (XLOC_012348), lincRNA [TCONS_00026002] |
| PCBP1 | NM_006196 | poly(rC) binding protein 1 (PCBP1), mRNA [NM_006196] |
| SLC22A9 | NM_080866 | solute carrier family 22 (organic anion transporter), member 9 (SLC22A9), mRNA [NM_080866] |
| XLOC_006321 | TCONS_00013660 | linc|BROAD Institute lincRNA (XLOC_006321), lincRNA [TCONS_00013660] |
| XLOC_010922 | TCONS_00022622 | linc|BROAD Institute lincRNA (XLOC_010922), lincRNA [TCONS_00022622] |
| ATP1A1 | ENST00000369494 | ens|ATPase, Na+/K+ transporting, alpha 1 polypeptide [Source:HGNC Symbol;Acc:799] [ENST00000369494] |
| XLOC_012315 | TCONS_00025500 | linc|BROAD Institute lincRNA (XLOC_012315), lincRNA [TCONS_00025500] |
| XLOC_006037 | THC2573057 | linc|BROAD Institute lincRNA (XLOC_006037), lincRNA [TCONS_00012989] |
| YTHDF1 | NM_017798 | YTH domain family, member 1 (YTHDF1), mRNA [NM_017798] |
| XLOC_002400 | AK057534 | linc|BROAD Institute lincRNA (XLOC_002400), lincRNA [TCONS_00003441] |
| THC2633118 | THC2633118 | tc|Q6GYI9_HHV1 (Q6GYI9) Tegument protein VP22, partial (5%) [THC2633118] |
| POLE | NM_006231 | polymerase (DNA directed), epsilon (POLE), mRNA [NM_006231] |
| ENST00000409758 | ENST00000409758 | Unknown |
| POU5F1 | NM_002701 | POU class 5 homeobox 1 (POU5F1), transcript variant 1, mRNA [NM_002701] |
| GCNT7 | NM_080615 | glucosaminyl (N-acetyl) transferase family member 7 (GCNT7), mRNA [NM_080615] |
| OVOL2 | NM_021220 | ovo-like 2 (Drosophila) (OVOL2), mRNA [NM_021220] |
| XLOC_007035 | TCONS_00014956 | linc|BROAD Institute lincRNA (XLOC_007035), lincRNA [TCONS_00014956] |
| XLOC_004467 | TCONS_00010037 | linc|BROAD Institute lincRNA (XLOC_004467), lincRNA [TCONS_00010037] |
| FLG | NM_002016 | filaggrin (FLG), mRNA [NM_002016] |
| XLOC_l2_007135 | TCONS_l2_00013242 | linc|BROAD Institute lincRNA (XLOC_l2_007135), lincRNA [TCONS_l2_00013242] |
| ATP11A | NM_015205 | ATPase, class VI, type 11A (ATP11A), transcript variant 1, mRNA [NM_015205] |
| LCLAT1 | ENST00000319406 | ens|lysocardiolipin acyltransferase 1 [Source:HGNC Symbol;Acc:26756] [ENST00000319406] |
| XLOC_012333 | TCONS_00025520 | linc|BROAD Institute lincRNA (XLOC_012333), lincRNA [TCONS_00025520] |
| LCE1C | NM_178351 | late cornified envelope 1C (LCE1C), mRNA [NM_178351] |
| CNOT8 | NM_004779 | CCR4-NOT transcription complex, subunit 8 (CNOT8), mRNA [NM_004779] |
| SNORD111 | NR_003079 | small nucleolar RNA, C/D box 111 (SNORD111), small nucleolar RNA [NR_003079] |
| THC2719114 | THC2719114 | tc|Q7TP87_RAT (Q7TP87) Ab1-233, partial (8%) [THC2719114] |
| LOC100288911 | NR_037631 | uncharacterized LOC100288911 (LOC100288911), non-coding RNA [NR_037631] |
| BLM | NM_000057 | Bloom syndrome, RecQ helicase-like (BLM), mRNA [NM_000057] |
| XLOC_005062 | TCONS_00010536 | linc|BROAD Institute lincRNA (XLOC_005062), lincRNA [TCONS_00010536] |
| XLOC_000909 | TCONS_00001570 | linc|BROAD Institute lincRNA (XLOC_000909), lincRNA [TCONS_00001570] |
| CHST7 | NM_019886 | carbohydrate (N-acetylglucosamine 6-O) sulfotransferase 7 (CHST7), mRNA [NM_019886] |
| ENST00000424895 | ENST00000424895 | gb|Homo sapiens cDNA FLJ41481 fis, clone BRTHA2002721. [AK123475] |
| SIRT5 | NM_031244 | sirtuin 5 (SIRT5), transcript variant 2, mRNA [NM_031244] |
| MARK3 | NM_001128918 | MAP/microtubule affinity-regulating kinase 3 (MARK3), transcript variant 1, mRNA [NM_001128918] |
| LOC100499467 | NR_036488 | uncharacterized LOC100499467 (LOC100499467), non-coding RNA [NR_036488] |
| CCDC17 | NM_001114938 | coiled-coil domain containing 17 (CCDC17), transcript variant 1, mRNA [NM_001114938] |
| ENST00000556583 | ENST00000556583 | Unknown |
| RABGAP1 | ENST00000317419 | ens|RAB GTPase activating protein 1 [Source:HGNC Symbol;Acc:17155] [ENST00000317419] |
| CSTL1 | NM_138283 | cystatin-like 1 (CSTL1), mRNA [NM_138283] |
| ZNF805 | NM_001023563 | zinc finger protein 805 (ZNF805), transcript variant 1, mRNA [NM_001023563] |
| MTO1 | NM_012123 | mitochondrial translation optimization 1 homolog (S. cerevisiae) (MTO1), nuclear gene encoding mitochondrial protein, transcript variant 2, mRNA [NM_012123] |
| HMGCS2 | NM_005518 | 3-hydroxy-3-methylglutaryl-CoA synthase 2 (mitochondrial) (HMGCS2), nuclear gene encoding mitochondrial protein, transcript variant 1, mRNA [NM_005518] |
| SORCS3 | NM_014978 | sortilin-related VPS10 domain containing receptor 3 (SORCS3), mRNA [NM_014978] |
| ARMCX5 | NM_022838 | armadillo repeat containing, X-linked 5 (ARMCX5), transcript variant 2, mRNA [NM_022838] |
| LOC100128098 | NR_034129 | uncharacterized LOC100128098 (LOC100128098), non-coding RNA [NR_034129] |
| SLC4A8 | NM_004858 | solute carrier family 4, sodium bicarbonate cotransporter, member 8 (SLC4A8), transcript variant 2, mRNA [NM_004858] |
| LOC728323 | NR_024437 | uncharacterized LOC728323 (LOC728323), non-coding RNA [NR_024437] |
| LOC100506957 | ENST00000419211 | ref|PREDICTED: Homo sapiens hypothetical LOC100506957 (LOC100506957), miscRNA [XR_108821] |
| XLOC_l2_005804 | EB385853 | gb|nbj08c11.y1 Human optic nerve. Unnormalized (nbj) Homo sapiens cDNA clone nbj08c11 5', mRNA sequence [EB385853] |
| CMPK2 | BC141802 | gb|Homo sapiens cytidine monophosphate (UMP-CMP) kinase 2, mitochondrial, mRNA (cDNA clone MGC:164756 IMAGE:40147165), complete cds. [BC141802] |
| XLOC_011659 | TCONS_00024341 | linc|BROAD Institute lincRNA (XLOC_011659), lincRNA [TCONS_00024341] |
| SYK | NM_003177 | spleen tyrosine kinase (SYK), transcript variant 1, mRNA [NM_003177] |
| CDK5R1 | NM_003885 | cyclin-dependent kinase 5, regulatory subunit 1 (p35) (CDK5R1), mRNA [NM_003885] |
| LOC100509620 | XM_003119862 | ref|PREDICTED: Homo sapiens aquaporin-7-like, transcript variant 1 (LOC100509620), mRNA [XM_003119862] |
| SGPP2 | NM_152386 | sphingosine-1-phosphate phosphatase 2 (SGPP2), mRNA [NM_152386] |
| BJ995728 | BJ995728 | gb|BJ995728 human hepatoblastoma cDNA Homo sapiens cDNA clone hmft-3130 5', mRNA sequence [BJ995728] |
| LOC100507254 | NR_038981 | uncharacterized LOC100507254 (LOC100507254), non-coding RNA [NR_038981] |
| SLC24A2 | NM_020344 | solute carrier family 24 (sodium/potassium/calcium exchanger), member 2 (SLC24A2), transcript variant 1, mRNA [NM_020344] |
| C6orf228 | NM_001135575 | chromosome 6 open reading frame 228 (C6orf228), mRNA [NM_001135575] |
| SPANXN3 | NM_001009609 | SPANX family, member N3 (SPANXN3), mRNA [NM_001009609] |
| A_33_P3312209 | A_33_P3312209 | Unknown |
| PLXDC1 | NM_020405 | plexin domain containing 1 (PLXDC1), mRNA [NM_020405] |
| MS4A13 | NM_001012417 | membrane-spanning 4-domains, subfamily A, member 13 (MS4A13), transcript variant 1, mRNA [NM_001012417] |
| HAS1 | NM_001523 | hyaluronan synthase 1 (HAS1), mRNA [NM_001523] |
| XLOC_l2_014771 | DN914066 | gb|MCF7RNAL18A23TF Human MCF7 breast cancer cell line near full length normalized library (MCF7_EST) Homo sapiens cDNA clone MCF7_RNA_L_18_A23, mRNA sequence [DN914066] |
| PRDM15 | NM_001040424 | PR domain containing 15 (PRDM15), transcript variant 2, mRNA [NM_001040424] |
| ROR1 | NM_005012 | receptor tyrosine kinase-like orphan receptor 1 (ROR1), transcript variant 1, mRNA [NM_005012] |
| CLC | NM_001828 | Charcot-Leyden crystal protein (CLC), mRNA [NM_001828] |
| TAS2R50 | NM_176890 | taste receptor, type 2, member 50 (TAS2R50), mRNA [NM_176890] |
| LOC646813 | NR_024504 | DEAH (Asp-Glu-Ala-His) box polypeptide 9 pseudogene (LOC646813), non-coding RNA [NR_024504] |
| GYPB | NM_002100 | glycophorin B (MNS blood group) (GYPB), mRNA [NM_002100] |
| ANKRD22 | NM_144590 | ankyrin repeat domain 22 (ANKRD22), mRNA [NM_144590] |
| LOC100506877 | XR_112264 | ref|PREDICTED: Homo sapiens hypothetical LOC100506877 (LOC100506877), miscRNA [XR_112264] |
| TEKT5 | NM_144674 | tektin 5 (TEKT5), mRNA [NM_144674] |
| GNE | NM_005476 | glucosamine (UDP-N-acetyl)-2-epimerase/N-acetylmannosamine kinase (GNE), transcript variant 2, mRNA [NM_005476] |
| AB088847 | AB088847 | gb|Homo sapiens BPA-1 mRNA for brain peptide A1, complete cds. [AB088847] |
| ELMO2 | NM_182764 | engulfment and cell motility 2 (ELMO2), transcript variant 2, mRNA [NM_182764] |
| LOC731789 | NR_026794 | uncharacterized LOC731789 (LOC731789), non-coding RNA [NR_026794] |
| FGF22 | NM_020637 | fibroblast growth factor 22 (FGF22), mRNA [NM_020637] |
| ARMC3 | NM_173081 | armadillo repeat containing 3 (ARMC3), mRNA [NM_173081] |
| PCDH10 | NM_032961 | protocadherin 10 (PCDH10), transcript variant 1, mRNA [NM_032961] |
| SPTA1 | NM_003126 | spectrin, alpha, erythrocytic 1 (elliptocytosis 2) (SPTA1), mRNA [NM_003126] |
| COL15A1 | NM_001855 | collagen, type XV, alpha 1 (COL15A1), mRNA [NM_001855] |
| XLOC_012689 | TCONS_00026627 | linc|BROAD Institute lincRNA (XLOC_012689), lincRNA [TCONS_00026627] |
| XLOC_004725 | TCONS_00010259 | linc|BROAD Institute lincRNA (XLOC_004725), lincRNA [TCONS_00010259] |
| SH3RF3 | NM_001099289 | SH3 domain containing ring finger 3 (SH3RF3), mRNA [NM_001099289] |
| XLOC_010681 | TCONS_00022079 | linc|BROAD Institute lincRNA (XLOC_010681), lincRNA [TCONS_00022079] |
| XLOC_l2_009456 | ENST00000425895 | tc|GGT1_HUMAN Gamma-glutamyltranspeptidase 1 precursor (Gamma-glutamyltransferase 1) (CD224 antigen) [Contains: Gamma-glutamyltranspeptidase 1 heavy chain; Gamma-glutamyltranspeptidase 1light chain]. {Homo sapiens} (exp=-1; wgp=-1; cg=-1) , complete [THC |
| LOC283404 | NR_027358 | uncharacterized LOC283404 (LOC283404), non-coding RNA [NR_027358] |
| XLOC_008777 | ENST00000450106 | tc|AC007764 F22C12.23 {Arabidopsis thaliana} (exp=0; wgp=1; cg=0), partial (6%) [THC2737253] |
| A_33_P3259750 | A_33_P3259750 | Unknown |
| COPA | NM_001098398 | coatomer protein complex, subunit alpha (COPA), transcript variant 1, mRNA [NM_001098398] |
| MTM1 | NM_000252 | myotubularin 1 (MTM1), mRNA [NM_000252] |
| AHNAK | NM_001620 | AHNAK nucleoprotein (AHNAK), transcript variant 1, mRNA [NM_001620] |
| LOC100131719 | AK128441 | gb|Homo sapiens cDNA FLJ46584 fis, clone THYMU3043688. [AK128441] |
| TBP | NM_003194 | TATA box binding protein (TBP), transcript variant 1, mRNA [NM_003194] |
| IL3RA | NM_002183 | interleukin 3 receptor, alpha (low affinity) (IL3RA), mRNA [NM_002183] |
| FAM87B | XR_112085 | ref|PREDICTED: Homo sapiens family with sequence similarity 87, member B (FAM87B), miscRNA [XR_112085] |
| C17orf6 | AJ272176 | gb|Homo sapiens partial mRNA, clone cha12. [AJ272176] |
| LYZL4 | NM_144634 | lysozyme-like 4 (LYZL4), mRNA [NM_144634] |
| LOC100509763 | XM_003119857 | ref|PREDICTED: Homo sapiens hypothetical protein LOC100509763 (LOC100509763), mRNA [XM_003119857] |
| METTL11B | NM_001136107 | methyltransferase like 11B (METTL11B), mRNA [NM_001136107] |
| FAM5C | NM_199051 | family with sequence similarity 5, member C (FAM5C), mRNA [NM_199051] |
| C17orf66 | NM_152781 | chromosome 17 open reading frame 66 (C17orf66), mRNA [NM_152781] |
| XLOC_l2_012135 | ENST00000505712 | tc|Q9BVX4_HUMAN (Q9BVX4) MGC5566 protein, partial (19%) [THC2690938] |
| XLOC_012725 | TCONS_00026374 | linc|BROAD Institute lincRNA (XLOC_012725), lincRNA [TCONS_00026374] |
| FAM159A | ENST00000440303 | ens|family with sequence similarity 159, member A [Source:HGNC Symbol;Acc:28757] [ENST00000440303] |
| TEX15 | NM_031271 | testis expressed 15 (TEX15), mRNA [NM_031271] |
| KIAA1644 | NM_001099294 | KIAA1644 (KIAA1644), mRNA [NM_001099294] |
| A_33_P3333327 | A_33_P3333327 | Unknown |
| C12orf47 | NR_015404 | chromosome 12 open reading frame 47 (C12orf47), non-coding RNA [NR_015404] |
| XLOC_002176 | TCONS_00004316 | linc|BROAD Institute lincRNA (XLOC_002176), lincRNA [TCONS_00004316] |
| TPK1 | NM_022445 | thiamin pyrophosphokinase 1 (TPK1), transcript variant 1, mRNA [NM_022445] |
| XLOC_009123 | ENST00000532307 | ref|PREDICTED: Homo sapiens hypothetical LOC100507384 (LOC100507384), miscRNA [XR_111174] |
| XLOC_001470 | ENST00000418451 | linc|BROAD Institute lincRNA (XLOC_001470), lincRNA [TCONS_00002893] |
| TMEM86A | NM_153347 | transmembrane protein 86A (TMEM86A), mRNA [NM_153347] |
| XLOC_006777 | ENST00000519063 | tc|Q28483_MACFA (Q28483) TMDC II protein, partial (22%) [THC2671138] |
| XLOC_009085 | TCONS_00019254 | linc|BROAD Institute lincRNA (XLOC_009085), lincRNA [TCONS_00019254] |
| XLOC_l2_001037 | TCONS_l2_00001430 | linc|BROAD Institute lincRNA (XLOC_l2_001037), lincRNA [TCONS_l2_00001430] |
| XLOC_004171 | ENST00000504167 | linc|BROAD Institute lincRNA (XLOC_004171), lincRNA [TCONS_00007904] |
| LOC100505835 | NR_040036 | uncharacterized LOC100505835 (LOC100505835), non-coding RNA [NR_040036] |
| BC013798 | BC013798 | gb|Homo sapiens cDNA clone IMAGE:4341068, **** WARNING: chimeric clone ****. [BC013798] |
| XLOC_003477 | ENST00000515188 | gb|RST23693 Athersys RAGE Library Homo sapiens cDNA, mRNA sequence [BG204292] |
| ENST00000367119 | ENST00000367119 | ens|chromosome 1 open reading frame 147 [Source:HGNC Symbol;Acc:32061] [ENST00000367119] |
| AY358815 | AY358815 | gb|Homo sapiens clone DNA108923 SFVP2550 (UNQ2550) mRNA, complete cds. [AY358815] |
| Q4KDJ5 | AK095699 | tc|Q4KDJ5_PSEF5 (Q4KDJ5) Drug resistance transporter, Bcr/CflA subfamily CC1230, partial (6%) [THC2621371] |
| SNX6 | NM_021249 | sorting nexin 6 (SNX6), transcript variant 1, mRNA [NM_021249] |
| XLOC_l2_008639 | ENST00000417630 | gb|BX112189 NCI_CGAP_GC6 Homo sapiens cDNA clone IMAGp998C085723, mRNA sequence [BX112189] |
| FP6628 | XR_108867 | ref|PREDICTED: Homo sapiens hypothetical LOC100132183 (FP6628), miscRNA [XR_108867] |
| XLOC_005589 | TCONS_00012648 | linc|BROAD Institute lincRNA (XLOC_005589), lincRNA [TCONS_00012648] |
| LOC728716 | NR_040065 | uncharacterized LOC728716 (LOC728716), non-coding RNA [NR_040065] |
| A_33_P3240158 | A_33_P3240158 | Unknown |
| THC2610631 | THC2610631 | Unknown |
| XLOC_012170 | ENST00000444464 | ref|PREDICTED: Homo sapiens hypothetical LOC100506643 (LOC100506643), miscRNA [XR_109456] |
| XLOC_l2_012661 | TCONS_l2_00024354 | linc|BROAD Institute lincRNA (XLOC_l2_012661), lincRNA [TCONS_l2_00024354] |
| PDE3B | NM_000922 | phosphodiesterase 3B, cGMP-inhibited (PDE3B), mRNA [NM_000922] |
| XLOC_004207 | CR936814 | linc|BROAD Institute lincRNA (XLOC_004207), lincRNA [TCONS_00009192] |
| OR6C4 | NM_001005494 | olfactory receptor, family 6, subfamily C, member 4 (OR6C4), mRNA [NM_001005494] |
| FLJ35424 | NR_040045 | uncharacterized FLJ35424 (FLJ35424), non-coding RNA [NR_040045] |
| ENST00000419160 | ENST00000419160 | tc|ALU1_HUMAN (P39188) Alu subfamily J sequence contamination warning entry, partial (13%) [THC2565852] |
| XLOC_001035 | THC2507453 | linc|BROAD Institute lincRNA (XLOC_001035), lincRNA [TCONS_00002485] |
| XLOC_007966 | TCONS_00017158 | linc|BROAD Institute lincRNA (XLOC_007966), lincRNA [TCONS_00017158] |
| LOC100505827 | XR_109685 | ref|PREDICTED: Homo sapiens hypothetical LOC100505827 (LOC100505827), miscRNA [XR_109685] |
| LOC100507206 | AY070435 | tc|LEAGP1G arabinogalactan {Lycopersicon esculentum} (exp=-1; wgp=0; cg=0), partial (10%) [THC2645249] |
| SERAC1 | ENST00000367101 | ens|serine active site containing 1 [Source:HGNC Symbol;Acc:21061] [ENST00000367101] |
| LOC415056 | NR_024369 | uncharacterized LOC415056 (LOC415056), non-coding RNA [NR_024369] |
| LOC100653014 | XR_132799 | ref|PREDICTED: Homo sapiens hypothetical LOC100653014 (LOC100653014), miscRNA [XR_132799] |
| S100A2 | NM_005978 | S100 calcium binding protein A2 (S100A2), mRNA [NM_005978] |
| MYLIP | NM_013262 | myosin regulatory light chain interacting protein (MYLIP), mRNA [NM_013262] |
| XLOC_007595 | TCONS_00016205 | linc|BROAD Institute lincRNA (XLOC_007595), lincRNA [TCONS_00016205] |
| PAFAH1B2 | NM_002572 | platelet-activating factor acetylhydrolase 1b, catalytic subunit 2 (30kDa) (PAFAH1B2), transcript variant 1, mRNA [NM_002572] |
| XLOC_007150 | TCONS_00015086 | linc|BROAD Institute lincRNA (XLOC_007150), lincRNA [TCONS_00015086] |
| XLOC_006338 | TCONS_00013676 | linc|BROAD Institute lincRNA (XLOC_006338), lincRNA [TCONS_00013676] |
| XLOC_000724 | TCONS_00001409 | linc|BROAD Institute lincRNA (XLOC_000724), lincRNA [TCONS_00001409] |
| XLOC_007486 | TCONS_00016095 | linc|BROAD Institute lincRNA (XLOC_007486), lincRNA [TCONS_00016095] |
| RAPH1 | NM_213589 | Ras association (RalGDS/AF-6) and pleckstrin homology domains 1 (RAPH1), transcript variant 1, mRNA [NM_213589] |
| ACRC | NM_052957 | acidic repeat containing (ACRC), mRNA [NM_052957] |
| XLOC_010805 | TCONS_00022477 | linc|BROAD Institute lincRNA (XLOC_010805), lincRNA [TCONS_00022477] |
| B3GAT1 | NM_054025 | beta-1,3-glucuronyltransferase 1 (glucuronosyltransferase P) (B3GAT1), transcript variant 2, mRNA [NM_054025] |
| LGALS3 | NM_002306 | lectin, galactoside-binding, soluble, 3 (LGALS3), transcript variant 1, mRNA [NM_002306] |
| SCG5 | NM_003020 | secretogranin V (7B2 protein) (SCG5), transcript variant 2, mRNA [NM_003020] |
| DEFB107A | NM_001037668 | defensin, beta 107A (DEFB107A), mRNA [NM_001037668] |
| AGAP1 | NM_001037131 | ArfGAP with GTPase domain, ankyrin repeat and PH domain 1 (AGAP1), transcript variant 1, mRNA [NM_001037131] |
| MGC15705 | BC007304 | gb|Homo sapiens hypothetical protein MGC15705, mRNA (cDNA clone MGC:15705 IMAGE:3352097), complete cds. [BC007304] |
| EDN1 | NM_001955 | endothelin 1 (EDN1), transcript variant 1, mRNA [NM_001955] |
| FLJ44124 | AK126112 | gb|Homo sapiens cDNA FLJ44124 fis, clone THYMU2005321. [AK126112] |
| XLOC_001435 | TCONS_00004755 | linc|BROAD Institute lincRNA (XLOC_001435), lincRNA [TCONS_00004755] |
| SLC4A10 | NM_022058 | solute carrier family 4, sodium bicarbonate transporter, member 10 (SLC4A10), transcript variant 2, mRNA [NM_022058] |
| XLOC_001943 | TCONS_00004098 | linc|BROAD Institute lincRNA (XLOC_001943), lincRNA [TCONS_00004098] |
| XLOC_002099 | TCONS_00003286 | linc|BROAD Institute lincRNA (XLOC_002099), lincRNA [TCONS_00003286] |
| XLOC_008025 | TCONS_00017209 | linc|BROAD Institute lincRNA (XLOC_008025), lincRNA [TCONS_00017209] |
| GH2 | NM_022558 | growth hormone 2 (GH2), transcript variant 3, mRNA [NM_022558] |
| LOC100506834 | XR_108969 | ref|PREDICTED: Homo sapiens hypothetical LOC100506834 (LOC100506834), miscRNA [XR_108969] |
| AS3MT | NM_020682 | arsenic (+3 oxidation state) methyltransferase (AS3MT), mRNA [NM_020682] |
| XLOC_l2_013751 | ENST00000423689 | linc|BROAD Institute lincRNA (XLOC_l2_013751), lincRNA [TCONS_l2_00026436] |
| TIGD6 | NM_030953 | tigger transposable element derived 6 (TIGD6), transcript variant 1, mRNA [NM_030953] |
| XLOC_009272 | THC2732835 | linc|BROAD Institute lincRNA (XLOC_009272), lincRNA [TCONS_00019459] |
| LHCGR | ENST00000477576 | ens|luteinizing hormone/choriogonadotropin receptor [Source:HGNC Symbol;Acc:6585] [ENST00000477576] |
| PPP1R36 | NM_172365 | protein phosphatase 1, regulatory subunit 36 (PPP1R36), mRNA [NM_172365] |
| THC2680667 | THC2680667 | tc|Q9BVX4_HUMAN (Q9BVX4) MGC5566 protein, partial (23%) [THC2680667] |
| VGLL1 | NM_016267 | vestigial like 1 (Drosophila) (VGLL1), mRNA [NM_016267] |
| BX114156 | BX114156 | gb|BX114156 Soares_testis_NHT Homo sapiens cDNA clone IMAGp998K154456, mRNA sequence [BX114156] |
| C9orf68 | ENST00000471669 | ens|chromosome 9 open reading frame 68 [Source:HGNC Symbol;Acc:25472] [ENST00000471669] |
| XLOC_002035 | ENST00000452212 | ref|PREDICTED: Homo sapiens hypothetical LOC100505774 (LOC100505774), miscRNA [XR_109967] |
| XLOC_000710 | ENST00000447908 | linc|BROAD Institute lincRNA (XLOC_000710), lincRNA [TCONS_00000472] |
| A_33_P3212052 | A_33_P3212052 | Unknown |
| FAM184B | NM_015688 | family with sequence similarity 184, member B (FAM184B), mRNA [NM_015688] |
| LOC100129884 | AK097079 | gb|Homo sapiens cDNA FLJ39760 fis, clone SMINT2019200. [AK097079] |
| LOC283483 | XR_109153 | ref|PREDICTED: Homo sapiens hypothetical LOC283483 (LOC283483), miscRNA [XR_109153] |
| XLOC_006348 | TCONS_00013686 | linc|BROAD Institute lincRNA (XLOC_006348), lincRNA [TCONS_00013686] |
| AQP9 | NM_020980 | aquaporin 9 (AQP9), mRNA [NM_020980] |
| XLOC_007432 | TCONS_00016037 | linc|BROAD Institute lincRNA (XLOC_007432), lincRNA [TCONS_00016037] |
| P39188 | THC2579385 | tc|ALU1_HUMAN (P39188) Alu subfamily J sequence contamination warning entry, partial (6%) [THC2579385] |
| MUC3A | XM_003403552 | ref|PREDICTED: Homo sapiens mucin 3A, cell surface associated (MUC3A), mRNA [XM_003403552] |
| LOC100130051 | AK127296 | gb|Homo sapiens cDNA FLJ45363 fis, clone BRHIP3015854. [AK127296] |
| MXRA7 | NM_001008528 | matrix-remodelling associated 7 (MXRA7), transcript variant 1, mRNA [NM_001008528] |
| RAD51AP2 | NM_001099218 | RAD51 associated protein 2 (RAD51AP2), mRNA [NM_001099218] |
| LOC100506777 | XR_110232 | ref|PREDICTED: Homo sapiens hypothetical LOC100506777 (LOC100506777), miscRNA [XR_110232] |
| ANXA8L2 | NM_001630 | annexin A8-like 2 (ANXA8L2), mRNA [NM_001630] |
| KDM4D | NM_018039 | lysine (K)-specific demethylase 4D (KDM4D), mRNA [NM_018039] |
| SNORD115-23 | NR_003315 | small nucleolar RNA, C/D box 115-23 (SNORD115-23), small nucleolar RNA [NR_003315] |
| XLOC_000680 | ENST00000457440 | linc|BROAD Institute lincRNA (XLOC_000680), lincRNA [TCONS_00000457] |
| XLOC_l2_000324 | TCONS_l2_00000436 | linc|BROAD Institute lincRNA (XLOC_l2_000324), lincRNA [TCONS_l2_00000436] |
| FASTKD5 | NM_021826 | FAST kinase domains 5 (FASTKD5), mRNA [NM_021826] |
| LOC100505921 | XR_108739 | ref|PREDICTED: Homo sapiens hypothetical LOC100505921 (LOC100505921), miscRNA [XR_108739] |
| XLOC_003485 | TCONS_00008021 | linc|BROAD Institute lincRNA (XLOC_003485), lincRNA [TCONS_00008021] |
| RSU1 | NM_012425 | Ras suppressor protein 1 (RSU1), transcript variant 1, mRNA [NM_012425] |
| XLOC_010357 | DB084706 | gb|DB084706 TESTI4 Homo sapiens cDNA clone TESTI4031748 5', mRNA sequence [DB084706] |
| LOC100129888 | AK127110 | gb|Homo sapiens cDNA FLJ45167 fis, clone BRAWH3044676. [AK127110] |
| ZNF193 | NM_006299 | zinc finger protein 193 (ZNF193), transcript variant 2, mRNA [NM_006299] |
| CAMKK1 | NM_032294 | calcium/calmodulin-dependent protein kinase kinase 1, alpha (CAMKK1), transcript variant 1, mRNA [NM_032294] |
| IL25 | NM_022789 | interleukin 25 (IL25), transcript variant 1, mRNA [NM_022789] |
| CYR61 | NM_001554 | cysteine-rich, angiogenic inducer, 61 (CYR61), mRNA [NM_001554] |
| CTNNB1 | NM_001904 | catenin (cadherin-associated protein), beta 1, 88kDa (CTNNB1), transcript variant 1, mRNA [NM_001904] |
| XLOC_001475 | TCONS_00003681 | linc|BROAD Institute lincRNA (XLOC_001475), lincRNA [TCONS_00003681] |
| RIPK3 | NM_006871 | receptor-interacting serine-threonine kinase 3 (RIPK3), mRNA [NM_006871] |
| ATF7 | NM_006856 | activating transcription factor 7 (ATF7), transcript variant 2, mRNA [NM_006856] |
| FAM71C | NM_153364 | family with sequence similarity 71, member C (FAM71C), mRNA [NM_153364] |
| ADORA2A | NM_000675 | adenosine A2a receptor (ADORA2A), mRNA [NM_000675] |
| Q2I700 | ENST00000417485 | tc|Q2I700_9GAST (Q2I700) NADH dehydrogenase subunit 1, partial (7%) [THC2764903] |
| A_33_P3254580 | A_33_P3254580 | Unknown |
| MASP2 | NM_006610 | mannan-binding lectin serine peptidase 2 (MASP2), transcript variant 1, mRNA [NM_006610] |
| MCHR2 | NM_001040179 | melanin-concentrating hormone receptor 2 (MCHR2), transcript variant 1, mRNA [NM_001040179] |
| C11orf82 | NM_145018 | chromosome 11 open reading frame 82 (C11orf82), mRNA [NM_145018] |
| XLOC_008895 | TCONS_00018569 | linc|BROAD Institute lincRNA (XLOC_008895), lincRNA [TCONS_00018569] |
| LOC100506982 | XR_111219 | ref|PREDICTED: Homo sapiens hypothetical LOC100506982 (LOC100506982), miscRNA [XR_111219] |
| A_33_P3379776 | A_33_P3379776 | Unknown |
| LOC286184 | NR_038875 | uncharacterized LOC286184 (LOC286184), non-coding RNA [NR_038875] |
| GOLGA3 | NM_001172557 | golgin A3 (GOLGA3), transcript variant 2, mRNA [NM_001172557] |
| MPRIP | ENST00000395807 | ens|myosin phosphatase Rho interacting protein [Source:HGNC Symbol;Acc:30321] [ENST00000395807] |
| SMCR5 | NR_024007 | Smith-Magenis syndrome chromosome region, candidate 5 (non-protein coding) (SMCR5), non-coding RNA [NR_024007] |
| LOC100509507 | XR_133082 | ref|PREDICTED: Homo sapiens putative uncharacterized protein C14orf165-like (LOC100509507), miscRNA [XR_133082] |
| XLOC_006035 | TCONS_00013404 | linc|BROAD Institute lincRNA (XLOC_006035), lincRNA [TCONS_00013404] |
| NKAIN2 | NM_001040214 | Na+/K+ transporting ATPase interacting 2 (NKAIN2), transcript variant 1, mRNA [NM_001040214] |
| TIGD7 | NM_033208 | tigger transposable element derived 7 (TIGD7), mRNA [NM_033208] |
| HS1BP3 | BC038847 | gb|Homo sapiens HCLS1 binding protein 3, mRNA (cDNA clone IMAGE:6056200), with apparent retained intron. [BC038847] |
| CACNA1B | ENST00000371367 | ens|calcium channel, voltage-dependent, N type, alpha 1B subunit [Source:HGNC Symbol;Acc:1389] [ENST00000371367] |
| XLOC_003690 | ENST00000507933 | tc|Q49RB8_9ROSI (Q49RB8) Auxin-binding protein 1, partial (7%) [THC2663615] |
| DBNDD1 | NM_001042610 | dysbindin (dystrobrevin binding protein 1) domain containing 1 (DBNDD1), transcript variant 1, mRNA [NM_001042610] |
| XLOC_l2_002767 | TCONS_l2_00005184 | linc|BROAD Institute lincRNA (XLOC_l2_002767), lincRNA [TCONS_l2_00005184] |
| XLOC_003857 | TCONS_00008412 | linc|BROAD Institute lincRNA (XLOC_003857), lincRNA [TCONS_00008412] |
| DEFB133 | NM_001166478 | defensin, beta 133 (DEFB133), mRNA [NM_001166478] |
| SNAR-H | NR_024342 | small ILF3/NF90-associated RNA H (SNAR-H), small nuclear RNA [NR_024342] |
| XLOC_005426 | TCONS_00012569 | linc|BROAD Institute lincRNA (XLOC_005426), lincRNA [TCONS_00012569] |
| FAM196B | NM_001129891 | family with sequence similarity 196, member B (FAM196B), mRNA [NM_001129891] |
| XLOC_011936 | TCONS_00024643 | linc|BROAD Institute lincRNA (XLOC_011936), lincRNA [TCONS_00024643] |
| BNIP2 | NM_004330 | BCL2/adenovirus E1B 19kDa interacting protein 2 (BNIP2), mRNA [NM_004330] |
| ENST00000503007 | ENST00000503007 | gb|AGENCOURT_6625132 NIH_MGC_114 Homo sapiens cDNA clone IMAGE:5763107 5', mRNA sequence [BM925358] |
| XLOC_l2_003911 | ENST00000413501 | linc|BROAD Institute lincRNA (XLOC_l2_003911), lincRNA [TCONS_l2_00007088] |
| LOC149134 | NR_015422 | uncharacterized LOC149134 (LOC149134), non-coding RNA [NR_015422] |
| CPEB4 | NM_030627 | cytoplasmic polyadenylation element binding protein 4 (CPEB4), mRNA [NM_030627] |
| LOC400568 | BC043554 | gb|Homo sapiens cDNA clone IMAGE:5176687. [BC043554] |
| XLOC_002938 | TCONS_00006322 | linc|BROAD Institute lincRNA (XLOC_002938), lincRNA [TCONS_00006322] |
| GSX2 | NM_133267 | GS homeobox 2 (GSX2), mRNA [NM_133267] |
| ENST00000525043 | ENST00000525043 | ens|chromosome 8 open reading frame 49 [Source:HGNC Symbol;Acc:32200] [ENST00000525043] |
| ZNF569 | NM_152484 | zinc finger protein 569 (ZNF569), mRNA [NM_152484] |
| PRODH | NM_016335 | proline dehydrogenase (oxidase) 1 (PRODH), nuclear gene encoding mitochondrial protein, transcript variant 1, mRNA [NM_016335] |
| CIB3 | NM_054113 | calcium and integrin binding family member 3 (CIB3), mRNA [NM_054113] |
| LOC400043 | NR_026656 | uncharacterized LOC400043 (LOC400043), non-coding RNA [NR_026656] |
| Q33DX6 | ENST00000431385 | tc|Q33DX6_9HYME (Q33DX6) Ecdysone receptor B isoform (Fragment), partial (16%) [THC2691143] |
| TSPY2 | NM_022573 | testis specific protein, Y-linked 2 (TSPY2), mRNA [NM_022573] |
| LOC100507160 | XR_110053 | ref|PREDICTED: Homo sapiens hypothetical LOC100507160 (LOC100507160), miscRNA [XR_110053] |
| XLOC_008601 | ENST00000450054 | linc|BROAD Institute lincRNA (XLOC_008601), lincRNA [TCONS_00017862] |
| XLOC_001849 | ENST00000428487 | tc|Q2I700_9GAST (Q2I700) NADH dehydrogenase subunit 1, partial (7%) [THC2764903] |
| AY358232 | AY358232 | gb|Homo sapiens clone DNA139644 QRWT5810 (UNQ5810) mRNA, complete cds. [AY358232] |
| XLOC_004773 | ENST00000507251 | tc|Q2SEC0_HAHCH (Q2SEC0) Uncharacterized protein conserved in bacteria, partial (7%) [THC2668578] |
| BTBD9 | NM_052893 | BTB (POZ) domain containing 9 (BTBD9), transcript variant 1, mRNA [NM_052893] |
| AP1S3 | ENST00000409375 | ens|adaptor-related protein complex 1, sigma 3 subunit [Source:HGNC Symbol;Acc:18971] [ENST00000409375] |
| LOC100506213 | XR_110839 | ref|PREDICTED: Homo sapiens hypothetical LOC100506213 (LOC100506213), miscRNA [XR_110839] |
| FLJ42709 | NR_021490 | uncharacterized LOC441094 (FLJ42709), non-coding RNA [NR_021490] |
| WARS2 | NM_201263 | tryptophanyl tRNA synthetase 2, mitochondrial (WARS2), nuclear gene encoding mitochondrial protein, transcript variant 2, mRNA [NM_201263] |
| ENST00000432377 | ENST00000432377 | gb|Homo sapiens, clone IMAGE:4779414, mRNA. [BC038366] |
| XLOC_004260 | TCONS_00010608 | linc|BROAD Institute lincRNA (XLOC_004260), lincRNA [TCONS_00010608] |
| LOC283481 | NR_036487 | uncharacterized LOC283481 (LOC283481), non-coding RNA [NR_036487] |
| HEPHL1 | NM_001098672 | hephaestin-like 1 (HEPHL1), mRNA [NM_001098672] |
| XLOC_006984 | TCONS_00014918 | linc|BROAD Institute lincRNA (XLOC_006984), lincRNA [TCONS_00014918] |
| IFITM5 | NM_001025295 | interferon induced transmembrane protein 5 (IFITM5), mRNA [NM_001025295] |
| CRTAM | NM_019604 | cytotoxic and regulatory T cell molecule (CRTAM), mRNA [NM_019604] |
| BC040680 | BC040680 | gb|Homo sapiens cDNA clone IMAGE:4817893. [BC040680] |
| XLOC_013737 | ENST00000422519 | tc|ALU7_HUMAN (P39194) Alu subfamily SQ sequence contamination warning entry, partial (15%) [THC2620692] |
| ZFP1 | NM_153688 | zinc finger protein 1 homolog (mouse) (ZFP1), mRNA [NM_153688] |
| LOC100507092 | XR_109285 | ref|PREDICTED: Homo sapiens hypothetical LOC100507092 (LOC100507092), miscRNA [XR_109285] |
| C8orf38 | NM_152416 | chromosome 8 open reading frame 38 (C8orf38), nuclear gene encoding mitochondrial protein, mRNA [NM_152416] |
| DMBX1 | NM_147192 | diencephalon/mesencephalon homeobox 1 (DMBX1), transcript variant 2, mRNA [NM_147192] |
| XLOC_003833 | TCONS_00008379 | linc|BROAD Institute lincRNA (XLOC_003833), lincRNA [TCONS_00008379] |
| XLOC_l2_011027 | ENST00000514073 | gb|AGENCOURT_6566087 NIH_MGC_67 Homo sapiens cDNA clone IMAGE:5550926 5', mRNA sequence [BM561118] |
| SNAR-B2 | NR_024230 | small ILF3/NF90-associated RNA B2 (SNAR-B2), small nuclear RNA [NR_024230] |
| LINC00256B | NR_024376 | long intergenic non-protein coding RNA 256B (LINC00256B), non-coding RNA [NR_024376] |
| CRYAA | NM_000394 | crystallin, alpha A (CRYAA), mRNA [NM_000394] |
| XLOC_011181 | AK058147 | tc|GB|AK058147.1|AK058147.1 Homo sapiens cDNA FLJ25418 fis, clone TST03512 [NP1154364] |
| LRRC8E | NM_025061 | leucine rich repeat containing 8 family, member E (LRRC8E), mRNA [NM_025061] |
| KRT33B | NM_002279 | keratin 33B (KRT33B), mRNA [NM_002279] |
| LOC644662 | XM_001714709 | ref|PREDICTED: Homo sapiens hypothetical protein LOC644662 (LOC644662), mRNA [XM_001714709] |
| ZNF555 | NM_152791 | zinc finger protein 555 (ZNF555), transcript variant 1, mRNA [NM_152791] |
| FBXO10 | NM_012166 | F-box protein 10 (FBXO10), mRNA [NM_012166] |
| LOC339260 | BC041488 | gb|Homo sapiens cDNA clone IMAGE:5494257, partial cds. [BC041488] |
| FLJ31356 | XR_109966 | ref|PREDICTED: Homo sapiens hypothetical protein FLJ31356 (FLJ31356), miscRNA [XR_109966] |
| COQ7 | NM_016138 | coenzyme Q7 homolog, ubiquinone (yeast) (COQ7), transcript variant 1, mRNA [NM_016138] |
| XLOC_008384 | ENST00000413286 | gb|zp30g05.r1 Stratagene neuroepithelium (#937231) Homo sapiens cDNA clone IMAGE:611000 5' similar to contains Alu repetitive element;contains element L1 repetitive element ;, mRNA sequence [AA176359] |
| PAWR | NM_002583 | PRKC, apoptosis, WT1, regulator (PAWR), mRNA [NM_002583] |
| LRRC31 | NM_024727 | leucine rich repeat containing 31 (LRRC31), mRNA [NM_024727] |
| XLOC_010460 | TCONS_00021865 | linc|BROAD Institute lincRNA (XLOC_010460), lincRNA [TCONS_00021865] |
| ABHD13 | NM_032859 | abhydrolase domain containing 13 (ABHD13), mRNA [NM_032859] |
| XLOC_010842 | TCONS_00022518 | linc|BROAD Institute lincRNA (XLOC_010842), lincRNA [TCONS_00022518] |
| XLOC_002965 | ENST00000417384 | gb|DB238603 TRACH3 Homo sapiens cDNA clone TRACH3034584 5', mRNA sequence [DB238603] |
| XLOC_006756 | ENST00000517491 | Unknown |
| XLOC_l2_013582 | ENST00000424515 | tc|Q8VW03_CORGL (Q8VW03) CytC-related protein, partial (5%) [THC2613552] |
| XLOC_003457 | THC2780555 | tc|Q952V6_9SMEG (Q952V6) Cytochrome b (Fragment), partial (5%) [THC2780555] |
| NXF5 | NM_032946 | nuclear RNA export factor 5 (NXF5), transcript variant 1, mRNA [NM_032946] |
| ST6GALNAC1 | NM_018414 | ST6 (alpha-N-acetyl-neuraminyl-2,3-beta-galactosyl-1,3)-N-acetylgalactosaminide alpha-2,6-sialyltransferase 1 (ST6GALNAC1), mRNA [NM_018414] |
| KRT38 | NM_006771 | keratin 38 (KRT38), mRNA [NM_006771] |
| HJURP | NM_018410 | Holliday junction recognition protein (HJURP), mRNA [NM_018410] |
| PNLIPRP3 | NM_001011709 | pancreatic lipase-related protein 3 (PNLIPRP3), mRNA [NM_001011709] |
| KCND3 | NM_004980 | potassium voltage-gated channel, Shal-related subfamily, member 3 (KCND3), transcript variant 1, mRNA [NM_004980] |
| XLOC_008950 | ENST00000421597 | linc|BROAD Institute lincRNA (XLOC_008950), lincRNA [TCONS_00018049] |
| LCMT2 | NM_014793 | leucine carboxyl methyltransferase 2 (LCMT2), mRNA [NM_014793] |
| XLOC_011090 | ENST00000554142 | linc|BROAD Institute lincRNA (XLOC_011090), lincRNA [TCONS_00022819] |
| LOC100652778 | XR_132647 | ref|PREDICTED: Homo sapiens hypothetical LOC100652778 (LOC100652778), miscRNA [XR_132647] |
| H1FX-AS1 | NR_026991 | H1FX antisense RNA 1 (non-protein coding) (H1FX-AS1), non-coding RNA [NR_026991] |
| SPIN1 | NM_006717 | spindlin 1 (SPIN1), mRNA [NM_006717] |
| CYP4Z1 | NM_178134 | cytochrome P450, family 4, subfamily Z, polypeptide 1 (CYP4Z1), mRNA [NM_178134] |
| TMEM22 | NM_025246 | transmembrane protein 22 (TMEM22), transcript variant 1, mRNA [NM_025246] |
| OR2H2 | NM_007160 | olfactory receptor, family 2, subfamily H, member 2 (OR2H2), mRNA [NM_007160] |
| RAD51 | NM_002875 | RAD51 homolog (S. cerevisiae) (RAD51), transcript variant 1, mRNA [NM_002875] |
| LOC389641 | NR_033928 | uncharacterized LOC389641 (LOC389641), non-coding RNA [NR_033928] |
| KIT | NM_000222 | v-kit Hardy-Zuckerman 4 feline sarcoma viral oncogene homolog (KIT), transcript variant 1, mRNA [NM_000222] |
| LOC100128019 | AK125579 | gb|Homo sapiens cDNA FLJ43591 fis, clone SMINT2002743. [AK125579] |
| A_33_P3281303 | A_33_P3281303 | Unknown |
| LAMA4 | NM_001105206 | laminin, alpha 4 (LAMA4), transcript variant 1, mRNA [NM_001105206] |
| KY | NM_178554 | kyphoscoliosis peptidase (KY), mRNA [NM_178554] |
| BCLAF1 | NM_014739 | BCL2-associated transcription factor 1 (BCLAF1), transcript variant 1, mRNA [NM_014739] |
| XLOC_008530 | ENST00000518000 | tc|O52174_SALBE (O52174) OrfVII, partial (19%) [THC2500857] |
| GSC | NM_173849 | goosecoid homeobox (GSC), mRNA [NM_173849] |
| ESRP2 | NM_024939 | epithelial splicing regulatory protein 2 (ESRP2), mRNA [NM_024939] |
| GYS2 | NM_021957 | glycogen synthase 2 (liver) (GYS2), mRNA [NM_021957] |
| BAG5 | NM_001015049 | BCL2-associated athanogene 5 (BAG5), transcript variant 1, mRNA [NM_001015049] |
| XLOC_l2_006594 | TCONS_l2_00012302 | linc|BROAD Institute lincRNA (XLOC_l2_006594), lincRNA [TCONS_l2_00012302] |
| LOC100506924 | ENST00000465970 | ref|PREDICTED: Homo sapiens hypothetical LOC100506924 (LOC100506924), miscRNA [XR_110015] |
| XLOC_l2_002477 | ENST00000444123 | gb|BX093419 Soares_testis_NHT Homo sapiens cDNA clone IMAGp998K141785 ; IMAGE:727813, mRNA sequence [BX093419] |
| EPHA6 | NM_173655 | EPH receptor A6 (EPHA6), transcript variant 2, mRNA [NM_173655] |
| TBC1D22A | NM_014346 | TBC1 domain family, member 22A (TBC1D22A), mRNA [NM_014346] |
| ENST00000399342 | ENST00000399342 | ens|Uncharacterized protein [Source:UniProtKB/TrEMBL;Acc:A8MV31] [ENST00000399342] |
| XLOC_l2_008434 | ENST00000423530 | linc|BROAD Institute lincRNA (XLOC_l2_008434), lincRNA [TCONS_l2_00016080] |
| SNORA77 | DB361496 | gb|DB361496 MAMMA1 Homo sapiens cDNA clone MAMMA1001810 3', mRNA sequence [DB361496] |
| ZCCHC16 | NM_001004308 | zinc finger, CCHC domain containing 16 (ZCCHC16), mRNA [NM_001004308] |
| A_33_P3218013 | A_33_P3218013 | Unknown |
| PTPLAD1 | NM_016395 | protein tyrosine phosphatase-like A domain containing 1 (PTPLAD1), mRNA [NM_016395] |
| ZNF200 | NM_003454 | zinc finger protein 200 (ZNF200), transcript variant 1, mRNA [NM_003454] |
| MS4A7 | NM_021201 | membrane-spanning 4-domains, subfamily A, member 7 (MS4A7), transcript variant 1, mRNA [NM_021201] |
| XLOC_l2_011983 | ENST00000505339 | linc|BROAD Institute lincRNA (XLOC_l2_011983), lincRNA [TCONS_l2_00023706] |
| BMP2 | NM_001200 | bone morphogenetic protein 2 (BMP2), mRNA [NM_001200] |
| XLOC_008654 | ENST00000451295 | tc|Q8TVR9_METKA (Q8TVR9) Predicted component of a thermophile-specific DNA repair system, contains a RAMP domain, partial (5%) [THC2730625] |
| FZD1 | NM_003505 | frizzled family receptor 1 (FZD1), mRNA [NM_003505] |
| TRIM49L2 | ENST00000432771 | ens|tripartite motif containing 49-like 2 [Source:HGNC Symbol;Acc:38877] [ENST00000432771] |
| PYDC2 | NM_001083308 | pyrin domain containing 2 (PYDC2), mRNA [NM_001083308] |
| THC2662725 | THC2662725 | tc|AF396935 seven-span membrane protein FIRE {Mus musculus} (exp=-1; wgp=0; cg=0), partial (5%) [THC2662725] |
| FAM83E | NM_017708 | family with sequence similarity 83, member E (FAM83E), mRNA [NM_017708] |
| ASB7 | NM_024708 | ankyrin repeat and SOCS box containing 7 (ASB7), transcript variant 1, mRNA [NM_024708] |
| UGT3A1 | NM_152404 | UDP glycosyltransferase 3 family, polypeptide A1 (UGT3A1), transcript variant 1, mRNA [NM_152404] |
| SAA4 | NM_006512 | serum amyloid A4, constitutive (SAA4), mRNA [NM_006512] |
| NANP | NM_152667 | N-acetylneuraminic acid phosphatase (NANP), mRNA [NM_152667] |
| XLOC_002588 | BC034913 | tc|Q5VSG2_HUMAN (Q5VSG2) OTTHUMP00000022690, partial (5%) [THC2607785] |
| XLOC_004377 | TCONS_00009957 | linc|BROAD Institute lincRNA (XLOC_004377), lincRNA [TCONS_00009957] |
| BTG3 | NM_006806 | BTG family, member 3 (BTG3), transcript variant 2, mRNA [NM_006806] |
| A_33_P3339269 | A_33_P3339269 | Unknown |
| XLOC_006734 | ENST00000523852 | gb|603075756F1 NIH_MGC_119 Homo sapiens cDNA clone IMAGE:5167741 5', mRNA sequence [BI827086] |
| XLOC_l2_008088 | BM708281 | gb|UI-E-CI1-afu-k-11-0-UI.r1 UI-E-CI1 Homo sapiens cDNA clone UI-E-CI1-afu-k-11-0-UI 5', mRNA sequence [BM708281] |
| ZPBP | ENST00000465922 | ens|zona pellucida binding protein [Source:HGNC Symbol;Acc:15662] [ENST00000465922] |
| PPIP5K1 | NM_001190214 | diphosphoinositol pentakisphosphate kinase 1 (PPIP5K1), transcript variant 7, mRNA [NM_001190214] |
| FAM172A | NM_032042 | family with sequence similarity 172, member A (FAM172A), transcript variant 1, mRNA [NM_032042] |
| BC014063 | BC014063 | gb|Homo sapiens hypothetical protein LOC151878, mRNA (cDNA clone IMAGE:3844647), partial cds. [BC014063] |
| NR4A3 | NM_173199 | nuclear receptor subfamily 4, group A, member 3 (NR4A3), transcript variant 4, mRNA [NM_173199] |
| XLOC_010776 | ENST00000552826 | gb|603078292F1 NIH_MGC_119 Homo sapiens cDNA clone IMAGE:5169862 5', mRNA sequence [BI828513] |
| TOMM40 | NM_006114 | translocase of outer mitochondrial membrane 40 homolog (yeast) (TOMM40), nuclear gene encoding mitochondrial protein, transcript variant 3, mRNA [NM_006114] |
| ENST00000415246 | ENST00000415246 | Unknown |
| BMPR2 | NM_001204 | bone morphogenetic protein receptor, type II (serine/threonine kinase) (BMPR2), mRNA [NM_001204] |
| C10orf67 | NM_153714 | chromosome 10 open reading frame 67 (C10orf67), mRNA [NM_153714] |
| XLOC_l2_010491 | AK091265 | linc|BROAD Institute lincRNA (XLOC_l2_010491), lincRNA [TCONS_l2_00020301] |
| TRIML2 | NM_173553 | tripartite motif family-like 2 (TRIML2), mRNA [NM_173553] |
| ADHFE1 | NM_144650 | alcohol dehydrogenase, iron containing, 1 (ADHFE1), nuclear gene encoding mitochondrial protein, mRNA [NM_144650] |
| XLOC_001410 | TCONS_00002862 | linc|BROAD Institute lincRNA (XLOC_001410), lincRNA [TCONS_00002862] |
| LOC100506907 | ENST00000506660 | ref|PREDICTED: Homo sapiens hypothetical LOC100506907 (LOC100506907), miscRNA [XR_108478] |
| FIGN | ENST00000409634 | ens|fidgetin [Source:HGNC Symbol;Acc:13285] [ENST00000409634] |
| XLOC_011880 | TCONS_00024587 | linc|BROAD Institute lincRNA (XLOC_011880), lincRNA [TCONS_00024587] |
| GLI3 | NM_000168 | GLI family zinc finger 3 (GLI3), mRNA [NM_000168] |
| CYTL1 | NM_018659 | cytokine-like 1 (CYTL1), mRNA [NM_018659] |
| XLOC_008343 | ENST00000446337 | gb|UI-H-BI1-aez-f-02-0-UI.s1 NCI_CGAP_Sub3 Homo sapiens cDNA clone IMAGE:2721147 3', mRNA sequence [AW206525] |
| POLR2D | NM_004805 | polymerase (RNA) II (DNA directed) polypeptide D (POLR2D), mRNA [NM_004805] |
| CES4A | NM_173815 | carboxylesterase 4A (CES4A), transcript variant 1, mRNA [NM_173815] |
| PLAT | NM_000930 | plasminogen activator, tissue (PLAT), transcript variant 1, mRNA [NM_000930] |
| XLOC_003721 | TCONS_00008279 | linc|BROAD Institute lincRNA (XLOC_003721), lincRNA [TCONS_00008279] |
| SEMA6C | NM_030913 | sema domain, transmembrane domain (TM), and cytoplasmic domain, (semaphorin) 6C (SEMA6C), transcript variant 2, mRNA [NM_030913] |
| HECW1 | NM_015052 | HECT, C2 and WW domain containing E3 ubiquitin protein ligase 1 (HECW1), mRNA [NM_015052] |
| XLOC_l2_010118 | AK027155 | linc|BROAD Institute lincRNA (XLOC_l2_010118), lincRNA [TCONS_l2_00019181] |
| CLDN25 | NM_001101389 | claudin 25 (CLDN25), mRNA [NM_001101389] |
| DKK3 | NM_015881 | dickkopf homolog 3 (Xenopus laevis) (DKK3), transcript variant 1, mRNA [NM_015881] |
| XLOC_001099 | ENST00000426575 | linc|BROAD Institute lincRNA (XLOC_001099), lincRNA [TCONS_00000691] |
| RIPK4 | NM_020639 | receptor-interacting serine-threonine kinase 4 (RIPK4), mRNA [NM_020639] |
| SHISA6 | NM_207386 | shisa homolog 6 (Xenopus laevis) (SHISA6), transcript variant 1, mRNA [NM_207386] |
| MKLN1 | NM_013255 | muskelin 1, intracellular mediator containing kelch motifs (MKLN1), transcript variant 2, mRNA [NM_013255] |
| XLOC_007710 | CR627148 | linc|BROAD Institute lincRNA (XLOC_007710), lincRNA [TCONS_00015825] |
| XLOC_003682 | TCONS_00008230 | linc|BROAD Institute lincRNA (XLOC_003682), lincRNA [TCONS_00008230] |
| WHSC2 | NM_005663 | Wolf-Hirschhorn syndrome candidate 2 (WHSC2), mRNA [NM_005663] |
| RGS4 | NM_005613 | regulator of G-protein signaling 4 (RGS4), transcript variant 2, mRNA [NM_005613] |
| C22orf23 | NM_032561 | chromosome 22 open reading frame 23 (C22orf23), transcript variant 1, mRNA [NM_032561] |
| RGS20 | NM_170587 | regulator of G-protein signaling 20 (RGS20), transcript variant 1, mRNA [NM_170587] |
| CYTH2 | NM_004228 | cytohesin 2 (CYTH2), transcript variant 2, mRNA [NM_004228] |
| LOC100505576 | NR_038847 | uncharacterized LOC100505576 (LOC100505576), non-coding RNA [NR_038847] |
| LCA5 | NM_181714 | Leber congenital amaurosis 5 (LCA5), transcript variant 1, mRNA [NM_181714] |
| XLOC_006283 | TCONS_00013619 | linc|BROAD Institute lincRNA (XLOC_006283), lincRNA [TCONS_00013619] |
| SMARCAD1 | NM_020159 | SWI/SNF-related, matrix-associated actin-dependent regulator of chromatin, subfamily a, containing DEAD/H box 1 (SMARCAD1), transcript variant 3, mRNA [NM_020159] |
| TECRL | NM_001010874 | trans-2,3-enoyl-CoA reductase-like (TECRL), mRNA [NM_001010874] |
| XLOC_012396 | ENST00000431343 | linc|BROAD Institute lincRNA (XLOC_012396), lincRNA [TCONS_00025211] |
| SETD6 | NM_024860 | SET domain containing 6 (SETD6), transcript variant 2, mRNA [NM_024860] |
| KCNS3 | NM_002252 | potassium voltage-gated channel, delayed-rectifier, subfamily S, member 3 (KCNS3), mRNA [NM_002252] |
| IRS1 | NM_005544 | insulin receptor substrate 1 (IRS1), mRNA [NM_005544] |
| SLC26A1 | NM_134425 | solute carrier family 26 (sulfate transporter), member 1 (SLC26A1), transcript variant 2, mRNA [NM_134425] |
| NSMAF | NM_003580 | neutral sphingomyelinase (N-SMase) activation associated factor (NSMAF), transcript variant 1, mRNA [NM_003580] |
| THC2780703 | THC2780703 | tc|Q9P1R7_HUMAN (Q9P1R7) HDCMB45P (Fragment), partial (19%) [THC2780703] |
| LOC100506859 | XM_003118889 | ref|PREDICTED: Homo sapiens serine/threonine-protein kinase Nek5-like (LOC100506859), mRNA [XM_003118889] |
| KCNC1 | NM_004976 | potassium voltage-gated channel, Shaw-related subfamily, member 1 (KCNC1), transcript variant B, mRNA [NM_004976] |
| PLSCR2 | ENST00000463633 | ens|phospholipid scramblase 2 [Source:HGNC Symbol;Acc:16494] [ENST00000463633] |
| SPACA5 | NM_205856 | sperm acrosome associated 5 (SPACA5), mRNA [NM_205856] |
| SEC62 | NM_003262 | SEC62 homolog (S. cerevisiae) (SEC62), mRNA [NM_003262] |
| LOC388456 | BC039671 | gb|Homo sapiens hypothetical gene supported by BC039671, mRNA (cDNA clone IMAGE:5167959). [BC039671] |
| LOC285401 | NR_027104 | uncharacterized LOC285401 (LOC285401), non-coding RNA [NR_027104] |
| XLOC_013615 | ENST00000449500 | linc|BROAD Institute lincRNA (XLOC_013615), lincRNA [TCONS_00027984] |
| ENST00000433310 | ENST00000433310 | gb|UI-H-FH1-bfk-h-04-0-UI.s1 NCI_CGAP_FH1 Homo sapiens cDNA clone UI-H-FH1-bfk-h-04-0-UI 3', mRNA sequence [BU618747] |
| LOC729866 | AK057458 | gb|Homo sapiens cDNA FLJ32896 fis, clone TESTI2005155. [AK057458] |
| IHH | NM_002181 | Indian hedgehog (IHH), mRNA [NM_002181] |
| COL6A4P2 | NR_027898 | collagen, type VI, alpha 4 pseudogene 2 (COL6A4P2), non-coding RNA [NR_027898] |
| EXOC3L2 | NM_138568 | exocyst complex component 3-like 2 (EXOC3L2), mRNA [NM_138568] |
| TBX20 | NM_001166220 | T-box 20 (TBX20), transcript variant 2, mRNA [NM_001166220] |
| XLOC_l2_004594 | TCONS_l2_00008471 | linc|BROAD Institute lincRNA (XLOC_l2_004594), lincRNA [TCONS_l2_00008471] |
| A_33_P3245126 | A_33_P3245126 | Unknown |
| XLOC_l2_009131 | AK311573 | gb|DA297795 BRHIP2 Homo sapiens cDNA clone BRHIP2011312 5', mRNA sequence [DA297795] |
| TRIM26 | NM_003449 | tripartite motif containing 26 (TRIM26), transcript variant 1, mRNA [NM_003449] |
| CLEC5A | NM_013252 | C-type lectin domain family 5, member A (CLEC5A), mRNA [NM_013252] |
| XLOC_008439 | ENST00000416310 | gb|BX114033 NCI_CGAP_GCB1 Homo sapiens cDNA clone IMAGp998C012036, mRNA sequence [BX114033] |
| LOC285768 | NR_027115 | uncharacterized LOC285768 (LOC285768), non-coding RNA [NR_027115] |
| XLOC_000983 | TCONS_00001644 | linc|BROAD Institute lincRNA (XLOC_000983), lincRNA [TCONS_00001644] |
| LOC100652883 | XM_003403469 | ref|PREDICTED: Homo sapiens arachidonate 15-lipoxygenase-like (LOC100652883), mRNA [XM_003403469] |
| XLOC_004299 | TCONS_00009877 | linc|BROAD Institute lincRNA (XLOC_004299), lincRNA [TCONS_00009877] |
| TACR3 | NM_001059 | tachykinin receptor 3 (TACR3), mRNA [NM_001059] |
| GALC | ENST00000445021 | ens|galactosylceramidase [Source:HGNC Symbol;Acc:4115] [ENST00000445021] |
| IFNA6 | NM_021002 | interferon, alpha 6 (IFNA6), mRNA [NM_021002] |
| SLC40A1 | NM_014585 | solute carrier family 40 (iron-regulated transporter), member 1 (SLC40A1), mRNA [NM_014585] |
| ARHGAP32 | NM_014715 | Rho GTPase activating protein 32 (ARHGAP32), transcript variant 2, mRNA [NM_014715] |
| XBP1 | NM_005080 | X-box binding protein 1 (XBP1), transcript variant 1, mRNA [NM_005080] |
| SETD5 | NM_001080517 | SET domain containing 5 (SETD5), mRNA [NM_001080517] |
| SPRED2 | NM_181784 | sprouty-related, EVH1 domain containing 2 (SPRED2), transcript variant 1, mRNA [NM_181784] |
| LOC100505678 | NR_040093 | uncharacterized LOC100505678 (LOC100505678), non-coding RNA [NR_040093] |
| POLR1C | NM_203290 | polymerase (RNA) I polypeptide C, 30kDa (POLR1C), mRNA [NM_203290] |
| XLOC_006863 | ENST00000522365 | tc|ALU7_HUMAN (P39194) Alu subfamily SQ sequence contamination warning entry, partial (8%) [THC2497639] |
| FRMD3 | NM_174938 | FERM domain containing 3 (FRMD3), transcript variant 1, mRNA [NM_174938] |
| LRRTM3 | NM_178011 | leucine rich repeat transmembrane neuronal 3 (LRRTM3), mRNA [NM_178011] |
| AMELX | NM_182681 | amelogenin, X-linked (AMELX), transcript variant 2, mRNA [NM_182681] |
| BMP3 | NM_001201 | bone morphogenetic protein 3 (BMP3), mRNA [NM_001201] |
| XLOC_001704 | TCONS_00003901 | linc|BROAD Institute lincRNA (XLOC_001704), lincRNA [TCONS_00003901] |
| TMEFF2 | NM_016192 | transmembrane protein with EGF-like and two follistatin-like domains 2 (TMEFF2), mRNA [NM_016192] |
| MYO16 | NM_001198950 | myosin XVI (MYO16), transcript variant 1, mRNA [NM_001198950] |
| THC2489055 | THC2489055 | tc|Q3MF38_ANAVT (Q3MF38) Extracellular solute-binding protein, family 3 precursor, partial (7%) [THC2489055] |
| P2RY6 | NM_176798 | pyrimidinergic receptor P2Y, G-protein coupled, 6 (P2RY6), transcript variant 2, mRNA [NM_176798] |
| A_33_P3238402 | A_33_P3238402 | Unknown |
| STIM2 | NM_020860 | stromal interaction molecule 2 (STIM2), transcript variant 2, mRNA [NM_020860] |
| PHF21A | NM_001101802 | PHD finger protein 21A (PHF21A), transcript variant 1, mRNA [NM_001101802] |
| KMO | NM_003679 | kynurenine 3-monooxygenase (kynurenine 3-hydroxylase) (KMO), mRNA [NM_003679] |
| NFATC3 | NM_173165 | nuclear factor of activated T-cells, cytoplasmic, calcineurin-dependent 3 (NFATC3), transcript variant 1, mRNA [NM_173165] |
| KRT35 | NM_002280 | keratin 35 (KRT35), mRNA [NM_002280] |
| SSX7 | NM_173358 | synovial sarcoma, X breakpoint 7 (SSX7), mRNA [NM_173358] |
| YY1AP1 | ENST00000405763 | ens|YY1 associated protein 1 [Source:HGNC Symbol;Acc:30935] [ENST00000405763] |
| NBR1 | ENST00000389311 | ens|neighbor of BRCA1 gene 1 [Source:HGNC Symbol;Acc:6746] [ENST00000389311] |
| ENST00000440711 | ENST00000440711 | tc|Q90X21_CHICK (Q90X21) Olig2 (Fragment), partial (17%) [THC2716970] |
| PITX2 | NM_153426 | paired-like homeodomain 2 (PITX2), transcript variant 2, mRNA [NM_153426] |
| XLOC_014105 | TCONS_00029166 | linc|BROAD Institute lincRNA (XLOC_014105), lincRNA [TCONS_00029166] |
| BQ888653 | BQ888653 | gb|AGENCOURT_8137257 Lupski_dorsal_root_ganglion Homo sapiens cDNA clone IMAGE:6184026 5', mRNA sequence [BQ888653] |
| XLOC_l2_001597 | AK022382 | linc|BROAD Institute lincRNA (XLOC_l2_001597), lincRNA [TCONS_l2_00003983] |
| FARP2 | NM_014808 | FERM, RhoGEF and pleckstrin domain protein 2 (FARP2), mRNA [NM_014808] |
| HSPB7 | NM_014424 | heat shock 27kDa protein family, member 7 (cardiovascular) (HSPB7), mRNA [NM_014424] |
| XLOC_000070 | ENST00000430540 | gb|BX113695 Soares_testis_NHT Homo sapiens cDNA clone IMAGp998L123517, mRNA sequence [BX113695] |
| LOC100499177 | NR_034075 | uncharacterized LOC100499177 (LOC100499177), transcript variant 1, non-coding RNA [NR_034075] |
| C6orf154 | NM_001012974 | chromosome 6 open reading frame 154 (C6orf154), mRNA [NM_001012974] |
| XLOC_003063 | TCONS_00007128 | linc|BROAD Institute lincRNA (XLOC_003063), lincRNA [TCONS_00007128] |
| CHST10 | NM_004854 | carbohydrate sulfotransferase 10 (CHST10), mRNA [NM_004854] |
| XLOC_004740 | ENST00000511443 | linc|BROAD Institute lincRNA (XLOC_004740), lincRNA [TCONS_00009609] |
| YIF1B | NM_033557 | Yip1 interacting factor homolog B (S. cerevisiae) (YIF1B), transcript variant 2, mRNA [NM_033557] |
| NUP54 | NM_017426 | nucleoporin 54kDa (NUP54), mRNA [NM_017426] |
| DDX18 | NM_006773 | DEAD (Asp-Glu-Ala-Asp) box polypeptide 18 (DDX18), mRNA [NM_006773] |
| XLOC_013776 | ENST00000446280 | tc|FIZ1_HUMAN (Q96SL8) Flt3-interacting zinc finger protein 1, partial (5%) [THC2671691] |
| XLOC_006663 | TCONS_00014019 | linc|BROAD Institute lincRNA (XLOC_006663), lincRNA [TCONS_00014019] |
| C10orf68 | NM_024688 | chromosome 10 open reading frame 68 (C10orf68), mRNA [NM_024688] |
| CEP120 | NM_153223 | centrosomal protein 120kDa (CEP120), transcript variant 1, mRNA [NM_153223] |
| LOC100130673 | NR_038454 | phosphoribosyl pyrophosphate synthetase 2 pseudogene (LOC100130673), non-coding RNA [NR_038454] |
| OTOP2 | NM_178160 | otopetrin 2 (OTOP2), mRNA [NM_178160] |
| BM981407 | BM981407 | gb|UI-CF-EN1-adh-h-23-0-UI.s1 UI-CF-EN1 Homo sapiens cDNA clone UI-CF-EN1-adh-h-23-0-UI 3', mRNA sequence [BM981407] |
| LOC730755 | NM_001165252 | keratin associated protein 2-4-like (LOC730755), mRNA [NM_001165252] |
| CRYBA1 | NM_005208 | crystallin, beta A1 (CRYBA1), mRNA [NM_005208] |
| PSG1 | NM_006905 | pregnancy specific beta-1-glycoprotein 1 (PSG1), transcript variant 1, mRNA [NM_006905] |
| GHRLOS2 | NR_026829 | ghrelin opposite strand RNA 2 (non-protein coding) (GHRLOS2), non-coding RNA [NR_026829] |
| CR591103 | CR591103 | gb|full-length cDNA clone CS0DL001YG15 of B cells (Ramos cell line) Cot 25-normalized of Homo sapiens (human) [CR591103] |
| LRCH2 | NM_020871 | leucine-rich repeats and calponin homology (CH) domain containing 2 (LRCH2), transcript variant 1, mRNA [NM_020871] |
| LPA | NM_005577 | lipoprotein, Lp(a) (LPA), mRNA [NM_005577] |
| C3orf80 | NM_001168214 | chromosome 3 open reading frame 80 (C3orf80), mRNA [NM_001168214] |
| ATP12A | NM_001676 | ATPase, H+/K+ transporting, nongastric, alpha polypeptide (ATP12A), transcript variant 2, mRNA [NM_001676] |
| XLOC_l2_009139 | ENST00000418954 | linc|BROAD Institute lincRNA (XLOC_l2_009139), lincRNA [TCONS_l2_00017429] |
| PPP1R2P9 | NR_002191 | protein phosphatase 1, regulatory (inhibitor) subunit 2 pseudogene 9 (PPP1R2P9), non-coding RNA [NR_002191] |
| HSD11B1 | NM_181755 | hydroxysteroid (11-beta) dehydrogenase 1 (HSD11B1), transcript variant 2, mRNA [NM_181755] |
| LAMB3 | NM_001017402 | laminin, beta 3 (LAMB3), transcript variant 2, mRNA [NM_001017402] |
| WDR63 | NM_145172 | WD repeat domain 63 (WDR63), mRNA [NM_145172] |
| AMZ2P1 | NR_026903 | archaelysin family metallopeptidase 2 pseudogene 1 (AMZ2P1), non-coding RNA [NR_026903] |
| SVEP1 | ENST00000374461 | ens|sushi, von Willebrand factor type A, EGF and pentraxin domain containing 1 [Source:HGNC Symbol;Acc:15985] [ENST00000374461] |
| LOC100130924 | AK094424 | gb|Homo sapiens cDNA FLJ37105 fis, clone BRACE2019510. [AK094424] |
| MYO1F | NM_012335 | myosin IF (MYO1F), mRNA [NM_012335] |
| ICAM1 | NM_000201 | intercellular adhesion molecule 1 (ICAM1), mRNA [NM_000201] |
| ECE1 | NM_001397 | endothelin converting enzyme 1 (ECE1), transcript variant 1, mRNA [NM_001397] |
| ANKRD39 | NM_016466 | ankyrin repeat domain 39 (ANKRD39), mRNA [NM_016466] |
| PKD1L3 | NM_181536 | polycystic kidney disease 1-like 3 (PKD1L3), mRNA [NM_181536] |
| XLOC_003333 | ENST00000457125 | gb|AGENCOURT_10197749 NIH_MGC_126 Homo sapiens cDNA clone IMAGE:6559929 5', mRNA sequence [BU533525] |
| FOXD4 | NM_207305 | forkhead box D4 (FOXD4), mRNA [NM_207305] |
| RAP1GDS1 | NM_001100426 | RAP1, GTP-GDP dissociation stimulator 1 (RAP1GDS1), transcript variant 1, mRNA [NM_001100426] |
| ZFHX3 | NM_006885 | zinc finger homeobox 3 (ZFHX3), transcript variant A, mRNA [NM_006885] |
| XLOC_012288 | THC2673276 | linc|BROAD Institute lincRNA (XLOC_012288), lincRNA [TCONS_00025471] |
| A_33_P3323641 | A_33_P3323641 | Unknown |
| A2M | NM_000014 | alpha-2-macroglobulin (A2M), mRNA [NM_000014] |
| XLOC_011448 | TCONS_00023648 | linc|BROAD Institute lincRNA (XLOC_011448), lincRNA [TCONS_00023648] |
| XLOC_012115 | TCONS_00025312 | linc|BROAD Institute lincRNA (XLOC_012115), lincRNA [TCONS_00025312] |
| XLOC_008470 | ENST00000418426 | linc|BROAD Institute lincRNA (XLOC_008470), lincRNA [TCONS_00017820] |
| FLJ34223 | AK091542 | gb|Homo sapiens cDNA FLJ34223 fis, clone FCBBF3023061. [AK091542] |
| HS3ST6 | NM_001009606 | heparan sulfate (glucosamine) 3-O-sulfotransferase 6 (HS3ST6), mRNA [NM_001009606] |
| PLXNA4 | NM_020911 | plexin A4 (PLXNA4), transcript variant 1, mRNA [NM_020911] |
| CARKD | NM_001242881 | carbohydrate kinase domain containing (CARKD), transcript variant 2, mRNA [NM_001242881] |
| RYR1 | NM_000540 | ryanodine receptor 1 (skeletal) (RYR1), transcript variant 1, mRNA [NM_000540] |
| AMD1 | NM_001634 | adenosylmethionine decarboxylase 1 (AMD1), transcript variant 1, mRNA [NM_001634] |
| XLOC_l2_013383 | TCONS_l2_00025854 | linc|BROAD Institute lincRNA (XLOC_l2_013383), lincRNA [TCONS_l2_00025854] |
| XLOC_l2_014077 | ENST00000458645 | gb|DA731816 NT2RP1 Homo sapiens cDNA clone NT2RP1000862 5', mRNA sequence [DA731816] |
| LOC100505918 | NR_037851 | uncharacterized LOC100505918 (LOC100505918), non-coding RNA [NR_037851] |
| CPEB3 | NM_014912 | cytoplasmic polyadenylation element binding protein 3 (CPEB3), transcript variant 1, mRNA [NM_014912] |
| RPS26 | NM_001029 | ribosomal protein S26 (RPS26), mRNA [NM_001029] |
| C8orf48 | NM_001007090 | chromosome 8 open reading frame 48 (C8orf48), mRNA [NM_001007090] |
| X56665 | X56665 | gb|H.sapiens mRNA for rearranged T-cell receptor beta. [X56665] |
| AF351612 | AF351612 | gb|Homo sapiens UG0651E06 mRNA, complete cds. [AF351612] |
| PARP1 | ENST00000366790 | ens|poly (ADP-ribose) polymerase 1 [Source:HGNC Symbol;Acc:270] [ENST00000366790] |
| TSHZ2 | NM_173485 | teashirt zinc finger homeobox 2 (TSHZ2), transcript variant 1, mRNA [NM_173485] |
| TMEM165 | ENST00000515591 | ref|PREDICTED: Homo sapiens hypothetical LOC100506486 (LOC100506486), miscRNA [XR_132720] |
| SLC6A2 | NM_001172501 | solute carrier family 6 (neurotransmitter transporter, noradrenalin), member 2 (SLC6A2), transcript variant 2, mRNA [NM_001172501] |
| SNAR-D | NR_024243 | small ILF3/NF90-associated RNA D (SNAR-D), small nuclear RNA [NR_024243] |
| BEND7 | ENST00000466271 | tc|Q8N5T7_HUMAN (Q8N5T7) C10orf30 protein, partial (52%) [THC2654820] |
| BAZ2B | NM_013450 | bromodomain adjacent to zinc finger domain, 2B (BAZ2B), mRNA [NM_013450] |
| TRIM36 | NM_018700 | tripartite motif containing 36 (TRIM36), transcript variant 1, mRNA [NM_018700] |
| ODZ2 | NM_001122679 | odz, odd Oz/ten-m homolog 2 (Drosophila) (ODZ2), mRNA [NM_001122679] |
| PLCE1 | NM_016341 | phospholipase C, epsilon 1 (PLCE1), transcript variant 1, mRNA [NM_016341] |
| NOB1 | NM_014062 | NIN1/RPN12 binding protein 1 homolog (S. cerevisiae) (NOB1), mRNA [NM_014062] |
| XLOC_011753 | TCONS_00024861 | linc|BROAD Institute lincRNA (XLOC_011753), lincRNA [TCONS_00024861] |
| WDR37 | ENST00000381329 | ens|WD repeat domain 37 [Source:HGNC Symbol;Acc:31406] [ENST00000381329] |
| XLOC_004829 | TCONS_00010340 | linc|BROAD Institute lincRNA (XLOC_004829), lincRNA [TCONS_00010340] |
| IL17RB | NM_018725 | interleukin 17 receptor B (IL17RB), mRNA [NM_018725] |
| XLOC_l2_000033 | ENST00000424215 | tc|Q7CVR7_AGRT5 (Q7CVR7) AGR_L_941GMp, partial (6%) [THC2648044] |
| BU622976 | BU622976 | gb|UI-H-FL1-bgb-n-06-0-UI.s1 NCI_CGAP_FL1 Homo sapiens cDNA clone UI-H-FL1-bgb-n-06-0-UI 3', mRNA sequence [BU622976] |
| XLOC_008114 | TCONS_00017292 | linc|BROAD Institute lincRNA (XLOC_008114), lincRNA [TCONS_00017292] |
| GRXCR1 | NM_001080476 | glutaredoxin, cysteine rich 1 (GRXCR1), mRNA [NM_001080476] |
| FRMD5 | NM_032892 | FERM domain containing 5 (FRMD5), transcript variant 2, mRNA [NM_032892] |
| MAP2K1 | NM_002755 | mitogen-activated protein kinase kinase 1 (MAP2K1), mRNA [NM_002755] |
| LOC100505658 | NR_038333 | uncharacterized LOC100505658 (LOC100505658), non-coding RNA [NR_038333] |
| XLOC_l2_003626 | ENST00000453309 | gb|BX089760 Soares_NFL_T_GBC_S1 Homo sapiens cDNA clone IMAGp998N124130 ; IMAGE:1628363, mRNA sequence [BX089760] |
| PSG3 | NM_021016 | pregnancy specific beta-1-glycoprotein 3 (PSG3), mRNA [NM_021016] |
| ARHGAP35 | NM_004491 | Rho GTPase activating protein 35 (ARHGAP35), mRNA [NM_004491] |
| LOC100506758 | XR_110007 | ref|PREDICTED: Homo sapiens hypothetical LOC100506758 (LOC100506758), miscRNA [XR_110007] |
| XLOC_003825 | TCONS_00008369 | linc|BROAD Institute lincRNA (XLOC_003825), lincRNA [TCONS_00008369] |
| XLOC_005984 | ENST00000451755 | gb|zq43g09.r1 Stratagene hNT neuron (#937233) Homo sapiens cDNA clone IMAGE:632512 5', mRNA sequence [AA252637] |
| XLOC_008678 | TCONS_00018395 | linc|BROAD Institute lincRNA (XLOC_008678), lincRNA [TCONS_00018395] |
| FLJ32756 | AK057318 | gb|Homo sapiens cDNA FLJ32756 fis, clone TESTI2001758. [AK057318] |
| ZNF8 | NM_021089 | zinc finger protein 8 (ZNF8), mRNA [NM_021089] |
| TAF5L | NM_001025247 | TAF5-like RNA polymerase II, p300/CBP-associated factor (PCAF)-associated factor, 65kDa (TAF5L), transcript variant 2, mRNA [NM_001025247] |
| THC2691182 | THC2691182 | tc|BC060845 l(3)mbt-like 3, isoform b {Homo sapiens} (exp=-1; wgp=0; cg=0), partial (8%) [THC2691182] |
| KLHL15 | NM_030624 | kelch-like 15 (Drosophila) (KLHL15), mRNA [NM_030624] |
| GJB7 | NM_198568 | gap junction protein, beta 7, 25kDa (GJB7), mRNA [NM_198568] |
| MYH8 | NM_002472 | myosin, heavy chain 8, skeletal muscle, perinatal (MYH8), mRNA [NM_002472] |
| FLJ16734 | AK131514 | gb|Homo sapiens cDNA FLJ16734 fis, clone BRACE2002589. [AK131514] |
| C12orf66 | AK095383 | gb|Homo sapiens cDNA FLJ38064 fis, clone CTONG2015204. [AK095383] |
| XLOC_002918 | TCONS_00006308 | linc|BROAD Institute lincRNA (XLOC_002918), lincRNA [TCONS_00006308] |
| LRRD1 | ENST00000343318 | ens|leucine-rich repeats and death domain containing 1 [Source:HGNC Symbol;Acc:34300] [ENST00000343318] |
| XLOC_l2_008252 | ENST00000375987 | tc|Q658I5_ORYSA (Q658I5) LMBR1 integral membrane family protein-like, partial (4%) [THC2624432] |
| KIAA1456 | NM_020844 | KIAA1456 (KIAA1456), transcript variant 1, mRNA [NM_020844] |
| BBS1 | ENST00000529955 | ens|Bardet-Biedl syndrome 1 [Source:HGNC Symbol;Acc:966] [ENST00000529955] |
| XLOC_013702 | TCONS_00028351 | linc|BROAD Institute lincRNA (XLOC_013702), lincRNA [TCONS_00028351] |
| XLOC_001501 | AW272487 | gb|xv14h03.x1 Soares_NFL_T_GBC_S1 Homo sapiens cDNA clone IMAGE:2813141 3', mRNA sequence [AW272487] |
| XLOC_000232 | TCONS_00002048 | linc|BROAD Institute lincRNA (XLOC_000232), lincRNA [TCONS_00002048] |
| XLOC_012269 | TCONS_00025946 | linc|BROAD Institute lincRNA (XLOC_012269), lincRNA [TCONS_00025946] |
| SNORD126 | NR_003693 | small nucleolar RNA, C/D box 126 (SNORD126), small nucleolar RNA [NR_003693] |
| LOC100505679 | NM_001243531 | uncharacterized LOC100505679 (LOC100505679), mRNA [NM_001243531] |
| LOC647020 | XM_001719614 | gb|PREDICTED: Homo sapiens hypothetical protein LOC647020 (LOC647020), mRNA [XM_001719614] |
| XLOC_006182 | TCONS_00013543 | linc|BROAD Institute lincRNA (XLOC_006182), lincRNA [TCONS_00013543] |
| TMED11P | NR_033768 | transmembrane emp24 protein transport domain containing 11, pseudogene (TMED11P), non-coding RNA [NR_033768] |
| FAM188B | NM_032222 | family with sequence similarity 188, member B (FAM188B), mRNA [NM_032222] |
| MT1E | NM_175617 | metallothionein 1E (MT1E), mRNA [NM_175617] |
| LOC339400 | AK056431 | gb|Homo sapiens cDNA FLJ31869 fis, clone NT2RP7002151. [AK056431] |
| TTC9C | ENST00000294161 | ens|tetratricopeptide repeat domain 9C [Source:HGNC Symbol;Acc:28432] [ENST00000294161] |
| XLOC_006411 | TCONS_00013778 | linc|BROAD Institute lincRNA (XLOC_006411), lincRNA [TCONS_00013778] |
| ENST00000411845 | ENST00000411845 | ens|Putative PERP-like protein [Source:UniProtKB/Swiss-Prot;Acc:A6NN79] [ENST00000411845] |
| KRT2 | NM_000423 | keratin 2 (KRT2), mRNA [NM_000423] |
| ZXDB | NM_007157 | zinc finger, X-linked, duplicated B (ZXDB), mRNA [NM_007157] |
| PAM | NM_001177306 | peptidylglycine alpha-amidating monooxygenase (PAM), transcript variant 5, mRNA [NM_001177306] |
| LOC100507616 | XR_110328 | ref|PREDICTED: Homo sapiens hypothetical LOC100507616 (LOC100507616), miscRNA [XR_110328] |
| HMOX1 | NM_002133 | heme oxygenase (decycling) 1 (HMOX1), mRNA [NM_002133] |
| XLOC_l2_001064 | TCONS_l2_00001461 | linc|BROAD Institute lincRNA (XLOC_l2_001064), lincRNA [TCONS_l2_00001461] |
| A_33_P3421007 | A_33_P3421007 | Unknown |
| HIST1H3D | NM_003530 | histone cluster 1, H3d (HIST1H3D), mRNA [NM_003530] |
| AY358225 | AY358225 | gb|Homo sapiens clone DNA86575 GRTR3118 (UNQ3118) mRNA, complete cds. [AY358225] |
| XLOC_010621 | ENST00000443679 | gb|RST14406 Athersys RAGE Library Homo sapiens cDNA, mRNA sequence [BG195226] |
| ENST00000446592 | ENST00000446592 | gb|Homo sapiens coiled-coil domain containing 26, mRNA (cDNA clone IMAGE:4305364). [BC070152] |
| TPTE2P3 | NR_002793 | transmembrane phosphoinositide 3-phosphatase and tensin homolog 2 pseudogene 3 (TPTE2P3), non-coding RNA [NR_002793] |
| XLOC_003521 | ENST00000510602 | linc|BROAD Institute lincRNA (XLOC_003521), lincRNA [TCONS_00007520] |
| USP25 | NM_013396 | ubiquitin specific peptidase 25 (USP25), mRNA [NM_013396] |
| DMTF1 | NM_021145 | cyclin D binding myb-like transcription factor 1 (DMTF1), transcript variant 1, mRNA [NM_021145] |
| XLOC_010113 | BG190604 | gb|RST9677 Athersys RAGE Library Homo sapiens cDNA, mRNA sequence [BG190604] |
| XLOC_004086 | ENST00000511234 | linc|BROAD Institute lincRNA (XLOC_004086), lincRNA [TCONS_00007840] |
| XLOC_l2_010386 | ENST00000471222 | gb|RST23433 Athersys RAGE Library Homo sapiens cDNA, mRNA sequence [BG204039] |
| SATB2 | NM_015265 | SATB homeobox 2 (SATB2), transcript variant 2, mRNA [NM_015265] |
| XLOC_001332 | ENST00000442864 | gb|AV688451 GKC Homo sapiens cDNA clone GKCAUE06 5', mRNA sequence [AV688451] |
| GPR116 | NM_001098518 | G protein-coupled receptor 116 (GPR116), transcript variant 2, mRNA [NM_001098518] |
| LOC285370 | NR_027102 | uncharacterized LOC285370 (LOC285370), non-coding RNA [NR_027102] |
| XLOC_001457 | CD513615 | gb|AGENCOURT_14366553 NIH_MGC_179 Homo sapiens cDNA clone IMAGE:30394729 5', mRNA sequence [CD513615] |
| SNRPE | NM_003094 | small nuclear ribonucleoprotein polypeptide E (SNRPE), mRNA [NM_003094] |
| XLOC_000595 | TCONS_00001315 | linc|BROAD Institute lincRNA (XLOC_000595), lincRNA [TCONS_00001315] |
| XLOC_006153 | TCONS_00013023 | linc|BROAD Institute lincRNA (XLOC_006153), lincRNA [TCONS_00013023] |
| H19 | NR_002196 | H19, imprinted maternally expressed transcript (non-protein coding) (H19), non-coding RNA [NR_002196] |
| LOX | NM_002317 | lysyl oxidase (LOX), transcript variant 1, mRNA [NM_002317] |
| C11orf92 | NR_034154 | chromosome 11 open reading frame 92 (C11orf92), non-coding RNA [NR_034154] |
| ENST00000409061 | ENST00000409061 | ens|ankyrin repeat domain 61 [Source:HGNC Symbol;Acc:22467] [ENST00000409061] |
[truncated: 222,839 more chars]
